# An individual-based modelling study estimating the impact of maternity service delivery on health in Malawi

**DOI:** 10.1038/s41467-025-59060-2

**Published:** 2025-04-25

**Authors:** Joseph H. Collins, Helen Allott, Wingston Ng’ambi, Ines Li Lin, Mosè Giordano, Matthew M. Graham, Eva Janoušková, Fannie Kachale, Kondwani Kawaza, Tara D. Mangal, Joseph Mfutso-Bengo, Emmanuel Mnjowe, Sakshi Mohan, Margherita Molaro, Dominic Nkhoma, Paul Revill, Alison Rodger, Bingling She, Asif U. Tamuri, Cally J. Tann, Pakwanja D. Twea, Valentina Cambiano, Timothy B. Hallett, Andrew N. Phillips, Tim Colbourn

**Affiliations:** 1 Institute for Global Health, University College London, London, UK; 2 International Public Health Department, Liverpool School of Tropical Medicine, Liverpool, UK; 3 Kamuzu University of Health Sciences, Lilongwe, Malawi; 4 Centre for Advanced Research Computing, University College London, London, UK; 5 Reproductive Health Department, Malawi Ministry of Health, Lilongwe, Malawi; 6 Queen Elizabeth Central Hospital, Blantyre, Malawi; 7 MRC Centre for Global Infectious Disease Analysis, Jameel Institute, School of Public Health, Imperial College London, London, UK; 8 Centre for Health Economics, University of York, York, UK; 9 Department of Infectious Disease Epidemiology and International Health, London School of Hygiene and Tropical Medicine, London, UK; 10 Department of Planning and Policy Development, Malawi Ministry of Health, Lilongwe, Malawi

**Keywords:** Health policy, Epidemiology, Epidemiology

## Abstract

Maternal and perinatal morbidity and mortality remain high in Malawi, partially due to gaps in the coverage and quality of health services. We developed an individual-based model of maternal and perinatal health and healthcare in Malawi, situated in a ‘whole-health system, all-disease’ framework (*Thanzi La Onse*). We modelled sixteen scenarios estimating the impact of current and improved coverage and quality of antenatal, intrapartum, and postnatal services from 2023 to 2030. Whilst current service delivery is inferred to avert morbidity and mortality, the largest reductions in the stillbirth, maternal and neonatal mortality rates were observed when the use and quality of all services was maximised concurrently (a 10%, 52% and 57% reduction respectively). When services were considered in isolation, generally, increased coverage without quality improvement did not impact mortality or DALYs. In only three scenarios was a sufficient reduction in neonatal mortality observed to achieve target 3.2 of the United Nation’s Sustainable Development Goals (SDG), and in no scenarios was a reduction in maternal mortality sufficient to achieve SDG target 3.1 observed, reaffirming that system wide investments are essential to achieve these goals.

## Abstract (web summary)

Maternal and perinatal morbidity and mortality remain high in Malawi, partially due to gaps in the coverage and quality of health services. Here the authors develop an individual-based simulation model of maternal and perinatal health and healthcare in Malawi, situated in the Thanzi La Onse ‘whole health system, all-disease’ framework, and apply it to estimate the impact of current and improved coverage and quality of maternity services on health in Malawi between 2023 and 2030.

## Introduction

Globally, substantial progress in improving both the health of, and healthcare access for, pregnant and postnatal women and their newborns has been achieved^1–3^. However, there remains significant inequality between nations demonstrated by the rates of maternal and perinatal morbidity and mortality, with the greatest burden concentrated in sub-Saharan Africa (SSA)^1–3^. This can partly be attributed to the complex interplay between insufficient access to, and availability of, high-quality maternity services^4–9^, a high burden of communicable disease such as HIV and Malaria^10^ and an increasing incidence of non-communicable disease^11^, many of which are evidenced to exacerbate poor maternal and perinatal outcomes^12–15^.

Malawi is one such country in SSA in which the population-level maternal mortality ratio (MMR), neonatal mortality rate (NMR) and stillbirth rate (SBR) are amongst the highest in the world^1–3^ and when combined, maternal and neonatal conditions are the leading contributor of Disability Adjusted Life Years (DALYs) in the country, accounting for 1,033,328 (Uncertainty Interval (UI) [811,533, 1,331,149]) DALYs in 2019^10^. Whilst there have been marked improvements in the availability and utilisation of essential maternity services in Malawi^16–19^, population level coverage and quality of such services remains low^19–21^. Currently 91% of women give birth within a healthcare facility^19^, however the uptake of routine antenatal and postnatal care services, essential for the identification of disability causing and/or life-threatening complications, remains below recommended levels set within World Health Organization (WHO) guidelines and recent global maternal and newborn health strategy targets^19,22,23^. Only 51% of women attend four or more antenatal care visits^19^, compared to a global target defined by the WHO and partners in the recent Ending Preventable Maternal Mortality (EPMM) strategy of 90% coverage to be achieved by 2025^22,23^. Similarly, only 42% of mothers and 60% of newborns receive postnatal care (PNC) within two days of birth^19^, compared to a target of 80% coverage globally defined in both the EPMM strategy and the Every Newborn Action Plan (ENAP)^22,23^.

Additionally, both recent sector-wide evaluation of national health facilities^21,24^ and direct observation of care delivered^20,21,25^ highlight the significant gaps in the quality of services provided, due to issues such as insufficient availability of trained healthcare workers and essential resources or equipment. Furthermore, maternal death reviews, in which the medical records of mothers who have died are independently reviewed by clinicians to certify cause of death and contributory factors, suggest that both a lack of availability of services and poor-quality service delivery contribute directly to poor maternal outcomes, including maternal death^26,27^.

Given the longstanding commitment from within the Malawian Government and Ministry of Health (MoH) to improve maternal and neonatal health^28–30^, the prominence of maternal and neonatal health within the Sustainable Development Goals (SDG) health-related targets (targets 3.1 and 3.2), and the limited recent progress in reducing mortality within this population, especially within mothers^3^, there is urgent need to develop initial estimates of the impact of closing aforementioned gaps in coverage and quality on health. Importantly, such estimates cannot feasibly be determined via experimentation due to ethical or practical constraints, and therefore mathematical modelling is best placed to predict the effect of changes to service delivery^31^. Whilst strategies such as EPMM^22^ and ENAP^23^ offer policy makers clear targets for antenatal, intrapartum, and postnatal service coverage at the national level, the potential health impacts of achieving these targets, within the context of current health system constraints (such as availability of drugs, other consumables, and quality of clinical care), remain unexplored in Malawi. Beyond this, whilst evidence suggests that in Malawi and other settings maternal and neonatal mortality is clustered around birth and the immediate postnatal period^24,32^, the isolated impact of improved coverage of these services is also unexplored along with the total potential health gains associated with ‘perfect’ service delivery, beyond current levels defined in global targets.

Here, we present a novel stochastic individual-based simulation model of maternal and perinatal health and healthcare utilisation within Malawi. The model is used to estimate the potential impact of current and improved maternity service delivery on a range of health and health system outcomes including morbidity, mortality, DALYs and healthcare worker time requirements. This model sits within the Thanzi La Onse (TLO) modelling framework, which simulates a demographically representative population of Malawi, the diseases contributing approximately 81% of the total deaths and their interactions; and represents the delivery of health services. An overview of the TLO model is available elsewhere^33^. Results from this analysis suggest that current delivery of maternity services is inferred to avert substantial morbidity and mortality at the population level during the simulation period (2023–2030) whilst highlighting that there is scope to further improve health through high coverage of high-quality services. We found that isolated improvements in service coverage without associated improvements in quality had limited impact on mortality whilst policies which seek to improve high quality service delivery across the pregnancy continuum are most likely needed to ensure Malawi can achieve SDG targets 3.1 and 3.2 by 2030.

## Results

Counterfactual scenarios were developed, simulated, and compared to a scenario of current assumed levels of coverage and quality of health services in Malawi, referred to here as a Status Quo (SQ) scenario. Broadly, counterfactual scenarios were developed to predict the effect of the following: improved coverage of antenatal, intrapartum, or postnatal services without any changes in quality from the SQ, improved coverage and quality of these services compared to the SQ, maximum coverage and quality of these services compared to the SQ. In addition, theoretical scenarios assuming none of these services were provided at all, were simulated to allow for estimation of the effect of the current levels of coverage and quality on outcomes. All modelled scenarios are summarised in Table 1.

**Table 1:** Scenarios modelled to determine the effect of current and improved antenatal, intrapartum, and postnatal healthcare services on maternal and perinatal health in Malawi

	Scenario full name (Short name)	Scenario description	Changes to service delivery during intervention period (2023–2030)
Comparator scenario:	Status Quo (SQ)	The coverage and quality of maternity services remain unchanged from assumed levels in Malawi during the intervention period. Population level coverage of four or more antennal care (ANC) visits is ~51%, facility delivery is ~91%, maternal postnatal care (PNC) is 42% and newborn PNC is 60% (see S1 File, §2, §4). This scenario acts as the comparator for all other scenarios within this table.	N/A
Scenarios relating to antenatal services:	Increased routine ANC coverage (AN coverage)	The population level coverage of routine ANC (four or more visits) is increased during the intervention period with no changes to service quality.	Changes in service utilisation/coverage: • 90% of women who deliver per year receive four or more ANC contacts Changes in service quality: • None Otherwise same as the Status Quo.
	Increased coverage and quality of antenatal services (AN coverage and qual.)	The population level coverage of routine ANC (four or more visits) is increased during the intervention period in addition to maximum quality in service delivery (e.g. all required interventions are delivered). In addition, the quality of inpatient antenatal services is set at maximum. There are no modelled changes to care delivered following termination of pregnancy (e.g. post abortion care/ectopic pregnancy case management).	Changes in service utilisation/coverage: • 90% of women who deliver per year receive four or more ANC contacts Changes in service quality: • All parameters representing the probability an intervention (e.g. initiation of iron and folic acid) may occur during ANC are set to 1.0 (as a proxy for quality). • All requested consumables (i.e. medicines) for ANC are available within a given health system interaction^‖^ (HSI). • All parameters representing the probability an intervention may occur during inpatient antenatal care as a proxy for quality set to 1.0. • All requested consumables for inpatient antenatal care are available within a given HSI. • The effect of delay three^a^ is disabled during inpatient antenatal care meaning perfect availability of health care worker (HCW) time is assumed leading to no effect of the ‘squeezed’ health system on treatment effectiveness during care. Otherwise same as the Status Quo.
	Maximum availability of antenatal services (AN max.)	The delivery of antenatal services within the pregnant population is maximised during the intervention period. This means that alongside all women receiving the maximum number of routine ANC contacts possible during their pregnancy, all care seeking for antenatal emergencies occurs and all required antenatal interventions are delivered. There are no modelled changes to care delivered following termination of pregnancy (e.g. post abortion care/ectopic pregnancy case management).	Changes in service utilisation/coverage: • All newly pregnant women during the intervention period initiate ANC within the first trimester. • Probability of attending any subsequent ANC visit set to 1.0 leading to maximum number of visits for a given pregnancy (dependent on pregnancy loss, preterm labour, or antenatal death). • Probability of maternal care seeking for antenatal emergencies (excluding those related to pregnancy loss) is set to 1.0. Changes in service quality: • All parameters representing the probability an intervention may occur during ANC as a proxy for quality set to 1.0. • All requested consumables for ANC are available within a given interaction. • All parameters representing the probability an intervention may occur during inpatient antenatal care as a proxy for quality set to 1.0. • All requested consumables for inpatient antenatal care are available within a given HSI. • The effect of delay three is disabled during inpatient antenatal care meaning perfect availability of health care worker (HCW) time is assumed leading to no effect of the ‘squeezed’ health system on treatment effectiveness during care. Otherwise same as the Status Quo.
	No antenatal services (AN min.)	Antenatal services are not delivered to the population during the intervention period. This allows for estimation of the effect of current delivery of antenatal services on the population in the SQ scenario.	Changes in service utilisation/coverage: • No routine ANC occurs for any newly pregnant woman during the intervention period. • Probability of maternal care seeking for antenatal emergencies (excluding those related to pregnancy loss) is set to 0 meaning no antenatal emergency inpatient care is delivered. Otherwise same as the Status Quo.
Scenarios relating to intrapartum services:	Increased availability of intrapartum Basic Emergency Obstetric and Newborn Care (BEmONC) interventions (IP BEmONC)	The availability of prophylactic and BEmONC interventions^b^ delivered to women in labour is increased.	Changes in service quality: • Probability of prophylactic intervention^c^ delivery during labour set at 90% • Probability of BEmONC intervention delivery during labour set at 90% • In addition, effect of delay three is disabled meaning perfect availability of health care worker (HCW) time is assumed leading to no effect of the ‘squeezed’ health system on treatment effectiveness during care. Otherwise same as the Status Quo.
	Increased availability of intrapartum Comprehensive Emergency Obstetric and Newborn Care (CEmONC) interventions (IP CEmONC)	The availability of prophylactic and CEmONC interventions^d^ delivered to women in labour is increased.	Changes in service quality: • Probability of prophylactic intervention delivery during labour set at 90% • Probability of BEmONC intervention delivery during labour set at 90% • Probability of CEmONC intervention delivery during labour set at 90% • In addition, effect of delay three is disabled meaning perfect availability of health care worker (HCW) time is assumed leading to no effect of the ‘squeezed’ health system on treatment effectiveness during care. Otherwise same as the Status Quo.
	Maximum availability of intrapartum services (IP max).	The delivery of intrapartum services within the pregnant population is maximised during the intervention period. This means that alongside all women giving birth in a health facility, all required interventions are delivered.	Changes in service utilisation/coverage: • All women will deliver in either a health centre or a hospital during the intervention period. Changes in service quality: • Probability of prophylactic intervention delivery during labour set at 100% • Probability of BEmONC intervention delivery during labour set at 100% • Probability of CEmONC intervention delivery during labour set at 100% • In addition, effect of delay three is disabled meaning perfect availability of health care worker (HCW) time is assumed leading to no effect of the ‘squeezed’ health system on treatment effectiveness during care. Otherwise same as the Status Quo
	No intrapartum services (IP min.)	Intrapartum services are not delivered to the population during the intervention period. This allows for estimation of the effect of current delivery of intrapartum services on the population in the SQ scenario.	Changes in service quality: • Probability of prophylactic intervention delivery during labour set at 0. • Probability of BEmONC intervention delivery during labour set at 0. • Probability of CEmONC intervention delivery during labour set at 0. Otherwise same as the Status Quo
Scenarios relating to postnatal services:	Increased postnatal care (PNC) coverage (PN coverage)	The population level coverage of routine PNC is increased during the intervention period with no changes to service quality.	Changes in service utilisation/coverage: • 90% of women and neonates who deliver per year receive one or more postnatal care visits. The first visit occurs within 48 h of delivery. • Otherwise same as the Status Quo
	Increased coverage and quality of postnatal services (PN coverage and qual.)	The population level coverage of routine PNC is increased during the intervention period in addition to maximum quality in service delivery (e.g. all required interventions are delivered). In addition, the quality of inpatient postnatal services is also maximised.	Changes in service utilisation/coverage: • 90% of women and neonates who deliver per year receive one or more postnatal care visits. The first visit occurs within 48 h of delivery. Changes in service quality: • All parameters representing the probability an intervention may occur during routine PNC as a proxy for quality set to 1.0. • All consumables for PNC are available within a given HSI. • All parameters representing the probability an intervention may occur during inpatient postnatal care as a proxy for quality set to 1.0 • All consumables for inpatient postnatal care available within a given interaction. • The effect of delay three is disabled during inpatient postnatal care meaning perfect availability of health care worker (HCW) time is assumed leading to no effect of the ‘squeezed’ health system on treatment effectiveness during care. Otherwise same as the Status Quo
	Maximum availability of postnatal services (PN max).	The delivery of postnatal services within the pregnant population is maximised during the intervention period. This means that alongside all women and newborns receiving early PNC, all required interventions are delivered. Additionally, all women and newborns with complications later in the postnatal period will seek and receive care.	Changes in service utilisation/coverage: • 100% of women and neonates who deliver per year receive their first PNC visit within 48 h of delivery. Additionally, any future PNC visits required (due to complications) are 100% likely to occur Changes in service quality: • All parameters representing the probability an intervention may occur during routine PNC as a proxy for quality set to 1.0. • All consumables for PNC are available within a given HSI. • Probability of maternal or neonatal care seeking for postnatal emergencies is set to 1.0. • All parameters representing the probability an intervention may occur during inpatient postnatal care as a proxy for quality set to 1.0. • All consumable for inpatient postnatal care available within a given interaction. • In addition, effect of delay three is disabled meaning perfect availability of health care worker (HCW) time is assumed leading to no effect of the ‘squeezed’ health system on treatment effectiveness during care. Otherwise same as the Status Quo
	No postnatal services (PN min.)	Postnatal services are not delivered to the population during the intervention period. This allows for estimation of the effect of current delivery of postnatal services on the population in the SQ scenario.	Changes in service utilisation/coverage: • No routine PNC occurs for any mother or newborn after birth. Changes in service quality: • Probability of maternal and neonatal care seeking for postnatal emergencies is set to 0, meaning no postnatal emergency inpatient care is delivered. Otherwise same as the Status Quo
Scenarios relating to all services:	Increased coverage and antenatal, postnatal services and improved availability of BEmONC interventions (All services coverage).	The changes to coverage of the services described in the scenarios AN coverage., IP BEmONC, and PN coverage., are enacted during the intervention period.	See scenarios AN coverage., IP BEmONC, and PN coverage.
	Increased coverage and quality of antenatal, postnatal services, and improved availability of CEmONC interventions (All services coverage and qual.)	The changes to coverage and quality of the services described in the scenarios AN coverage and qual., IP CEmONC, and PN coverage and qual., are enacted during the intervention period.	See scenarios AN coverage and qual., IP CEmONC, and PN coverage and qual.
	Maximum availability of antenatal, intrapartum, and postnatal services (All services max).	The changes to coverage and quality of the services described in the scenarios AN max., IP max., and PN max. are enacted during the intervention period.	See scenarios AN max., IP max., and PN max.
	No availability of antenatal, intrapartum, and postnatal services (All services min).	The changes to the availability of the services described in the scenarios AN min., IP min., and PN min. are enacted during the intervention period.	See scenarios AN min., IP min., and PN min.

‖A health system interaction is a discrete event in the simulation in which healthcare is delivered. Interventions are housed within HSIs defined by the level of facility at which they occur and the required healthcare worker time. This is described in the methodology section.

^a^Thaddeus and Maine’s (74) three delays are modelled as described in the methodology section and S1 File. Delay three is assumed to occur due to overdemand of available healthcare worker time, resulting in a ‘squeeze’ of time devoted to health services thereby leading to reduced treatment effectiveness.

^b^Parenteral antibiotics, Uterotonic drugs, Parenteral anticonvulsants, Assisted Vaginal Delivery, Neonatal resuscitation.

^c^Clean birth practices and intravenous corticosteroids.

^d^BEmONC interventions plus blood transfusion, obstetric surgery including caesarean section.

### Model calibration to epidemiological and health system data

To ensure the model sufficiently represents the SQ in Malawi, key outputs from the model between 2011 and 2022 were compared to both epidemiological and health systems data from the country. Figure 1 compares model outputs related to maternal and perinatal mortality with estimates from Malawi, showing a good agreement between the model and data sources. Additional figures comparing other outputs, including DALYs and health service coverage, to data sources from Malawi are shown in Figs S33–S46 in the supporting information (S1 File). Figures S47–S79 show the annual incidence/prevalence of all modelled conditions compared to data from Malawi or similar setting when Malawian data was unavailable. Finally, Table S61 reports the total pregnancies, births, stillbirths, maternal deaths and neonatal deaths for the SQ scenario.

**Fig. 1:**
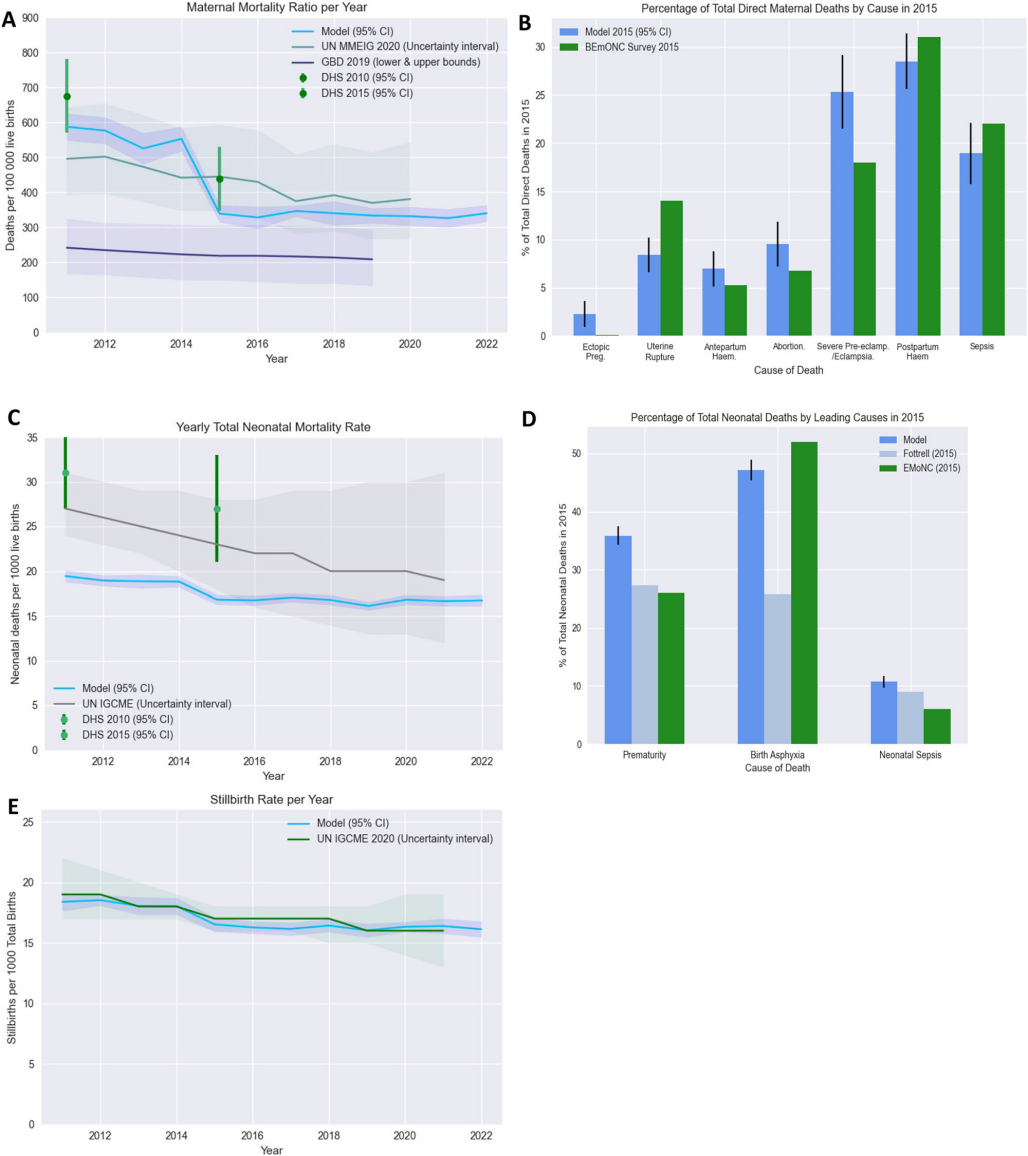
Model calibration to estimates of maternal and perinatal mortality within Malawi. **A**^±^ The mean MMR (95% CI) across 20 simulation runs per year outputted from the model (shown in blue) is plotted against estimates of MMR produced by the Global Burden of Disease (GBD) group (purple), the UN Maternal Mortality Estimation Inter-Agency Group (grey) and the Malawi Demographic and Health Survey estimates from 2010 and 2015 (green). **B** The mean percentage of total direct maternal deaths by cause in 2015 are compared to estimates from Malawi’s most recent national EmONC needs assessment survey (green) in which all direct deaths were recorded over at twelve-month observation period. **C**^±^ The mean NMR (95% CI) across 20 simulation runs per year (blue) is plotted against estimates of NMR produced by the UN Inter-agency Group for Child Mortality Estimation (IGME) (grey) and the DHS (green). **D** The mean percentage of total neonatal deaths by leading causes in 2015 are compared to estimates from Malawi’s most recent national EmONC needs assessment survey (green) and prospective surveillance study conducted in two Malawian sites by Fottrell et al. (grey). **E**^±^ The mean SBR (95% CI) across 20 simulation runs per year (blue) is plotted against estimates of SBR produced by the UN IGCME (green). Model calibration is discussed further in the supporting information. (^±^The first year of the simulation, 2010, acts as a ‘burn-in’ period where outputs of the model stabilise and therefore are not presented. DHS data from reports published in 2010 is therefore plotted in 2011).

### Impact of maternity service delivery on mortality and DALYs

**Fig. 2:**
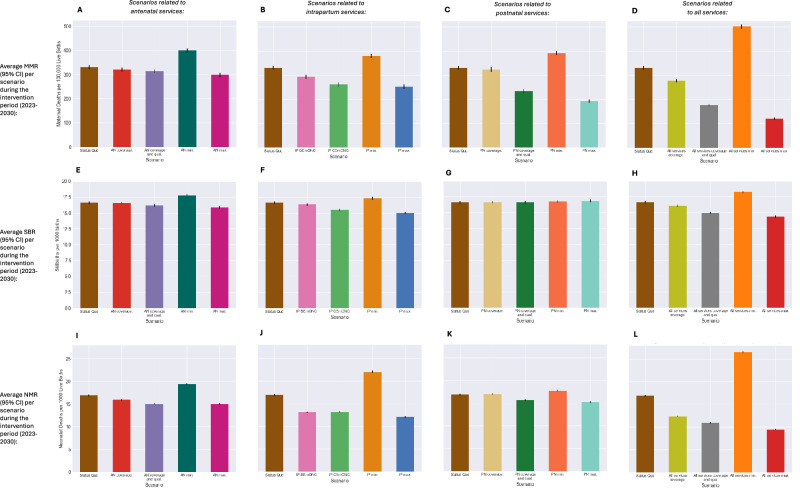
Maternal and neonatal mortality and stillbirth rates by scenario. Scenarios relating to antenatal, intrapartum and postnatal services share a colour scheme with each scenario represented by a unique colour—this is maintained throughout the manuscript and figures in supporting information. **A**–**D** The average MMR across the intervention period (2023–2030) is provided for each scenario alongside 95% CI. **E**–**H** The average SBR across the intervention period (2023–2030) is provided for each scenario alongside 95% CI. **I**–**L** The average NMR across the intervention period (2023–2030) is provided for each scenario alongside 95% CI.

**Fig. 3:**
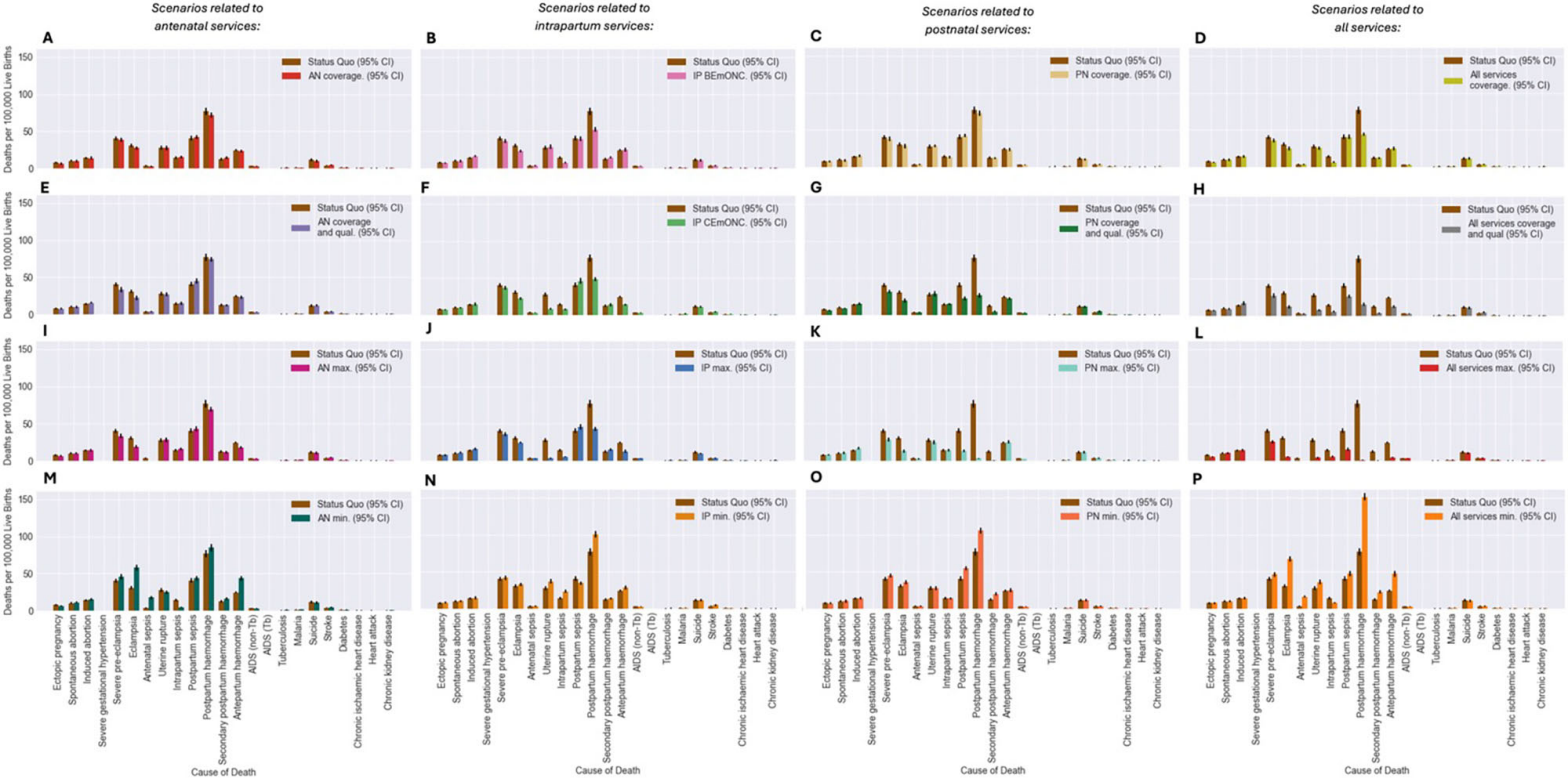
Cause-specific MMRs for each of the potentially fatal modelled maternal complications by scenario for each of the four analyses conducted. **A**, **E**, **I**, **M** The average cause specific MMRs across the intervention period (2023–2030) is provided for each complication alongside 95% CI for scenarios related to antenatal services. **B**, **F**, **J**, **N** The average cause specific MMRs across the intervention period (2023–2030) is provided for each complication alongside 95% CI for scenarios related to intrapartum services. **C**, **G**, **K**, **O** The average cause specific MMRs across the intervention period (2023–2030) is provided for each complication alongside 95% CI for scenarios related to postnatal services. **D**, **H**, **L**, **P** The average cause specific MMRs across the intervention period (2023–2030) is provided for each complication alongside 95% CI for scenarios related to all services.

**Fig. 4:**
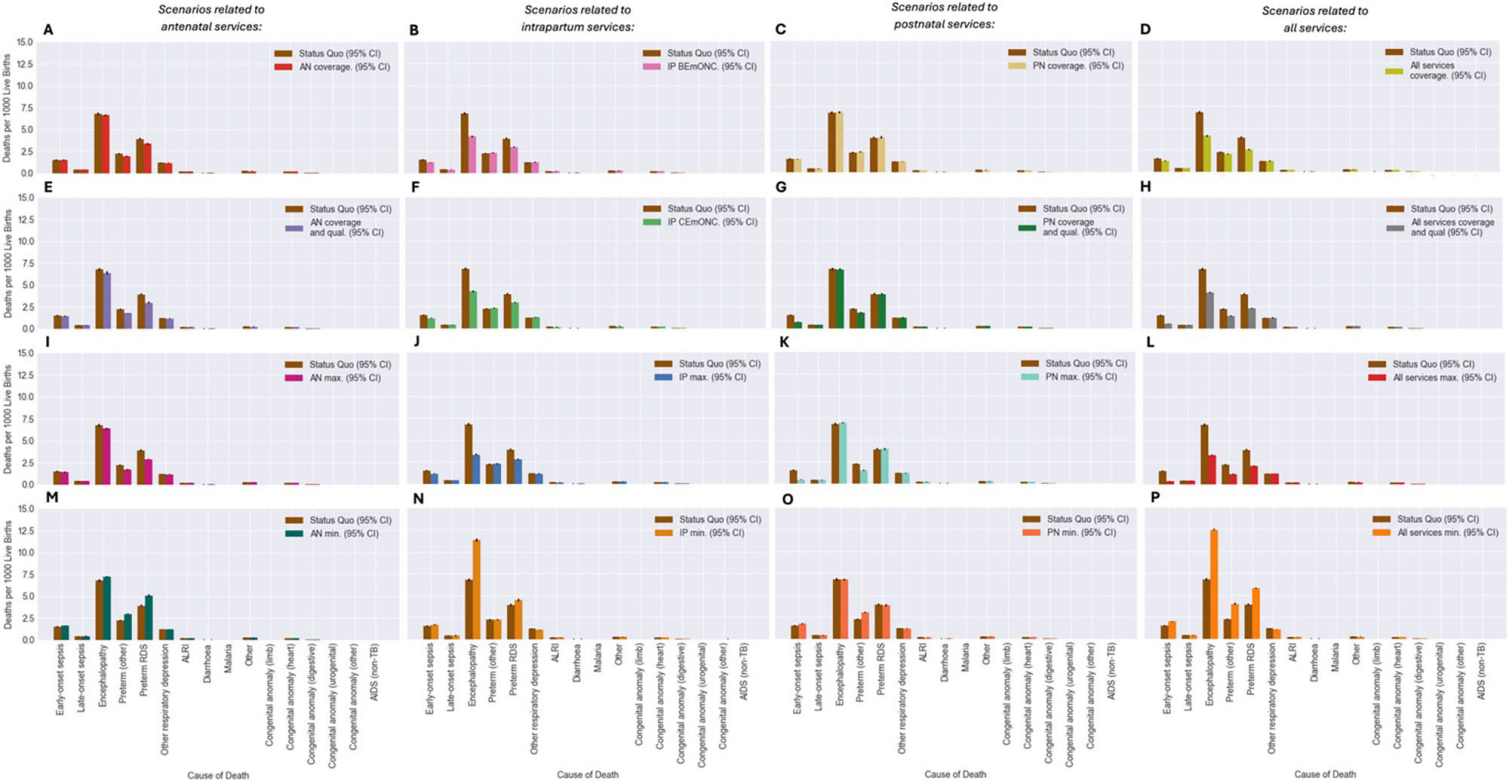
Cause-specific NMRs for each of the potentially fatal modelled maternal complications by scenario for each of the four analyses conducted. **A**, **E**, **I**, **M** The average cause specific NMRs across the intervention period (2023–2030) is provided for each complication alongside 95% CI for scenarios related to antenatal services. **B**, **F**, **J**, **N** The average cause specific NMRs across the intervention period (2023–2030) is provided for each complication alongside 95% CI for scenarios related to intrapartum services. **C**, **G**, **K**, **O** The average cause specific NMRs across the intervention period (2023–2030) is provided for each complication alongside 95% CI for scenarios related to postnatal services. **D**, **H**, **L**, **P** The average cause specific NMRs across the intervention period (2023–2030) is provided for each complication alongside 95% CI for scenarios related to all services.

Figure 2 shows the average MMR, NMR and SBR during the intervention period (2023–2030) for each modelled scenario. Tables 2–4 report the values for these rates, in addition to the total number of DALYs attributable to maternal and neonatal disorders, by scenario and the mean and percentage difference between each scenario and the SQ. Figures 3, 4 show the cause-specific maternal and neonatal mortality rates by scenario. Additionally, within the supporting information, Figure S80 shows the mean rate and 95% CI for these metrics over time. Scenarios are referred to by their short names first presented in Table 1.

**Table 2:** Average MMR, total maternal deaths and total DALYs due to ‘Maternal Disorders’ during the intervention period (2023–2030) by scenario

	Scenario full name (Short name)	Average MMR (95% CI) Mean difference from SQ (95% CI) Percentage difference from SQ^a^	Total maternal deaths (95% CI) Mean difference from SQ (95% CI) Percentage difference from SQ^a^	Total Maternal DALYs (95% CI) Mean difference from SQ (95% CI) Percentage difference from SQ^a^
Comparator scenario:	Status Quo (SQ)	330.5 (322.4, 338.6) Ref.	19,541 (19,066, 20,016) Ref.	795,868 (774,804, 816,933) Ref.
Scenarios relating to antenatal services:	Increased routine ANC coverage (AN coverage)	320.0 (312.1, 327.9) –10.5 (–22.1, 1.0) N/A	18,922 (18,450, 19,394) –618 (–1304, 66) N/A	771,773 (751,356, 792,109) –24,095 (–53,107, 4916) N/A
	Increased coverage and quality of antenatal services (AN coverage and qual.)	314.4 (307.0, 321.9) –16.1(–28.2, –4.0) –4.9 %	18,609 (18,173, 19,044) –932 (–1655, –208) –4.8%	755,897 (737,837, 773,956) –39,971 (–70,865, –9078) –5.0%
	Maximum availability of antenatal services (AN max.)	298.1 (289.2, 307) –32.4 (–46.5, –18.3) –9.8%	17,628 (17,123, 18,134) –1913 (–2738, –1087) –9.8%	711,742 (692,539, 730,946) –84,126 (–116,389, –51,863) –10.6%
	No antenatal services (AN min.)	399.4 (391.0, 407.8) 68.9 (55.9, 82.2) 20.8%	23,595 (23,116, 24,074) 4054 (3278, 4831) 20.8%	976,468 (959,658, 993,278) 180,600 (149,942, 211,257) 22.7%
Scenarios relating to intrapartum services:	Increased availability of intrapartum BEmONC interventions (IP BEmONC)	293.5 (285.6, 301.4) –37.0 (–47.8, –26.3) –11.2%	17,361 (16,898, 17,823) –2180 (–2812, –1549) –11.2%	700,516 (682,721, 718,311) –95,352 (–119,811, –70,893) –12.0%
	Increased availability of intrapartum CEmONC interventions (IP CEmONC)	261.1 (252.7, 269.5) –69.4 (–80.3, –58.4) –21.0%	15,482 (14,991, 15,973) –4059 (–4683, –3434) –20.8%	627,728 (607,760, 647,696) –168,140 (–194,105, –142,176) –21.1%
	Maximum availability of intrapartum services (IP max).	252.3 (243.1, 261.5) –78.3 (–89.1, –67.4) –23.7%	14,988 (14,439, 15,537) –4553 (–5189, –3918) –23.3%	604,284 (583,308, 625,260) –191,585 (–216,665, –166,504) –24.1%
	No intrapartum services (IP min.)	379.7 (371.0, 388.4) 49.2 (37.1, 61.3) 14.9%	22,457 (21,953, 22,962) 2913 (2222, 3611) 14.9%	914,736 (894,337, 935,135) 118,868 (90,126, 147,610) 14.9%
Scenarios relating to postnatal services:	Increased PNC coverage (PN coverage)	322.2 (312.4, 332.0) –8.3 (–21.2, 4.49) N/A	19,087 (18,494, 19,680) –454.0(–1220, 312) N/A	781,925 (758,710, 805,140) –13,943 (–46,035, 18,149) N/A
	Increased coverage and quality of postnatal services (PN coverage and qual.)	233.9 (226.6, 241.1) –96.7 (–107.1, –86.3) –29.3%	13,854 (13,432, 14,276) –5687 (–6297, –5077) –29.1%	548,956 (532,992, 564,920) –246,913 (–270,748, –223,078) –31.0%
	Maximum availability of postnatal services (PN max).	191.7 (184.8, 198.7) –138.9 (–149.8, –127.8) –42.0%	11,359 (10,952, 11,766) –8182 (–8822, –7542) –41.9%	442,829 (426,546, 459,111) –353,040 (–379,183, –326,896) –44.4%
	No postnatal services (PN min.)	391.2 (383.5, 399.0) 60.7 (50.6, 70.7) 18.4%	23,126 (22,627, 23,581) 3586 (3000, 4171) 18.4%	948,241 (924,914, 971,569) 152,373 (124,651, 180,096) 19.2%
Scenarios relating to all services:	Increased coverage and antenatal, postnatal services and improved availability of BEmONC interventions (All services coverage)	277.6 (270.7, 284.6) –52.9 (–63.8, –42.0) –16.0%	16,450 (16,018, 16,883) –3091 (–3744, –2437) –15.8%	662,468 (643,887, 681,049) –133,400 (–163,950, –102,851) –16.8%
	Increased coverage and quality of antenatal, postnatal services, and improved availability of CEmONC interventions (All services coverage and qual.)	176.4 (170.8, 181.9) 154.2 (–162.7, –145.6) –46.6%	10,452 (10,122, 10,781) –9089 (–9605, –8573) –46.5%	403,699 (387,415, 419,984) –392,169 (–415,351, –368,988) –49.3%
	Maximum availability of antenatal, intrapartum, and postnatal services (All services max.)	120.0 (114.8, 125.2) –210.5 (–220.0, –201.1) –63.7%	7113 (6806, 7421) –12,427 (–12,983, –11,872) –63.6%	261,359 (251,506, 271,213) –534,509 (–556,750, –512,267) –67.2%
	No availability of antenatal, intrapartum, and postnatal services (All services min.)	501.8 (493.0, 510.7) 171.3 (161.2, 181.4) 51.8%	29,642 (29,108, 30,175) 10,101 (9514, 10,688) 51.7%	1,233,184 (1,210,792, 1,255,576) 437,316 (410,491, 464,141) 55.0%

^a^Percentage difference from the SQ is only presented for those scenarios in which the 95% confidence interval does not contain 0.

**Table 3:** Average SBR and total stillbirths during the intervention period (2023–2030) by scenario

	Scenario full name (short name)	Average SBR mean difference (95% CI) Percentage difference from SQ^a^	Total Stillbirths (95% CI) mean difference (95% CI). Percentage difference from SQ^a^
Comparator scenario:	Status Quo (SQ)	16.6 (16.4, 16.8) Ref.	98,358 (97,197, 99,518) Ref.
Scenarios relating to antenatal services:	Increased routine ANC coverage (AN coverage)	16.6 (16.4, 16.7) –0.1 (–0.3, 0.2) N/A	97,907 (97,020, 98,793) –451 (–1736, 835) N/A
	Increased coverage and quality of antenatal services (AN coverage and qual.)	16.2 (16.0, 16.4) –0.5 (–0.7, –0.2) –2.7%	95,743 (94,722, 96,765) –2614 (–4184, –1045) –2.7%
	Maximum availability of antenatal services (AN max.)	15.9 (15.7, 16.1) –0.7 (–1, –0.5) –4.4%	94,058 (92,826, 95,289) –4301 (–5858, –2743) –4.4%
	No antenatal services (AN min.)	17.8 (17.5, 18.0) 1.1 (0.8, 1.5) 6.8%	104,996 (103,627, 106,366) 6639 (4614, 8663) 6.8%
Scenarios relating to intrapartum services:	Increased availability of intrapartum BEmONC interventions (IP BEmONC)	16.3 (16.2, 16.5) –0.3 (–0.5, –0.1) –1.71%	96,752 (95,685, 97,820) –1605 (–2886, –324) –1.6%
	Increased availability of intrapartum CEmONC interventions (IP CEmONC)	15.5 (15.2, 15.7) –1.2 (–1.5, –0.8) –7.0%	91,768 (90,173, 93,363) –6589 (–8751, –4428) –6.7%
	Maximum availability of intrapartum services (IP max.)	15.0 (14.8, 15.2) –1.6 (–1.9, –1.4) –9.7%	89,169 (88,133, 90,204) –9189 (–10,625, –7753) –9.3%
	No intrapartum services (IP min.)	17.3 (17.1, 17.6) 0.7 (0.4, 1.0) 4.1%	102,394 (101,046, 103,741) 4036 (2458, 5615) 4.1%
Scenarios relating to all services:	Increased coverage and antenatal, postnatal services and improved availability of BEmONC interventions (All services coverage)	16.1 (15.9, 16.3) –0.6 (–0.8, –0.3) –3.5%	95,104 (93,919, 96,288) –3254 (–4837, –1671) –3.3%
	Increased coverage and quality of antenatal, postnatal services, and improved availability of CEmONC interventions (All services coverage and qual.)	14.9 (14.8, 15.1) –1.69 (–1.94, –1.44) –10.2%	88,599 (87,559, 89,638) –9759 (–11,242, –8276) –9.9%
	Maximum availability of antenatal, intrapartum, and postnatal services (All services max.)	14.4 (14.2, 14.6) –2.3 (–2.6, –2.0) –13.6%	85,281 (84,157, 86,405) –13,077 (–14,802, –11,351) –13.3%
	No availability of antenatal, intrapartum, and postnatal services (All services min.)	18.2 (18.0, 18.4) 1.9 (1.3, 1.9) 9.6%	107,654 (106,499, 108,809) 9297 (7777, 10816) 9.5%

Scenarios relating to postnatal service delivery are not included here.

^a^Percentage difference from the SQ is only presented for those scenarios in which the 95% confidence interval does not contain 0.

**Table 4:** Average NMR, total neonatal deaths and total DALYs due to ‘Neonatal Disorders’ during the intervention period (2023–2030) by scenario

	Scenario Full name (Short name)	Average NMR (95% CI) Mean difference (95% CI) Percentage difference from SQ^a^	Total Neonatal Deaths (95% CI) Mean difference (95% CI) Percentage difference from SQ^a^	Total Neonatal DALYs (95% CI) Mean difference (95% CI) Percentage difference from SQ^a^
Comparator scenario:	Status Quo (SQ)	16.9 (16.7, 17.1) Ref.	99,902 (98,661, 101,142) Ref.	6,981,837 (6,901,833,7,061,841) Ref.
Scenarios relating to antenatal services:	Increased routine ANC coverage (AN coverage)	15.9 (15.8, 16.1) –1.0 (–1.3, –0.7) –5.8%	94,135 (93,136, 95,134) –5766 (–7548, -3985) –5.8%	6,556,867 (6,485,449, 6,628,285) –424,940 (–548,281, –301,695) –6.1%
	Increased coverage and quality of antenatal services (AN coverage and qual.)	15.0 (14.8, 15.2) –1.9 (–2.2, –1.6) –11.2%	88,700 (87,273, 90,128) –11,201 (–12,903, –9500) –11.2%	6,181,879 (6,090,215, 6,273,543) –799,958 (–908,339, –691,578) –11.5%
	Maximum availability of antenatal services (AN max.)	15.0 (14.8, 15.2) –1.9 (–2.2, –1.6) –11.3%	88,663 (87,564, 89,761) –11,239 (–13,096, –9382) –11.3%	6,159,507 (6,084,861, 6,234,153) –822,330 (–946,250, –698,409) –11.8%
	No antenatal services (AN min.)	19.4 (19.2, 19.6) 2.5 (2.3, 2.8) 14.9%	114,697 (113,647, 115,748) 14,796 (13,348, 16,243) 14.8%	8,008,887 (7,940,545, 8,077,227) 1,027,050 (933,805, 1,120,295) 14.7%
Scenarios relating to intrapartum services:	Increased availability of intrapartum BEmONC interventions (IP BEmONC)	13.1 (13.0, 13.2) –3.8 (–4.0, –3.6) –22.4%	77,580 (76,280426, 78,357) –22,321(–23,476, –20,187) –22.3%	5,400,914 (5,354,350, 5,447,479) –1,580,923 (–1,661,139, –1,500,707) –22.6%
	Increased availability of intrapartum CEmONC interventions (IP CEmONC)	13.1 (13.0, 13.3) –3.8 (–4.0, –3.5) –22.2%	77,976 (76,960, 78,992) –21,926 (–23,664, –20,187) –21.9%	5,452,852 (5,382,481, 5,523,223) –1,528,985 (–1,640,003, –1,417,967) –21.9%
	Maximum availability of intrapartum services (IP max.)	12.0 (11.8, 12.2) –4.9(–5.1, –4.6) –28.8%	71,398 (70,135, 72,661) –28,503 (–29,961, –27,046) –28.5%	4,992,053 (4,907,342, 5,076,764) –1,989,783 (–2,087,019, –1,892,589) –28.5%
	No intrapartum services (IP min.)	22.0 (21.7, 22.3) 5.1 (4.9, 5.4) 30.3%	130,193 (128,578, 131,809) 30,292 (28,832, 31,751) 30.3%	9,096,867 (8,985,226, 9,208,508) 2,115,030 (2,005,525, 2,224,535) 30.1%
Scenarios relating to postnatal services:	Increased PNC coverage (PN coverage)	16.9 (16.7, 17.1) –0.1(–0.2, 0.4) N/A	100,765 (99,521, 102,009) –864 (–719, 2447) N/A	7,026,456 (6,948,270, 7,104,642) 44,619 (–59,374, 148,612) N/A
	Increased coverage and quality of postnatal services (PN coverage and qual.)	15.7 (15.4, 15.9) –1.2 (–1.5, –0.9) –7.1%	92,827 (91,718, 93,395) –7075 (–8983, –5167) –7.1%	6,470,768 (6,387,697, 6,553,840) –511,069 (–645,141, –442,389) –7.3%
	Maximum availability of postnatal services (PN max.)	15.3 (15.1, 15.3) –1.6 (–1.9, –1.4) –9.7%	90,338 (89,491, 91,184) –9564 (–10,919, –8209) –9.6%	6,311,920 (6,254,857, 6,368,984) –669,917 (–760,532, –579,301) –9.6%
	No postnatal services (PN min.)	17.7 (17.5, 18.0) 0.9 (0.6, 1.1) 5.1%	104,903 (103,710, 106,096) 5002 (3365, 6638) 5.0%	7,318,664 (7,240,983, 7,396,345) 336,824 (221,265, 442,389) 4.8%
Scenarios relating to all services:	Increased coverage and antenatal, postnatal services and improved availability of BEmONC interventions (All services coverage)	12.4 (12.2, 12.5) –4.5 (–4.8, –4.3) –26.9%	73,128 (72,237, 74,020) –26,773 (–28,050, –25,496) –26.8%	5,079,849 (5,021,468, 5,138,229) –1,901,989 (–1,990,484, –1,813,493) 27.2%
	Increased coverage and quality of antenatal, postnatal services, and improved availability of CEmONC interventions (All services coverage and qual.)	10.9 (10.8, 11.1) –6.0 (–6.3, –5.7) –35.4%	64,649 (63,773, 65,525) –35,253 (–37,062, –33,444) –35.3%	4,475,043 (4,416,512, 4,533,574) –2,506,794 (–2,625,589, –2,388,000) –35.9%
	Maximum availability of antenatal, intrapartum, and postnatal services (All services max.)	9.4 (9.3, 9.5) –7.5 (–7.6, –7.3) –44.2%	55,908 (55,251, 56,564) –43,994 (–45,132, –42,856) –44.0%	3,882,514 (3,839,590, 3,925,438) –3,099,323 (–3,174,268, –3,024,379) –44.4%
	No availability of antenatal, intrapartum, and postnatal services (All services min.)	26.5 (26.3, 26.7) 9.6 (9.3, 9.9) 57.0%	156,699 (155,346, 158,052) 56,798 (54,956, 58,639) 56.9%	10,964,980 (10,868,610, 11,061,350) 3,983,143 (3,853,275, 4,113,011) 57.1%

^a^Percentage difference from the SQ is only presented for those scenarios in which the 95% confidence interval does not contain 0.

As evident from Table 2, no scenarios led to an average MMR during the intervention period below the SDG 3.1 target of 70 maternal deaths per 100,000 live births^34^ and only the scenario *All services max*. led to an average MMR below the EPMM supplementary national target of 140 maternal deaths per 100,000 live births^22^. In this scenario the average MMR was 64% lower than the SQ scenario at 120 (95% CI [114.8, 125.2]) maternal deaths per 100,000 live births. Notably, this observed reduction is lower than the sum of the observed average reductions in MMR from the scenarios *AN max., IP max*., and *PN max*. This effect was however not observed for stillbirths or neonatal deaths.

Among scenarios simulating isolated changes to specific services, the greatest change in MMR from the SQ was observed in the scenario PN max., in which the utilisation and quality of services offered in the postnatal period were maximised so that all women and neonates received postnatal care (PNC) within forty-eight hours of birth, seek additional care following complication onset and all required interventions are delivered. In this scenario the average MMR within the intervention period was reduced by nearly half (42%), a difference of 138.9 (95% CI [127.8, 149.8]) maternal deaths per 100,000 live births- equating to 11,359 (95% CI [10,952, 11,766]) fewer maternal deaths during that time and over 350,000 fewer maternal DALYs (Table 2). A similarly large reduction was observed in the scenario *PN coverage and qual*., where the population level coverage of early PNC was increased to 90% alongside maximum quality of postnatal services delivered, as shown in Fig. 2C and Table 2. As evident from Fig. 3, reduction in the total MMR is largely due to a reduced number of maternal deaths associated with post-partum haemorrhage and sepsis. Notably, in the scenarios where coverage of routine antenatal or postnatal services was increased without an associated increase in quality (AN Coverage. and PN Coverage.), there was no reduction in the rate of maternal deaths.

By modelling scenarios in which no antenatal, intrapartum, or postnatal maternity services were delivered during the intervention period (AN Min., IP Min., PN Min and All services min.) we aimed to estimate the effect of current service delivery on outcomes through comparison with the SQ. Current delivery of all included services within the SQ scenario are estimated to reduce the average MMR by 171.3 (95%) CI [161.2, 181.4] deaths per 100,000 live births (a 52% difference from the scenario All services min.) between 2023 and 2030. Within scenarios simulating isolated changes to specific services delivery, the largest difference was observed for AN min. suggesting delivery of antenatal services (including routine and emergency antenatal interventions) within the SQ scenario is estimated to reduce the average MMR by 68.9 (95% CI [55.9, 82.2]) maternal deaths per 100,000 (a 21% difference).

Considering stillbirth (Table 3), the greatest reductions in the SBR and total stillbirths was observed in the scenarios where the coverage and quality of all services were increase concurrently (All services coverage and qual., All services max.). A 14% reduction in the average SBR during the intervention period was observed in the scenario All services max. The scenario IP max., in which all women gave birth in a healthcare facility and all women who require any interventions during labour (e.g. assisted vaginal delivery) led to the largest reduction in the average SBR of the remaining scenarios. In this scenario the SBR was reduced by 10%, equating to 9189 (95% CI [7753, 10,625]) fewer stillbirths between 2023 and 2030. Similar gains were made in scenario IP CEmONC. where facility delivery coverage remained unchanged from SQ (91%) however coverage of prophylactic and all EmONC interventions was 90% (Table 1, Fig. 2F). Improved coverage and quality of antenatal services (AN coverage and qual.) led to a smaller reduction in the total SBR and was predicted to avert 3% of the average SBR, an average reduction of 0.5 (95% CI [0.2, 0.7]) stillbirths per 1000 births (Fig. 2E). However, current antenatal services delivery was estimated to avert more stillbirths than intrapartum services when comparing scenarios AN Min. and IP Min. with the SQ. There was a 7% difference in the number of stillbirths with 6639 (95% CI [4614, 8663]) more stillbirths occurring in the scenario AN Min.

For the rate of neonatal deaths, only three scenarios led to an average NMR at least as low as the SDG 3.2 target of an NMR of 12 neonatal deaths per 1000 live births^34^, including All services max., All services coverage and qual. and IP max (Table 4). Maximum availability of maternity services, as defined in Table 1 as All services max., was predicted to reduce NMR by nearly half (44%) potentially averting over 3 million DALYs during the intervention period. Notably, through changes in service delivery modelled in the scenario IP max., in which coverage of facility delivery and availability of all intrapartum prophylactic and EmONC interventions was set at 100%, an additional 28,503 (95% CI [27,046, 29,961]) deaths and 1,989,783 (95% CI [1,892,589, 2,087,019]) neonatal DALYs were averted between 2023 and 2030, a 29% reduction from the SQ. Similar results (22% reduction) were observed by comparing the SQ scenario with the scenario IP CEmONC. (Table 3). Figure 4 shows that reduction in NMR in scenarios related to intrapartum service delivery is mostly attributable to reduction in deaths caused by neonatal encephalopathy and preterm respiratory distress syndrome due to greater availability of newborn resuscitation and antenatal corticosteroid administration. Results also suggest that current intrapartum service delivery defined within the SQ scenario is predicted to prevent 30,292 (95% CI [28,832, 31,751]) neonatal deaths and 2,115,030 (95% CI [2,005,525, 2,224,535]) neonatal DALYs between 2023 and 2030 when comparing the SQ scenario with the scenario IP Min. This substantial impact is however less than half of the predicted impact of all services (All services min.) on neonatal mortality during the intervention period which are predicted to avert nearly four million DALYs (3,983,143 (95%) CI [3,853,275, 4,113,011]) and nearly 57,000 (56,798 (95% CI [54,956, 58,639])) neonatal deaths.

Additionally, scenarios where changes to antenatal services were modelled also demonstrated important reductions in the NMR which was reduced by 11% in both the scenario AN coverage and qual., and AN Max. This can be attributed to a reduction in deaths caused by preterm respiratory distress syndrome and other complications of prematurity (Fig. 4) linked to reduction in the rate of preterm births discussed below. Interestingly, there was no reduction in neonatal deaths or DALYs observed when coverage of routine PNC was increased without increased quality (*PN coverage)* (Table 3).

### Impact of maternity service delivery on morbidity

The effect of service coverage on morbidity was explored by comparing the incidence/prevalence of modelled maternal and neonatal conditions within the SQ and the other scenarios during the intervention period. Figures S81–S83 show the average rate or prevalence of the conditions discussed, organised by scenario. A list of all conditions included in the model is available in Table S1 whilst definitions for each modelled complication are available in the supporting information (S1 File, §3).

Reduction in the rates of maternal morbidity were most often observed within scenarios related to antenatal services delivery. In all scenarios where antenatal service coverage was increased (AN coverage, AN coverage and qual., AN max., All services coverage., All services coverage and qual., All services max.) a reduction in the rates of maternal anaemia, malaria during pregnancy, mild pre-eclampsia, severe pre-eclampsia, eclampsia, gestational hypertension, severe gestational hypertension, preterm birth, low birth weight and preterm respiratory distress syndrome in newborns were observed as shown in Figs. S81–S82. As evident from the figure, excluding the scenarios in which availability of all services were increased, the largest reduction from the SQ scenario was noted in the scenarios AN coverage and qual., and AN max., where coverage and quality of ANC were both increased, however important reductions in morbidity were seen in AN coverage. In this scenario 90% of mothers received four or more antenatal care (ANC) visits however quality remained unchanged from assumptions in the SQ. For example, in this scenario, the prevalence of maternal anaemia at birth was 5.7% (95% CI [5.6, 5.8]) lower than the status quo (a 13.8% reduction) and the rate of preterm birth was reduced by 13% equating to 1.7 (95% CI [1.6, 1.8]) fewer preterm births per 100 births.

Additionally, results suggest that current antenatal service delivery in Malawi, as defined in the SQ, is predicted to prevent a substantial amount of maternal and perinatal morbidity between 2023 and 2030 for all conditions listed above. Notably, there was a 95%, 48% and 30% difference in the rates or prevalence of eclampsia, maternal anaemia at birth and pre-term labour respectively within the SQ scenario and the scenario AN Min.

In scenarios relating to intrapartum service delivery, the improved availability of intrapartum interventions led to substantial reduction in the rate of intrapartum sepsis, postpartum haemorrhage (PPH), eclampsia, neonatal sepsis, neonatal encephalopathy, and preterm respiratory distress syndrome (Fig. S83-S84). Notably, as evident from the figure, the reduction in these rates was similar in both scenarios IP BEmONC and IP CEmONC suggesting that improvements in coverage of prophylactic and BEmONC interventions (listed in Table 1) without improvements in CEmONC intervention availability could still lead to important reductions in morbidity. Additionally, as seen in Fig. S83D, scenarios in which the coverage of CEmONC interventions delivered in labour were increased (IP CEmONC, IP max.) led to an increased rate of maternal postnatal sepsis due to the modelled relationship between caesarean section and this condition.

Results indicate that changes to coverage of postnatal services was not predicted to impact the incidence of as many conditions but there was a notable reduction in the rates of severe pre-eclampsia and anaemia at the end of the postnatal period (Fig. S85).

### Health service outcomes

For each of the scenarios considered, we analysed the expected change in the number of routine healthcare appointments delivered. For scenarios relating to antenatal service coverage we extracted the number of ANC contacts required to deliver the modelled levels of coverage. At current service coverage within the SQ scenario an average of 16,380,000 (95% CI [16,330,000, 16,430,000]) ANC contacts would be delivered between 2023 and 2030, which is 8,110,000 (95% CI [8,060,000, 8,152,000]) fewer than the number of contacts delivered in the scenario AN coverage and qual. (24,490,000) (95% CI [24,440,000, 24,540,000]). This is a 49% increase in the total number of visits. Within the model each ANC contact is assumed to take an average of 12 min of nursing/midwifery time, sourced from Berman et al.^35^; the additional number of contacts would require an average of 1,622,000 h of healthcare worker (HCW) time over the intervention period, 202,750 hours per year, to be delivered: this is 2% of the total time available of all nurses/midwives in the health system.

For the scenarios relating to postnatal services we extracted the number of PNC contacts required to deliver the modelled levels of coverage. At current service coverage within the SQ scenario an average of 3,280,000 (95% CI [3,273,000, 3,290,000]) maternal PNC contacts and 3,881,000 (95% CI [3,874,000, 3,889,000]) neonatal PNC contacts would be delivered between 2023 and 2030. To achieve 90% coverage of one or more PNC visit an additional 1,834,000 (95% CI [1,826,000, 1,842,000]) maternal contacts and 1,658,000 (95% CI [1,650,000, 1,666,000]) neonatal visits were estimated in the scenario PN coverage and qual. Given that each PNC contact is assumed to take an average of 15 min of nursing/midwifery time, the additional number of contacts would require an average of 458,500 h of HCW time over the intervention period to deliver maternal PNC and 414,500 to deliver newborn PNC. This equates to a total of 109,125 hours per year. Combined this accounts for 1% of the total time available of all nurses/midwives in the health system.

### Maximum ability to pay for modelled changes in service delivery

Finally, for each of the scenarios in which both an increase in service coverage and/or quality was modelled and the scenario was found to reduce maternal and/or neonatal DALYs (e.g. the 95% confidence interval for the estimate of difference between the intervention scenario and SQ does not contain 0) we calculated the maximum ability to pay by the Malawian government to achieve modelled changes that would be consistent with being cost effective over the intervention period.

Health economic evaluation assumes an intervention should be delivered within a health system if the intervention generates more health than could be produced otherwise within the system with the same resources (i.e. the intervention’s benefits exceed its opportunity costs). We assume that for every DALY averted due to an intervention, the system should not pay more than the cost at which it is already able to avert a DALY from existing interventions, at the margin^36^. Therefore the maximum ability to pay for each scenario was calculated as the product of the incremental health impact of a scenario compared to the SQ (e.g. DALYs averted) and the cost-effectiveness threshold (CET), representing the marginal productivity of Malawi’s health system^36^. We compared these values to the projected total national health spending in Malawi during 2023 to 2030. This was estimated as $10.1 billion using the projected annualised growth rate in health spending per capita between 2015-2030 estimated by Dieleman et al.^37^. We used a CET value of $62.3 (2016 value adjusted for inflation) for Malawi as estimated by Ochalek et al.^38^ and applied by the MoH and partners during the development of the HSSP II and III^28,38–40^.

For the scenario All services max. in which the greatest number DALYs were averted (maternal and neonatal combined), the maximum ability to pay is $226.4 million between 2023 and 2030 (2.3% of projected total health spending during 2023–2030). Considering scenarios which focused on changes to the coverage and/or quality of single services the scenario *IP. Max*. led to the greatest number of DALYs averted presenting a maximum ability to pay of $135.9 million (1.4% of total health spending during 2023–2030). This contrasts with scenarios in which the incremental impact on health was the lowest, such as AN coverage, in which the maximum ability to pay was $26.5 million during this period (0.26% of total health spending during 2023–2030). The maximum ability to pay for each scenario and the percentage of total national health spending is presented in Table S62.

## Discussion

We present here an individual-based simulation model of maternal and newborn health and healthcare utilisation in Malawi which we have applied to estimate the impact of both current and improved antenatal, intrapartum, and postnatal service delivery on a breadth of health outcomes between 2023 and 2030. Firstly, our results indicate that, at their current assumed levels of coverage and quality in Malawi, these services are predicted to remain instrumental in improving population health through the prevention of maternal and perinatal morbidity, mortality and DALYs. When delivered in tandem, these services are predicted to prevent nearly 437,000 and 4 million maternal and neonatal DALYs respectively during this time, partially driven by 10,000 and 57,000 fewer maternal and neonatal deaths respectively. Considering services in isolation, intrapartum services, as they are currently delivered in Malawi, are predicted to prevent the greatest number of neonatal deaths/DALYs (2 million neonatal DALYs and 30,000 neonatal deaths) whilst antenatal service are predicted to prevent the greatest number of maternal deaths/DALYS (180,000 maternal DALYs and over 4000 maternal deaths).

Secondly, our results highlight both the potential gains and missed opportunities associated with expanding the coverage only, or both coverage *and* quality of these services beyond their current levels in Malawi. Generally, we found that increased coverage of routine preventative services, such as ANC and PNC, without any change in quality did not lead to meaningful reductions in stillbirth or maternal mortality compared to the Status Quo, despite important reductions in morbidity. This can be attributed to several factors such as: the limited availability of key consumables (e.g. medicines) and low quality of clinical care, meaning that increased coverage does not equate to sufficiently increased delivery of interventions to prevent deaths, and the potential effect of increased demand for services on health service ‘squeeze’ leads to reduced treatment effectiveness and negates the potential positive effects of increasing coverage.

Broadly, these findings suggest that current service quality in Malawi is a significant barrier to achieving improvements in maternal and perinatal health in the coming years and that quality improvement must remain a priority area for policy makers. This is supported by additional findings that stillbirths, maternal and neonatal mortality and DALYs were most substantially reduced when service-quality was increased in tandem with coverage compared to scenarios where only coverage was increased. As expected, scenarios where coverage and quality of all services were maximised in tandem led to the greatest percentage reduction in the average MMR (64%), NMR (44%) and SBR (14%). Considering services in isolation, a very large reduction in the average MMR between 2023 and 2030 of over 40% was also observed when postnatal service coverage and quality were maximised, whilst the SBR and NMR were reduced by 10% and 29% respectively where intrapartum services coverage and quality were maximised.

Fortunately, as highlighted earlier, targeted improvement in quality of maternal and newborn health services remains central to the policy agenda of the Malawian government and the ministry of health. This has been clearly signified by the establishment of the Quality Management Directorate in 2016, which provides strategic oversight for quality management and improvement across the health system^41^, and through membership of ‘The Network to Improve Quality of Care for Maternal, Newborn and Child health’ (QCN) between 2017 and 2022^42^. The National Quality Management Policy (2017-2030)^41^ already identifies system-wide bottlenecks that must be addressed to further progress towards improved coverage and quality of health services in Malawi. Addressing these bottlenecks is at the heart of the recent Health Sector Strategic Plan (HSSP III 2023–2030)^28^, which prioritises system level reforms across the seven of the nine HSSP III pillars. In the ‘service delivery’ pillar for instance, a reform on transitioning from vertical programming to integrated and optimised platforms of care aim to delivery high quality services under severe resource constraints^28^. Similarly, reforms on provider autonomy, harmonisation of in-service training, provider payment reforms, direct facility financing and strengthening of community governance of facility Public Financial Management Systems are already underway and aim to identify efficiency savings that can then be funnelled back into the system to expand access to and quality of care. Our results offer policy makers much needed insight into the areas where efficiency savings from such reform initiatives into the system could be redirected to generate increased coverage and quality of care and thus contribute significantly to improvements in population health.

Thirdly, we found that none of the sixteen modelled scenarios was sufficient to reduce maternal mortality by a sufficient degree to meet SDG target 3.1 by 2030 of no country having an MMR of greater than 70 per 100,000 live births^34^. Similar findings were reported by Ward et al.^43^, who applied the Global Maternal Health model, an individual-based microsimulation of the reproductive histories of women in 200 countries, to evaluate the impact of several interventions and strategies on maternal mortality across modelled countries between 2022 and 2030. Ward et al. reported minimal effect of ‘single interventions’, such as increased coverage of antenatal care, in isolation but reported the largest impacts on mortality where quality was improved in tandem with coverage, in addition to modelled increases in referral and linkages between facilities^43^. An integrated comprehensive package of services, in which family planning, community-based, facility-based and health system-relevant aspects were improved, was predicted to decrease the MMR in Malawi by 40% (to 187 (95% UI 79-284) per 100,000) by 2030^43^ which is insufficient to achieve SDG goal 3.1. Alternatively, three scenarios, in which the coverage and quality of all services was increased or maximised or only intrapartum services were maximised, reduced the average NMR to equal or below 12 deaths per 1000 livebirths in line with SDG target 3.2 largely due to deaths associated with the intrapartum period. Similarly large reductions in NMR associated with alternative intrapartum care delivery were predicted by Shrime et al., who used an agent-based simulation model to estimate the effect of regionalised delivery care, where all births occur in facilities capable of caesarean section, in Malawi^44^. They reported a reduction in the NMR of 11.4 (95% posterior credible interval 9.8–13.1) deaths per 1000 live births from the status quo^44^.

Finally, we further contextualised results which detail the impact of modelled scenarios on health by calculating the maximum ability of the Malawian health system to pay to achieve modelled changes to service delivery that would be consistent with being cost effective over the intervention period. Considering the high number of maternal and neonatal DALYs averted due to modelled service changes across several scenarios (i.e. > 2 million DALYs averted during the intervention period) we found that the maximum ability of the health system to pay for any scenario did not exceed 2.5% of the projected total health spending in Malawi between 2023 and 2030^37^. However, total health spending projections from Dieleman et al. are inclusive of governmental, pre-paid private, out-of-pocket and development assistance for health spending meaning that maximum ability to pay values would constitute a much greater percentage of government spending (versus total) given that for 2030 the percentage of total health spending by the government is estimated as 17.6% (95% UI 10.4, 24.3) of total spending^37^. Additionally, we note that there are inherent challenges with applying a fixed cost-effectiveness threshold for large expenditures in the future as likely non-marginal effects and the expected growth of any marginal costs per unit of health produced by the health system are not accounted for^45,46^; accounting for these challenges is outside the scope of this paper.

Whilst other individual-based models have explored similar research questions in Malawi, ours is the first model of maternal and perinatal health in Malawi, or SSA more widely, to sit within a comprehensive ‘whole health system, all disease’ modelling framework^47^—the Thanzi La Onse Model – allowing for advanced epidemiological analyses of health policy implementation at the population level. Such analyses can account for the relationships *between* modelled health conditions, including direct and indirect causes of maternal ill-health, which are well documented in the literature^48–50^. Additionally, the model can explore the potential emergent systems-level behaviours and their relationship with population health. This is pivotal given health systems have long been regarded as complex adaptive systems that can exhibit non-linear behaviour^51–53^, meaning that introduction of new interventions or changes to coverage of existing interventions may have unforeseen effects on the outcome of interest despite evidence that the intervention is effective^54,55^. Furthermore, the model represents a broad range of relevant outcomes beyond mortality, including both direct and indirect causes of maternal and perinatal morbidity and DALYs allowing for a more complete evaluation of the predicted impact of changes in service delivery on population health which have not been evaluated in other similar modelling studies^43,44,47^. Given these factors, our approach differs from other commonly applied models such as, the Lives Saved Tool (LiST), a self-described linear and deterministic model^56^, used extensively to evaluate and guide strategic policy in LMICs^57^, which is not individual based, is primarily focused on mortality outcomes, and does not represent national health systems. As such, we believe results provided here may be a more accurate approximation of the potential effect of improved maternity service delivery in Malawi.

We note that our approach does however have several limitations given that, by necessity, our model is a simplification of reality in Malawi and therefore it would not be possible to incorporate all possible relevant structural factors or parameters. Firstly, we have not captured all maternal and perinatal health conditions driving morbidity and mortality in Malawi (e.g. maternal infections not resulting in sepsis, haemolytic disease, and other neonatal jaundice) meaning a small proportion of the disease burden is not represented and cannot be affected by interventions. This is because we opted to pragmatically include conditions contributing the greatest burden in the country due to high case fatality or high incidence. Additionally, even for conditions included in the model the availability of data is often limited and where data is available from Malawi it is often not nationality representative and is instead derived from one (or a few) districts meaning that we need to assume this data represents the population. Secondly, whilst we have captured all interventions defined in Malawi’s Essential Health Package (EHP)^28,39^ evidence suggests that additional interventions are offered within the health system that are not defined within the EHP. This includes high-dependency (HDU) and intensive care (ICU) for both mothers and neonates. Whilst some critical care services are available at several district and central hospitals there is limited readiness to provide effective care due to resource and infrastructure constraints^58,59^. It is difficult to determine the potential impact of including HDU or ICU interventions within the model on the results here, given limited national data on the availability of care. In addition to the factors listed above we also note that, at present, the model does not capture the potential referrals between facilities for patients who cannot receive treatment due to issues of poor quality (e.g. unavailable resources), care delivered within the private sector, the effect of delays in referral associated with transport availability or counselling interventions.

Finally, regarding our analysis, we have not estimated the associated costs of changes to the availability and use of services. Whilst we have endeavoured to offer insight into the predicted increased requirements of appointments without cost, it is difficult to fully contextualise cost-effectiveness of changes to service delivery. Future iterations of the TLO model will include a costing module which is currently in development.

The model described here offers a data-driven and comprehensive representation of maternal and perinatal health and healthcare utilisation within Malawi. Results from application of the model suggest that whilst the current delivery of maternity services is predicted to continue to avert morbidity and mortality at the population level, there is substantial scope to improve health through high coverage of high-quality services. Importantly, policies which seek to improve service delivery across the pregnancy continuum are most likely needed to ensure Malawi can achieve SDG targets 3.1 and 3.2 by 2030.

## Methods

This research complies with all relevant ethical regulations. The Thanzi La Onse project received ethical approval from the Malawi College of Medicine research ethics committee in November 2019 (P.10/19/2820).

**Fig. 5:**
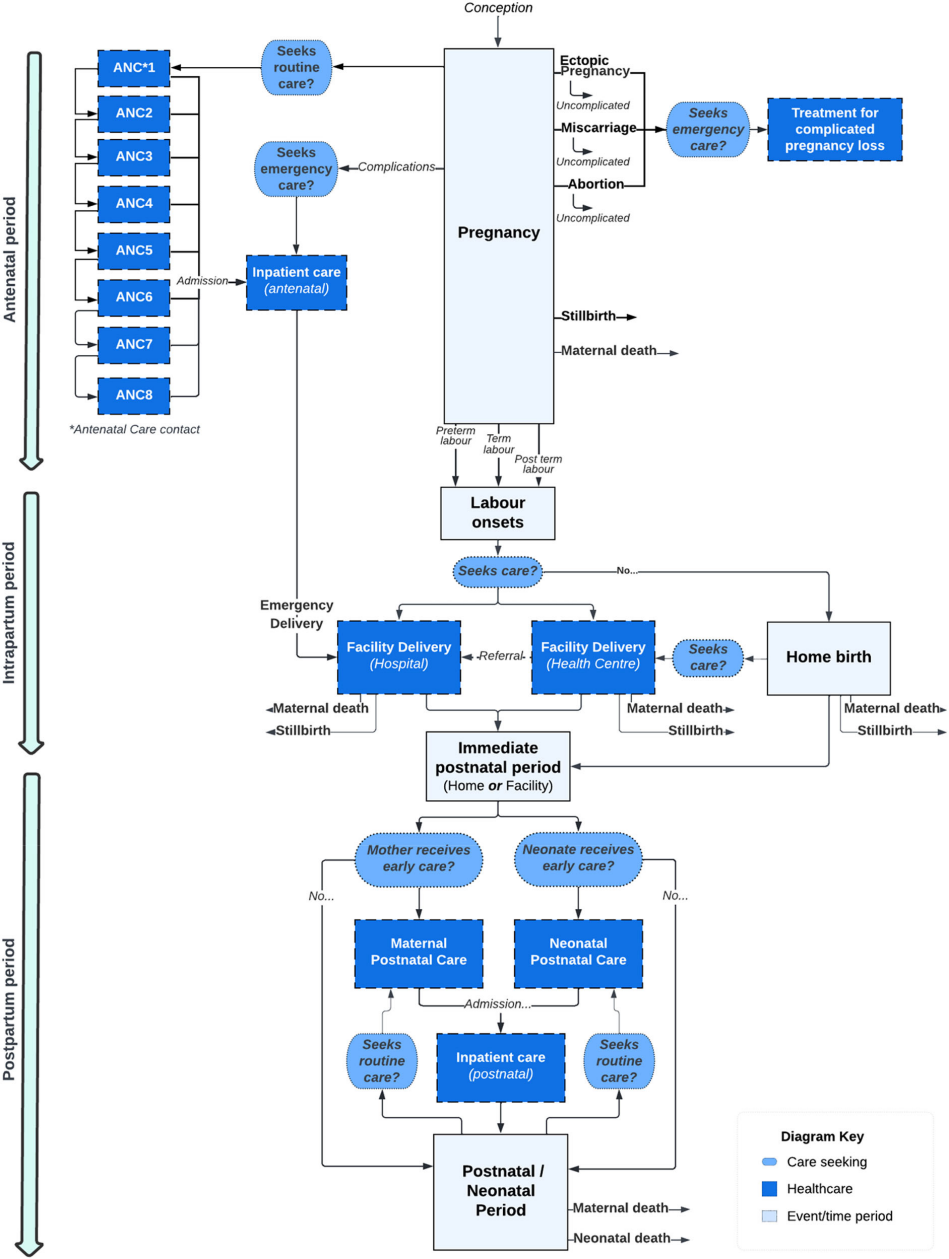
Maternal and perinatal health model schematic. The model explicitly represents the antenatal, intrapartum, and postpartum periods of an individual’s pregnancy as it progresses. Care seeking is driven either by the progression of a pregnancy, the transition between one pregnancy period and another (e.g. seeking healthcare once labour onsets) or following the onset of a complication. Complication-specific risk of maternal or perinatal death is applied following onset, allowing first for the delivery of treatment for those who seek care.

Figure 5 is a diagrammatic representation of the individual-based simulation model described here. The model was developed in the Python programming language (v3.8.18)^60^ using the Pandas data analysis library (v2.0.3)^61^. The source code for the version of the model used to perform the analyses presented here is publicly available^62^. A detailed overview of the model, including structure, variables, parameters, data sources, software verification and model validation procedures (e.g. validation of model structure with clinicians and calibration to data) is provided within the supporting information (S1 File). Additionally, technical documentation relating to other key aspects of the TLO model is available online^63^.

Broadly, the maternal and perinatal health model aims to mechanistically replicate the epidemiology of common maternal and perinatal health conditions within the population of Malawi at the individual-level, alongside the demand for and delivery of maternity services to mothers and newborns. The structure and functionality of the model is mainly informed by data sources from Malawi and similar countries where Malawian data was unavailable, including the Demographic and Health Surveys (DHS) (2010 and 2015/16)^19,64^, the Malawi Emergency Obstetric and Newborn Care (EmONC) needs assessments (2010 and 2014)^24,65^, Malawian Obstetrics and Gynaecology Protocols and Guidelines^66^, the Malawi Standard Treatment Guidelines 5th Edition^67^ and the Malawi Harmonized Health Facilities Assessment 2018/19^21^.

Individuals within the TLO model are characterised by a set of ‘properties’, which are initially defined to generate a population representative of Malawi in terms of demographic and lifestyle variables. Demographic variables include whether an individual is alive, date of birth, age (in days and years), sex, district and region of residence, and cause and date of death. Scaled outputs of the population size and number of births over time, by district, are calibrated to the Malawi 2018 census^68^ and the Global Burden of Disease study^10^ due to the lack of reliable mortality registers in Malawi. Lifestyle variables include wealth level (defined across five wealth quintiles), education status, marital status, and body mass index (BMI) and factors such as alcohol use and sugar intake. Data from the Malawi DHS^19,64^ largely informs the distribution of these variables within the population. Further detail on the methods and data used to initialise the modelled population is available elsewhere^63^.

Models within the TLO framework define, populate, and update additional properties relevant to modelled health processes and conditions (e.g. pregnancy status, gestational age). Feedback between an individual’s properties and scheduled events as the simulation moves forward in time, such as initiating health care, generate the model’s behaviour.

### Maternal and perinatal health

#### Overview

Women can become pregnant with a probability informed by their contraceptive use and fecundity via the TLO contraception model as described by Colbourn et al.^69^. Briefly, this model calculates and applies probability of contraception initiation, switching, discontinuation, and failure calculated from the DHS contraception calendar data within women of reproductive age as the simulation moves forward in time^69^. Parameters are applied on a monthly timestep including the probability of pregnancy in women who are not on contraception or experience contraception failure. These probabilities have been derived through calibration to the World Population Prospects medium variant population projection for Malawi^70^.

All pregnant women are initially exposed to a monthly time-since-conception specific risk of pregnancy-related health conditions, such as gestational diabetes or preeclampsia, as their pregnancy progresses. There are also risks of termination of pregnancy via ectopic pregnancy, abortion, or stillbirth. Women who remain pregnant will go into labour, either at preterm, term or post term gestation, and are then exposed to a risk of intrapartum complications, such as obstructed labour, followed by immediate then weekly risk of complications in the postnatal period. Similarly, neonates are exposed to risk of complications immediately following birth, such as preterm respiratory distress syndrome, followed by a weekly risk of complications in the remainder of the neonatal period.

#### Maternal and perinatal health conditions

A list of relevant conditions specific to pregnancy or the neonatal period contributing the greatest morbidity and mortality in Malawi for inclusion in the model was initially developed through our clinical experience, evaluation of local data sources^24^ and the Global Burden of Disease (GBD) study^10^. To identify relevant relationships between these conditions, or other diseases in the TLO framework, (e.g. the relationship between maternal malaria infection and risk of preterm labour^71^), several targeted literature searches were conducted. A compiled list of relationships was then reviewed utilising our clinical experience to determine the suitability of inclusion in the model. Relationships were represented in the model based on significant evidence of effect and reasonable biological plausibility suggestive of a causal effect. These relationships, and any relevant assumptions, are described in detail for each modelled condition in the supporting information (S1 file, §3).

Table S1 (S1 file) details all the conditions which are modelled. For each condition a natural history model was first developed which mechanistically represents the risk of onset and progression (if relevant) at the appropriate point of pregnancy/postnatal period or the neonate’s life in the absence of treatment. The primary aim of this process was to adopt the simplest structure consistent with the conditions and interactions that would be represented. Figures S7-S32 are the models for the health conditions demonstrating the individual level variables and parameters representing rate of onset and rate of progression along with the parameters representing the effect of other conditions on risk of disease onset.

Both morbidity and mortality outcomes associated with modelled conditions are represented. Disability weights, sourced from Salomon et al.^72^, are attached to each of the conditions in Table S1 following onset which are captured monthly at the population level and combined additively and capped at 1.0 in the case of multimorbidity. Tables S12 and S13 list the disability weights for modelled conditions (S1 File). The probability of cause-specific maternal or neonatal death is applied to any individual experiencing a potentially fatal condition after it has been determined if they will receive any healthcare. If treatment is received, this probability of death is reduced by the relevant treatment effect. Deaths attributed to each cause are then recorded as the simulation progresses. This allows for the estimation of morbidity (e.g. incidence and prevalence of conditions), mortality rates (such as the maternal mortality ratio (MMR)) and Disability Adjusted Life Years (DALYs) attributable to maternal and neonatal conditions.

### Maternity health service unitisation and delivery

#### Healthcare seeking behaviour

The probability a woman will seek routine maternity care is calculated at a relevant time during the pregnancy including initiation of pregnancy, the onset of labour, and the beginning of the postnatal period. For example the probability that a pregnant woman will initiate routine ANC is calculated at pregnancy onset. Individual probability of care seeking for ANC, facility delivery and PNC is calculated from multivariable regression models accounting for the effect of sociodemographic and pregnancy variables on care seeking modelled with data from the Malawian DHS^19,64^. At present, quality of care delivered within an individual’s previous interactions with the health system does not impact probability of future care seeking. Where relevant, we model timing and frequency of care seeking. Again, considering ANC as an example, we replicate the average number of ANC visits per pregnancy and the timing of the initiation of first ANC visit at the population level in keeping with estimates from the DHS^19,64^.

It is assumed that women who experience a condition during or after pregnancy, for which emergency care is likely required to preserve life and/or pregnancy, will choose to seek care with probability of care seeking. This probability was sourced from Chinkhumba et al.^73^ based on self-reported care seeking following obstetric emergencies in a sample of pregnant women in Malawi.

#### Maternity services

We have aimed to replicate maternity service provision as it currently stands within Malawi and therefore have modelled services which are defined within Malawi’s EHP^28,39^. These services can be broadly categorised as Post Abortion Care and Ectopic Pregnancy Case Management, Routine and Emergency Antenatal Care, Intrapartum Care and Postnatal Care. Components of distinct interventions within each maternal and perinatal health ‘service’, such as indication for treatment and required consumables for intervention delivery (i.e. medication), were derived from national clinical treatment guidelines^66,67^ and reviewed using our clinical experience. A full list of the interventions included in the model is shown in Table 5, which are classified as screening (leading to initial treatment after diagnosis), preventative (reducing an individual’s risk of a given health outcome), or curative (reducing risk of morbidity and death associated with a health condition).

**Table 5:** Interventions included in the model

Health system interaction^c^	Modelled interventions
Antenatal care (Contacts 1–8)	Screening interventions: • Blood pressure measurement • Urine dipstick • Point-of-care haemoglobin testing • Syphilis testing (rapid plasma reagin) • HIV testing^a^ • Gestational diabetes testing (blood glucose) • Screening for depression^a^ • Screening for tuberculosis^a^ Preventative interventions: • Daily iron and folic acid supplementation • Calcium supplementation^b^ • Balanced energy and protein supplementation • Antibiotic treatment for syphilis • Albendazole (deworming) • Intermittent preventive treatment of malaria during pregnancy (IPTp) • Insecticide treated bed nets^a^ • Tetanus toxoid immunisation^a^
Inpatient antenatal services	Preventative interventions: • Antibiotics prophylaxis following PROM • Referral for emergency delivery Curative interventions: • Gestational diabetes case management (diet/exercise, oral diabetic medications, insulin) • Blood transfusion • Antihypertensive treatment (oral and intravenous) • Magnesium sulphate
Facility delivery (BEmONC)	Preventative interventions: • Clean birth practices • Antibiotics prophylaxis following PROM • Antenatal corticosteroids following preterm labour Curative interventions: • Maternal sepsis case management (intravenous antibiotics) • Antihypertensive treatment (intravenous) • Magnesium sulphate • Assisted vaginal delivery • Active management of the third stage of labour • Neonatal stimulation and resuscitation
Facility delivery (CEmONC)	Curative interventions: • Delivery via caesarean section • Additional curative surgery (uterine rupture repair/hysterectomy) • Blood transfusion
Postnatal care (maternal)	Screening interventions: • HIV testing^a^ • Screening for depression^a^ Preventative interventions: • Daily iron and folic acid supplementation Curative interventions: • Postpartum haemorrhage case management (uterotonics, manual removal of retained placenta, referral for surgery) • Maternal sepsis case management (intravenous antibiotics) • Antihypertensive treatment (intravenous) • Magnesium sulphate
Postnatal care (neonatal)	Screening interventions: • HIV testing^a^ Preventative interventions: • Other essential newborn care – vitamin K prophylaxis, eye care, immunisations Curative interventions: • Neonatal sepsis case management – injectable antibiotics • Neonatal sepsis case management – full supportive care • Kangaroo mother care
CEmONC – Postnatal	Curative interventions: • Surgery (management of postpartum haemorrhage) • Blood transfusion

^a^The effect of this intervention on outcomes is determined by the relevant disease model and is therefore not described within the supplementary material of this paper.

^b^Calcium supplementation is only delivered to women deemed to be at risk of developing pre-eclampsia in line with Malawian guidelines.

^c^A health system interaction (HSI) is an event in the simulation in which healthcare is delivered. Interventions are housed within HSIs defined by the level of facility at which they occur and the required healthcare worker time.

Healthcare services within the TLO health system model are delivered via distinct health system interaction (HSI) events. Each of these events are defined in terms of the health facility level at which they are delivered, the required consumables, required health care worker time, by cadre, and required inpatient bed days. Intervention effects are included in the model as relative risks or rates and derived from the literature. Further detail on the health system modelling within TLO is available elsewhere^62,63^.

Quality of care within maternity services is represented by the probability that an intervention, which is indicated and should be provided according to the national clinical guidelines, will or will not be provided. The administration of interventions is therefore contingent on the required consumables being available and the HCW identifying the need for a given intervention (i.e., clinical competence). In addition, for interventions classified as EmONC, it is also contingent on the availability of appropriately trained HCWs^24,65^. Probability of intervention delivery is calculated independently from parameters representing the above factors for each intervention that an individual requires. Further detail on the representation of quality of care can be found in the supporting information alongside relevant parameters and assumptions (S1 File - §2.3.1 and §2.4.3.2).

Finally, we have sought to replicate the effect of Thaddeus and Maine’s ‘three delays’^74^ on outcomes. These refer to delays in initiating care seeking or reaching care when care is required for emergency treatment or facility delivery (delay one and two respectively) and during receipt of treatment (delay three). A fixed probability is used to determine if an individual will experience delay one or two. These are sourced from Tiruneh et al.^75^ who conducted an institutional-based cross sectional study reporting the proportion of postpartum women who were delayed in deciding to seek emergency obstetric care for institutional delivery in a study conducted in Ethiopia. Delay three is assumed to occur if the requested healthcare worker time within a given HSI exceeds the remaining availability of HCW time for a simulated day. Mechanistically this logic utilises a parameter of the TLO health system model called the squeeze factor which is calculated for each HSI event that is executed within a simulation run using the fractional over-demand among the HCW cadres who are required to deliver said event. The squeeze factor is therefore calculated as the required HCW time divided by available time, for a given day in the simulation, minus one. This leads to a squeeze factor of less than 0 when required time is less than available time and a squeeze factor of greater than 0 when required time is greater than available time. To determine the squeeze factor value in the model over which delay three would occur the model was run over one year and the squeeze factor extracted for each of the relevant HSIs. The median value across the squeeze factors was determined in the model’s current state and was approximately 3 across the events. This value was selected for the parameter meaning approximately 50% of women receiving care would experience a delay with evidence from contextually similar settings that delay three is high^26,27,76,77^. Delays are assumed to reduce treatment effectiveness leading to increased risk of maternal or neonatal mortality.

### Model calibration

Model validation was primarily achieved through calibration of the model to data from Malawi. The primary calibration ‘areas of focus’ included maternal and perinatal mortality, morbidity, and maternity health service utilisation and coverage. Empirical estimates from nationally representative data sets were prioritised. Population-level maternal and neonatal mortality rate estimates from Malawi, to which the model was calibrated, were sourced from the DHS^19,64^, whilst the estimated proportion of maternal and neonatal deaths due to leading causes was sourced from the national EmONC needs assessment surveys^24,65^ and prospective observational study data from Malawi^78^. The stillbirth rate was sourced from the UN Inter-agency Group for Child Mortality Estimation (UN IGME) estimates for Malawi^79^. Estimates relating maternity service utilisation and coverage, including population-level coverage of antenatal care (in addition to timing of initiation of ANC, average visits per pregnancy, proportion of women attending four or more visits), facility delivery (coverage and delivery location (e.g. hospital, health centre), and postnatal care were taken from the most recent DHS surveys conducted in Malawi^19,64^). Due to lacking population-level data on the modelled conditions, data for calibration of morbidity was sourced from both Malawi and similar settings.

To calibrate the model we first defined initial values for parameters based on the literature, as described above. Following this the selection of calibration targets from available sources was undertaken to ensure that the model would be able to reliably evaluate the effect of maternity services on a breadth of key outcomes. Due to the number of model parameters which could reasonably drive outcomes for calibration (e.g. the MMR), and the computational cost of running the model multiple times, we determined that goodness-of-fit would be assessed visually by comparison of central model estimate with central data estimates for the above areas between 2011 and 2022. Parameters we believed were underspecified by the available data were identified as those we were willing to update. These included parameters defining monthly/weekly risk of disease acquisition and risk of untreated cause-specific maternal and neonatal mortality. Parameters manipulated during calibration are highlighted within the parameter tables in S1 File. We noted there was initially a poor fit in respect to annual MMR and NMR and therefore parameter values were iteratively updated – this process was stopped when goodness-of-fit was subjectively deemed to not be inconsistent with available data and where further updating did not lead to substantially improved fits.

Calibration of the model to mortality data is presented in the results section. Additional detail relating to calibration methods, calibration data sources and plots demonstrating model fit to remaining calibration data are available within the supporting information (S1 File, §4).

### Analysis plan—estimating the impact of maternity service delivery

An initial population of individuals was modelled with a model-to-real population ratio in 2010 of 1:58, and the model was run from 1/1/2010 until 1/1/2031. The modelled population is demographically and epidemiologically representative of the Malawian population meaning we did not limit the simulation to women of reproductive age. This is decision was made to preserve the functionality of other TLO components which generate disease incidence based on transmission between men and women that also drive maternal outcomes, such as HIV.

Levels of service coverage in the modelled scenarios (Table 1) were adapted from recent global targets relating to maternal and newborn health, namely the EPMM^22^ and ENAP^23^ strategies, and local monitoring and evaluation targets linked to national strategic policy^28,39^. We modelled any changes occurring within a scenario (e.g. increase coverage and quality of a given service) to take effect on 1/1/2023 to avoid assumptions relating to the rate of implementation of service delivery changes over time during the intervention period. Furthermore, we make no attempt to predict changes in population health driven by other factors not explicitly captured in the model as these are unknown and any such improvements would be assumed to occur equally across each modelled scenario and would not impact our conclusions. Each scenario was run 20 times with the same initial parameters set and a different fixed run seed used to initialise random number generators. Model results were rescaled to correspond to the real size of the population of Malawi.

The outcomes of interest are maternal and perinatal morbidity, mortality (total deaths, mortality rates and cause-specific mortality), DALYs and relevant health system outputs such as additional number of appointments required to deliver increased service coverage. We calculated the mean value of a given outcome for each year across the 20 runs of the simulation for each scenario alongside the 95% confidence interval (CI). We present here the mean rate and 95% CI of these outcomes during the intervention period of 2023 and 2030 by taking the mean outcome during these years for each run of each scenario and then calculating the mean across runs of the 2023–2030 means. As we have opted to model changes in the intervention scenarios from 1/1/2023, this approach allows for greater accuracy in our results by accounting for yearly variation in outcomes which are relatively rare in each simulation (e.g. maternal death). The difference between outcomes from the SQ scenario and intervention scenarios during the intervention period was calculated by first determining the difference between 2023 and 2030 mean outcomes for each run of the scenarios, then, the mean and 95% CI of these differences across runs.

#### Analysis plan—maximum ability to pay

Maximum ability to pay values were calculated as product of the incremental health impact of a scenario compared to the SQ (e.g. DALYs averted) and the cost-effectiveness threshold (CET), representing the marginal productivity of Malawi’s health system^36^. For each scenario we also report the percentage of the projected total health spending in Malawi during the intervention period (2023–2030) each maximum ability to pay value represents. Annual total national health spending during this time was calculated as the product of the projected population size from the modelled SQ scenario and estimated health spending per capita (2023–2030). Health spending per capita (measured in 2017 USD) was calculated using the estimated health spending per capita in Malawi for 2015 and the projected annualised growth rate in health spending per capita between 2015-2030 estimated by Dieleman et al.^37^ as 0.9%.

#### Ethics and inclusion statement

This study was conducted by a diverse team of researchers, clinicians and policy makers based in both the UK and Malawi. Due to the contextual specificity of both the model and the study’s results, this research is highly relevant to those in Malawi working towards improving the health of mothers and newborns. This research was initially led by JHC as part of their PhD research and developed further in close collaboration with all listed co-authors. The model structure, where possible, has been informed by data and empirical research conducted in Malawi as signified in the reference list and the reference list of the supplementary material. As stated in the methods section, ethical approval for this study was provided by the Malawi College of Medicine research ethics committee.

## Supplementary information

Supplementary Information

Supplementary Information

Supplementary Information

Supplementary Information

## Data Availability

Documentation for all data sources, model parameters, and model assumptions have been provided as supplementary information. Additional documentation relating to the model is available online (https://www.tlomodel.org/writeups.html). The raw data generated from the model which supports the findings described during this manuscript is available in the article and on Zenodo. This data is publicly and freely available for download here: 10.5281/zenodo.14976918.

## Code availability

The source code for the version of the model used to perform the analyses presented here is publicly available for download here: 10.5281/zenodo.14976918. This includes both the model source code and all scripts used to analyse data generated by the model.
