# Supplementary material for: An individual-based modelling study estimating the impact of maternity service delivery on health in Malawi
Source: Nat Commun. 2025 Apr 25;16:3925. doi: 10.1038/s41467-025-59060-2 (PMC12032021; doi:10.1038/s41467-025-59060-2)
Supplement: Supplementary file 1 — Supplementary Information [file 41467_2025_59060_MOESM1_ESM.pdf]

Supplementary information to the manuscript:

An individual-based modelling study estimating the impact of maternity service delivery on health in Malawi

Table of Contents

TABLE OF TABLES **4**

TABLE OF FIGURES **6**

1 –MODEL STRUCTURE AND MODELLED OBSTETRIC AND EPIDEMIOLOGICAL PROCESSES **8**

1.1 MODEL STRUCTURAL OVERVIEW 8
1.1.1 Key approaches in model development 9
1.1.2 Model variables 12
1.2 MODELLING OBSTETRIC PROCESSES AND EPIDEMIOLOGY 27
1.2.1 Model parameters 28
1.2.2 Antenatal processes 30
1.2.3 Intrapartum and birth-related processes 33
1.2.4 Postnatal and neonatal processes 38
1.2.5 Common processes 39

2 – HEALTHCARE MODELLING **54**

2.1 OVERVIEW OF MODELLED HEALTHCARE 54
2.2 MODELLING THE 'THREE DELAYS' 60
2.2.1 Delays one and two 60
2.2.2 Delay three 61
2.2.3 Applying the effect of the three delays 63
2.3 MODELLING THE EMERGENCY OBSTETRIC AND NEWBORN CARE SIGNAL FUNCTIONS 64
2.3.1 Quality of EmONC interventions 64
2.4 ANTENATAL HEALTHCARE 68
2.4.1 Post abortion care 68
2.4.2 Ectopic pregnancy case management 70
2.4.3 Routine antenatal care 70
2.4.4 Antenatal inpatient care 88
2.4.5 Induction of labour 93
2.5 INTRAPARTUM AND IMMEDIATE NEWBORN 93
2.5.1 Care seeking 93
2.5.2 Intervention delivery 100
2.6 POSTNATAL HEALTHCARE 104
2.6.1 Maternal postnatal care 105
2.6.2 Neonatal postnatal care 114
2.6.3 Obstetric fistula case management 119

3 – HEALTH CONDITION MODELLING **122**

3.1 MATERNAL COMPLICATION MODELS 122
3.1.1 Ectopic Pregnancy 122
3.1.2 Spontaneous and induced abortion 128
3.1.3 Maternal Anaemia 140
3.1.4 Gestational diabetes 149
3.1.5 Syphilis 155
3.1.6 Premature rupture of membranes 158
3.1.7 Preterm and post term labour 161
3.1.8 Maternal sepsis 167
3.1.9 Antepartum Haemorrhage 180
3.1.10 The Hypertensive Disorders of Pregnancy 190
3.1.11 Obstructed labour 208
3.1.12 Uterine rupture 215
3.1.13 Postpartum haemorrhage 222
3.1.14 Stillbirth 234
3.1.15 Obstetric Fistula 243

3.2 Neonatal complication models 246
3.2.1 Complications of prematurity 246
3.2.2 Neonatal encephalopathy and neonatal respiratory depression 257
3.2.3 Neonatal sepsis 266
3.2.4 Congenital birth anomalies 276

4 – MODEL VERIFICATION AND VALIDATION 282

4.1 Verification Methods 282
4.2 Validation Methods 284
4.2.1 Face validation 284
4.2.2 Model calibration methods 285
4.2.3 Additional model calibration results 294

5 – ADDITIONAL ANALYSES FIGURES AND TABLES 330

SUPPLEMENTARY REFERENCES 340

Table of Tables

Table S1 – Maternal and perinatal conditions included in the model 11
Table S2 – Maternal epidemiological and obstetric variables stored in the population data frame 16
Table S3 – Neonatal epidemiological variables stored in the population data frame 18
Table S4 – Maternal healthcare variables stored in the population data frame 21
Table S5 – Neonatal healthcare variables stored in the population data frame 21
Table S6 – Maternal variables stored in the mother_and_newborn_care dictionary 26
Table S7 – Neonatal variables stored in the newborn_care_info dictionary 27
Table S8 – Parameter determining rate of twin birth 30
Table S9 – Parameters determining neonatal birthweight 34
Table S10 – Parameters determining the breastfeeding status of newborns 37
Table S11 – Parameters determining baseline obstetric history 42
Table S12 – Disability weights for maternal conditions included in the model 46
Table S13 – Disability weights for neonatal conditions included in the model 50
Table S14 – Parameters representing the probability of impairment in neonates who experience modelled conditions 53
Table S15 – Interventions included in the model 59
Table S16 – Parameter representing delay in healthcare seeking 61
Table S17 – Parameters representing 'squeeze factor thresholds' for the modelled health system interactions 62
Table S18 – Parameters representing the effect of delayed care on treatment effectiveness 63
Table S19 – Parameters related to quality of B/CEmONC interventions delivered during inpatient care 67
Table S20 – Parameter for care seeking following abortion. 68
Table S21 – Details of Post Abortion Care (PAC) health system interaction within the MPHM 69
Table S22 – Details of ectopic pregnancy case management health system interaction within the MPHM 70
Table S23 – WHO 2016 ANC schedule by recommended gestational age at attendance 71
Table S24- Parameters relating to care seeking for ANC 79
Table S25- Interventions delivered during ANC 83
Table S26 – Parameters relating to quality of ANC and sensitivity and specific of screening interventions 87
Table S27 - Parameter representing care seeking following antenatal complications 88
Table S28 – Summary of interventions delivered as part of inpatient antenatal care for relevant antenatal conditions 92
Table S29 – Parameter representing care seeking for induction of labour. 93
Table S30 – Parameters representing care seeking intrapartum care 99
Table S31 – Interventions delivered as part of intrapartum care 103
Table S32 – Parameters representing the probabilities of operative delivery in mothers without modelled indications 104
Table S33 – Parameters relating to care seeking for maternal PNC 110
Table S34- Interventions available to mothers during PNC 113
Table S35 – Parameters representing care seeking following neonatal complications 115
Table S36 – Interventions available to newborns during postnatal care 118
Table S37 – Parameter representing the probability of KMC for a low-birth-weight newborn 119
Table S38 – Parameters relating to care seeking following obstetric fistula 120
Table S39 – Details of obstetric fistula case management health system interaction within the MPHM 121
Table S40 – Parameters of the ectopic pregnancy model 127

TABLE S41 – PARAMETERS OF THE SPONTANEOUS AND INDUCED ABORTION MODELS 139
TABLE S42 – PARAMETERS FOR THE ANAEMIA MODEL 148
TABLE S43 – PARAMETERS OF THE GESTATIONAL DIABETES MODEL 154
TABLE S44 – PARAMETER OF THE SYPHILIS MODEL 157
TABLE S45 – PARAMETER OF THE PROM MODEL 160
TABLE S46 – PARAMETERS OF THE PRETERM AND POST TERM LABOUR MODEL 166
TABLE S47 -PARAMETERS OF THE MODELS FOR MATERNAL SEPSIS 179
TABLE S48 – PARAMETERS OF THE ANTEPARTUM AND INTRAPARTUM HAEMORRHAGE MODEL 189
TABLE S49 – PARAMETERS FOR THE HYPERTENSIVE DISORDERS OF PREGNANCY MODEL 207
TABLE S50- PARAMETERS FOR THE OBSTRUCTED LABOUR MODEL 214
TABLE S51 PARAMETERS OF THE UTERINE RUPTURE MODEL 221
TABLE S52 – PARAMETERS FOR THE POSTPARTUM HAEMORRHAGE MODEL 233
TABLE S53 – PARAMETERS OF THE STILLBIRTH MODELS 242
TABLE S54 – PARAMETERS OF THE OBSTETRIC FISTULA MODEL 245
TABLE S55 – PARAMETERS DESCRIBING PRETERM RESPIRATORY DISTRESS MODEL 252
TABLE S56 – PARAMETERS DESCRIBING TREATMENT AND RISK OF DEATH ASSOCIATED WITH UNMODELLED CAUSES OF PRETERM MORTALITY 254
TABLE S57 – PARAMETERS DESCRIBING PROBABILITY OF ROP IN PRETERM NEONATES 256
TABLE S58 – PARAMETERS OF THE NEONATAL ENCEPHALOPATHY MODEL 265
TABLE S59 – PARAMETERS OF THE NEONATAL SEPSIS MODEL 275
TABLE S60 – PARAMETERS OF THE CONGENITAL BIRTH ANOMALY MODEL 281
TABLE S61- OUTCOMES AND THE DATA SOURCES USED TO CALIBRATE THE MPHM 292
TABLE S62- YEARLY PREGNANCIES, BIRTHS, STILLBIRTHS, MATERNAL DEATHS AND NEONATAL DEATHS DURING THE STATUS QUO SCENARIO 2010-2030 332
TABLE S63- MAXIMUM ABILITY TO PAY FOR MODELLED SCENARIOS 333

Table of Figures

FIGURE S1 – RISK APPLICATION DURING THE ANTENATAL PERIOD OF PREGNANCY. 32
FIGURE S2 – RISK APPLICATION DURING THE POSTNATAL AND NEONATAL PERIODS. 38
FIGURE S3 – SUMMARY OF MODELLED ANTENATAL AND INTRAPARTUM HEALTH SYSTEM INTERACTIONS 55
FIGURE S4 – SUMMARY OF THE MODELLED POSTNATAL HEALTH SYSTEM INTERACTIONS. 56
FIGURE S5 – CARE SEEKING FOR ROUTINE ANC WITHIN THE MODEL 72
FIGURE S6 – DIAGRAMMATIC REPRESENTATION OF PNC SCHEDULING IN THE MODEL 107
FIGURE S7 – MODEL OF ECTOPIC PREGNANCY 124
FIGURE S8 – MODEL OF SPONTANEOUS ABORTION 131
FIGURE S9 – MODEL OF INDUCED ABORTION 134
FIGURE S10 – MODEL OF ANTENATAL ANAEMIA 141
FIGURE S11 – MODEL OF POSTNATAL ANAEMIA 142
FIGURE S12 – MODEL OF GESTATIONAL DIABETES MELLITUS 150
FIGURE S13 – MODEL OF SYPHILIS 156
FIGURE S14 – MODEL OF PREMATURE RUPTURE OF MEMBRANES 159
FIGURE S15 – MODEL OF POST TERM AND PRETERM BIRTH 163
FIGURE S16 – MODEL OF ANTENATAL SEPSIS 170
FIGURE S17 – MODEL OF INTRAPARTUM SEPSIS 172
FIGURE S18 – MODEL OF POSTNATAL SEPSIS 174
FIGURE S19 – MODEL OF ANTEPARTUM HAEMORRHAGE 182
FIGURE S20 – MODEL OF INTRAPARTUM HAEMORRHAGE 183
FIGURE S21 -MODEL OF GESTATIONAL HYPERTENSION 192
FIGURE S22 – MODEL OF PRE-ECLAMPSIA (ANTENATAL) 193
FIGURE S23 – MODEL OF PRE-ECLAMPSIA (POSTNATAL) 194
FIGURE S24 – MODEL OF OBSTRUCTED LABOUR 210
FIGURE S25 – MODEL OF UTERINE RUPTURE 216
FIGURE S26 – MODEL OF PRIMARY POSTPARTUM HAEMORRHAGE 224
FIGURE S27 – MODEL OF SECONDARY POSTPARTUM HAEMORRHAGE 225
FIGURE S28 – MODEL OF PRETERM RESPIRATORY DISTRESS SYNDROME 248
FIGURE S29 – MODEL OF NEONATAL ENCEPHALOPATHY AND NEONATAL RESPIRATORY DEPRESSION 260
FIGURE S30- MODEL OF EARLY-ONSET NEONATAL SEPSIS 268
FIGURE S31 – MODEL OF LATE-ONSET NEONATAL SEPSIS 269
FIGURE S32- MODEL OF CONGENITAL BIRTH ANOMALIES 278
FIGURE S33 – MODEL OUTPUT OF THE DIRECT MMR PER YEAR 295
FIGURE S34 – MODEL OUTPUT OF TOTAL DALYS PER YEAR ATTRIBUTABLE TO MATERNAL DISORDERS 296
FIGURE S35 – MODEL OUTPUT OF THE ANTENATAL AND INTRAPARTUM SBR PER YEAR 297
FIGURE S36 – MODEL OUTPUT OF TOTAL DALYS PER YEAR ATTRIBUTABLE TO 'NEONATAL DISORDERS' 299
FIGURE S37 -MODEL OUTPUT OF THE YEARS LIVED WITH DISABILITY GENERATED BY 'NEONATAL DISORDERS' PER YEAR COMPARED TO CALIBRATION DATA 300
FIGURE S38 – MODEL OUTPUT OF THE PERCENTAGE OF WOMEN WHO GAVE BIRTH IN THE LAST YEAR AND RECEIVED ANY ANC DURING PREGNANCY 301
FIGURE S39 – MODEL OUTPUT OF THE PERCENTAGE OF WOMEN WHO GAVE BIRTH IN THE LAST YEAR WHO ATTENDED AT LEAST FOUR ANC VISITS DURING THEIR PREGNANCY 302
FIGURE S40 – MODEL OUTPUT OF THE MATERNAL GESTATIONAL AGE AT FIRST ANC CONTACT.BY YEAR 303
FIGURE S41 – MODEL OUTPUT OF THE NUMBER OF ANC CONTACT ATTENDED BY WOMEN AS A PERCENTAGE OF THE TOTAL WOMEN ATTENDING ONE OR MORE CONTACTS 304
FIGURE S42 – MODEL OUTPUT OF THE PERCENTAGE OF TOTAL BIRTHS OCCURRING IN A HEALTH FACILITY 305
FIGURE S43- MODEL OUTPUT OF THE PERCENTAGE OF TOTAL BIRTHS LOCATION OF DELIVERY 306
FIGURE S44 – MODEL OUTPUT OF THE PERCENTAGE OF TOTAL BIRTHS DELIVERED VIA CAESAREAN SECTION 307

FIGURE S45- MODEL OUTPUT OF THE PERCENTAGE OF WOMEN RECEIVING ANY POSTNATAL CARE FOLLOWING BIRTH 308
FIGURE S46 – MODEL OUTPUT OF THE PERCENTAGE OF NEONATES WHO RECEIVED ANY POSTNATAL CARE AFTER BIRTH COMPARED TO CALIBRATION DATA 309
FIGURE S47 – PERCENTAGE OF TOTAL MODELLED PREGNANCIES ENDING IN A LIVE BIRTH PER YEAR 310
FIGURE S48 – ECTOPIC PREGNANCIES PER 1000 PREGNANCIES PER YEAR IN THE MODEL 310
FIGURE S49 – TWIN BIRTHS PER 100 BIRTHS PER YEAR IN THE MODEL 311
FIGURE S50 – SPONTANEOUS ABORTIONS PER 1000 COMPLETE PREGNANCIES PER YEAR IN THE MODEL 311
FIGURE S51 – INDUCED ABORTIONS PER 1000 COMPLETED PREGNANCIES PER YEAR IN THE MODEL 312
FIGURE S52 – SYPHILIS CASES IN PREGNANCIES PER 1000 COMPLETED PREGNANCIES IN THE MODEL....313
FIGURE S53 – YEARLY RATE OF GESTATIONAL DIABETES MELLITUS WITHIN THE MODEL....313
FIGURE S54 – PREMATURE RUPTURE OF MEMBRANES PER 1000 BIRTHS PER YEAR IN THE MODEL 314
FIGURE S55 – PREVALENCE OF MATERNAL ANAEMIA AT BIRTH PER YEAR IN THE MODEL 314
FIGURE S56 – MILD GESTATIONAL HYPERTENSION CASES PER 1000 BIRTHS PER YEAR IN THE MODEL....315
FIGURE S57 – SEVERE GESTATIONAL HYPERTENSION CASES PER 1000 BIRTHS PER YEAR IN THE MODEL 316
FIGURE S58 – MILD PRE-ECLAMPSIA CASES PER 1000 BIRTHS PER YEAR IN THE MODEL....316
FIGURE S59 – SEVERE PRE-ECLAMPSIA CASES PER 1000 BIRTHS PER YEAR IN THE MODEL 317
FIGURE S60 – ECLAMPSIA CASES PER 1000 BIRTHS PER YEAR IN THE MODEL 317
FIGURE S61 – PLACENTA PRAEVIA PER 1000 PREGNANCIES PER YEAR IN THE MODEL....318
FIGURE S62 – PLACENTAL ABRUPTION PER 1000 BIRTHS PER YEAR IN THE MODEL 318
FIGURE S63 – ANTEPARTUM AND INTRAPARTUM HAEMORRHAGES PER 1000 BIRTHS PER YEAR IN THE MODEL 319
FIGURE S64 – PRETERM BIRTHS PER 100 BIRTHS PER YEAR IN THE MODEL 319
FIGURE S65 – POST TERM BIRTHS PER 100 BIRTHS PER YEAR IN THE MODEL....320
FIGURE S66 – OBSTRUCTED LABOUR PER 1000 BIRTHS PER YEAR IN THE MODEL 320
FIGURE S67 – UTERINE RUPTURES PER 1000 BIRTHS PER YEAR IN THE MODEL....321
FIGURE S68 – MATERNAL SEPSIS PER 1000 BIRTHS PER YEAR IN THE MODEL....322
FIGURE S69 – POSTPARTUM HAEMORRHAGES PER 1000 BIRTHS PER YEAR IN THE MODEL....322
FIGURE S70 – OBSTETRIC FISTULAS PER 1000 BIRTHS PER YEAR IN THE MODEL....323
FIGURE S71 – LOW BIRTH WEIGHT BIRTHS PER 100 BIRTHS PER YEAR IN THE MODEL 324
FIGURE S72 – SMALL FOR GESTATIONAL AGE BIRTHS PER 100 BIRTHS PER YEAR IN THE MODEL 325
FIGURE S73 – MACROSOMIC BIRTHS PER 100 BIRTHS PER YEAR IN THE MODEL 325
FIGURE S74 – NEONATAL SEPSIS PER 1000 BIRTHS PER YEAR IN THE MODEL 326
FIGURE S75 – NEONATAL ENCEPHALOPATHY PER 1000 BIRTHS PER YEAR IN THE MODEL....326
FIGURE S76 – PRETERM RESPIRATORY DISTRESS SYNDROME PER 1000 PRETERM BIRTHS PER YEAR IN THE MODEL ...327
FIGURE S77 – ALL RESPIRATORY COMPLICATIONS PER 100 BIRTHS PER YEAR....327
FIGURE S78 – CONGENITAL BIRTH ANOMALIES PER 1000 BIRTHS PER YEAR IN THE MODEL 328
FIGURE S79 – PERCENTAGE OF TOTAL BIRTHS DELIVERED BY ASSISTED VAGINAL DELIVERY PER YEAR IN THE MODEL ..329
FIGURE S80 – YEARLY MMR, SBR, NMR BY SCENARIO ORGANISED BY SCENARIOS RELATING TO ANTENATAL, INTRAPARTUM, AND POSTNATAL SERVICE DELIVERY 334
FIGURE S81 – AVERAGE RATE OR PREVALENCE OF SELECTED COMPLICATIONS DURING THE INTERVENTION PERIOD WITHIN SCENARIOS RELATING TO DELIVERY OF ANTENATAL SERVICES 335
FIGURE S82 – AVERAGE RATE OR PREVALENCE OF SELECTED COMPLICATIONS DURING THE INTERVENTION PERIOD WITHIN SCENARIOS RELATING TO DELIVERY OF ALL SERVICES....336
FIGURE S83 – AVERAGE RATE OR PREVALENCE OF SELECTED COMPLICATIONS DURING THE INTERVENTION PERIOD WITHIN SCENARIOS RELATING TO DELIVERY OF INTRAPARTUM SERVICES 337
FIGURE S84 – AVERAGE RATE OR PREVALENCE OF SELECTED COMPLICATIONS DURING THE INTERVENTION PERIOD WITHIN SCENARIOS RELATING TO DELIVERY OF ALL SERVICES....338
FIGURE S85 – AVERAGE PREVALENCE OF MATERNAL ANAEMIA AT THE END OF THE POSTNATAL PERIOD WITHIN SCENARIOS RELATING TO DELIVERY OF POSTNATAL SERVICES....339

1 –Model structure and modelled obstetric and epidemiological processes

In this section we provide structural overview of the maternal and perinatal health model (MPHM), followed by description of the obstetric and epidemiological processes which are simulated. The following sections describe modelled healthcare (§2), health conditions (§3) and verification and validation methods (§4) in detail.

1.1 Model structural overview

Figure 5 in the accompanying manuscript is a high-level diagrammatic representation of the MPHM. The MPHM simulates the three key periods of pregnancy; the antenatal period, from conception until the onset of labour, the intrapartum period from labour onset until birth and postnatal period which last from birth until six weeks postpartum. In addition, the neonatal period is simulated which includes the first twenty-eight days of life.

For women in the antenatal period, the model simulates processes related to pregnancy by replicating progression of gestational age, early pregnancy loss, the epidemiology of common maternal pathophysiological conditions associated with pregnancy (referred to as 'complications' of pregnancy throughout this document) including preterm labour onset, the progression of these complications, and the incidence of antenatal stillbirth and antenatal maternal death. In addition, healthcare interventions routinely delivered to women as part of ANC or emergency obstetric care during pregnancy are modelled to replicate current service delivery within Malawi with regards to coverage, access, and quality of services.

For pregnancies which progress to the intrapartum period, the model simulates the obstetric processes of labour and birth alongside signalling to the demography module that a new individual should be appended onto the data frame following successful delivery, representing live birth. In addition, the model generates, stores and updates variables relating to obstetric history, additional information relating to the status of a woman's labour and common complications associated with the intrapartum period. Healthcare interventions routinely delivered to women as part of routine and emergency intrapartum care are designed to replicate current service delivery in Malawi alongside delivery location.

Following labour and delivery, for the remaining six weeks of the postnatal period, the model generates and updates variables relating to time spent within the postnatal period alongside application of risk of common complications which may onset during this time. Routine and emergency postnatal healthcare is modelled and may occur immediately following birth or later in the postnatal period.

For neonates the model generates and stores information relating to health outcomes which may occur across the first 28 days of life and includes healthcare interventions delivered to newborns as part of routine intrapartum care and postnatal care to replicate current service delivery within Malawi.

1.1.1 Key approaches in model development

1.1.1.1 Identifying health conditions to model

During the development of the model, a core set of maternal and perinatal health conditions were identified to explicitly represent within the framework to simulate the epidemiology of ill-health within this population in Malawi. These conditions drive population level rates of maternal and perinatal morbidity, mortality, and Disability-Adjusted Life Years (DALYs). The 2019 Global Burden of Disease (GBD) study was used for preliminary identification of relevant conditions to include in the model (1). In addition, as part of the iterative process of model validation, utilisation of our clinical expertise, led to the identification of several other relevant conditions to include within the model which are key drivers or predictors of outcomes.

The GBD study categorises causes of death and disability within four levels from the broadest categorisation, level one, to most specific, level four (1). Maternal and neonatal conditions are categorised under the level one cause “Communicable, maternal, neonatal and nutritional disease”, the level two cause “Maternal and neonatal disorders” and level three causes of “Maternal Disorders” and “Neonatal Disorders”. Level four maternal disorders include: maternal haemorrhage, maternal sepsis and other maternal infections, maternal hypertensive disorders, maternal obstructed labour and uterine rupture, maternal abortion and miscarriage, ectopic pregnancy, indirect maternal deaths, late maternal

deaths, maternal deaths aggravated by HIV/AIDS and other maternal disorders. Level four neonatal disorders include complications of preterm birth, neonatal encephalopathy secondary to 'birth asphyxia' or trauma, neonatal sepsis and other infections, haemolytic disease and other neonatal jaundice, and 'other' neonatal disorders.

The following conditions listed in the GBD were not included in the model due to very low number of cases reported in the study for Malawi suggesting a very small contribution to the overall disease burden. These include late maternal deaths (e.g., maternal deaths occurring between six weeks and one year after birth), other maternal disorders, haemolytic disease and other neonatal jaundice, and 'other' neonatal disorders (1).

Table S1 lists all conditions included in the model.

**Modelled maternal conditions:**	**Modelled perinatal conditions:**
• Ectopic pregnancy<br>• Spontaneous and induced abortion<br>• Maternal anaemia<br>• Gestational diabetes<br>• Maternal syphilis<br>• Premature rupture of membranes<br>• Preterm and post term labour<br>• Maternal sepsis<br>• Placenta praevia<br>• Placental abruption<br>• Antepartum and intrapartum haemorrhage<br>• Hypertensive disorders of pregnancy *(gestational hypertension, pre-eclampsia/eclampsia)*<br>• Obstructed labour<br>• Uterine rupture<br>• Postpartum haemorrhage *(primary and secondary)*	• Stillbirth *(antenatal and intrapartum)*<br>• Early- and late-onset neonatal sepsis<br>• Neonatal respiratory depression and neonatal encephalopathy<br>• Complications of prematurity *(preterm respiratory distress syndrome, retinopathy of prematurity)*<br>• Congenital birth anomalies *(heart anomalies, limb and musculoskeletal anomalies, urogenital anomalies, digestive anomalies, and "other" anomalies)*

• Obstetric fistula *(rectovaginal and vesicovaginal)*<br>• Indirect maternal deaths†<br>• Maternal death aggravated by HIV/AIDS±	
†Indirect maternal deaths in the model are those occurring in women who are pregnant or within 42 days of birth due to chronic ischemic heart disease, chronic kidney disease, malaria, non-gestational diabetes, suicide, tuberculosis, and stroke. The rates of deaths within the pregnant/postnatal population due to these conditions are generated by other models within the TLO framework not described here. As a simplifying assumption, all deaths due to these causes which occur during pregnancy, or the postnatal period are classified as indirect maternal deaths.<br>± Deaths due to HIV/AIDS occurring during pregnancy/the postnatal period are generated by the TLO HIV model. In line with WHO methodology for MMR estimation we assume that 0.3 of these deaths are due to aggravation of HIV/AIDS by the processes of pregnancy/the postnatal period.	

Table S1 – Maternal and perinatal conditions included in the model

1.1.1.2 Identifying and modelling interactions between variables

A primary advantage of an IBM framework is the ability to model the interaction between variables within and among individuals in the model. In an epidemiological model the modeller can simulate the assumed relationship between an individual's characteristics and behaviours, such as the propensity to seek healthcare, or health outcomes, including the probability of disease acquisition. Additionally, and particularly pertinent within maternal and perinatal epidemiology, the assumed biological relationship between maternal conditions (e.g., the effect of malaria infection on risk of anaemia) and between maternal and perinatal outcomes (e.g., the effect of maternal syphilis infection on risk of perinatal death) can be explicitly represented. We approached the identification of these relationships distinctly in the modelling of healthcare seeking and epidemiological modelling.

For antenatal and intrapartum healthcare seeking, novel analyses were conducted using the Malawian Demographic and Health Survey (DHS) data to identify socio-demographic variables associated with care seeking whilst for postnatal care a previously conducted analysis of the Malawian DHS data was used to inform model parameters (2).

In the modelling of health conditions, to identify relevant relationships between variables several targeted literature searches using the PubMed database were conducted. These reviews were undertaken for each modelled condition focusing on studies from Malawi and nearby territories including Mozambique, Zambia, Tanzania, Uganda, and Kenya, with the search expanded to sub-Saharan Africa (SSA) if no relevant studies were found. Potential relationships were identified based on significant evidence of effect and reasonable biological plausibility suggestive of a causal effect. A compiled list of relationships was then reviewed using our clinical expertise to determine the suitability of including such relationships and to determine if any key relationships missing which should be included (3).

In the MPHM, unless otherwise stated, the effect of a variable on the probability of an outcome is represented through a linear multiplicative model, as shown in the below equation. Here let y be the probability of a complication, β_0 the baseline risk, or intercept value, x_i the indicator variable for a risk factor and β_i the 'effect' of the risk factor giving:

$$y = \beta_0 * (\beta_1 * x_1) * (\beta_2 * x_2) \ldots. \quad (1)$$

Within the description of each complication in §3 the equation used to calculate risk of acquisition is presented and the relationship between variables within the multiplicative models is discussed.

1.1.2 Model variables

Tables S2-S5 contain the individual-level variables generated and managed by the MPHM which are largely representative of health conditions and healthcare related to pregnancy and the neonatal period. Tables S7 and S8 list variables which are stored for individuals outside of the main data frame but within an individual level dictionary for each pregnant individual or each newborn.

Variable	Data Type	Description
ps_gestational_age_in_weeks	Integer	The gestational age in weeks of a woman's pregnancy.
ps_date_of_anc1	Date	The date on which a pregnant woman's first antenatal care (ANC) visit is scheduled.
ps_ectopic_pregnancy	Categorical	Whether a pregnant woman is experiencing an ectopic pregnancy, and if so, its current 'state'. Categories include none, not ruptured, ruptured.
ps_multiple_pregnancy	Boolean	Whether a pregnant woman is pregnant with multiple foetuses.
ps_placenta_praevia	Boolean	Whether a pregnant woman is experiencing placenta praevia in her current pregnancy.
ps_syphilis	Boolean	Whether a pregnant woman has a syphilis infection.
ps_anaemia_in_pregnancy	Categorical	Whether a pregnant woman is experiencing maternal anaemia in the antenatal period, and if so, its current severity. Categories include none, mild, moderate, severe.
ps_abortion_complications	Bitset[1]	The current complications associated with an abortion that a woman is experiencing. Bitset* list values represent the following complications: sepsis, haemorrhage, injury, other.
ps_prev_spont_abortion	Boolean	Whether a woman has had any previous pregnancies which have ended in spontaneous abortion.
ps_prev_stillbirth	Boolean	Whether a woman has had any previous pregnancies which have ended in stillbirth.
ps_htn_disorders	Categorical	Whether a pregnant woman is experiencing any of the hypertensive disorders of pregnancy

[1] A Bitest is an ordered binary set which stored elements through toggling of an array of numbers. In this example the first 0 within the set would indicate not having the first complication and toggling to 1 would indicate having that complication

		during the antenatal period. Categories include none, gestational hypertension, severe gestational hypertension, mild pre-eclampsia, severe pre-eclampsia, eclampsia.
ps_prev_pre_eclamp	Boolean	Whether a woman has had any previous pregnancies which have been complicated by pre-eclampsia.
ps_gest_diab	Categorical	Whether a pregnant woman is experiencing gestational diabetes. Categories include none, uncontrolled, controlled.
ps_prev_gest_diab	Boolean	Whether a woman has had any previous pregnancies which have been complicated by gestational diabetes.
ps_placental_abruption	Boolean	Whether a pregnant woman is currently experiencing antenatal placental abruption.
ps_antepartum_haemorrhage	Categorical	Whether a pregnant woman is currently experiencing an antepartum haemorrhage, and if so, its severity. Categories include none, mild/moderate, severe.
ps_premature_rupture_of _membranes	Boolean	Whether a pregnant woman is experiencing rupture of membranes before the onset of labour.
ps chorioamnionitis	Boolean	Whether a pregnant woman is currently experiencing sepsis due to chorioamnionitis infection.
ps_emergency_event	Boolean	Whether a woman is experiencing an acute emergency event in her pregnancy and requires healthcare.
la_due_date_current_pregnancy	Date	Date on which a pregnant woman's labour will onset if pregnancy continues until this point.
la_currently_in_labour	Boolean	Whether a pregnant woman is currently in labour.
la_intrapartum_still_birth	Boolean	Whether a pregnant woman has experienced an intrapartum stillbirth during her current

		pregnancy. It is reset after the model determines if a birth should occur following labour.
la_parity	Integer	The number of previous births a woman has undergone.
la_obstructed_labour	Boolean	Whether a pregnant woman in labour is currently experiencing obstructed labour.
la_antepartum_haem	Categorical	Whether a pregnant woman in labour is currently experiencing an intrapartum haemorrhage, and if so, its severity. Categories include none, mild/moderate, severe.
la_uterine_rupture	Boolean	Whether a pregnant woman in labour is currently experiencing uterine rupture.
la_sepsis	Boolean	Whether a pregnant woman in labour is currently experiencing intrapartum sepsis.
la_date_most_recent_delivery	Date	The date on which a woman has most recently delivered, inclusive of live birth or intrapartum stillbirth.
la_is_postpartum	Boolean	Whether a woman is currently in the postnatal period meaning less than forty-two days have occurred since she gave birth most recently
la_sepsis_pp	Boolean	Whether a postnatal woman has developed sepsis within the first forty-eight hours after birth.
la_posptpartum_haem	Boolean	Whether a postnatal woman has developed a primary postpartum haemorrhage within twenty-four hours of birth.
pn_postnatal_period_in_weeks	Integer	The current week of the postnatal period for a postnatal woman starting at week one.
pn_htn_disorders	Categorical	Whether a woman is experiencing any of the hypertensive disorders of pregnancy during the postnatal period. Categories include none, gestational hypertension, severe gestational

		hypertension, mild pre-eclampsia, severe pre-eclampsia, eclampsia.
pn_postpartum_haem_secondary	Boolean	Whether a woman in the postnatal period is experiencing a secondary postpartum haemorrhage.
pn_sepsis_late_postpartum	Boolean	Whether a woman in the postnatal period is experiencing a postnatal sepsis that has onset after forty-eight hours.
pn_obstetric_fistula	Categorical	Whether a woman in the postnatal period has developed an obstetric fistula following birth. Categories include none, vesicovaginal, and rectovaginal.
pn_anaemia_following_pregnancy	Categorical	Whether a woman is experiencing maternal anaemia in the postnatal period, and if so, its current severity. Categories include none, mild, moderate, severe.
pn_emergency_event_mother	Boolean	Whether a postnatal woman is undergoing an acute emergency event and requires healthcare.

Table S2 – Maternal epidemiological and obstetric variables stored in the population data frame

**Variable**	**Data Type**	**Description**
nb_is_twin	Boolean	Whether a neonate is a part of a twin pair.
nb_twin_sibling_id	Integer	The unique identifier for the twin-sibling of a neonate within a twin pair.
nb_early_preterm	Boolean	Whether a neonate was born before thirty-four weeks gestational age (GA).
nb_late_preterm	Boolean	Whether a neonate was born between thirty-four- and thirty-six-weeks GA.
nb_preterm_birth_disab	Categorical	Whether a neonate who was born prematurely has developed any level of neurodevelopmental impairment following birth. Categories include none, mild motor and cognitive impairment, mild

		motor impairment, moderate motor impairment, severe motor impairment.
nb_congenital_anomaly	Bitset	Whether a neonate has a congenital birth anomaly. Bitset list values represent the following conditions: cardiac anomaly, limb or musculoskeletal anomaly, urogenital anomaly, digestive anomaly and 'other' anomaly.
nb_early_onset_neonatal_sepsis	Boolean	Whether a neonate has developed early-onset neonatal sepsis immediately after birth.
nb_neonatal_sepsis_disab	Categorical	Whether a neonate who developed sepsis following birth developed any level of neurodevelopmental impairment following birth. Categories include none, mild motor and cognitive impairment, mild motor impairment, moderate motor impairment, severe motor impairment.
nb_preterm_respiratory_distress	Boolean	Whether a neonate who was born prematurely has developed respiratory distress syndrome.
nb_not_breathing_at_birth	Boolean	Whether a neonate is breathing spontaneously following birth.
nb_encephalopathy	Categorical	Whether a neonate has developed neonatal encephalopathy following birth.
nb_encephalopathy_disab	Categorical	Whether a neonate who developed encephalopathy following birth developed any level of neurodevelopmental impairment following birth. Categories include none, mild motor and cognitive impairment, mild motor impairment, moderate motor impairment, severe motor impairment.
nb_retinopathy_prem	Categorical	Whether a preterm neonate has experienced any severity of retinopathy following birth. Categories include none, mild, moderate, severe, blindness.
nb_low_birth_weight_status	Categorical	The birthweight 'category' of a neonate. Categories include macrosomia, normal birth weight, low birth weight, very low birth weight and extremely low birth weight.

nb_size_for_gestational_age	Categorical	The size for GA 'category' of a neonate. Categories include large for GA, average for GA and small for GA.
nb_early_init_breastfeeding	Boolean	Whether a neonate has started breastfeeding within the first hour of life.
nb_breastfeeding_status	Categorical	The breastfeeding 'statuses of a neonate. Categories include none, non-exclusive and exclusive.
pn_sepsis_early_neonatal	Boolean	Whether a neonate has developed early onset neonatal sepsis during the first week of life.
pn_sepsis_late_neonatal	Boolean	Whether a neonate has developed late onset neonatal sepsis, sepsis occurring after 7 days of life.

Table S3 – Neonatal epidemiological variables stored in the population data frame

Variable	Data Type	Description
ps_anc4	Boolean	Whether a pregnant woman is predicted to attend four or more antenatal care visits during her current pregnancy.
ac_total_anc_visits_current_pregnancy	Integer	The running total of ANC visits a pregnant woman has attended during their current pregnancy.
ac_date_next_contact	Date	Scheduled date on which the next ANC visit a pregnant woman will attend will occur.
ac_to_be_admitted	Boolean	Whether a pregnant woman will be admitted as result of ANC attendance. Set to True on the day on which admission occurs then reset.
ac_receiving_iron_folic_acid	Boolean	Whether a pregnant woman is currently receiving daily iron and folic acid supplementation.

ac_receiving_bep_supplements	Boolean	Whether a pregnant woman is currently receiving daily balanced energy and protein supplementation.
ac_receiving_calcium_supplements	Boolean	Whether a pregnant woman is currently receiving daily calcium supplementation.
ac_gest_htn_on_treatment	Boolean	Whether a pregnant woman is currently receiving daily oral medication for treatment of hypertension during pregnancy.
ac_gest_diab_on_treatment	Categorical	Whether a pregnant woman is receiving treatment for gestational diabetes and, if so, which treatment. Categories include none, diet and exercise, orals and insulin.
ac_ectopic_pregnancy_treated	Boolean	Whether a woman with an ectopic pregnancy has received treatment as part of ectopic pregnancy case management. Reset to False after risk of death has been calculated.
ac_received_post_abortion_care	Boolean	Whether a woman experiencing complications of abortion has received post abortion care. Reset to False after risk of death has been calculated.
ac_received_abx_for_prom	Boolean	Whether a woman experiencing premature rupture of membranes (PROM) has received prophylactic antibiotics. Reset to False after delivery.
ac_mag_sulph_treatment	Boolean	Whether a woman experiencing severe pre-eclampsia or eclampsia has received treatment with magnesium sulphate. Reset to False after risk of death has been calculated.
ac_iv_anti_htn_treatment	Boolean	Whether a pregnant woman experiencing severe hypertension in pregnancy has received intravenous treatment with antihypertensives. Reset to False after risk of death has been calculated.

ac_admitted_for_immediate_delivery	Categorical	Whether a pregnant woman has been admitted to the labour ward for delivery and, if so, by what method. Categories include none, induction now, induction future, caesarean now or caesarean future.
la_previous_cs_delivery	Integer	Whether a woman has ever previously delivered via caesarean section.
la_uterine_rupture_treatment	Boolean	Whether a pregnant woman in labour experiencing uterine rupture has received treatment.
la_sepsis_treatment	Boolean	Whether a pregnant woman in labour currently experiencing intrapartum sepsis has received treatment.
la_eclampsia_treatment	Boolean	Whether a pregnant woman in labour currently experiencing eclampsia has received treatment.
la_severe_pre_eclampsia_treatment	Boolean	Whether a pregnant woman in labour currently experiencing severe pre-eclampsia has received treatment.
la_maternal_hypertension_treatment	Boolean	Whether a pregnant woman in labour currently experiencing severe hypertension has received treatment.
la_has_had_hysterectomy	Boolean	Whether a woman has undergone a hysterectomy following labour and delivery as a treatment for uterine rupture or refractory postpartum haemorrhage.
la_postpartum_haem_treatment	Bitset	The treatment received by a postnatal woman who has experienced postpartum haemorrhage and received care. Bitset list values represent the following complications: manual removal of placenta, uterine preserving surgery, and hysterectomy.
la_pn_checks_maternal	Integer	Total number of postnatal care (PNC) visits a woman in the postnatal period has received.

la_gest_htn_on_treatment	Boolean	Whether a woman with postnatal hypertension is taking oral antihypertensives.
la_iron_folic_acid_postnatal	Boolean	Whether a woman in the postnatal period is taking daily iron and folic acid supplementation.

Table S4 – Maternal healthcare variables stored in the population data frame

**Variable**	**Data Type**	**Description**
nb_received_neonatal_resus	Boolean	Whether a neonate has received basic neonatal resuscitation following birth.
nb_clean_birth	Boolean	Whether clean birth and postnatal practices were adhered to during the delivery of a neonate.
nb_inj_abx_neonatal_sepsis	Boolean	Whether a neonate with sepsis has received injectable antibiotics as treatment for their condition.
nb_supp_care_neonatal_sepsis	Boolean	Whether a neonate with sepsis has received full supportive care as treatment for their condition.
nb_kangaroo_mother_care	Boolean	Whether a neonate has received Kangaroo Mother Care (KMC).
nb_pnc_check	Integer	The number of PNC visits a neonate has undergone during the neonatal period.

Table S5 – *Neonatal healthcare variables stored in the population data frame*

Variables used only for programming purposes are not listed here.

Variable	Data Type	Description
delay_one_two	Boolean	Whether a pregnant or postnatal woman has experienced delay one or two before receiving care.
delay_three	Boolean	Whether a pregnant or postnatal woman has experienced delay three during care.
ga_anc_one	Integer	The gestational age of a pregnant woman's pregnancy when she attends her first ANC visit.
abortion_onset	Date	Date on which an abortion has occurred to a previously pregnant woman.
abortion_haem_onset	Date	Date on which an abortion complicated by haemorrhage has occurred to a previously pregnant woman.
abortion_sep_onset	Date	Date on which an abortion complicated by sepsis has occurred to a previously pregnant woman.
eclampsia_onset	Date	Date on which eclampsia has occurred to a pregnant or postnatal woman.
mild_mod_aph_onset	Date	Date on which a mild/moderate antepartum haemorrhage has occurred to a pregnant woman.
severe_aph_onset	Date	Date on which a severe antepartum haemorrhage has occurred to a pregnant woman.
chorio_onset	Date	Date on which sepsis secondary to chorioamnionitis has occurred to a pregnant woman.
ectopic_onset	Date	Date on which an ectopic pregnancy has occurred to a pregnant woman.
ectopic_rupture_onset	Date	Date on which a ruptured ectopic pregnancy has occurred to a pregnant woman.
gest_diab_onset	Date	Date on which gestational diabetes has onset in a pregnant woman.

gest_diab_diagnosed_onset	Date	Date on which a pregnant woman with gestational diabetes was diagnosed.
gest_diab_resolution	Date	Date on which gestational diabetes resolved in a pregnant woman who was previously suffering from gestational diabetes.
mild_anaemia_onset	Date	Date on which mild anaemia has onset in a pregnant woman.
mild_anaemia_resolution	Date	Date on which mild anaemia has resolved in a pregnant woman.
moderate_anaemia_onset	Date	Date on which moderate anaemia has onset in a pregnant woman.
moderate_anaemia_resolution	Date	Date on which moderate anaemia has resolved in a pregnant woman.
severe_anaemia_onset	Date	Date on which severe anaemia has onset in a pregnant woman.
severe_anaemia_resolution	Date	Date on which severe anaemia has resolved in a pregnant woman.
mild_anaemia_pp_onset	Date	Date on which mild anaemia has onset in a postnatal woman.
mild_anaemia_pp_resolution	Date	Date on which mild anaemia has resolved in a postnatal woman.
moderate_anaemia_pp_onset	Date	Date on which moderate anaemia has occurred to a postnatal woman.
moderate_anaemia_pp_resolution	Date	Date on which moderate anaemia has resolved in a postnatal woman.
severe_anaemia_pp_onset	Date	Date on which severe anaemia has onset in a postnatal woman.
severe_anaemia_pp_resolution	Date	Date on which severe anaemia has resolved in a postnatal woman.

hypertension_onset	Date	Date on which hypertension has onset in a pregnant or postnatal woman.
hypertension_resolution	Date	Date on which hypertension has resolved in a pregnant or postnatal woman.
obstructed_labour_onset	Date	Date on which a pregnant woman in labour has experienced obstructed labour.
sepsis_onset	Date	Date on which a pregnant woman in labour or postnatal woman has experienced sepsis.
uterine_rupture_onset	Date	Date on which a pregnant woman in labour has experienced uterine rupture.
mild_mod_pph_onset	Date	Date on which a postnatal woman has experienced mild/moderate postpartum haemorrhage.
severe_pph_onset	Date	Date on which a postnatal woman has experienced severe postpartum haemorrhage.
secondary_pph_onset	Date	Date on which a postnatal woman has experienced a secondary postpartum haemorrhage.
vesicovaginal_fistula_onset	Date	Date on which a postnatal woman has experienced a vesicovaginal fistula.
vesicovaginal_fistula_resolution	Date	Date on which a postnatal woman has experienced resolution of their vesicovaginal fistula.
rectovaginal_fistula_onset	Date	Date on which a postnatal woman has experienced a rectovaginal fistula.
rectovaginal_fistula_resolution	Date	Date on which a postnatal woman has experienced resolution of their rectovaginal fistula.
pred_syph_infect	Date	Date on which syphilis will onset in a pregnant woman.

labour_state	Categorical	Whether a pregnant woman in labour is in term, preterm or post term labour. Categories include term labour, early preterm labour, late preterm labour, or post term
birth_weight	Categorical	Birth weight category of a pregnant woman's foetus at the time of labour onset. Categories include macrosomia, normal birth weight, low birth weight, very low birth weight and extremely low birth weight.
birth_size	Categorical	Size category of a pregnant woman's foetus at the time of labour onset. Categories include large for GA, average for GA and small for GA.
delivery_setting	Categorical	Where a pregnant woman in labour will give birth. Categories include home, health centre and hospital.
corticosteroids given	Boolean	Whether a pregnant woman in preterm labour has received antenatal corticosteroids.
clean_birth_practices	Boolean	Whether a pregnant woman in labour has received clean birth practices.
abx_for_prom_given	Boolean	Whether a pregnant woman has received antibiotic prophylaxis after PROM.
endo_pp	Boolean	Whether a postnatal woman has experienced sepsis secondary to endometritis.
retained_placenta	Boolean	Whether a postnatal woman is experiencing retained placenta after birth.
uterine_atony	Boolean	Whether a postnatal woman is experiencing uterine atony after birth.
amtsl_given	Boolean	Whether a postnatal woman has received active management of third stage of labour following birth
cpd	Boolean	Whether a pregnant woman in labour is experiencing cephalopelvic disorder.

mode_of_delivery	Categorical	The mode of birth for a postnatal woman. Categories include vaginal delivery, assisted vaginal delivery, caesarean section.
neo_will_receive_resus_if_needed	Boolean	Whether the newborn of a postnatal woman can receive resuscitation if required as the required consumables and HCWs are available.
received_blood_transfusion	Boolean	Whether a pregnant or postnatal woman has received a blood transfusion during or following labour.
will_receive_pnc	Boolean	Whether a postnatal woman will receive postnatal care and when. Categories include none, early or late.

Table S6 – *Maternal variables stored in the mother_and_newborn_care dictionary*

**Variable**	**Data Type**	**Description**
ga_at_birth	Integer	Gestational age, in weeks, of a neonate at birth.
maternal_chorio	Boolean	Whether a neonate's mother had chorioamnionitis during pregnancy/labour.
maternal_gest_diab	Boolean	Whether a neonate's mother had gestational diabetes.
vit_k	Boolean	Whether a neonate received vitamin K prophylaxis after birth.
tetra_eye_d	Boolean	Whether a neonate received eye care after birth.
abx_for_prom_given	Boolean	Whether a neonate's mother was given antibiotics following PROM.
corticosteroids_given	Boolean	Whether a neonate's mother was given antenatal corticosteroids following preterm labour onset.

delivery_setting	Categorical	Where a neonate was born. Categories include home, health centre and hospital.
cause_of_death_after_birth	List	List of any potential causes of death which a neonate is at risk of following complication risk onset
sepsis_postnatal	Boolean	Whether a neonate experienced sepsis during the neonatal period.
passed_through_week_one	Boolean	Whether *PostnatalWeekOneNeonatalEvent* has run for a neonate.
will_receive_pnc	Categorical	Whether a neonate will receive PNC and at which time. Categories include none, early and late
third_delay	Boolean	Whether a neonate has experienced the third delay during care.

Table S7 – *Neonatal variables stored in the newborn_care_info dictionary*

1.2 Modelling obstetric processes and epidemiology

In this section the modelling of obstetric processes (e.g., calculation and progression of gestational age) and epidemiological processes (e.g., the application of the risk of complication onset) within the population of interest is described. For clarity processes are categorised according to the period of pregnancy to which they are relevant followed by common processes across the model.

1.2.1 Model parameters

Model parameters are presented in tables throughout this and the following two sections, accompanied by a description and the relevant data sources used to derive each parameter. Parameter rows are colour coded to aid interpretation:

- Green – the parameter represents the probability of maternal or neonatal health condition onset or condition severity
- Pink – the parameter is used to determine a pregnancy or birth related characteristic (e.g., foetal birth weight)
- Blue – the parameter represents the probability of an individual seeking healthcare or is related directly to healthcare seeking
- Grey – the parameter is the effect of a variable on a behaviour or outcome
- Yellow – the parameter is the effect of treatment on an outcome or is related directly to treatment delivery or effectiveness
- Orange – the parameter represents risk of mortality associated with a complication

In addition, parameters which are only applied once at simulation baseline are identified. Finally, the names of any parameters which have been estimated through the process of calibration have been underlined and highlighted in yellow within the relevant parameter table with additional information provided in the parameter description.

1.2.1.1 Multiple values for a given parameter

Due to limited availability of historic time-series data in Malawi for the calibration of the model we have opted to calibrate to data points from 2010 and 2015 for which there are reliable estimates. This decision was made to reflect historic changes in key outcomes such as mortality and healthcare coverage.

As such, within the parameter tables, where two values are presented the first represents the value applied in the model between 2010 and 2014 and the second value represents the value applied from 2015 onwards. If a single value is presented, there is no change in that parameter following initialisation of the simulation. Model calibration is discussed at length in §4.

1.2.1.2 Parameter scaling

Several parameters which act as the intercept value within the multiplicative models used to model relationships between variables are scaled at the initialisation of any simulation run. This allowed for calculation of an unknown intercept value of a multiplicative model given the known coefficients, known 'target' probability of that outcome within the population but unknown distribution of variables within the model in the simulated population.

To calculate the scaled intercept value, which is the probability of an outcome in an individual in the absence of the effect of predictor variables, the multiplicative model is solved for all women of reproductive age currently in the data frame, using an intercept of 1, and then calculate a scaled intercept as:

$$Scaled\ intercept = 1 * \frac{target\ intercept}{mean\ result} \tag{2}$$

The parameter **odds_deliver_in_health_centre** (Table S23) is an example of a parameter which is scaled at initialisation and acts as an intercept value in a multiplicative model. The coverage of health centre delivery which should be replicated by the model is 52% in 2015 (sourced from the DHS). The value of this parameter (1.09[2]) would lead to the correct probability of delivery in a health centre if there was no effect of predictor variables. However several predictor variables have been found to affect odds of health centre delivery (e.g. age, parity etc.) and are included in the multiplicative model. At initialisation the distribution of these variables in the modelled population is not known. Therefore the true value that **odds_deliver_in_health_centre** should take, given the effect of these variables and their unknown distribution in the population, to still produce the correct coverage is not known. Using the method above a scaled intercept is calculated and replaces the value for **odds_deliver_in_health_centre** to be used in the model.

Parameters which are scaled at initialisation are highlighted appropriately within the parameter tables.

---

[2] Odds converted to probability as 1.09/2.09 = 0.52

1.2.2 Antenatal processes

1.2.2.1 Initiation of multiple pregnancy

At the initiation of any pregnancy, the parameter **prob_multiples** determines if the individual is pregnant with one or two foetuses. Twin pregnancy has been included in the model due to its assumed causal relationship with other modelled complications and perinatal outcomes. In the interest of parsimony, and due to lacking data in Malawi, it is assumed all multiple pregnancies are twins and exclude the possibility of greater than two foetuses. All twin pregnancies carried successfully to delivery lead to the generation of two individuals following birth in a simulation run.

Parameter name	Description	Value	Data source and/or relevant calculations
**prob_multiples**	The probability that a pregnant individual is pregnant with two foetuses	0.0399	The number of twin births in Malawi was reported in the 2010 DHS, a nationally representative population-level survey, from which Monden & Smits (4) estimate the prevalence of twin births in Malawi. The authors report that 3.9% of births are twin births. This parameter was derived from calibration to this rate.

Table S8 – Parameter determining rate of twin birth

1.2.2.2 Determining gestational age of pregnancy

Gestational age (GA) is the primary measure of pregnancy duration utilised in both clinical obstetrics and maternal and foetal epidemiological research. Commonly, GA is calculated by from the date of woman’s last menstrual period (5). As such, the GA of a pregnancy includes two weeks in which an individual is not pregnant, given that ovulation occurs approximately fourteen days following the end of a woman’s period. Therefore, in the model, GA is updated on a weekly time step calculated by taking the difference, in weeks, between the date of conception and the current simulation date and adding fourteen days. These fourteen days are the additional two weeks before which the individual became pregnant, a date which is not recorded in the model. Whilst foetal age from conception in the model is known, simulating GA of a modelled pregnancy is important considering most of the epidemiological and obstetric research data used to inform the model parameter values utilises this measure.

1.2.2.3 Risk of antenatal complication onset

In the pregnant population of the model, complication onset is determined by GA and risk is applied mostly on a monthly time-step as pregnancy progresses unless otherwise stated in the condition models descriptions in §3. Figure S1 shows the time points during the antenatal period of pregnancy at which risk of modelled complications is applied. The rationale for these decisions is explored in the associated model descriptions in §3. Individual risk of condition onset is determined either by a fixed parameter or calculated via a multiplicative model to account for the effect of relevant variables on probability of onset.

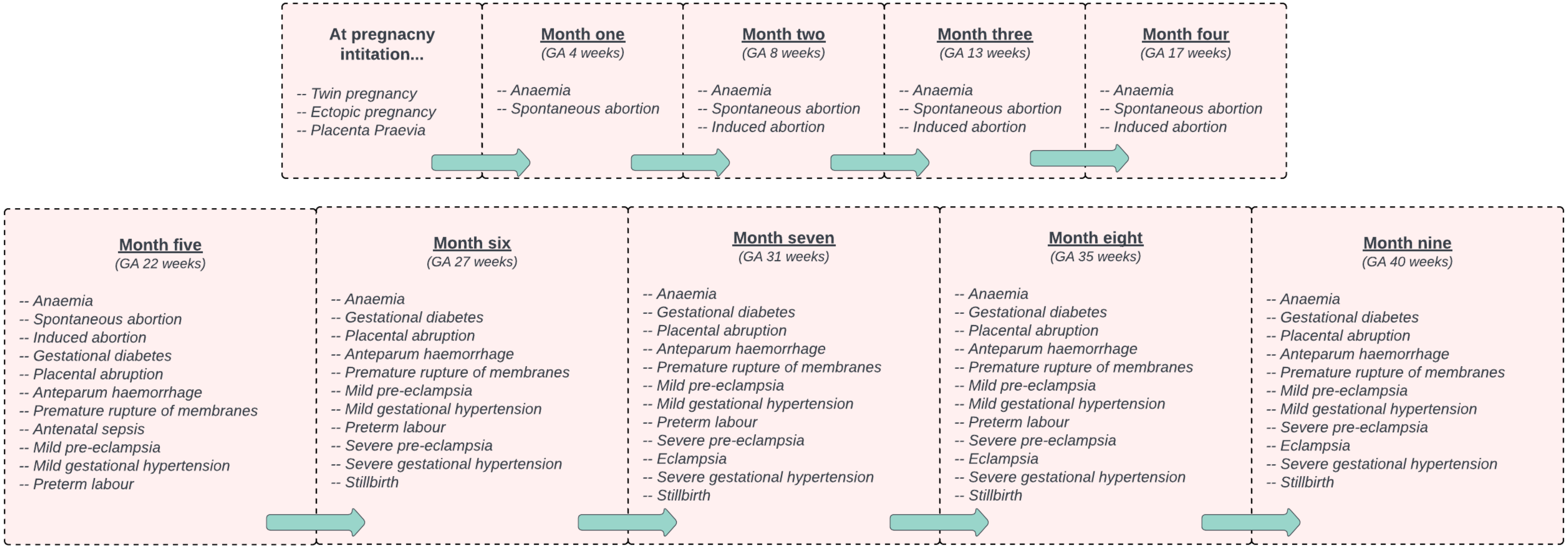

Figure S1 – Risk application during the antenatal period of pregnancy.
Time points during the antenatal period of pregnancy at which risk of modelled complications is applied. Each box represents a new time step in which risk of acquisition is applied as a pregnancy progresses.

1.3.2.4 Antenatal pregnancy loss

The explicit causes of pregnancy loss included in the model are ectopic pregnancy, spontaneous abortion, induced abortion, and antenatal stillbirth. Figure S1 demonstrates how the application of risk of pregnancy loss changes as a woman's pregnancy progresses, with early pregnancy loss categorised predominantly as ectopic pregnancy or abortion and later pregnancy loss being due to stillbirth. Following the onset of any of these conditions in the model, a woman's pregnancy is ended with all relevant variables updated by the model. Through the combined incidence of pregnancy loss within the model the emergent proportion of pregnancies leading to a live birth is ~71% per year by 2022.

1.2.3 Intrapartum and birth-related processes

1.2.3.1 Birthweight

The weight in grams of a foetus is calculated prior to birth as foetal weight is a predictor within multiplicative models of certain maternal complications (e.g., obstructed labour, postpartum haemorrhage). Weight is determined via a random draw from a normal distribution with an estimated mean and standard deviation value of foetal weight for GA shown in Table S9 (parameters **mean_birth_weights** and **standard_deviation_birth_weights).** Values were sourced from Malawian (6) and the United States (US) (7) studies of birthweight by GA. The resultant weight in grams is used to categorise the foetus as extremely low birth weight (< 1000g), very low birth weight (1000 – 1499g), low birth weight (1500 – 2499g), normal birth weight (2500g-3999g) and macrosomic (>4000g) (8) with the categorisation stored as a variable of the mother initially and then the newborn if live birth occurs. As it was found that the assumed distribution did not generate the correct assumed rate of macrosomia within the population, **residual_prob_of_macrosomia** is used to reset a proportion of the normal birthweight newborns as macrosomic. In addition to birthweight, size for GA is also recorded for each foetus with foetuses whose weight is in the 10th centile or lower for their GA and those above the 90th centile being categorised as small and large for GA respectively (9).

Parameter name	Description	Value	Data source and/or relevant calculations
**mean_birth_weights**	The mean birthweights in grams of neonates born at 24 to 41 weeks GA.	657, 746, 851, 966, 1096, 1240, 1440, 1641, 1841, 2041, 2242, 2442, 2736, 2856, 2995, 3036, 3117, 3136	We were unable to identify nationally representative population level data on newborn birthweight in grams by GA in Malawi. Data of observed mean birthweights in newborns born from 24 to 29 weeks was taken directly from a study conducted in the US of over 180,000 preterm infants by Boghossian et al (7) data for mean birthweights for 35 to 41 weeks are taken directly from a study conducted In Malawi of 1,800 infants by Kalanda et al. (6). Malawian data was presented by gender, so the average birthweight was calculated for these values. Birth weights for 30-34 weeks were estimated via linear interpolation using the Python Pandas library.
**standard_deviation_birth_weights**	The standard deviation of birthweight in grams of neonates born at 24 to 41 weeks GA.	113, 140, 169, 196, 218, 235, 274, 312 ,351, 390, 428, 460, 467, 398, 356, 351, 396, 351	See **mean_birth_weights.** Standard deviations were taken from the same studies as referenced above with values for 30-34 weeks estimated via linear interpolation using the python Pandas library.
**residual_prob_of_macrosomia**	The probability of macrosomic birth weight in a neonate who is not low birth weight.	0.057	This proportion was derived from Ngwira (10) who report approximately 5.13% of newborns in Malawi are born macrosomic according to the Malawi DHS data.

Table S9 – Parameters determining neonatal birthweight

1.2.3.2 Risk of intrapartum complication onset

Risk of maternal intrapartum complications is applied to all mothers at labour onset. Complication risk is applied sequentially for relationships between intrapartum complications to be represented (i.e., risk of obstructed labour is applied before the risk of uterine rupture as there is an assumed causal pathway between these two complications). Mothers delivering in a healthcare facility have risk of complication onset applied following the delivery of relevant prophylactic treatments to allow for the effect of these treatments to be applied.

1.2.3.3 Intrapartum pregnancy loss

All mothers who go into labour are at risk of intrapartum stillbirth which is applied immediately prior to the generation of a new live birth within the simulation. In §3 the modelling of stillbirth is described in detail.

1.2.3.4 Birth

Within the model, a birth occurs following the process of labour and leads to the generation of a new individual if a live birth has occurred. Newly generated individuals are appended onto the end of the population data frame with newborns linked to mothers via a unique identifier stored as a variable of the newborn. On birth, variables of the newborn are updated by each disease module. These modules propagate the new rows of the data frame with the relevant variables for that module. Due to the link between mother and newborn, maternal characteristics can be used to determine the status of newborn variables to reflect processes such as vertical transmission (i.e., newborn HIV acquisition) or the relationship between labour and newborn complications.

1.2.3.5 Breastfeeding

Breastfeeding status is determined for all newborns following birth. A probability-weighted random draw, using parameter **prob_breastfeeding_type**, is used to select between three distinct breastfeeding 'states' including 'none', 'non-exclusive' and, 'exclusive' representing neonates who are never breastfed, those who are breastfed alongside additional feeds/fluids, and those who are only breastfed respectively. The probabilities used within this parameter are calculated from the Malawi DHS surveys in which breastfeeding status

across the first six-months is reported (11,12). Full details of this calculation are provided in Table S10.

For neonates who are breastfed, parameter **prob_early_breastfeeding_hb** or **prob_early_breastfeeding_hf** is used to determine if breastfeeding was initiated 'early', commonly defined as the initiation within the first hour of life (13), for infants born at home or in a health facility respectively. The Malawian DHS reports the proportion of women who initiated early breastfeeding following a homebirth and the same proportion following a facilities delivery which has been used to inform these parameters (11,12). Breastfeeding status is updated at six-months after birth, at which point exclusively breastfed infants have an equal probability of becoming non-exclusively fed or stopping entirely, and non-exclusively breastfed infants have an equal probability of remaining non-exclusively breastfed or stopping entirely. An event at two years post-birth occurs for all infants to reset status to 'none'. The effect of early initiation of breastfeeding on risk of neonatal sepsis is discussed under the corresponding heading in §3, with breastfeeding status also affecting several other outcomes for conditions external to the MPHM such as risk of acute lower respiratory infection and diarrhoea.

Parameter name	Description	Value*	Data source and/or relevant calculations
**prob_breastfeeding_type**	The probabilities that a neonate will not be breastfed, will be non-exclusively breastfed or will be exclusively breastfed	[0.005, 0.277, 0.718] / [0.101, 0.289, 0.61]	Values were calculated using data from the DHS surveys conducted in 2010 and 2015 which reports the 'Percent distribution of youngest children under age 2 who are living with their mother, by breastfeeding status and percentage currently breastfeeding (11,12). This data is disaggregated by age in months.<br><br>As the values here pertain to breastfeeding status in the first sixth months of life in the model, the average proportion of feeding status across the first sixth months of life was calculated.
**prob_early_breastfeeding_hb**	The probability that a breastfed neonate who was born at home will initiate breastfeeding within one hour of birth.	0.94 / 0.67	The 2010 and 2015 values are sourced directly from the DHS (11,12). There is no apparent change in survey methodology to account of observed difference between early initiation of breastfeeding between these two dates which suggests this observation is due to a change in breastfeeding practices in the population
**prob_early_breastfeeding_hf**	The probability that a breastfed neonate who was born in a health facility will initiate breastfeeding within one hour of birth.	0.94 / 0.77	See **prob_early_breastfeeding_hf.** The 2010 and 2015 values are sourced directly from the DHS (11,12).

** Where two values (or sets of values) are provided the first set is applied from 2010-2014 and the second set from 2015 onwards for a given simulation run (§1.2.1.1)*

Table S10 – Parameters determining the breastfeeding status of newborns

1.2.4 Postnatal and neonatal processes

1.2.4.1 Risk of maternal and neonatal postnatal complications

Following birth, the application of risk of maternal postnatal complications occurs during several time steps including immediately following delivery and then weekly for each of the remaining six weeks of the postnatal period. A similar structure is implemented for neonatal complications with initial risk calculated and applied on birth followed by weekly application of risk for the four weeks of the neonatal period. Risk application is represented diagrammatically in Figure S2 below.

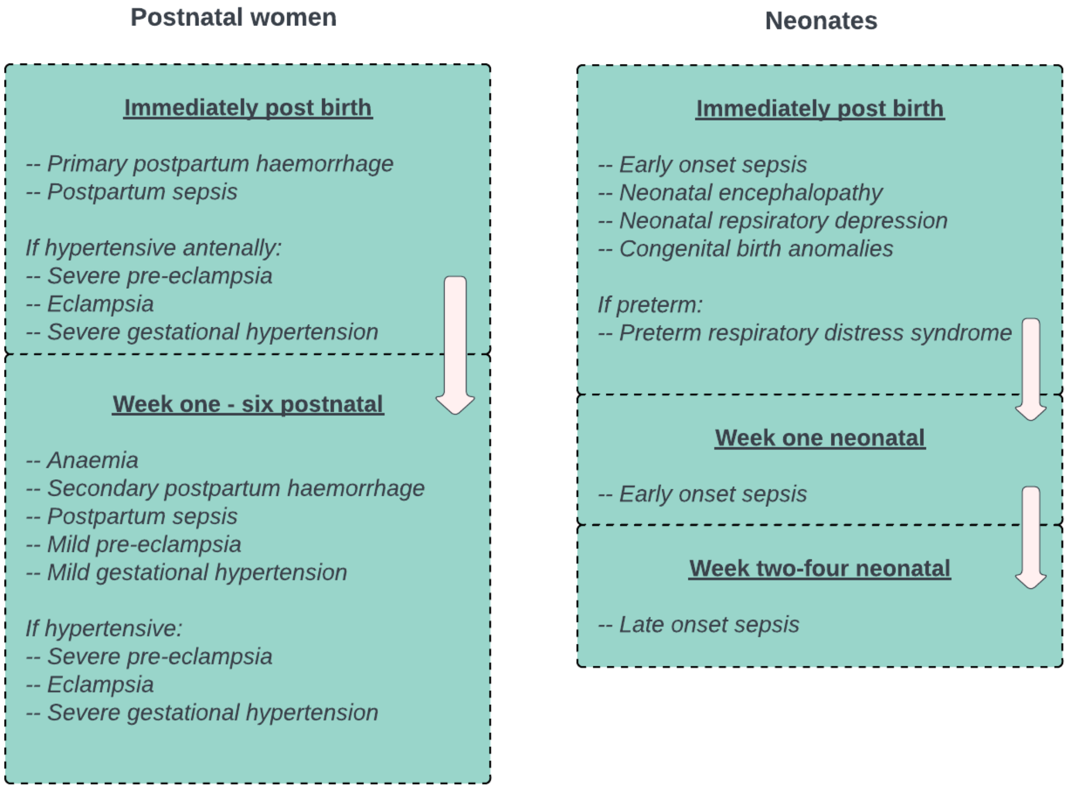

Figure S2 – Risk application during the postnatal and neonatal periods.
Time points during the postnatal period of pregnancy at which risk of modelled complications is applied. On the left, each box represents a new time step in which risk of acquisition is applied to postnatal mothers whilst on the right each box represents a new time step in which risk of acquisition is applied neonates.

1.2.5 Common processes

1.2.5.1 Obstetric history

At the initialisation of any simulation the parity and previous delivery status for all women of reproductive age within the population is predicted. Parity for these individuals is calculated using a linear regression model derived from the Malawi 2010 DHS dataset (12). The intercept value and coefficients for this model are presented in the parameter Table S11. The result from this regression model is calculated for individuals at initialisation of the simulation and rounded to the nearest integer representing their parity.

Currently, whilst parity at baseline is influenced by socio-demographic variables, the probability of pregnancy, as determined by the contraception module, is not. This is a limitation of the approach taken here. In addition, the parameters **prob_previous_caesarean_at_baseline** and **prob_previous_miscarriage_at_baseline** determines if women of reproductive age have previously delivered via CS and have previously experienced a miscarriage respectively.

Parameter name	Description	Value	Data source and/or relevant calculations
**intercept_parity_lr2010**	The intercept value for linear regression predicting individual parity at simulation baseline<br><br>The parameters below starting with "effect_" refer to the effect on parity at baseline	-2.81	A linear regression model was developed using the 2010 Malawian DHS data (12). The dependent variable was the number of children ever born as reported at the time of the survey and model coefficients were selected for inclusion in the model were based on evidence from contextually similar settings. The effect of these coefficients on predicted parity is shown in the following table rows. The output of the model is rounded to the nearest integer to give predicted parity.
**effect_age_parity_lr2010**	The effect of a unit increase in age from 15 years old	0.21	See **intercept_parity_lr2010**.
**effect_mar_stat_2_parity_lr2010**	The effect of being married compared to never married	0.84	See **intercept_parity_lr2010**.
**effect_mar_stat_3_parity_lr2010**	The effect of being previously married compared to never married	0.14	See **intercept_parity_lr2010**.
**effect_wealth_lev_4_parity_lr2010**	The effect of being in the fourth wealth quintile compared to the fifth	-0.09	See **intercept_parity_lr2010**. Wealth quintiles are defined within the DHS according to household assets with the first quintile being highest and fifth the lowest (11,12).
**effect_wealth_lev_3_parity_lr2010**	The effect of being in the third wealth quintile compared to the fifth	-0.18	See **intercept_parity_lr2010**.
**effect_wealth_lev_2_parity_lr2010**	The effect of being in the second wealth quintile compared to the fifth quintile o	-0.22	See **intercept_parity_lr2010**.

**effect_wealth_lev_1_parity_lr2010**	The effect of being in the first wealth quintile compared to the fifth	-0.48	See **intercept_parity_lr2010**.
**effect_edu_lev_2_parity_lr2010**	The effect of being in education level two (having received primary schooling) compared to no education	-0.33	See **intercept_parity_lr2010**.
**effect_edu_lev_3_parity_lr2010**	The effect of being in education level three (having received secondary schooling or greater) compared to no education	-1	See **intercept_parity_lr2010**.
**effect_rural_parity_lr2010**	The effect of living in a rural area compared to an urban area	0.19	See **intercept_parity_lr2010**.
**prob_previous_caesarean_at_baseline**	The probability that a woman of reproductive age (15-49 years) who has previously given birth at least once has previously delivered via CS at simulation baseline	0.046	The proportion of total births in the last five years which occurred via CS was sourced from the 2010 Malawian DHS (12) as 4.6%. Whilst this is likely lower than the proportion of all women of reproductive age who have delivered via CS in their lifetime this has been used as a proxy for the purpose of the model.

**prob_previous_miscarriage_at_baseline**	The probability that a woman of reproductive age has previously experienced a spontaneous abortion at simulation baseline	0.047	The 2015 Malawi DHS survey (11) reports the proportion of women who have experienced a miscarriage in the previous 5 years (4.7%) which has been used for this parameter. Whilst this is likely lower than the proportion of all women of reproductive age who have experienced a miscarriage in their lifetime this has been used as a proxy for the purpose of the model.

**All parameters in this table are applied only once at simulation intialisation*

Table S11 – Parameters determining baseline obstetric history

1.2.5.2 Maternal and neonatal death

For conditions in the model which have been deemed potentially fatal, a parameter represents the probability of death due to the condition in the absence of treatment. This probability of cause-specific maternal or neonatal death is applied to any individual experiencing the condition after it has been determined if they will receive any healthcare and such healthcare has been delivered. If treatment is received, this probability of death is multiplied by the relevant treatment effect as depicted in the model diagrams for relevant conditions in §3. For example, probability of maternal death following postnatal sepsis is calculated as:

y = **cfr_pp_sepsis** * (*la_sepsis_treatment* * **sepsis_treatment_effect_md**)

(3)

Where *y* is the cause-specific probability of death, *la_sepsis_treatment* is the variable signifying treatment has been delivered and **sepsis_treatment_effect_md** is the effect of the treatment on the probability of death without treatment, **cfr_pp_sepsis**. If the result of a random draw is lower than the probability of death, then the death occurs.

As the model allows for multiple conditions to occur to an individual it is possible an individual may be at risk of death from several conditions at the same time. First, the cause-specific probability of death is calculated for each relevant condition (accounting for treatment delivery where appropriate). Then total probability of death is then equal to:

P(death) = 1 – ((1- P(death_cause1)) * (1- P(death_cause2)) * …

(4)

When death occurs in the model the cause of death is logged and passed to the demography module. If a single condition has occurred then this is recorded as the cause of death, if multiple conditions have occurred in the same woman, then a probability weighted draw, accounting for probability of death for each relevant cause, determines the primary cause of death to be logged.

1.2.5.3 Maternal and neonatal morbidity

1.2.5.3.1 Maternal disability weights

To calculate the total DALYs attributable to health conditions within Malawi, disability weights from the 2019 GBD Study (14) are assigned to individuals in the population who experience health conditions within the TLO framework and are calculated across the population in a monthly timestep.

Table S12 contains the disability weights for maternal conditions in the MPHM. As described in the tables, it is assumed that for acute complications, such as obstructed labour, the entire value of the weight is applied to that individual during the month the complication occurs. For other conditions, which may persist across the length of pregnancy, the entire weight is assigned if the individual has experienced the condition for the entirety of the prior month, otherwise a fraction of the weight depending on the number of days in the previous month the complication has been experienced is returned.

Model Condition	Weight	GBD Sequelae
Complications where total weight is applied once within month of complication onset:		
Abortion (+/- complications)	0.114 (0.078-0.159)	Maternal abortive outcome
Abortion complicated by haemorrhage	0.114 (0.078-0.159)	Maternal haemorrhage (< 1L blood lost)
Abortion complicated by sepsis	0.133 (0.088-0.19)	Puerperal sepsis
Ectopic pregnancy	0.114 (0.078-0.159)	Ectopic Pregnancy
Ectopic pregnancy rupture†	0.114 (0.078-0.159)	Maternal haemorrhage (< 1L blood lost)
Mild or moderate antepartum haemorrhage	0.114 (0.078-0.159)	Maternal haemorrhage (< 1L blood lost)
Severe antepartum haemorrhage	0.324 (0.22 -0.442)	Maternal hemorrhage (> 1L blood lost)
Antenatal sepsis	0.133 (0.088-0.19)	Puerperal sepsis
Eclampsia†	0.263 (0.173-0.367)	Epilepsy, seizures 1-11 per year
Obstructed Labour	0.324 (0.22 -0.442)	Obstructed labour, acute event
Uterine Rupture†	0.324 (0.22 -0.442)	Maternal hemorrhage (> 1L blood lost)
Mild or moderate postpartum haemorrhage	0.114 (0.078 – 0.159)	Maternal hemorrhage (< 1L blood lost)
Severe postpartum haemorrhage	0.324 (0.22 -0.442)	Maternal hemorrhage (> 1L blood lost)
Secondary postpartum haemorrhage	0.114 (0.078 – 0.159)	Maternal hemorrhage (< 1L blood lost)

Conditions where a fraction of the weight is applied for every day the condition has occurred in the previous month:*		
Mild anaemia	0.004 (0.001 – 0.008)	Anemia, mild
Moderate anaemia	0.052 (0.034-0.076)	Anemia, moderate
Severe anaemia	0.149 (0.101 -0.209)	Anemia, severe
Hypertension	0.049 (0.031-0.072)	Other hypertensive disorders of pregnancy
Gestational diabetes†	0.049 (0.031-0.072	Uncomplicated diabetes mellitus
Vesicovaginal fistula	0.342 (0.227 – 0.478)	Vesicovaginal fistula
Rectovaginal fistula	0.501 (0.339 -0.657)	Rectovaginal fistula

† The 2019 GBD study did not include disability weights for these conditions, so we have used weights from conditions which were deemed most similar in terms of sequelae.
** Daily weight is calculated by dividing the total weight by 362.25.*

Table S12 – Disability weights for maternal conditions included in the model

1.2.5.3.2 Neonatal disability weights

As opposed to applying a disability weight to all neonates who experience a condition in the model, the probability of neurodevelopmental impairment is applied to any neonates who survive the neonatal period (i.e., the first 28 days of life) and have experienced one of the modelled conditions. This decision was made as the probabilities of impairment for each condition were sourced from studies evaluating impairment only in survivors of the first 28 days.

For example, all preterm neonates who survive to 28 days of life are at risk of developing mild motor impairment, mild motor and cognitive impairment, moderate motor impairment or severe motor impairment secondary to their prematurity. 'Type' of impairment is stored

as a categorical variable which signifies the relevant disability weight should be assigned to that neonate for a given month. Currently it is assumed that impairment is life-long meaning the disability weight associated with a specific level of impairment remains with the newborn for the remainder of their life in the simulation contributing to DALYs.

Table S13 contains disability weights for neonatal conditions in the model alongside the probability that a specific type of impairment will occur if the preceding condition has been experienced by that neonate. Table S14 contains details on parameters representing said probabilities.

**Model condition causing impairment**	**GBD Sequalae**	**Parameter representing probability of impairment following condition** *(Probability)*	**Disability weight**
Prematurity (<32 weeks GA)	"Mild motor impairment due to neonatal preterm birth complications 28-32wks"	**prob_mild_disability_preterm_<32weeks*** *(0.324)*	0.01 (0.005, 0.01)
	"Mild motor plus cognitive impairments due to neonatal preterm birth complications 28-32wks"	**prob_mild_disability_preterm_<32weeks*** *(0.324)*	0.031 (0.018, 0.05)
	"Moderate motor impairment due to neonatal preterm birth complications 28-32wks"	**prob_mod_severe_disability_preterm _<32weeks*** *(0.246)*	0.06 (0.04, 0.089)
	"Severe motor impairment due to neonatal preterm birth complications 28-32wks"	**prob_mod_severe_disability_preterm _<32weeks*** *(0.246)*	0.4029 (0.268, 0.545)
Prematurity (32-36 weeks GA) *Prematurity*	"Mild motor impairment due to neonatal preterm birth complications 32-36wks"	**prob_mild_disability_preterm_32_36weeks*** *(0.034))*	0.01 (0.005, 0.01)
	"Mild motor plus cognitive impairments due to neonatal preterm birth complications 32-36wks"	**prob_mild_disability_preterm_32_36weeks*** *(0.034))*	0.031 (0.018, 0.05)
	"Moderate motor impairment due to neonatal preterm birth complications 32-36wks"	**prob_mod_severe_disability_preterm_32_36weeks*** *(0.018))*	0.06 (0.04, 0.089)
	"Severe motor impairment due to neonatal preterm birth complications 32-36wks"	**prob_mod_severe_disability_preterm_32_36weeks*** *(0.018))*	0.4029 (0.268, 0.545)

Retinopathy of prematurity	"Mild vision impairment due to retinopathy of prematurity"	**prob_retinopathy_severity_no_treatment** *(0.03)*	0.003 (0.001, 0.007)
	"Moderate vision impairment due to retinopathy of prematurity"	**prob_retinopathy_severity_no_treatment** *(0.1)*	0.031 (0.019, 0.049)
	"Severe vision impairment due to retinopathy of prematurity"	**prob_retinopathy_severity_no_treatment** *(0.12)*	0.184 (0.125, 0.258)
	"Blindness due to retinopathy of prematurity"	**prob_retinopathy_severity_no_treatment** *(0.47)*	0.187 (0.124, 0.26)
Neonatal encephalopathy	"Mild motor impairment due to neonatal encephalopathy due to birth asphyxia and trauma"	**prob_mild_impairment_post_enceph*** *(0.21)*	0.01 (0.005, 0.019)
	"Mild motor plus cognitive impairments due to neonatal encephalopathy due to birth asphyxia and trauma"	**prob_mild_impairment_post_enceph*** *(0.21)*	0.031 (0.018, 0.05)
	"Moderate motor impairment due to neonatal encephalopathy due to birth asphyxia and trauma"	**prob_mod_severe_impairment_post_enceph*** *(0.269)*	0.061 (0.04, 0.089)
	"Severe motor impairment due to neonatal encephalopathy due to birth asphyxia and trauma"	**prob_mod_severe_impairment_post_enceph*** *(0.269)*	0.402 (0.268, 0.545)

Neonatal sepsis	"Mild motor impairment due to neonatal sepsis and other neonatal infections"	**prob_mild_impairment_post_sepsis*** *(0.12)*	0.01 (0.005, 0.019)
	"Mild motor plus cognitive impairments due to neonatal sepsis and other neonatal infections	**prob_mild_impairment_post_sepsis*** *(0.12)*	0.031 (0.018, 0.05)
	"Moderate motor impairment due to neonatal sepsis and other neonatal infections"	**prob_mod_severe_impairment_post_sepsis*** *(0.23)*	0.061 (0.04, 0.089)
	"Severe motor impairment due to neonatal sepsis and other neonatal infections"	**prob_mod_severe_impairment_post_sepsis*** *(0.23)*	0.402 (0.268, 0.545)

*(*See Table S13 for detailed description on these parameters – source data does not differentiate between mild motor impairment +/- cognitive impairment or between moderate and severe impairment. As such, a probability of any mild or moderate/severe impairment is applied and random draw determines final weight.)*

Table S13 – Disability weights for neonatal conditions included in the model

Parameter name	Description	Value	Data source and/or relevant calculations
**prob_mild_disability_preterm_<32weeks**	The probability that a preterm neonate born before 32 weeks GA who survives the first 28 days of life will experience mild impairment	0.324	We were unable to identify a data source from Malawi which reported disability status in preterm infants which corresponded to the appropriate disability weights. Therefore, this value is sourced directly from Blencowe et al (15) who estimate impairment in this population through a systematic review and meta-analysis of relevant studies.
**prob_mod_severe_disability_preterm_<32weeks**	The probability that a preterm neonate born before 32 weeks GA who survives the first 28 days of life will experience moderate or severe impairment	0.246	See **prob_mild_disability_preterm_<32weeks.**
**Ppob_mild_disability_preterm_32_36weeks**	The probability that a preterm neonate born between 32- and 36-weeks GA who survives the first 28 days of life will experience mild impairment	0.034	See **prob_mild_disability_preterm_<32weeks.**
**prob_mod_severe_disability_preterm_32_36weeks**	The probability that a preterm neonate born between 32- and 36-weeks GA who survives the first 28 days of life will experience moderate or severe impairment	0.018	See **prob_mild_disability_preterm_<32weeks.**

**prob_retinopathy_severity_no_treatment**	A list of probabilities used in a probability weighted random draw determining if neonate who is experiencing retinopathy of prematurity will experience no lifelong visual impairment or impairment that is mild, moderate, severe or blindness	[0.28, 0.03, 0.1, 0.12, 0.47]	We were unable to identify a data source from Malawi which reported the prevalence of disability due to retinopathy of prematurity (ROP). As such these values are sourced directly from Blencowe et al. (16) in which the authors estimate the proportion of neonates with ROP who experience visual impairment through a systematic review and meta-analysis of published estimates.
**prob_mild_impairment_post_enceph**	The probability that a neonate with neonatal encephalopathy who survives the first 28 days of life will experience life-long mild motor impairment	0.21	We were unable to identify a data source from Malawi which reported the prevalence of disability due to neonatal encephalopathy. As such these values are sourced directly from Lee et al. (17) who estimate impairment in this population through a systematic review and meta-analysis of relevant studies.
**prob_mod_severe_impairment_post_enceph**	The probability that a neonate with neonatal encephalopathy who survives the first 28 days of life will experience life-long moderate or severe motor impairment	0.269	See **prob_mild_impairment_post_enceph.**
**prob_mild_impairment_post_sepsis**	The probability that a neonate with sepsis who survives the first 28 days of life will experience life-long mild impairment	0.12	We were unable to identify a data source from Malawi which reported the prevalence of disability due to neonatal sepsis. As such these values are sourced directly from Seale et al. (18) who estimated the proportion of survivors of neonatal meningitis who experience either mild or moderate/severe neurodevelopmental impairment via a systematic review and meta-analysis. Due to lacking data the authors could not estimate impairment for neonatal sepsis therefore the meningitis estimates are used here as a proxy.

**prob_mod_severe_impairment_post_sepsis**	The probability that a neonate with sepsis who survives the first 28 days of life will experience life-long moderate or severe impairment	0.23	See **prob_mild_impairment_post_sepsis.**

Table S14 – Parameters representing the probability of impairment in neonates who experience modelled conditions

2 – Healthcare modelling

In this section the representation of healthcare within the MPHM is described by first providing an overview of which services are modelled, followed by a detailed description of how care seeking, and intervention delivery are structured. Whilst all interventions are introduced in this section, we have opted to provide further detail on how interventions impact specific health conditions within the relevant descriptions in §3.

2.1 Overview of modelled healthcare

Figure 1 in the accompanying manuscript, the diagrammatic representation of the MPHM, shows the main interactions between an individual and the healthcare system included in the model. As with the epidemiological and obstetric processes described thus far, the modelling of maternal and perinatal healthcare has been designed to represent maternity service delivery in Malawi. Therefore, the model includes antenatal, intrapartum, and postpartum care in addition to treatment for women facing complications associated with abortion or ectopic pregnancy.

In Figures S3 and S4 below the healthcare available to mothers and newborns within the MPHM is summarised. Within the TLO model healthcare is delivered to individuals via discrete Health System Interactions (HSIs), which have two key properties; the facility level at which the event can occur and the amount of healthcare worker (HCW) time required to deliver this instance of care.

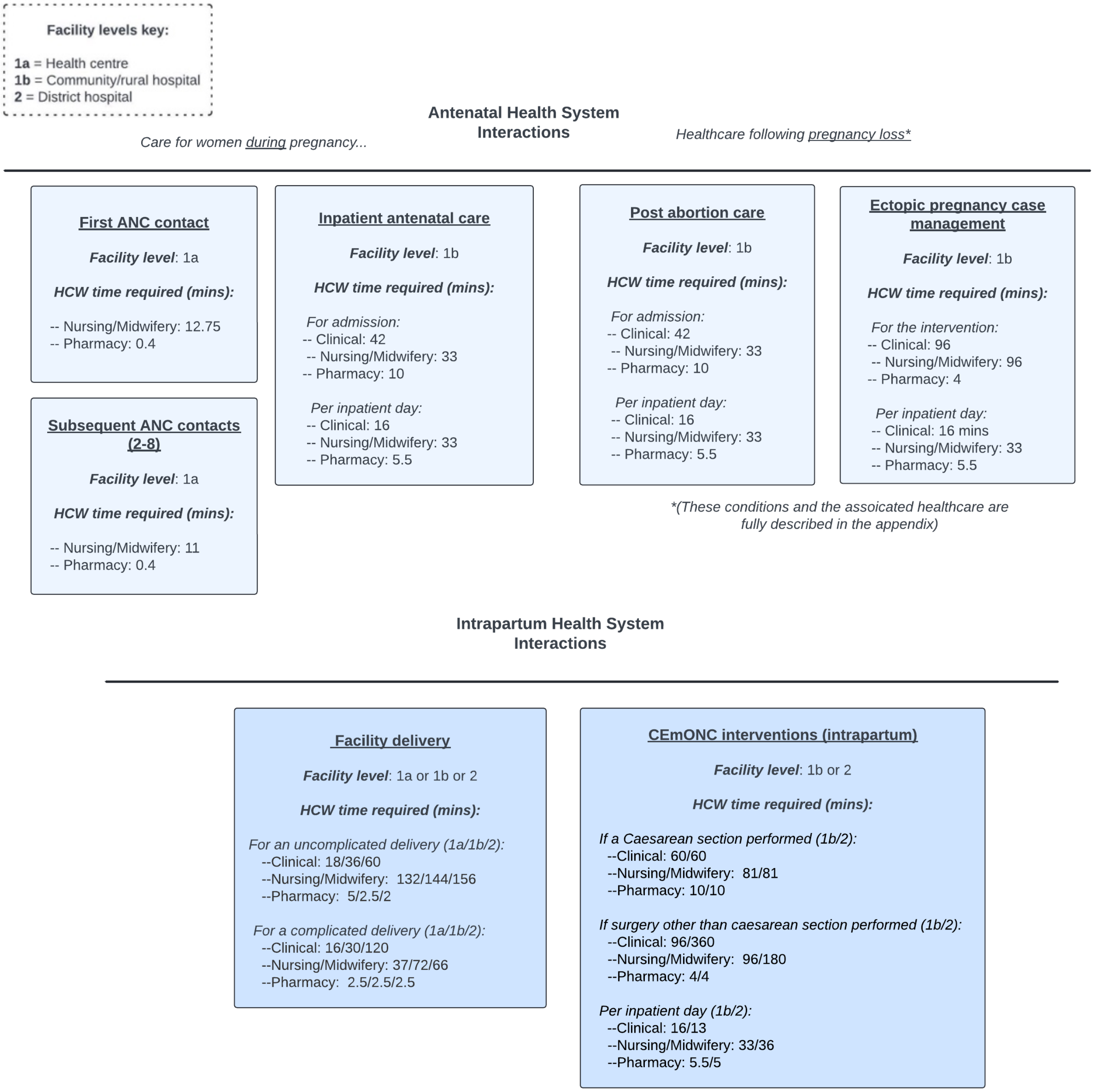

Figure S3 – Summary of modelled antenatal and intrapartum health system interactions
Here HSIs representing healthcare delivered to mothers in the MPHM are shown as boxes. Care delivered during the antenatal and intrapartum phase is separated. For each HSI the possible facility level at which the event can occur is provided in addition to the required HCW time, in minutes, by cadre.

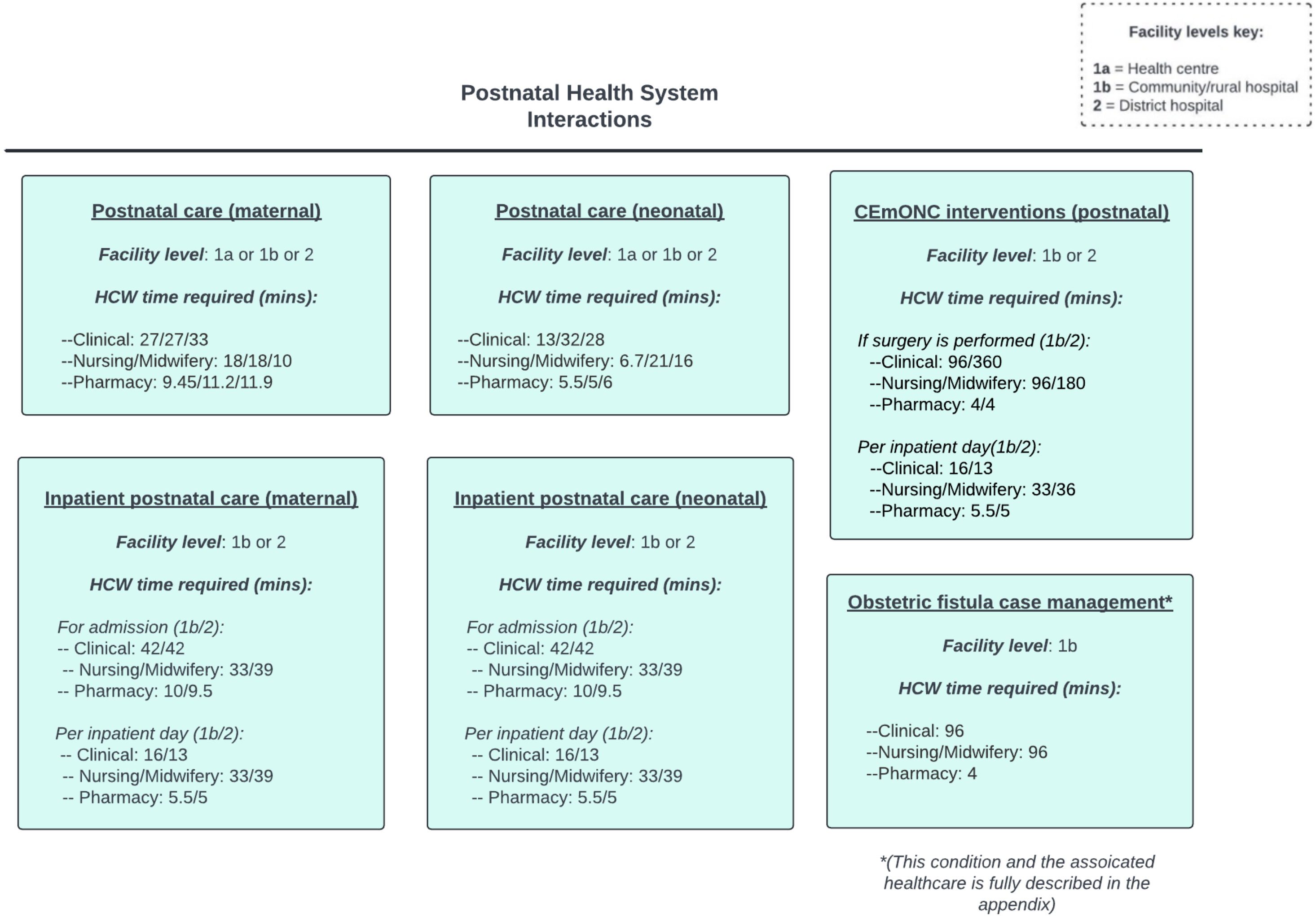

Figure S4 – Summary of the modelled postnatal health system interactions.
Here HSIs representing healthcare delivered to mothers and newborns in the MPHM during the postnatal period are shown as boxes. For each HSI the possible facility level at which the event can occur is provided in addition to the required HCW time, in minutes, by cadre.

Of note, the HCW time requirements disaggregated by cadre and facility level presented in Figures S3 and S4 are defined by the healthcare system model and have been sourced directly from the Malawi Human Resources for Health Strategic Plan 2018-2022 (19). As such these values are not parameters of the MPHM and have been defined externally. Reasons for observed variation in HCW time requirements by facility level is currently not recorded in the data set but is assumed to be a product of the greater acuity of cases clustered at higher facility levels. For example, for the HSI ***'facility delivery'*** the HCW time requirement appears to increase with facility level suggesting that more time intensive deliveries are concentrated at higher facility levels.

Table S15 lists all the modelled interventions included within the MPHM, by HSI, including how EmONC signal functions are represented. Additional detail (consumables required, effect on outcomes) of each intervention is provided throughout this section.

Health system interaction†	Modelled interventions
**Antenatal care (Contacts 1-8)**	*Screening interventions:*<br>● Blood pressure measurement<br>● Urine dipstick<br>● Point-of-care haemoglobin testing<br>● Syphilis testing (rapid plasma reagin)<br>● HIV testing±<br>● Gestational diabetes testing (blood glucose)<br>● Screening for depression±<br>● Screening for tuberculosis±<br><br>*Preventative interventions:*<br>● Daily iron and folic acid supplementation<br>● Calcium supplementation*<br>● Balanced energy and protein supplementation<br>● Antibiotic treatment for syphilis<br>● Albendazole (deworming)<br>● Intermittent preventive treatment of malaria during pregnancy (IPTp)<br>● Insecticide treated bed nets±<br>● Tetanus toxoid immunisation±
**Inpatient antenatal services**	*Preventative interventions:*<br>● Antibiotics prophylaxis following PROM<br>● Referral for emergency delivery<br><br>*Curative interventions:*<br>● Gestational diabetes case management (diet/exercise, oral diabetic medications, insulin)<br>● Blood transfusion<br>● Antihypertensive treatment (oral and intravenous)<br>● Magnesium sulphate
**Facility delivery (BEmONC)**	*Preventative interventions:*<br>● Clean birth practices<br>● Antibiotics prophylaxis following PROM<br>● Antenatal corticosteroids following preterm labour<br><br>*Curative interventions:*<br>● Maternal sepsis case management (intravenous antibiotics)<br>● Antihypertensive treatment (intravenous)<br>● Magnesium sulphate<br>● Assisted vaginal delivery<br>● Active management of the third stage of labour<br>● Neonatal stimulation and resuscitation

**Facility delivery (CEmONC)**	*Curative interventions:*<br>● Delivery via caesarean section<br>● Additional curative surgery (uterine rupture repair/hysterectomy)<br>● Blood transfusion
**Postnatal care (maternal)**	*Screening interventions:*<br>● HIV testing±<br>● Screening for depression±<br><br>*Preventative interventions:*<br>● Daily iron and folic acid supplementation<br><br>*Curative interventions:*<br>● Postpartum haemorrhage case management (uterotonics, manual removal of retained placenta, referral for surgery)<br>● Maternal sepsis case management (intravenous antibiotics)<br>● Antihypertensive treatment (intravenous)<br>● Magnesium sulphate
**Postnatal care (neonatal)**	*Screening interventions:*<br>● HIV testing±<br><br>*Preventative interventions:*<br>● Other essential newborn care – vitamin K prophylaxis, eye care, immunisations<br><br>*Curative interventions:*<br>● Neonatal sepsis case management – injectable antibiotics<br>● Neonatal sepsis case management – full supportive care<br>● Kangaroo mother care
**CEmONC – Postnatal**	*Curative interventions:*<br>● Surgery (management of postpartum haemorrhage)<br>● Blood transfusion
±The effect of this intervention on outcomes is determined by the relevant disease model and is therefore not described within the supplementary material of this paper.<br>*Calcium supplementation is only delivered to women deemed to be at risk of developing pre-eclampsia in line with Malawian guidelines<br>† A health system interaction (HSI) is an event in the simulation in which healthcare is delivered. Interventions are housed within HSIs defined by the level of facility at which they occur and the required healthcare worker time.	

Table S15 – Interventions included in the model

2.2 Modelling the 'Three Delays'

Thaddeus and Maine's (20) seminal paper examining factors contributing to maternal mortality in low-and-middle income settings (LMIC) contexts identified three 'delays' which exacerbate poor outcomes for women following obstetric complications; delay in deciding to seek care, delay in reaching care and delay in receiving treatment within a facility. We have sought to explicitly model these delays within the framework as evidence suggests that all three delays have been reported as an important predictive factor for poor maternal outcomes within Malawi (21,22).

2.2.1 Delays one and two

In the model, it is assumed that individuals who seek care for the following reasons may be delayed in reaching a health facility (i.e., either delay one or delay two), which may reasonably affect the outcome of their treatment:

1.) Following complications of pregnancy loss (i.e., abortion or ectopic pregnancy)
2.) Following emergency obstetric complications in the antenatal period
3.) Following the onset of labour
4.) Following emergency obstetric complications in the postnatal period
5.) Following emergency neonatal complications in the neonatal period

Emergency complications, in this context, refer to any which are modelled as being potentially fatal and are assumed to trigger possible care seeking as described in the respective descriptions in §3. Care seeking for routine antenatal and postnatal care is not assumed to be affected by this type of delay.

For simplicity and due to limited available data, a single parameter represents the probability of a delay in reaching a health facility, **prob_delay_one_two_fd** (Table S15). This parameter is representative of both the first and second delay as travel from home to facility is not explicitly modelled in the framework.

Parameter Name	Description	Value	Data source and/or relevant calculations
**prob_delay_one_two_fd**	The probability that an individual seeking emergency care in the antenatal or postnatal period or seeking care following the onset of labour will experience a delay in attending a facility for treatment	0.363	We were unable to find a study from Malawi estimating the proportion of women who are delayed in seeking obstetric care. This value is taken from Tiruneh et al. (23) who conducted an institutional-based cross sectional study reporting the proportion of postpartum women who were delayed in deciding to seek emergency obstetric care for institutional delivery in a study conducted in Ethiopia.

Table S16 – Parameter representing delay in healthcare seeking

2.2.2 Delay three

Additionally, Thaddeus and Maine's third delay (20) is also modelled in which delivery of care is delayed within a health facility. Mechanistically this logic utilises a parameter of the TLO health system model called the squeeze factor (SF). The SF is calculated for each HSI event that is executed within a simulation run using the fractional over-demand among the HCW cadres who are required to deliver said event, as stored in the appointment footprint. The SF is therefore calculated as the required HCW time divided by available time, for a given day in the simulation, minus one. This leads to a SF of less than 0 when required time is less than available time, an SF of 0 when required time is equal to available time, and an SF of greater than 0 when required time is greater than available time. As opposed to using the SF to determine if the event will or will not run, it is assumed that when the SF exceeds a pre- specified threshold that intervention delivery is delayed as shown in Table S16.

Parameter Name	Description	Value*	Data source and/or relevant calculations
squeeze_threshold_for_delay_three_an	The squeeze factor value over which an individual receiving treatment during antenatal inpatient care will experience delayed care	3	To determine this value the model was run over one year and the squeeze factor extracted for each of the relevant HSIs. The median value across the squeeze factors was determined in the model's current state and was approximately 3 across the events. This value was selected for the parameter meaning approximately 50% of women receiving care would experience a delay. Whilst we were unable to estimate the proportion of women who experience delay three in Malawi evidence supports that in many contextually similar settings, delay three is high (22,24–26).
squeeze_threshold_for_delay_three_bemonc	The squeeze factor value over which an individual receiving initial intrapartum care will experience delayed care		
squeeze_threshold_for_delay_three_cemonc	The squeeze factor value over which an individual receiving comprehensive intrapartum or postpartum care will experience delayed care		
squeeze_threshold_for_delay_three_pn	The squeeze factor value over which an individual receiving emergency postnatal care will experience delayed care		

The same value is used for all parameters listed here

Table S17 – Parameters representing 'squeeze factor thresholds' for the modelled health system interactions

2.2.3 Applying the effect of the three delays

Whilst there is evidence from Malawi which attributes delays in care to increased likelihood of poor maternal outcomes including death (22,24–26), we were unable to identify any studies from the county which quantified this relationship. Additionally, whilst several recent facility-based studies conducted in Ethiopia report the effect of various delays to healthcare on severe maternal outcomes there is significant variation on the possible size of effect and how each delay is defined (27–30). Because we feel representation of this effect is Important, we have opted to include the effect of delays on treatment effectiveness within the model by assuming that experiencing either delay 1, 2 or 3 will reduce the effect by 25%, and experiencing all delays will reduce the effect by 50%. This effect applied as:

$$AdjustedTE = 1 - \big((1 - TE) * TE\_modifier\big) \tag{5}$$

Where *AdjustedTE* is the treatment effect after adjustment for delays in care, *TE* is the unadjusted treatment effect and *TE_modifier* is the effect of delay on the treatment effect. In Table S17 **treatment_effect_modifier_one_delay** and **treatment_effect_modifier_all_delays** are the effect of experiencing one or multiple delays on the treatment effect respectively.

Parameter Name	Description	Value	Data source and/or relevant calculations
**treatment_effect_modifier_one_delay**	The effect of a mother having experienced delay in receiving care on the effectiveness of treatment for a given condition	0.75	This parameter value is an assumption. See §2.2.3 for how this effect is applied to reduce the total effectiveness of a treatment by 25%.
**treatment_effect_modifier_all_delays**	The effect of a mother having experienced all three delays in receiving care on the effectiveness of treatment for a given condition	0.5	This parameter value is an assumption. See §2.2.3 for how this effect is applied to reduce the total effectiveness of a treatment by 50%.

Table S18 – Parameters representing the effect of delayed care on treatment effectiveness

2.3 Modelling the Emergency Obstetric and Newborn Care signal functions

In Malawi, EmONC interventions are available to mothers and neonates as part of the case management of common conditions (e.g., uterotonic drug administration is one part of the treatment cascade for postpartum haemorrhage according to Malawian guidelines (31,32). In recent years, two national facility-based assessments of the availability and quality of EmONC signal functions have been conducted in Malawi (33,34) which have been used to develop the model related to the availability of these interventions to mothers and newborns.

2.3.1 Quality of EmONC interventions

For interventions in the model which are classified as Emergency Obstetric and Newborn Care (EmONC) signal functions, replication of the assumed quality of care experienced by mothers in Malawi is achieved by conditioning intervention delivery on the result a random draw against the output of the following equation:

$$P(Intervention) = P(\boldsymbol{hcw\_availabile})_{(Int)} * \boldsymbol{mean\_hcw\_competence}_{(FT)} * \prod c_{i(t)} \tag{6}$$

Here *P(Intervention)* is the probability of intervention delivery and P(***hcw_available***) is the probability that a HCW trained in delivery of the Basic/Comprehensive EmONC intervention *(int)*, is available to deliver the intervention. These intervention specific probabilities were extracted from national survey data in Malawi capturing the availability of B/CEmONC services (33) as described further in Table S18. Currently there is no modelled relationship between HCW time requirements for the delivery of a given HSI and P(***hcw_available***)*(int)*, as the latter is an average probability that a HCW trained to deliver a given EmONC intervention is available in a facility.

Next ***mean_hcw_competence***$_{(FT)}$ is the probability that a HCW at facility type, identifies the need for an intervention to be delivered, parameters **mean_hcw_competence_hc** and **mean_hcw_competence_hp** are the probabilities for either a health centre or hospital**.** Finally, $\prod c_{i(t)}$ is the probability that all required consumables for intervention delivery are available at the facility level of interest for a given time, where $c_i(t)$ represents the availability of the ith consumable c at the facility level for time t. Consumables deemed both

essential and optional for the delivery of an intervention were identified using Malawian guidelines and are listed in the following section. Only the availability of essential consumables affects the probability of intervention delivery.

Parameter Name	Description	Value	Source and/or relevant calculations
**prob_hcw_avail_iv_abx**	The probability that a HCW trained to deliver intravenous antibiotic therapy will be available within a given HSI	0.99	The value for this parameter was taken directly from the Malawian BEmONC survey 2015 (33). The authors have estimated the average percentage availability of HCWs who were trained to deliver this intervention. This percentage has been used as a probability that HCWs are available to deliver the given intervention on the day care is delivered.
**prob_hcw_avail_anticonvulsant**	The probability that a HCW trained to deliver anticonvulsant therapy will be available within a given HSI	0.93	*See* **prob_hcw_avail_iv_abx.**
**prob_hcw_avail_retained_prod**	The probability that a HCW trained to undertake removal of retained products of conception be available within a given HSI	0.49	*See* **prob_hcw_avail_iv_abx.**
**prob_hcw_avail_avd**	The probability that a HCW trained to undertake AVD will be available within a given HSI	0.46	*See* **prob_hcw_avail_iv_abx.**
**prob_hcw_avail_uterotonic**	The probability that a HCW trained to deliver uterotonic therapy will be available within a given HSI	0.99	*See* **prob_hcw_avail_iv_abx.**
**prob_hcw_avail_man_r_placenta**	The probability that a HCW trained to deliver manual removal of retained placenta will be available within a given HSI	0.82	*See* **prob_hcw_avail_iv_abx.**

Parameter Name	Description	Value	Source and/or relevant calculations
**prob_hcw_avail_neo_resus**	The probability that a HCW trained to deliver neonatal resuscitation will be available within a given HSI	0.98	*See* **prob_hcw_avail_iv_abx.**
**prob_hcw_avail_surg**	The probability that a HCW trained to undertake obstetric surgery be available within a given HSI	0.74	*See* **prob_hcw_avail_iv_abx.**
**prob_hcw_avail_blood_tran**	The probability that a HCW trained to deliver blood transfusion therapy will be available within a given HSI	0.86	*See* **prob_hcw_avail_iv_abx.**
**mean_hcw_competence_hc**	The probability that a HCW providing care for a woman in a health centre will correctly identify the need for treatment of a given condition.	0.602	This value is taken from a study by Arsenault et al. (35) in which the authors analysed data from direct observation of 474 deliveries in Malawi. The authors report the mean score across several indicators relating to quality of care which has been used here as a proxy for overall intervention quality.
**mean_hcw_competence_hp**	The probability that a HCW providing care for a woman in a hospital will correctly identify the need for treatment of a given condition	0.662	*See* **mean_hcw_competence_hc.**

Table S19 – Parameters related to quality of B/CEmONC interventions delivered during inpatient care

In the following sections we provide a detailed overview of how antenatal, intrapartum, and postpartum maternity services are represented in the model including care seeking and intervention characteristics (e.g., effects and required consumables).

2.4 Antenatal healthcare

In the MPHM, healthcare in the antenatal period consists of treatment of complications associated with pregnancy loss (e.g., post abortion care), routine ANC and inpatient care for pregnant women experiencing antenatal emergencies.

2.4.1 Post abortion care

Risk of pregnancy loss secondary to induced or spontaneous abortion is applied monthly to all pregnant women as shown in Figure S1 and described further in §4. Any women who experience complications following an induced or spontaneous abortion may seek healthcare, with the parameter **prob_seek_care_pregnancy_loss** (Table S19) representing the probability that post abortion care (PAC) will be sought prior to risk of death being applied. The value is taken from a study conducted by Chinkhumba et al. (36) investigating the effect of a result-based financing policy of financial and time costs associated with obstetric emergencies in Malawi. The authors report the proportion of women experiencing obstetric complications who sought care during the pre-intervention baseline which has been used here to represent probability of care seeking.

**Parameter Name**	**Description**	**Value**	**Source and/or relevant calculation**
**prob_seek_care_pregnancy_loss**	The probability that a woman experiencing complications following a spontaneous or induced abortion or ruptured ectopic pregnancy will seek emergency care.	0.782	Sourced directly from Chinkhumba et al. (36)

Table S20 – Parameter for care seeking following abortion.

The recommended treatment for complications of abortion in Malawi is defined in the most recent clinical guidelines from which the treatment cascade applied within the model and

the required consumables have been sourced (32). Consumables used to treat post-abortion complications vary according by presenting complications as detailed in Table S20 below. Importantly, it is assumed that treatment is equally effective in reducing risk of death in all cases of complicated abortion regardless of 'type' of abortion complication present at time of treatment.

Healthcare interaction	Required and optional consumables	Modelled effect and source
Post abortion care (PAC)	***For all PAC cases…***<br>*Required:* Misoprostol 200mcg<br><br>*Optional:* Complete blood count, Blood collecting tube, Disposables gloves, Paracetamol, Pethidine, 50 mg/ml, 2 ml ampoule	If delivered, PAC reduces the risk of death associated with complications of induced or spontaneous abortion (RR 0.2 (37)).
	In addition, for septic PAC cases…<br>*Required:* Benzylpenicillin 3g (5 MU), Gentamycin 40 mg/ml in 2 ml vial<br><br>*Optional:* Sodium chloride 0.9% 500 ml, intravenous (IV) cannula, Disposables gloves, IV giving set, Oxygen	
	In addition, for haemorrhagic PAC cases…<br>*Required* Blood, one unit (x2)<br><br>*Optional:* IV cannula, Disposables gloves, IV giving set	
	In addition, for PAC cases complicated by injury…<br>*Required:* Sodium chloride 0.9% 500 ml, Oxygen<br><br>*Optional:* IV cannula, Disposables gloves, IV giving set	

Table S21 – Details of Post Abortion Care (PAC) health system interaction within the MPHM

2.4.2 Ectopic pregnancy case management

Similarly, to PAC, ectopic pregnancy case management in Malawi is defined in the most recent guidelines from which the treatment cascade in the model and the required consumables have been sourced (32). Women with an ectopic pregnancy may seek care prior to or post rupture of the fallopian tube, which alters the treatment effect as shown in Table S21 below. Treatment is described in further detail in §3.

**Treatment**	**Logged consumables**	**Modelled effect and source**
Ectopic pregnancy case management	*Required:* Halothane (fluothane),<br><br>*Optional:* Scalpel blade, Sodium chloride 0.9% 500 ml, Paracetamol, Pethidine 50 mg/ml 2 ml ampoule, Suture pack, Gauze, IV cannula, IV giving set, Disposables gloves	If delivered pre-rupture, treatment prevents rupture and averts the application of the risk of death.<br><br>If delivered post-rupture, then treatment reduces risk of death secondary to ectopic pregnancy (relative risk (RR) 0.1 (37)).

Table S22 – *Details of ectopic pregnancy case management health system interaction within the MPHM*

2.4.3 Routine antenatal care

Modelling of routine ANC within the MPHM was developed to replicate current service delivery in Malawi as outlined in recent guidelines documents (38,39). Therefore, in the model, an eight-contact ANC schedule was developed allowing women to receive routine care at the recommended GA in-line with these guidelines. The recommended scheduling for ANC in Malawi is shown below in Table S22.

ANC contact	Recommended GA at attendance
1	*Up to 12 weeks*
2	*20 weeks*
3	*26 weeks*
4	*30 weeks*
5	*34 weeks*
6	*36 weeks*
7	*38 weeks*
8	*40 weeks*

Table S23 – WHO 2016 ANC schedule by recommended gestational age at attendance

2.4.3.1 Care seeking

Care seeking for ANC has been designed to allow for calibration to recent estimates of coverage of ANC and timing of first ANC appointment by GA. Figure S5 provides a detailed overview of how ANC care seeking is simulated within the model with all relevant parameters depicted described in Table S23.

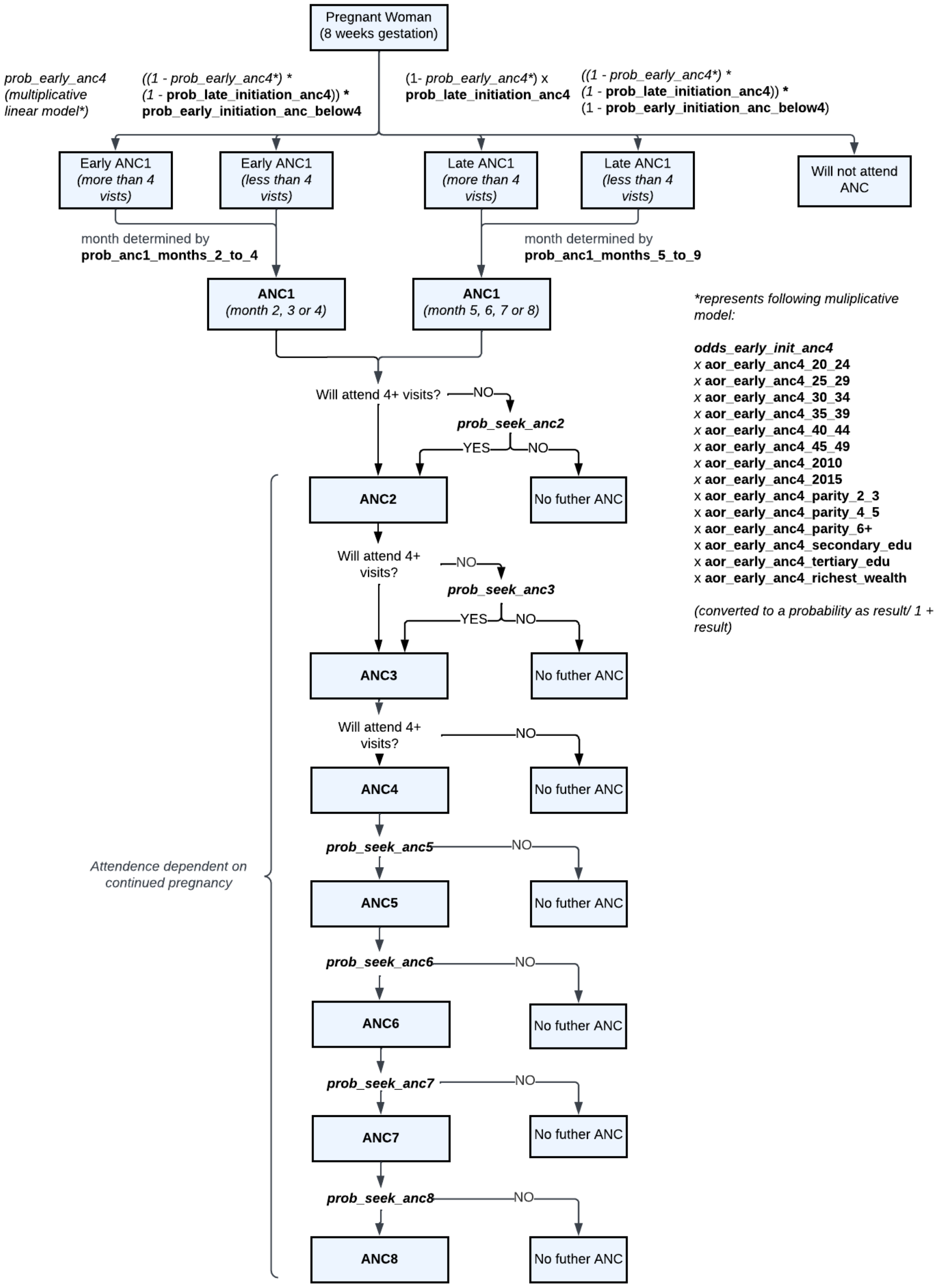

Figure S5 – Care seeking for routine ANC within the model

Scheduling of ANC HSIs within the model is represented in this figure. Relevant model parameters representing probability of receiving ANC and at what time point are defined.

As shown in Figure S5, at 8 weeks GA women are categorised into five groups: (i) will attend four or more ANC contacts with the first visit before month five of pregnancy, (ii) will attend four or more ANC contacts with the first visit after month five of pregnancy, (iii) will attend less than four ANC contacts with the first visits before five months gestation, (iv) will attend less than four ANC contacts with the first visits after five months gestation and (v) will not attend ANC.

As evident from these groupings, we have opted to focus on replicating the coverage of four or more ANC visits (ANC4+), as reported in the most recent DHS surveys conducted in Malawi (11,12) within the model. This choice was made because following implementation of the 2016 WHO ANC guidelines there have been no population level surveys capturing ANC attendance in Malawi, and whilst ANC attendance is captured in the DHIS2 system there are significant issues with data completeness. ANC4+ coverage in these surveys therefore represents the proportion of women who attended the recommended number of visits under the current policy recommendation during their previous pregnancy at the time of survey collection. Importantly, contemporary global maternal and newborn strategies, including the Ending Preventable Maternal Mortality and Every Newborn Action Plan targets focus on coverage targets for coverage of ANC4+.

The probability of categorisation into group (I) Is calculated from a logistic regression model developed using data from the Malawi DHS datasets between 2004-2016, by colleagues working on the TLO project including me, to explore the effect of sociodemographic variables on ANC4+ attendance (40). Ng'ambi et al. (40) use a composite binary dependent variable representing women who have attended ANC4+ in their last pregnancy with the first visit being before the fifth month. The dependent variable is considered as a proxy for 'perfect' ANC attendance as at the time of data collection as all women were recommended to attend at least four visits, with the first visit occurring between 12 and 17 weeks in keeping with Focused ANC guidelines in Malawi (40,41). The parameters for this regression model are shown in Table S23 below.

As demonstrated in Figure S5, the results from the logistic regression model, converted to a probability, and the parameters **prob_late_initiation_anc4** and **prob_early_initiation_anc_below4** (Table S23) are used to further categorise individuals. Dependent on categorisation, the first ANC contact is scheduled to occur on a random date within a selected month of pregnancy which is determined using probability weighted random draws with values from parameter **prob_anc1_months_2_to_4** and **prob_anc1_months_5_to_9**.

Before the interventions within an ANC contact can be delivered, the SF of the HSI is calculated and used to determine if the squeeze for a given ANC contact is too high for the event to run and if the event should be rescheduled for the next day. If the SF is too high for seven consecutive events the ANC contact never runs. The SF threshold, **squeeze_factor_threshold_anc,** is shown below in Table S23.

If the ANC HSI runs it will determine if the next contact should be scheduled. Women who are predicted to attend a minimum of four visits automatically are scheduled the next event in the sequence until they reach the fourth contact (Figure S5). The process repeats with every ANC contact that runs for an individual. It is assumed that all women who present for ANC1 (and choose to return for ANC2) will be scheduled their next contact dependent on their GA at presentation to ANC1, in keeping with the recommended ANC schedule (Table S22). For example, if a woman presents for ANC1 at 27 weeks, she will be booked to return at 30 weeks, the nearest next visit in the schedule.

Parameter Name	Description	Value*	Source and/or relevant calculations
**odds_early_init_anc4**	This parameter is scaled at intialisation of the simulation. The values shown here represents the known 'target' odds of a pregnant woman attending four or more ANC visits, with the first visit being prior to the fifth month of pregnancy (early ANC4+) the population for 2010 and 2015.<br><br>Once scaled, as the simulation runs this parameter is the odds of early ANC4+ for a woman who is younger than 20, nulliparous or primiparous, has received no formal education or only primary education and is not in the richest wealth quintile.<br><br>The parameters below starting with "aor_early_anc4" refer to the effect on the odds of attending early ANC4+	0.32 / 0.58	The model has been calibrated to replicate the coverage of early ANC4+ as reported by Ng'ambi et al. (40) as 24% in 2010 and 36% in 2015. The 'target' odds were calculated as coverage / (1-coverage) leading to these values. If required, manipulation of these values occurred during the process of calibration, with further detail on ANC calibration is provided in §4
**aor_early_anc4_20_24**	The effect of a pregnant woman's age being 20-24 years compared to 15-19 years	1.26	See **odds_early_init_anc4.**
**aor_early_anc4_25_29**	The effect of a pregnant woman's age being 25-29 years compared to 15-19 years	1.44	See **odds_early_init_anc4.**
**aor_early_anc4_30_34**	The effect of a pregnant woman's age being 30-34 years compared to 15-19 years	1.49	See **odds_early_init_anc4.**

Parameter Name	Description	Value*	Source and/or relevant calculations
**aor_early_anc4_35_39**	The effect of a pregnant woman's age being 35-39 years compared to 15-19 years	1.64	See **odds_early_init_anc4.**
**aor_early_anc4_40_44**	The effect of a pregnant woman's age being 40-44 years compared to 15-19 years	1.51	See **odds_early_init_anc4.**
**aor_early_anc4_45_49**	The effect of a pregnant woman's age being 45-49 years compared to 15-19 years	1.91	See **odds_early_init_anc4.**
**aor_early_anc4_2010**	The effect of the year being earlier than 2015	1.51	See **odds_early_init_anc4.**
**aor_early_anc4_2015**	The effect of the year being later than 2015	2.03	See **odds_early_init_anc4.**
**aor_early_anc4_parity_2_3**	The effect of a pregnant woman having previously delivered 2-3 previous times compared to delivering once or less	0.74	See **odds_early_init_anc4.**
**aor_early_anc4_parity_4_5**	The effect of a pregnant woman having previous delivered 4-5 previous times compared to delivering once or less	0.65	See **odds_early_init_anc4.**
**aor_early_anc4_parity_6+**	The effect of a pregnant woman having previous delivered 6 or more previous times compared to delivering once or less	0.61	See **odds_early_init_anc4.**
**aor_early_anc4_secondary_edu**	The effect of a pregnant woman having received secondary education compared to no education	1.24	See **odds_early_init_anc4.**

Parameter Name	Description	Value*	Source and/or relevant calculations
**aor_early_anc4_tertiary_edu**	The effect of a pregnant woman having received tertiary education compared to no education	2.64	See **odds_early_init_anc4.**
**aor_early_anc4_richest_wealth**	The effect of a pregnant woman being in the richest wealth quintile compared to the poorest	1.32	See **odds_early_init_anc4.**
**prob_late_initiation_anc4**	The probability that a woman who will not attend early ANC4+ will attend four or more ANC visits with the first visit occurring during or after 5 months of pregnancy.	0.3 / 0.29	This parameter was derived from calibration to the total proportion of women attending four or more ANC visits regardless of timing of initiation sourced from the Malawi DHS (11,12).
**prob_early_initiation_anc_below4**	The probability that a woman who will not attend four or more ANC visit will attend her first visit within the first five months of pregnancy.	0.115 / 0.101	See **prob_late_initiation_anc4.** This parameter was derived from calibration to proportion of women attending less than four ANC visits and timing of first ANC visit (11,12).
**prob_anc1_months_2_to_4**	A list of probabilities used in a probability weighted random draw determining if a woman's first ANC contact will occur in month 2, 3 or 4 of pregnancy.	[0.165, 0.165, 0.67] / [0.165, 0.364, 0.471]	The 2010 and 2015 DHS surveys in Malawi (11,12). Report the timing of first ANC visit by month of pregnancy. We have aimed to replicate this in the model and as such these parameters are derived through calibration to the proportion of first ANC visits by GA in the respective surveys.

Parameter Name	Description	Value*	Source and/or relevant calculations
prob_anc1_months_5_to_9	A list of probabilities used in a probability weighted random draw determining if a woman's first ANC contact will occur in month 5, 6, 7, 8, 9 of pregnancy or not at all.	[0.345, 0.245, 0.28, 0.015, 0.015, 0.1] / [0.41, 0.25, 0.21, 0.015, 0.015, 0.1]	See **prob_anc1_months_2_to_4.**
prob_seek_anc2	The probability that a woman who attends her first ANC contact, and is not predicted to attend early ANC4+, will return for a second contact.	0.98	The 2015 DHS survey (11) reports the total number of ANC visits attended by women during their last pregnancy. These parameters have been derived through calibration to the proportion of women by visit number.
prob_seek_anc3	The probability that a woman who attends her second ANC contact, and is not predicted to attend early ANC4+, will return for a third contact.	0.55	See **prob_seek_anc2**
prob_seek_anc5	The probability that a woman who attends her fourth ANC contact will return for a fifth contact.	0.125	See **prob_seek_anc2.** The 2015 DHS survey provides the proportion of women who received 0, 1, 2, 3 or 4+ visits during their last pregnancy (11). As this survey was conducted prior to the initiation of the 2016 WHO 8-contact recommendation it is assumed a small proportion of women receive more ANC contacts than four.
prob_seek_anc6	The probability that a woman who attends her fifth ANC contact will return for a sixth contact.	0.0625	See **prob_seek_anc2, prob_seek_anc5**

Parameter Name	Description	Value*	Source and/or relevant calculations
prob_seek_anc7	The probability that a woman who attends her sixth ANC contact will return for a seventh contact.	0.03125	See **prob_seek_anc2, prob_seek_anc5**
prob_seek_anc8	The probability that a woman who attends her seventh ANC contact will return for an eighth contact.	0.03125	See **prob_seek_anc2, prob_seek_anc5**
squeeze_factor_threshold_anc	The squeeze factor value over which an ANC HSI will not run and will be rescheduled for an individual attempting to receive ANC who will return the next day to receive treatment.	3	See **Table S15.**

** Where two values (or sets of values) are provided the first set is applied from 2010-2014 and the second set from 2015 onwards for a given simulation run (§1.2.1.1)*

Table S24- Parameters relating to care seeking for ANC

2.4.3.2 Intervention delivery and quality of care

Interventions are delivered to individuals who receive ANC according to the number of visits they have previously attended. The schedule of interventions by ANC contact number is presented in Table S24 and was developed from the Malawian ANC intervention matrix provided by colleagues at the Reproductive Health Directorate within the Ministry of Health and Population (MoH) (38), and reflects current national practice.

For individuals who present for their first ANC contact beyond the recommended GA (e.g., after 12 weeks GA), WHO guidelines recommend that all interventions which would have been delivered in earlier contacts are delivered at initiation of ANC. For example, in line with the schedule shown in Table S22, an individual who presents for their first contact at 26 weeks GA should receive any interventions they missed from contacts one and two and then those usually delivered at contact three, which is the number of contacts they should have received in line with their GA and the schedule. This logic is replicated within the model.

Table S24 is a summary of the interventions delivered during routine ANC, further detail on the impact of interventions on maternal or perinatal outcomes are provided in §3.

**Intervention**<br>*(ANC Contact at which delivered)*	**Consumables***	**Summary of modelled effect of intervention**
**Blood pressure measurement**<br>*(Each contact)*	*None (availability of equipment such as sphygmomanometer not currently captured)*	Individuals with hypertension due to one of the hypertensive disorders of pregnancy are admitted for inpatient care for the initiation of treatment.
**Urinalysis with urine dipstick**<br>*(Each contact)*	*Required:* Urine dipstick	Individuals who test positive for proteinuria due to pre-eclampsia are admitted for inpatient care for the initiation of treatment.
**Screening for depression**<br>*(Each contact)*	*Intervention delivered by another model.*	Whilst scheduling for this intervention occurs during ANC, screening and treatment for depression is managed by the depression module (see https://www.tlomodel.org/writeups.html). In short, if depression in pregnancy is detected then the individual is initiated on anti-depressants and referred for 'talking therapy'.
**Screening for HIV**<br>*(First contact)*	*Intervention delivered by another model.*	Whilst scheduling for this intervention occurs in ANC, screening and treatment for HIV is managed by the HIV module (see https://www.tlomodel.org/writeups.html). In short, if HIV is detected the woman is commenced on antiretroviral therapy. HIV positive women are then screened for tuberculosis.
**Screening for tuberculosis**<br>*(First contact)*	*Intervention delivered by another model.*	Whilst scheduling for this intervention occurs in ANC, screening and treatment for tuberculosis is managed by the tuberculosis module (see https://www.tlomodel.org/writeups.html). In short, women who are symptomatic are formally tested and, if positive, commenced on appropriate treatment
**Screening for syphilis**<br>*(First and fifth contact)*	*Required:* Rapid plasma reagin (RPR) test<br><br>*Optional:* Blood collecting tube, IV cannula, Disposable gloves	Individuals who test positive for syphilis on screening may be treated if consumables are available (see **Treatment for syphilis**).

**Point-of-care haemoglobin testing**<br>*(First and sixth contact)*	*Required:* N/A<br><br>*Optional:* Blood collecting tube, IV cannula, Disposable gloves	Individuals who are found to be anaemic are admitted for inpatient care for the initiation of treatment.
**Blood glucose testing for gestational diabetes**<br>*(Third contact)*	*Required:* Blood glucose level test<br><br>*Optional:* Blood collecting tube, IV cannula, Disposable gloves	Individuals with hyperglycaemia detected via screening due to gestational diabetes are admitted for inpatient care for the initiation of treatment.
**Daily Iron and folic acid supplementation**<br>*(First contact)*	*Required:* Iron and Folic Acid, tablet, 225 mg* (x3)<br><br>*Dose is 325mg twice a day	If treatment is delivered and the individual will be adherent, then the individual's monthly risk of developing anaemia is reduced (RR 0.30 (42)).
**Balanced energy and protein (BEP) supplementation**<br>*(First contact)*	*Required:* Dietary supplements	If treatment is delivered, then the individual's monthly risk of antenatal stillbirth is reduced (RR 0.6 (43)).
**Insecticide treated bed nets**<br>*(First contact)*	*Intervention delivered by another model.*	Malaria incidence in the pregnant population is driven by the Malaria model. Coverage estimates for bed net use in Malawi are used to derive expected incidence across age groups. Consumable use is captured through the ANC model. (See https://www.tlomodel.org/writeups.html).
**Tetanus toxoid vaccination**<br>*(First and second contact)*	*Intervention delivered by another model.*	At the time of writing vaccine administration is represented through the Extended Programme on Immunisation model alongside several vaccine preventable diseases (see https://www.tlomodel.org/writeups.html). Currently tetanus in mothers or newborns is not modelled and therefore this intervention does not have an effect.

**Treatment for syphilis**<br>*(First and fifth contact)*	<u>*Required:*</u> Benzathine benzylpenicillin, 2.4 million international units<br><br><u>*Optional:*</u> IV cannula, Disposable gloves	Individuals who are screened positive for syphilis and receive treatment are assumed to be free of infection, removing the effect of syphilis infection on risk of antenatal stillbirth.
**Daily calcium supplementation†**<br>*(Second contact)*	<u>*Required:*</u> Calcium, tablet, 600 mg*<br>**Dose is 3 tablets per day*	If the treatment is delivered, then the individual's monthly risk of developing pre-eclampsia (RR 0.45 (44)) and gestational hypertension (RR 0.65 (44)) is reduced.
**Albendazole**<br>*(Second contact)*	<u>*Required:*</u> Albendazole 200mg	Due to lacking evidence of effect on maternal outcomes that are included in the model this intervention does not have a modelled effect. It is included to map consumable use accurately.
**Intermittent preventive treatment of malaria during pregnancy (IPTp)**<br>*(Second, third, fourth, fifth and seventh contact)*	*Intervention delivered by another model*	Malaria incidence in the pregnant population is driven by the Malaria model, (see https://www.tlomodel.org/writeups.html). Receipt of IPTp clears current malaria infection reducing the probability of outcomes for which malaria is a predictor including anaemia, preterm labour, and stillbirth.

('Required' consumables must be available for the intervention to be delivered. Optional consumables are logged but intervention delivery is not conditional on their availability; †Intervention only delivered to women "at risk" of developing pre-eclampsia as defined in Malawian guidelines (Body Mass Index (BMI) <18))*

Table S25- Interventions delivered during ANC

To replicate quality of ANC, intervention delivery is conditional on the availability of relevant consumables and the probability that a HCW will administer the intervention as shown in the following equation:

$$P(Intervention) = P(\boldsymbol{intervention\_delivered})_{(Int)} * \prod c_{i(t)} \qquad (7)$$

Where *P(Intervention)* is the probability of intervention delivery and P(***intervention_delivered***)$_{(int)}$, is the probability that the HCW providing ANC will administer an intervention given consumables are available. These probabilities have been calculated from data from the SPA survey (45) and the HHFA 2018/19 (46) in Malawi, in which direct observation of ANC was undertaken in facilities to ascertain the proportion of women who received the recommended interventions. Where the observed proportion of women receiving an intervention was greater in the Malawi Service Provision Assessment Survey (SPA) (2013/14) or Harmonized Health Facilities Assessment (HHFA) (2019) than the mean availability of consumables for the relevant facility level, no quality parameter was used. Table S25 details these parameters alongside the values for the sensitivity and specificity of any screening interventions. As discussed previously, $\prod c_{i(t)}$ is the probability that all required consumables for intervention delivery are available.

Parameter Name	Description	Value*	Source and/or relevant calculations
**prob_intervention_delivered_urine_ds**	The probability that a HCW will attempt to conduct urinalysis during an ANC contact if consumables are available.	0.53	The 2014 Malawi SPA survey (45) reports data from observation of ANC contacts conducted in Malawi in which 9% of women across facility types underwent urine dipstick testing.<br><br>In the model this level of intervention coverage is assumed to be the product of consumable availability and the probability a HCW will choose to deliver this intervention – which is represented by this parameter. This probability is unknown and was derived from the total intervention coverage taken from the SPA (9%) and the mean consumable availability at level 1a (17%) as 0.17***0.53** = 0.09.
**prob_intervention_delivered_bp**	The probability that a HCW will measure a woman's blood pressure during an ANC contact.	0.70 / 0.69	Both the 2014 Malawi SPA survey (45) and the later 2019 HHFA (46) report the proportion of women during observed ANC who received blood pressure measurement (70% and 69% respectively). As availability of BP equipment is not captured in the mode these probabilities are sourced directly from the survey.
**prob_intervention_delivered_syph_test**	The probability that a HCW will attempt to administer a syphilis test during an ANC contact.	0.43 / 0.14	See **prob_intervention_delivered_urine_ds.** The proportion of women receiving syphilis screening during ANC was taken from Malawi's AIDS Response Progress Reports as 22% in 2010 and 7% in 2015 (47,48). The mean availability of the required consumables at level 1a is 51%.

Parameter Name	Description	Value*	Source and/or relevant calculations
**prob_intervention_delivered_gdm_test**	The probability that a HCW will attempt to administer a test for gestational diabetes during an ANC contact if consumables are available.	0.48	See **prob_intervention_delivered_urine_ds.** Data on the proportion of women screened via blood glucose monitoring during ANC in Malawi is not available. As such it is assumed the overall coverage is the same as point of care haemoglobin testing due to similarities in testing approach. The 2014 SPA reports 14% of women were observed to have haemoglobin testing (45) whilst the average consumable availability for blood glucose testing at level 1a is 31%
**sensitivity_bp_monitoring**	The sensitivity of blood pressure measurement for hypertension during pregnancy	0.74	Sourced directly from Karnjanapiboonwong et al. (49)
**specificity_bp_monitoring**	The specificity of blood pressure measurement for hypertension during pregnancy	0.79	Sourced directly from Karnjanapiboonwong et al. (49)
**sensitivity_urine_protein_1_plus**	The sensitivity of urinalysis via a dipstick test for proteinuria during pregnancy	0.541	Sourced directly from Gangaram et al. (50)
**specificity_urine_protein_1_plus**	The specificity of urinalysis via a dipstick test for proteinuria during pregnancy	0.841	Sourced directly from Abebe et al. (51)
**sensitivity_poc_hb_test**	The sensitivity of point-of-care haemoglobin testing for anaemia during pregnancy	0.851	Sourced directly from Van Den Broek et al. (52)
**specificity_poc_hb_test**	The specificity of point-of-care haemoglobin testing for anaemia during pregnancy	0.801	Sourced directly from Van Den Broek et al. (52)

Parameter Name	Description	Value*	Source and/or relevant calculations
**sensitivity_blood_test_glucose**	The sensitivity of blood glucose testing for gestational diabetes in pregnancy	1.0	Assumed.
**specificity_blood_test_glucose**	The specificity of blood glucose testing for gestational diabetes in pregnancy	1.0	Assumed.
**sensitivity_blood_test_syphilis**	The sensitivity of syphilis testing for syphilis during pregnancy	0.82	Sourced directly from Bristow et al. (53)
**specificity_blood_test_syphilis**	The specificity of syphilis testing for syphilis during pregnancy	0.96	Sourced directly from Bristow et al. (53)
**sensitivity_fbc_hb_test**	The sensitivity of full blood count testing for anaemia during pregnancy	1.0	Assumed
**specificity_fbc_hb_test**	The specificity of full blood count testing for anaemia during pregnancy	1.0	Assumed

** Where two values (or sets of values) are provided the first set is applied from 2010-2014 and the second set from 2015 onwards for a given simulation run (§1.2.1.1)*

Table S26 – Parameters relating to quality of ANC and sensitivity and specific of screening interventions

2.4.4 Antenatal inpatient care

In addition to routine ANC, pregnant individuals may also receive inpatient care which is scheduled either via screening and referral from an ANC appointment or when individuals seek emergency healthcare following the onset of obstetric complications. These conditions, which include antepartum haemorrhage (APH), severe pre-eclampsia, eclampsia, premature rupture of membranes (PROM) and antenatal sepsis, were deemed likely to develop symptoms of sufficient severity to initiate possible care seeking through discussion with a clinical expert in obstetrics (54). The parameter in Table S26 represents probability of care seeking.

Parameter Name	Description	Value	Source and/or relevant calculation
**prob_seek_care_pregnancy_complication**	The probability that a woman experiencing APH, severe pre-eclampsia, eclampsia, PROM, or antenatal sepsis will seek emergency antenatal care.	0.782	Sourced directly from Chinkhumba et al. (36)

Table S27 - Parameter representing care seeking following antenatal complications

2.4.4.1 Intervention delivery and quality

Table S27 details the treatment delivered during antenatal inpatient care categorised by presenting condition. Where care includes delivery of an EmONC intervention, probability of intervention delivery is calculated as described in §2.3, including capturing the effect of delays in care seeking described in §2.2. Further detail on how interventions act mechanistically within the relevant condition models is provided in §3.

Presenting condition*	Intervention(s)	Consumables	Modelled effect of intervention
**Anaemia**	**Full blood count (FBC)**	_Required:_ Complete blood count<br><br>_Optional:_ Blood collecting tube, 5 ml, Cannula iv, Disposable gloves	Returns severity of anaemia in the tested individual with the result used to guide treatment delivery.
	If FBC determines anaemia is mild/moderate…<br>**Iron and folic acid supplementation (IFA)**	_Required:_ Iron and Folic Acid, tablet, 225 mg* (x3)<br><br>*Dose is 325mg twice a day	Initiated in individuals who are not already receiving IFA. A fixed probability that initiation of IFA will resolve current anaemia is applied (0.7). See §3.1.1 for how this value is calculated.
	If FBC determines anaemia is severe…<br>**Blood transfusion**	_Required:_ Blood, one unit<br><br>_Optional:_ Cannula iv, Giving set iv, Disposables gloves	A fixed probability that a transfusion will resolve current anaemia is applied (0.9). See §3.1.1 for how this value is calculated.
**Gestational Diabetes†**	*Firstline treatment for new cases…*<br>**Diet and exercise**	*N/A*	Once initiated on treatment, a fixed probability (0.5) is used to determine if this treatment will be effective in controlling hyperglycaemia. If so, the effect of gestational diabetes on risk of antenatal stillbirth is removed (see §3.1.2 for how this value is calculated). Otherwise, the individual is scheduled to return for second line treatment.
	Second line treatment for cases…<br>**Oral antidiabetics**	_Required:_ Glibenclamide 5mg* (x2)<br><br>*Dose is 10mg daily	Once initiated on treatment a fixed probability (0.936 (55)) is used to determine if this treatment will be effective in controlling hyperglycaemia. If so, the effect of gestational diabetes on risk of antenatal stillbirth is removed (see §3.1.12). Otherwise, the individual is scheduled to return for third line treatment.

	Third line treatment for cases…<br>**Insulin**	*Required:* Insulin, soluble, 100 IU/ml	Once initiated, treatment is assumed to control hyperglycaemia removing the effect of gestational diabetes on risk of antenatal stillbirth (see §3.1.12).
**Hypertensive disorders**	*For mild pre-eclampsia, gestational hypertension…*<br>**Oral antihypertensives**	*Required:* Methyldopa 250mg* (x4)<br><br>*Dose is 1g daily	Reduces the risk of progression from mild to severe gestational hypertension (RR 0.45 (56)).
	For severe gestational hypertension or severe pre-eclampsia/eclampsia…<br>**Intravenous (IV) antihypertensives**	*Required:* Hydralazine 20mg ampoule<br><br>*Optional:* Cannula iv, Giving set iv, Disposables gloves	For individuals with severe gestational hypertension the intervention is assumed to revert severe hypertension to mild hypertension, averting associated risk of death from severe gestational hypertension (see §3.1.8).<br><br>For individuals with severe pre-eclampsia/eclampsia the intervention reduces the risk of death secondary to these conditions (0.5 (37)).
	For severe pre-eclampsia or eclampsia…<br>**Anticonvulsants (for severe pre-eclampsia or eclampsia) plus case management**	*Required:* Magnesium sulphate, injection, 500 mg/ml in 10-ml ampoule<br><br>*Optional*: Misoprostol, tablet, 200 mcg, Oxytocin, injection, 10 IU in 1 ml ampoule, Sodium chloride, injectable solution, 0.9 %, 500 ml, Cannula IV, Giving set, Disposable gloves, Oxygen, 1000 litres, primarily with oxygen cylinders, Complete blood count, Foley catheter, Urine bag 2000ml	For women with severe pre-eclampsia, treatment reduces the risk of progression from severe pre-eclampsia to eclampsia during labour (RR 0.41 (57)) and reduces risk of death from severe pre-eclampsia (RR 0.4 (37)).<br><br>For women with eclampsia treatment reduces risk of death from eclampsia (RR 0.4 (37)).

	For severe pre-eclampsia or eclampsia… **Referral for delivery**	*N/A*	In line with Malawian guidelines, emergency delivery is recommended for cases of severe pre-eclampsia/eclampsia (32). Individuals are scheduled for immediate delivery either via induction, AVD or CS.
**Antepartum Haemorrhage**	**Referral for delivery and further treatment**	*N/A*	In line with Malawian guidelines, emergency delivery is recommended for cases of APH (32). Individuals are scheduled for delivery via CS which may be delayed, according to aetiology and severity of bleed, until GA has increased. Further treatment is delivered to mothers as delivery occurs (i.e., blood transfusion) which reduces risk of death. (See §3.1.7).
**Premature Rupture of Membranes (PROM)**	**Prophylactic IV antibiotics**	*Required:*<br>Benzathine benzylpenicillin, 2.4 million international units<br><br>*Optional:*<br>Cannula iv, Giving set iv, Disposables gloves	Reduces the risk of early onset neonatal sepsis (RR 0.61 (58)) and reduces risk of maternal sepsis secondary to chorioamnionitis (RR 0.66 (59)).
	**Referral for delivery**	N/A	In line with Malawian guidelines, emergency delivery is recommended for cases of PROM (32). If PROM presents without infection and GA is below 37, delivery is delayed until gestation increases. Otherwise, delivery is scheduled.

**Sepsis – chorioamnionitis†**	**Referral for delivery and further treatment**	N/A	In line with Malawian guidelines, emergency delivery is recommended for cases of chorioamnionitis (32). Further treatment is delivered to mothers as delivery occurs (e.g., maternal sepsis case management) which reduces risk of death. (See §3.1.6.)

**If multiple conditions are present on admission, treatment for all conditions will be delivered*

†Treatment is initialised in inpatient care. Initiation of second- or third-line treatment is managed by a separate HSI not described here.

Table S28 – Summary of interventions delivered as part of inpatient antenatal care for relevant antenatal conditions

2.4.5 Induction of labour

Finally, induction of labour for women whose pregnancy continues post term is modelled. Currently this is not explicitly linked with routine ANC and instead a fixed probability, **prob_seek_care_induction** applied to post term women each week until they either seek care or go into labour spontaneously. Due to lacking data on care seeking for induction in women who are post-term in Malawi an assumption is made about this probability. Future versions of the model will need to link routine ANC to induction for post-term women.

Parameter Name	Description	Value	Source and/or relevant calculation
**prob_seek_care_induction**	The probability that a woman whose pregnancy has continued beyond 41 weeks will seek care for induction of labour.	0.2	This parameter is assumed.

Table S29 – Parameter representing care seeking for induction of labour.

2.5 Intrapartum and immediate newborn

2.5.1 Care seeking

As evident from the model structure diagram in Figure 5 of the accompanying manuscript the location in which a mother will give birth is determined at the onset of labour. The results from a multinomial logistic regression model developed using data from the Malawian 2010 and 2015 DHS data sets (11,12) are used to calculate the probability that an individual will deliver at home, in a health centre or in a hospital to account for the effect of relevant sociodemographic variables on delivery location. The parameters for these models are available in Table S29 alongside details of the relevant coefficients.

Women who are scheduled to deliver at home may decide to present for care following the onset of any intrapartum conditions with parameter **prob_careseeking_for_complication** representing the probability of care seeking. The model simulates delays in healthcare seeking and this is also relevant for intrapartum care. All women who present for intrapartum care after initially labouring at home are assumed to be delayed whilst women

who are scheduled to present for care when labour onsets are determined to be delayed according to **prob_delay_one_two_fd** (Table S15).

Parameter Name	Description	Value*	Source and/or relevant calculation
**odds_deliver_in_health_centre**	This parameter is scaled at intialisation of the simulation. The value shown here represents the known 'target' odds that a mother in labour will deliver in a health centre compared to a hospital.<br><br>Once scaled, as the simulation runs this parameter is the odds that a mother in labour will deliver in a health centre compared to a hospital if aged less than 20 years, is in wealth quintile 5, is nulliparous or primiparous, lives in an urban setting and is not married.<br><br>The parameters below starting with "rrr_hc_delivery" refer to the effect on the odds of delivering in a health centre compared to a hospital.	0.69 / 1.08	A multinomial logistic regression model was built using data from 2010 and 2015 Malawian DHS surveys sets (11,12) to determine the effect of relevant coefficients on delivery location. The intercept value for this model is unknown within the modelled population. The 'target' odds of health centre delivery were calculated using the coverage of health centre delivery in the DHS as coverage / (1-coverage) leading to these values.
**rrr_hc_delivery_age_20_24**	The effect of a woman's age being 20-24 years compared to 15-19	0.79 / 1	*See* **odds_deliver_in_health_centre.**
**rrr_hc_delivery_age_25_29**	The effect of a woman's age being 25-29 years compared to 15-19	0.5 / 0.56	*See* **odds_deliver_in_health_centre**
**rrr_hc_delivery_age_30_34**	The effect of a woman's age being 30-34 years compared to 15-19	0.27 / 0.32	*See* **odds_deliver_in_health_centre**
**rrr_hc_delivery_age_35_39**	The effect of a woman's age being 35-39 years compared to 15-19	0.13 / 0.14	*See* **odds_deliver_in_health_centre**

**rrr_hc_delivery_age_40_44**	The effect of a woman's age being 40-44 years compared to 15-19	0.06 / 0.07	*See* **odds_deliver_in_health_centre**
**rrr_hc_delivery_age_45_49**	The effect of a woman's age being 45-49 years compared to 15-19	0.02 / 0.03	*See* **odds_deliver_in_health_centre**
**rrr_hc_delivery_wealth_4**	The effect of a woman being within the fourth wealth quintile compared to the fifth	0.82 / 0.79	*See* **odds_deliver_in_health_centre.**
**Rrr_hc_delivery_wealth_3**	The effect of a woman being within the third wealth quintile compared to the fifth	0.77 / 0.73	*See* **odds_deliver_in_health_centre**
**rrr_hc_delivery_wealth_2**	The effect of a woman being within the second wealth quintile compared to the fifth	0.62 / 0.58	*See* **odds_deliver_in_health_centre**
**rrr_hc_delivery_wealth_1**	The effect of a woman being within the first wealth quintile compared to the fifth	0.55 / 0.43	*See* **odds_deliver_in_health_centre**
**rrr_hc_delivery_parity_3_to_4**	The effect of a woman having previously delivered three or four children compared to two or less	2 / 1.85	*See* **odds_deliver_in_health_centre**
**rrr_hc_delivery_parity_>4**	The effect of a woman having previously delivery four or more children compared to two or less	3.9 / 3.8	*See* **odds_deliver_in_health_centre**
**rrr_hc_delivery_rural**	The effect of a woman living in a rural setting compared to urban setting	1.99 / 1.81	*See* **odds_deliver_in_health_centre**
**rrr_hc_delivery_married**	The effect of a woman being currently married compared to never being married	1.3 / 1.42	*See* **odds_deliver_in_health_centre**

**odds_deliver_at_home**	This parameter is scaled at intialisation of the simulation. The value shown here represents the known 'target' odds that a mother in labour will deliver at home compared to a hospital.<br><br>Once scaled, as the simulation runs this parameter is the odds that a mother in labour will deliver at home compared to in a hospital if aged less than 20 years, living in an urban setting, having received tertiary education, being in wealth quintile 5, is nulliparous or primiparous, and is not married.<br><br>The parameters below starting with "rrr_hb_delivery" refer to the effect on the odds of delivering at home compared to a hospital	0.37 / 0.09	*See* **odds_deliver_in_health_centre.**
**rrr_hb_delivery_age_20_24**	The effect of a woman's age being 20-24 years compared to 15-19 years at the time of delivery	1.42 / 1	*See* **odds_deliver_in_health_centre.**
**rrr_hb_delivery_age_25_29**	The effect of a woman's age being 25-29 years compared to 15-19 years at the time of delivery	1 / 0.58	*See* **odds_deliver_in_health_centre.**
**rrr_hb_delivery_age_30_34**	The effect of a woman's age being 30-34 years compared to 15-19 years at the time of delivery	0.42 / 0.25	*See* **odds_deliver_in_health_centre.**
**rrr_hb_delivery_age_35_39**	The effect of a woman's age being 35-39 years compared to 15-19 years at the time of delivery	0.24 / 0.16	*See* **odds_deliver_in_health_centre.**
**rrr_hb_delivery_age_40_44**	The effect of a woman's age being 40-44 years compared to 15-19 years at the time of delivery	0.12 / 0.07	*See* **odds_deliver_in_health_centre.**

**rrr_hb_delivery_age_45_49**	The effect of a woman's age being 45-49 years compared to 15-19 years at the time of delivery	0.05 / 0.04	*See* **odds_deliver_in_health_centre.**
**rrr_hb_delivery_rural**	The effect of a woman living in a rural setting compared to an urban setting	1.73 / 1	*See* **odds_deliver_in_health_centre.**
**rrr_hb_delivery_primary_education**	The effect of a woman having primary education compared to no education	0.76 / 0.79	*See* **odds_deliver_in_health_centre.**
**rrr_hb_delivery_secondary_education**	The effect of a woman having secondary education compared to no education	0.46 / 0.51	*See* **odds_deliver_in_health_centre.**
**rrr_hb_delivery_wealth_4**	The effect of a woman being within the fourth wealth quintile compared to the fifth	0.82 / 0.74	*See* **odds_deliver_in_health_centre.**
**rrr_hb_delivery_wealth_3**	The effect of a woman being within the third wealth quintile compared to the fifth	0.72 / 0.68	*See* **odds_deliver_in_health_centre.**
**rrr_hb_delivery_wealth_2**	The effect of a woman being within the second wealth quintile compared to the fifth	0.41 / 0.49	*See* **odds_deliver_in_health_centre.**
**rrr_hb_delivery_wealth_1**	The effect of a woman being within the first wealth quintile compared to the fifth	0.2 / 0.17	*See* **odds_deliver_in_health_centre.**
**rrr_hb_delivery_parity_3_to_4**	The effect of a woman having previously delivery three or four children compared to two or less	2.4 / 1	*See* **odds_deliver_in_health_centre.**
**rrr_hb_delivery_parity_>4**	The effect of a woman having previously delivery four or more children compared to two or less	5.25 / 1	*See* **odds_deliver_in_health_centre.**

**rrr_hb_delivery_married**	The effect of a woman being currently married compared to never being married	0.63 / 1	*See* **odds_deliver_in_health_centre.**
**probability_delivery_hospital**	The probability that a woman will deliver in a hospital	0.32 / 0.40	*See* **odds_deliver_in_health_centre.** The value of this parameter was derived through calibration to the proportion of women giving birth in a hospital as reported in the 2010 and 2015 DHS surveys (11,12).
**prob_careseeking_for_complication**	The probability that a woman who is in labour at home and develops a complication will seek intrapartum care at a health facility.	0.782	Sourced directly from Chinkhumba et al. (36).

** Where two values (or sets of values) are provided the first set is applied from 2010-2014 and the second set from 2015 onwards for a given simulation run (§1.2.1.1)*

Table S30 – Parameters representing care seeking intrapartum care

2.5.2 Intervention delivery

Once location of delivery has been determined, healthcare is delivered to labouring individuals through HSIs representing intrapartum care at a health centre or at a hospital. Intrapartum care at a health centre occurs at level 1a of the health system, whilst hospital-based care can occur at either level 1b (district hospital) or level 2. Individuals who seek healthcare after condition onset during labour at home will present at either facility type with equal probability. Interventions within the model delivered before, during or immediately following labour can be categorised as preventative or curative, addressing the conditions that are described in §3.

2.5.2.1 Preventative and curative interventions and operative delivery

As with many of the interventions introduced thus far, the treatment effect of curative interventions is applied to the probability of death from a complication or intrapartum stillbirth whilst the effect of preventative treatments is to reduce individual risk of a complication as described in the following descriptions of the complication models. The interventions selected for inclusion in the model are taken from several sources, including Malawian Obstetrics and Gynaecology Clinical guidelines (32) ,the Malawi Standard Treatment Guidelines (31), and the Malawian EHP (60) to accurately reflect intrapartum care interventions delivered in Malawi. They are summarised in Table S30.

2.5.2.2 Referral

The model replicates the process of referral from lower level to higher level facilities. It is assumed that individuals who deliver at a health centre have access to BEmONC interventions and, if need for CEmONC care is identified (e.g., caesarean delivery as treatment of obstructed labour), then referral to a higher-level facility (level 1b and above) occurs. Whilst the process maps the additional requirements of HCW time and consumables, we do not currently model any potential effects of interfacility transfer on maternal and neonatal outcomes.

**Intervention**<br>*(Modelled indication)*	**Consumables**	**Modelled treatment effect and source***
Preventative interventions:		
**Clean birth practices**<br>(*All deliveries)*	*Required:* Clean delivery kit, Chlorhexidine 1.5% solution	Reduces risk of intrapartum and postpartum sepsis (RR 0.4 (37)) and reduces risk of early onset neonatal sepsis (RR 0.73 (61)).
**Antenatal corticosteroids**<br>*(Preterm deliveries)*	*Required:* Dexamethasone 5mg/ml<br><br>Optional: Cannula IV, Giving set, Disposable gloves	Reduces the risk of respiratory distress syndrome (RDS) in preterm neonates (RR 0.69 (62)).
**Antibiotics for PROM**<br>*(Premature rupture of membranes)*	*Described previously in Table S27.*	*Described previously in Table S27.*
**Active management of the third stage of labour**<br>*(All deliveries)*	*Required:* Oxytocin, injection, 10 IU in 1 ml ampoule<br><br>Optional: Cannula IV, Giving set, Disposable gloves	Reduces the risk of PPH secondary to uterine atony and retained placenta (RR 0.34 (63)).
Curative interventions:		
**Intravenous antihypertensives**<br>*(Severe pre-eclampsia, Eclampsia, Severe gestational hypertension)*	*Described previously in Table S27.*	*Described previously in Table S27.*
**Anticonvulsants plus case management**<br>(*Severe pre-eclampsia, Eclampsia)*	*Described previously in Table S27.*	*Described previously in Table S27.*
**Maternal sepsis case management**<br>*(Antenatal/intrapartum sepsis)*	*Required:* Benzylpenicillin 3g (5MU), Gentamycin, injection, 40 mg/ml in 2 ml vial	Reduces risk of sepsis related death (RR 0.2 (37)).

	Optional: Cannula IV, Giving set, Disposable gloves, Oxygen, Foley catheter, Urine bag 2000ml, complete blood count	
**Blood transfusion**<br>*(Antenatal/intrapartum haemorrhage, Uterine rupture)*	*Required:* Blood, one unit (x2)<br><br>*Optional:* Cannula iv, Giving set iv, Disposables gloves	Reduces risk of death from APH and/or uterine rupture (RR 0.4 (37)).
**Assisted vaginal delivery (AVD)**<br>*(Obstructed labour, Severe pre-eclampsia, Eclampsia)*	*Required:* Vacuum, obstetric<br><br>*Optional***: Lidocaine (in dextrose 7.5%), ampoule 2 m', Benzylpenicillin 3g (5MU), Gentamycin, injection, 40 mg/ml in 2 ml vial, Sodium chloride, injectable solution, 0.9 %, 500 ml, Cannula IV, Giving set, Disposable gloves, Complete blood count, Foley catheter, Urine bag 2000ml, Paracetamol, tablet, 500 mg, Pethidine, 50 mg/ml, 2 ml ampoule, Gauze, absorbent 90cm x 40m, Suture pack<br><br>***For obstructed labour 'case management'*	Reduces the risk of intrapartum stillbirth (RR 0.2). This treatment effect is an assumption and is discussed further in §3.1.12.
**Caesarean delivery (CS)**<br>*(Obstructed labour, Severe pre-eclampsia, Eclampsia, Antenatal/intrapartum haemorrhage, Uterine rupture)*	*Required:* Halothane (fluothane), Ceftriaxone 1g, Metronidazole 200mg<br><br>*Optional:* Scalpel blade, Cannula iv, Paracetamol, Diclofenac injection, Pethidine, 50 mg/ml, 2 ml	Reduces the risk of intrapartum stillbirth (RR 0.2). This treatment effect is an assumption and is discussed further in §3.1.12. Also reduces risk of maternal death due to APH (RR 0.25 (37)).

	ampoule', Foley catheter, Urine bag 2000ml, Hartmann's solution 1000 ml, Sodium chloride injectable solution 0.9 % 500 ml, Giving set	
**Surgical repair of uterus**<br>*(Uterine rupture)*	*See **caesarean delivery**– consumable requirements assumed to be the same.*	Reduces risk of death from uterine rupture (RR 0.25 (37)).
**Hysterectomy**<br>*(Uterine rupture)*	*See **caesarean delivery**– consumable requirements assumed to be the same.*	Reduces risk of death from uterine rupture (RR 0.25 (37)).
**Newborn resuscitation**<br>*(Neonatal encephalopathy, Preterm respiratory distress syndrome, respiratory depression (other))*	*Required:* Infant resuscitator, bag and mask	Reduces the risk of death due to neonatal encephalopathy (RR 0.6 (64)).<br><br>Reduces the risk of death due to preterm respiratory distress syndrome (RR 0.8 (64)).<br><br>Reduces the risk of death in neonates with respiratory depression (RR 0.6 (64)).<br><br>Reduces risk of encephalopathy in neonates with respiratory distress syndrome (64) as described further in §3.2.2

Table S31 – Interventions delivered as part of intrapartum care

2.5.2.3 Operative delivery population rates

As shown in Table S30 above, AVD and CS are scheduled for individuals in the model in response to a set of core complications. However, all possible indications for operative delivery are not explicitly captured in the model (e.g., foetal distress, maternal exhaustion). To ensure the correct rate of operative delivery is achieved in the model, when compared to data sources in Malawi, a probability of either AVD or CS for those who are not already scheduled to receive these interventions is applied to mothers and the appropriate care is scheduled. These probabilities are shown in Table S31 below.

**Parameter Name**	**Description**	**Value***	**Source and/or relevant calculation**
**residual_prob_avd**	The probability that a mother will deliver via AVD secondary to an indication not included in the model.	0.128	The AVD rate in Malawi is reported as 1% in the 2015 integrated HIV report in Malawi (65). The model is calibrated to this rate. The value is much higher than 0.01 due to limited availability of AVD.
**residual_prob_caesarean**	The probability that a mother will deliver via CS secondary to an indication not included in the model.	0.0188 / 0.022	The caesarean delivery rate in Malawi is reported in the 2010 and 2015 national EmONC needs assessment surveys (33,34) to which the model is calibrated. The rate was 3.7% in 2010 and 4% in 2015.

** Where two values (or sets of values) are provided the first set is applied from 2010-2014 and the second set from 2015 onwards for a given simulation run (§1.2.1.1)*

Table S32 – Parameters representing the probabilities of operative delivery in mothers without modelled indications

2.6 Postnatal healthcare

Modelling of postnatal healthcare was designed to replicate the recommended care and interventions described in the Malawian National Reproductive Health Service Delivery Guidelines (NRHSDG) (2014-2019), which are the most recent available PNC guidelines for Malawi (66). The NRHSDG recommend that all women receive PNC with the first 48-hours after birth, ideally with women who have delivered in a health facility, and their newborns,

receiving postnatal care within six hours of delivery; and women who delivered at home should receive at least one PNC contact within a maximum of three days (66). This is in line with the most recent WHO postnatal care guidelines (67). Additionally, guidelines recommended further PNC at weeks 1 and 6 of the postnatal period (66). In the model, the proportion of women who receive at least one PNC contact is simply replicated, and percentage of those women who received care within or following 48 hours from birth. In addition to the first visit, women may seek further PNC following the onset of postnatal complications as described below. This decision was made due to the availability of data on these outcomes from Malawi captured by the DHS allowing for model calibration.

It is assumed that PNC can be delivered to mothers across multiple levels of the health system, as designated by the I's facility level – Figure S3. For women who deliver in a facility and receive early PNC following birth, this will occur at the same facility level at which intrapartum care was delivered – as in reality these individuals will receive care at the same facility they deliver at. This logic is also applied to the inpatient care events. Otherwise, for PNC sought from the community, a random draw from a discrete uniform distribution determines the facility level from which treatment is delivered.

2.6.1 Maternal postnatal care

2.6.1.1 Care seeking for maternal postnatal care

Figure S6 is a diagrammatic representation of how PNC is scheduled within the model, separated by women who do and do not experience immediate postnatal complications. Immediately following birth, for mothers without complications the probability of receiving PNC is calculated via a logistic regression model using data from the Malawian DHS surveys in 2010 and 2015 to determine the socioeconomic and health system factors impacting probability of postnatal care (2). The equation is visible within the figure and the parameters representing the intercept for this model and the coefficient values are shown in Table S32 below. If PNC will be received, a probability weighted random draw using probabilities in parameter **prob_timings_pnc** determines if PNC will be received within forty-eight hours following birth or after this, and similarly is derived from the Malawian DHS survey (11,12).

If a mother develops complications later within the postnatal period, the probability they will seek care is **prob_care_seeking_postnatal_emergency**. This process may repeat multiple times across the postnatal period allowing for several PNC contacts in women who experience more than one complication. For mothers who *are* experiencing complications it is assumed that the probability of receiving PNC is greater, as facility staff are likely to triage immediate care based on clinical need, and mothers with complications at home may be more likely to present to facilities postnatally, as women with knowledge of obstetric danger signs are shown in Malawi and other settings to be more likely to receive PNC (68,69). The model is calibrated to DHS data relating to coverage of maternal PNC in the population, and as such parameter **prob_careseeking_for_complication_pn** represents a higher chance of PNC compared to without complications. This parameter is also used to determine if care will be sought later in the postnatal period for surviving who experience complications.

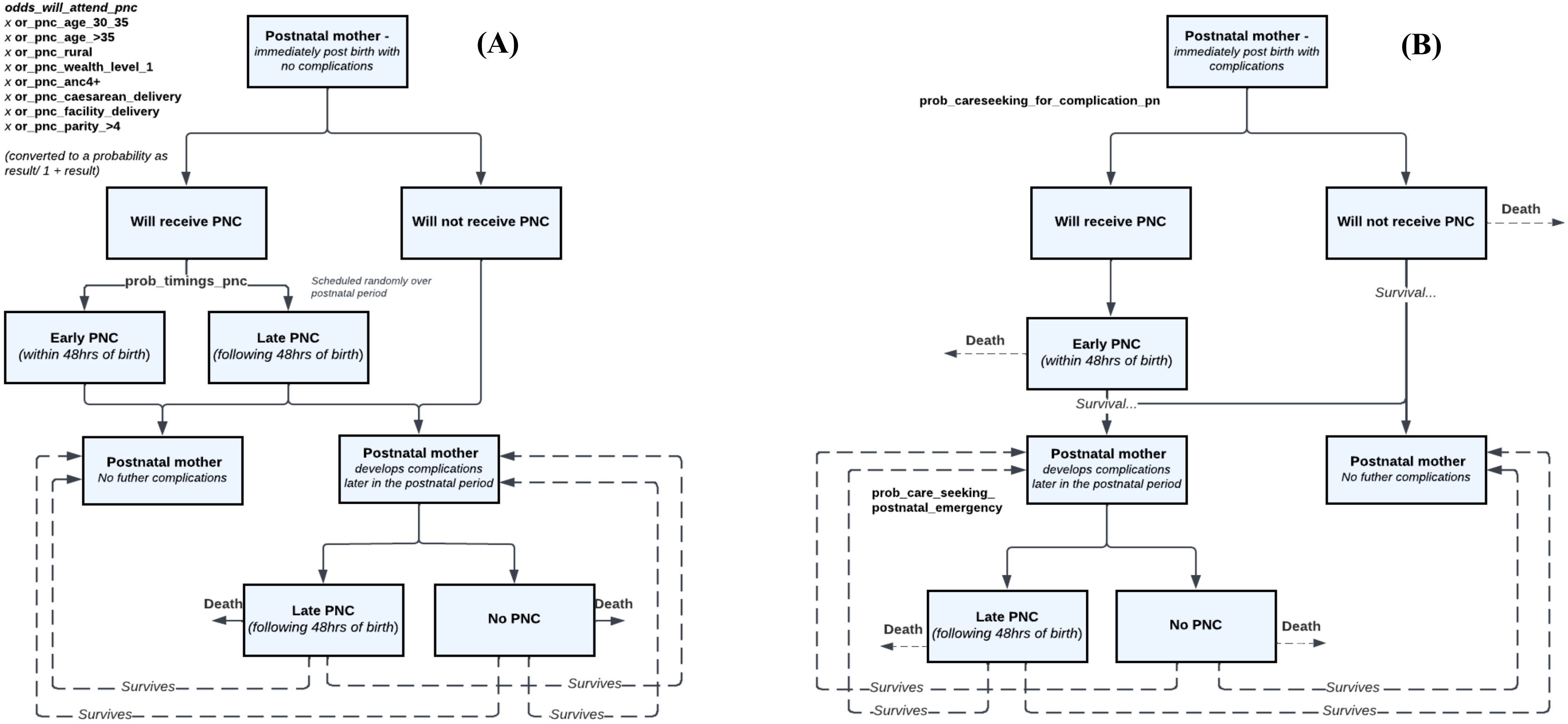

Figure S6 – Diagrammatic representation of PNC scheduling in the model

Diagrams showing how PNC is scheduled for mother's dependent on whether they have experience complications or not following birth. Parameters representing the probability of PNC attendance, and effect of relevant predictors are also defined. (A) Represents care seeking for PNC where the mother does not have immediate postnatal complications. (B) resents care seeking for PNC where the mother does have immediate postnatal complications.

Parameter Name	Description	Value*	Source and/or relevant calculation
**odds_will_attend_pnc**	This parameter is scaled at intialisation of the simulation. The value shown here represents the known 'target' odds that a mother will receive postnatal care<br><br>Once scaled, as the simulation runs this parameter is the odds that a mother will receive postnatal care if she is younger than 30, lives in an urban setting, is in wealth quintile five, has attended less than four ANC visits, did not deliver via caesarean, did not deliver in a health facility and has given birth less than four times<br><br>The parameters below starting with "or_pnc" refer to the effect on the odds of receiving postnatal care.	0.9	The proportion of women in Malawi who received postnatal care following birth was sourced from the 2010 and 2015 Malawian DHS surveys sets (11,12). These values, 50% of mothers in 2010 and 48% of mothers in 2015, represent the target coverage of PNC for mothers in the model. Due to similarity between the proportion of women receiving PNC in both surveys a single parameter value has been used here as the rate is assumed to be constant over time. The value of this parameter was therefore calculated as coverage/ (1-coverage). The value has then been manipulated to ensure calibration given the distribution of caesarean delivery is not known when the parameter is scaled.
**or_pnc_age_30_35**	The effect of a postnatal woman being aged between 29 and 36 years old compared to 20 years or younger	1.75	Sourced directly from Khaki et al. (2) who developed a logistic regression model to explore the association of key sociodemographic and healthcare variables on the odds of PNC attendance in Malawi using 2015 DHS data set.
**or_pnc_age_>35**	The effect of a postnatal woman being over the age of 35 years old compared to 20 years or younger	1.86	See **or_pnc_age_30_35**.

**or_pnc_rural**	The effect of a postnatal woman living in a rural setting compared to an urban setting	0.55	See **or_pnc_age_30_35**.
**or_pnc_wealth_level_1**	The effect of a postnatal woman being in the first wealth quintile compared to the fifth wealth quintile	0.72	See **or_pnc_age_30_35**.
**or_pnc_anc4+**	The effect of a postnatal woman having attended four or more antenatal care visits compared to less than four	1.2	See **or_pnc_age_30_35**
**or_pnc_caesarean_delivery**	The effect of a postnatal woman having delivered via CS compared to another mode of birth	1.93	See **or_pnc_age_30_35**.
**or_pnc_facility_delivery**	The effect of a postnatal woman having given birth in a health facility compared to at home	1.91	See **or_pnc_age_30_35**.
**or_pnc_parity_>4**	The effect of a postnatal woman having given birth four or more times compared to less than four	0.03	See **or_pnc_age_30_35**.
**prob_careseeking_for_complication_pn / prob_care_seeking_postnatal_emergency**	The probability that a mother experiencing postnatal complications will seek care	0.782	Sourced directly from Chinkhumba et al. (36)
**prob_timings_pnc**	The probabilities that a newly postnatal woman who will receive postnatal care will receive care within 48 hours from birth or after 48 hours from birth.	[0.85, 0.15] / [0.88, 0.12]	The percentage of women who receive PNC that receive this care within or following forty-eight hours after their delivery was calculated from the Malawian DHS surveys (11,12).

**probs_of_attending_pn_event_by_day**	The probabilities that the event PostnatalWeekOneMaternalEvent will occur respectively on the second, third, fourth, fifth, sixth day of the postnatal period	[0.4, 0.3, 0.2, 0.05, 0.05]	The PostnatalWeekOneMaternalEvent applies risk of complication onset in the first week after birth. This parameter determines which day of the first week the event will occur to prevent clustering. This is weighted towards the earlier days as evidence suggests postnatal morbidity is most common in the first few days after birth (70).

** Where two values (or sets of values) are provided the first set is applied from 2010-2014 and the second set from 2015 onwards for a given simulation run (§1.2.1.1)*

Table S33 – Parameters relating to care seeking for maternal PNC

2.6.1.2 Intervention delivery and quality

In this section an overview of the interventions/services which are available to individuals during this time is provided. Interventions have been sourced primarily from the NRHSDG (66) and the most recent obstetrics and gynaecology guidelines (32) which provide detail on the recommended clinical care for complications which may occur in the postpartum period. Table S33 details the required consumables and the modelled effects for each intervention.

**Intervention**<br>*(Modelled indication)*	**Consumables**	**Modelled treatment effect and source**
Screening and preventative interventions:		
**Screening for HIV**<br>*(All women))*	*Described previously in Table S24.*	*Described previously in Table S24.*
**Screening for depression**<br>*(All women)*	*Described previously in Table S24.*	*Described previously in Table S24.*
**Daily iron and folic acid supplementation**<br>*(All women)*	*Described previously in Table S24.*	If treatment is delivered, and the individual will be adherent, then the individual's weekly risk of developing postnatal anaemia is reduced (RR 0.30 (42)).
Curative interventions:		
**Uterotonics**<br>*(Postpartum haemorrhage due to atonic uterus)*	<u>*Required:*</u> Oxytocin, injection, 10 IU in 1 ml ampoule<br><br><u>Optional</u>: Misoprostol, tablet, 200 mcg, Pethidine, 50 mg/ml, 2 ml ampoule, Oxygen, 1000 litres, primarily with oxygen cylinders, Cannula iv, Urine bag 2000 ml, Foley catheter, Giving set, Disposables gloves, Complete blood count	If haemostasis is achieved, uterotonic delivery resets PPH property averting application of the risk of death (71). See §3.1.13 for more detail.
**Manual removal of retained placenta**<br>*(Postpartum haemorrhage due to retained placenta)*	<u>*Required:*</u> N/A<br><br><u>Optional</u>: Misoprostol, tablet, 200 mcg, Pethidine, 50 mg/ml, 2 ml ampoule, Oxygen, 1000 litres, primarily with oxygen cylinders, Cannula iv, Urine bag 2000 ml, Foley catheter, Giving set, Disposables gloves, Complete blood count	If successful uterotonic delivery resets PPH property averting application of the risk of death (37). See §3.1.13 for more detail.

**Blood transfusion**<br>(Postpartum haemorrhage)	*Described previously in Table S27.*	Reduces the risk of death from postpartum haemorrhage (RR 0.4 (37)).
**Surgical management of postpartum haemorrhage**<br>(Postpartum haemorrhage)	*Required:* Halothane (fluothane), Ceftriaxone 1g, Metronidazole 200mg<br><br>*Optional:* Cannula iv, Paracetamol, Diclofenac injection, Pethidine, 50 mg/ml, 2 ml ampoule', Foley catheter, Urine bag 2000ml, Hartmann's solution 1000 ml, Sodium chloride injectable solution 0.9 % 500 ml, Giving set	Reduces the risk of death from postpartum haemorrhage (RR 0.25 (37)).
**Intravenous antihypertensives**<br>*(Severe pre-eclampsia, Eclampsia, Severe gestational hypertension)*	*Described previously in Table S27.*	*Described previously in Table S27.*
**Anticonvulsants plus case management**<br>(*Severe pre-eclampsia, Eclampsia)*	*Described previously in Table S27.*	*Described previously in Table S27.*
**Maternal sepsis case management**<br>*(Postnatal sepsis)*	*Described previously in Table S30.*	*Described previously in Table S30.*

Table S34- Interventions available to mothers during PNC

2.6.2 Neonatal postnatal care

2.6.2.1 Care seeking for neonatal postnatal care

Immediately following delivery, it is determined if a newborn will receive PNC. For newborns born in a health facility, **prob_pnc_check_newborn** is the probability they will receive PNC in the absence of complications, whilst **prob_care_seeking_for_complication** is the probability they will receive PNC given complications. The parameter **prob_pnc_timing_newborn** stores the probabilities that PNC will occur within 48 hours from birth or after. Those predicted to receive PNC within 48 hours will receive care, whilst those predicted to receive PNC after 48 hours of life are randomly scheduled using a uniform distribution to undergo this PNC event before the end of the neonatal period.

If a neonate develops a complication later in the neonatal period, they may receive initial or further postnatal care (depending on the initial scheduling) with the parameter **prob_care_seeking_postnatal_emergency_neonate** representing the probability of care seeking. Table S34 contains the relevant parameters governing this process.

Parameter Name**	Description	Value	Source and/or relevant calculation
**prob_pnc_check_newborn**	The probability that a newborn who is not experiencing complications will receive postnatal care during the neonatal period.	0.6	Sourced directly from the Malawi DHS 2015-16 (11) in which the coverage of PNC is reported as 60%.
**prob_pnc_timing_newborn**	The probability that a newborn who is not experiencing complications and will receive postnatal care will receive that care within 48 hours of birth or after.	[0.97, 0.03]	Timing of first PNC visit is captured within the Malawi DHS 2015 (11). The proportion of neonates who received PNC that received care within 48 hours, the first figure, and after 48 hours, the second figure is calculated from this data source.
**prob_care_seeking_for_complication / prob_care_seeking_postnatal_emergency_neonate**	The probability that a newborn experiencing neonatal sepsis will receive emergency postnatal care.	0.782	Sourced directly from Chinkhumba et al. (36).

*** If two names are provided for the same parameter this means the name varies by python file. Both are provided to ensure clarity when reviewing any code.*

Table S35 – Parameters representing care seeking following neonatal complications

5.6.2.2 Intervention Delivery

Table S35 lists the interventions available to neonates as part of PNC. During the first PNC visit newborns are administered essential newborn care including vitamin k administration, eye care and high-risk newborns are screened for HIV. Any low-birth-weight babies receiving early PNC may initiate Kangaroo Mother Care (KMC). The parameter **prob_kmc_available** in Table S36 represents the probability KMC will be initiated for a given low-birth weight newborn given the predicted availability of such services in Malawi. In addition, newborns who have developed either early or late onset sepsis may receive treatment, either intravenous antibiotics or intravenous antibiotics plus full supportive care, dependent on the facility level of the PNC appointment.

The administration and effect of essential newborn Immunisations Is managed by the Extended Programme of Immunisation module and as such, is not described here.

**Intervention**<br>*(Modelled indication)*	**Consumables**	**Modelled treatment effect and source**
**Tetracycline eye drops**<br>*(All neonates)*	*Required*: Tetracycline eye ointment 1% (5mg tube)	Due to lacking evidence of effect on neonatal outcomes that are included in the model this intervention does not have a modelled effect. It is included to map consumable use accurately.
**Vitamin K prophylaxis**<br>*(All neonates)*	*Required*: Phytonadione (1mg/ml)<br><br>*Optional*: Cannula IV, Giving set, Disposable gloves	Due to lacking evidence of effect on neonatal outcomes that are included in the model this intervention does not have a modelled effect. It is included to map consumable use accurately.
**HIV screening**<br>*(All neonates)*	*N/A*	Whilst scheduling for this intervention occurs in PNC, screening and treatment for HIV is managed by the HIV module (see https://www.tlomodel.org/writeups.html). In short, if HIV is detected the neonate is commenced on antiretroviral therapy. HIV positive neonates are then screened for tuberculosis.
**Kangaroo mother care**<br>*(Preterm neonates)*	*N/A*	Reduces the risk of death from prematurity (RR 0.49 (72)).
**Injectable antibiotics**<br>(Neonatal sepsis)	*Required:* Benzylpenicillin 1g, Gentamicin 40mg/ml<br><br>Optional: Cannula IV, Giving set, Disposable gloves	Reduces the risk of death from neonatal sepsis (RR 0.35 (73))

**Full supportive care**<br>(Neonatal sepsis)	*Required:* Benzylpenicillin 1g, Gentamicin 40mg/ml, Oxygen, 1000 litres, primarily with oxygen cylinders<br><br>Optional: Dextrose (glucose) 5%, 1000ml, feeding tube, Cannula iv, Giving set iv, Disposable gloves	Reduces the risk of death from neonatal sepsis (RR 0.2 (73))

Table S36 – Interventions available to newborns during postnatal care

Parameter Name	Description	Value	Source and/or relevant calculation
**prob_kmc_available**	The probability that a low-birth-weight neonate will receive KMC if early postnatal care is initiated.	0.62	Sourced directly from Chavula et al. (74) who analysed data on KMC service availability in all hospitals in Malawi collected as part of the 2014 EmONC needs assessment. They report that 62% of hospitals met the most basic definition of readiness to deliver KMC.

Table S37 – Parameter representing the probability of KMC for a low-birth-weight newborn

2.6.3 Obstetric fistula case management

In addition to routine PNC, women who develop obstetric fistula following labour may choose to seek care for management of this condition. Probability of care seeking is calculated at the point of fistula onset via a multiplicative linear model using parameters sourced directly from a pooled analysis of data from women experiencing fistula across multiple countries in SSA conducted by Gebremedhin et al. (75) described in Table S37. Treatment is scheduled on a random date between days seven and forty-two after birth for those seeking care and currently treatment is assumed to be effective for all women who attend. Treatment within the module simply resets the maternal variable signifying fistula which in turn removes associated disability weight. For simplicity, there are no required consumables for the delivery of this treatment, yet consumables are logged when treatment is delivered.

Parameter Name	Description	Value*	Source and/or relevant calculation
**odds_care_seeking_fistula_repair**	The odds that a woman aged 20 or more, with more than primary education who has developed an obstetric fistula will seek care for treatment	1.5	Gebremedhin et al.(75) conducted an analysis of DHS survey data from across SSA to estimate care seeking for fistula alongside the effect of sociodemographic variables on odds of care seeking. They reported that 60.3% (95% CI: 56.9-63.6%) of women with fistula sought care for repair. As such, the odds of fistula repair shown here are calculated as 0.60/ (1- 0.60).
**aor_cs_fistula_age_15_19**	The effect of a mother being 15-19 years old compared to 35 years and above on the odds that she will seek care for fistula repair	0.31	See **odds_care_seeking_fistula_repair**. Sourced directly from Gebremedhin et al. (75).
**aor_cs_fistula_age_lowest_education**	The effect of a mother having primary education or lower compared to secondary education or higher on the odds that she will seek care for fistula repair	0.69	Sourced directly from Gebremedhin et al.(75).

Table S38 – Parameters relating to care seeking following obstetric fistula

Treatment	Logged consumables	Modelled effect and source
Obstetric fistula case management	*Optional:* Halothane (fluothane), Ceftriaxone 1g, Metronidazole 200mg, Scalpel blade, Cannula iv, Paracetamol, Diclofenac injection, Pethidine, 50 mg/ml, 2 ml ampoule', Foley catheter, Urine bag 2000ml, Hartmann's solution 1000 ml, Sodium chloride injectable solution 0.9 % 500 ml, Giving set	Treatment is assumed to be 100% effective in repairing an obstetric fistula. Treated women therefore do not accrue any monthly disability weight associated with obstetric fistula.

Table S39 – Details of obstetric fistula case management health system interaction within the MPHM

3 – Health condition modelling

In this section the models for each of the maternal and neonatal conditions included in the MPHM are described. For each model the condition is defined, aetiology and epidemiology are briefly outlined, present a diagrammatic representation of the model[3] is presented which is then described with supporting evidence alongside further details of treatment, followed by a table of all associated parameters and data sources. Where relevant, we highlight any simplifying assumptions which have been made during the process of model development.

3.1 Maternal complication models

3.1.1 Ectopic Pregnancy

3.1.1.1 Condition overview

Ectopic pregnancy (EP) refers to any pregnancy in which implantation of the embryo occurs outside of the uterus (76). Most commonly implantation occurs within the fallopian tube but may also occur in the cervix, abdominal cavity, or other sites (77). EP occurring within the fallopian tube, Tubal EP, is likely caused by both impairments within the embryo-tubal transport system and changes to the tubal environment leading to early implantation, however the current evidence is limited (77).

Predictors of EP which were identified in the literature from neighbouring settings to Malawi included previous pelvic inflammatory disease (PID), previous sexually transmitted infection (STI) and maternal smoking (78–81). At the time of MPHM design PID and STI are not modelled in the TLO framework meaning we were unable to model a relationship between these conditions, however if included in future iterations of the model, then these relationships can be incorporated. In addition, whilst maternal smoking is modelled, the prevalence is very low in women in Malawi (0.6% (11)) and for simplicity this was also not included as a predictor of EP in the model.

---

[3] Within each figure light blue boxes represent the natural history in the absence of treatment and light green boxes represent treatment. Bold text represents model parameters.

In many instances untreated EP can lead to significant morbidity and mortality in mothers and is a leading cause of death during the first trimester of pregnancy (82). In tubal EP, death occurs via rupture of the fallopian tube leading to haemorrhage and hypovolaemic shock (76,83,84). In SSA effective diagnosis and management of EP is hampered by limited use or availability of diagnostic ultrasound and late-presentation to health-service often after fallopian tube rupture, leading to shock (84).

Globally the incidence of ectopic pregnancy appears to vary considerably between settings and populations but is likely around 1-2% of all pregnancies but may be as high as 5% in women using assisted reproductive technology to conceive (665). Reliable estimates of the incidence of ectopic pregnancy in Malawi are lacking and therefore the incidence rate used in the model was taken from Panelli et al. (76) as shown in the table below.

3.1.1.2 Model

Figure S7 describes the model of ectopic pregnancy and the Table S39 describes the relevant parameters.

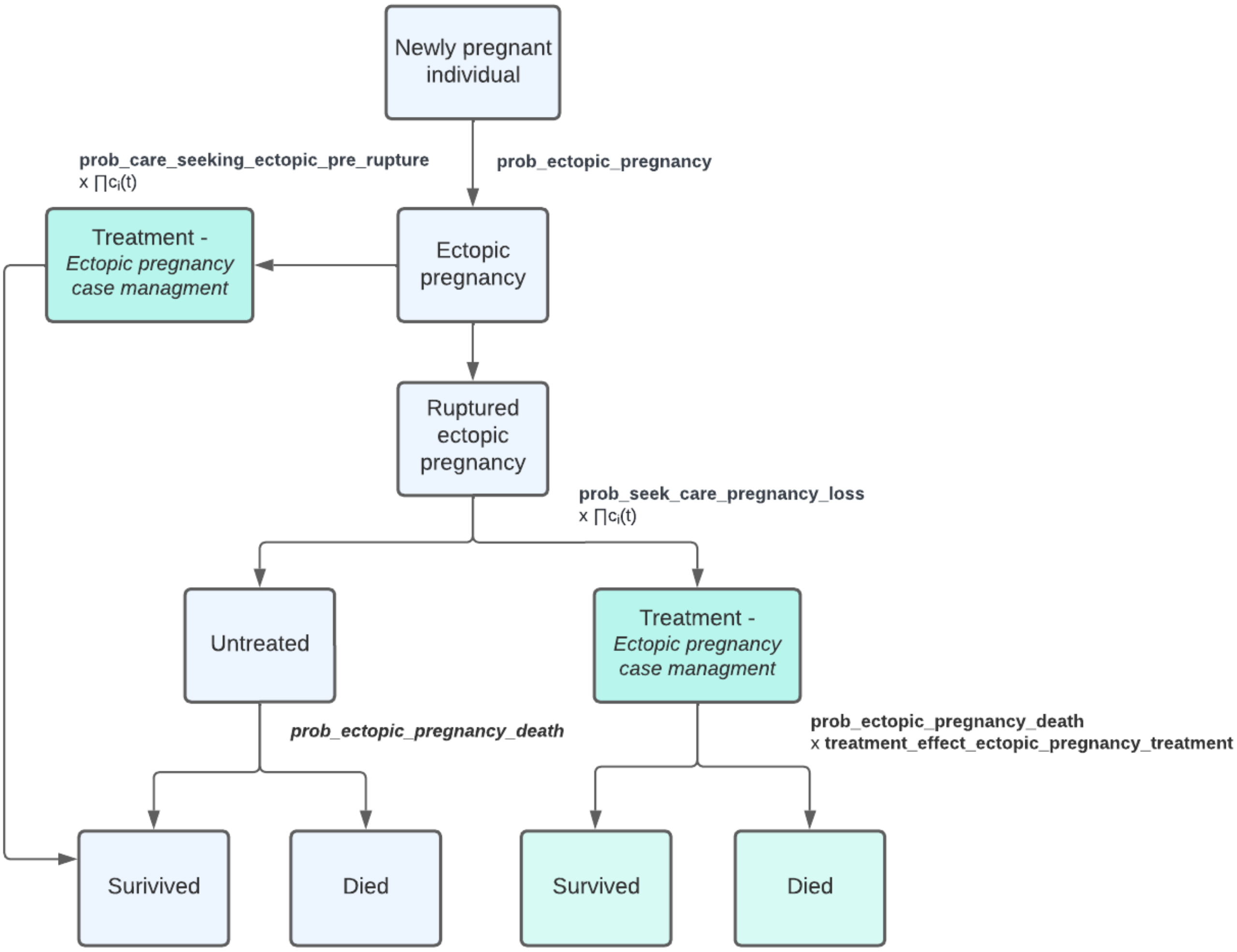

Figure S7 – Model of ectopic pregnancy
Diagrammatic representation of the model of ectopic pregnancy. Light blue represents the model's natural history without treatment whilst teal represents treatment pathways. Parameters representing progression through model states are shown here.

A probability of EP is applied to all newly pregnant women at initiation of pregnancy, **prob_ectopic_pregnancy**, as seen in see Figure S7. This probability was calculated from estimated incidence of EP reported by Panelli et al. (76) leading to a mean rate of 10 EP per 1000 pregnancies within the population. For women who experience EP, a probability of care seeking prior to rupture, **prob_care_seeking_ectopic_pre_rupture**, is applied at

between 6-8 weeks GA with this time step chosen to mimic probable onset of symptom triggering care seeking after discussion within clinical experts (54).

Failure to receive treatment leads to rupture at between 8-10 weeks GA (85). Probability of care seeking for treatment following rupture is assumed to be equal to the probability of care seeking following other abortive outcomes, **prob_seek_care_pregnancy_loss**, probability of death is applied to all individuals who experience rupture, **prob_ectopic_pregnancy_death**.

We have chosen to exclude the possibility of non-tubal ectopic pregnancies (incidence 5-8% of all ectopic pregnancies (86)) due to low incidence within an already rare event. Non-tubal ectopic pregnancies can progress to a greater gestation and in some, very rare cases, have led to live births (87). As such it is assumed all ectopic pregnancies in the model end in abortion of the foetus and end of viable pregnancy. Additionally, it is assumed that ectopic pregnancy and its associated complications do not affect the future probability of an individual in the data frame becoming pregnant again due to evidence that future fertility remains largely unaffected following treatment (88,89).

3.1.1.2.1 Treatment

As evident in Figure S7, the probability of treatment before rupture is equal to the product of **prob_care_seeking_ectopic_pre_rupture** and the probability of availability of consumables required to deliver the necessary treatment (Table S21) defined as $\prod c_{i(t)}$. If treatment is delivered successfully prior to rupture, it is assumed that, because rupture is on the causal pathway to death, risk of death is not applied to those successfully treated at this stage. Otherwise, successful receipt of treatment following rupture leads to a reduced risk of death because of treatment, **treatment_effect_ectopic_pregnancy_treatment.**

3.1.1.3 Data sources and parameters

Parameter Name	Description	Value*	Notes on data sources and relevant calculations
**prob_ectopic_pregnancy**	The per-pregnancy risk of ectopic pregnancy	0.01	We were unable to identify a reliable data source for the rate of EP within the population in Malawi. As such a conservative rate of EP of 10 per 1000 pregnancies (1%) as reported by Panelli et al. (76) is assumed.
**prob_care_seeking_ectopic_pre_rupture**	The probability a woman with experiencing ectopic pregnancy will seek care prior to rupture	0.08	Flores et al. (84) conducted a systematic review of SSA studies evaluating ultrasound diagnosis of EP in which the authors estimate the proportion of EP cases receiving treatment that were ruptured on admission as 92.3%. It is therefore assumed that only 8% of women seek care prior to rupture and used this value to represent the probability of care seeking prior to rupture in the model.
**prob_ectopic_pregnancy_death**	The probability of death from a ruptured EP	0.02 / 0.012	The model has been calibrated to both the reported MMR in 2010 and 2015 sourced from the Malawian DHS surveys in those years (11,12) and the proportion of total direct maternal deaths by cause soured from the 2010 and 2015 Malawian EmONC needs assessments (33,34).<br><br>As such untreated case fatality parameters have been estimated through the process of calibration to ensure that the model replicates both the assumed MMR and the proportion of deaths by cause. Additional detail is provided in §4.

**treatment_effect_ectopic_pregnancy_treatment**	The effect of treatment of ruptured EP on risk of death from EP	0.1	Sourced directly from Pollard et al. (37) in which the authors estimate the effect of ectopic pregnancy case management on maternal death due to ectopic pregnancy via a Delphi survey of relevant experts. Effectiveness is reported as 90%.

** Where two values (or sets of values) are provided the first set is applied from 2010-2014 and the second set from 2015 onwards for a given simulation run (§1.2.1.1)*

Table S40 – Parameters of the ectopic pregnancy model

3.1.2 Spontaneous and induced abortion

3.1.2.1 Condition overview

3.1.2.1.1 Spontaneous abortion

Spontaneous abortion (SA), often referred to as miscarriage, is defined as any pregnancy loss, excluding induced abortion or ectopic pregnancy, in the first 28 weeks of pregnancy, whilst pregnancy loss following 28 weeks is classified as stillbirth (90). Despite the regularity in which women experience SA, the aetiology of primary and recurrent SA is not well understood and remains unknown in most cases (91). However commonly cited predictors of SA include maternal age and previous early pregnancy loss (92). These factors were also identified during review of the literature and are included in the model (93) as shown below. SA is a significant driver of maternal morbidity due to potentially long-lasting psychological impact on mothers (94) and in some settings is also associated with several possibly life-threatening complications including maternal sepsis and haemorrhage (95,96).

SA is a common outcome of pregnancy with an estimated 15% of all global pregnancies ending spontaneously (97). However, there is likely considerable uncertainty around this estimate as the population level incidence of SA is not routinely collected in many countries across the globe (97). In addition, due to similarities in presentation at health services it is often difficult to distinguish between spontaneous or induced abortion, especially in countries with restrictive abortion laws where women may be less likely to disclose, meaning estimates of incidence and outcomes are often mixed under a heading of abortion (98). Polis et al. (98) estimate this total number of SA cases in Malawi in 2015 leading to an approximate rate of 153 spontaneous abortions per 1000 pregnancies as discussed in Table S40.

3.1.2.1.2 Induced abortion

Induced abortion (IA) is defined as “the termination of pregnancy using drugs or surgical intervention after implantation and before the embryo or foetus has become independently viable” (99). IA, when performed by an appropriate method, a trained healthcare professional in an appropriately equipped healthcare facility and at the correct GA, is a safe procedure of which the provision is essential to women’s rights to sexual and reproductive

healthcare (100). However, in many settings, access to safe IA is restricted to circumstances in which the life of the mother is at risk from the continuation of the pregnancy (101). This has led to widespread practice of unsafe IA which is associated with considerable morbidity and mortality in many settings and in 2014 was estimated to constitute as many as 45.1% of all abortions globally, with the greatest density of these situated in the countries with the most restrictive abortion laws (102).

Despite recent shifts in political priority towards liberalising abortion laws (103), Malawi is one such country in which access to safe IA is restricted to circumstances where it is essential to preserve a woman's life due to laws introduced during colonisation by the British Empire (104). As such, the incidence of complications associated with IA is high in Malawi and in 2015 an estimated 51, 693 women who underwent IA required post-abortion care at a health facility (98). Facility based studies in Malawi report a case fatality rate of 387 deaths per 100,000 post abortion care procedures mainly due to sepsis and blood loss (1).

Several contemporary studies have identified predictors for IA in sub-Saharan Africa. Commonly reported factors included marital status, maternal age, maternal education, intimate partner violence within the current relationship and unintended pregnancy (106–112). Through the review process during this study it was deemed that most of these characteristics are likely related to IA through unintended pregnancy. However, at the time of writing the proportion of unintended pregnancies within the model, generated via the contraception module, does not match the proportion estimated by Polis et al. (98), the study from which IA incidence in the model is sourced. This is because in the contraception model only women who experience contraception failure experience an unintended pregnancy. Due to this, a fixed monthly risk of IA has been applied to all women.

Globally there is considerable heterogeneity in the national and regional rates of both unintended pregnancy and IA with the greatest range in estimated incidence rates found in SSA largely due to lacking data (113). In this region Bearak et al. (113) estimated that in 2015-2019, 91 (80% UI 86 to 97) pregnancies per 1000 women of reproductive age are estimated to be unintended and 34 (80% UI 29 to 38) per 1000 end in IA. Nationally

representative survey data from Malawi estimated a national rate of 38 abortions (95% CI: 32 to 43) per 1000 women aged 15-49 in 2015 with 53% of all pregnancies estimated to be unintended, 30% of which end in abortion (98).

3.1.2.2 Models

3.1.2.2.1 Spontaneous abortion

Figure S8 describes the model of spontaneous abortion and Table S40 contains the relevant parameters.

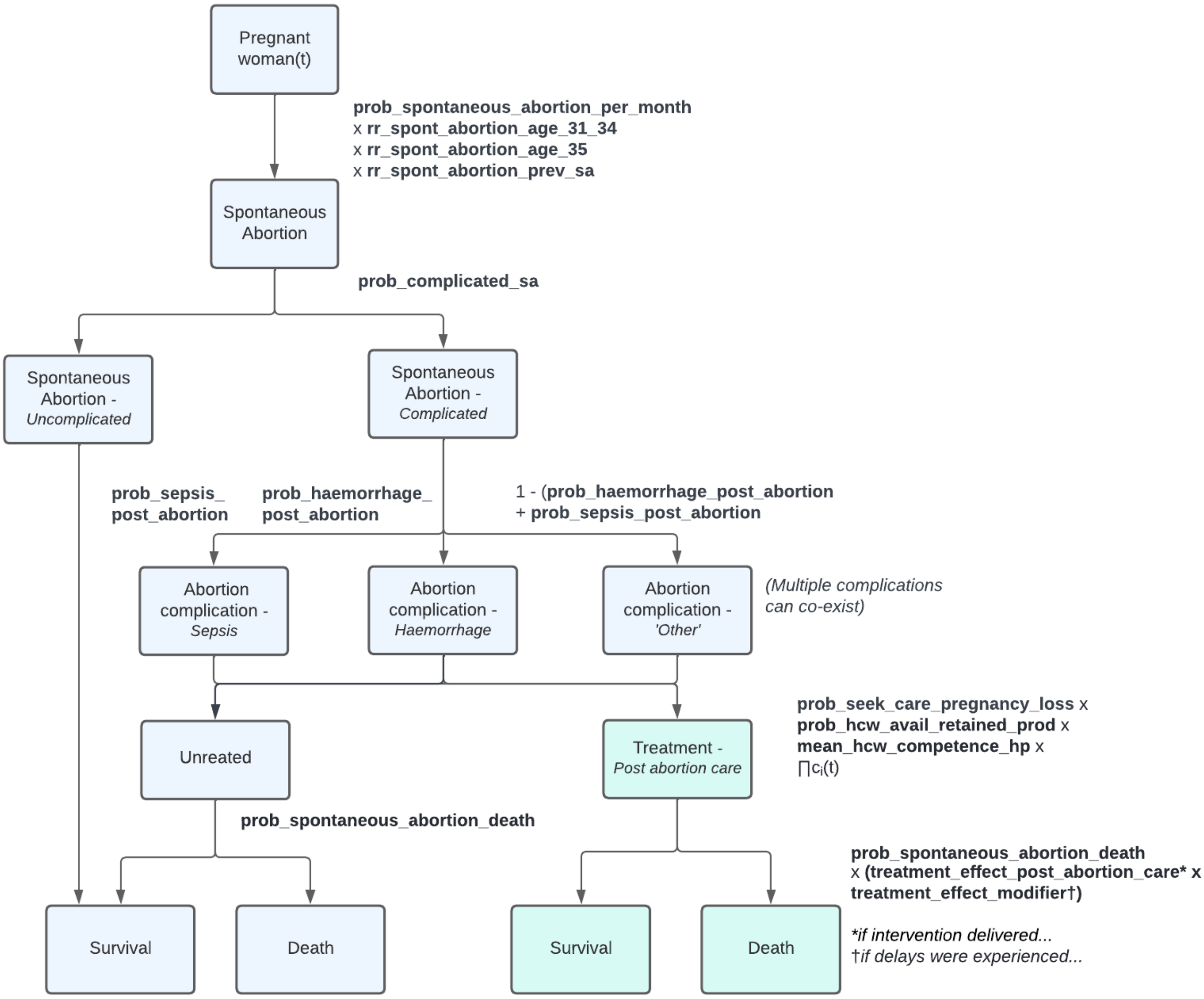

Figure S8 – Model of spontaneous abortion
Diagrammatic representation of the model of spontaneous abortion. Light blue represents the model's natural history without treatment whilst teal represents treatment pathways. Parameters representing progression through model states are shown here.

A probability of SA is applied to all pregnant women on the final week of each month of pregnancy up until 28 weeks' GA. This probability for a given month, $Y_{(t)}$ is calculated via the following multiplicative model:

$$Y_{(t)} = \textbf{prob\_spontaneous\_abortion\_per\_month}_{(t)} * (age\_years\_31\_34 * \textbf{rr\_spont\_abortion\_age\_31\_34}) * (age\_years\_35\_plus * \textbf{rr\_spont\_abortion\_age\_35}) * (ps\_prev\_spont\_abortion * \textbf{rr\_spont\_abortion\_prev\_sa}) \quad (8)$$

Here, **prob_spontaneous_abortion_per_month**$_{(t)}$ is the intercept value of the multiplicative model, the risk of SA at time*(t)* for a woman for whom there is no effect of predictor variables (i.e. she is younger than 31 years and has not had a previous SA) whilst **rr_spont_abortion_age_31_ 34**, **rr_spont_abortion_age_35**, **rr_spont_abortion_prev_sa** are the effects of being aged 31 to 34 years, being aged 35 years and older and having previously experienced SA on risk of SA (93).

The total rate of SA In the model, 153 SA per 1000 pregnancies, is derived from a Malawian study estimating the incidence of induced abortion in Malawi in which the authors also estimate the number of SA and pregnancies in Malawi in 2015 (98) as described further in Table S39.

Following onset, a fixed probability of developing complications associated with spontaneous abortion is applied, **prob_complicated_sa**. As evident in Figure S8, the complement of this probability determines an uncomplicated pregnancy loss in which women experience no adverse complications and simply stop being pregnant in the model. For those individuals for which this pregnancy loss will be complicated, the type or types of complications they experience are determined include sepsis, haemorrhage or ‘other’. Risk of haemorrhage, **prob_haemorrhage_post_abortion**, and risk of sepsis, **prob_sepsis_post_abortion**, are applied sequentially with the model allowing for co-occurrence of both complications within a single individual. If it is determined neither of these complications will occur the individual is assumed to be experiencing ‘other’ complications. These complications were selected after review of studies of evaluating common clinical outcomes in women presenting for care in Malawi and other LMIC settings (105,114). Parameter **prob_seek_care_pregnancy_loss** is the probability that an individual will seek care following a complicated SA.

3.1.2.2.2 Induced abortion

Figure S9 describes the model of induced abortion and Table S40 contains the relevant parameters.

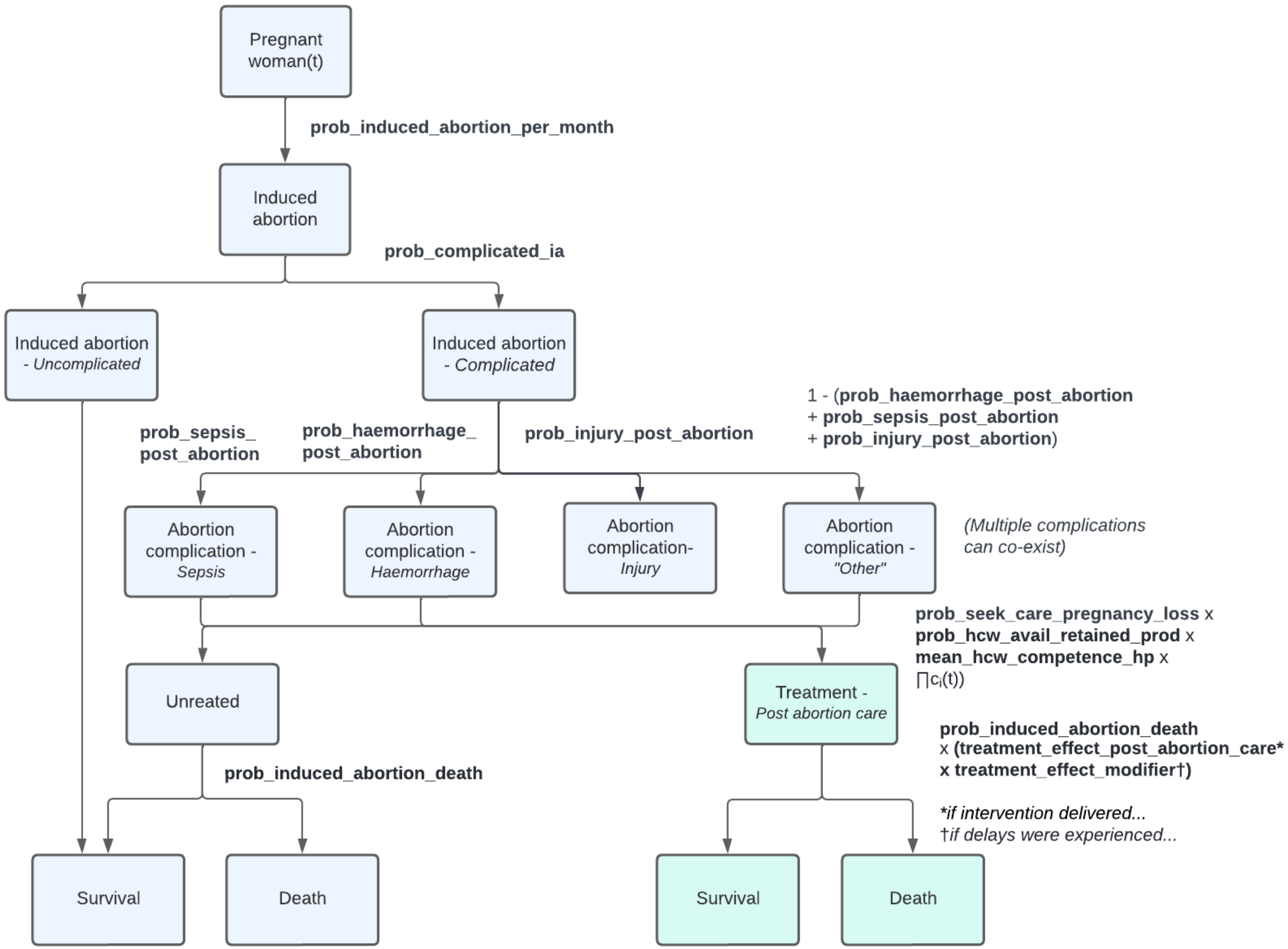

Figure S9 – Model of induced abortion
Diagrammatic representation of the model of induced abortion. Light blue represents the model's natural history without treatment whilst teal represents treatment pathways. Parameters representing progression through model states are shown here.

A monthly risk of IA, **prob_induced_abortion_per_month**, is applied to all pregnant women on the final week of each month of pregnancy up until 28 weeks. It is assumed that IA may occur from week 8 in line with data from the US which suggests that mean GA of pregnancy awareness is around 5.5 weeks (115), therefore the 8-week time step was chosen to begin risk application for IA to account for this and time for decision making relating to termination. As with SA, IA parameter determines if complications occur, **prob_complicated_ia,** after which probabilities determining complication type are applied, including risk of having experienced injury post induced abortion, **prob_injury_post_abortion**. Parameter **prob_seek_care_pregnancy_loss** is the probability that an individual will seek care following a complicated IA.

Whilst most abortions conducted in Malawi occur outside of the formal health system, given they are illegal, there is likely a gradient in abortion safety depending on the method and practitioner given that not all illegal abortions are as unsafe as others. This thinking is evident in recent studies estimating global abortion safety, where researchers have categorised abortions as safe, less safe, and least safe (102). For simplicity we have opted to apply a fixed risk of complication associated with induced abortion instead of grading abortions by assumed safety, as described above.

3.1.2.2.3 Treatment

As evident in Figures S8 and S9, for those women who seek care following complication onset, the probability of intervention delivery is dependent by the EmONC quality parameters defined in §2.3, in addition to the availability of consumables. Listed. As the clinical interventions defined in Malawian clinical guidelines are delivered dependent on presenting complications (e.g., signs of infection) (32) it is assumed that care can only be delivered if the consumables for complication specific treatment are available. For example, women who are post-abortion, seek care and are experiencing sepsis will require antibiotic treatment, if available, to benefit from care. Whilst this approach is taken to capture the probability of treatment delivery related to specific type of complication, we have opted to utilise a fixed treatment effect for successful PAC, **treatment_effect_post_abortion_care**, regardless of complication type due to limited estimates of complication specific treatment effects in the context of SA or IA.

3.1.2.3 Data sources and parameters

Parameter Name	Description	Value*	Notes on data sources and relevant calculations
**prob_spontaneous_abortion_per_month**	This parameter is scaled at intialisation of the simulation to account the distribution of maternal ages and proportion of women who have previously experienced spontaneous abortion at baseline. Once scaled, as the simulation runs this parameter is the probabilities that a pregnant woman younger than 31 years who has not experienced a SA previously will experience a SA in month 1, 2, 3, 4 or 5 of pregnancy	[0.025, 0.037, 0.05, 0.037, 0.025]	The assumed rate of SA in the model is sourced from Polis et al (98) in which the authors report the estimated the total number of SA, IA, and pregnancies in 2015 in Malawi via a nationally representative survey of health facilities. The rate, is calculated as the total number of SA divided by total pregnancies (x 1000), is equal to 153 SA per 1000 pregnancies.<br><br>In line with evidence from a prospective study conducted in Kenya evaluating weekly SA rates in a pregnant cohort (93) The probabilities within this parameter have been adapted to replicate a greater risk of SA in month 3 of pregnancy.
**rr_spont_abortion_age_31_ 34**	The effect of a pregnant woman being aged between 29 and 35 years compared to 15-20 years on the monthly risk of SA	2.31	Sourced directly from Dellicour et al. (93) in which the authors report weekly miscarriage rates for a cohort of pregnancies (1134 total) in rural Kenya alongside the effect of sociodemographic and health variables on overall risk of miscarriage. Similar data was not available from Malawi.
**rr_spont_abortion_age_35**	The effect of a pregnant woman being aged 35 years or older compared to 15-20 years on the monthly risk of SA	4	See **rr_spont_abortion_age_31_ 34.**
**rr_spont_abortion_prev_sa**	The effect of a pregnant woman having previously experienced a SA on the monthly risk of SA	2.23	See **rr_spont_abortion_age_31_ 34.**

**prob_complicated_sa**	The probability that a woman who experiences a SA will experience any complications	0.11	See **prob_spontaneous_abortion_per_month**. Polis et al (98) also estimated the total number of spontaneous abortion cases requiring treatment in Malawi in 2015. To calculate this parameter, the total cases of SA was divided by the total cases requiring treatment to arrive at 11.2% risk of requiring treatment following SA. Whilst this is unlikely to represent the true risk of complications post SA, as an unknown number of women will not have sought care, it is assumed to be a suitable proxy within the model.
**prob_haemorrhage_post_abortion**	The probability that a woman experiencing a complicated SA will experience a haemorrhage	0.23	We were unable to identify an estimate for the proportion of women who experience a haemorrhage as a complication of abortion in Malawi. As such an estimate from Calvert et al. (114) is utilised who estimated the pooled prevalence of haemorrhage in women with abortion (both induced and spontaneous) related hospital admissions in settings with limited access to abortion via a systematic review. The authors report this value at 23% which is assumed to be equal to the risk of bleeding post abortion however this is likely an underestimate due to missing data on women who did not seek care.
**prob_sepsis_post_abortion**	The probability that a woman experiencing a complicated SA will experience sepsis	0.137	A cross-sectional facility-based study in Malawi estimated the proportion of women receiving post abortion care who experience sepsis (113). 13.7% of participants were reported to be septic at admission which is assumed this to be equivalent to the risk of sepsis post abortion however this is likely an underestimate due to missing data on women who did not seek care.

**treatment_effect_post_abortion_care**	The effect of post abortion care on risk of death following spontaneous or induced abortion	0.2	Sourced directly from Pollard et al. (37) in which the authors estimate the effect of post abortion care on maternal death due to abortion via a Delphi survey of relevant experts. Effectiveness is reported as 80%.
**prob_spontaneous_abortion_death**	The probability that a woman will die due to complications following a SA without treatment	0.01 / 0.005	See **prob_ectopic_pregnancy_death** in Table S39.
**prob_induced_abortion_per_month**	The probability that a woman who is currently pregnant and in month 2, 3, 4 or 5 of her pregnancy	0.038	See **prob_spontaneous_abortion_per_month.** The assumed rate of IA in Malawi is also sourced from Polis et al. (98) in which the total number of IAs in Malawi in 2015 was estimated. The rate is calculated as the total number of IA divided by total pregnancies (x 1000), which gives a rate of 159 IA per 1000 pregnancies. As this rate, given the other assumptions relating to the use and availability of healthcare, led to too many deaths in the model attributed to abortion it was reduced slightly. This parameter was derived through calibration to this lower rate.
**prob_complicated_ia**	The probability that a woman who experiences an induced abortion will experience complications	0.37	See **prob_induced_abortion_per_month**. Polis et al. (98) also estimated the total number of IA requiring treatment in Malawi in 2015. To calculate this parameter, the total cases of IA is divided by the total cases requiring treatment to arrive at 37% risk of requiring treatment following IA. Whilst this is not likely to accurately represent the true risk of complications post IA, as an unknown number of women will not have sought care, it is assumed to be a suitable proxy within the model.

**prob_injury_post_abortion**	The probability that a woman experiencing a complicated abortion will experience an injury	0.056	We were unable to identify an estimate for the proportion of women who experience an injury as a complication of IA in Malawi. As such an estimate from Calvert et al. (114) is utilised who estimated the pooled prevalence of abortion (both induced and spontaneous) related hospital admissions secondary to injury in settings with limited access to abortion via a systematic review. The authors report this value at 5.6% which is assumed to be equal to the risk of injury post abortion however this is likely an underestimate due to missing data on women who did not seek care.
**prob_induced_abortion_death**	The probability of death from a complicated induced abortion	0.005 / 0.0025	See **prob_spontaneous_abortion_death.**

** Where two values (or sets of values) are provided the first set is applied from 2010-2014 and the second set from 2015 onwards for a given simulation run (§1.2.1.1)*

Table S41 – Parameters of the spontaneous and induced abortion models

3.1.3 Maternal Anaemia

3.1.3.1 Condition overview

In Malawi anaemia during or following pregnancy is defined as a maternal haemoglobin level (Hb) of less than 11 grams/decilitre (g/dl) at any GA (32). Several causative factors of anaemia have been identified within the literature suggesting a complex aetiology due to the co-existence of and interaction between multiple factors in one woman (116–118). In SSA and southeast Asia these factors most commonly include iron, vitamin B-12 and folate deficiencies, hookworm infection, malaria parasitaemia and untreated HIV infection (116–119). In addition to antenatal anaemia, maternal anaemia can, and often does, onset during the postnatal period or can be present in postnatal women due to pre-existing anaemia during pregnancy (120,121) and therefore it was important to incorporate postnatal anaemia within the MPHM.

Anaemia is commonly categorised by severity, which is determined by blood haemoglobin (Hb) level, with Malawian clinical guidelines defining levels of between 10.0-10.9 grams/decilitre (g/dl) as mild, 7.0-9.9 g/dl as moderate and a Hb of less than 7.0 g/dl as severe (32).Within Malawi, mild and moderate cases of anaemia are significantly more prevalent than severe cases (11), however there is limited data relating to rates of progression between stages of severity. Maternal anaemia during pregnancy is associated with both poor neonatal outcomes, including prematurity and maternal outcomes, such as maternal death (122).

Globally, anaemia during pregnancy is a relatively common condition and is particularly widespread in countries with a greater burden of communicable disease, such as malaria and HIV. The estimated prevalence during pregnancy in East Africa ranges from 23.36% to 57.1% (123). Data from Malawi suggests that the prevalence of anaemia during pregnancy may be as high as 45% with around 23% of women experiencing moderate or severe cases (11).

3.1.3.2 Model

Figures S10 and S11 describe the models of antenatal and postnatal anaemia and Table S41 describes the relevant parameters.

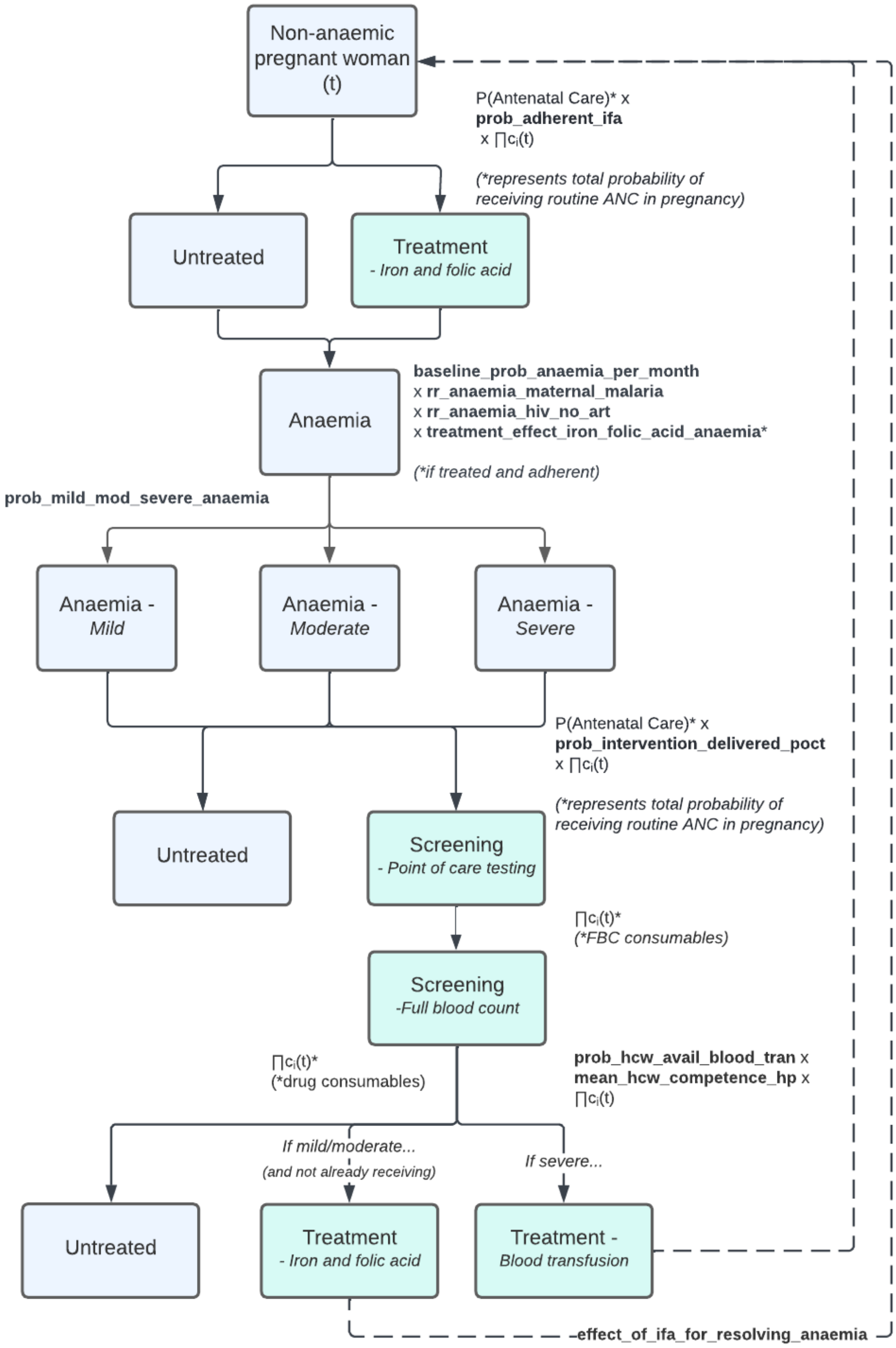

Figure S10 – Model of antenatal anaemia
Diagrammatic representation of the model of anaemia during pregnancy. Light blue represents the model's natural history without treatment whilst teal represents treatment pathways. Parameters representing progression through model states are shown here.

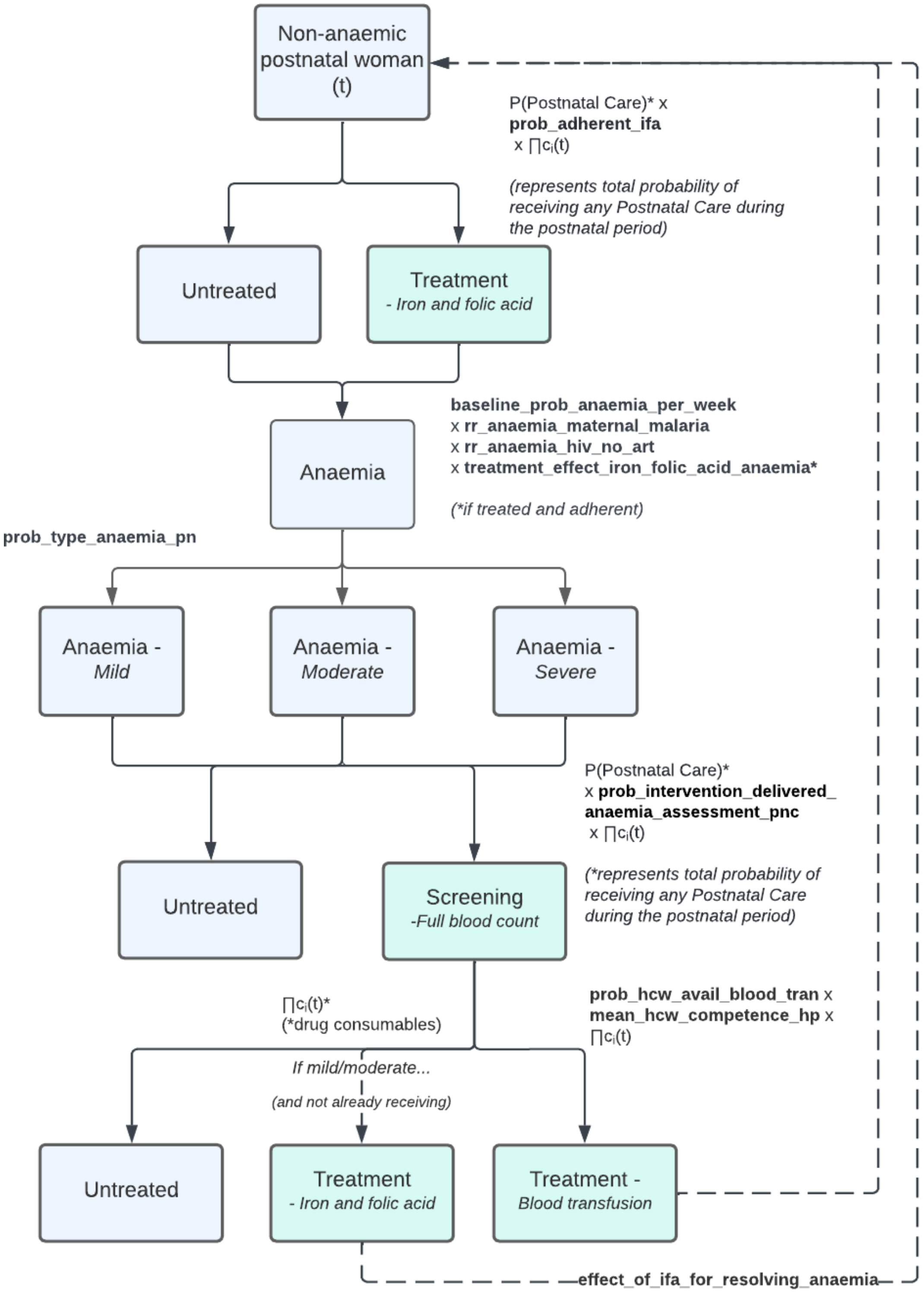

Figure S11 – Model of postnatal anaemia

Diagrammatic representation of the model of anaemia during the postnatal period. Light blue represents the model's natural history without treatment whilst teal represents treatment pathways. Parameters representing progression through model states are shown here.

A monthly risk of developing anaemia is applied to all pregnant women from conception until the end of pregnancy. Equation 9, demonstrates how monthly risk of anaemia for a given individual in the model at a given time, $Y_{(t)}$, is calculated with components in bold representing parameters and those in italics representing individual level variables[4]:

$$Y_{(t)} = \mathbf{baseline\_prob\_anaemia\_per\_month}_{(t)} * (ac\_receiving\_iron\_folic\_acid * \mathbf{treatment\_effect\_iron\_folic\_acid\_anaemia}) * (ma\_is\_infected * \mathbf{rr\_anaemia\_maternal\_malaria}) * (hiv\_inf * hiv\_art * \mathbf{rr\_anaemia\_hiv\_no\_art}) \tag{9}$$

As shown from this equation, Malaria infection (124), untreated HIV infection (125) and receipt of daily iron and folic acid (IFA) supplementation (42), as the primary predictors of anaemia within the model. Both malaria and HIV are modelled explicitly within the TLO framework whilst other possible causative agents – including hookworm infection – have not been deemed necessary to model due to their limited impact on population level morbidity and mortality. Additionally, to ensure model parsimony and due to limitations in the availability of data we opted not to build in additional modelling of micronutrient deficiencies associated with anaemia. Daily IFA supplementation is initiated through attendance of ANC as described in §3.1.3.2.1 and reduces risk of anaemia acquisition as shown by parameter **effect_of_ifa_for_resolving_anaemia.** Severity of anaemia is determined for all newly anaemic women via a probability weighted random draw, using probabilities in **prob_mild_mod_sev_anaemia**.

The same multiplicative model shown above is applied during the postnatal period, which includes the effect of IFA supplementation which should be continued postnatally. This means women are at weekly risk of developing anaemia from birth until week six postpartum. As outlined in §1 the risk of anaemia in the postnatal period is applied weekly.

---

[4] Throughout this section, where a variable name in an equation has the prefix "mni" or "nci" it is not stored in the population data frame but in an individual-level dictionary. Additionally, where an individual variable does not take a binary form, the name has been adapted to reflect the variable state which is related to the parameter.

We have made two key simplifying assumptions in the development of this model. Firstly, it is assumed that the onset of anaemia, regardless of severity, does not lead to care seeking. This decision was made in collaboration with clinical experts who deemed that symptoms for anaemia are often non-distinct, ignored or absent all together (54) therefore detection of anaemia only occurs via screening during antenatal or postnatal care. Secondly, anaemia is not modelled as being a distinct cause of maternal death. Instead, maternal anaemia of any severity is assumed to increase the risk of death in women who experience obstetric haemorrhage as described in §3.1.9 and §3.1.13. This is because anaemic women have reduced oxygen-carrying capacity meaning they are unable to tolerate similar levels of blood loss when compared to non-anaemic women and therefore they are at greater risk of mortality following haemorrhage (126).

3.1.3.2.1 Treatment

Modelled women presenting for ANC should be initiated on IFA supplementation regardless of anaemia status, as indicated in Table S41. However, data from Malawi suggests adherence to daily treatment is often low (127) possibly due to a combination of factors, such as reported incidence of side-effects and forgetfulness (128,129). Parameter **prob_adherent_ifa** is the probability a woman started on IFA will be adherent. In non-adherent women it is assumed there is no effect of treatment on risk of anaemia.

Screening for antenatal anaemia occurs in ANC contacts one and six via point of care testing and, if conducted, anaemic women are scheduled to receive further care as an inpatient[5]. Full blood count (FBC) testing is undertaken to determine anaemia severity and if mild or moderate anaemia is detected, and if the woman is not already receiving supplementation, then IFA is administered. The parameter **effect_of_ifa_for_resolving_anaemia** is the probability that initiation of this treatment will cure current anaemia with future risk of anaemia onset in pregnancy reduced via **treatment_effect_iron_folic_acid_anaemia.** Following the detection of severe anaemia individuals will undergo blood transfusion dependent on the availability of consumables and trained HCWs leading to a high probability that anaemia will be cured- **treatment_effect_blood_transfusion_anaemia.**

[5] Malawian guidelines are not clear on where treatment for confirmed anaemia is initiated (32). For now it is assumed to occur within the inpatient HSI.

Follow up FBC testing is scheduled to occur four weeks post treatment to ascertain treatment success and anaemia status with additional treatment scheduled if required. As shown in Figure S11, women presenting for PNC are also assumed to be screened for anaemia via FBC testing and will be treated as described here.

3.1.3.3 Data sources and parameters

Parameter Name**	Description	Value*	Source and/or relevant calculation
baseline_prob_anaemia_per_month	This parameter is scaled at initialisation of the simulation to account for the prevalence of HIV within the modelled population. Once scaled, as the simulation runs, this parameter is the probability that a pregnant woman without malaria, living with treated HIV infection or living without HIV and not taking daily iron and folic acid supplementation will develop anaemia per month of her pregnancy.	*4.2* / 0.12	The prevalence of anaemia at birth for pregnant women in Malawi is reported as 37.5% in the 2010 Malawi DHS survey and 45.1% in the 2015 survey (11,12). The parameters presented here were derived through calibration to these prevalences in the pregnant population given that risk of anaemia is applied monthly.
**rr_anaemia_maternal_malaria**	The effect of a pregnant woman experiencing malaria infection compared on her monthly risk of developing anaemia	1.45	Sourced directly from Ayoya et al. (124) who report the risk of anaemia given Malaria infection status for a sample of women in Mali.
**rr_anaemia_hiv_no_art**	The effect of a pregnant woman experiencing untreated HIV infection on her monthly risk of developing anaemia	4.19	Sourced directly from Adamu et al. (125) who report the results of a multinomial logistic regression model using data from 14,978 Malawian women exploring the effect of key variables on risk of anaemia.
**treatment_effect_iron_folic_acid_anaemia**	The effect of a pregnant woman receiving daily iron and folic acid supplementation on her monthly risk of developing anaemia	0.3	Sourced directly from Peña-Rosas et al. (42) who estimate that iron supplementation reduces the risk of anaemia at term by 70% (RR 0.30; 95% CI 0.19 to 0.46) via a Cochrane review of RCTs.

**prob_mild_mod_sev_anaemia / prob_type_of_anaemia_pn**	The probabilities that a woman who has developed anaemia will develop mild, moderate, or severe anaemia	[0.52, 0.475, 0.005] / [0.50, 0.46, 0.04]	See **baseline_prob_anaemia_per_month.** Both DHS surveys also disaggregate anaemia in pregnancy by severity. For example, in the 2015 survey, 22.7% of pregnant women had mild anaemia, 20.8% moderate and 1.6% severe. To calculate these values, the proportion of pregnant women with mild/moderate/severe anaemia is divided by the total proportion of pregnant women with any anaemia.
**effect_of_ifa_for_resolving_anaemia**	The probability that a woman who is mildly/moderately anaemic, and not currently receiving IFA supplementation, will no longer be anaemic after the initiation of IFA for the first time.	0.7	See **treatment_effect_iron_folic_acid_anaemia.** This treatment effect is an assumption. For the purposes of the model, because we were unable to identify a study which suitably quantified the probability that initiating IFA would resolve current anaemia, it is assumed that there is a 0.7 probability initiating IFA will correct current anaemia as Peña-Rosas et al (42) show a 70% reduction in anaemia at term with IFA.
**treatment_effect_blood_transfusion_anaemia**	The probability that a woman who is severely anaemic will no longer be anaemic after receipt of a blood transfusion	0.9	We were unable to identify a study which quantified the effect of blood transfusion on anaemia status. Due to clinical guidelines, in which blood transfusion is guided by repeat haemoglobin measurements (32), it is assumed that blood transfusion is extremely effective at resolving anaemia.

**baseline_prob_anaemia_per_week**	The probability that a postnatal woman without malaria, untreated HIV infection and daily iron and folic acid supplementation will develop anaemia per week of the postnatal period	[0.017, 0.028]	See **baseline_prob_anaemia_per_month.** Due to lacking data on postnatal anaemia in Malawi the prevalence postnatally is assumed to be the same as in pregnancy. This assumption is supported from data from an observational study in Ethiopia reporting the prevalence of postnatal anaemia as 47.16% (95% CI; 41.30–53.0) (130).<br><br>The parameters presented here were derived by calibration to this prevalence in the postnatal population.
**prob_adherent_ifa**	The probability that a woman who is provided iron and folic acid during ANC or PNC will be adherent to treatment.	0.37 / 0.34	Sourced directly from Titilayo et al (131) and Ba *et al.* (133) in which the authors extracted self-reported adherence to IFA from DHS data in Malawi.

** Where two values (or sets of values) are provided the first set is applied from 2010-2014 and the second set from 2015 onwards for a given simulation run (§1.2.1.1)*

*** If two names are provided for the same parameter this means the name varies by python file. Both are provided to ensure clarity when reviewing any code.*

Table S42 – Parameters for the anaemia model

3.1.4 Gestational diabetes

3.1.4.1 Condition overview

Gestational diabetes mellitus (GDM) is defined as "carbohydrate intolerance resulting in hyperglycaemia of variable severity with onset or first recognition during pregnancy" (133). Pregnancy is associated with a host of metabolic and endocrine changes undergone by the mother to support foetal development (134,135). This includes development of increased insulin resistance later in pregnancy to ensure a slightly elevated blood glucose which ensures sufficient transport across the placenta to the foetus (136). In some women, due to a complex relationship between genetic and environmental factors, this insulin resistance leads to hyperglycaemia classified as GDM (136).

Two systematic reviews of predictive factors associated with gestational diabetes in women in SSA reported several possible drivers of GDM including history of previous pregnancy loss, family history of diabetes and maternal overweight and obesity (137,138). From these factors, obesity was selected as the primary predictor, as seen in equation 10. Family history was not included, as mother-child dyads are the only family structure captured within the TLO model and previous pregnancy loss was not included as pregnancy loss, especially stillbirth, appears to be potentially caused by GDM and therefore is unlikely to be a cause in and of itself. Outcomes associated with GDM include stillbirth, poor preterm outcomes such as respiratory distress syndrome, and other foetal outcomes associated with maternal complications such as foetal macrosomia (139–141).

Several systematic reviews have attempted to estimate the prevalence of GDM in Africa and SSA (137,138,142) with considerable variation in results, with one study estimating a pool prevalence as high as 13.61% (95% CI: 10.99, 16.23) (138). Data from Malawi suggests that diagnostic criteria can lead to considerable variation in prevalence estimates as prevalence of GDM was determined to be 1.6% using current WHO GDM diagnostic criteria and 24% when using the International Association of the Diabetes and Pregnancy Study Groups (IAGPSG) criteria in women attending ANC in urban Blantyre (143). Differences between prevalence estimates by diagnostic criteria could be due to the much lower fasting plasma glucose cut-off level within the IAGPSG criteria compared to the WHO criteria (133, 143, 144).

3.1.4.2 Model

Figure S12 describes the model of GDM, and Table S42 describes the relevant parameters.

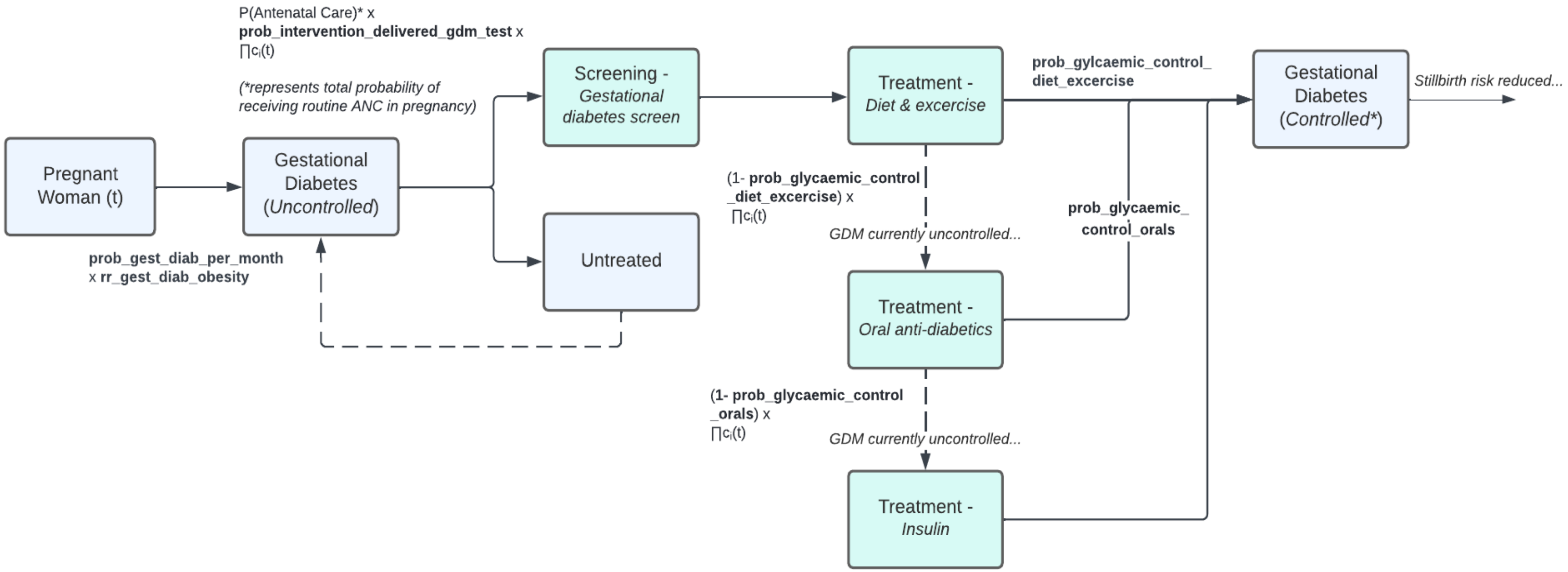

Figure S12 – Model of gestational diabetes mellitus
Diagrammatic representation of the model of gestational diabetes. Light blue represents the model's natural history without treatment whilst teal represents treatment pathways. Parameters representing progression through model states are shown here.

A per-month risk of GDM onset is applied to all pregnant women from 22 weeks GA (month five) with this time point being selected as the vast majority of cases onset later in pregnancy, as normal metabolic processes do not occur, and insulin resistance leads to uncontrolled hyperglycaemia (136). The total monthly risk of GDM onset, $Y_{(t)}$ in equation 10 is calculated as:

$$Y_{(t)} = \mathbf{prob\_gest\_diab\_per\_month}_{(t)} * (li\_bmi\_30\_plus * \ \mathbf{rr\_gest\_diab\_obesity}) \tag{10}$$

Parameters for this equation are shown in Table S42. Obesity was identified as the primary causal influence on individual probability of gestational diabetes to be included in the model (145). New-onset GDM is assumed to present as hyperglycaemia whilst successful treatment leads to glycaemic control as signified in the model variable representing GDM. In the model it is assumed that uncontrolled GDM will not trigger care seeking and treatment will only be initiated following successful screening during ANC. This assumption was made based on input from clinical experts (54). Furthermore, GDM does not lead to maternal death within the model as death from GDM was determined to be extremely rare during pregnancy (54). Therefore GDM resolves at birth and whilst there is evidence that women who experience GDM are at greater risk of type-2-diabetes mellitus (146,147), this relationship is not yet included in the TLO model and will be considered for future iterations

3.1.4.2.1 Treatment

Cases of GDM detected through screening during ANC require admission to initiate first line treatment as shown in Figure S12. According to Malawian guidelines, first line treatment is a trial of diet and exercise to control an individual's hyperglycaemia and scheduled to return for a blood glucose test in four weeks' time (32). After initiation of treatment, a probability that this treatment will effectively control an individual's hyperglycaemia, **prob_glycaemic_control_diet_exercise**, is applied prior to the follow-up appointment. If the treatment is effective, no additional action will be taken during the follow-up appointment and no further follow up is scheduled. If the initial treatment has not been effective, and a woman's hyperglycaemia is still 'uncontrolled' then she will be started on the next treatment as per guidelines. Second line treatment is the use of oral antidiabetics, with the

probability of treatment success shown in parameter **prob_glycaemic_control_orals**, whilst third line treatment is the use of insulin, and this is assumed to be completely effective. Only women who are on treatment and their blood sugar levels are well controlled will benefit from treatment which reduces the risk of stillbirth via the parameter **treatment_effect_gdm_case_management** within the multiplicative model predicting risk of antenatal stillbirth – this parameter is defined in Table S52 in §3.1.14.

3.1.4.3 Data sources and parameters

Parameter Name	Description	Value	Source and/or relevant calculation
**prob_gest_diab_per_month**	This parameter is scaled at intialisation of the simulation to account for the unknown prevalence of obesity within the modelled population. Once scaled, as the simulation runs this parameter the parameter is the probability that a pregnant woman, who is not obese, will develop GDM during her pregnancy applied during months 5-9 of pregnancy.	0.0056	Phiri et al. (143) report the prevalence of GDM in 2,274 women attending ANC in five sites in urban Blantyre, Malawi as 1.6%. The parameter presented here was calculated to generate this prevalence within the model when applied monthly to women during pregnancy.
**rr_gest_diab_obesity**	The effect of a pregnant woman being obese (BMI >29) on her risk of developing GDM	3.97	Sourced directly from Santos et al. (145) who report the effect of obesity on risk of GDM from an individual patient data meta-analysis over a quarter of a million pregnancies in the US, Europe, and Australia.
**prob_glycaemic_control_diet_exercise**	The probability that diet and exercise treatment will lead to effective glycaemic control in an individual with GDM	0.5	As we were unable to identify any reliable estimates of the probability diet and exercise leads to effective glycaemic control, therefore the value for this parameter is assumed.

**prob_glycaemic_control_orals**	The probability that oral antidiabetic treatment will lead to effective glycaemic control in an individual with GDM	0.936	Sourced directly from Balsells et al. (55) who estimated treatment failure for Glibenclamide therapy as 6.37% via a systematic review and meta-analysis of trials evaluating GDM treatments. As such it is assumed the probability that treatment affords glycaemic control is 1 – treatment failure.

Table S43 – Parameters of the gestational diabetes model

3.1.5 Syphilis

3.1.5.1 Condition overview

Syphilis is a sexually transmitted bacterial infection caused by *Treponema pallidum* which spreads via skin-to-skin contact from an individual with active lesions (148). Syphilis is included within this model, despite having no causative link with pregnancy, due to the impact of infection on risk of stillbirth (149) and its relatively high incidence in Malawi (150). Within high burden settings, congenital syphilis secondary to untreated syphilis infection is a leading cause of stillbirth and in some cases neonatal death following vertical transmission (149). For simplicity, and as described below, we do not model maternal outcomes of syphilis infection.

The estimated global prevalence of maternal syphilis Infection during pregnancy Is approximately 0.69% (95% CI 0.57- 0.81%), leading to a congenital syphilis rate of 473 (385-561) cases per 100, 000 live births (149). Of the total congenital syphilis cases Korenromp et al. (149) estimate that 53% are experiencing adverse birth outcomes including stillbirth, neonatal death, preterm birth, and clinical cases of syphilis disease (i.e., neonates with clinical signs of syphilis infection). The prevalence of maternal syphilis appears to be highest within the African region compared to other settings and is estimated to be around 1.62% (149). Data of maternal syphilis prevalence during pregnancy for Malawi was extracted from the quarterly integrated HIV program report and is estimated to be around 2% in 2019 (150).

6.1.3.2 Model

Figure S13 describes the model of syphilis and Table S43 contains the model parameters.

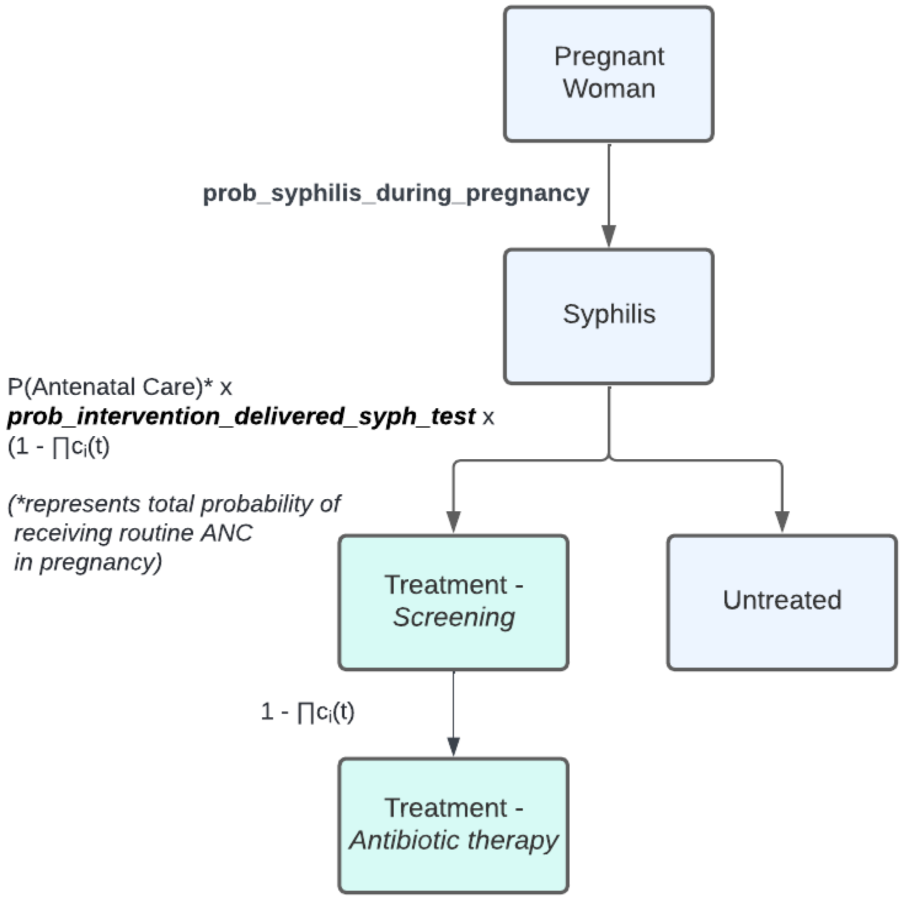

Figure S13 – Model of syphilis
Diagrammatic representation of the model of maternal syphilis. Light blue represents the model's natural history without treatment whilst teal represents treatment pathways. Parameters representing progression through model states are shown here.

The parameter **prob_syphilis_during_pregnancy** represents the total probability of developing syphilis during pregnancy. This probability is applied to all women in the model at the onset of pregnancy to generate the assumed prevalence of maternal syphilis in Malawi. Onset of infection is scheduled to occur randomly over the length of pregnancy and is not assumed to trigger care seeking. This simple model is a significant abstraction from reality and has been included within the MPHM to capture the effect of syphilis screening and treatment during ANC on the risk of stillbirth in the population. As such two main simplifying assumptions have been made within this model. First, we have opted not to model any maternal outcomes of untreated syphilis infection and assume that either following treatment, or the end of pregnancy, any infections will end. Secondly, apart from the effect of syphilis on stillbirth we have chosen not to model congenital syphilis infection in surviving neonates. Whilst congenital syphilis infection in newborns is an important cause

of death in children under-five globally (149,152) it was deemed outside of the remit of the MPHM model at this time due to the models focus on maternal and early newborn outcomes.

6.1.3.2.1 Treatment

Screening occurs during routine ANC for those mothers who seek care. If screening occurs, and the relevant consumables are available, then antibiotic therapy can be administered which, for the purposes of the model, is assumed to be 100% effective at treating infection. The effect of this intervention on perinatal outcomes occurs by removing the effect of syphilis infection on risk of stillbirth as demonstrated in §3.1.14.

6.1.3.3 Data sources and parameters

**Parameter Name**	**Description**	**Value***	**Source and/or relevant calculation**
**prob_syphilis_during_pregnancy**	The per-pregnancy probability that a woman's pregnancy is complicated with syphilis infection	0.026	The prevalence of syphilis during pregnancy in Malawi was reported as 2% in the 2019 Integrated HIV Program Report (150). This source was used as 90% of a cohort of over 160,000 women during ANC were screened for syphilis indicating a likely accurate estimate of prevalence in this population. This parameter has been derived through calibration to this prevalence.

Table S44 – Parameter of the syphilis model

3.1.6 Premature rupture of membranes

3.1.6.1 Condition overview

Rupture of the amniotic membranes at the onset of labour is a normal part of the physiological process of birth, however premature rupture of these membranes, which can occur at any point in pregnancy prior to this, is a significant complication. Infective processes are thought to drive premature rupture of membranes (PROM), especially if occurring early in pregnancy (153), with studies in SSA identifying common gynaecological and sexually transmitted infections as the most common conditions associated with PROM (154,155). At the time of writing there is no modelling of either STIs (excluding HIV and syphilis) or other genital tract infections within the TLO framework, therefore these predictors have not been included.

PROM has been included within the model due to its relationship with both maternal and perinatal outcomes. Commonly, PROM is associated with poor neonatal outcomes, largely due to the relationship between PROM and preterm birth (§3.1.1) and neonatal sepsis (§3.2.3), as preterm neonates in most settings experience greater rates of morbidity and mortality (156,157) as explored in §3.2.1. In addition to poor neonatal outcomes, PROM is associated with maternal infection which may lead to sepsis and mortality in some cases (158) as modelled in §3.1.6.

Estimates of the number of pregnancies that are complicated by PROM globally are lacking, however results from large hospital-based studies suggest that around 3% of singleton pregnancies are complicated by PROM (159). Within SSA there is variation in reported estimates of PROM with studies in South Africa and Nigeria estimating an incidence of 2.7% (95%CI 1.9–3.4) and 4.2% respectively, whilst a systematic review of studies from Ethiopia reporting a much greater pooled prevalence of 9.2% (95% CI 5.0-16.4) (160–162). Due to a lack of estimates from Malawi the incidence rate from Onwughara et al. (160), a study conducted in South Africa, is used in the model as discussed in Table S44.

3.1.6.2 Model

Figure S14 describes the model of PROM and Table S44 contains the model parameter.

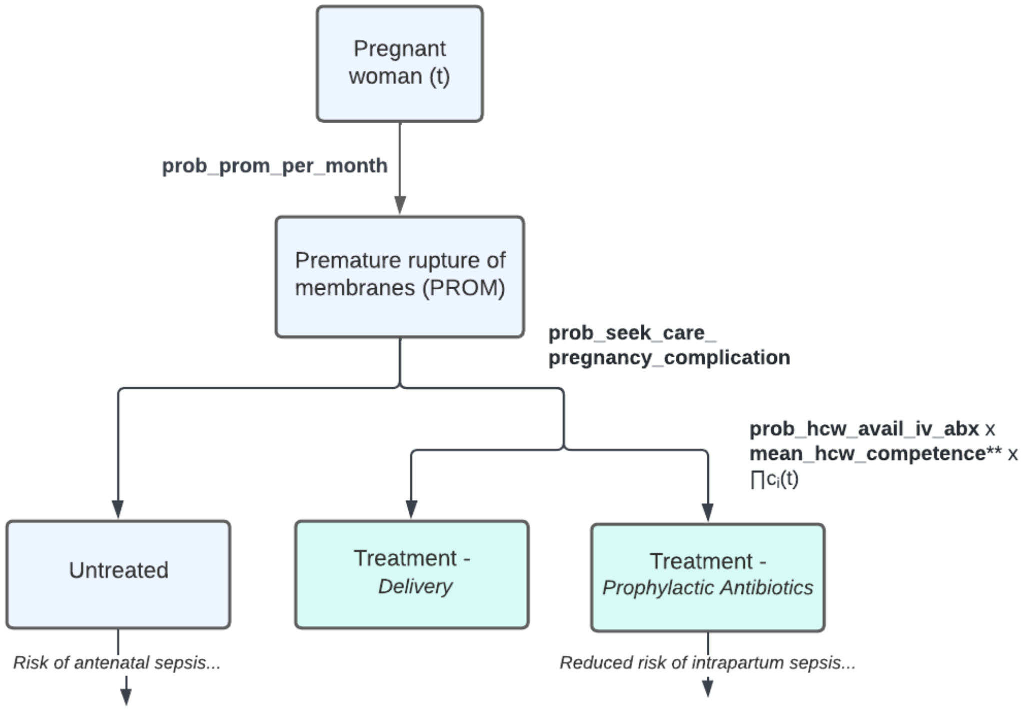

Figure S14 – Model of premature rupture of membranes
Diagrammatic representation of the model of premature rupture of membranes. Light blue represents the model's natural history without treatment whilst teal represents treatment pathways. Parameters representing progression through model states are shown here.

A fixed per-month risk of PROM, **prob_prom_per_month**, is applied to all women from 22 weeks GA. The onset of PROM is assumed to trigger possible care seeking as an antenatal emergency following onset, allowing for treatment to be initiated as shown in Figure S14. Untreated PROM can lead to antenatal sepsis as described later in this section.

3.1.6.2.1 Treatment

Treatment for PROM as described in Malawian guidelines includes administration of prophylactic antibiotics and, for women who are not already in labour, admission and scheduled delivery via induction of labour. This is replicated in the model with delivery scheduled for women after reaching 37 weeks GA. The effect of prophylactic antibiotics on maternal and neonatal outcomes is described in §3.1.6.and §3.2.3 respectively.

3.1.6.3 Data sources and parameters

Parameter Name	Description	Value*	Source and/or relevant calculation
prob_prom_per_month	The monthly probability that a pregnant woman will experience PROM applied during months 5-9 of pregnancy.	0.0064	The assumed rate of PROM in the model is sourced from a study conducted by Onwughara et al. (160). The authors observed the number of PROM cases in 1758 deliveries at a regional hospital in South Africa reporting a rate of 27 cases per 1000 births. This study was chosen due to lacking data from Malawi and geographic proximity.<br><br>The Initial value was calculated as (27/1000) / 5 (months risk is applied) = 0.0054. This value was then manipulated during calibration to generate the correct rate at birth (given the rate of pregnancy loss in the population).

Table S45 – Parameter of the PROM model

3.1.7 Preterm and post term labour

3.1.7.1 Condition overview

Term gestation, or birth at term, is usually defined as delivery between 37- and 41-weeks GA (163). Births occurring before this are classically defined as preterm and those occurring after are categorised as post term with both 'early' and 'late' births being associated with poor foetal and neonatal outcomes, such as stillbirth and neonatal mortality (163). Preterm birth can be further sub-categorised as either early preterm, occurring between 24- and 33-weeks GA, and late preterm occurring between 34- and 36-weeks GA (165).

Whilst the aetiology of preterm birth has been extensively researched within the literature some authors suggesting the underlying causes of preterm birth remain poorly understood (165). However, epidemiological research has identified several key drivers on preterm birth, especially in high burden settings. Following review of the literature from Malawi and surrounding territories, the following factors impacting risk of preterm birth were identified for inclusion in the model: PROM (166), maternal anaemia (164,166,167), maternal malaria (164,166,167) and twin pregnancy (166). Despite the potentially causal relationship between maternal malaria and anaemia, data from a study within Malawi suggests the effect of these factors is likely independent (168).

Globally, preterm birth is reported as the leading cause of neonatal death (169). Mortality associated with prematurity is driven by several possible life-threatening complications including preterm respiratory distress syndrome, necrotising enterococcus, intraventricular haemorrhage, hypothermia, and sepsis (170). Additionally, preterm neonates who survive into child and adulthood are considerably more likely than their term counterparts to experience neurodevelopmental disability (15,171,172). Complications associated with prematurity are included in the neonatal model and discussed in detail in §3.2.1.

Post term pregnancy, where pregnancy continues beyond 41-weeks GA, does not have a clear aetiology, however evidence suggests that maternal obesity may be a driver for pregnancies continuing past term (173), with this relationship included in the model. Poor perinatal outcomes associated with post term pregnancy appear to be less substantial than those from preterm birth; however, there is strong evidence to suggest that post term

pregnancy is associated with an increased risk of stillbirth, which increases with each week the pregnancy progress (174), as depicted in the model currently.

Preterm and post term delivery are relatively common globally with evidence of international variation in incidence. The average global rate of preterm birth in 2014 was estimated to be approximately 10.6% (UI 9·0, 12·0) leading to nearly fifteen-million preterm live births for that year, but this rate is reported to be much higher within northern Africa (13·4% (6·3, 30·9)) and SSA (12·0% (8·6, 16·7)) (175). Recent estimates of the PTB rate in Malawi are provided by Chawanpaiboon et al. (175) who predict a rate of 10.5 (UI 7.4, 14.3) preterm births per 100 births (see Table S45). Conversely, data on post term birth incidence at the global level is limited, but a cohort study evaluating over six million pregnancies in China reported that 1.16% of births occurring after term occurred after 42 weeks GA (176). In Malawi, van den Broek et al. (164) report a higher rate of post term birth, 3.2%, in a cohort of pregnant Malawians, which is used in the model.

3.1.7.2 Model

Figure S15 describes the model of preterm and post term birth with Table S45 containing the relevant parameters.

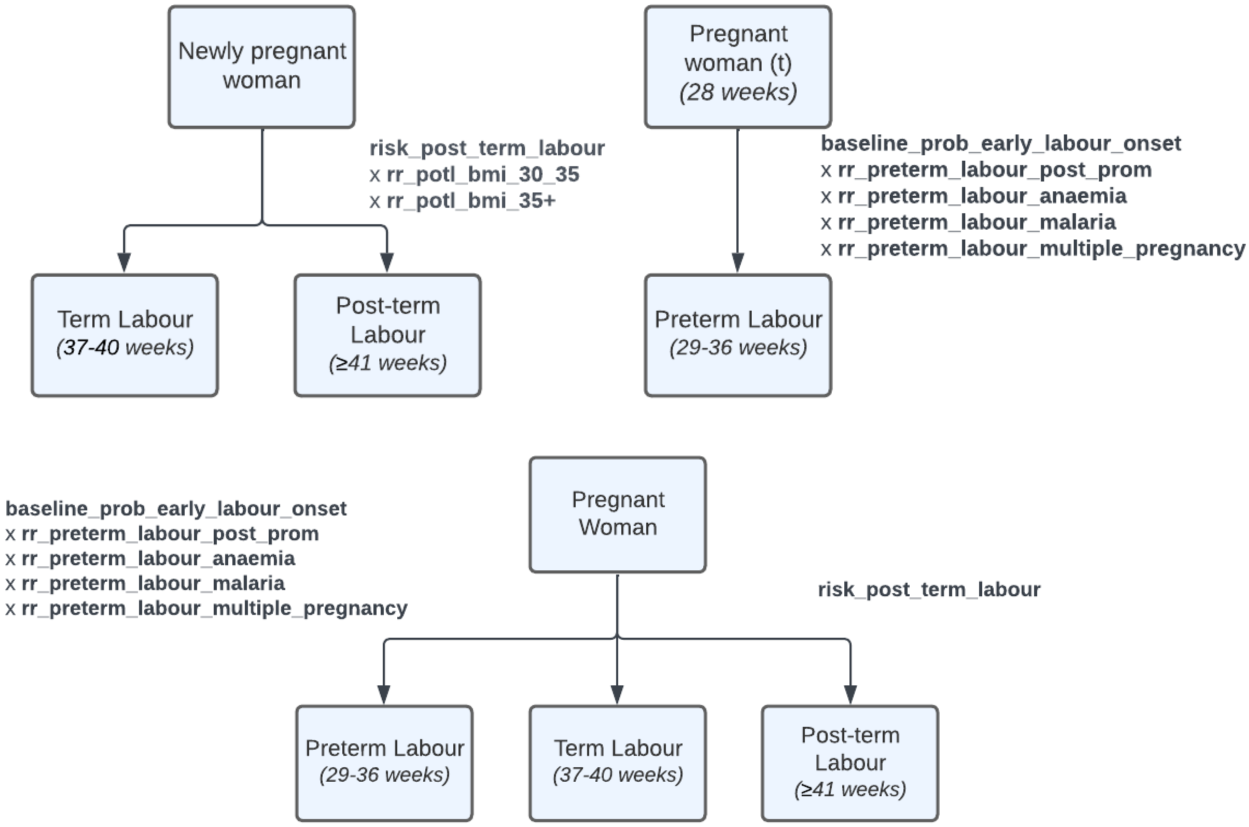

Figure S15 – Model of post term and preterm birth
Diagrammatic representation of the models of preterm and post term birth. Light blue represents the model's natural history without treatment. Parameters representing progression through model states are shown here.

As shown in the figure above, at the initiation of each pregnancy the newly pregnant woman is scheduled to go into labour and give birth after 36 weeks GA either at term or post term. An individual's risk that labour will continue beyond 40 weeks is calculated as:

$$Y_{(t)} = \mathbf{risk\_post\_term\_labour} * (li\_bmi\_30\_35 * \mathbf{rr\_potl\_bmi\_30\_35}) * (li\_bmi\_35plus * \mathbf{rr\_potl\_bmi\_35} +) \quad (11)$$

As explained above, it is assumed that around 3.2% of pregnancies in Malawi continue past 41 weeks, in line with data from the country (164) and include maternal obesity as a predictor. Since this risk is applied following conception, equation 11 generates the rate of post term birth in the model accounting for pregnancy loss and the rate of preterm birth applied to women as their pregnancy progresses discussed below.

To generate the correct assumed rate of preterm labour in the model, from 28 weeks GA the risk of preterm labour onset is applied to all women on a monthly timestep until they reach term gestation. This risk of preterm labour onset for a given month is calculated as follows:

$$
\begin{aligned}
Y_{(t)} = \mathbf{baseline\_prob\_early\_labour\_onset}_{(t)} \\
&* (ps\_premature\_rupture\_of\_membranes * \mathbf{rr\_preterm\_labour\_post\_prom}) \\
&* (ps\_multiple\_pregnancy * \mathbf{rr\_preterm\_labour\_multiple\_pregnancy}) \\
&* (ps\_anaemia\_in\_pregnancy * \mathbf{rr\_preterm\_labour\_anaemia}) \\
&* (ma\_is\_infected * \mathbf{rr\_preterm\_labour\_malaria})
\end{aligned}
\tag{12}
$$

The constant for this equation, **baseline_prob_early_labour_onset**, is variable by month of gestation, to produce the correct distribution of newborns who are born early preterm and late preterm, as observed within Malawi (164). At this point the individual's previously predicted delivery date, which is stored in the model at pregnancy onset, is overridden. The interaction between preterm labour onset and neonatal outcomes is described in §3.2.1.

3.1.7.3 Data sources and parameters

Parameter Name	Description	Value*	Source and/or relevant calculation
**baseline_prob_early_labour_onset**	This parameter is scaled at intialisation of the simulation to account for the prevalence of malaria within the modelled population. Once scaled, as the simulation runs this parameter is the probability that a pregnant woman who has not experienced PROM, is not anaemic, does not have malaria and is not pregnant with twin foetuses will go into labour between 28- and 36-weeks GA.<br><br>The parameters below starting with "rr_preterm_labour" refer to the effect on the probability of preterm labour	[0.0008, 0.0033, 0.017, 0.04],	The assumed rate of preterm birth in Malawi is sourced from Chawanpaiboon et al. (175) as 10.5 preterm births per 100 births. The authors estimate this rate via linear regression using population representative survey data points from Malawi.<br><br>The figures within the list of values represents the baseline risk of preterm birth onset in months 5, 6, 7 and 8 of pregnancy. The probability of preterm birth increases with GA to ensure the model reproduces the assumed distribution of early vs late preterm births (24.8% vs 75.2%) as sourced from van de Broek at al. (164). The values here were calculated to achieve both the rate of preterm birth and distribution of timing.
**rr_preterm_labour_post_prom**	The effect of a pregnant woman having experienced PROM	5.9	Sourced directly from Laelago et al. (166) in which the authors report the effect of several determinants on risk of preterm birth derived from a systematic review and meta-analysis of studies conducted in east Africa.
**rr_preterm_labour_anaemia**	The effect of a pregnant woman being anaemic	4.58	See **rr_preterm_labour_post_prom.**
**rr_preterm_labour_malaria**	The effect of a pregnant woman experiencing malaria infection	3.08	See **rr_preterm_labour_post_prom.**
**rr_preterm_labour_multiple_pregnancy**	The effect of a pregnant woman being pregnant with twin foetuses	3.44	See **rr_preterm_labour_post_prom.**

**risk_post_term_labour**	The per-pregnancy probability that a woman's new pregnancy will end in the onset of post term labour	0.077	Rate of post term birth sourced directly from van de Broek at al. (164) in which 3.2% of observed deliveries in a cohort of 2149 births occurred post term in Malawi. Final value derived from calibration to total post term birth rate outputted by model accommodating for pregnancy loss and preterm birth.
**rr_potl_bmi_30_35**	The effect of a woman having a BMI between 30 and 34.9 compared to less than 27.5 at the beginning of pregnancy on her risk of post term labour	1.42	Sourced directly from Heslehurst et al. (173) in which the authors report the effect of maternal BMI on risk of post term labour via a systematic review and meta-analysis of 39 studies including over four million births.
**rr_potl_bmi_35+**	The effect of a woman having a BMI of 35 or greater compared to less than 27.5 at the beginning of pregnancy on her risk of post term labour	1.55	see **rr_potl_bmi_30_35.**

Table S46 – Parameters of the preterm and post term labour model

3.1.8 Maternal sepsis

3.1.8.1 Condition overview

Maternal sepsis is "a life-threatening condition defined as organ dysfunction resulting from infection during pregnancy, child-birth, post-abortion, or post-partum period" (177). Within the MPHM we explicitly model sepsis secondary to the five most common infections observed in mothers: chorioamnionitis, endometritis, urinary tract infection and skin or soft tissue infection and abortion related, which have been reported to constitute over 80% of observed maternal infections in multi-centre studies (178). Other potential causes of maternal sepsis (i.e., respiratory infection) are not currently modelled and are outside of the remit of this study. Additionally, we have also endeavoured to capture the variation in the aetiology of sepsis across the pregnancy continuum as described below.

3.1.8.1.1 Antenatal and intrapartum sepsis

Maternal infection during the antenatal and intrapartum period of pregnancy is often attributed to chorioamnionitis in which the placenta, chorion and/or amnion become inflamed and/or infected during pregnancy (179,180). The aetiology of chorioamnionitis is complex, involving a possibly circular causal relationship with PROM (180), but is most often attributed to vertical bacterial transmission following membrane rupture.

The relationship between PROM and chorioamnionitis Is supported by several studies (180,181) and has therefore been captured in the model as shown below. Both clinical and histological chorioamnionitis can have significant impact on maternal and perinatal health outcomes and in some cases can lead to mortality (182–185).

3.1.8.1.2 Postpartum sepsis

Sepsis which onsets during the postnatal period is a significant driver of poor maternal outcomes globally, as infection during this time is more common than other stages of the pregnancy continuum (178). We focused on the most common causes of direct postnatal sepsis including endometrial, skin, or soft tissue and urinary tract infections (177,185,186).

Endometritis, broadly defined as infection within the uterine lining, or endometrium, can occur frequently following birth and is often reported as the leading cause of postnatal

sepsis (178,187). The pathogenesis of postpartum endometritis is associated with uterine cavity contamination with vaginal organisms during the process of labour and delivery (187,188). Evidence suggests an association between delivery of invasive interventions, such as CS, and endometritis, with much higher incidence rates observed in women post caesarean (188). This was confirmed in consultation with clinical experts leading to inclusion of this relationship within the model (3).

Skin and soft tissue infections, often originating in wounds caused by obstetric surgery, are a similarly common cause of sepsis in most settings (178,185,189). Despite the recommendation and widespread use of prophylactic antibiotics prior to the conduct of a caesarean delivery (190), many mothers in east African settings experience these infections which onsets within the community (191). Importantly, these infections are not limited to caesarean wounds but can occur following any invasive procedure, such as episiotomy or intravenous cannulation, and as such are highly preventable.

Urinary tract infections (UTI), the final cause of postnatal sepsis included in the model, can onset during pregnancy, but is seemingly more common in the postnatal period as UTI may onset following invasive procedures, such as urinary catheterisation during labour or obstetric surgery (192) although they may also onset following unassisted vaginal delivery (193). Due to inconclusive results from studies investigating predictors of UTI in settings like Malawi specific risk factor for UTI are not included in the model.

Globally, postnatal sepsis, and maternal sepsis more generally, is associated with significant morbidity and mortality in women (194), with Bonet et al. (178) estimating an interfacility case fatality of 6.5% for women with severe postnatal sepsis in their multi-site study conducted in 52 LMIC and HIC including Malawi. Sepsis represents one of the leading causes of maternal death (194), including in Malawi (33), and untreated can lead to shock and multi-system organ failure.

Estimates of the incidence of maternal sepsis vary within the literature. The recent WHO Global Maternal Sepsis Study estimated in-facility rates of severe maternal outcomes secondary to infection in the study population as 10·9 (95% CI 9·8–12·0) women per 1000

livebirths, occurring most frequently in postnatal and post abortion women (178). The authors report interregional disparities with higher rates of infection noted in facilities from LMICs (178). Estimates produced by Woodd et al. (185), via a systematic review of published literature, are more conservative reporting an incidence of maternal sepsis of 0.5 per 1000 births, however the authors do report that these studies were mostly conducted in the US and Europe. In Malawi the number of in-facility postpartum sepsis cases per survey year reported is captured by the Malawian BEmONC surveys leading to a rate of 2.34 per 1000 births in 2010 and 1.5 per 1000 births in 2015 (33,34) which have been used as the calibration rates for the model.

3.1.8.2 Models

3.1.8.2.1 Antenatal sepsis

Figure S16 describes the model of sepsis onsetting prior to labour and Table S46 contains the relevant parameters.

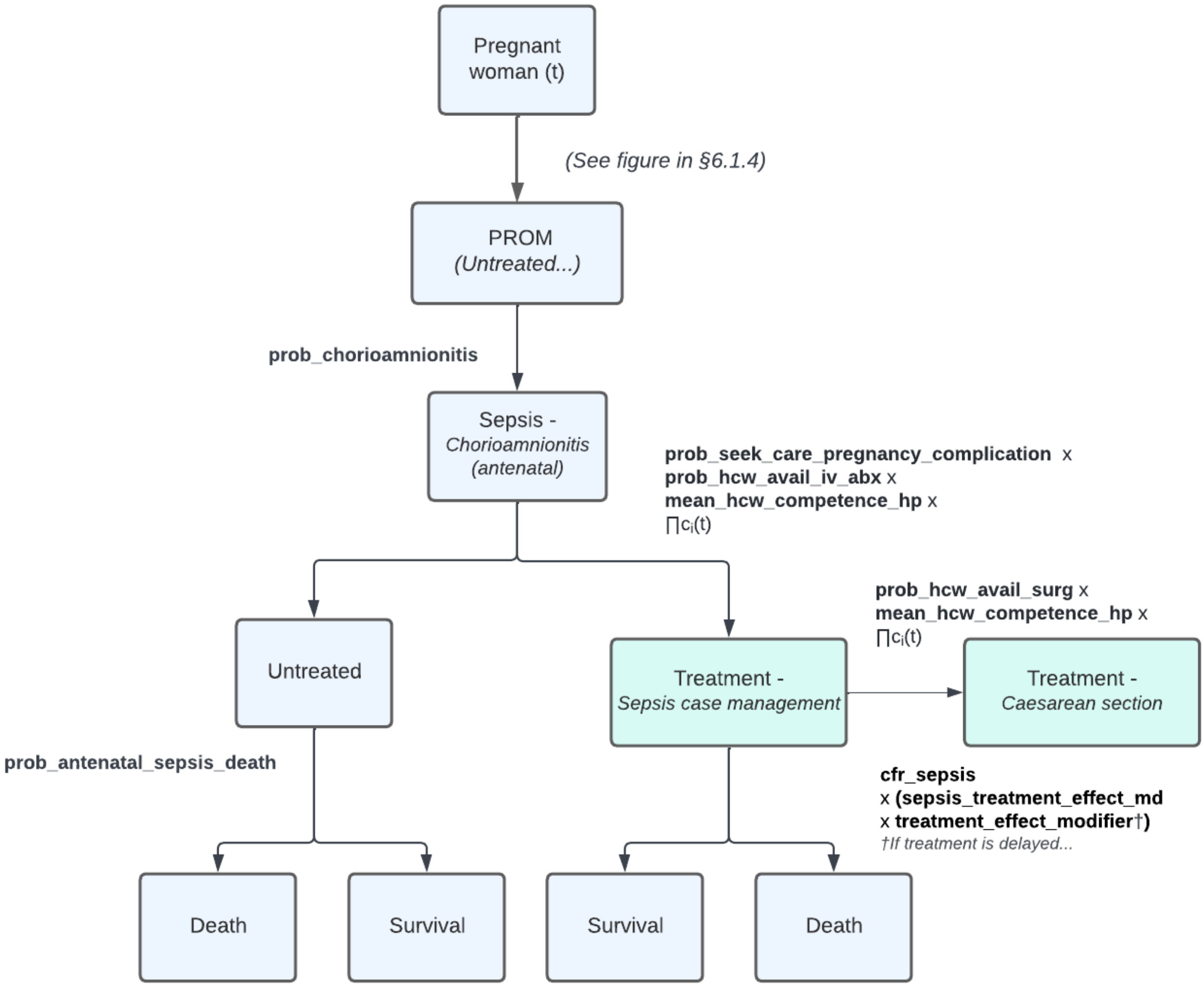

Figure S16 – Model of antenatal sepsis
Diagrammatic representation of the model of antenatal sepsis. Light blue represents the model's natural history without treatment whilst teal represents treatment pathways. Parameters representing progression through model states are shown here.

Risk of antenatal sepsis due to chorioamnionitis during pregnancy, **prob_chorioamnionitis,** is applied to any woman who experiences PROM and has not received antenatal treatment. Women who develop antenatal sepsis may choose to seek care as shown in Figure S16 which leads to the initiation of treatment described below. The parameter **prob_antenatal_sepsis_death** represents the risk of maternal mortality in those women

who choose not to receive treatment. Otherwise, risk of death is calculated during the intrapartum phase of pregnancy accounting for treatment received.

3.1.8.2.1.1 Treatment

Individuals who seek antenatal inpatient care following sepsis onset are scheduled for delivery via induction of labour from 28 weeks GA, in keeping with Malawian clinical guidelines (32). Further to this, case management for maternal sepsis at any point of pregnancy includes the delivery of broad-spectrum parenteral antibiotics and the administration of fluids and oxygen to any individual experiencing two or more clinical indicators (32). The treatment effect, **sepsis_treatment_effect_md,** is sourced from Pollard et al. (37) and is a Delphi estimate of the effectiveness of parenteral antibiotics on risk of sepsis death in pregnancy. Interestingly, this study reports a greater effectiveness of parenteral antibiotic delivery alone compared to 'BEmONC' as an intervention to manage sepsis and whilst this inconsistency is noted in the discussion it is not fully justified. Therefore, it was deemed appropriate to take the estimate relating to antibiotic delivery as the primary treatment effect. As such, whilst consumables relating to oxygen and fluids are requested, they are not deemed essential to deliver the intervention.

3.1.8.2.2 Intrapartum sepsis

Figure S17 describes the model of sepsis onsetting during labour and Table S46 contains the relevant parameters.

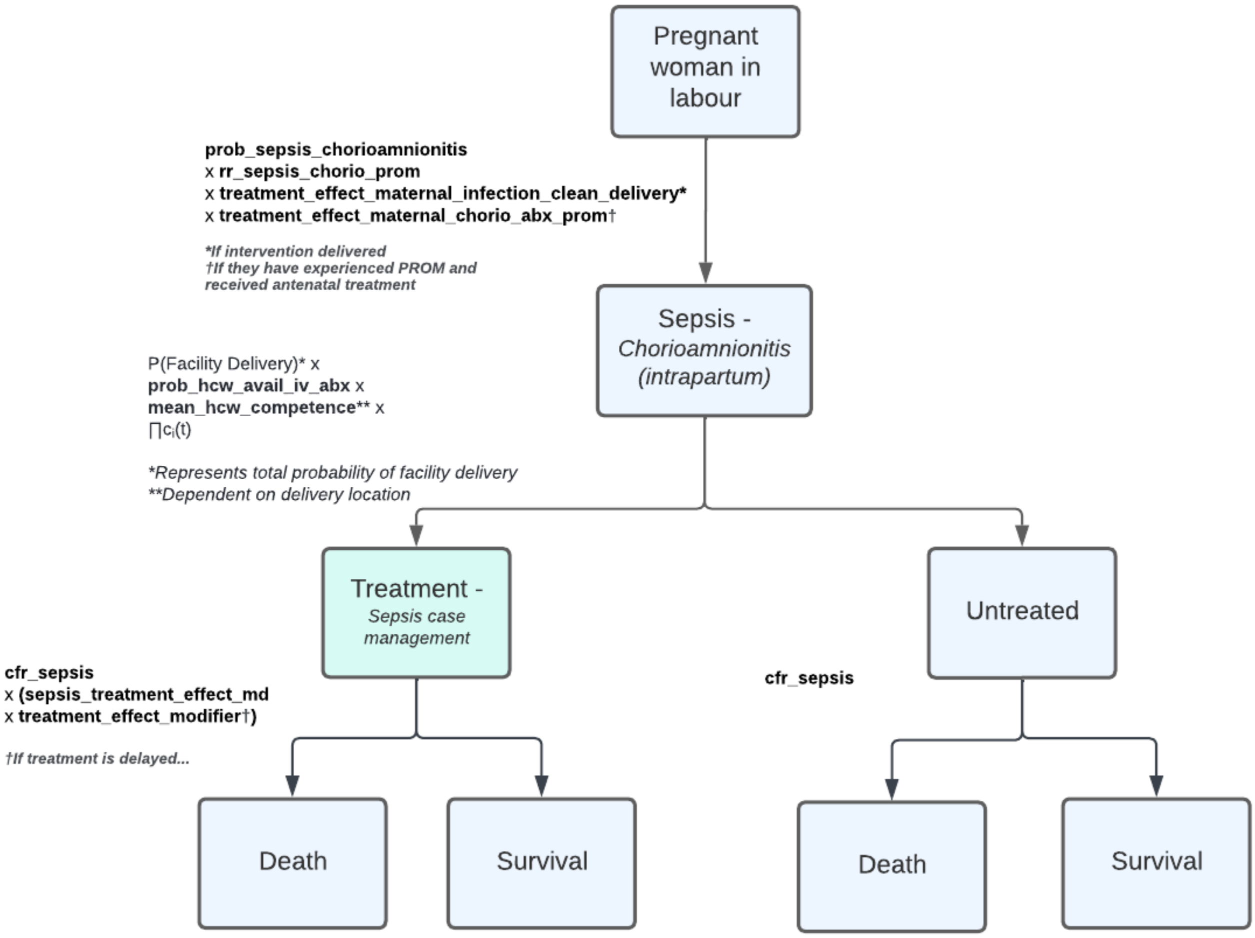

Figure S17 – Model of intrapartum sepsis
Diagrammatic representation of the model of intrapartum sepsis. Light blue represents the model's natural history without treatment whilst teal represents treatment pathways. Parameters representing progression through model states are shown here.

The probability that a mother in labour will develop sepsis secondary to chorioamnionitis is calculated shown in equation 13:

$$Y_{(t)} = \mathbf{prob\_sepsis\_chorioamnionitis} * (ps\_premature\_rupture\_of\_membranes * \mathbf{rr\_sepsis\_chorio\_prom}) * (ac\_received\_abx\_for\_prom * \mathbf{treatment\_effect\_maternal\_chorio\_abx\_prom}) * (mni\_clean\_birth\_practices * \mathbf{treatment\_effect\_maternal\_infection\_clean\_delivery}) \tag{13}$$

Unlike the risk of chorioamnionitis applied in the antenatal period, in which only women with PROM may develop infection, we have opted to model an underlying risk of sepsis during labour to women without PROM, **prob_sepsis_chorioamnionitis**, under the assumption that membrane rupture will occur in all delivering women leading to the possibility of infection associated with either natural or interventional processes of labour and delivery (195). As seen in equation 13, PROM remains a predictor for infection (181) but in this equation, risk is mitigated in the presence of two treatments; prophylactic antibiotics given to women who have sought and received care due to PROM antenatally (59) and clean delivery practices during labour (37), which were first introduced in §2.

As with the other intrapartum complications, the onset of intrapartum sepsis during home birth is assumed to trigger possible care seeking, via parameter **prob_careseeking_for_complication,** allowing mothers to receive treatment described below. The parameter **cfr_sepsis** represents the risk of death in untreated women as described in Table S46.

3.1.8.2.2.1 Treatment

Similarly to individuals who develop sepsis prior to labour, those who develop intrapartum sepsis whilst labour in a health facility, or at home but choose to seek care, may receive maternal sepsis case management, as described above and shown in Figure S17, reducing risk of death.

3.1.8.2.3 Postpartum sepsis

Figure S18 describes the model of sepsis onsetting during labour and Table S46 contains the relevant parameters.

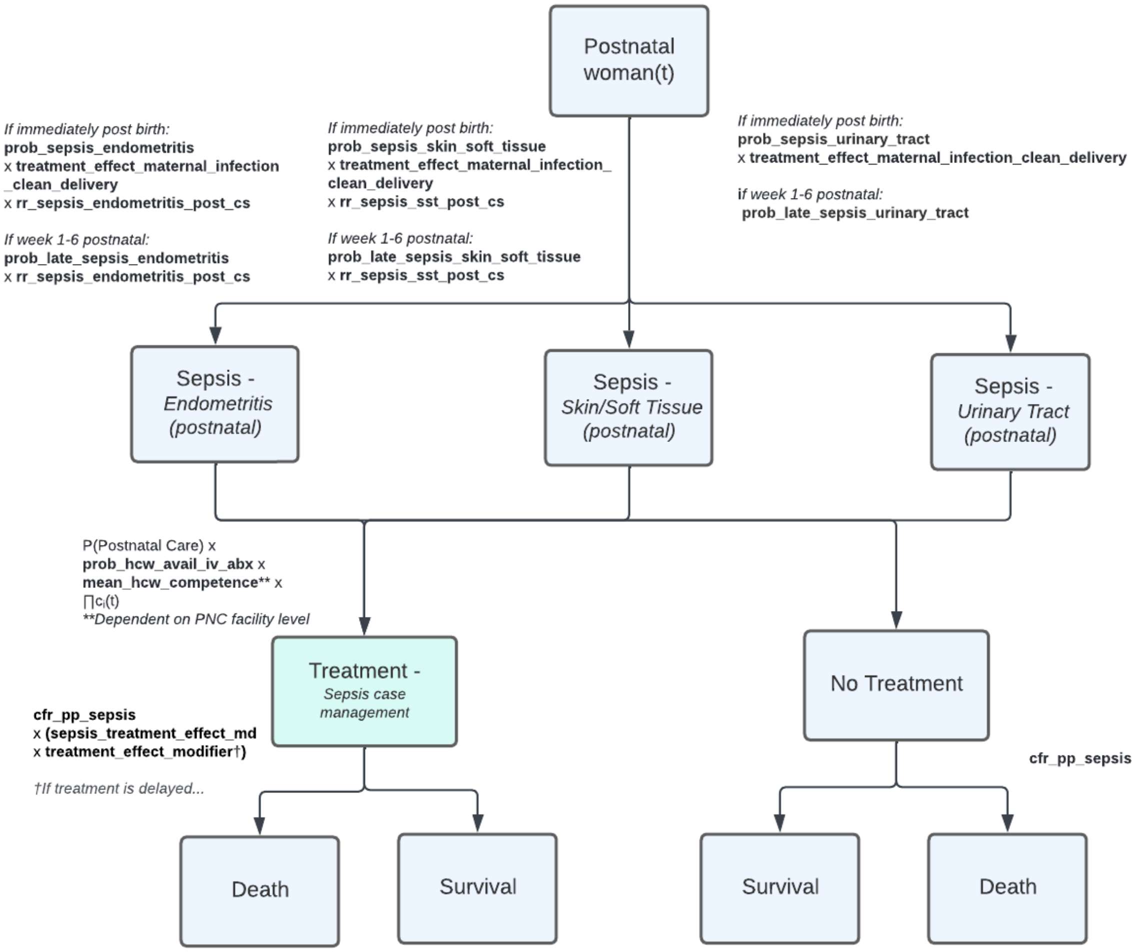

Figure S18 – Model of postnatal sepsis
Diagrammatic representation of the model of postnatal sepsis. Light blue represents the model's natural history without treatment whilst teal represents treatment pathways. Parameters representing progression through model states are shown here.

In-keeping with other key postnatal complications, the risk of postnatal sepsis is applied immediately post birth, during the first week of the postnatal period and then during each week of the remaining postnatal period within the relevant events.

The individual risk of sepsis secondary to endometritis and skin/soft tissue infection for each of these time steps is calculated as:

$$Y_{(t)} = \mathbf{prob\_sepsis\_endometritis} * (mni\_delivery\_mode * \mathbf{rr\_sepsis\_endometritis\_post\_cs}) * (mni\_clean\_birth\_practices * \mathbf{treatment\_effect\_maternal\_infection\_clean\_delivery}) \quad (14)$$

$$Y_{(t)} = \mathbf{prob\_sepsis\_skin\_soft\_tissue} * (mni\_delivery\_mode * \mathbf{rr\_sepsis\_endometritis\_post\_cs}) * (mni\_clean\_birth\_practices * \mathbf{treatment\_effect\_maternal\_infection\_clean\_delivery}) \quad (15)$$

Whilst the risk of sepsis secondary to urinary tract infection is simply:

$$Y_{(t)} = \mathbf{prob\_sepsis\_urinary\_tract} * (mni\_clean\_birth\_practices * \mathbf{treatment\_effect\_maternal\_infection\_clean\_delivery}) \quad (16)$$

As is evident from the equations above it is assumed that receipt of clean delivery practices during labour reduces the risk of mothers developing sepsis in the immediate postnatal period via the parameter **treatment_effect_maternal_infection_clean_delivery**. This effect is not applied after the first week of the postnatal period as evident in the figure above.

Additionally, it is assumed that women can experience infection from multiple sources simultaneously therefore the total risk of sepsis for each time point is equal to:

$$1 - ((1 - p(sepsis\_endometritis) * (1 - p(sepsis\_skin\_soft\_tissue) * (1 - p(sepsis\_urinary\_tract)) \quad (17)$$

3.1.8.2.3.1 Treatment

Treatment for postnatal sepsis is initiated via PNC as previously described. Women who are identified as septic during routine PNC will be admitted to the postnatal ward and will receive maternal sepsis case management as described above.

3.1.8.3 Data sources and parameters

Parameter Name	Description	Value*	Source and/or relevant calculation
Antenatal parameters			
**prob_chorioamnionitis**	The probability that a pregnant woman who has experienced PROM and has not sought care will develop sepsis due to chorioamnionitis prior to labour onset	0.015 / 0.013	Initially the assumed rate of maternal sepsis in Malawi was sourced from the 2010 and 2015 Malawi EmONC assessment surveys (33,34) by dividing the total sepsis cases observed in the survey by the estimated births for the survey year giving a rate of 2.34 and 1.5 per 1000 births respectively.<br><br>The proportion of the total cases of sepsis attributable to each underlying cause included in the model was sourced from Bonet et al. (178). From this study it is estimated that 16% of sepsis cases are due to chorioamnionitis leading to an approximate rate of 0.3 per 1000 the model.<br><br>This parameter and **prob_sepsis_chorioamnionitis** have been derived through the process of calibration to the overall rate of sepsis and the estimated proportion of cases due to this cause.
**prob_antenatal_sepsis_death**	The probability that a pregnant woman will die from sepsis due to chorioamnionitis which has onset during the antenatal period without treatment	0.75 / 0.49	See **prob_ectopic_pregnancy_death** in Table S39**.**
Intrapartum parameters			
**prob_sepsis_chorioamnionitis**	The probability that a pregnant woman in labour will develop sepsis secondary to chorioamnionitis	0.0002 / 0.00018	See **prob_chorioamnionitis.**

**rr_sepsis_chorio_prom**	The effect of a pregnant woman having experienced PROM compared to not having experienced PROM on her risk of developing sepsis secondary to chorioamnionitis.	1.76	Sourced directly from Seaward et al. (181) in which the authors report the effect of several determinants of chorioamnionitis in a cohort of over 5000 women across several HICs via a multivariate logistic regression model.
**cfr_sepsis**	The probability that a pregnant woman will die following sepsis during labour without treatment.	0.75 / 0.49	See **prob_antenatal_sepsis_death.**
Postnatal parameters			
**prob_sepsis_endometritis**	The probability of a postnatal woman developing postnatal sepsis secondary to endometritis within the first forty-eight hours after birth who has not delivered via CS	0.000069/ 0.000062	See **prob_chorioamnionitis.** From Bonet et al (178) it is estimated 36% of sepsis cases are due to endometritis leading to an approximate rate of 0.71 per 1000 births.
**rr_sepsis_endometritis_post_cs**	The effect of a postnatal woman having delivered via CS compared to vaginal delivery on their risk of developing postpartum sepsis, secondary to endometritis	12.1	Sourced directly from Newton et al. (196) who identified predictors of endometritis in a cohort of 607 labouring women via multivariate logistic regression.
**prob_sepsis_urinary_tract**	The probability of a postnatal woman developing postnatal sepsis secondary to a urinary tract infection within the first forty-eight hours since birth	0.000054 /0.000054	See **prob_chorioamnionitis.** From Bonet et al (178) it is estimated 27% of sepsis cases are due to urinary tract infection leading to a rate of 0.54 per 1000.

**prob_sepsis_skin_soft_tissue**	The probability of a postnatal woman developing postnatal sepsis secondary to a skin/soft tissue infection within the first forty-eight hours since birth	0.000039/ 0.000035	See **prob_chorioamnionitis.** From Bonet et al (178) it is estimated 21% of sepsis cases are due to skin or soft tissue infection leading to rate of 0.41 per 1000 births**.**
**rr_sepsis_sst_post_cs**	The effect of a postnatal woman having delivered via CS compared to vaginal delivery on their risk of developing postpartum sepsis, secondary to skin/soft tissue infection	3.9	Sourced directly from Ngonzi et al. (197) who reported demographic and clinical factors associated with postnatal sepsis through a prospective study in Uganda.
**cfr_pp_sepsis / cfr_postpartum_sepsis**	The probability that a postnatal woman will die following postnatal sepsis that has onset during the postnatal period without treatment	0.75 / 0.49	See **prob_antenatal_sepsis_death.**
**prob_late_sepsis_endometritis**	The probability of a postnatal woman (who has not delivered via CS) developing postnatal sepsis secondary to endometritis per week of the postnatal period	0.000069/ 0.000062	See **prob_sepsis_endometritis.**
**prob_late_sepsis_urinary_tract**	The probability of a postnatal woman developing postnatal sepsis secondary to urinary tract infection per week of the postnatal period	0.000054 / 0.000054	See **prob_sepsis_urinary_tract**

**prob_late_sepsis_skin_soft_tissue**	The probability of a postnatal woman (who has not recently delivered via CS) developing postnatal sepsis secondary to skin or soft tissue infection per week of the postnatal period	0.000039/ 0.000035	See **prob_sepsis_skin_soft_tissue**
Treatment parameters			
**treatment_effect_maternal_infection_clean_delivery**	The effect of a pregnant woman in labour receiving clean delivery practices on risk of sepsis	0.4	Sourced directly taken from Pollard et al. (37) who estimate the effect of clean birth and postnatal practices on risk of maternal death due to sepsis via Delphi method as a 60% reduction. For the purposes of the model, the same effect on reducing the risk of sepsis onset is assumed.
**treatment_effect_maternal_chorio_abx_prom**	The effect of antibiotic treatment on maternal risk of sepsis secondary to chorioamnionitis	0.66	Sourced directly from Kenyon et al. (59) who reports the effect of antibiotic treatment for PROM on risk of chorioamnionitis as derived from a Cochrane review of RCTs as RR 0.66 (95% CI 0.46 to 0.96).
**sepsis_treatment_effect_md**	The effect of case management of maternal sepsis on risk of maternal death due to sepsis	0.2	Sourced directly from Pollard et al. (37) who estimate the effect of antibiotic therapy on maternal death due to sepsis via a Delphi survey. Effectiveness is reported as 80%.

** Where two values (or sets of values) are provided the first set is applied from 2010-2014 and the second set from 2015 onwards for a given simulation run (§1.2.1.1)*

Table S47 -Parameters of the models for maternal sepsis

3.1.9 Antepartum Haemorrhage

3.1.9.1 Condition overview

Antepartum haemorrhage (APH) is defined as any bleeding from or into the genital tract, occurring from 24 weeks of pregnancy and prior to the birth of the baby (198). As such within the model APH may onset either prior to or during labour. Although a proportion of APH cases present with unclear aetiology, several studies report that placental abruption and placenta praevia are the leading causes of APH both globally and in Malawi (199–204). Due to this, we have opted to explicitly model both underlying causes of APH within this framework.

Placenta praevia is a condition in which the placenta covers the internal os, the opening of the cervix into the uterus, completely (205). Commonly, the risk of placenta praevia is associated with previous CS delivery, as it is hypothesised that the presence of a uterine scar in the lower segment may encourage placentation closer to the internal os (206–208). Whilst placenta praevia refers to the position of the placenta within the uterine wall, placental abruption describes a complication in which the implanted placenta separates prematurely, prior to delivery of the foetus (209). Similarly to placenta praevia, placental abruption is also associated with previous caesarean delivery but has also been found to be associated with maternal hypertension (210,211) .

Both conditions, if undetected and untreated, may lead to significant bleeding either prior to labour onset or during the intrapartum phase of pregnancy. Substantial bleeding from the genital tract during the antenatal and intrapartum period of pregnancy, and its antecedent causes, occur less frequently than bleeding following delivery, but are still strongly associated with poor maternal and perinatal outcomes globally (199,200,214) and in Malawi (203, 215).

Globally, the incidence of placenta praevia is reported at 5.2 (95% CI: 4.5, 5.9) per 1000 pregnancies with evidence of significant variation between regions (212). Studies estimating incidence in SSA reported the lowest overall incidence, 2.7 (95% CI: 0.3, 11.0) per 1000 pregnancies; however the number of studies from that region in the review were limited

(212). Importantly Creswell et al. (212) include both placenta praevia (complete coverage of the internal os) and low-lying placenta (where the placental edge is within 20mm of the internal os) within their study definition. Despite the low incidence, the dangers associated with placenta praevia are high, with an estimated 51.6% (95% CI 42.7, 60.6) of pregnant women with placenta praevia experiencing APH (199). Placental abruption appears to occur at a higher rate than placenta praevia, with most studies estimating an incidence ranging from 0.5 to 1.0% of births being complicated by abruption across multiple different settings (202).

As discussed in Table S47, we were unable to identify data sources reporting the incidence of either placental abruption or placenta praevia in Malawi. However, as with maternal sepsis, the number of APH cases is captured in the 2015 EmONC needs assessment survey which, when divided by the approximated births for the survey year, leads to a rate of 4.6 APH cases per 1000 births (33).

3.1.9.2 Model

Figures S19 and S20 describe the model of APH and Table S47 describes the relevant parameters.

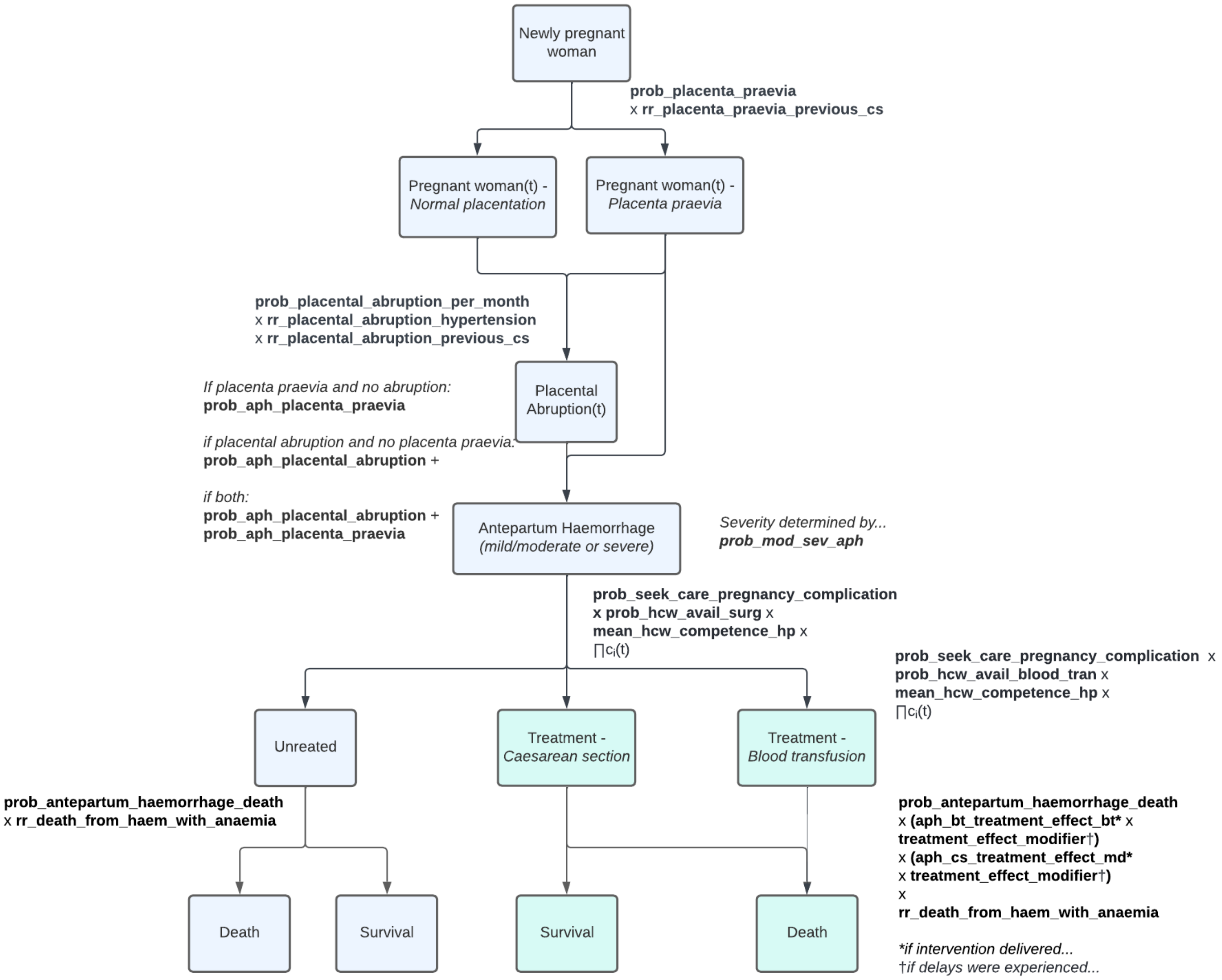

Figure S19 – Model of antepartum haemorrhage
Diagrammatic representation of the model of antepartum haemorrhage. Light blue represents the model's natural history without treatment whilst teal represents treatment pathways. Parameters representing progression through model states are shown here.

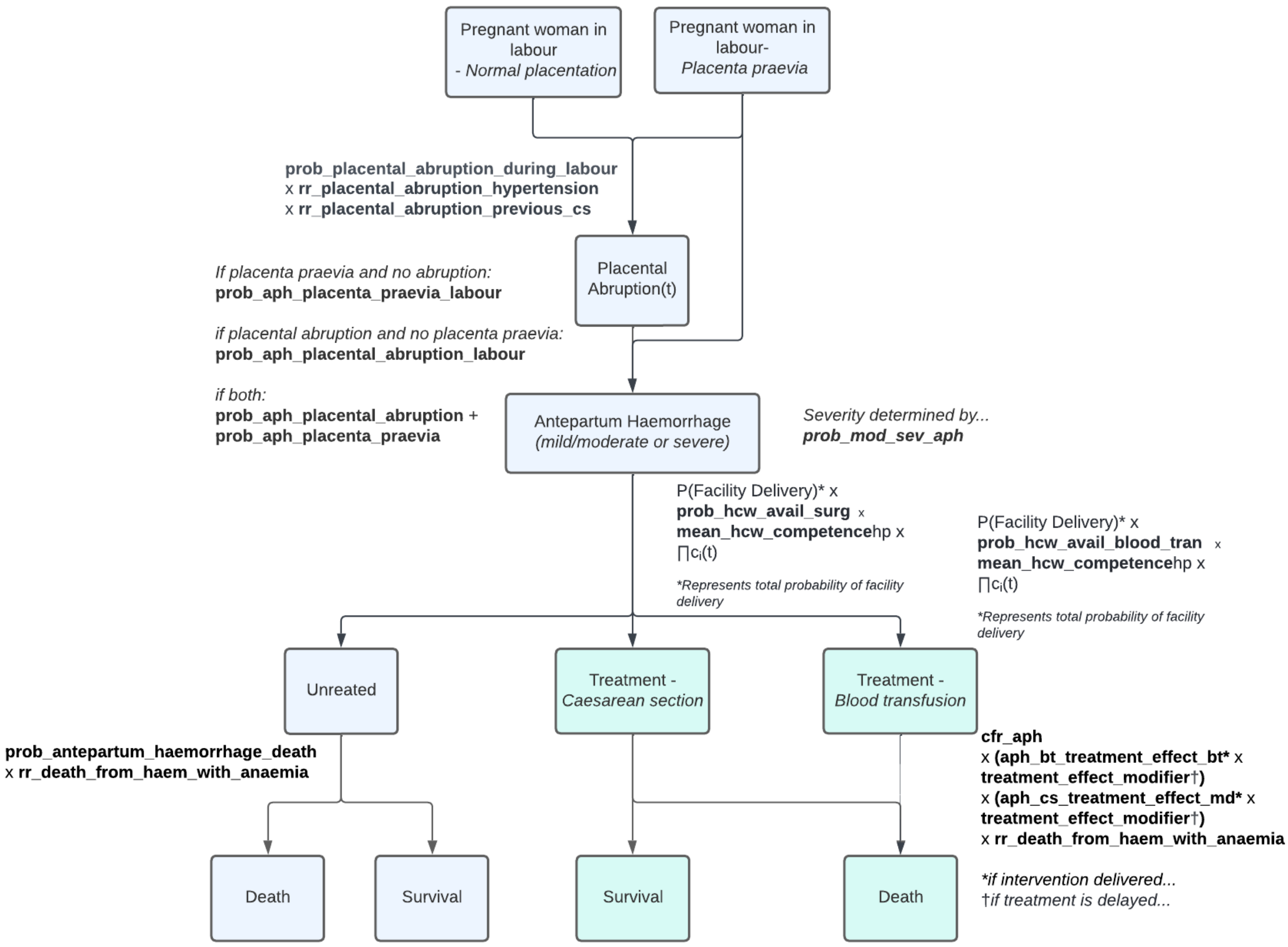

Figure S20 – Model of intrapartum haemorrhage
Diagrammatic representation of the model of intrapartum haemorrhage. Light blue represents the model's natural history without treatment whilst teal represents treatment pathways. Parameters representing progression through model states are shown here.

A single per-pregnancy risk of a woman developing placenta praevia is applied at the start of each pregnancy calculated via the following equation:

$$Y = \textbf{prob\_placenta\_praevia} * (ps\_prev\_cs * \textbf{rr\_placenta\_praevia\_previous\_cs}) \tag{18}$$

This leads to an incidence of 5 cases per 1000 births within the modelled population, which is supported by a large historical cohort study conducted in Tanzania which was selected due to lacking data from Malawi and its proximity as a bordering nation (200). Many of the studies reporting on incidence and outcomes of placenta praevia that were used within

model development still rely on outdated definition of placenta praevia, including 'grading' of severity in relation to proximity to the internal os (199,200,212). Because of this we have opted to assume that women with placenta praevia are at risk of APH, but that APH is not a definite outcome of all cases carried to term and delivered vaginally (i.e. not all cases cover the os entirely).

Unlike placenta praevia, risk of placental abruption is applied at several time steps during pregnancy. Antenatally this risk is applied every month of pregnancy from month five, and then additionally risk is applied once during the intrapartum period. At each time step, individual risk is calculated as:

$$Y_{(t)} = \mathbf{prob\_placental\_abruption} * (ps\_prev\_cs * \mathbf{rr\_placental\_abruption\_previous\_cs}) * (ps\_htn\_disorders * \mathbf{rr\_placental\_abruption\_hypertension}) \quad (19)$$

The modelled incidence of placental abruption, 3 per 1000 births, is also taken from a Tanzanian study using a large historical cohort due to limited data from Malawi (210). In addition to having previously delivered via CS (206), maternal hypertension caused by any hypertensive disorders of pregnancy (§3.1.10) has been identified as a predictor of placental abruption (210) as seen in equation 19.

As evident from Figures S19 and S20 and Table S47, only women who have experienced these antecedent conditions are at risk of developing APH. From month five of pregnancy, also following a monthly time step, and during labour the risk of APH secondary to placental abruption (**prob_aph_placental_abruption**), or placenta praevia, (**prob_aph_placenta_praevia)**, is applied to all women with these condition to a rate of 4.6 cases per 1000 births as discussed in Table S47. Severity of bleeding is determined via the parameters **prob_mod_sev_aph** / **severity_maternal_haemorrhage** with severity used to guide antenatal treatment and to map to relevant DALY weights.

Prior to labour, women with APH may choose to seek emergency antenatal inpatient care, after which treatment is delivered reducing overall risk of death. Women who develop intrapartum bleeding are liable to receive treatment if they are already delivering in a health facility; individuals during labour at home may seek care similarly to other intrapartum complications in the model. Risk of death is applied to all women who experience APH, using the parameters shown in Table S47 with risk mitigated by treatment. At present, severity of bleeding does not impact risk of death as case fatality estimates to which the model is calibrated do not specify mortality by severity. However, women who are anaemic at the time of haemorrhage are at greater risk of death, with parameter **rr_death_from_haem_with_anaemia** representing that risk.

3.1.9.2.1 Treatment

For cases of haemorrhage occurring prior to labour onset, treatment delivered to mothers with APH in Malawi varies according to the underlying aetiology and severity of the bleeding, yet in all cases CS is indicated to prevent risk of maternal and perinatal death (32). In keeping with these guidelines, individuals with APH due to placental abruption are scheduled for immediate CS delivery regardless of the severity of bleeding. Individuals with APH due to placenta praevia with non-severe bleeding are scheduled to undergo CS once they reach 37 weeks GA, whilst severe bleeding cases are scheduled for immediate delivery. In addition, all haemorrhage cases receive blood transfusion after bleeding onset.

Both CS and blood transfusion are modelled to reduce risk of maternal death. If CS is performed the parameter **aph_cs_treatment_effect_md** represents the effect of this intervention on risk of death as the aetiology of bleeding is placental; delivery of the placenta is therefore assumed to stop ongoing bleeding and reduce risk of death. In addition, the parameter **aph_bt_treatment_effect_md** is the effect of blood transfusion on risk of maternal death due to bleeding.

3.1.9.3 Data sources and parameters

Parameter Name**	Description	Value*	Source and/or relevant calculation
**prob_placenta_praevia**	This parameter is scaled at intialisation of the simulation to account for the proportion of women at baseline who have previously delivered via CS.<br><br>Once scaled, as the simulation runs this parameter is the probability that a newly pregnant woman, who has never previous delivered via CS, has developed placenta praevia.	0.005 / 0.0058	Due to lacking data on the incidence of placenta praevia within Malawi the rate used in the model is sourced from a study conducted in Tanzania in which a rate of 5 cases per 1000 births was identified (200). This study was chosen due to the geographic proximity to Malawi and the large sample size of over 47,000 deliveries. This parameter has been derived from calibration to this rate.
**rr_placenta_praevia_previous_cs**	The effect of a woman having ever previously delivered via CS on her risk of developing placenta praevia	1.47	Sourced directly from Yang et al. (206) who report the association of previous caesarean delivery and placenta praevia in a sample of over five million deliveries in the US.
**prob_placental_abruption_per_month**	This parameter is scaled at intialisation of the simulation to account for the proportion of women at baseline who have previously delivered via CS.<br><br>Once scaled, as the simulation runs this parameter is the probability that a pregnant woman, who is normotensive and has never previously delivered via CS, will experience placental abruption applied during months 5-9 of pregnancy.	0.0005	Due to lacking data on the incidence of placental abruption within Malawi the rate used in the model is sourced from a study conducted in Tanzania in which a rate of 3 cases per 1000 births was identified (210). Again, this study was chosen due to the geographic proximity to Malawi and the large sample size of nearly 40,000 deliveries. This parameter has been derived from calibration to this rate.

**rr_placental_abruption_hypertension**	The effect of a pregnant woman having a hypertensive disorder of pregnancy on her risk of experiencing placental abruption	2.2	See **prob_placental_abruption_per_month**. Sourced directly from Macheku et al. (210).
**rr_placental_abruption_previous_cs**	The effect of a pregnant woman having previously delivered via CS on her risk of experiencing placental abruption.	1.3	see **rr_placental_abruption_hypertension.**
**prob_aph_placenta_praevia**	The probability that a pregnant woman with placenta praevia will develop an APH applied during months 5-9 of pregnancy.	0.09	As with the number of maternal sepsis cases, the number of APH cases during the survey period were captured in the 2015 Malawi EmONC needs assessments (33). This rate is calculated by dividing the total number of observed APH cases assessment survey by the estimated number of births leading to a rate of APH within the pregnant population of approximately 4.6 cases per 1000 births.<br><br>Due to the structure of the model this parameter and parameter **prob_aph_placental_abruption** drive the total rate of APH and therefore have been derived through calibration to the total rate of APH.
**prob_aph_placental_abruption**	The probability that a pregnant woman with placental abruption will develop APH applied during months 5-9 of pregnancy.	0.9	See **prob_aph_placenta_praevia.** It is assumed that the probability of APH secondary to placental abruption is high in keeping with evidence (210).
**prob_mod_sev_aph/ severity_maternal_haemorrhage**	The probabilities that woman who is experiencing APH will experience mild/moderate or severe bleeding.	[0.8, 0.2]	Due to lacking epidemiological data on the severity of APH this parameter has been approximated under the assumption that mild cases of bleeding are more common than severe cases.

**prob_antepartum_haemorrhage_death/ cfr_aph**	The probability that a pregnant woman will die due to APH without treatment.	0.11 / 0.04	See **prob_ectopic_pregnancy_death** in Table S39**.**
**rr_death_from_haem_with_anaemia**	The effect of maternal anaemia on a woman's risk of dying from a haemorrhage	1.5	The value of this parameter is assumed. Whilst there is evidence to suggest anaemia is associated with mortality (122) we were unable to find a reliable estimate for the effect of anaemia on haemorrhage related mortality.
**aph_bt_treatment_effect_md**	The effect of blood transfusion on risk of maternal death due to APH	0.4	Pollard et al. (37) estimate the effect of interventions on maternal death due to APH. The effectiveness of 'CEmONC' services on APH deaths is reported as 90%. These services are assumed to consist of surgery and blood transfusion. As such, blood transfusion effect is assumed to be 0.4 and the surgical effect is 0.25. (0.25 x 0.4 = 0.1 (90% effective))
**aph_cs_treatment_effect_md**	The effect of CS delivery on risk of maternal death due to APH	0.25	See **aph_bt_treatment_effect_md.**
**prob_placental_abruption_during_labour**	The probability that a pregnant woman in labour will experience placental abruption.	0.0005	*See* **prob_placental_abruption_per_month**.
**prob_aph_placenta_praevia_labour**	The probability of APH during labour for women with placenta praevia.	0.25	See **prob_aph_placenta_praevia**.

**prob_aph_placental_abruption_labour**	The probability of APH during labour for women with placental abruption.	0.9	See **prob_aph_placental_abruption**.

** Where two values (or sets of values) are provided the first set is applied from 2010-2014 and the second set from 2015 onwards for a given simulation run (§1.2.1.1)*

*** If two names are provided for the same parameter this means the name varies by python file.*

Table S48 – Parameters of the antepartum and intrapartum haemorrhage model

3.1.10 The Hypertensive Disorders of Pregnancy

3.1.10.1 Condition overview

The ‘hypertensive disorders of pregnancy’ (HDP) are a complex group of maternal health conditions associated with hypertension during or following pregnancy. These conditions include chronic hypertension, pre-eclampsia (inclusive of all stages, including cases superimposed on chronic hypertension) and gestational hypertension (214). Maternal hypertensive disorders that onset antenatally may resolve following delivery, persist into the postnatal period or as suggested by significant epidemiological evidence, onset de novo within the postnatal period (215–217). This has been reflected in the representation of the HDPs within the MPHM as described below. Of note, chronic hypertension is not discussed here as the incidence of this condition is managed by the Cardio-metabolic disease module and is not governed by this model.

In Malawi, pre-eclampsia is defined clinically as a maternal blood pressure of 140-150/90-109 mmHg onsetting after 20 weeks GA in the presence of proteinuria (32). The aetiology of pre-eclampsia is not well understood, however contemporary pathogenic research suggests that the condition occurs following ‘defective’ spiral artery remodelling, those which supply nutrients to the placenta, leading to ischaemia within placental cells driving an imbalance between anti- and pro-angiogenic factors (218,219). Following a review of the literature in SSA, the predictors deemed to have causal influence on risk of pre-eclampsia include obesity (145), multiple pregnancy (220), hypertension prior to pregnancy (221), diabetes mellitus (222) and calcium supplementation delivered as part of ANC (44).

Without delivery of the foetus and placenta, progression to a more severe form of the disease is possible, most notably severe pre-eclampsia, defined clinically as a maternal blood pressure of 160/110 mmHg or higher onsetting after 20 weeks’ gestation in the presence of severe proteinuria. Potential diagnostic signs include severe headaches and visual disturbances (32). In SSA, and many other parts of the world, untreated severe pre-eclampsia, and eclampsia, where tonic-clonic seizures occur, can lead to severe consequences for the mother and newborn, including maternal death and stillbirth (223).

Gestational hypertension shares a similar clinical definition to pre-eclampsia, however, is characterised by new hypertension in pregnancy or the postnatal period in the absence of proteinuria (32). Evidence suggests that the two conditions are distinct, due to variations in predictive factors (224), and progression from gestational hypertension to pre-eclampsia is possible and does occur in some women (225). As with pre-eclampsia, gestational hypertension can develop into severe disease which can lead to poor maternal and perinatal outcomes (226,227).

Hypertension during pregnancy is one of, if not, the most common complications experienced by mothers globally, with historic and contemporary data sources suggesting that the global incidence is rising leading to an estimated 18.08 million (95 % UI 15.26, 21.11 million) cases occurring in 2019 (228). As with many of the conditions discussed thus far, incidence appears to vary considerably between nations and regions with a greater number of cases occurring in SSA (228). A large systematic review exploring the prevalence of HDP by sub-condition in Africa reported an overall prevalence of HDP of 100.4 per 1000 pregnant or postnatal women (95% CI 81.4, 121.2), the prevalence of gestational hypertension of 49.8 (95% CI 32.3, 70.7), the prevalence of pre-eclampsia (non-severe) of 44.0 (95% CI 36.7, 52.0), prevalence of severe pre-eclampsia of 22.1 (95% CI 14.8, 30.8) and prevalence of eclampsia of 14.7 (95% CI 8.1, 23.2), suggesting a considerable burden in the region (229).

3.1.10.2 Model

Figure S21 describes the model of gestational hypertension whilst Figures S22 and S23 represent the pre-eclampsia model

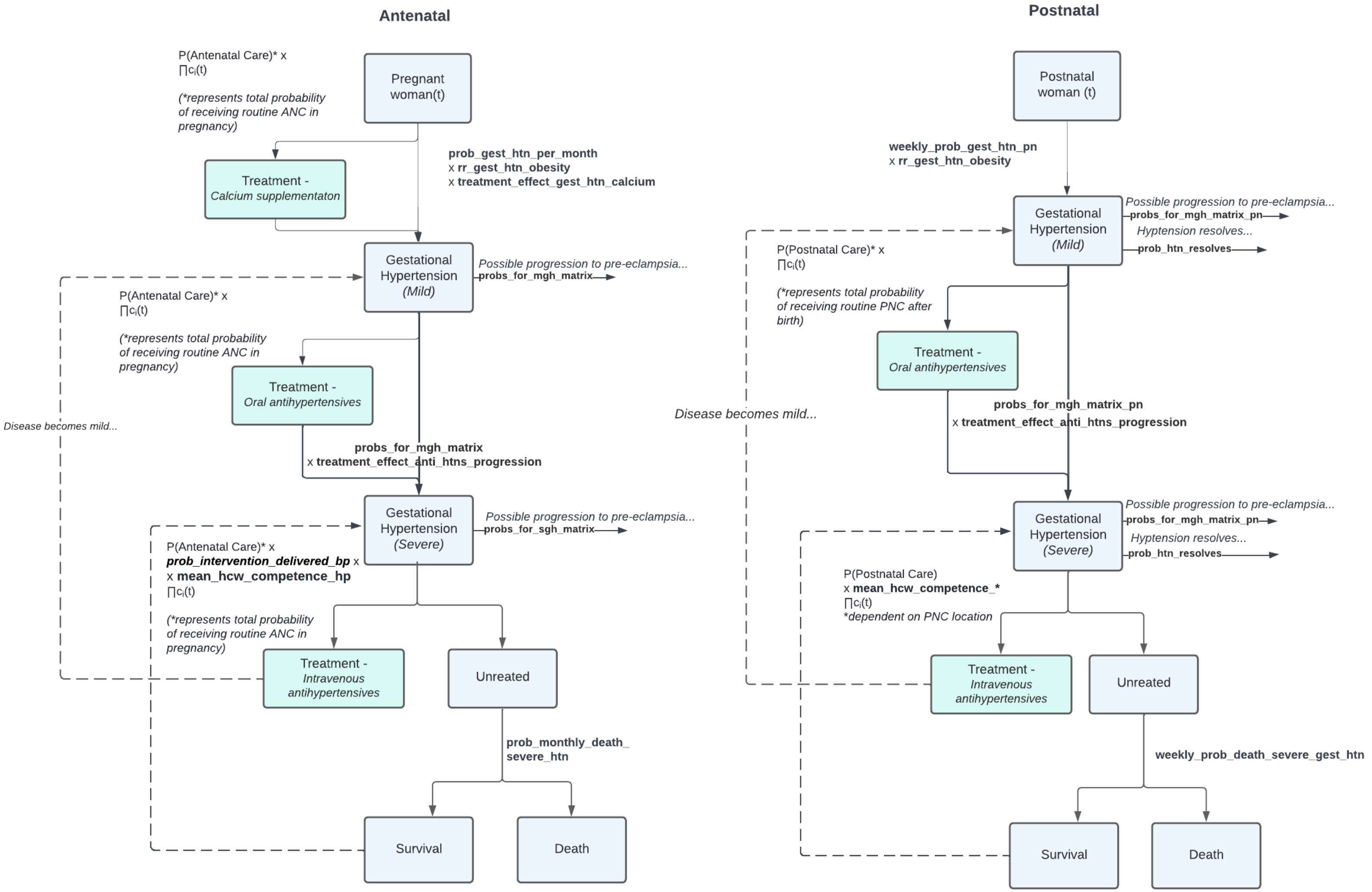

Figure S21 -Model of gestational hypertension

Diagrammatic representation of the model of gestational hypertension. Light blue represents the model's natural history without treatment whilst teal represents treatment pathways. Parameters representing progression through model states are shown here.

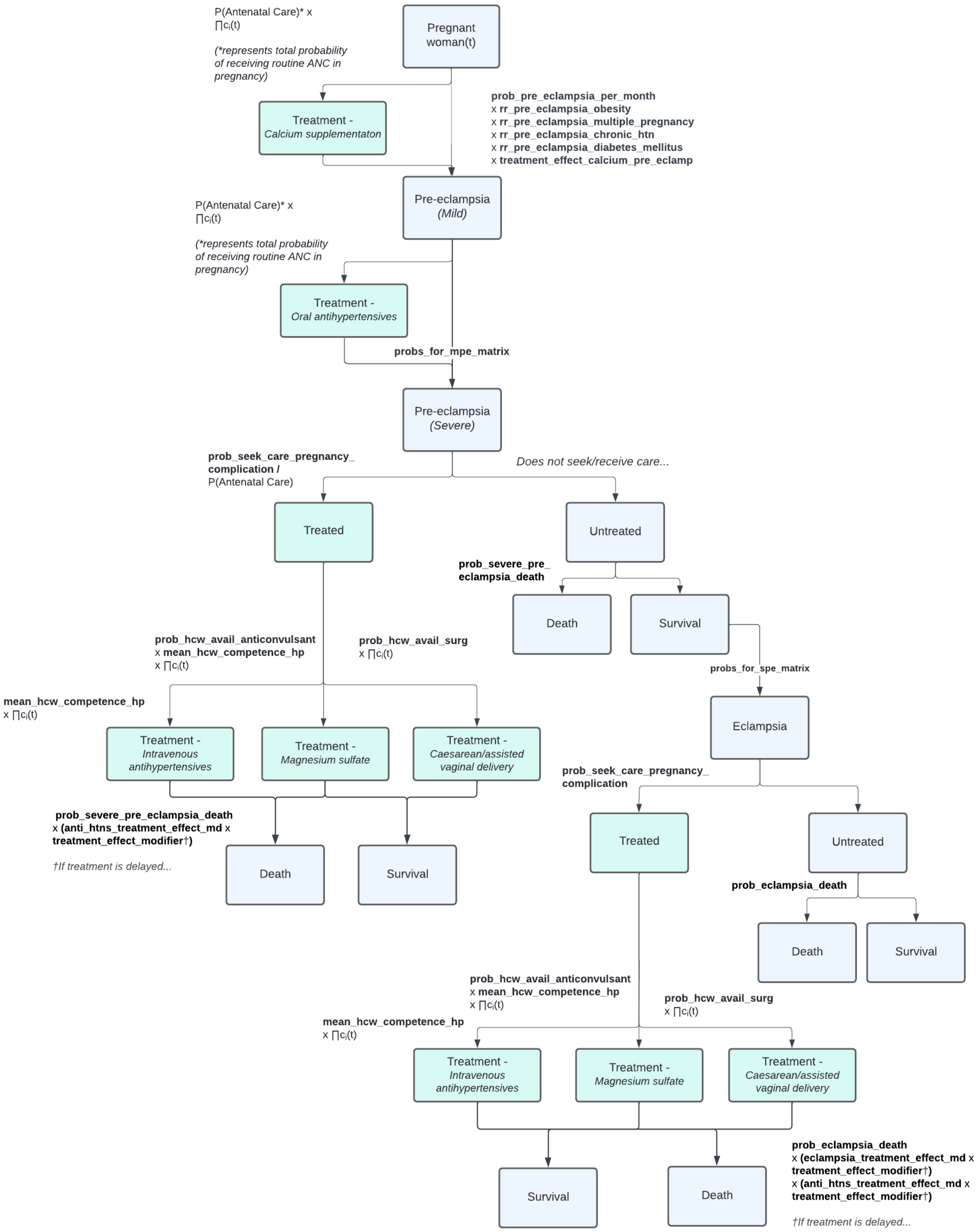

Figure S22 – Model of pre-eclampsia (antenatal)

Diagrammatic representation of the model of antenatal pre-eclampsia. Light blue represents the model's natural history without treatment whilst teal represents treatment pathways. Parameters representing progression through model states are shown here.

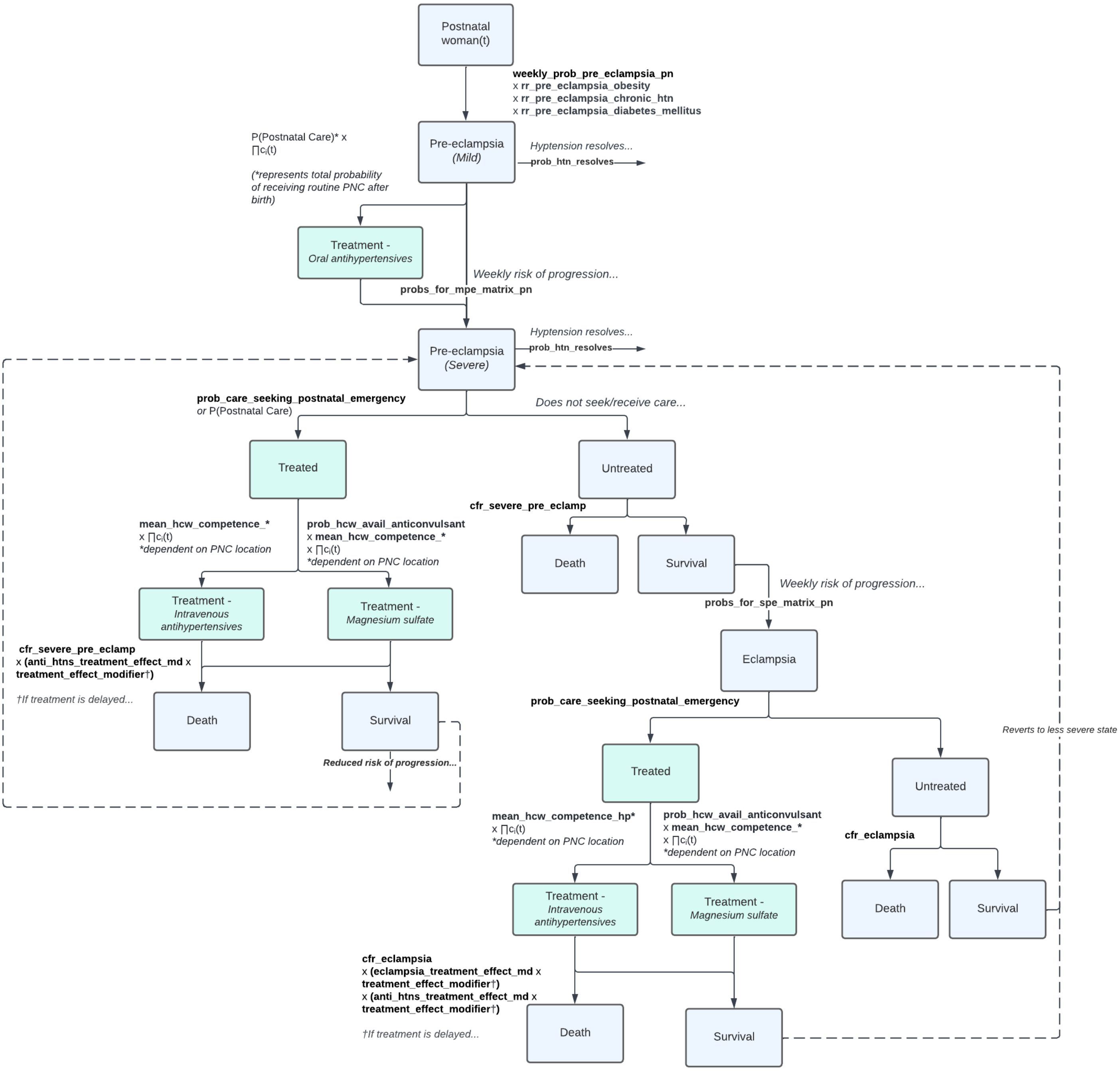

Figure S23 – Model of pre-eclampsia (postnatal)

Diagrammatic representation of the model of postnatal pre-eclampsia. Light blue represents the model's natural history without treatment whilst teal represents treatment pathways. Parameters representing progression through model states are shown here.

Gestational hypertension

During pregnancy, from 22 weeks GA a monthly risk of mild gestational hypertension is applied to all pregnant women calculated as:

$$Y_{(t)} = \mathbf{prob\_gest\_htn\_per\_month}_{(t)} * (li\_bmi\_25\_plus * \mathbf{rr\_gest\_htn\_obesity}) * (ac\_receiving\_calcium\_supplements * \mathbf{treatment\_effect\_gest\_htn\_calcium}) \quad (20)$$

In addition to the effect of BMI, receipt of daily calcium supplementation also reduces individual risk of gestational hypertension acquisition (44) which is delivered to mothers during ANC shown in Figure S21. In this model it is assumed that *either* gestational hypertension or pre-eclampsia can onset in an individual during a given month of pregnancy, as there is extensive discussion in the literature surrounding the distinction between the predictors and outcomes of both conditions (224,230).

Following the onset of gestational hypertension, an individual is at risk that their disease might progress into a more severe state, with risk of progression applied monthly for the remainder of the pregnancy. Gestational hypertension is assumed to be mild at onset and an individual with mild disease is at risk of progression to mild pre-eclampsia (225) *or* severe gestational hypertension every month.

The parameter **probs_for_mgh_matrix,** described in Table S48, contains the probabilities that an individual will change disease state (or remain in the same state) each month. The probabilities employed within this matrix were derived through calibration to the assumed rate of each form of severe disease within the population as discussed in the table. During the antenatal period, it is assumed that progression is linear in all cases and individuals in the model do not revert to a less severe disease state. Due to this logic the incidence of severe gestational hypertension disease is only generated through the process of progression. In addition, in both the antenatal and postnatal period, risk of progression is reduced in women with mild disease who have been started on regular oral antihypertensives following ANC screening as described further below.

During the postnatal period women may remain hypertensive following birth or develop de novo gestational hypertension. The parameter **prob_htn_resolves** is used to determine if antenatal disease will resolve, otherwise hypertension persists. A similar approach to disease onset is employed during the postnatal period, with a weekly risk of de novo gestational hypertension acquisition applied to all postnatal mothers calculated as:

$$Y_{(t)} = \mathbf{weekly\_prob\_gest\_htn\_pn}_{(t)} * (li\_bmi\_25\_plus * \ \mathbf{rr\_gest\_htn\_obesity}) \quad (21)$$

Weekly risk of disease progression is applied following the same methodology as described above however, the parameter **prob_htn_resolves** is also applied weekly to all women with postnatal hypertension to determine if their condition will resolve during this time. All cases resolve automatically at the end of the postnatal period.

Neither mild nor severe gestational hypertension will lead to care seeking during either the antenatal or postnatal period due to being largely symptomless (219). Therefore, as with GDM, initiation of treatment is dependent entirely on screening when an individual interacts with the health service via a HSI event. Additionally, it is assumed that only women experiencing severe disease have an increased risk of death. Antecedent causes of death associated with severe hypertension are not modelled explicitly (e.g. stroke), instead a weekly or monthly risk of death (dependent on time point in pregnancy period), indicated by the parameters **weekly_prob_death_severe_gest_htn** and **prob_monthly_death_severe_htn** is applied to all women in the severe state due to clinical evidence of mortality in women with severe hypertension without characteristics of pre-eclampsia (231).

Pre-eclampsia

As evident from Figures S22 and S23 the method of application of risk of pre-eclampsia both in the antenatal and postnatal period follows a similar pattern to gestational hypertension. Individual risk of antenatal onset at time *t* is calculated as follows:

$$Y_{(t)} = \mathbf{prob\_pre\_eclampsia\_per\_month}_{(t)} * (li\_bmi\_25\_plus * \mathbf{rr\_pre\_eclampsia\_obesity}) * (ps\_multiple\_pregnancy * \mathbf{rr\_pre\_eclampsia\_multiple\_pregnancy}) * (nc\_diabetes * \mathbf{rr\_pre\_eclampsia\_diabetes\_mellitus}) * (nc\_hypertension * \mathbf{rr\_pre\_eclampsia\_chronic\_htn}) * (ac\_receiving\_calcium\_supplements * \mathbf{treatment\_effect\_calcium\_pre\_eclamp})$$

(22)

Whilst weekly risk in the postnatal period is calculated as:

$$Y_{(t)} = \mathbf{weekly\_prob\_pre\_eclampsia\_pn}_{(t)} * (li\_bmi\_25\_plus * \mathbf{rr\_pre\_eclampsia\_obesity}) * (nc\_diabetes * \mathbf{rr\_pre\_eclampsia\_diabetes\_mellitus}) * (nc\_hypertension * \mathbf{rr\_pre\_eclampsia\_chronic\_htn})$$

(23)

As with gestational hypertension, when pre-eclampsia onsets in the model it is assumed to be mild, and women face either a weekly or monthly risk of disease progression depending on time point. Resolution of diseases may occur as in the same manner as described above. Mild pre-eclampsia, like mild gestational hypertension, is not assumed to trigger possible care seeking, and therefore treatment is only delivered during routine care. However, on progression to severe disease (either severe pre-eclampsia or eclampsia) a probability of care seeking is applied. In the case of severe pre-eclampsia or eclampsia, following potential care seeking and receipt of treatment, risk of death parameters **prob_severe_pre_eclampsia_death/ cfr_severe_pre_eclamp** and **prob_eclampsia_death/ cfr_eclampsia** respectively are applied with treatment mitigating risk.

3.1.10.2.1 Treatment

Gestational hypertension – Mild disease

Pregnant women are screened for hypertension during ANC via blood pressure measurement (Table S24). If hypertension is detected in the absence of proteinuria (tested via urine dipstick) then the mother is initiated on oral antihypertensives (31,32), which act in the model to reduce risk of progression from mild to severe disease in women with gestational hypertension (56). The parameter **treatment_effect_anti_htns_progression** is

the effect of this treatment on risk of progression. Similarly, hypertensive women during the postnatal period are initiated on antihypertensives following attendance of PNC which have the same effect.

Severe gestational hypertension

Women with severe hypertension detected either in ANC, facility delivery or PNC are admitted (if not already an inpatient) and administered intravenous (IV) antihypertensives in keeping with Malawian guidelines (31,32). In the model, administration of IV antihypertensives is assumed to reset maternal disease state to mild, circumnavigating the monthly/weekly risk of death associated with severe gestational hypertension.

Pre-eclampsia – mild disease

Screening for pre-eclampsia occurs in antenatal and postnatal care. In keeping with Malawian guidelines women with mild pre-eclampsia are also indicated to receive oral antihypertensive treatment which is initiated in the model. However, Abalos et al. (56) found no effect on progression from mild to severe disease so we do not include an effect in the model and this treatment is therefore included to map consumable use.

Severe pre-eclampsia and Eclampsia

The delivery of intravenous and/or intramuscular magnesium sulphate ($MgSO_4$) is the primary treatment indicated for severe pre-eclampsia and eclampsia and in this context. $MGSO_4$ is an effective anticonvulsant shown to halve the risk of eclamptic seizures in women with severe pre-eclampsia (57). In most settings, including Malawi, delivery of $MGSO_4$ is indicated in all cases of either severe pre-eclampsia or eclampsia alongside the delivery of intravenous antihypertensives (32,232).

In the model, women with severe pre-eclampsia who receive $MgSO_4$ are at reduced risk of progression to eclampsia following administration (Figures S22 and S23). Parameter **eclampsia_treatment_effect_severe_pe** in Table S48 is the effect of this treatment on the risk of progression. In addition, these women should receive intravenous antihypertensives which reduces risk of maternal death as shown in parameter **anti_htns_treatment_effect_md.** Women who receive healthcare whilst experiencing

eclampsia may receive $MGSO_4$ and antihypertensives which reduce risk of maternal death secondary to eclampsia – **eclampsia_treatment_effect_md** and **anti_htns_treatment_effect_md** respectively. In addition, in line with clinical guidance, and to map the relationship between these conditions and the overall rate of instrumental and operative delivery, the parameters **prob_delivery_modes_spe** and **prob_delivery_modes_ec** determine the mode of delivery for cases of these conditions assuming that CS is the primary route of delivery due the severity of the condition and risk to maternal and perinatal life.

3.1.10.3 Data sources and parameters

Parameter Name**	Description	Value*	Source and/or relevant calculation
Gestational hypertension parameters			
**prob_gest_htn_per_month**	This parameter is scaled at intialisation of the simulation to account for the proportion of women at baseline who have a BMI over 25.<br><br>Once scaled, as the simulation runs this parameter represents the probability that a pregnant woman with a BMI of 25 or less will develop mild gestational hypertension per month of the pregnancy starting at month 5.	0.0073	Due to limited data on gestational hypertension rates in Malawi the assumed prevalence was sourced from a systematic review of African studies estimating prevalence of the disease by Noubiap et al. (229). This review included a mix of studies estimating prevalence during and following pregnancy, so any estimates are assumed to be for the condition across the entire pregnancy continuum. The authors estimated a pooled prevalence of 49.8 cases of gestational hypertension per 1000 pregnant or parturient women. This parameter was therefore derived through calibration to this rate.<br><br>In the model 88% of these cases are assumed to be mild, using an estimate from a study conducted in the USA (226) with the remaining being severe.<br><br>In addition, 70% of all HDP cases (GH and PE) are assumed to occur antenatally and 30% postnatally in the model. Reliable estimates of the total proportion of gestational hypertension which onsets before or after delivery are unavailable however consensus exists that antenatal cases are more common (233,234).<br><br>Adjusting the initial rate leads to an assumed rate of mild gestational hypertension of 43.8 per 1000 pregnant or parturient women (30.7 cases per 1000 occurring antenatally and 13.1 cases per 1000 occurring postnatally).

			This parameter was estimated as (30.7/1000) / 5 (months risk is applied) to give 0.0061. This parameter was then manipulated to achieve the desired rate in the model accounting for pregnancy loss in the population and the effect of predictors/treatment.
**weekly_prob_gest_htn_pn**	The probability that a postnatal woman with a BMI of 25 or less will develop mild gestational hypertension per week of the postnatal period	0.0025	see **prob_gest_htn_per_month.** (13.1/1000) / 7 = 0.0019. Manipulation then occurred during calibration.
**rr_gest_htn_obesity**	The effect of a pregnant or postnatal woman being obese (BMI>25) compared to normal weight (BMI 18.5-24.9) on her risk of developing antenatal or postnatal gestational hypertension	3.31	Sourced directly from Santos et al. (145) who report the effect of obesity on risk of gestational hypertension from an individual patient data meta-analysis over a quarter of a million pregnancies in HICs.
**prob_monthly_death_severe_htn**	The probability that a pregnant woman with severe gestational hypertension will die per month of the antenatal period	0.0001	Assumption as data from Malawi reporting deaths from severe gestational hypertension was unavailable. Due to the small total probability of death and the relatively low incidence of severe gestational hypertension between 0.5 and 1 deaths per 100,000 live births per year are attributable to this cause in the model.
**weekly_prob_death_severe_gest_htn**	The probability that a postnatal woman with severe gestational hypertension will die per week of the postnatal period	0.00002	See prob**_monthly_death_severe_htn.**

Pre-eclampsia parameters			
**prob_pre_eclampsia_per_month**	This parameter is scaled at intialisation of the simulation to account for the proportion of women at baseline who have a BMI over 25, chronic hypertension or diabetes.<br><br>Once scaled, as the simulation runs this parameter represents the probability that a pregnant woman, with BMI <=25, without diabetes mellitus, without chronic hypertension and without a twin pregnancy will develop mild pre-eclampsia per month of the antenatal period.<br><br>The parameters below starting with "rr_pre_eclampisa" refer to the effect on the probability of pre-eclampsia onset	0.0065	See **prob_gest_htn_per_month**. From Noubiap et al. (229) the total pooled prevalence of mild pre-eclampsia was 44 cases per 1000 pregnant or parturient women. It is assumed 30.8 per 1000 cases occur antenatally.<br><br>(30.8/1000) * 5 = 0.0062. Manipulated during calibration.
**weekly_prob_pre_eclampsia_pn**	The probability that a postnatal woman, with BMI <=25 and without diabetes mellitus will develop mild pre-eclampsia per week of the postnatal period	0.00198	See **prob_gest_htn_per_month.** From Noubiap et al. (229) the total pooled prevalence of mild pre-eclampsia was 44 cases per 1000 pregnant or parturient women. It is assumed 13.2 per 1000 cases occur postnatally. (13.2/1000) / 7 = 0.0019. Manipulated during calibration.
**rr_pre_eclampsia_multiple_pregnancy**	The effect of a pregnant woman carrying a twin pregnancy	4.07	Sourced directly from Laine et al. (220) who estimate the effect of twin pregnancy on risk of pre-eclampsia via multivariable logistic regression with data on over 16,000 twin pregnancies.

**rr_pre_eclampsia_obesity**	The effect of a pregnant or postnatal woman being obese (BMI>25) compared to normal weight (BMI 18.5-24.9).	3.2	See **rr_gest_htn_obesity**
**rr_pre_eclampsia_chronic_htn**	The effect of a pregnant or postnatal woman having hypertension that onset prior to pregnancy compared to no hypertension	2.26	Sourced directly from Meazaw et al. (221) who estimate the effect of several determinants, including chronic hypertension, on the risk of pre-eclampsia via a systematic review and meta-analysis of studies conducted in sub-Saharan Africa.
**rr_pre_eclampsia_diabetes_mellitus**	The effect of a pregnant or postnatal woman having diabetes mellitus that onset prior to pregnancy	3.7	Sourced directly from Bartsch et al (222) who estimate the effect of several clinical risk factors on risk of pre-eclampsia via a systematic review and meta-analysis of cohort studies leading to a sample of over 25 million pregnancies.
**prob_severe_pre_eclampsia_death / cfr_severe_pre_eclamp**	The probability that a pregnant or postnatal woman will die due to severe pre-eclampsia without treatment.	0.018	See **prob_ectopic_pregnancy_death** in Table S39**.**
**prob_eclampsia_death / cfr_eclampsia**	The probability that a pregnant or postnatal woman will die due to eclampsia that has onset during labour without treatment.	0.028 / 0.03	See **prob_ectopic_pregnancy_death** in Table S39**.**

Parameters relating to disease progression			
**probs_for_mgh_matrix / probs_for_mgh_matrix_pn**	The probabilities that a woman with antenatal or postnatal mild gestational hypertension will have their disease state newly classified as mild gestational hypertension (no change), severe gestational hypertension, mild pre-eclampsia, severe pre-eclampsia, or eclampsia respectively.<br><br>Values of 0 suggest change between disease state is not possible.	[0.918, 0.032, 0.05, 0, 0]	These probabilities are derived entirely through calibration to the overall assumed rates of the key hypertensive disorders within the model. The rates for mild pre-eclampsia and mild gestational hypertension are discussed in parameters **prob_gest_htn_per_month** and **prob_pre_eclampsia_per_month** in this table sourced from Noubiap et al. (229) and Vousden et al. (235) – who estimated the incidence of eclampsia in Malawi.
**probs_for_sgh_matrix / probs_for_sgh_matrix_pn**	See **probs_for_mgh_matrix.** This parameter represents probabilities of progression for severe gestational hypertension.	[0, 0.87, 0, 0.13, 0] / [0, 0.92, 0, 0.08, 0]	See **probs_for_mgh_matrix** and **prob_gest_htn_per_month.** The total rate of SGH in the model is 5.9 per 1000 births. This is equal to 12% of the total GH rate reported in Noubiap et al. (229). The assumed antenatal rate is 4.2 per 1000 (70% total rate) with the remaining cases occurring postnatally.
**probs_for_mpe_matrix / probs_for_mpe_matrix_pn**	See **probs_for_mgh_matrix.** This parameter represents probabilities of progression for mild pre-eclampsia.	[0, 0, 0.78, 0.22, 0] / [0, 0, 0.95, 0.05, 0]	See **probs_for_mgh_matrix**.
**probs_for_spe_matrix / probs_for_spe_matrix_pn**	See **probs_for_mgh_matrix.** This parameter represents probabilities of progression for severe pre-eclampsia	[0, 0, 0, 0.16, 0.84] / [0, 0, 0.95, 0.05, 0]	See **probs_for_mgh_matrix** and **prob_pre_eclampsia_per_month.** The total rate of SPE in the model is 22 per 1000 births as reported in (229). The assumed antenatal rate is 15.4 per 1000 (70% total rate) with the remaining cases occurring postnatally.

**probs_for_ec_matrix / probs_for_ec_matrix_pn**	See **probs_for_mgh_matrix.** This parameter represents probabilities of progression for eclampsia.	[0, 0, 0, 0, 1]	See **probs_for_mgh_matrix** and **prob_pre_eclampsia_per_month.** The total rate of eclampsia in the model is 10 per 1000 births as reported in Vousden et al. (235). The assumed antenatal rate is 7 per 1000 (70% total rate) with the remaining cases occurring postnatally.
**prob_progression_gest_htn**	The probability that a pregnant woman in labour with mild gestational hypertension will progress to severe gestational hypertension.	0.036	See **probs_for_mgh_matrix.**
**prob_progression_severe_gest_htn**	See **prob_progression_gest_htn.**	0.13	See **probs_for_sgh_matrix.**
**prob_progression_mild_pre_eclamp**	See **prob_progression_gest_htn.**	0.14	See **probs_for_mpe_matrix.**
**prob_progression_severe_pre_eclamp**	See **prob_progression_gest_htn.**	0.29	See **probs_for_spe_matrix.**
**prob_htn_resolves**	The weekly probability that a postnatal experiencing one of the hypertensive disorders will no longer experience hypertension and her disease will have resolved	0.167	This parameter has been estimated under the assumption that all hypertensive disorders will end at the end of the postnatal period. Therefore, a probability of 1 was divided by the total weeks to approximate weekly a resolution rate as HPDs resolve at the end of the postnatal period automatically.
Treatment effects and other healthcare parameters			
**treatment_effect_gest_htn_calcium**	The effect of daily calcium supplementation on risk of gestational hypertension onset during the antenatal period	0.65	Sourced directly from Hofmeyr et al. (44) report the effect of the intervention on risk of gestational hypertension onset as RR 0.65 (95% CI 0.53 to 0.81) through a Cochrane review of RCTs.

**treatment_effect_calcium_pre_eclamp**	The effect of daily calcium supplementation on risk of pre-eclampsia onset during the antenatal period	0.45	See **treatment_effect_gest_htn_calcium**. Hofmeyr et al. (44) report the effect of the intervention on pre-eclampsia onset as RR 0.45, (95% CI 0.31 to 0.65).
**treatment_effect_anti_htns_progression**	The effect of oral antihypertensive treatment on the risk of a pregnant or postnatal woman with mild gestational hypertension progressing to severe disease	0.49	Sourced directly from Abalos et al. (56) who report the effect of the intervention on progression to severe disease as (RR) 0.49; 95% (CI 0.40 to 0.60) through a Cochrane review of RCTs
**eclampsia_treatment_effect_severe_pe**	The effect of $MGSO_4$ treatment on risk of a pregnant or postnatal woman progressing from severe pre-eclampsia to eclampsia.	0.41	Sourced directly from Duley et al. (57) which reports the effect of $MGSO_4$ therapy delivered to women with severe pre-eclampsia on risk of progression to eclampsia as derived from a Cochrane review of relevant trials. They report the effect as RR 0.41 (95% CI 0.29 to 0.58).
**eclampsia_treatment_effect_md**	The effect of $MGSO_4$ treatment on risk of maternal death due to eclampsia	0.4	Sourced directly from Pollard et al. (37) in which the authors estimate the effect of $MGSO_4$ therapy on maternal death due to eclampsia via a Delphi survey of relevant experts. Effectiveness is reported as 60%.
**anti_htns_treatment_effect_md**	The effect of intravenous antihypertensive treatment on risk of maternal death due to severe pre-eclampsia or eclampsia	0.5	Sourced directly from Pollard et al. (37) in which the authors estimate the effect of hypertensive therapy on maternal death due to hypertensive disorders via a Delphi survey or relevant experts. Effectiveness is reported as 50%.

**prob_delivery_modes_ec**	The probabilities that a woman experiencing eclampsia in pregnancy will deliver without intervention, via AVD or via CS respectively.	[0, 0, 1]	Assumption
**prob_delivery_modes_spe**	The probabilities that a mother experiencing severe pre-eclampsia in pregnancy will deliver without intervention, via AVD or via CS respectively.	[0.05, 0.05, 0.9]	Assumption

** Where two values (or sets of values) are provided the first set is applied from 2010-2014 and the second set from 2015 onwards for a given simulation run (§1.2.1.1)*
*** If two names are provided for the same parameter this means the name varies by python file. Both are provided to ensure clarity when reviewing any code*

Table S49 – Parameters for the hypertensive disorders of pregnancy model

3.1.11 Obstructed labour

3.1.11.1 Condition overview

Obstructed labour (OL) can be defined as "a situation when the descent of the presenting part (of the foetus) is arrested during labour due to an insurmountable barrier. This occurs in spite of strong uterine contractions and further progress cannot be made without assistance. Obstruction usually occurs at the brim but it may occur in the cavity or at the outlet of the pelvis" (236). The onset of OL can be secondary to several distinct or co-existing causes, most notably cephalopelvic disproportion (CPD), in which there is a mismatch between foetal head size and maternal pelvis, foetal malpresentation or malposition, such as breech or face presentation, and other maternal or foetal physiological causes (237–240).

Predictive factors of CPD, the most reported cause of OL in east African settings for which there is data (238), were identified from the literature and in collaboration with clinicians before inclusion in the model and include foetal macrosomia (242) and maternal stunting (242–244). Due to inconclusive findings relating to predictors of malpresentation/malposition and 'other' causes of OL only predictors of CPD have been included in the model.

OL is associated with considerable maternal and perinatal morbidity and mortality, especially in settings where access to timely treatment may be limited (236,240,241). Most notably OL has a causal relationship with maternal uterine rupture which itself is associated with a very high probability of death. Regarding morbidity, OL is credited as one of the primary causes of obstetric fistula which can lead to significant life-long disability and societal impacts for women of reproductive age and is particularly prevalent in Malawi (25,245).

Estimates of the pooled global incidence of OL appears to be limited. Whilst the GBD study does report the assumed global incidence rate of OL of 121.62 (97.77, 151.60) per 100, 000 people (1), this is a combined estimate including cases of uterine rupture. Interestingly, within east Africa, a systematic review of studies conducted in Ethiopia, containing data

from over twenty-eight thousand births, reported a pooled incidence of OL among mothers who gave birth in Ethiopia of 12.93% (95% CI: 10.44, 15.42) suggesting a considerable number of births are affected per year (238) and a much higher rate than reported by the GBD group, likely due to the authors study definition of OL which included prolonged labour. Similarly, data from Malawi suggests that the incidence of OL is high, albeit lower than estimates provided by Ayenew (238), with the 2015 BEmONC survey reporting a total of 20,232 cases of OL giving an estimated rate of 33.8 cases per 1000 live births (33).

3.1.11.2 Model

Figure S24 describes the model of OL and Table S49 describes model parameters.

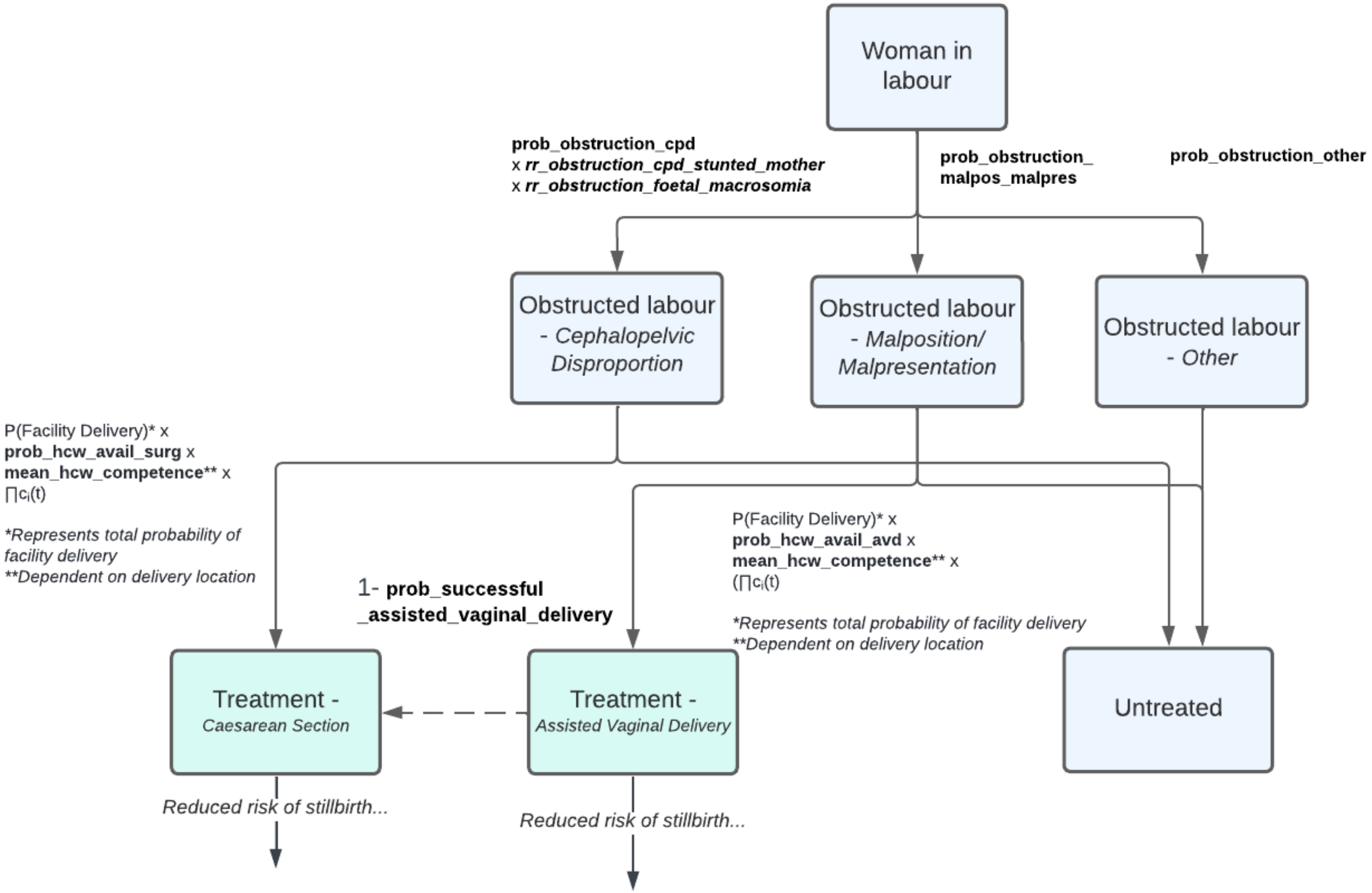

Figure S24 – Model of obstructed labour

Diagrammatic representation of the model of obstructed labour. Light blue represents the model's natural history without treatment whilst teal represents treatment pathways. Parameters representing progression through model states are shown here.

The probability that labour will become obstructed is applied to all individuals at labour onset. It is assumed that multiple potential 'causes' of obstructed labour (CPD, malposition/malpresentation and 'other) can co-exist in the same individual and therefore the probabilities of OL onset are applied independently from each cause.

The probability of developing OL due to CPD is calculated as shown below:

$$\mathrm{Y} = \mathbf{prob\_obstruction\_cpd} * (nb\_low\_birth\_weight\_status * \mathbf{rr\_obstruction\_foetal\_macrosomia}) * (un\_HAZ\_catagory\_stunting * \mathbf{rr\_obstruction\_cpd\_stunted\_mother}) \quad (24)$$

Here foetal birthweight, which is calculated at labour onset, acts as a predictor for risk of CPD alongside maternal Height-for-Age Z score, which denotes if a mother has experienced stunting during childhood and is therefore likely to be small stature. Currently individual height/stature is not modelled however as there was strong evidence of a relationship between stature and OL, maternal stunting was deemed an appropriate proxy. The parameters **prob_obstruction_malpos_malpres** and **prob_obstruction_other** represent risk of OL due to the remaining causes in the model. These parameters lead to a population level incidence of OL of 33.8 cases per 1000 births as sourced from estimates in Malawi (33,34) and discussed further in Table S49.

I do not assign OL as a primary cause of maternal death within the model and instead, where indicated either by literature or clinical review, include OL as a predictor in multiplicative models calculating the probability of other causes of maternal death. Most importantly the relationship between OL and uterine rupture is included which has been well described in the literature and is discussed further §3.1.11. OL is also a predictor of intrapartum stillbirth as described in §3.1.14.

3.1.11.2.1 Treatment

Assisted vaginal delivery for OL

Treatment of OL may be provided to mothers who present during labour to a healthcare facility, including those who initially decided to deliver at home but sought care following the onset of the complication. Receipt of AVD is dependent on the underlying cause of OL being either malposition/malpresentation or 'other' as it is assumed that for true cases of CPD delivery can only occur via CS (32). If AVD will be performed, depending on the quality parameters shown in Figure S24, the success of the intervention is dependent on parameter **prob_successful_assisted_vaginal_delivery,** which represents the probability of AVD leading to a successful delivery given a certain proportion of attempts at delivery can fail (246). If AVD occurs the treatment effect, parameter **treatment_effect_avd_still_birth**, is applied which reduces risk of intrapartum stillbirth, and if delivery cannot occur via AVD the individual is referred for CS, described below.

Caesarean Section for OL

All women with CPD, and those for which AVD was unsuccessful, are referred to deliver via CS. Similarly to all other interventions in the model, delivery of CS is conditional on quality parameters and consumable availability however, unlike AVD, if performed is assumed to always lead to a delivery of the foetus. The parameter **treatment_effect_cs_still_birth** is the effect of CS on risk of stillbirth. Both **treatment_effect_avd_still_birth** and **treatment_effect_cs_still_birth** are described in Table S52.

3.1.11.3 Data sources and parameters

Parameter Name	Description	Value*	Source and/or relevant calculation
prob_obstruction_cpd	The probability that a woman in labour who is not stunted and who is not carrying a macrosomic foetus will develop OL due to CPD	0.012 / 0.022	The assumed rate of OL in Malawi was sourced from the 2010 and 2015 Malawi EmONC assessment surveys (33,34) by dividing the total cases observed in the survey by the estimated births for the survey year giving a rate of 18.3 and 33.8 per 1000 births respectively.<br><br>Next the proportion of OL cases by cause was taken from Ethiopian systematic review conducted by Ayenew (238). The overall rate was multiplied by proportion of cases due to CPD (65%) to give rate secondary to CPD.
**rr_obstruction_cpd_stunted_mother**	The effect of a pregnant woman being stunted compared to not on her risk of OL due to CPD	2.4	Sourced directly from Toh-Adam et al. (245) who report the effect of short stature (height <145cm) on risk of CPD obstruction in a cohort study of over 9,000 deliveries in Thailand.
**rr_obstruction_foetal_macrosomia**	The effect of a pregnant woman's foetus being macrosomic compared to the foetus weighing less than 4kg on her risk of OL due to CPD	3.3	Sourced directly from Tsvieli et al. (241) who report the effect of macrosomia, through a retrospective population-based study including nearly a quarter of a million pregnancies in Israel.
prob_obstruction_malpos_malpres	The probability that a pregnant woman in labour will develop OL due to malposition and/or malpresentation	0.005/ 0.009	*See* **prob_obstruction_cpd.** Rate of obstruction secondary to malpresentation/position assumed to be 27.24% of total OL rate in keeping with estimate from Ayenew (238).
prob_obstruction_other	The probability that a pregnant woman in labour will develop OL due to 'other' causes.	0.0015 / 0.0027	*See* **prob_obstruction_cpd**. Rate of obstruction secondary to 'other causes' assumed to be 8.11% of total OL rate in keeping with estimate from Ayenew (238).

**prob_successful_assisted_vaginal_delivery**	The probability that AVD will be successful in delivering the foetus without the need for further intervention.	0.7	Whilst evidence from several settings suggest that failure rate of vacuum delivery is approximately 5% (246,247) studies indicate that foetal position and operative delivery method can affect success rate considerably (248). In addition, due to lacking estimates from Malawi, we have opted to use a more conservative estimate of success of 70% given that AVD is performed much less commonly than in HICs like the UK (33,65,249)

** Where two values (or sets of values) are provided the first set is applied from 2010-2014 and the second set from 2015 onwards for a given simulation run (§1.2.1.1)*

Table S50- Parameters for the obstructed labour model

3.1.12 Uterine rupture

3.1.12.1 Condition overview

Uterine rupture (UR) is defined as "tearing of the uterine wall during pregnancy or delivery" (250). Rupture of the uterine wall is commonly associated with previous delivery via CS in which tearing occurs along the scar tissue from the previous uterine incision (251). However, although less common, UR can occur in women who have never delivered before or have only delivered vaginally especially in contexts where access to appropriate treatment for prolonged/obstructed may be delayed (252). In addition to caesarean delivery, OL is also a significant driver of UR in which prolonged contractions during labour contribute to the likelihood of tearing within the uterine wall (238).

Considering this, and through the conduct of a literature review to determine predictive factors for UR to include in the model, the following variables were selected when calculating risk of UR: multiparity (having previously delivered more than once), grand multiparity (having previously delivered five or more times), having previously delivered via CS and current labour being obstructed (252,253).

Where UR does occur, the risk of severe maternal outcomes and perinatal death is high, especially in the context of delayed care, with large sample study data reporting an adjusted odds ratio for severe maternal outcomes of 40.22 (95% CI, 24.01–67.36) and perinatal death 33.34 (95% CI, 21.59– 51.51) when compared to women without UR (254). Poor maternal and perinatal outcomes are largely associated with maternal haemodynamic compromise secondary to hypovolemic shock (255,256).

Largely, UR is considered an extremely rare clinical event with data taken from population-based studies across settings estimating a pooled prevalence of around 0.053% of births being affected by UR (250). However, the incidence in women who have delivered previously via CS is reportedly much higher, and UR may occur in as many as 0.5% of births within this population (254). Notably, when evaluating incidence by country, the observed incidence of UR is greater in women delivering in LMIC when compared to HIC (250,254).

Within Malawi, an estimated 507 cases of UR were documented in the 2015 EmONC survey leading to an overall rate of 1.06 per 1000 births (or 0.106% of all births) (33).

3.1.12.2 Model

Figure S25 describes the model of UR that is employed within the module.

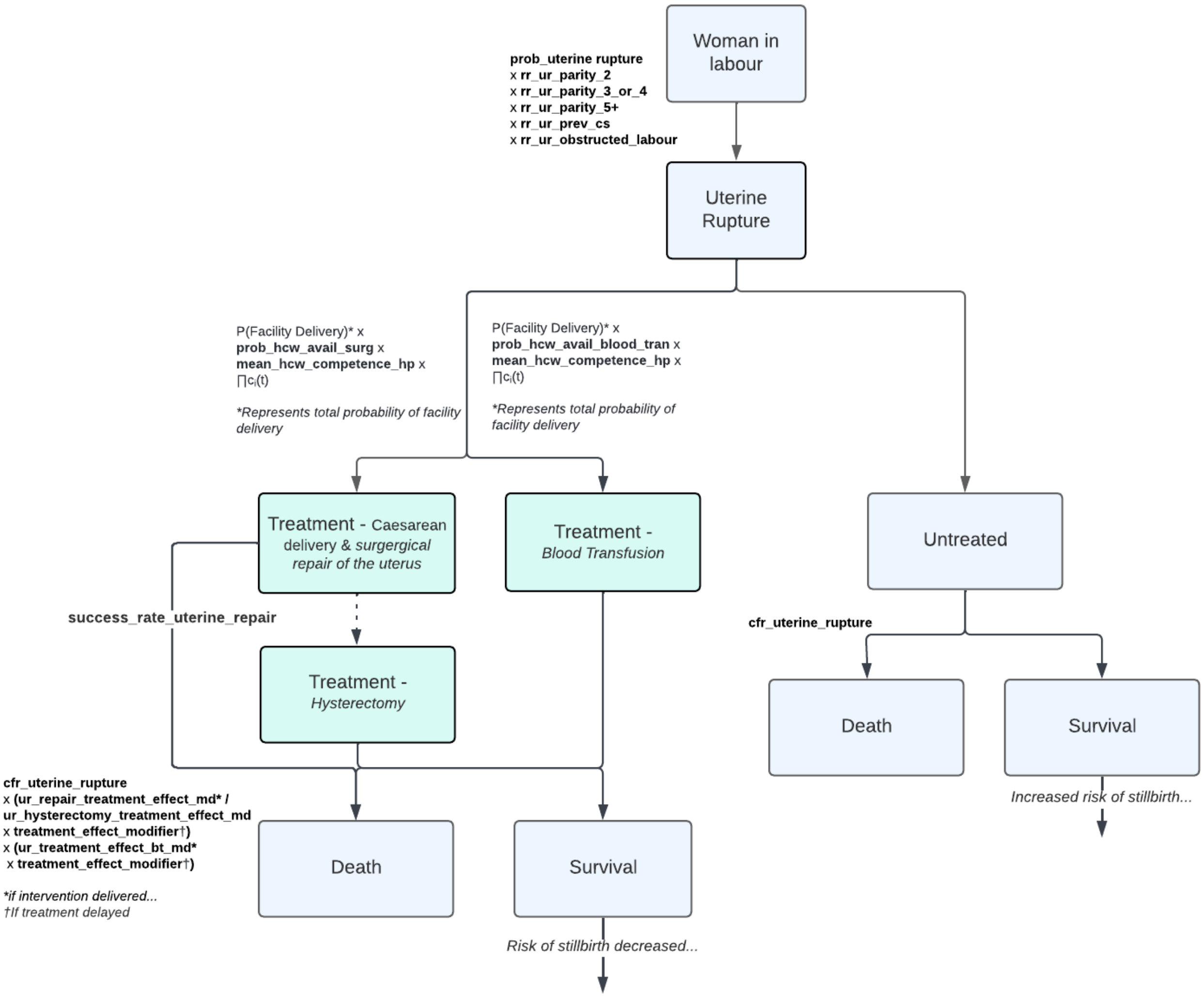

Figure S25 – Model of uterine rupture
Diagrammatic representation of the model of uterine rupture. Light blue represents the model's natural history without treatment whilst teal represents treatment pathways. Parameters representing progression through model states are shown here.

Individual risk of UR during labour is calculated via the following multiplicative model in equation 25:

$$Y = \mathbf{prob\_uterine\_rupture} * (la\_parity\_2 * \mathbf{rr\_ur\_parity\_2}) * (la\_parity\_3\_4 * \mathbf{rr\_ur\_parity\_3\_or\_4}) * (la\_parity\_5\_plus * \mathbf{rr\_ur\_parity\_5} +) * (la\_prev\_cs * \mathbf{rr\_ur\_prev\_cs}) * (la\_obstructed\_labour * \mathbf{rr\_ur\_obstructed\_labour}) \quad (25)$$

To ensure that the variable which denotes a woman is currently in OL has been updated prior to the calculation of risk of UR, the application of the risks of these complications occurs sequentially. As such interventions delivered to treat obstructed labour, such as AVD, do not currently reduce risk of uterine rupture if successfully delivered.

Calculation of individual risk through equation 25 within the model generates a rate of 1.15 uterine rupture cases per 1000 births in keeping with estimates from Malawi (33). Similarly to OL, UR is considered an obstetric emergency and mothers who are delivering at home may seek care for treatment after the complication onsets.

3.1.12.2.1 Treatment

Clinical guidelines recommend immediate surgical intervention for the management of UR which entails delivery of the foetus and placenta via CS, followed by either repair of the uterine wall or, in the case where damage to the uterus is irreparable, total hysterectomy via laparotomy (32,257). To replicate this process in the model, women with uterine rupture are scheduled to receive CEmONC care, where first it is determined if the required resources or staff are available to undertake surgical care. If so, CS delivery is performed, and the appropriate variables are updated. Following this, the parameter **success_rate_uterine_repair** is used to determine if surgical repair of the uterus is possible, otherwise a hysterectomy is performed meaning this individual is unable to become pregnant again for the remainder of the simulation. The effect of CS delivery on risk of intrapartum stillbirth is applied as described in §3.1.14 whilst the effect of uterine repair or hysterectomy is applied to risk of maternal death secondary to rupture through parameters

ur_repair_treatment_effect_md or **ur_hysterectomy_treatment_effect_md** (Table S50). In addition, individuals may receive a blood transfusion alongside surgical interventions which further reduces the risk of death through parameter **ur_treatment_effect_bt_md.**

One primary assumption in this model is that women are at increased risk of UR after experiencing obstructed labour (Table S50 below), regardless of whether successful treatment for OL (AVD/CS) occurs. Whilst one could assume that there is no longer a relationship between OL and UR if treatment is delivered successfully, this is an abstraction of reality given women may be in obstructed labour for a significant amount of time prior to treatment which would likely increase their risk of UR. Additionally, blocking UR in women who were successfully treated was determined not to reflect reality as women may experience uterine rupture due to reasons other than obstructed labour (258,259).

3.1.12.3 Data sources and parameters

Parameter Name	Description	Value*	Source and/or relevant calculation
**prob_uterine_rupture**	This parameter is scaled at intialisation of the simulation to account for the proportion of women at baseline who have previously delivered by caesarean section and whose parity is greater than 1.<br><br>Once scaled, as the simulation runs, this parameter represents the probability that a pregnant woman who is primiparous, has not delivered previously by CS and is not in obstructed labour will experience UR.<br><br>The parameters below starting with “rr_ur” refer to the effect on the probability of UR	0.0011 / 0.0005	The assumed rate of UR in Malawi was sourced from the 2010 and 2015 Malawi EmONC assessment surveys (33,34) by dividing the total UR cases observed in the survey by the estimated births for the survey year giving a rate of 1.25 and 0.84 per 1000 births respectively.
**rr_ur_parity_2**	The effect of a pregnant woman having previously delivered twice compared to once or never	2.74	Sourced directly from Delafield et al. (252) who report the effect of several predictors of UR via a mixed-effects logistic regression model including data on over 84,000 women in Mali and Senegal.
**rr_ur_parity_3_or_4**	The effect of a pregnant woman having previously delivered three or four times compared to once	4.89	See **rr_ur_parity_2.**
**rr_ur_parity_5+**	The effect of a pregnant woman having previously delivered five or more times compared to once or never	7.57	See **rr_ur_parity_2.**

**rr_ur_prev_cs**	The effect of a pregnant woman having ever previously delivered via CS	2.02	See **rr_ur_parity_2.**
**rr_ur_obstructed_labour**	The effect of a pregnant woman in labour being in OL	23.6	See **rr_ur_parity_2.**
**cfr_uterine_rupture**	The probability that a pregnant woman will die following UR during labour without treatment	0.65 / 0.50	See **prob_ectopic_pregnancy_death** in Table S39**.**
**success_rate_uterine_repair**	The probability that surgical repair of a ruptured uterus will be successful, avoiding hysterectomy	0.83	Due to lacking estimates from Malawi this parameter was sourced directly from Sinha et al. (260) who report outcomes from a seven-year retrospective analysis of treatment of UR cases in a tertiary facility in New Delhi.
**ur_repair_treatment_effect_md**	The effect of surgical repair of ruptured uterus on risk of maternal death due to UR	0.25	Pollard et al. (37) estimate the effect of interventions on maternal death due to obstructed labour but not UR. Due to the relationship between these factors, it is assumed these effects are interchangeable for the purpose of the model. The effectiveness of 'CEmONC' services on obstructed labour deaths is reported as 90%. These services are reasonably constituted of surgery and blood transfusion. As such blood transfusion effect is assumed to be 0.4 and the surgical effect is 0.25. (0.25 x 0.4 = 0.1)
**ur_hysterectomy_treatment_effect_md**	The effect of hysterectomy on risk of maternal death due to UR	0.25	See **ur_repair_treatment_effect_md**. For the model, it is assumed the effects of uterine preserving surgery and hysterectomy on maternal mortality are the same.

**ur_treatment_effect_bt_md**	The effect of blood transfusion on risk of maternal death due to UR	0.4	*See* **ur_repair_treatment_effect_md.**

** Where two values (or sets of values) are provided the first set is applied from 2010-2014 and the second set from 2015 onwards for a given simulation run (§1.2.1.1)*

Table S51 Parameters of the uterine rupture model

3.1.13 Postpartum haemorrhage

3.1.13.1 Condition overview

The term postpartum haemorrhage (PPH) broadly refers to clinically significant bleeding following birth, which can be sub-categorised as primary PPH, if a woman experiences blood loss of 500ml or more within twenty-four hours of birth, or secondary PPH, if any 'abnormal or excessive' bleeding from the birth canal occurs between twenty-four hours and twelve weeks after delivery (261).

The aetiology of both primary and secondary PPH Is extensively discussed In the literature and underlying causes of bleeding can often be attributed to one of the 'Four T's', a mnemonic popular in clinical obstetrics, namely Tone, Tissue, Trauma and Thrombin (262). 'Tone' refers to PPH caused by uterine atony, in which the uterus fails to contract sufficiently following birth to achieve haemostasis. This is widely reported as the leading cause of PPH and is particularly associated with primary bleeding (263–266). 'Tissue' relates to bleeding associated with retention of tissues within the uterus (such as the placenta) or conditions of invasive placenta. When co-existing with maternal infection, it is often attributed as the most common cause of secondary bleeding (262,267). 'Trauma' relates to bleeding that can occur for many reasons but is often attributed to genital tract laceration or tears occurring during labour due to natural or nosocomial causes. 'Thrombin' refers to bleeding secondary to maternal coagulopathy often caused by clotting disorders (262).

As described below, we have opted to model primary and secondary PPH as distinct conditions and to include risk of primary PPH due to uterine atony (tone), retained placenta/products (tissue) and 'other causes' (trauma/thrombin). Regarding primary PPH, the review of the literature for predictive factors identified maternal hypertension, twin pregnancy, foetal macrosomia, and placental abruption as predictors of PPH secondary to uterine atony (268). Significant important risk factors for primary PPH associated with retained placenta/products were not identified for inclusion.

PPH is one of the most common causes of maternal death globally (269). Haemorrhage is particularly dangerous in settings with a significant burden of maternal anaemia, because

the volume of maternal blood loss leading to clinically significant outcomes is much less in anaemic women than in those with normal haemoglobin levels (270). This relationship is captured explicitly in the model.

Globally as many as 6.09% (95% CI 6.06, 6.11) of women who deliver will experience PPH; however, this estimate varies considerably according to how bleeding is measured (objectively or not) and how the severity of haemorrhage is defined (271). Estimates of PPH prevalence vary significantly between regions: from 7.2% (95% CI 6.3, 8.1) in Oceania to 25.7% (95% CI 13.9, 39.7) in Africa; however, there is evidence of considerable heterogeneity between studies associated with study country, methods of blood loss measurement and mode of delivery (272).

In Malawi, data suggests that the incidence of PPH is much lower than estimated by the above studies. Lokken et al. (273), who conducted a systematic review and meta-analysis of studies reporting incidence of PPH in Malawi reported that 2% (95% CI 1.7%, 2.4%) of births are complicated by postpartum haemorrhage, however only two studies were identified for review. Additionally, data taken from the EmONC surveys conducted in 2010 and 2015 captured 4658 and 6958 cases of PPH respectively leading to an estimated 1.61% and 1.46% prevalence of PPH after birth, significantly lower that global or regional estimates (33,34).

3.1.13.2 Model

Figures S26 and S27 describe the models of primary and secondary PPH respectively which are employed within the module.

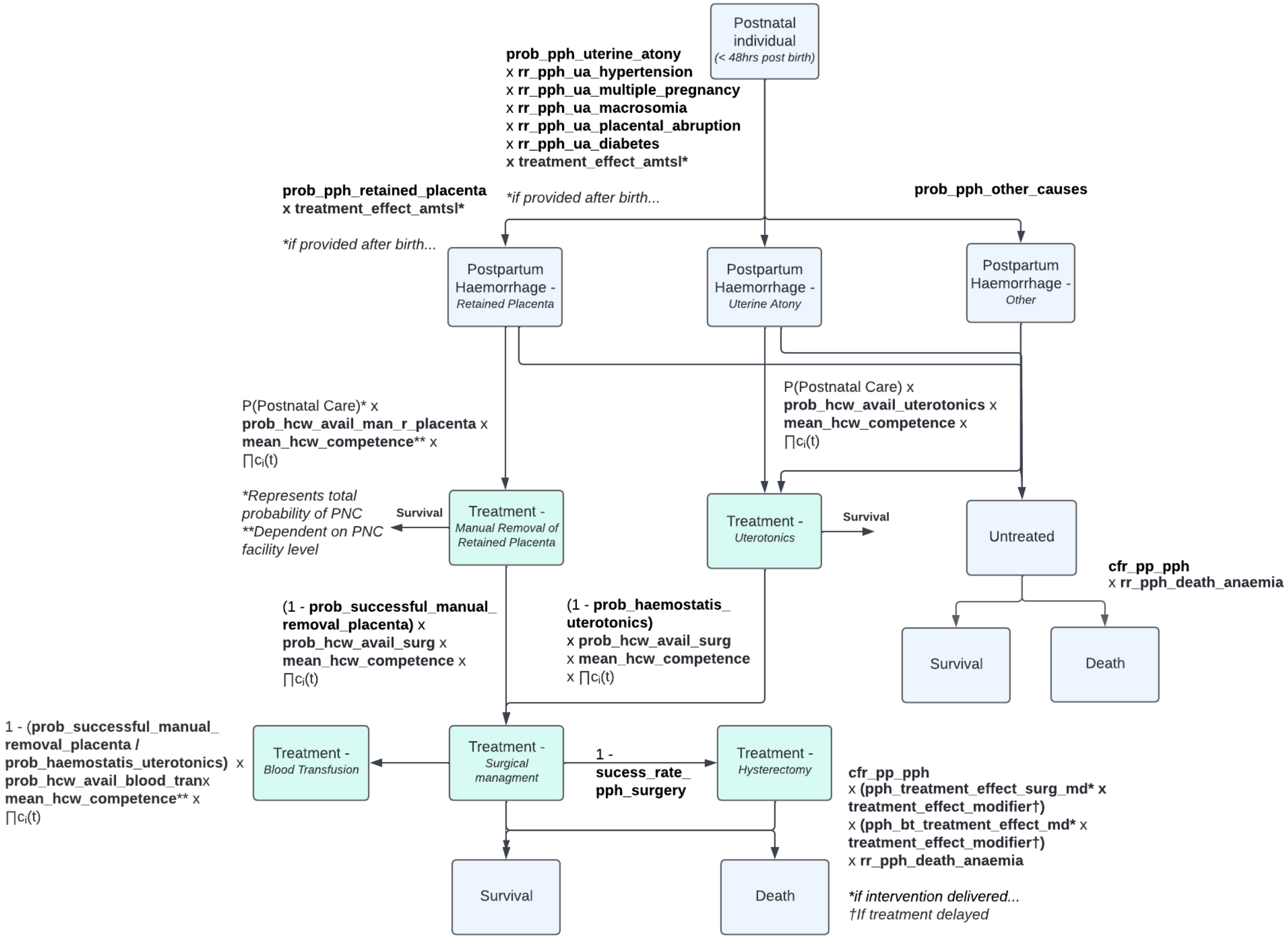

Figure S26 – Model of primary postpartum haemorrhage

Diagrammatic representation of the model of primary postpartum haemorrhage. Light blue represents the model's natural history without treatment whilst teal represents treatment pathways. Parameters representing progression through model states are shown here.

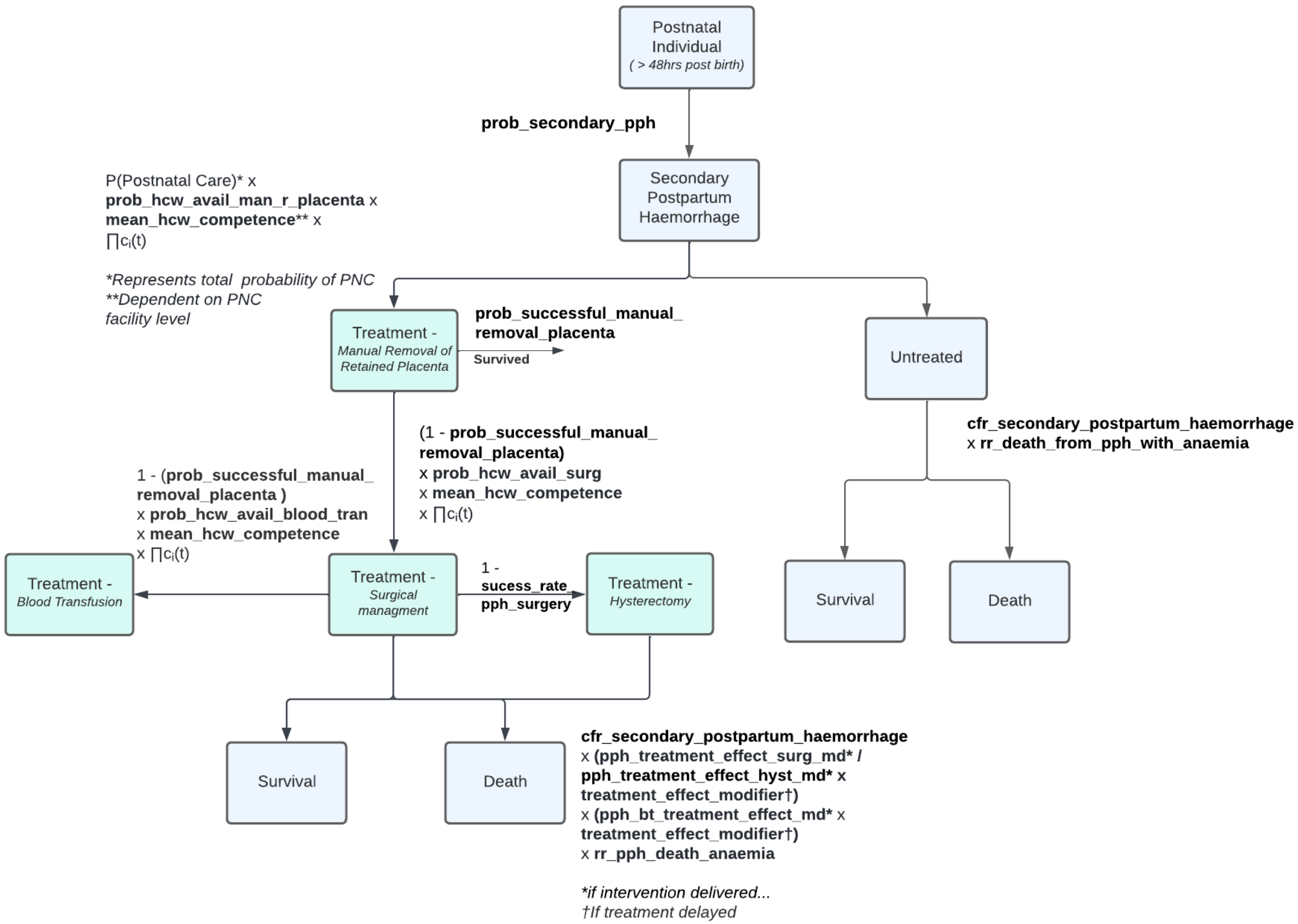

Figure S27 – Model of secondary postpartum haemorrhage
Diagrammatic representation of the model of secondary postpartum haemorrhage. Light blue represents the model's natural history without treatment whilst teal represents treatment pathways. Parameters representing progression through model states are shown here.

Primary PPH

As described above, PPH may be due to several distinct or coexisting causes. To replicate this within the simulation, and to allow for treatment delivery based on the aetiology of the bleed, which is common practice within Malawi (31,32), overall incidence of primary PPH is driven by probability of bleeding secondary to uterine atony, retained placenta or 'other' causes (i.e. coagulopathies, iatrogenic/non-iatrogenic lacerations). Similarly, to the model of OL, these probabilities are applied independently under the assumption that multiple causes of PPH may coexist in the same woman. Individual risk of primary PPH due to uterine atony is applied to all women immediately following delivery and is calculated by equation 26:

$$\begin{aligned} \mathrm{Y} = \mathbf{prob\_pph\_uterine\_atony} * (&ps\_htn\_disorders * \ \mathbf{rr\_pph\_ua\_hypertension}) \\ &* (ps\_multiple\_pregnancy * \mathbf{rr\_pph\_ua\_multiple\_pregnancy}) \\ &* (mni\_birth\_weight * \mathbf{rr\_pph\_ua\_macrosomia}) \\ &* (ps\_placental\_abruption * \mathbf{rr\_pph\_ua\_placental\_abruption}) \\ &* (mni\_amtsl\_given * \mathbf{treatment\_effect\_amtsl}) \end{aligned} \quad (26)$$

In addition to the predictors of PPH due to atonic uterus introduced above, prophylactic treatment in the form of active management of the third stage of labour (AMTSL) is included as an intervention shown to reduce the risk of bleeding for primary PPH due to atonic uterus and retained placenta (63), as described further below.

The risk of primary PPH due to retained placenta is calculated as:

$$\mathrm{Y} = \mathbf{prob\_pph\_retained\_placenta} * \ (mni\_amtsl\_given * \mathbf{treatment\_effect\_amtsl}) \quad (27)$$

Finally, parameter **prob_pph_other_causes**, represents the risk of primary PPH due to 'other' causes. Severity of bleeding is determined using a weighted random draw using parameter **severity_maternal_haemorrhage** (Table S51) which is used to determine the appropriate DALY weight for each woman. It is assumed, similarly to APH, that the severity of bleeding does not directly impact risk of death and instead only affects morbidity via

disability weighting. This decision was made as the data source used to calibrate case fatality parameters did not provide varying estimates by severity and therefore the numerator used by the authors included all PPH cases.

As evident from Figure S26 above, parameter **cfr_pp_pph** which represents the risk of death due to PPH is only applied to women who do not receive treatment or women for whom first line treatment was unsuccessful – under the assumption that successful first line treatment completely blocks the causal pathway to death. Additionally, women who are currently anaemic are at greater risk of death, with risk of death multiplied by **rr_death_from_haem_with_anaemia** for these women – this remains true for all other causes of PPH.

Secondary PPH

A per-week risk of secondary PPH is applied to all women during weeks 1-6 of the postnatal period (**prob_secondary_pph)**. Individuals who experience secondary PPH may seek postnatal care with treatment reducing associated risk of death. Likelihood of death from secondary PPH is assumed to be the same as risk of death from primary PPH due to lacking data to the contrary.

3.1.13.2.1 Treatment

Primary PPH –AMTSL

Prophylactic treatment for the prevention of primary PPH is initiated for women who deliver within health facilities. The third stage of labour, in which the uterus contracts and the placenta is delivered following birth, can be managed either actively or physiologically. A physiological third stage is one without intervention, whereas AMTSL involves intervention by a HCW who will deliver a prophylactic uterotonic drug during or immediately following birth, clamp the umbilical cord (either immediately or after a period of delay) and apply controlled cord traction to deliver the placenta (257). In both international (274,275) and Malawian guidelines (32) AMTSL is recommended for all deliveries to reduce the incidence of severe postpartum bleeding (63). Within the model it is assumed that AMTSL reduces the risk of primary postpartum haemorrhage secondary to either uterine atony or retained

placenta via the parameter **treatment_effect_amtsl** and does not impact other causes of primary PPH or any cases of secondary PPH as shown in Figures S26 and S27.

Primary PPH – Uterotonics

Treatment for primary PPH is initiated within PNC. Uterotonics drugs such as oxytocin are recommended as first line treatment within international guidelines (274,275) and clinical guidelines in Malawi (31,32). If treatment is delivered, parameter **prob_haemostatis_uterotonics** determines if treatment is effective in stopping haemorrhage, in which case the property denoting PPH is reset for this individual and they are no longer at risk of death. If treatment is unsuccessful the individual is assumed to require surgery and are referred accordingly.

Primary PPH – Manual removal of retained placenta

Manual removal of retained placenta (MRRP) is a clinical intervention, that can be delivered at the bedside, in which a HCW detaches the placenta from the uterine wall manually and removes the placenta and any remaining tissue from the uterus to stop/prevent bleeding (257). MRRP is indicated for any woman in which haemorrhage is occurring and the placenta has not been delivered within 30 minutes of birth (257) and is within the PPH treatment cascade in Malawian guidelines (31,32).

In individuals with PPH secondary to retained placenta who receive MRRP, similarly to uterotonic treatment, the parameter **prob_successful_manual_removal_placenta** is used to determine if the intervention is successful and, if so, resets the relevant properties averting risk of death. In the case of failed MRRP an individual is referred for surgical treatment (31,32) in which a laparotomy and surgical removal of retained placenta can be conducted.

Primary PPH – Surgical management and blood transfusion

In the context of PPH, which is refractory to first line treatment, surgical intervention is indicated to preserve maternal life (257). In Malawi, recommended treatment for uncontrolled PPH secondary to atonic uterus is a laparotomy followed by B-lynch suture, a compression suture that manually contracts an atonic uterus (276), bilateral uterine artery ligation, or hysterectomy (32). In the context of retained placenta, guidelines are less

explicit but commonly, if retained products cannot be removed safely post laparotomy then subtotal or total hysterectomy is performed (32).

Modelled individuals can be referred for surgical treatment of PPH if first line treatments are unsuccessful as described above. Successful surgical management of atonic PPH, in which the uterus is preserved, is determined using a random draw against parameter **success_rate_pph_surgery.** If the uterus cannot be preserved, it is assumed a hysterectomy is performed and the individual can no longer become pregnant within the model. Women undergoing surgery for refractory PPH are assumed to be at risk of death, shown in parameter **cfr_pp_pph**, which is reduced by the treatment effect of surgery parameter **pph_treatment_effect_surg_md**. Currently there is no assumed difference in effect on mortality between uterine conserving surgery and hysterectomy and therefore the treatment effects of both interventions are identical as shown in Table S51.

In addition to surgery, transfusion of red blood cells during severe haemorrhage is standard clinical practice in all settings regardless of the aetiology of bleeding. Malawian guidelines are explicit in the need to cross match for transfusion and repeat key blood tests such as a full blood count after PPH onset but are not explicit at which point, or under what indications, women should be transfused following a PPH (32). As such, in the model it is assumed that only women for whom first line treatments (uterotonics/MRRP) are unsuccessful will require blood transfusion. The parameter **pph_bt_treatment_effect_md** is the effect of blood transfusion on risk of maternal death.

Secondary PPH

Whilst the underlying cause of secondary PPH are not modelled, data suggests that retained placental tissue is a leading cause (277,278). As such, the same treatment pathway as for women experiencing retained placenta are used. Whilst this is an abstraction from reality it was deemed appropriate for the purposes of the model at this time.

3.1.13.3 Data sources and parameters

Parameter Name	Description	Value*	Source and/or relevant calculation
**prob_pph_uterine_atony**	The probability of a postnatal woman experiencing primary PPH secondary to uterine atony<br><br>The parameters below starting with "rr_pph_ua" refer to the effect on the probability of PPH due to uterine atony	0.006 / 0.011	The assumed rate of PPH in Malawi was sourced from the 2010 and 2015 Malawi EmONC assessment surveys (33,34) by dividing the total PPH cases observed in the survey by the estimated births for the survey year giving a rate of 7.95 and 12.8 per 1000 births respectively.<br><br>In keeping with estimates on the incidence of secondary PPH from other settings (277) it is assumed that 85% of the total PPH rate is due to primary bleeding and the remaining is due to secondary bleeding. This leads to a rate of primary PPH 6.76 per 1000 births in 2010 and 10.88 in 2015.<br><br>To ensure the percentage of primary PPH cases due to uterine atony is correct, estimates of proportion of total PPH cases by cause were estimated from data reported from the US (279). Therefore, it is assumed that 80% of primary PPH cases are due to uterine atony leading to a rate of 5.4 per 1000 births in 2010 and 8.7 in 2015.<br><br>We have derived these parameters through calibration to these rates.
**rr_pph_ua_hypertension**	The effect of a postnatal woman being hypertensive compared to normotensive	1.84	Sourced directly from Ende et al. (268) who report the effect of several risk factors on risk of PPH due to uterine atony via a systematic review and meta-analysis of published studies.
**rr_pph_ua_multiple_pregnancy**	The effect of a postnatal woman having been pregnant with a twin pregnancy compared to a single foetus	2.16	See **rr_pph_ua_hypertension**
**rr_pph_ua_macrosomia**	The effect of a postnatal woman having delivered a	1.46	See **rr_pph_ua_hypertension**

	macrosomic foetus compared to a non-macrosomic foetus		
**rr_pph_ua_placental_abruption**	The effect of a postnatal woman having experienced placental abruption during pregnancy	2.74	See **rr_pph_ua_hypertension**
**prob_pph_retained_placenta**	The probability of a postnatal woman experiencing primary PPH secondary to a retained placenta	0.001 / 0.0016	See **prob_pph_uterine_atony**. Bateman et al. (279) report 10% of observed PPH was due to retained placenta. Therefore, the rate of PPH secondary to retained placenta in the model is equal to 0.68 per 1000 births in 2010 and 1.1 per 1000 births in 2015.
**prob_pph_other_causes**	The probability of a postnatal woman experiencing primary PPH secondary to other causes	0.0007/ 0.001	See **prob_pph_uterine_atony**. Bateman et al. (279) report 10% of observed PPH was due to other causes. Therefore, the rate of PPH secondary to other causes in the model is equal to 0.68 per 1000 births in 2010 and 1.1 per 1000 births in 2015.
**cfr_pp_pph**	The probability that a postnatal woman will die following PPH without treatment	0.22 / 0.08	See **prob_ectopic_pregnancy_death** in Table S39**.**
**rr_death_from_haem_with_anaemia**	The effect of maternal anaemia on a postnatal woman risk of dying from PPH	1.5	See **rr_death_from_haem_with_anaemia** Table S41.
**prob_secondary_pph**	The probability that a postnatal woman who has recently delivered will experience a PPH occurring more than twenty-	0.0002 / 0.0004	See **prob_pph_uterine_atony.** In keeping with the assumption that 20% of total PPH burden is due to secondary bleeding the assumed rate of secondary PPH is 1.19 per 1000 in 2010 and 1.92 per 1000 in 2015.

	four hours after birth per week of the postnatal period		
cfr_secondary_postpartum_haemorrhage	The probability that a postnatal woman will die following a secondary PPH without treatment	0.22 / 0.08	See **prob_ectopic_pregnancy_death** in Table S39**.**
**treatment_effect_amtsl**	The effect of AMTSL on maternal risk of PPH secondary to atonic uterus or retained placenta	0.34	Sourced directly from Begley et al. (63) who reports the effect of AMTSL on risk of haemorrhage as RR 0.34 (95% CI 0.14 to 0.87) through a Cochrane review of RCTs.
**prob_haemostatis_uterotonics**	The probability that the administration of uterotonic drugs to a postnatal woman experiencing PPH secondary to uterine atony will stop bleeding	0.57	Gallos et al. (71) estimate the relative effect of oxytocin administration on the whether a woman experiencing PPH who has received treatment with the drug will require additional uterotonics as RR 0.43 (0.32, 0.58) via a Cochrane review. This equates to a 57% reduction in need for additional treatment post oxytocin. For the model, it is assumed therefore that there is a 0.57 probability that oxytocin will prevent the need for additional treatment and stop bleeding.
**prob_successful_manual_removal_placenta**	The probability that MRRP will stop additional bleeding in a postnatal woman experiencing PPH secondary to retained placenta	0.75	Pollard et al. (37) report a 75% effectiveness of 'BEmONC' on reducing mortality caused by PPH via a Delphi survey of relevant experts which has been used for this parameter. Whilst the authors do report an estimate for the effect or MRRP on death due to PPH (30%) this is considerably lower than the estimate for BEmONC.

**pph_bt_treatment_effect_md**	The effect of blood transfusion on risk of death secondary to PPH	0.4	Pollard et al. (37) estimate of 'CEmONC' services on PPH deaths is reported as 90%. These services are reasonably constituted of surgery and blood transfusion. As such blood transfusion effect is assumed to be 0.4 and the surgical effect is 0.25. (0.25 x 0.4 = 0.1)
**success_rate_pph_surgery**	The probability that uterine preserving surgery for the management of PPH will be successful and the postnatal woman will not require a hysterectomy	0.79	The success rate of uterine preserving surgery in the management of intractable PPH taken directly from a five-year review of surgical management in a Nigerian centre by Cengiz et al. (281).
**pph_treatment_effect_surg_md**	The effect of uterine preserving surgical management of PPH on risk of death secondary to PPH	0.25	See **pph_bt_treatment_effect_md**
**pph_treatment_effect_hyst_md**	The effect of hysterectomy as management of PPH on risk of death secondary to PPH	0.25	See **pph_treatment_effect_surg_md**. Presently, it is assumed that uterine preserving surgery and hysterectomy are equally effective in preventing death.

** Where two values (or sets of values) are provided the first set is applied from 2010-2014 and the second set from 2015 onwards for a given simulation run (§1.2.1.1)*

Table S52 – Parameters for the postpartum haemorrhage model

3.1.14 Stillbirth

3.1.14.1 Condition overview

3.1.14.1.1 Antenatal stillbirth

Stillbirth is defined as a baby born with no signs of life at or after 28 weeks' gestation (281). Stillbirths can occur prior to the onset of labour, referred to as antenatal stillbirths, or during labour and delivery, referred to as intrapartum stillbirths. There is extensive literature evaluating the aetiology of antenatal stillbirth across settings. Through evaluation of the literature which has attempted to classify underlying causes of antenatal stillbirth in contexts like Malawi, the antecedent causes of antenatal stillbirth can be categorised broadly as haemorrhage secondary to placental issues, maternal infection, other maternal disorders and other or unknown causes (such as non-survivable structural foetal anomalies, pathological placental conditions, unknown cause) (282–286).

Using this framework, and through review of the literature and discussion with clinical experts, the following factors have been included in the model that impact risk of stillbirth; APH (287), antenatal infection due to chorioamnionitis (288), syphilis (151), malaria (289), other direct maternal conditions associated with pregnancy (pre-eclampsia and gestational hypertension (290) and GDM (291), indirect maternal conditions which onset during pregnancy or are already present to (chronic hypertension (290), diabetes mellitus (292)) and post term pregnancy (174). The rate of stillbirth in Malawi is described in Table S52.

3.1.14.1.2 Intrapartum stillbirth

Intrapartum stillbirth refers to a foetal death occurring after the onset of labour in which "documentation of a live foetus prior to or at the onset of labour exists" (293) and can clinically be defined as "delivery of a foetus occurring after 22 weeks of gestation or with a birthweight more than 500 g, who had foetal heart sounds (FHS) at admission but no FHS present 15 minutes before delivery and never breathed spontaneously after birth or after 10 minutes of resuscitation"(294). Importantly, within the model, as recommended by the WHO (281) a 28 -week GA cut off is used.

As with stillbirths occurring in the antenatal period, determining underlying cause of foetal death can be particularly challenging due to the relationship between individual pathophysiological and wider health system factors which often coexist in many settings contributing to death (295). However, evidence does suggest that intrapartum stillbirths are often due to sustained foetal hypoxia, surpassing usual foetal tolerance for transient hypoxia associated with normal labour, caused by intrapartum complications (296,297). This is supported by several studies conducted in LMICs demonstrating significant association between intrapartum foetal death and commonly occurring maternal complications such as obstructed labour, uterine rupture, and intrapartum haemorrhage (254,287). As such, predictive factors identified for inclusion in the model from relevant literature include maternal death, uterine rupture, obstructed labour, APH, maternal hypertension, maternal sepsis, and twin gestation (254,287).

3.1.14.2 Models

3.1.14.2.1 Antenatal stillbirth

A per-month risk of antenatal stillbirth is applied to all women following 28 weeks' gestation in keeping with the WHO definition discussed above. The model used to calculate individual risk of stillbirth, at time (t), is described in equation 28:

$$
\begin{aligned}
Y_{(t)} = \mathbf{prob\_still\_birth\_per\_month} \\
&* (ps\_gestational\_age\_in\_weeks\_41 \; * \; \mathbf{rr\_still\_birth\_ga\_41}) \\
&* (ps\_gestational\_age\_in\_weeks\_42 * \; \mathbf{rr\_still\_birth\_ga\_42}) \\
&* (ps\_gestational\_age\_in\_weeks\_43\_plus * \; \mathbf{rr\_still\_birth\_ga\_} > \mathbf{42}) \\
&* \; (ps\_gest\_diab * \mathbf{rr\_still\_birth\_gest\_diab}) \\
&* \; (nc\_diabetes \; * \mathbf{rr\_still\_birth\_diab\_mellitus}) \\
&* \; (ma\_is\_infected * \mathbf{rr\_still\_birth\_maternal\_malaria}) \\
&* (ps\_syphilis \; * \mathbf{rr\_still\_birth\_maternal\_syphilis}) \\
&* \; (ps\_htn\_disorders\_pe * \mathbf{rr\_still\_birth\_pre\_eclampsia}) \\
&* (ps\_htn\_disorders\_ec \; * \mathbf{rr\_still\_birth\_eclampsia}) \\
&* \; (ps\_htn\_disorders\_gh \; * \mathbf{rr\_still\_birth\_gest\_htn}) \\
&* \; (nc\_hypertension * \mathbf{rr\_still\_birth\_chronic\_htn}) \\
&* (ps\_antepartum\_haem \; * \mathbf{rr\_still\_birth\_aph}) \\
&* \; (ps\_chorioamnionitis * \mathbf{rr\_still\_birth\_chorio}) \\
&* (ac\_receiving\_bep\_supplements \\
&* \mathbf{treatment\_effect\_still\_birth\_food\_supps}) \\
&* (ps\_gest\_diab \; * \mathbf{treatment\_effect\_gdm\_case\_management})
\end{aligned}
\tag{28}
$$

To replicate the effect of post-term gestation on risk of stillbirth, the risk of stillbirth is applied in a weekly time step to all women whose pregnancy continues past 40 weeks. Equation 28 is calculated for each post term woman with the final risk divided by 4.348 (estimated weeks in a month) to give a weekly risk of stillbirth which will have been increased due to their GA as evident from parameters in Table S52.

In addition to the effect of GA and maternal conditions, receipt of balanced energy and protein supplementation in at risk women may reduce overall risk of pregnancy loss via the parameter **treatment_effect_still_birth_food_sups** and is administered during ANC. Additionally for mothers with gestational diabetes, who are at increased risk of stillbirth, that have received treatment, the parameter **treatment_effect_gdm_case_management** is applied to reduce this risk. If a pregnancy will end in an antenatal stillbirth, then the stillbirth is logged, the relevant pregnancy variables are updated, and the pregnancy ends.

For simplicity, women undergoing antenatal stillbirth do not currently progress through the labour module and as such they do not use healthcare resources through delivery or occur

risk of labour or postnatal related outcomes. This was deemed acceptable as antenatal stillbirths likely constitute less than one percent of total births in Malawi (281).

3.1.14.2.2 Intrapartum stillbirth

Following the application of the risk that an individual will develop any complications in labour, and whether they will receive treatment, the probability that the individual will experience an intrapartum stillbirth is calculated as follows and applied to all women:

$$\begin{aligned}
\mathrm{Y} = \mathbf{prob_ip_still_birth} * (&la_uterine_rupture * \mathbf{rr_still_birth_ur}) \\
&* (la_obstructed_labour * \mathbf{rr_still_birth_ol}) \\
&* (la_antepartum_haem * \mathbf{rr_still_birth_aph}) \\
&* (ps_htn_disorders * \mathbf{rr_still_birth_hypertension}) \\
&* (la_sepsis * \mathbf{rr_still_birth_sepsis}) \\
&* (ps_multiple_pregnancy * \mathbf{rr_still_birth__multiple_pregnancy}) \\
&* (mni_delivery_mode_avd * \mathbf{treatment_effect_avd_still_birth}) \\
&* (mni_delivery_mode_cs * \mathbf{treatment_effect_cs_still_birth})
\end{aligned} \quad (29)$$

As evident from the variables in equation 29, risk of intrapartum stillbirth is applied sequentially to the risk of maternal death to allow for maternal death to be a predictor in stillbirth risk but also allows for foetus to survive following intrapartum maternal death (298). Additionally, it is here that the effect of AVD and CS are applied to reduce risk of stillbirth through the treatment parameters **treatment_effect_avd_still_birth** and **treatment_effect_cs_still_birth** (see Table S52) is applied. Following stillbirth, live birth is blocked in the model meaning a new individual is not appended to the data frame and is logged accordingly.

In the case of multiple pregnancy, if an intrapartum stillbirth will occur, then parameter **prob_both_twins_ip_still_birth** is used to determine if both foetuses will die during labour or if one will survive- currently it is assumed that the probability of the death of both foetuses is very high. Women experiencing intrapartum stillbirth enter the postnatal model like all other women and are eligible to experience complications and seek care during this time.

3.1.14.3 Data sources and parameters

Parameter Name	Description	Value*	Source and/or relevant calculation
Antenatal parameters			
**prob_still_birth_per_month**	This parameter is scaled at intialisation of the simulation to account for the proportion of women at baseline who have diabetes mellitus and malaria.<br><br>Once scaled, as the simulation runs, this parameter represents the probability that a pregnant woman, whose GA is less than 40, does not have GDM, does not have diabetes mellitus, does not have malaria, does not have syphilis, does not have pre-eclampsia or eclampsia, does not have gestational hypertension, does not have chronic hypertension and has not experience an APH will experience an antenatal stillbirth after 28 weeks GA.<br><br>The parameters in this section ("*Antenatal parameters*"), whose name initiates with "rr_still_birth" indicate different effects on the risk of antenatal stillbirth.	0.0031 / 0.0026	The total stillbirth rate in Malawi was sourced from the UN Inter-agency Group for Child Mortality Estimation group estimates for Malawi in 2010 and 2015 (281). The authors estimate the stillbirth rate in Malawi as 19.76 per 1000 births in 2010 and 17.27 in 2015.<br><br>In keeping with estimates from other settings (299) it is assumed that approximately 50% of stillbirths occur in the antenatal period and 50% in the intrapartum period leading to a rate of antenatal and intrapartum stillbirth of 9.88 per 1000 births in 2010 and 8.64 per 1000 births in 2015.
**rr_still_birth_ga_41**	The effect of a woman's pregnancy continuing to 41 weeks compared to delivering at term	2.24	Sourced directly from Muglu et al. (174) who report the effect of GA in weeks on risk of stillbirth via a systematic review and meta-analysis of cohort studies leading to a sample of over 15 million pregnancies.

**rr_still_birth_ga_42**	The effect of a woman's pregnancy continuing to 42 weeks compared to delivering at term	3.88	See rr**_still_birth_ga_41**
**rr_still_birth_ga_>42**	The effect of a woman's pregnancy continuing to and beyond 42 weeks compared to delivering at term GA	6.94	See rr**_still_birth_ga_41**
**rr_still_birth_gest_diab**	The effect of a pregnant woman experiencing GDM	3.91	Sourced directly from Tabatabaee et al. (291), a case control study conducted in Iran.
**rr_still_birth_diab_mellitus**	The effect of a pregnant woman experiencing non-gestational diabetes mellitus	3.52	Sourced directly from Yu et al. (292) who estimate the effect of pre-gestational diabetes on stillbirth risk via systematic review and meta-analysis of over 100 studies leading to a sample of over 40 million pregnancies.
**rr_still_birth_maternal_malaria**	The effect of a pregnant woman experiencing malaria infection	1.81	Sourced directly from Moore et al. (289) a systematic review and meta-analysis of over 59 studies leading to a sample of over 140,000 women.
**rr_still_birth_maternal_syphilis**	The effect of a pregnant woman experiencing syphilis infection	6.87	Sourced directly from Arnesen et al. (151), a retrospective study conducted in 11 countries across Latin America and the Caribbean.
**rr_still_birth_pre_eclampsia**	The effect of a pregnant woman experiencing pre-eclampsia	4.15	Sourced directly from Xiong et al. (290) who estimate the effect of the hypertensive disorders of pregnancy on risk of stillbirth using data from nearly 6 million births in China.
**rr_still_birth_eclampsia**	The effect of a pregnant woman experiencing eclampsia	4.15	See **rr_still_birth_pre_eclampsia.**

**rr_still_birth_gest_htn**	The effect of a pregnant woman experiencing gestational hypertension	1.21	See **rr_still_birth_pre_eclampsia.**
**rr_still_birth_chronic_htn**	The effect of a pregnant woman experiencing chronic hypertension	2.32	See **rr_still_birth_pre_eclampsia.**
**rr_still_birth_aph**	The effect of a pregnant woman experiencing APH	2.1	See **rr_still_birth_pre_eclampsia.**
**rr_still_birth_chorio**	The effect of a pregnant woman experiencing antepartum chorioamnionitis	2	Sourced directly from McClure et al. (288) who estimate the effect of maternal infection on stillbirth risk via literature review.
**treatment_effect_still_birth_food_sups**	The effect of a pregnant woman receiving daily balanced energy and protein supplementation during pregnancy on her risk of antenatal stillbirth	0.6	Sourced directly from Ota et al. (43), a Cochrane review of published RCTs. They report an effect of RR 0.60, (95% CI 0.39 to 0.94)
**treatment_effect_gdm_case_management**	The effect of effective management of gestational diabetes on risk of antenatal stillbirth	0.9	Sourced directly from Syed et al. (300), a Delphi survey of relevant experts.
Intrapartum parameters			
**prob_ip_still_birth**	The probability that a pregnant woman in labour who survives labour, does not experience uterine rupture, obstructed labour, APH, maternal sepsis, does not have hypertension and is not pregnant with twins will experience an intrapartum stillbirth.	0.006 / 0.005	See **prob_still_birth_per_month.**

	The parameters In this section (“*Intrapartum parameters*”), whose name contains “rr” or “effect” indicate different effects on the risk of intrapartum stillbirth		
**rr_still_birth_maternal_death**	The effect of maternal death	180	Assumption. (180*0.005 = 0.9 risk of stillbirth in case of maternal death).
**rr_still_birth_ur**	The effect of maternal uterine rupture	56.6	Sourced directly from Motomura et al. (254) a study using data from the WHO Multicountry Survey on Maternal and newborn Health.
**rr_still_birth_ol**	The effect of maternal obstructed labour	4.5	Sourced directly from Ashish et al. (287), a case control study of mothers in Nepal.
**rr_still_birth_aph**	The effect of maternal APH	2.1	See **rr_still_birth_ol.**
**rr_still_birth_hypertension**	The effect of maternal hypertension	4.5	See **rr_still_birth_ol.**
**rr_still_birth_sepsis**	The effect of maternal sepsis	2	See **rr_still_birth_ol.**
**rr_still_birth_multiple_pregnancy**	The effect of multiple pregnancy	3	See **rr_still_birth_ol.**
**prob_both_twins_ip_still_birth**	The probability that a pregnant woman who will experience intrapartum stillbirth will experience stillbirth of both foetuses in the context of twin pregnancy	0.8	This parameter has been approximated under the assumption that risk of death for both foetuses is high in the context of intrapartum stillbirth.

**treatment_effect_avd_still_birth**	The effect of AVD	0.2	I was unable to identify research which quantified the effect of AVD on risk of stillbirth or perinatal death when compared to no treatment. A previous systematic review was similarly unable to identify studies estimating the effect of AVD (301) and recent Cochrane reviews are limited to antenatal interventions (302). As such this value is an assumption.<br><br>It was noted after the model had been calibrated that the LiST team estimate that BEmONC and CEmONC could reduce stillbirths by 45% and 75% respectively (303). This estimate could be used in the model going forward and is notably more conservative than the assumption used here.
**treatment_effect_cs_still_birth**	The effect of CS delivery	0.1	See **treatment_effect_avd_still_birth.**

** Where two values (or sets of values) are provided the first set is applied from 2010-2014 and the second set from 2015 onwards for a given simulation run (§1.2.1.1)*

Table S53 – Parameters of the stillbirth models

3.1.15 Obstetric Fistula

3.1.15.1 Condition overview

Anatomically, a fistula is an abnormal connection between two hollow epithelised surfaces. A fistula may develop because of processes associated with labour and delivery and are classically categorised as: vesicovaginal if there is 'an abnormal opening between the bladder and the vagina that results in continuous and unremitting urinary incontinence' (304) or rectovaginal if there is 'an abnormal epithelial-lined connections between the rectum and vagina (305).

Within Malawi, and other African settings, the burden of obstetric fistula is largely driven by delays in women receiving adequate care around the time of delivery, especially in the context of prolonged and/or obstructed labour (25). In this situation, prolonged compression of the soft tissues of the pelvis by the presenting part of the foetus leads to tissue ischaemia followed by necrosis and fistula formation (306). As such, obstructed labour was selected as the primary predictor of fistula in the model, as supported by several studies conducted in Uganda and Kenya which demonstrated the role of prolonged labour on fistula risk (242,307). The impact of fistula on women is profound and complex. It can lead to clinical outcomes such as incontinence and repeated infection, with consequent nuanced social outcomes, such as degradation of marital relationships and community stigmatisation (245,308,309).

Within LMIC settings the estimated pooled prevalence of fistula, using data from population-based surveys, is reported as 0.29 (95% CI 0.00, 1.07) fistula per 1000 women of reproductive age, with significantly higher prevalence in SSA (1.60 (95% CI 1.16, 2.10)) (310). Although often due to different causal mechanisms, obstetric fistula can and does occur in high income settings, with a large study in Norway reporting a rate of 0.16 per 1000 deliveries (95% CI 0.10–0.26). In this study almost all cases were iatrogenic or secondary to trauma, as opposed to obstructed labour (311).

Estimates of fistula prevalence in Malawi vary according to source. In the 2015-2016 DHS survey 0.6% of interviewed women of reproductive age reported having experienced fistula

in their lifetime, which is equivalent to 6 per 1000 women of reproductive age (11), which is over three times greater than estimated by Adler et al. (310) for the region of SSA. However, a community and facility-based survey study across nine districts in Malawi estimated a lower prevalence than the DHS: 1.6 per 1000 women who experienced fistula (312). For the purposes of the model, we have calibrated to the DHS estimates of fistula as the survey is a population level national survey and therefore more likely to be a reliable estimate.

3.1.15.2 Model

A per delivery risk of fistula is applied to all women following birth. Risk of fistula onset is calculated as:

$$Y = \mathbf{prob\_obstetric\_fistula} * (la\_obstructed\_labour * \mathbf{rr\_obstetric\_fistula\_obstructed\_labour}) \quad (30)$$

The constant for this equation, parameter **prob_obstetric_fistula**, represents risk of fistula in the absence of obstructed labour with this risk being significantly increased in the context of this complication (313). If a fistula occurs, a probability weighted random draw, using the probabilities in parameter **prevalence_type_of_fistula,** assigns whether this fistula is either vesicovaginal or rectovaginal to capture morbidity through the specific DALY weights introduced in §1.2.5.3.1.

3.1.15.2.1 Treatment

In Malawian clinical guidelines surgical repair of obstetric fistula is indicated for cases within 8 weeks of delivery, following the administration of several potential investigative tests (such as the dye test) (32). Currently surgical management of fistula is assumed to be 100% effective for all women who seek care for treatment in the model. Treatment resets the variable denoting current fistula for a given individual which in turn removes the disability weight associated with fistula for that individual – as symptoms will now have resolved.

3.1.15.3 Data sources and parameters

Parameter Name	Description	Value*	Source and/or relevant calculation
**prob_obstetric_fistula**	The per- delivery probability that a postnatal woman who has given birth and has not suffered obstructed labour will experience an obstetric fistula	0.00426	The Malawi 2015-16 DHS estimates there is a prevalence of obstetric fistula of 0.6% within surveyed women aged 15-49 (11). This parameter has been derived by calibration to that assumed prevalence.
**rr_obstetric_fistula_obstructed_labour**	The effect of a postnatal woman having experienced obstructed labour in her most recent birth per-delivery risk of developing obstetric fistula on a multiplicative scale	14.8	Sourced directly from Lewis-Wall et al. (313) who report characteristics associated with obstetric fistula from a case control study conducted in Ethiopia. Study uses variable 'labor lasting longer than 1 day' which has been assumed to reflect obstructed labour for the purposes of the model.
**prevalence_type_of_fistula**	The probabilities of an obstetric fistula being vesicovaginal or rectovaginal	[0.924, 0.076]	Sourced directly from Rijken & Chilopora (314) in which the authors report clinical features of 407 fistula cases in Malawi.

Table S54 – Parameters of the obstetric fistula model

3.2 Neonatal complication models

3.2.1 Complications of prematurity

Complications associated with preterm birth are a leading cause of newborn death and disability globally (169) particularly in settings like Malawi where the rate of preterm birth is higher than the global average (175). Here, how these complications are represented within the model to simulate the effect of prematurity on newborn outcomes whilst maintaining model parsimony is described.

Within neonatal epidemiology, complications associated with preterm birth have often been unified as one distinct contributing cause of death, often referred to simply as 'prematurity' (170). However, there are numerous distinct physiological complications caused or exacerbated by prematurity which may contribute to death and disability, which vary between high- and low-income settings and GA at delivery (315). Studies evaluating cause-specific mortality in preterm neonates within east Africa suggest that the leading causes of neonatal death in this group are respiratory distress syndrome, infection, 'birth asphyxia' and 'other' causes (including intraventricular haemorrhage, necrotising enterocolitis, congenital anomalies, and others) (170).

To replicate this the following conditions which can cause preterm death are explicitly modelled: preterm respiratory distress syndrome (§3.2.1.1), neonatal encephalopathy (§3.2.2) and early-onset and late onset neonatal sepsis (§3.2.3). Where supported by evidence prematurity 'status' is included as a predictor in models calculating incidence of these complications, such as with neonatal sepsis. Other causes of preterm death are not explicitly included but are captured via a fixed case-fatality rate applied to all preterm new-borns as described below. In addition, due to evidence of an increasing incidence in SSA (316), retinopathy of prematurity is modelled as a driver of morbidity within this population as described below.

3.2.1.1 Preterm respiratory distress syndrome

Respiratory distress syndrome (RDS) in neonates is characterised by impairment to spontaneous respiration following birth and occurs most frequently in preterm infants

(317). The aetiology of RDS is attributed to “developmental insufficiency of surfactant production and function, as well as by structural immaturity of the lungs” (317). Due to the relationship between RDS and lung development, the incidence of RDS appears much greater in neonates born at an earlier GA and may be as high as 60% in infants born before twenty-eight weeks GA (318,319). Whilst the majority of RDS cases occur in preterm neonates the condition can onset in term and post term infants but is very infrequent (320) and therefore risk of RDS in the model has only been applied to preterm neonates.

Aside from prematurity, the primary predictors of RDS in preterm neonates is maternal diabetes mellitus in pregnancy (321). Evidence suggests that both maternal gestational and non-gestational diabetes are associated with an increased risk of RDS, possibly due to the effect of maternal diabetes on phosphatidylglycerol secretion, an essential component of surfactant, and the relationship between hyperglycaemia, insulin and gene expression of key surfactant proteins in the lungs (321).

Outcomes associated with RDS are often poor, and the condition remains a leading cause of death of preterm infants within African settings (170,322) with data from a neonatal intensive care unit (NICU) within Ethiopia suggesting as many as forty-five percent of all RDS cases admitted to the unit dying prior to discharge (323).

There is limited data on the global incidence of preterm RDS, however large observational studies from high income settings, such as the US, report that RDS is a common complication of prematurity and that in 2014 there were 361 cases of RDS per 1000 preterm live births across the country (324). Facility-based studies conducted in Ethiopia report the incidence of RDS in preterm neonates admitted to the NICU (170,323). Birihane et al. (323) reported that 40% (95% CI 35.8, 44.3) of preterm neonates admitted to the NICU during their study period were diagnosed with RDS and similarly, Muhe et al. (170) report that during their multi-site prospective study that the admitting diagnosis for 45% of newborns was RDS. As we were unable to identify sources of data from Malawi reporting the incidence of RDS, the incidence as 180 cases per 1000 preterm births is estimated using data from the Ethiopian studies above and an assumed proportion of preterm neonates requiring NICU care as described further in Table S54 in this section.

3.2.1.1.2 Model

Figure S28 below describes the model of RDS whilst Table S54 contains the model parameters.

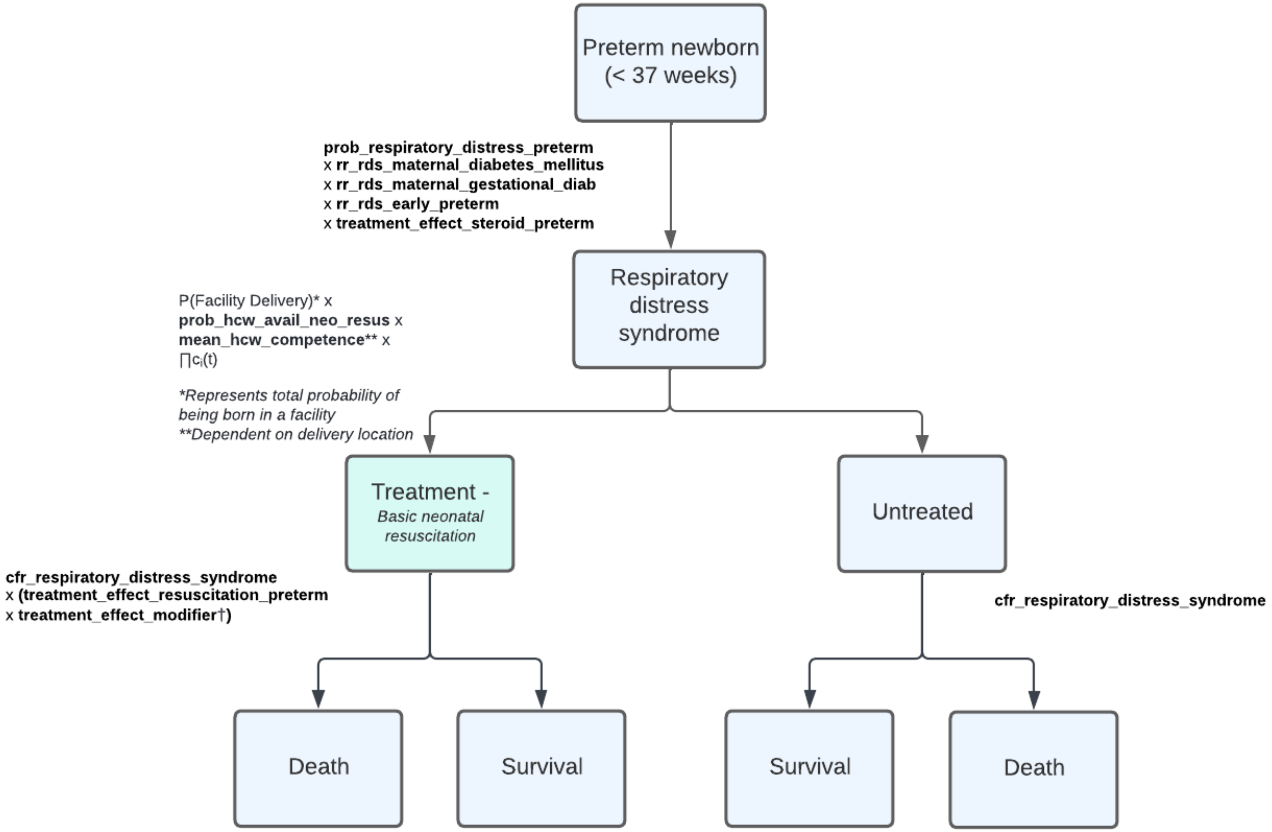

Figure S28 – Model of preterm respiratory distress syndrome
Diagrammatic representation of the model of preterm respiratory distress syndrome. Light blue represents the model's natural history without treatment whilst teal represents treatment pathways. Parameters representing progression through model states are shown here.

The risk of RDS onset is applied to all preterm neonates following birth. Individual risk is calculated as:

$$\begin{aligned} \mathrm{Y} = &\ \mathbf{prob\_respiratory\_distress\_syndrome} \\ &* (ni\_diabetes * \ \mathbf{rr\_rds\_maternal\_diabetes\_mellitus}) \\ &* (ps\_gest\_diab * \mathbf{rr\_rds\_maternal\_gest\_diab}) \\ &* (nb\_early\_preterm * \mathbf{rr\_rds\_early\_preterm}) \\ &* (nci\_corticosteroids\_given * \mathbf{treatment\_effect\_steroid\_preterm}) \end{aligned} \quad (31)$$

The parameters are described in Table S54. The parameter **cfr_respiratory_distress_syndrome** represents the risk of death associated with RDS which is applied to all RDS cases, allowing for the administration of treatment where indicated.

3.2.1.1.2.1 Treatment

Delivery of intravenous corticosteroids to women at risk of preterm birth (i.e. those in preterm labour, undergoing planned preterm delivery or experiencing PROM) is a well-established intervention shown to be effective in reducing the risk RDS and mortality in the preterm infants of these mothers by accelerating the production of pulmonary surfactant (62). There have been some concerns that the evidence base purported to support the use of steroids has largely been derived from studies conducted in HIC, and that administration may be associated with unintended neonatal and maternal complications following results from a large trial intending to increase steroid use in LMIC (325). However the use of antenatal steroids is be recommended by the WHO in their 2022 guidelines following the recent publication of new evidence of effectiveness (326). In addition, the administration of steroids to these mothers is recommended in the Malawian EHP, Obstetric and Standard Treatment guidelines (31,32,60). Within the model the administration of steroids during preterm labour reduces the probability of neonatal preterm RDS onset as shown in equation 31.

Additionally, all newborns who are delivered in health facilities are assessed to determine if neonatal resuscitation is required. It is assumed all preterm newborns with RDS will require resuscitation to reduce likelihood of death post-delivery as recommended through

discussion with clinical experts (327). The parameter **treatment_effect_resuscitation_preterm** is the effect of basic newborn resuscitation on risk of death secondary to RDS and, at the time of writing, treatment for RDS within the model is currently limited to basic neonatal resuscitation as higher-level care is not explicitly modelled.

3.2.1.1.3 Data sources and parameter values

Parameter Name	Description	Value*	Source and/or relevant calculation
**prob_respiratory_distress_preterm**	The probability that a preterm neonate born to a mother that does not have gestational diabetes or diabetes mellitus and was born after 34 weeks will experience preterm RDS following birth<br><br>The parameters below starting with "rr_rds" refer to the effect on the probability of RDS	0.12	Due to lacking data from Malawi reporting the incidence of RDS in the preterm population we have estimated the rate to which the model is calibrated.<br><br>Data from Ethiopia suggests that approximately 40% of preterm NICU admission suffer from RDS (170,323). Assuming approximately 40% of preterm neonates require NICU care (328) The total RDS rate is calculated as 40 * 0.40 = 18 preterm RDS cases per 100 preterm newborns (180 per 1000 preterm births). Whilst sufficient for the model's current purpose this is likely an underestimate due to cases of untreated RDS.
**rr_rds_maternal_diabetes_mellitus**	The effect of a neonate's mother experiencing non-gestational diabetes mellitus	2.66	Sourced directly from Li et al. (329) a systematic review of 24 studies across several settings.
**rr_rds_maternal_gestational_diab**	The effect of a neonate's mother experiencing gestational diabetes mellitus	1.57	See **rr_rds_maternal_diabetes_mellitus.**
**rr_rds_early_preterm**	The effect of a neonate being born before 34 weeks GA	2.64	Sourced directly from Birihane et al. (323) who explore the determinant factors of RDS via an institution-based retrospective study in Ethiopia of 535 preterm infants.
**treatment_effect_steroid_preterm**	The effect of a preterm neonate's mother receiving antenatal corticosteroids on a neonate's risk of developing preterm RDS	0.71	Sourced directly from McGoldrick et al. (62) who estimate the effect of the intervention as RR 0.71, (95% CI 0.65 to 0.78) through a Cochrane review of RCTs.

**treatment_effect_resuscitation_preterm**	The effect of a preterm neonate receiving basic neonatal resuscitation on risk of death secondary to preterm RDS	0.81	Sourced directly from Lee et al. (64). The authors estimate the effect of immediate assessment and stimulation and basic newborn resuscitation on risk of preterm death separately. Both are estimated to reduce risk of death by 10% leading to a final treatment effect of 0.81 (1 – (0.9x0.9) = 19% reduction).
**cfr_respiratory_distress_syndrome**	The probability that a preterm neonate will die due to preterm respiratory distress syndrome without treatment	0.29 / 0.2	The model has been calibrated to both the reported NMR in 2010 and 2015 sourced from the Malawian DHS surveys in those years (11,12) and the proportion of total direct neonatal deaths by cause sourced from the 2010 and 2015 Malawian EmONC needs assessments (33,34).<br><br>As such untreated case fatality parameters have been estimated through the process of calibration to ensure that the model replicates both the assumed NMR and the proportion of deaths by cause.

** Where two values (or sets of values) are provided the first set is applied from 2010-2014 and the second set from 2015 onwards for a given simulation run (§1.2.1.1)*

Table S55 – Parameters describing preterm respiratory distress model

3.2.1.2 Other unmodelled causes of preterm mortality

To reduce model complexity, additional complications associated with prematurity that may cause neonatal death (i.e., necrotising enterocolitis, intraventricular haemorrhage, hypothermia etc) are not explicitly modelled. To capture the associated risk of death from such complications, and other factors relating to prematurity, probability of death is calculated for each preterm neonate via the following equation:

$$\mathrm{Y} = \mathbf{cfr\_preterm\_birth} * (nb\_early\_preterm * \mathbf{rr\_preterm\_death\_early\_preterm}) * (nb\_kmc * \mathbf{treatment\_effect\_kmc}) \quad (32)$$

Within this equation it is assumed that early preterm neonates are at greater risk of death than their late preterm counterparts which is widely supported by evidence (315, 330). In addition, it is here that the effect of KMC is applied to risk of death for those neonates who will receive this treatment postnatally – see §2.6.2.2.

Individual risk of death calculated via equation 32 is applied to all preterm neonates with the date of death being distributed across the first 14 days of life to prevent clustering. Parameter **prob_preterm_death_by_day**, Table S55, is the probability death will occur on each of these days and generates a distribution skewed towards the first day of birth where most newborn deaths are clustered (334).

Parameter Name	Description	Value*	Source and/or relevant calculation
cfr_preterm_birth	The probability that a preterm neonate will die due to complications associated with prematurity not explicitly modelled	0.0227 / 0.0187	See **cfr_respiratory_distress_syndrome** in Table S53.
**rr_preterm_death_early_preterm**	The effect of a neonate being born before 34 weeks GA on their risk of death due complications associated with prematurity not explicitly modelled	1.66	Sourced directly from Gou et al. (330) who report the determinants of preterm mortality via multivariate regression using data on 2651 preterm births across China.
**treatment_effect_kmc**	The effect of a preterm neonate receiving KMC on their risk of death secondary to unmodelled complications associated with prematurity	0.49	Sourced directly from Lawn et al. (72) who conducted a meta-analysis of RCTs evaluating the effect of KMC on neonatal mortality in preterm infants. They report an effect of 0.49 (95% CI 0.29-0.82).
**prob_preterm_death_by_day**	The probabilities that a neonate who will die due to unmodelled complications associated with prematurity will die on one of the first 14 days of life	[0.4, 0.2, 0.15, 0.05, 0.02, 0.018, 0.018, 0.018, 0.018, 0.018, 0.018, 0.018, 0.018, 0.018, 0.018]	These values have been estimated to prevent clustering of deaths on the first day of life. The deaths were spread across the first 14 days under the assumption that the probability of preterm death is greatest closest to birth (330).

** Where two values (or sets of values) are provided the first set is applied from 2010-2014 and the second set from 2015 onwards for a given simulation run (§1.2.1.1)*

Table S56 – Parameters describing treatment and risk of death associated with unmodelled causes of preterm mortality

3.2.1.3 Retinopathy of prematurity and other preterm morbidity

In addition to mortality associated with prematurity, long term morbidity is captured within the model both through explicit representation of retinopathy of prematurity (ROP) and through application of risk of long-term neurodevelopmental impairment in preterm infants. ROP is an emerging disease across SSA, attributed in part to advances in health system development and neonatology leading to an increased proportion of preterm neonates receiving oxygen therapy, as the administration of high concentration of oxygen in neonatal care has been identified as a causal influence on ROP (332).

ROP is explicitly modelled due to its role in long term morbidity associated with preterm birth. For preterm neonates who survive the first 28 days of life, risk of ROP is applied according to GA at birth with parameter **prob_retinopathy_preterm_early** being the probability of any ROP in those born before 32 weeks and **prob_retinopathy_preterm_late** being the probability in those born after 32 weeks but before 37 weeks. If ROP will occur then severity is determined via a probability weighted random draw with **prob_retinopathy_severity_no_treatment** (Table S14) being the probability of ROP having no effect on visual acuity or leading to mild, moderate, or severe impairment or blindness. These categories match to the disability weights described in Table S13.

Whilst there is evidence of ophthalmologists in Malawi treating cases of ROP (333) we were unable to identify any data on coverage of treatment or recommended treatment in available clinical guidelines. Therefore there is no treatment included in the model for ROP and its effects are currently assumed to be permanent, meaning a lifelong disability weight is applied to affected individuals. As described in Table S14 these parameters are informed by Blencowe et al. (16) who estimated the global and regional incidence of ROP in 2010 using available data sources.

Parameter Name	Description	Value	Source and/or relevant calculation
**prob_retinopathy_preterm_early**	The probability that a neonate born before 32 weeks GA will develop ROP	0.365	Sourced directly from Blencowe et al. (16) in which the authors estimate the incidence of any ROP in survivors of preterm birth via meta-analysis. They report an incidence of 36.5% (95% CI: 31.8, 41.1%) in neonates born before 32 weeks.
**prob_retinopathy_preterm_late**	The probability that a neonate born after 32 weeks and before 37 weeks GA will develop ROP	0.077	See **prob_retinopathy_preterm_early**. Blencowe et al. (16) estimate that 7.7% (95% CI: 6.7, 8.7%) survivors of preterm birth in neonates born after 32 weeks experience ROP.

Table S57 – Parameters describing probability of ROP in preterm neonates

3.2.2 Neonatal encephalopathy and neonatal respiratory depression

3.2.2.1. Condition overview

Neonatal respiratory depression (NRD), in which a baby is not breathing adequately following birth, can be due to several distinct causes, including conditions explicitly represented in the model such as neonatal encephalopathy (NE) or RDS but also due to other causes not captured such as intracranial or neuromuscular disease, effects of maternal anaesthetic, infection or meconium aspiration (17). As such, in addition to the inclusion of NE and RDS in the model a probability of NRD is applied due to other causes as described further in the following section, which if left untreated can lead to NE secondary to hypoxia (17).

NE, a leading cause of NRD, can be defined as a "syndrome of disturbed neurologic function in the earliest days of life in an infant born at or beyond 35 weeks of gestation, manifested by a subnormal level of consciousness or seizures, and often accompanied by difficulty with initiating and maintaining respiration and depression of tone and reflexes" (334). Commonly applied definitions often exclude very preterm infants due to the difficulty in identifying clinical features of the condition in infants with less mature nervous systems (334). However, there is evidence to suggest encephalopathy does occur within this group (335) and as such risk within the model is applied to all neonates.

NE can onset following several possible causes but is often attributed to intrapartum-related hypoxia, in which prolonged disruption to the exchange of oxygen and carbon dioxide between foetus and mother (i.e., asphyxia) causes cerebral hypoxic-ischaemia that overwhelms foetal compensatory mechanisms (334). Evidence suggests that intrapartum-related hypoxia is the leading cause of NE in both high- and low-income settings (336). Terminology related to intrapartum-related NE varies considerably in the clinical literature due to varying clinical definitions and includes 'birth asphyxia', 'perinatal asphyxia', and 'hypoxic-ischaemic encephalopathy' (334). In the model, in keeping with consensus calls by clinicians and epidemiologists in the field, the term NE is favoured, especially if the aetiology remains unclear to allow for improved epidemiological surveillance (17).

Excluding intrapartum-related causes, there are several other causes of NE including placental insufficiency, perinatal infection, metabolic disorders, malformation or infection of the central nervous system and genetic disorders (336,337). Evaluation of the literature related to predictive factors of NE onset within SSA identified very few studies which applied the definition of NE used here. Tann et al. (338) report predictive factors of NE through an unmatched case-control study conducted in Uganda which included data on preconception, antepartum and intrapartum factors and reported the effect of perinatal infection, obstructed labour and acute intrapartum events (including APH and uterine rupture) on risk of NE as shown in parameter Table S57. This study was chosen due to the case definition used by the authors which matches the definition applied in the model, and after discussion with Dr Tann, who provided validation for the encephalopathy model.

Outcomes of NE vary by severity, which is commonly graded using the Sarnat score (339,340). Sarnat and Sarnat (340) classify NE cases as either stage one (hyperalert), stage two (lethargic/obtunted) or stage three (stuporous) dependent on clinical signs such as neuromuscular control and complex reflex function. Stage three cases of NE are associated with significant risk of death, with Lee et al. (17) estimating a case fatality of 91.7% in countries with NMR > 15. NE of any severity is also strongly associated with long term neurodevelopmental delay with as many as 26.9% of all NE cases experiencing moderate to severe impairment (17).

Estimates of the incidence of NE fluctuate between studies due to variation in the clinical definition of NE used and variation between in-facility and population-based estimates (345). Kurinczuk et al. (341) report an incidence of NE across several countries of between 2 and 6 cases per 1000 live births. In addition to estimated incidence of NE of any aetiology, several studies have estimated the global incidence of NE associated with intrapartum events. Lee et al. (17) report that in 2010 there were approximately 8.5 cases of intrapartum related NE per 1000 live births, leading to 1.15 million (UI 0.89, 1.60 million) cases – of which 96% occurred in LMICs. More contemporary estimates, that focused solely on LMICs, suggest the rates of NE may be even higher and range from 1.5 per 1000 term live births to 20.3 per 1000 term live births in some settings (342). There are no available population or facility-based study estimates of NE using the definition applied here within Malawi. The

GBD study reports estimated number of NE cases attributed to 'birth asphyxia' and trauma per year and reported 11,438 cases in 2019 leading to a rate of 18 per 1000 births (1) which is applied in the model.

3.2.2.2 Model

Figure S29 shows the model of neonatal encephalopathy and Table S57 contains model parameters.

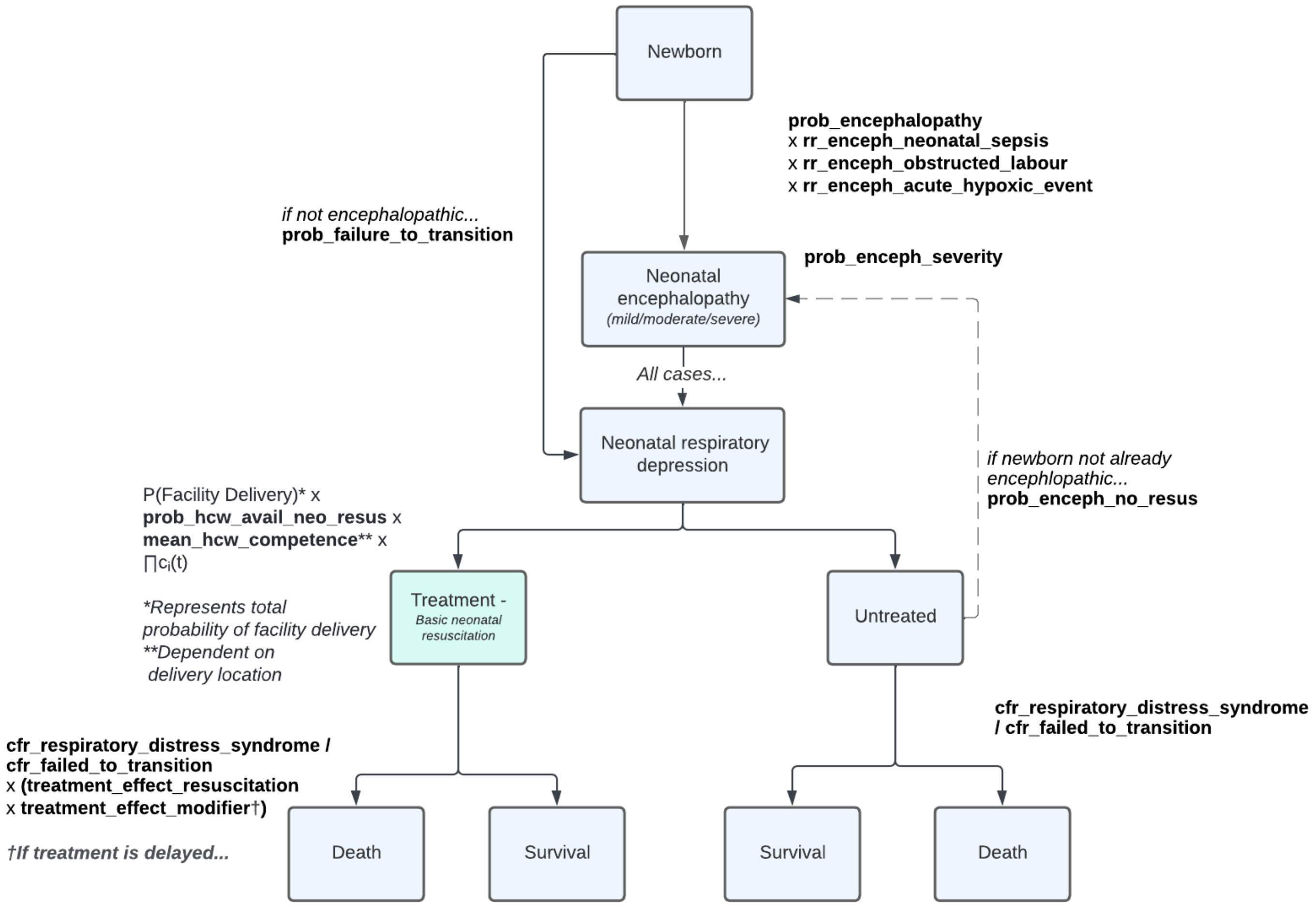

Figure S29 – Model of neonatal encephalopathy and neonatal respiratory depression
Diagrammatic representation of the model of neonatal encephalopathy. Light blue represents the model's natural history without treatment whilst teal represents treatment pathways. Parameters representing progression through model states are shown here.

Initial risk of neonatal encephalopathy is applied to all term and preterm neonates following delivery and is calculated as follows:

$$\begin{aligned} \mathrm{Y} = \mathbf{prob\_encephalopathy} \\ &* (nb\_early\_onset\_neonatal\_sepsis * \mathbf{rr\_enceph\_neonatal\_sepsis}) \\ &* (la\_obstructed\_labour * \mathbf{rr\_enceph\_obstructed\_labour}) \\ &* (la\_uterine\_rupture * \mathbf{rr\_enceph\_acute\_hypoxic\_event}) \\ &* (ps\_antepartum\_haem * \mathbf{rr\_enceph\_acute\_hypoxic\_event}) \end{aligned} \tag{33}$$

In accordance with the definition of NE provided above, and following recommendation from clinical experts, it is assumed that all neonates with NE experience some degree of neonatal respiratory depression requiring intervention as discussed below.

In addition to application of risk of NE to all neonates following birth, it was deemed appropriate to apply risk of encephalopathy to any neonates who are not encephalopathic but are experiencing NRD secondary to other causes as shown in Figure S29. This causal pathway was recommended for inclusion following consultation with experts in the field of NE.

For each new case in the model, severity is assigned using a probability weighted random draw, parameter **prob_enceph_severity**, which generates the correct assumed distribution of mild, moderate, and severe cases estimated to occur within LMICs (17). Severity of NE is associated directly with outcomes (17) and as such it is assumed that the baseline NE case fatality, parameter **cfr_enceph**, is increased in severe cases by multiplying this probability by parameter **cfr_multiplier_severe_enceph** to generate the assumed risk of death for neonates experiencing NE at this severity (Table S57).

Surviving neonates are at risk of lifelong neurodevelopmental impairment, which is captured through the application of disability weights. The parameters **prob_mild_impairment_post_enceph** and **prob_mod_severe_impairment_post_enceph** (Table S14) are the probabilities of impairment used in the model also sourced from Lee et al. (17) in which the authors estimate prevalence of impairment in survivors of NE

regardless of severity. As such, severity grading of NE, determined via **prob_enceph_severity**, does not directly affect probability of long-term impairment which is a limitation of the approach taken in the model.

3.2.2.2.1 Treatment

Modelled treatment for NE is limited to basic neonatal resuscitation which can be delivered to any neonates who are born within a health facility.

3.2.2.3 Data sources and parameters

Parameter Name	Description	Value*	Source and/or relevant calculation
**prob_encephalopathy**	The probability that a neonate who is not septic and whose mother did not have obstructed labour or experience an acute hypoxic event during labour will develop NE following birth.<br><br>The parameters below starting with "rr_enceph" refer to the effect on the probability of NE	0.0097	Due to lacking reliable estimates from other sources the assumed rate of NE in Malawi was derived from the GBD study data (1) by dividing the total NE cases reported by the estimated births for the survey year giving a rate of 18.59 per 1000 live births in 2015 to which the model was calibrated. This parameter was derived through the process of calibration to this rate.
**rr_enceph_neonatal_sepsis**	The effect of a neonate experiencing early-onset neonatal sepsis	8.67	Sourced directly from Tann et al. (338) who report the effect of determinants of NE via a case-control study of encephalopathic neonates in Uganda.
**rr_enceph_obstructed_labour.**	The effect of a neonate's mother having experienced obstructed labour	3.8	See **rr_enceph_neonatal_sepsis**
**rr_enceph_acute_hypoxic_event**	The effect of an acute hypoxic event (APH or uterine rupture) during labour	8.74	See **rr_enceph_neonatal_sepsis**
**prob_enceph_severity**	The probabilities that a neonate who is experiencing NE will experience mild, moderate, or severe NE.	[0.422, 0.338, 0.24]	Sourced directly from Lee et al. (17) who estimate the proportion of intrapartum-related NE cases that are mild, moderate, or severe in countries with an NMR ≥ 15 via a systematic review and meta-analysis
**cfr_enceph**	The probability that a neonate will die due to NE without treatment.	0.48 / 0.28	See **cfr_respiratory_distress_syndrome** in Table S54.

**cfr_multiplier_severe_enceph**	The effect of the most severe stage of NE on risk of death due to NE.	1.91 / 3.28	Lee et al. (17) estimate that in countries with an NMR ≥ 15, 91.8% (83.4-99.4%) of neonates with grade severe NE will die. Using the case fatality probability in parameter **cfr_enceph** these values were estimated to approximate that risk of death in neonates with severe NE without treatment.
**prob_failure_to_transition**	The probability that a neonate who is not encephalopathic and is not experiencing preterm respiratory distress syndrome will not spontaneously breathe following birth	0.01	The model has been calibrated to an assumed total incidence of neonatal respiratory complications (5.74%) sourced from Vossius et al. (343) including NE, preterm RDS and other causes not explicitly modelled which this rate represents.
**prob_enceph_no_resus**	The probability that a neonate who is not encephalopathic but is not breathing spontaneous at birth will develop encephalopathy if not resuscitated	0.5	See **prob_encephalopathy** This parameter was derived from calibration to the overall rate of NE in the model.
**cfr_failed_to_transition**	The probability that a neonate who is not encephalopathic and is not experiencing preterm respiratory distress syndrome but is not breathing spontaneously will die without treatment	0.2	See **cfr_respiratory_distress_syndrome** in Table S54.

**treatment_effect_resuscitation**	The effect of a neonate receiving basic neonatal resuscitation on risk of death secondary to encephalopathy or respiratory depression.	0.63	Sourced directly from Lee et al. (64) who estimate the effect of immediate assessment and stimulation and basic newborn resuscitation on risk of intrapartum-related newborn death separately. Immediate assessment and stimulation is estimated to reduced risk of death by 10% and in-facility basic newborn resuscitation is estimated to reduce risk of death by 30% leading to a final treatment effect of 0.63 (1-(0.9x0.7) = 37% reduction). Effects are estimated via a Delphi survey of relevant experts.

** Where two values (or sets of values) are provided the first set is applied from 2010-2014 and the second set from 2015 onwards for a given simulation run (§1.2.1.1)*

Table S58 – Parameters of the neonatal encephalopathy model

3.2.3 Neonatal sepsis

3.2.3.1 Condition overview

Neonatal sepsis refers to “a systemic condition of bacterial, viral, or fungal (yeast) origin that is associated with haemodynamic changes and other clinical manifestations and results in substantial morbidity and mortality” (344). Commonly, within epidemiology and clinical practice, neonatal sepsis is sub-divided into early- and late-onset disease due to variation in both aetiology and prevention of infections which onset at different time points within the neonatal period (345).

Definitions of early-onset neonatal sepsis (EONS) vary between clinicians and researchers and may include sepsis which manifests in the first seventy-two hours of life up until seven days post birth (344,346,347).Within the model, any case of sepsis which onsets within the first week of life is classified as EONS. EONS is attributed to infections which occur via vertical mother-to-neonate transmission either in utero, trans-placentally or through ascending bacteria that enters the uterus from the genital tract following membrane rupture, or during birth if the neonate is exposed to pathogenic organisms during passage through the birth canal (344–347). Commonly EONS is caused by either Group B streptococcus or Escherichia coli infection, however several other pathogens can lead to sepsis during this period (344).

In keeping with the presumed aetiology of EONS, and following a review of the relevant literature relating to predictors of EONS onset, maternal chorioamnionitis, premature rupture of membranes (348) and prematurity (349) are included as predictors in the model. In addition, and discussed further in the following section, several treatments which have been demonstrated to reduce risk of neonatal infection are modelled.

In contrast to EONS, late onset neonatal sepsis (LONS) can be defined as sepsis secondary to “infections present after delivery, or beyond 3 to 7 days of age, and are attributed to organisms acquired from interaction with the hospital environment or the community” (344). The definition of LONS varies depending on the infective organism and prematurity status of the neonate (344), but for the context of this model the above is the definition applied. There are several potential causative organisms responsible for LONS, such as

Escherichia coli, Klebsiella pneumoniae, and Coagulase-negative Staphylococcus (350,351) and similarly, site of infection can vary considerably with common sites including the blood stream, lungs, urinary tract, and central nervous system (352). To avoid complicating the model further, we have opted not to include predictive factors of LONS here.

Neonatal sepsis is associated with significant mortality, especially in LMICs. Using data from SSA, south Asia, and Latin America, Seale et al. estimate a 9·8% (95% CI 7·4, 12·2) case-fatality rate for neonates with severe bacterial infection (353). Similarly, Fleischmann et al. (354) report that globally, between 1% and 5% of neonates experiencing sepsis die, and 9% to 20% of those experiencing severe sepsis die.

Fleischmann et al. (354) estimated the global incidence of neonatal sepsis via a systematic review and meta-analysis and reported a random effects estimated incidence of both EONS and LONS combined as 2824 (95% CI 1892, 4194) cases per 100,000 live births across populations. When limiting this analysis to studies conducted in LMIC, the authors reported a higher incidence of 3930 (95% CI 1937, 7812) per 100,000 live births (354). Importantly, when limiting their analysis to studies in which EONS and LONS were captured distinctly and without possible overlap they found that the incidence of LONS is 2.6 times less than the incidence of EONS (EONS 2469/100,000 (95% CI 1424, 4250) and LONS (946/100,000 (95% CI 544 to 1642)). Sepsis at any point within the neonatal period can be a cause of long-term neurodevelopmental impairment, which persists well into the life course, especially in neonates who are at high risk due to prematurity and low birth weight (355). Risk of mortality from LONS is high, contributing to the fact that neonatal sepsis remains a leading cause of neonatal and under five mortality globally (169, 356).

As with many of the complications presented thus far, reliable contemporary estimates of neonatal sepsis from Malawi are lacking. Within the model, we have opted to use the estimates produced by Fleischmann et al. (354) described above for calibration of the model to overall sepsis rate as described in Table S58.

3.2.3.2 Models

Figures S30 and S31 describe the models of early- and late-onset neonatal sepsis within the module whilst Table S58 contains model parameters.

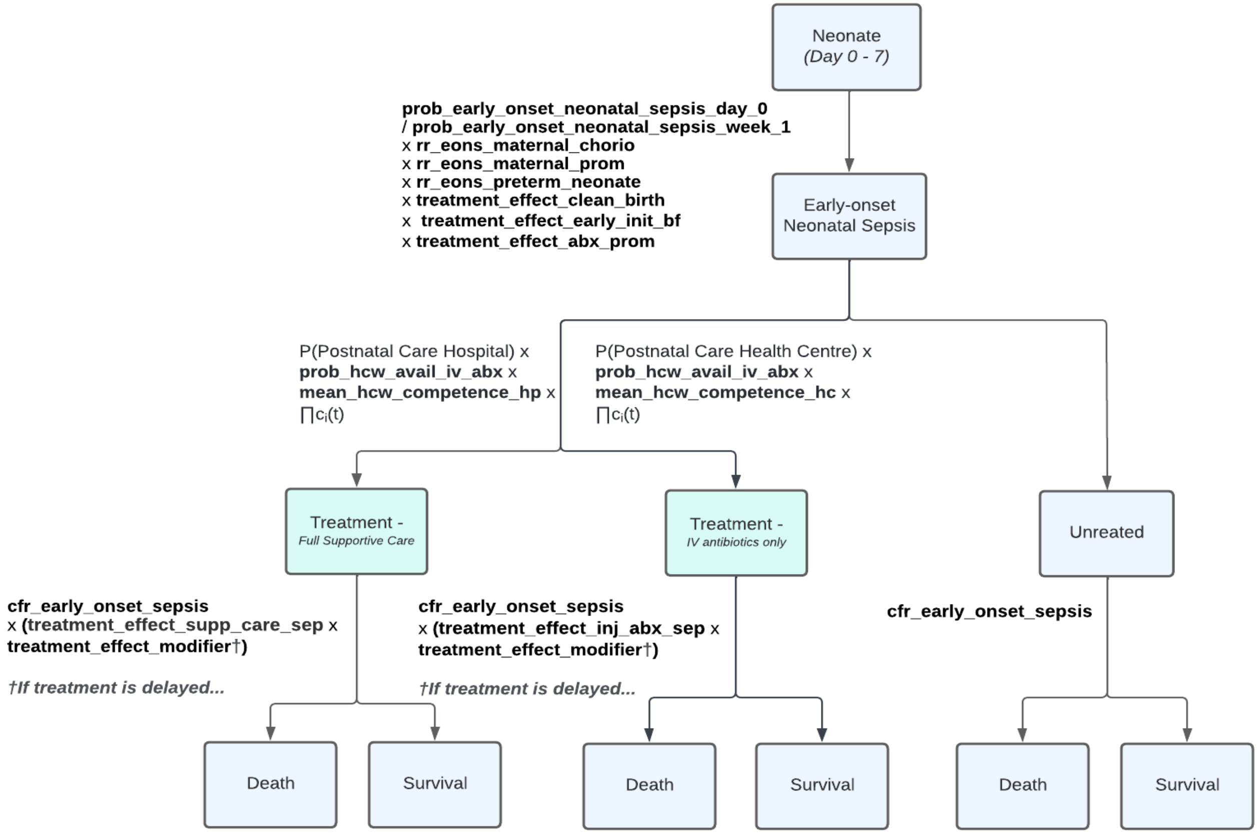

Figure S30- Model of early-onset neonatal sepsis
Diagrammatic representation of the model of early-onset neonatal sepsis. Light blue represents the model's natural history without treatment whilst teal represents treatment pathways. Parameters representing progression through model states are shown here.

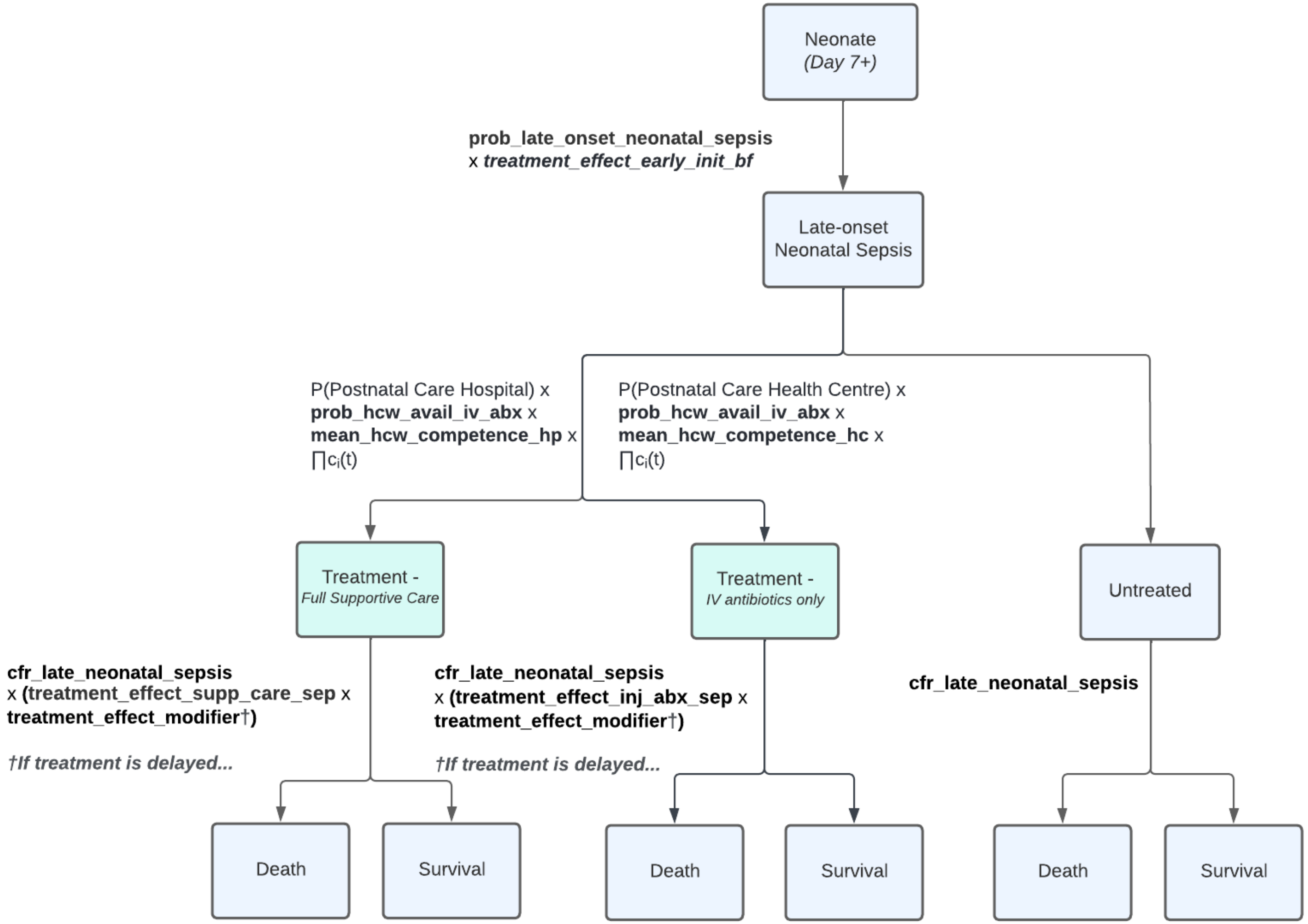

Figure S31 – Model of late-onset neonatal sepsis
Diagrammatic representation of the model of late-onset neonatal sepsis. Light blue represents the model's natural history without treatment whilst teal represents treatment pathways. Parameters representing progression through model states are shown here.

Risk of EONS is applied at two time points within the model. First, risk is applied immediately following birth in keeping with the other conditions discussed in this section and secondly risk is applied again prior to the end of the first week of life. Individual probability of EONS onset is calculated as follows[6]:

$$
\begin{aligned}
\mathrm{Y} = \mathbf{prob\_early\_onset\_sepsis\_neonatal\_day\_0} \\
&* (nci\_maternal\_chorio * \mathbf{rr\_eons\_maternal\_chorio}) \\
&* (ps\_premature\_rupture\_of\_membranes * \mathbf{rr\_eons\_maternal\_prom}) \\
&* (nb\_early\_preterm * \mathbf{rr\_eons\_preterm\_neonate}) \\
&* (nb\_late\_preterm * \mathbf{rr\_eons\_preterm\_neonate}) \\
&* (nb\_clean\_birth * \mathbf{treatment\_effect\_clean\_birth}) \\
&* (nb\_early\_init\_breastfeeding * \mathbf{treatment\_effect\_early\_init\_bf}) \\
&* (nci\_abx\_for\_prom\_given * \mathbf{treatment\_effect\_abx\_prom})
\end{aligned}
$$

(34)

Several prophylactic treatments delivered to both mothers and newborns are included in the model which reduce initial risk of sepsis onset including clean birth practices, early initiation of breastfeeding and, for neonates born to mothers with PROM who received treatment, prophylactic antibiotics. These have been introduced in §2.5.2.

Care for neonates with EONS is initiated via PNC, if care seeking occurs. The probability of death following EONS in the absence of treatment is indicated by the parameter **cfr_early_onset_neonatal_sepsis** in Table S58 and is mitigated by interventions that can be delivered following care seeking for postnatal care, as shown in the figure above and described later in this section.

All neonates who survive following EONS, and survive the remainder of the neonatal period, are at risk of developing long term neurodevelopmental impairment due to their condition. Neonates who develop impairment are currently assumed to experience impairment for life and as such the disability weight for sepsis related impairment, in Table S14, will be

[6] When risk is calculated in week two, intercept parameter name is **prob_early_onset_neonatal_sepsis_week_1**. The value is unchanged.

attached to the individual for the remainder of their life in the simulation contributing to YLD.

Individual probability of LONS onset is applied to all neonates during weeks two, three and four of the neonatal periods. Parameter **prob_late_onset_neonatal_sepsis** is the weekly probability of LONS onset used in this model. As shown in Figure S30 risk of acquisition is reduced for neonates who experienced early initiation of breastfeeding. As with EONS, treatment for LONS is initiated via PNC.

3.2.4.2.1 Treatment

In keeping with global and Malawian clinical guidelines, any neonate displaying signs of sepsis following delivery should receive comprehensive case management starting with the delivery of parenteral antibiotics (66,357). The EHP defined within Malawi's HSSP II differentiates between 'Newborn sepsis – full supportive care' and 'Newborn sepsis – injectable antibiotics' (60) and as such both full supportive care and injectable antibiotics have been conceptualised as two distinct interventions in the model.

A definition of 'full supportive care' is not provided within the EHP and as such we have chosen to adopt the definition provided by Zaidi et al (73). Their definition of 'hospital-based supportive care' includes:

- "Administration of intravenous antibiotics
    - Wider choice of antibiotics including broad spectrum antibiotics
    - Option of using frequent/higher dosage if needed to maintain high blood antibiotic levels or coverage for meningitis
    - Access to second-line antibiotic therapy for neonates with treatment failure on first line antibiotics
- Intravenous access and administration of intravenous fluids if needed
- Oxygen supplementation if required
- Access to appropriate diagnostic procedures, such as monitoring of pulse, blood pressure, and oximetry reading, as well as monitoring/correction of hypoglycaemia if required"

To align with this definition, it is assumed in the model that management of neonatal sepsis varies according to the level of health facility in which care for sepsis is sought. If a neonate receives PNC at a lower lower-level facility (i.e., a health centre) septic individuals may receive treatment with parenteral antibiotics, **treatment_effect_inj_abx_sep**, but do not receive full case management which is restricted to higher-level facilities. Alternatively, those presenting at higher-level facilities may receive ‘full supportive care’ which is more effective at reducing risk of death, **treatment_effect_sup_care_sep** as shown in Table S58. Treatment does not vary between EONS or LONS cases.

3.2.3.3 Data sources and parameters

Parameter Name	Description	Value	Source and/or relevant calculation
prob_early_onset_neonatal_sepsis_day_0	The probability that a neonate will develop EONS immediately after delivery.<br><br>The parameters below starting with "rr_eons" refer to the effect on the probability of EONS	0.02 / 0.017	Due to limited data on neonatal sepsis rates in Malawi the assumed incidence was sourced from a systematic review and meta-analysis of studies estimating incidence by Fleischmann et al. (354).The authors estimate a total incidence of neonatal sepsis in LMICs of 3930 (95% CI 1937 to 7812) per 100,000 live births. From that study the authors report that approximately 75% of total neonatal sepsis cases are early onset whilst the remaining are late onset. As such, a rate of 29.48 EONS cases per 1000 live births and 9.82 LONS cases per 1000 live births is assumed.
**rr_eons_maternal_chorio**	The effect of a neonate's mother having experienced sepsis secondary to chorioamnionitis	6.6	Sourced directly from Chan et al. (348) who estimate the effect of maternal infection or colonisation on risk of early onset neonatal sepsis via a global systematic review and metanalysis of 83 studies.
**rr_eons_maternal_prom**	The effect of a neonate's mother having experienced PROM	4.9	See **rr_eons_maternal_chorio.**
**rr_eons_preterm_neonate**	The effect of a neonate being preterm	3.36	Sourced directly from Belachew et al (349) who estimate the effect of prematurity on risk of neonatal sepsis via a systematic review of 8 studies conducted in Ethiopia.
**treatment_effect_clean_birth**	The effect of a neonate's mother receiving clean birth practices on neonatal risk of developing EONS during the neonatal period	0.73	Sourced directly from Blencowe et al. (61) in which the authors estimate the effect of clean birth and postnatal care practices on neonatal deaths from sepsis and tetanus via Delphi method. For the purposes of the model, the same effect on reducing the risk of sepsis is assumed.

**treatment_effect_early_init_bf**	The effect of early initiation of breastfeeding on neonatal risk of developing sepsis during the neonatal period	0.55	Sourced directly from Debes et al. (359) who estimate the effect of early initiation of breastfeeding on the risk of neonatal mortality secondary to sepsis via meta-analysis of observational studies. As the mechanism of action through which breastfeeding reduces infection mortality is through reduction in incidence of infection (365,366) this treatment effect is applied to risk of sepsis acquisition.
**treatment_effect_abx_prom**	The effect of a neonate's mother receiving antibiotic prophylaxis following PROM on neonatal risk of developing EONS during the neonatal period	0.67	Sourced direct from Kenyon et al. (59) who reports the effect of antibiotic treatment for PROM on risk of neonatal infection as derived from a Cochrane review of relevant RCTs as RR 0.67 (95% CI 0.52 to 0.85).
**prob_early_onset_neonatal_sepsis_week_1**	The probability that a neonate will develop EONS during the first week of life	0.02 / 0.017	See **prob_early_onset_neonatal_sepsis_day_0.**
**treatment_effect_inj_abx_sep**	The effect of injectable antibiotic treatment on the risk of neonatal death due to sepsis	0.35	Sourced directly from Zaidi et al. (73) who estimate the effect of antibiotic treatment on neonatal sepsis mortality for the LiST using a Delphi survey of relevant experts. They report the intervention is 65% effective in reducing deaths.
**treatment_effect_supp_care_sep**	The effect of full supportive care on the risk of neonatal death due to sepsis	0.2	See **treatment_effect_supp_care_sep.** Zaidi et al. (73) report the intervention is 80% effective in reducing deaths.
**cfr_early_onset_neonatal_sepsis**	The probability that a neonate will die following EONS without treatment	0.064 / 0.056	See **cfr_respiratory_distress_syndrome** in Table S54.
**prob_late_onset_neonatal_sepsis**	The probability that a neonate will experience LONS per week of the neonatal period	0.0045 / 0.0038	See **prob_early_onset_neonatal_sepsis_day_0.**

cfr_late_neonatal_sepsis	The probability that a neonate will die following LONS without treatment	0.064 / 0.056	See **cfr_respiratory_distress_syndrome** in Table S54.

** Where two values (or sets of values) are provided the first set is applied from 2010-2014 and the second set from 2015 onwards for a given simulation run (§1.2.1.1)*

Table S59 – Parameters of the neonatal sepsis model

3.2.4 Congenital birth anomalies

3.2.4.1 Condition overview

Congenital birth anomalies (CBA) can be defined as “structural or functional abnormalities, including metabolic disorders, which are present at birth” (362). CBA represent a varied group of conditions which, by definition, are prenatal in origin and are most-often associated with single-gene defects and chromosomal disorders (362–364). In addition, environmental factors can drive onset of CBA including maternal exposure to environmental teratogens (e.g., certain recreational drugs) or significant malnutrition leading to micronutrient deficiency (362–365). Importantly, in most observed cases, it is not possible to identify a clear aetiology (363,364). For the purposes of surveillance, CBA are often categorised by the primary organ, organ system or structure which is predominantly affected by the condition. Within the GBD study this leads to ten distinct groupings[7] with the three most commonly occurring CBA groupings globally in 2019 being congenital heart anomalies, congenital limb or musculoskeletal anomalies and urogenital congenital anomalies (366).

CBA are associated with significant morbidity and mortality in children globally. Children with CBA are found to be at greater risk of mortality at all ages during childhood when compared to children without CBA and are significantly more likely to experience death related to circulatory, respiratory, or digestive causes (367). Within Europe as much as 17-42% of all infant mortality can be attributed to CBA, leading to an average rate of infant mortality due to CBA of 1.1 per 1000 births (368), with global estimates of child mortality by cause suggesting CBA remains a leading contributor to neonatal and childhood death (369). In addition to risk of mortality, child disability is associated with CBA (370), especially in settings where access to surgical care is limited (371,372).

Within SSA the prevalence of congenital birth anomalies has been estimated in a systematic review of studies within the region leading to a pooled prevalence of 20.40 (95% CI: 17.04, 23.77) cases per 1,000 births (373). The authors reported inter-regional variation, with the

[7] Congenital heart anomalies, Congenital musculoskeletal and limb anomalies, Urogenital congenital anomalies, Other chromosomal abnormalities, Digestive congenital anomalies, Neural tube defects, Orofacial clefts, Down syndrome, Turner syndrome, Klinefelter syndrome

highest prevalence detected in southern Africa where 43 (95% CI: 14.89, 71.10) cases of CBA were present per 1000 live births (373). The authors propose that variation could be due to variation in exposure of mothers to environmental teratogens (373) but could also be associated with variation in quality of care with more pregnancies continuing to live births in some settings leading to a greater number of live born infants with birth anomalies. As discussed further in Table S59, the rate of CBA in the model is sourced from Adane et al. (373) due to a lack of publicly available population-level estimates of CBA prevalence in Malawi[8].

3.2.4.2 Model

Figure S32 provides an overview of the model of congenital birth anomalies used within this module whilst Table S59 contains the model parameters.

---

8 Birth-defect surveillance in Malawi is currently being undertaken by The International Training and Education Center for Health (I-TECH) at several high-volume birth sites across the country (374). However, data is not yet publicly available. Future iterations of the model could incorporate this data to improve accuracy of estimates.

.

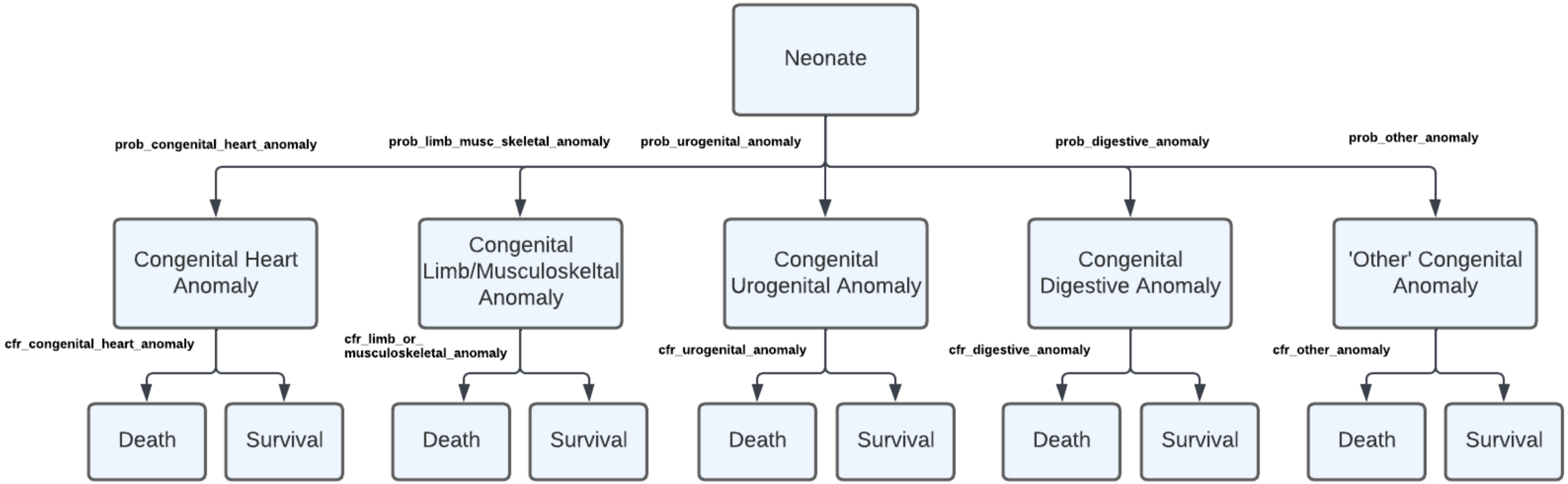

Figure S32- Model of congenital birth anomalies
Diagrammatic representation of the model of congenital birth anomalies. Light blue represents the model's natural history pathways. Parameters representing progression through model states are shown here.

As shown in Figure S32 five ‘types’ or ‘groupings’ of CBA are modelled which have been adapted from the GBD study categorisation. We opted to include the top four highest incidence CBA groupings for Malawi as reported in the GBD study and grouped all remaining types of CBA as ‘other’.

It is assumed that any neonate who survives until birth has a risk of being born with a congenital anomaly and that non-survivable anomalies are implicitly modelled in rates of spontaneous abortion and stillbirth. Risk of CBA is therefore applied on birth with via the parameters described in Table S59. To allow for co-existence of multiple congenital anomalies in the same individual risk is applied sequentially.

CBA-specific case fatality parameters have been calculated from the estimated number of deaths attributed to each anomaly and the number of cases within the GBD (Table S59). Risk of death is applied after birth and for individuals in which the primary cause of death is a congenital anomaly the function enacting death in the simulation is scheduled to occur randomly within an age range selected for the individual based on the distribution of deaths by age. These age groups taken from the GBD study include early neonatal (days 0-6), late neonatal (days 6-28), post neonatal (day 29- 1 years), 1-4 years, 5-9 years, 10-14 years, and 15-69 years.

Whilst mortality is captured, any disability associated with these conditions are not included in this version of the MPHM. This is largely due to the complexity in accurately portraying the associated disability attributable to this very diverse range of conditions. Therefore, it was deemed this was outside the scope and focus of this current work and should be notified as a limitation that will be rectified in future versions. Similarly, treatment for any of the CBA groupings is not modelled and will be included in future iterations.

3.2.4.3 Data sources

Parameter Name	Description	Value*	Source and/or relevant calculation
prob_congenital_heart_anomaly	The probability that a neonate has been born with a congenital heart anomaly	0.009	The total rate of CBA for the model was sourced from Adane et al (373) as 20.40 per 1,000 births (95% CI: 17.04, 23.77). The specific rate for each CBA grouping was calculated from the total rate by multiplying this rate by the proportion of total CBA cases due to each of the five groupings using the GBD estimates of CBA in Malawi (1).
prob_limb_musc_skeletal_anomaly	The probability that a neonate has been born with a congenital limb or musculoskeletal anomaly	0.008	See **prob_congenital_heart_anomaly**.
prob_urogenital_anomaly	The probability that a neonate has been born with a congenital urogenital anomaly	0.003	See **prob_congenital_heart_anomaly**.
prob_digestive_anomaly	The probability that a neonate has been born with a congenital digestive tract anomaly	0.0007	See **prob_congenital_heart_anomaly**.
prob_other_anomaly	The probability that a neonate has been born with a congenital anomaly other than those already defined in the model	0.0004	See **prob_congenital_heart_anomaly**.
cfr_congenital_heart_anomaly	The probability that an individual with a congenital heart anomaly will die due to their condition during their lifetime	0.062	Lifetime probability of death for each of the CBA cases was calculated by dividing the total deaths due to each of the CBA grouping by the total cases of each CBA in Malawi using data from the 2019 GBD study (1).

**cfr_limb_or_musculoskeletal_anomaly**	The probability that a neonate with a congenital limb or musculoskeletal anomaly will die due to their condition	0.0069	See **cfr_congenital_heart_anomaly**
**cfr_urogenital_anomaly**	The probability that a neonate with a congenital urogenital anomaly will die due to their condition	0.0085	See **cfr_congenital_heart_anomaly**
**cfr_digestive_anomaly**	The probability that a neonate with a congenital digestive tract anomaly will die due to their condition	0.2829	See **cfr_congenital_heart_anomaly**
**cfr_other_anomaly**	The probability that a neonate with a congenital anomaly other than those already defined in the model will die due to their condition	0.131	See **cfr_congenital_heart_anomaly**
**prob_cba_death_by_age_group**	The probabilities that a neonate who will die due to their congenital anomaly will die within the early neonatal period (day 0-6), the late neonatal period (day 7-28), post neonatal (day 29- 1 years), years 1-4, years 5-9, years 10-14 or years 15-69	[0.27, 0.08, 0.38, 0.2, 0.02, 0.01, 0.04]<br><br>/<br><br>[0.3, 0.09, 0.35, 0.18, 0.03, 0.02, 0.03]	These values were calculated using the GBD study data which reports both incidence and deaths due to CBA in Malawi (1). For simplicity the total deaths due to CBA (not by type) were broken down by age group allowing for the calculation of the proportion of total deaths by cause for each of the groupings outlined in the description.<br><br>A limitation of this approach Is that other causes may lead to an individual's death in the model before their scheduled death due to CBA meaning the probabilities will not exactly lead to the correct distribution. This was deemed appropriate for the current model iteration.

Table S60 – Parameters of the congenital birth anomaly model

4 – Model Verification and Validation

This section outlines the methods that have been undertaken to ensure the validity of the estimates produced by the MPHM through the processes of verification and validation. Model verification is defined here as the process of "ensuring that the computer program of the computerized model and its implementation are correct" (375) whilst model validation process is complementary to but distinct from verification and can be defined as "substantiation that a model within its domain of applicability possesses a satisfactory range of accuracy consistent with the intended application of the model" (375).

4.1 Verification Methods

The process of code verification, from a software development perspective, can be undertaken via static and dynamic testing methods. Static methods entail the visual evaluation of code and code debugging without running the program itself, while dynamic testing methods involve the execution of the code (376). Both static and dynamic testing processes were conducted in parallel to the development of the model code with the dynamic methods discussed below.

Unit and integration testing

To verify the code that was written for each file constituting the MPHM, both unit and integration testing was undertaken during the development process. Within software development, unit testing is the process by which developers build "tests of individual programs or modules in order to ensure that there are no analysis or programming errors" (376). To achieve this, unique Python test files were developed for each of the associated Python scripts which constitute the MPHM. These test files verify the implementation of the code base using Python's inbuilt *Pytest* framework, a Python framework with features enabling the development of various types of verification testing (377).

In principle, each test file houses a series of functions which test the assumed logic of some subset of the code, usually an isolated function or event class. Within a given test this involves replication of simulation logic with varied parameter values to ensure agent behaviour occurs as expected. In this way the most important logic within a module can be

isolated and tested under a series of parameter assumptions, e.g., that varying treatment effectiveness parameters affects probability of survival. Commonly this involves 'boundary testing' in which parameters are replaced by either their minimum or maximum value and the behaviour of the model is evaluated to ensure the code logic brings about expected outcomes.

In addition, but not discussed here, similar test files exist for all implemented modules (or other key classes) within the TLO framework which are all executed whenever additional changes are merged into the model itself. This approach to verification is limited in that every possible permutation cannot be tested for, however focused testing on the core functions of the modules should increase confidence that the implementation of the code leads to the expected outcomes.

In addition to unit testing, integration testing, which involves 'progressive linking and testing of programs or modules in order to ensure their proper functioning in the complete system' (376) was regularly conducted. At the most basic level this entails running a simulation in which all currently developed modules are registered for several years and asserting that no error codes have been logged during the simulation run. Testing, and other aspects of code base verification was supported by authors who are research software development experts based at UCL to ensure quality and consistency with TLO model conventions.

In-built error messaging

To support the process of unit testing, a system of error-logging where module level error codes are logged if predetermined conditions are met that signified agent, or model, behaviour had deviated from the expected logical function of the model itself was implemented. For example, a series of conditional statements are used to evaluate the variables of all individuals who have moved to the event which signifies the beginning of a pregnant individual's labour and an error code is logged and detected by the test file if any of the conditions are not met (e.g. the individual is not currently pregnant).

4.2 Validation Methods

4.2.1 Face validation

Collaboration between modellers and other subject matter experts to improve the validity of model is a practice utilised in many disciplines including healthcare modelling (378). Face validation is defined as the process in which individuals with expert domain knowledge related to the system of interest are asked to determine if the model and/or its behaviour are reasonable given their expertise (375). Important structural areas of the model which should be reviewed include the overall level of detail, model logic and representation of any relationships, data sources and outcomes (378). Klügl (379) suggests that the process of face validation demonstrates that both model behaviours and outcomes are reasonable as they relate to the "theoretic basis and implicit knowledge" of the subject matter experts. They contrast this with empirical validity (e.g. calibration) in which outputs from the model are compared, sometimes statistically, to data from the reference system (379) as discussed below.

4.2.1.1 Approach taken for this model

Face validation of the MPHM was largely undertaken alongside the processes of model development. Subject matter experts were those deemed to have significant clinical experience relative to the setting of Malawi, the health area of interest (i.e., clinical obstetrics or neonatology) or both and were identified either from their direct association with the wider TLO project or through the networks of other project members. Across the project length a total of five clinicians assisted with this process include two obstetricians, two neonatologists and an infectious disease specialist practising across the UK, Malawi, and Uganda. It was deemed most appropriate to gain clinical expert review prior to or alongside development of the computational framework in Python meaning that suggested changes could be incorporated in real time.

Experts were all provided with module documentation, similar in structure and content to sections 1-3 in this document. Documentation included a descriptive overview, model variables and parameters, diagrammatic representation of the model and sub-models (including all modelled diseases) and any key assumptions for review. The experts who

assisted with this process include the authors HA, AR, CT, and KK. In addition, the MPHM model was presented to stakeholders within the Malawian MoH on 11/09/2020 and 02/10/2020 to elicit feedback from those within the Ministry who will be utilising the model over the coming years. Model structure, data sources and key assumptions were presented and further validated by attendees including the Head of the Reproductive Health Directorate, author FK.

4.2.2 Model calibration methods

4.2.2.1 Common approaches to calibrating IBMs

Calibration, or operational validation (375), is a central part of the model validation process in which the model is fit to data to improve confidence in the model's predictions (378,380). The process of model calibration involves comparing results generated by the model with outcome distributions that have been estimated by an alternative analytical method and may be observational or experimental (381). Calibration of IBMs is often achieved through a number of processes, including the selection of summary statistics from the empirical data to calibrate to (i.e., targets), a parameter search strategy which explores the parameter space, utilisation of a measure of goodness-of-fit (GOF) to quantify model fit to calibration targets and definition of explicit acceptance criteria and stopping rules to signify when calibration is complete (378,380). McCulloch et al. (382) further categorises the calibration of IBMs as either point estimation or 'categorical or distributional estimation'. Point estimation calibration, the method used here, involves identifying a single set of parameter values which produces the best calibration to target data (382). Using this approach, variation in model output across runs is due to the stochastic variation introduced using random number generators as opposed to parametric uncertainty. Alternatively, categorical, or distributional calibration assigns probabilities to multiple parameter sets over a range of possible values (382).

In the next section the calibration procedures undertaken for the MPHM including how calibration targets were identified and the rationale for these choices, the approach to parameter searches and assessment of GOF are presented. Following this, output graphs demonstrating the model's calibration to targets are shown.

4.2.2.2 Maternal and perinatal health model calibration

4.2.2.2.1 Selection of empirical calibration targets

The selection of calibration targets from available sources was undertaken to ensure that the model would be able to reliably evaluate the effect of maternity services on a breadth of key outcomes. As such the primary calibration 'areas of focus' included maternal and perinatal mortality and morbidity and health service coverage. In line with the wider TLO framework, the MPHM was calibrated to data from 2010 to 2022. During evaluation of data sources available for each outcome it was evident that there was a paucity of reliable time-series data for many modelled outcomes. One of the key potential sources of data for the model in Malawi was the District Health Information System (DHIS2) which is the primary health information management system in Malawi (383). The DHIS2 captures a substantial amount of data relating to maternal and perinatal health outcomes and service use, however there are significant issues with data-completeness and incomplete reporting across several regions within the country (60,383) meaning that at the time of model development this data was not appropriate to use for calibration.

Because of the lack of reliable time series data in the DHIS2, and following review of the available nationally representative data available in Malawi, it was deemed appropriate to calibrate the model to data sourced from two years within the 2010-2022 period. These years, 2010 and 2015, were largely selected because they are the data collection years of the two most recent Demographic and Health Surveys and Emergency Obstetric and Newborn Care needs assessment surveys in Malawi – see Table S60. These four surveys are nationally representative population and health system surveys containing a wealth of information for calibration of the model and were deemed the most appropriate calibration data for most of the model. To achieve calibration to the data in 2010 and 2015 two parameter sets were used with the first applied from 2010-2014 and the second applied from 2015 onwards.

**Calibration outcome**<br>*Definition*	**Data sources**	**Data source study design**	**Notes on methodology**
**Direct MMR**<br>*Number of direct maternal deaths per 100,000 live births per year*	The Malawian DHS Surveys for 2010 and 2015 (11,12)	Population survey	The DHS is a population survey conducted in Malawi which collects a range of sociodemographic and health data including data related to maternal and newborn health and healthcare use in the region. The methodology of these surveys allows for presentation of key indicators for the country, for urban and rural populations and for each of Malawi's 28 districts.<br><br>The 2010 and 2015 DHS employ a multi-stage stratified design utilising the sampling frame for the 2008 Malawian census. Districts were stratified into urban and rural areas leading to 56 sampling strata from which standard enumeration areas (SEAs), geographic areas containing an average of 235 households, were independently selected in a two-stage process leading to selection of households for survey administration. Due to the population-level sampling approach these surveys were selected for model calibration as other estimates of maternal death in Malawi are generated via modelling as opposed to direct measurement (1,384).<br><br>The number of maternal deaths In the seven years prior to the survey Is captured via the 'sisterhood method'. Female respondents provide a list of siblings, identifying if any are alive at the time of the survey, along with current age. For any sisters who have died at age 12 or older, questions are administered to determine if the death was maternal. Deaths from accidental causes and violence were excluded.
**Maternal DALYs due to 'Maternal Disorders'†**<br>*Number of DALYs per year which are solely attributable to 'Maternal Disorders' according to GBD criteria.*	The Global Burden of Disease Study (2019) (1)	Modelled estimate	The GBD study group produces modelled annual estimates for the number of DALYs attributable to "causes" within their framework for each country. The estimation process within the GBD utilises any available relevant country data sources which are processed and modelled using a set of three

			standardised tools: Cause of Death Ensemble model (CODEm), spatiotemporal Gaussian process regression (ST-GPR), and DisMod-MR (1).<br><br>The constituent components of DALYs, YLD and Years of Life Lost (YLL) are estimated for each cause. YLL due to premature mortality are calculated by first determining the lowest observed age-specific mortality rates by location to establish a minimum risk reference life table. YLL is then calculated by multiplying the number of estimated deaths from a given cause by standard life expectancy at age of death (1). YLDs for a given health outcome within a population are computed by multiplying those with that condition by a disability weight which represents health loss associated with that outcome.
**Maternal deaths due to PPH**<br>*Proportion of maternal deaths per year due solely to PPH*	Malawi 2010 EmONC Needs assessment and Malawi Emergency Obstetric and Newborn Care Needs Assessment (2014) (33,34)	National survey of facilities	The 2010 and 2014 EmONC needs assessments were national facility-based cross-sectional surveys which evaluated the provision of emergency obstetric care in Malawi. The 2010 survey was administered at all hospitals and 50% of health centres which conducted deliveries. Health centres were selected randomly from a list of facilities providing relevant services provided by the MoH to ensure appropriate representation across districts. The 2014 survey was also administered in all hospitals, but the sample of health centres was increased to 60% of those operating in Malawi. Due to the representativeness of these surveys they were selected for calibration.<br><br>Facility-based maternal deaths are recorded during a period of observation during survey conduct. Both surveys report the percentage of direct deaths attributable to leading causes of maternal death in the sample.
**Maternal deaths due to sepsis**<br>*Proportion of maternal deaths per year due solely to maternal sepsis*			
**Maternal deaths due to severe pre-eclampsia/eclampsia**<br>*Proportion of maternal deaths per year due solely to maternal severe pre-eclampsia/eclampsia*			
**Total stillbirth rate**<br>*Number of stillbirths occurring per 1000 births per year*	The UN Inter-agency Group for Child Mortality Estimation (UN IGME)	Modelled estimate	The UN IGME estimates of stillbirth in Malawi are model based. First, all relevant data sources which may record stillbirths from the country are compiled (i.e., registration systems, population surveys etc) alongside data

**Antenatal stillbirth rate**<br>*Number of stillbirths occurring prior to the onset of labour per 1000 births per year*<br><br>**Intrapartum stillbirth rate**<br>*Number of stillbirths occurring following the onset of labour per 1000 births per year*	stillbirth rate estimates for Malawi (299)		describing factors related to stillbirth. These factors are used as covariates in the stillbirth prediction model.<br><br>A Bayesian hierarchical temporal sparse regression model (BHTSRM) Is used to both estimate SBR and address data challenges. This method combines the identified covariates with a process of temporal smoothing, leading to stillbirth estimates which are data driven for country-periods where data are available and derived from covariates for country-periods for which data is unavailable (385).
**NMR**<br>*Number of neonatal deaths per 1000 live births per year*	The Malawian DHS Surveys (11,12)	Population survey	See **Direct MMR**. Neonatal deaths are captured through respective birth history provided by sampled mothers who list all children they have borne, death of these births, survivorship status, age, or age at death.
**Neonatal DALYs due to 'Neonatal Disorders'**<br>*Number of DALYs per year which are solely attributable to 'Neonatal Disorders' according to GBD criteria.*	The Global Burden of Disease Study (2019) (1)	Modelled estimate	See **Maternal DALYs due to 'Maternal Disorders'**
**Neonatal deaths due to prematurity**<br>*Proportion of neonatal deaths per year due solely to complications associated with prematurity.*	Cause-specific neonatal mortality: analysis of 3772 neonatal deaths in Nepal, Bangladesh, Malawi and India (331)	Multi-district study<br><br>/	Fottrell et al. (331) conducted a prospective study within two surveillance sites in Malawi which were previously established as part of a Randomised Control Trial (RCT) evaluating community mobilisation and women's groups. The MaiMwana trial was based in a rural setting in Mchinji district and the MaiKhanda trial covered three districts in the central region of the country –

**Neonatal deaths due to sepsis**<br>*Proportion of neonatal deaths per year due solely to early onset sepsis*	Malawi Emergency Obstetric and Newborn Care Needs Assessment (2014) (33)	National survey of facilities	Kasungu, Lilongwe and Salima. Data for MaiMwana was collected between June 2004 and January 2011 and data for MaiKhanda between June 2007 and December 2010. The number of neonatal deaths was captured for each survey surveillance site with cause of death ascertained via Verbal Autopsy. Cause specific neonatal mortality fractions were calculated for relevant ICD classifications including prematurity, neonatal sepsis, and birth asphyxia.<br><br>For the EmONC needs assessments see **Maternal deaths due to PPH** for notes on survey methodology. Within the 2015 EmONC needs assessments, 408 neonatal deaths for 174 facilities over a 12-month observation period were reviewed. Cause of death was reported for these deaths as a crude number and as proportion of total neonatal deaths observed.
**Neonatal deaths due to intrapartum related events**<br>*Proportion of neonatal deaths per year due solely to intrapartum related events – previously 'birth asphyxia'*	*(Model outputs were compared to both estimates of cause specific mortality for neonates)*		
**Proportion of women attending any ANC**<br>*Proportion of women who have had a delivery that year that have attended at least one or more ANC visit during pregnancy.*	The Malawian DHS Surveys (11,12)	Population survey	See **Direct MMR** for a description of general methods and sampling of the Malawian DHS.
**Proportion of women attending four or more ANC visits**<br>*Proportion of women who have had a delivery that year that have attended at least four or more ANC visits during pregnancy.*			
**Gestational age at first ANC visit**<br>*The proportion of total ANC1 visits by maternal GA at the time of visit.*			

**Total ANC visits**<br>*The total number of visits undertaken by women who delivered and attended any ANC visits per year.*			
**Proportion of births occurring in a health facility**<br>*Proportion of total deliveries per year which occur in a health facility (any level)*			
**Proportion of births occurring in a hospital**<br>*Proportion of total deliveries per year which occur in a hospital.*			
**Proportion of births occurring in a health centre**<br>*Proportion of total deliveries per year which occur in a health centre.*			
**Proportion of births occurring at home**<br>*Proportion of total deliveries per year which occur in the home of the mother.*			
**Caesarean delivery rate**<br>*Proportion of total deliveries per year which occur via CS.*			

**Proportion of women attending PNC**<br>*Proportion of women who have had a delivery that year who receive any postnatal care.*			
**Proportion of neonates receiving PNC**<br>*Proportion of neonates born that year who receive any postnatal care*.			

Table S61- Outcomes and the data sources used to calibrate the MPHM

4.2.2.2.2 Parameter search strategy and calibration using visual GOF

Algorithmic parameter search strategies were not used within the calibration of this model. Because of the breadth of the primary research question and ensuing complexity of the model it was deemed necessary to ensure the model was calibrated to a significant number of outcomes (Table S60). As such, there were a considerable number of parameters that have influential effect on the overall rate of these outcomes within the model including complication incidence, care seeking, quality parameters and treatment effectiveness. Algorithmic exploration of the parameter space for so many key parameters meant that algorithmic methods would likely not have been appropriate calibration of the MPHM.

It was deemed most appropriate and practical to hand-manipulate relevant parameters and assess GOF visually by plotting model outcomes against calibration target data points as discussed below. Across the MPHM there are a total of 452[9] parameter values. Once the model was conceptualised and parameters in the model were identified, the literature was searched for data sources to inform the values from Malawi. Currently, uncertainty around parameter estimates is not incorporated into the model. This means that as opposed to any parameters being drawn from a probability distribution within a given model run, instead the same fixed parameter set of point estimates is used for each model run.

Broadly, parameters in the model are either taken directly from a relevant data source and inputted into the model (e.g. the effect of treatment on the risk of a given outcome such as the parameter **treatment_effect_iron_folic_acid_anaemia** in Table S41) or have been derived through calibration to one of the data sources presented in Table S60. For example, parameter **cfr_pp_pph,** which represents the probability of death associated with postpartum haemorrhage in the absence of treatment, is derived from calibration of the model to the percentage of total maternal deaths due to PPH given the availability of treatment.

The calibration outcomes of Interest were ordered according to the logical relationship between outcomes. For example, outcomes related to coverage of health services would

[9] This does include parameters which share the same function but are housed within different python files

require initial calibration as the delivery of interventions within these services would have a direct effect on complication incidence, outcome and in turn mortality. Following this, the incidence of all death and disability-causing complications would need to be calibrated in the model and finally the mortality and DALY outcomes. Practically, this entailed running the model on a population of at least 50,000 individuals and generating plots of the outcome per year during this period with GOF to target datapoints assessed visually. Where there was a direct relationship between the parameter and an outcome of interest, parameter values were manually adjusted by multiplying the value by the quotient of the module output and the calibration target following which the model was rerun to check the fit.

Otherwise, where the relationship between a key parameter and an outcome was less direct (e.g. cause-specific case fatality parameters and total mortality) a potential pathway from the parameter to the outcome was constructed using Microsoft excel.

4.2.3 Additional model calibration results

In the following sections, plots are presented which further demonstrate the model's calibration to the outcomes listed in Table S60. Calibration plots have been generated from a model run with a population of 250,000 individuals simulated from 2010 to 2030 for 20 runs with each run having a different fixed seed. Where model data is presented as a line graph over time, the blue line is the mean value across these runs for the outcome of interest, whilst the shaded area represents the 95% confidence intervals (95% CI) quantifying the stochastic variation across the 20 runs. All plots are presented with data from 2011 onwards to allow for the model to stabilise in 2010. As outlined in the manuscript 2010 acts as a burn-in period for the model as women may only become pregnant once the simulation starts and no women begin the simulation pregnant.

Figure 1 within the accompanying manuscript shows the calibration plots for the total MMR, direct maternal deaths by cause, NMR, neonatal deaths by cause and SBR. Additional plots are presented here

4.2.3.1 Maternal mortality and DALYs

4.2.3.1.1 Direct maternal mortality ratio

Figure S33 shows the MMR driven by direct deaths only between 2010 and 2022 compared to DHS calibration targets. Additional data presented within the figure allows for comparison to other prominent estimates of maternal mortality in Malawi including the GBD (1) and the WHO Maternal Mortality Estimation Interagency Group (386). Estimates presented by both these groups are derived through varied advanced statistical modelling utilising available data sources from Malawi. These data points have been adjusted to approximate the MMR associated with direct causes of maternal death by reducing the value taken from the DHS surveys by 30%, which is the percentage of observed maternal deaths attributed to indirect causes in the most recent Malawi BEmONC needs assessment (33).

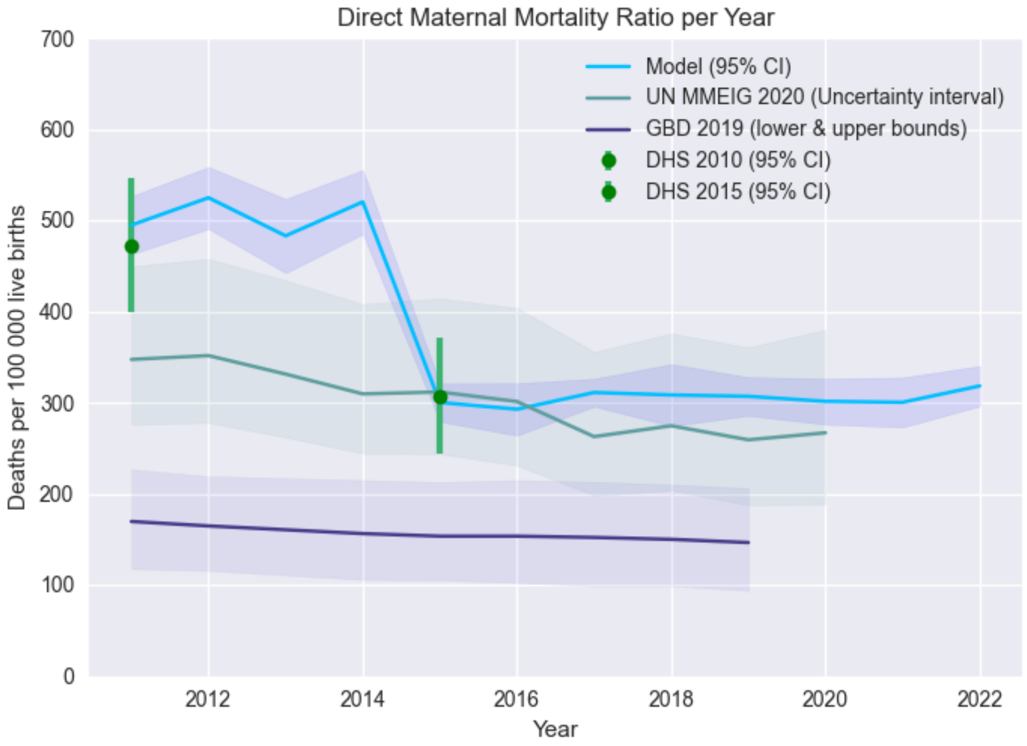

Figure S33 – Model output of the direct MMR per year
The mean MMR (95% CI) across 20 simulation runs per year outputted from the model (shown in blue) is plotted against estimates of MMR produced by the Global Burden of Disease (GBD) group (purple), the UN Maternal Mortality Estimation Inter-Agency Group (grey) and the Malawi Demographic and Health Survey estimates from 2010 and 2015 (green).

4.2.3.1.2 Maternal DALYs

Figure S34 shows the number of DALYs due to ‘Maternal Disorders’ generated by the model each year.

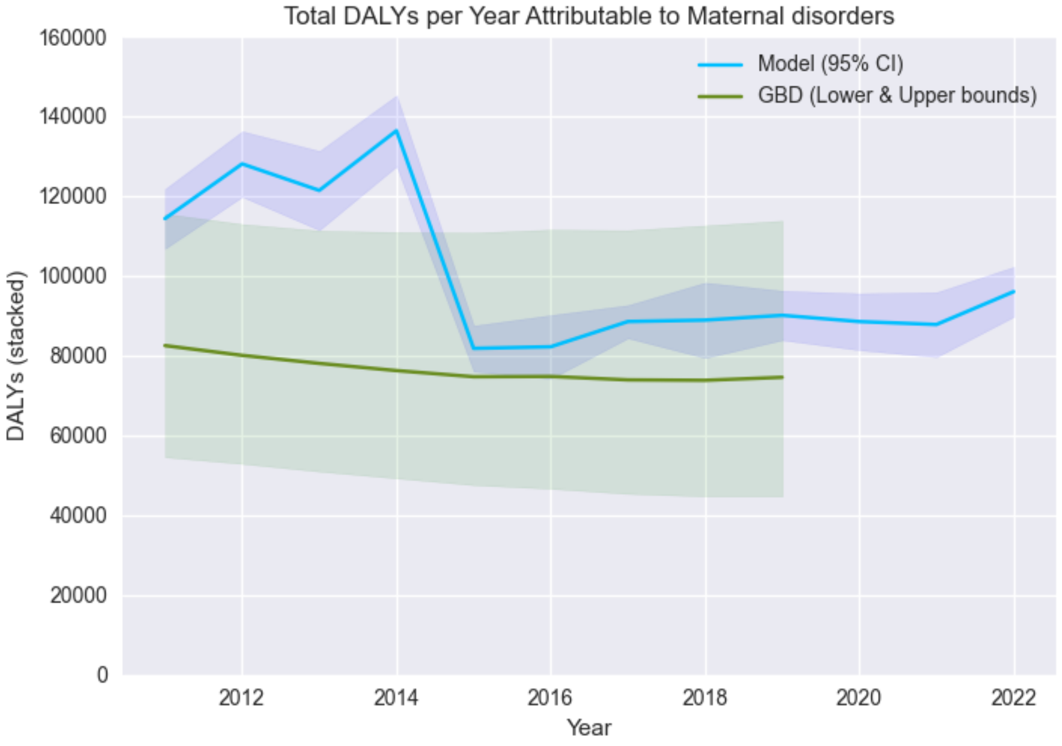

Figure S34 – Model output of total DALYs per year attributable to maternal disorders
The mean DALYs (stacked) (95% CI) across 20 simulation runs per year outputted from the model (shown in blue) is plotted against estimates of DALYS attributable to “Maternal Disorders” produced by the Global Burden of Disease (GBD) group (green).

Within this figure DALYs generated by the model are attributable to direct obstetric causes. The GBD categorisation of ‘Maternal Disorders’ is inclusive of both DALYs due to direct obstetric causes and DALYs generated by ‘indirect maternal deaths’ and ‘deaths aggravated by HIV/AIDS’ which are not included in model outputs. DALYs outputted by the model in this figure are stacked, meaning that all the life-years lost up to an individual’s predicted life expectancy are ascribed to the year of death, which is the same approach used by GBD study. The model fits well to GBD estimates of maternal DALYs per year from 2015 although there is divergence between the estimates earlier in the calibration period. This is as expected because maternal mortality in the model is calibrated to DHS datapoints which report an overall much higher MMR in that time than the GBD estimates (Figure S33).

4.2.3.2 Antepartum and intrapartum stillbirth

Figure S35 shows the yearly rate of antenatal and intrapartum stillbirths per 1000 births outputted by the model during the calibration period.

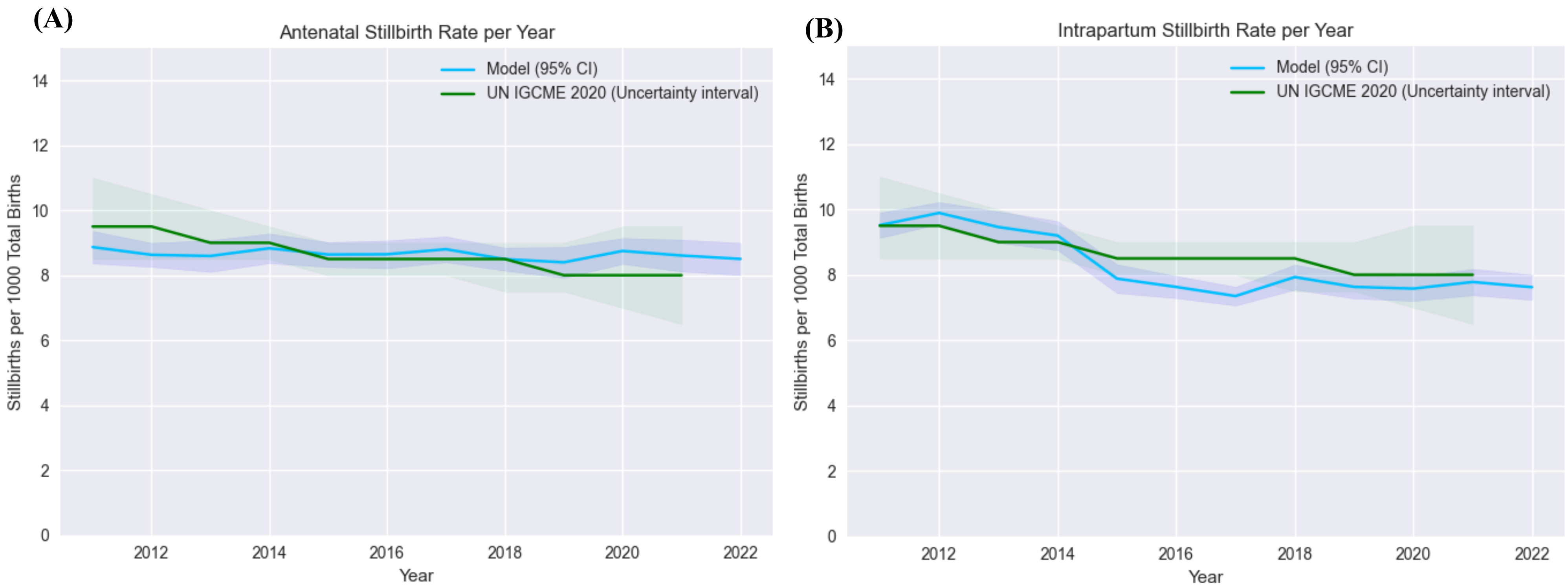

Figure S35 – Model output of the antenatal and intrapartum SBR per year
(A) The mean antenatal SBR (95% CI) across 20 simulation runs per year (blue) is plotted against estimates of SBR produced by the UN IGCME (green). Model calibration is discussed further in the supporting information.) (B) The mean intrapartum SBR (95% CI) across 20 simulation runs per year (blue) is plotted against estimates of SBR produced by the UN IGCME (green).

Disaggregation of SBR by these time periods is common practice in perinatal epidemiology due to variation in the aetiology of pregnancy loss prior to or during labour and delivery (296, 297). Therefore, it was deemed important to ensure that the burden of stillbirth in the model was accurately spread across the intrapartum and antenatal time periods as reflective of Malawi. It was assumed that approximately half (49%) of stillbirths would occur antenatally and the remainder during delivery in keeping with estimates for SSA (281). The UN IGCME data was selected for calibration as these estimates incorporate several historic population level estimates of stillbirth rates within Malawi into their estimation model as discussed in Table S60.

4.2.3.3 Neonatal Mortality and DALYs

Figure 1 within the accompanying manuscript shows the calibration plots for the total NMR and neonatal deaths by leading cause.

4.2.3.3.1 Neonatal DALYs

Figure S36 shows the number of DALYs attributable to 'Neonatal Disorders' per year between 2010 and 2022.

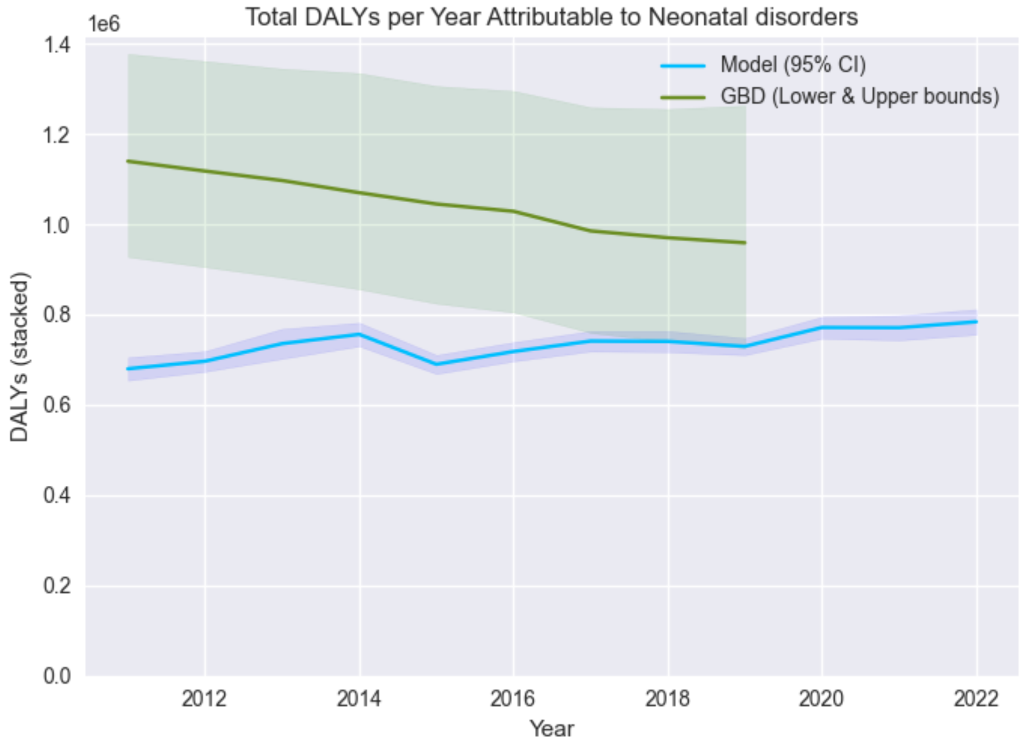

Figure S36 – Model output of total DALYs per year attributable to 'neonatal disorders'
The mean DALYs (stacked) (95% CI) across 20 simulation runs per year outputted from the model (shown in blue) is plotted against estimates of DALYS attributable to "Neonatal Disorders" produced by the Global Burden of Disease (GBD) group (green).

Similarly to maternal DALYs, the number of DALYs generated per year for the relevant conditions are stacked. Figure S36 shows convergence between model output and GBD values over time with overlapping confidence and uncertainty intervals towards the end of the calibration period. Observed difference between GBD estimates and model outputs is due to fewer YLL and YLD generated by the model. There is divergence between the number of deaths generated by the model attributed to 'Neonatal Disorders' and those estimated by the GBD meaning fewer YLL associated with neonatal disorders are being generated (this is evident from Figure 3 in the main paper). This is likely due to the additional 'Neonatal Disorders' conditions which are not modelled in the MPHM. Additionally, as shown in Figure S37 the model appears to generate fewer YLD per year than the GBD estimates. This could also be attributed to unmodelled conditions and additionally that incidence of neonatal conditions in the MPHM do not necessarily match estimates in the GBD which may predict high rates of morbidity driving conditions in this period.

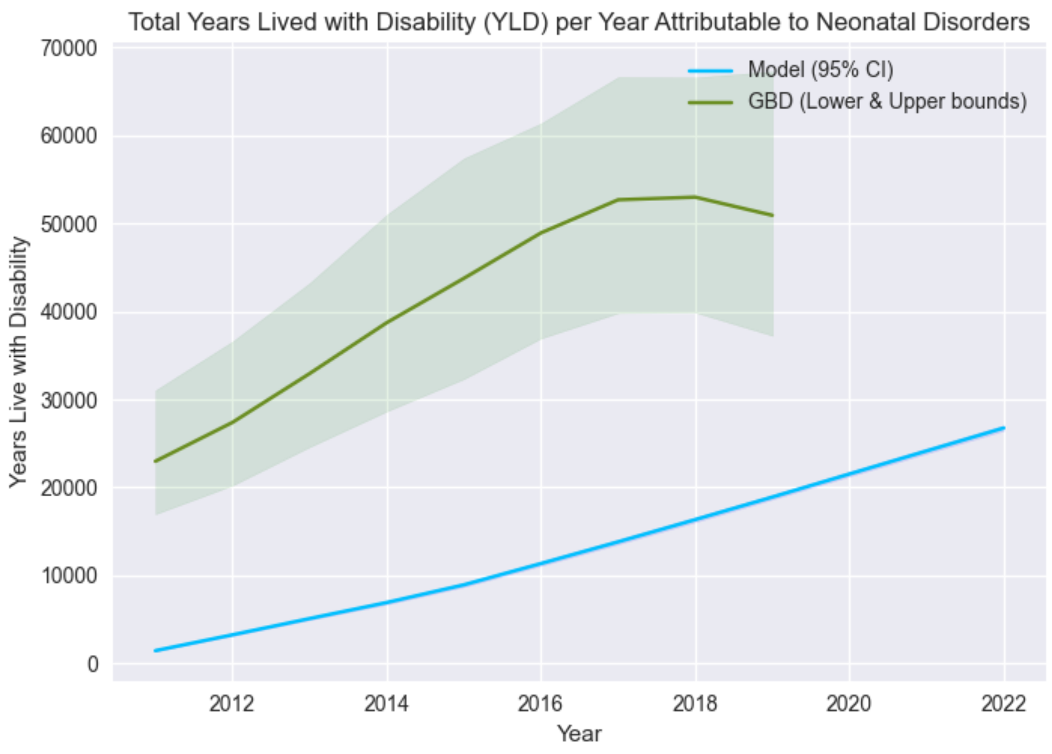

Figure S37 -Model output of the Years Lived with Disability generated by 'Neonatal Disorders' per year compared to calibration data
The mean YLD (95% CI) across 20 simulation runs per year outputted from the model (shown in blue) is plotted against estimates of YLD attributable to "Neonatal Disorders" produced by the Global Burden of Disease (GBD) group (green).

4.2.3.4 Antenatal care coverage

Figure S38 shows the percentage of women who gave birth in 2010 and 2015 who received one or more antenatal care visits during their last pregnancy and Figure S39 shows the percentage of women who attended four or more visits during their last pregnancy. As outlined in Table S60, the model has been calibrated to estimates from the most recent DHS which report that whilst coverage of at least one visit has historically been high in Malawi significantly less women receive four or more visits during pregnancy (ANC4+). Ensuring the model outputs the correct coverage of ANC4+ within the population was deemed crucial due to the importance of ANC4+ coverage as an indicator of effective maternal health services. The continued importance of ANC4+ in contemporary global maternal health strategies informed the decision to model improvements to ANC4+ coverage.

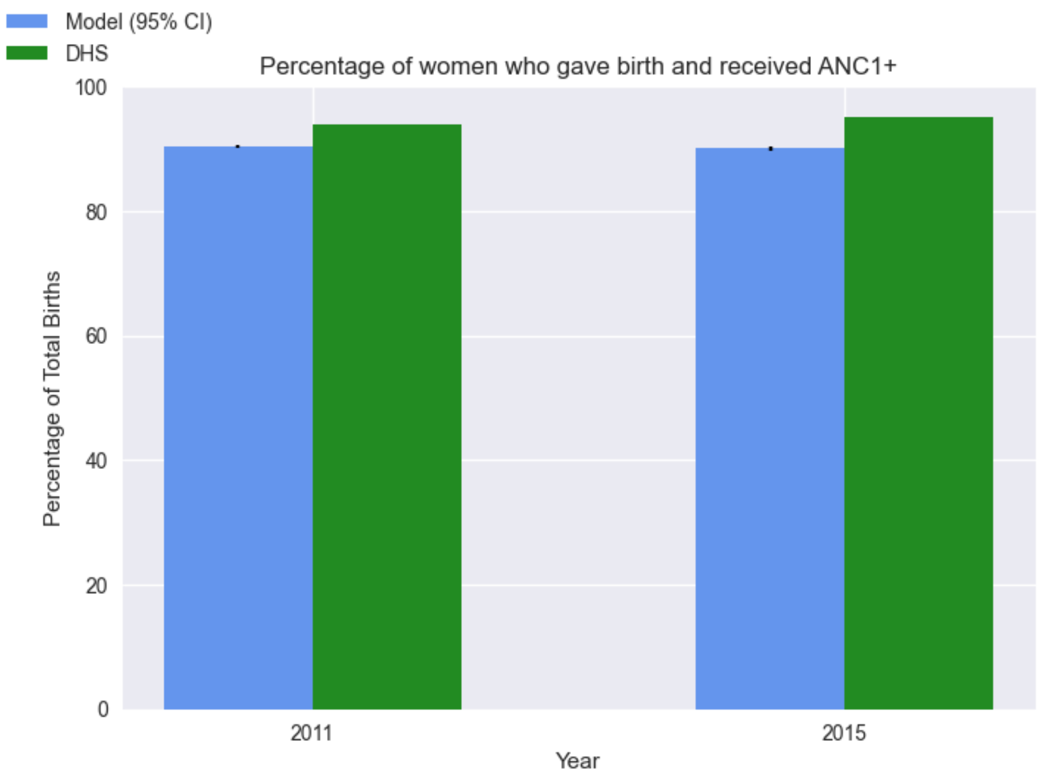

Figure S38 – Model output of the percentage of women who gave birth in the last year and received any ANC during pregnancy

The mean percentage of ANC1+ (95% CI) across 20 simulation runs per year outputted from the model (shown in blue) is plotted against estimates of ANC1+ coverage from the DHS (green).

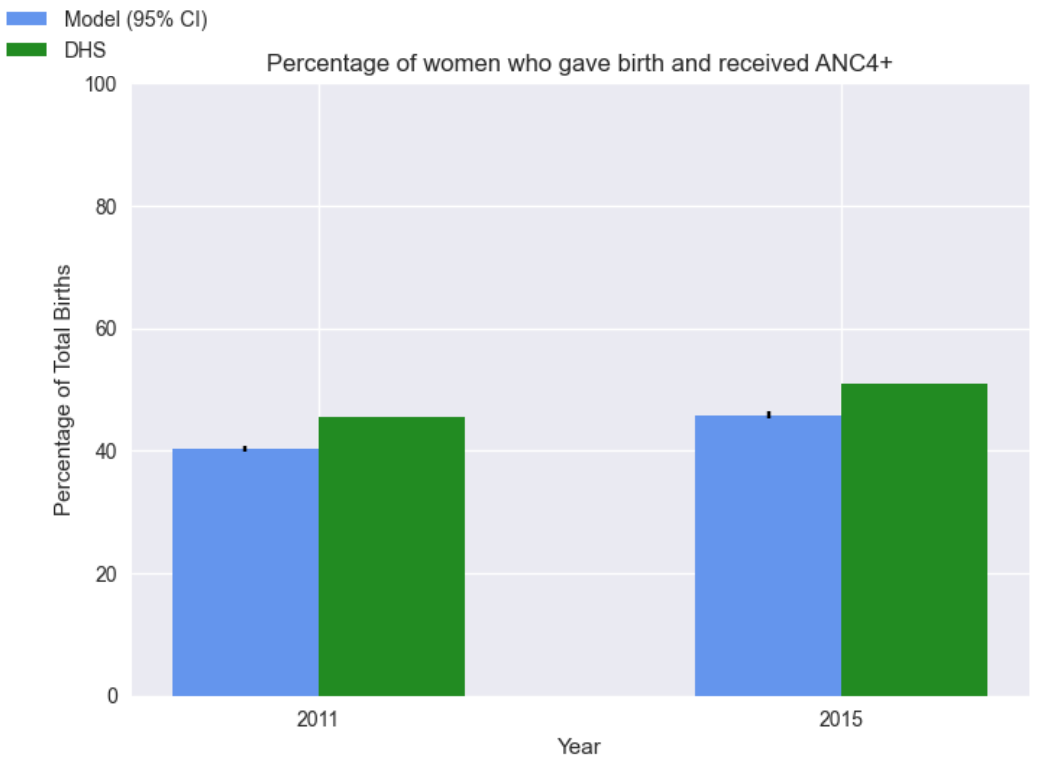

Figure S39 – Model output of the percentage of women who gave birth in the last year who attended at least four ANC visits during their pregnancy
The mean percentage of ANC4+ (95% CI) across 20 simulation runs per year outputted from the model (shown in blue) is plotted against estimates of ANC4+ coverage from the DHS (green).

Gestational age at first ANC contact

Alongside ensuring the model replicates ANC coverage it was deemed important to calibrate the model to data reporting the gestational age (GA) at which women attend their first ANC contact as seen in Figure S40. WHO and Malawian ANC guidelines recommend ANC is initiated early within pregnancy with the first visit at twelve weeks GA. As with ANC coverage, the DHS also reports timing of ANC initiation and as you can see within the figure the model is calibrated well to this data and demonstrates the shift towards earlier initiation of attendance between the two surveys.

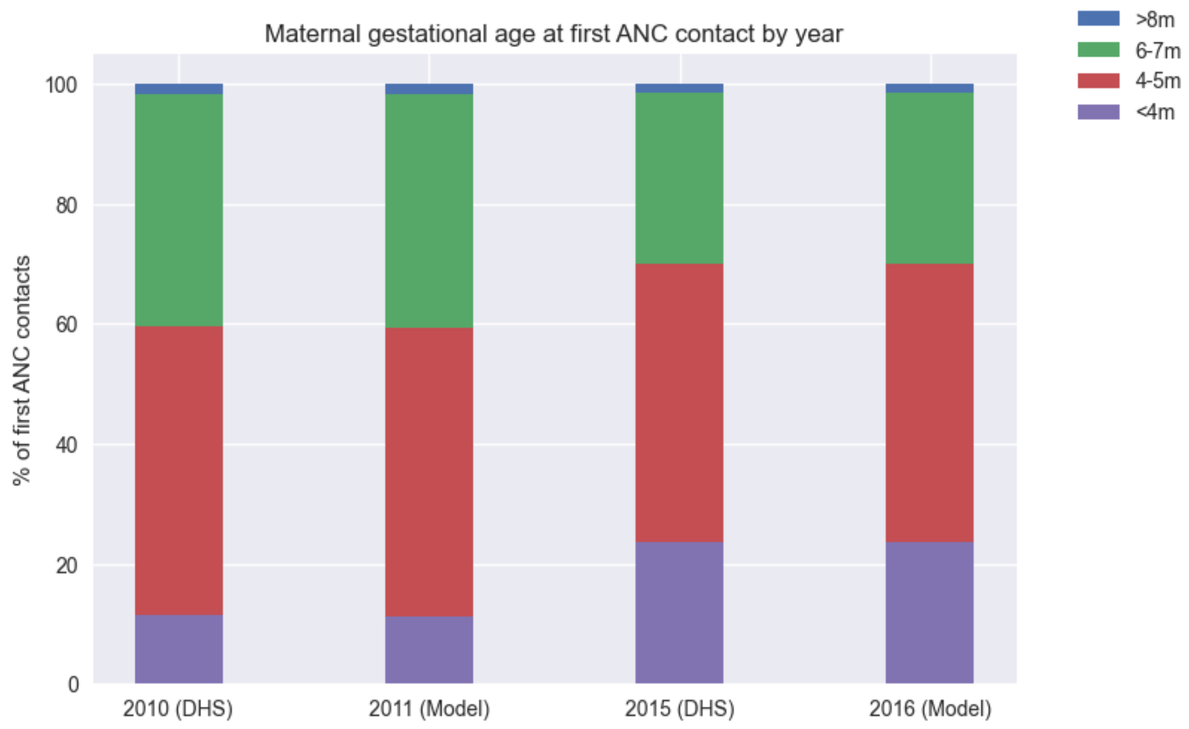

Figure S40[10] – Model output of the maternal gestational age at first ANC contact.by year
The gestational age (in months) of women attending ANC1 across 20 simulation runs is presented as the percentage of all ANC1 contacts. Model data is compared to estimates from the DHS data. Coloured sections of the stacked bar chart correspond to gestational age as shown in the key.

Total visits per pregnancy

Alongside calibration of coverage and timing of initiation, the model was calibrated to the average total number of visits per woman undertaking one or more contacts at birth. As shown in Figure S44, as with the other ANC calibration targets, the model fits well to data from the DHS.

[10] Model data from 2016 is shown as opposed to 2015 as some women in 2015 will have been scheduled their first ANC appointment in 2015 prior to the parameters updating to reflect changes observed in the DHS data.

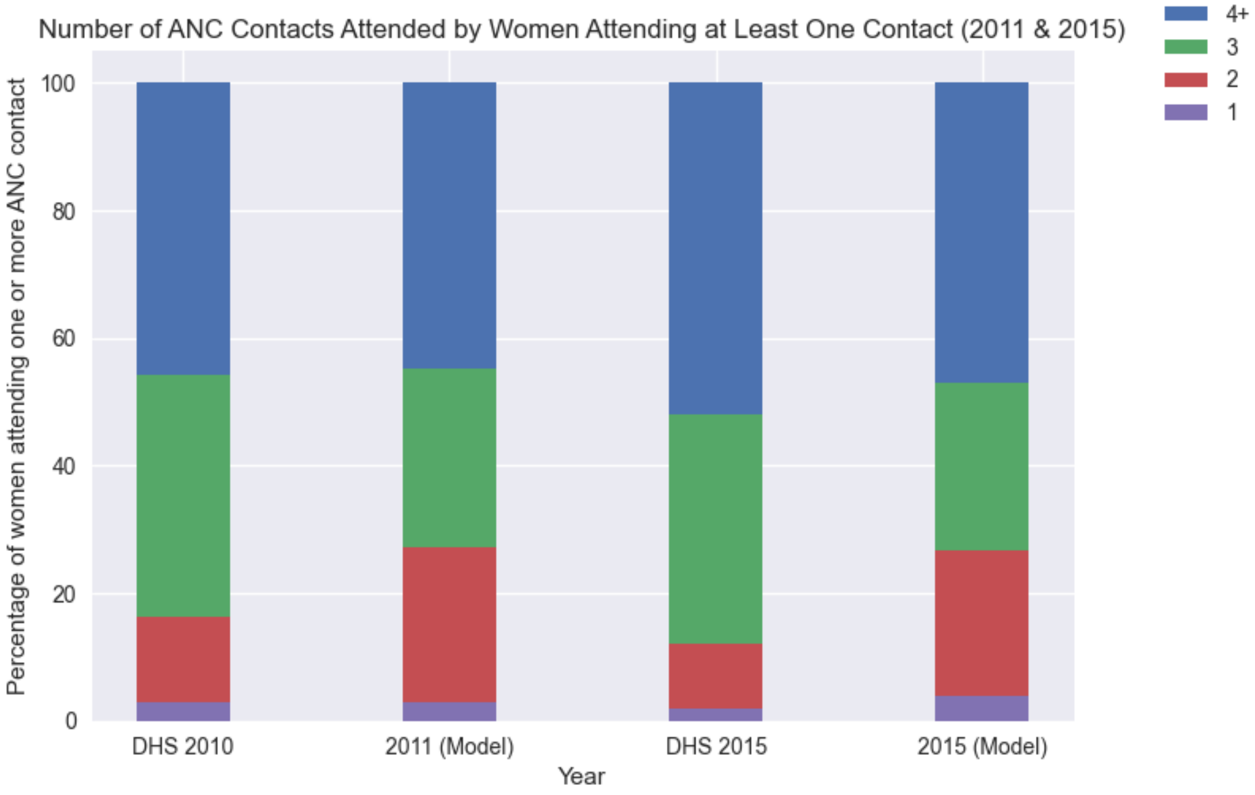

Figure S41 – Model output of the number of ANC contact attended by women as a percentage of the total women attending one or more contacts
The mean total number of ANC contacts attended by a woman at birth across 20 simulation runs is presented as the percentage of women attending one or more ANC contacts and is compared to estimates from the DHS. Coloured sections of the stacked bar chart correspond to number of contacts as shown in the key.

4.2.3.5 Intrapartum care coverage

Facility delivery rates

Figures S42 and S43 show the percentage of births which occur within health facilities in the model in 2010 and 2015 and the percentage of total births by all delivery locations including home. As with many of the other health-service coverage calibration outcomes, model outcomes were calibrated to the DHS data sets due to their assumed reliability. The model fits well to the data and demonstrates the reduction in homebirth during the calibration period.

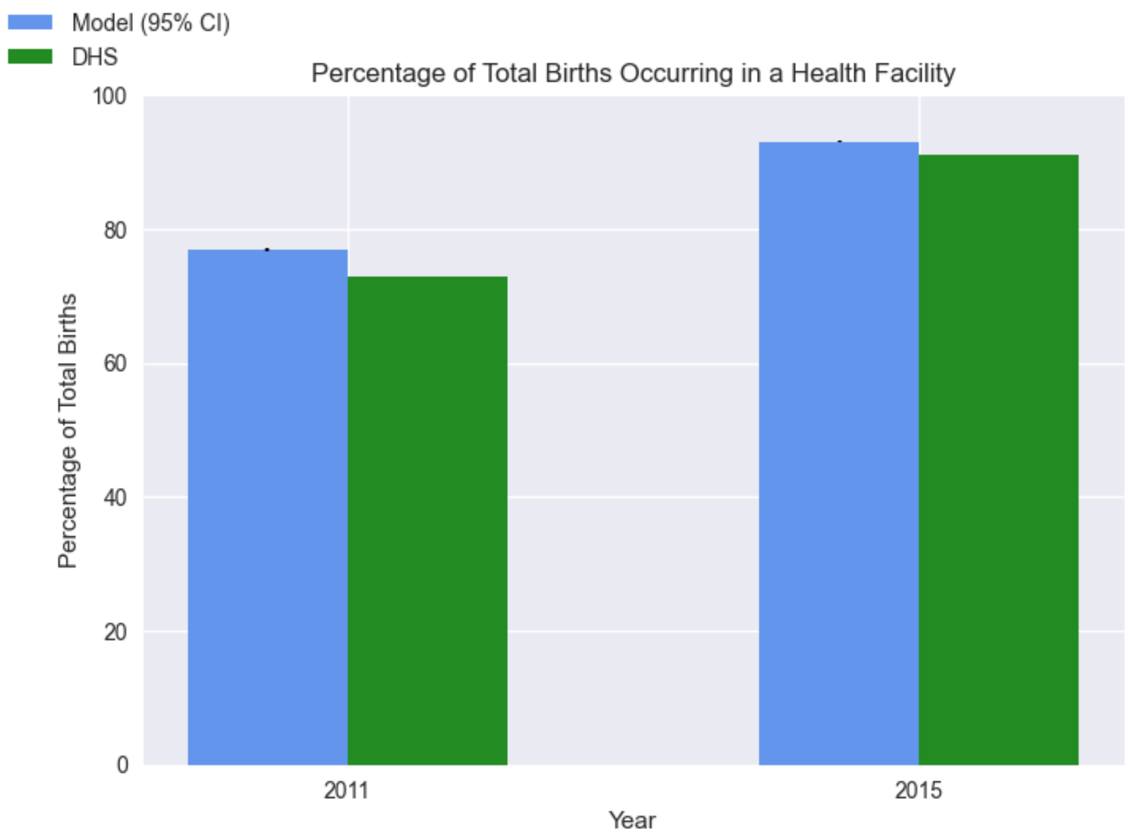

Figure S42 – Model output of the percentage of total births occurring in a health facility
The mean percentage of total births occurring in a health facility (95% CI) across 20 simulation runs per year outputted from the model (shown in blue) is plotted against estimates of total births occurring in a health facility from the DHS (green).

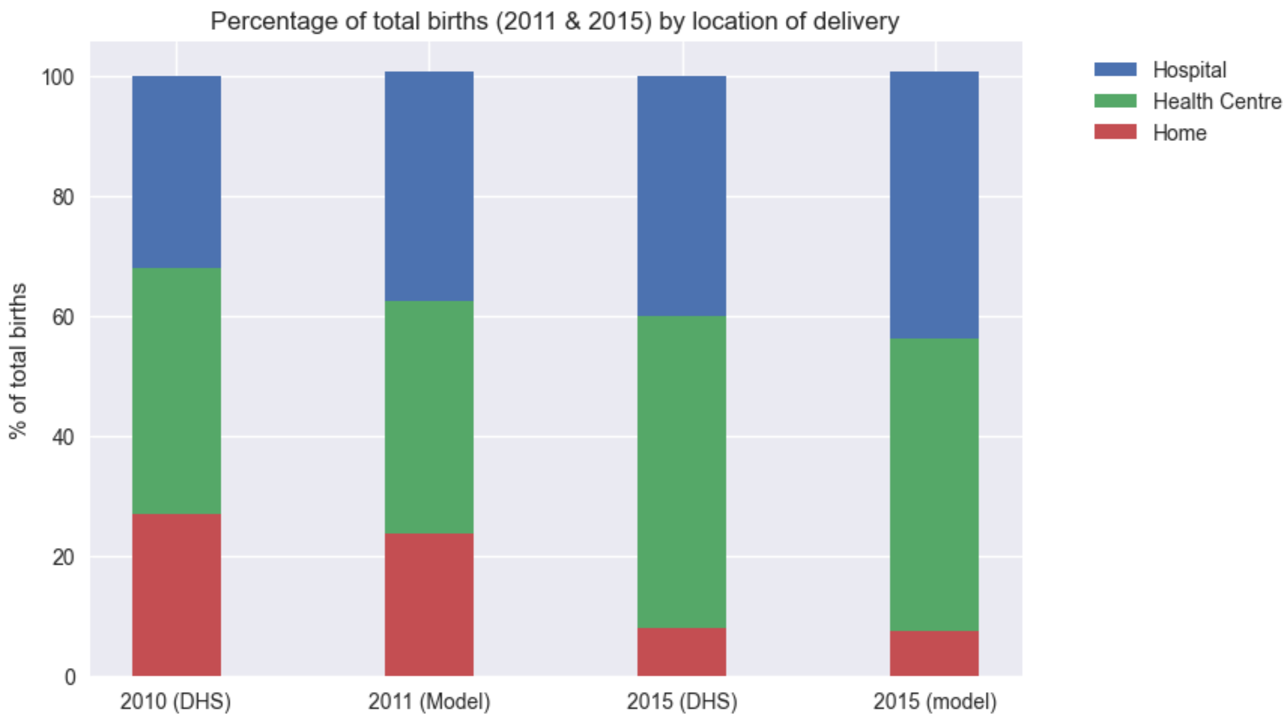

Figure S43- Model output of the percentage of total births location of delivery
The mean percentage of total births by location of delivery across 20 simulation runs is compared to estimates from the DHS. Coloured sections of the stacked bar chart correspond to location of delivery as shown in the key.

Caesarean section rate

Alongside ensuring the model accurately outputs the correct coverage of delivery setting, the model was also calibrated to delivery mode. In Figure S44 the proportion of total births delivered via CS is presented. Data points from the 2010 and 2015 EmONC surveys were used for model calibration, with the model outputting a slightly lower rate of CS than reported in these surveys. This could be due to an underestimation of the percentage of women with complications requiring CS in the model or due to greater availability of resources to conduct CS in Malawi than assumed in the model at present.

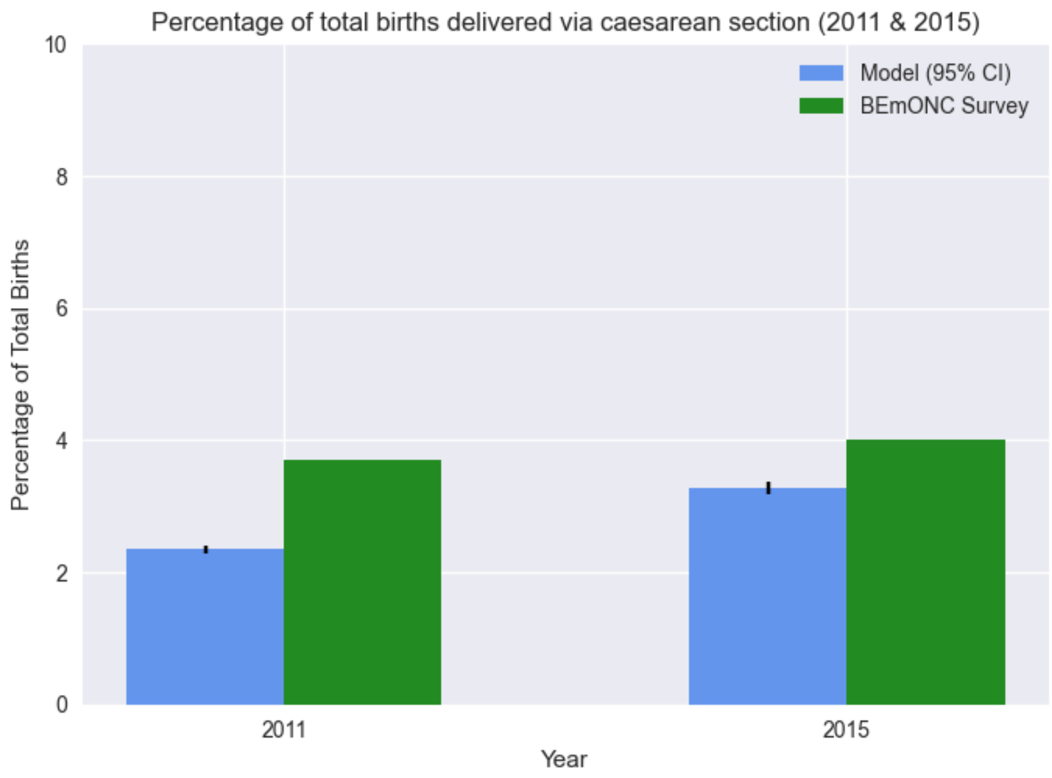

Figure S44 – Model output of the percentage of total births delivered via caesarean section
The mean percentage of caesarean section births (95% CI) across 20 simulation runs per year outputted from the model (shown in blue) is plotted against estimates of caesarean section delivery from the DHS (green).

4.2.3.6 Postnatal care coverage

Finally, Figures S45 and S46[11] show the coverage of both maternal and neonatal PNC outputted by the model compared to data points from the DHS surveys. As described in §2, PNC can be delivered immediately after birth or during the postnatal period, following care seeking from women in the community and as such women or newborns may attend PNC more than once. The coverage rates here refer to women and newborns who have received any amount of PNC at any point before the end of the postnatal period or neonatal period respectively.

[11] The 2010 DHS final report from Malawi does not report neonatal PNC coverage therefore model outputs are compared only the 2015 survey as evident from the figure.

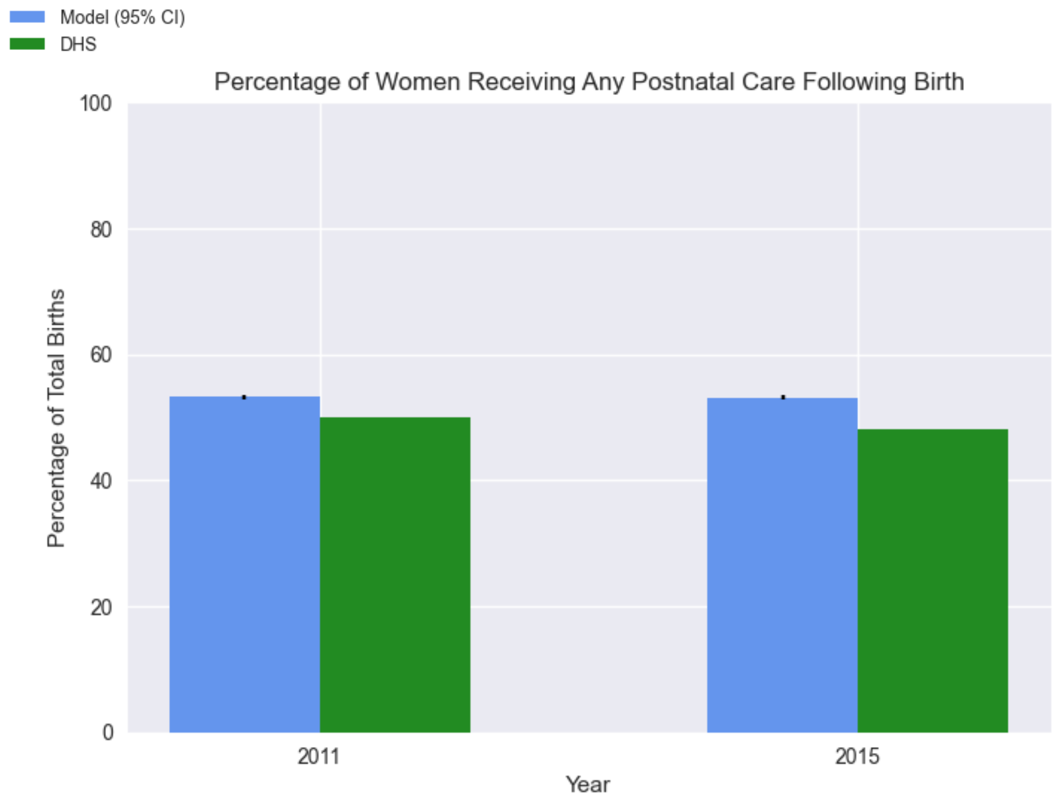

Figure S45- Model output of the percentage of women receiving any postnatal care following birth
The mean percentage of women receiving any postnatal care after birth (95% CI) across 20 simulation runs per year outputted from the model (shown in blue) is plotted against estimates of postnatal care after birth coverage from the DHS (green).

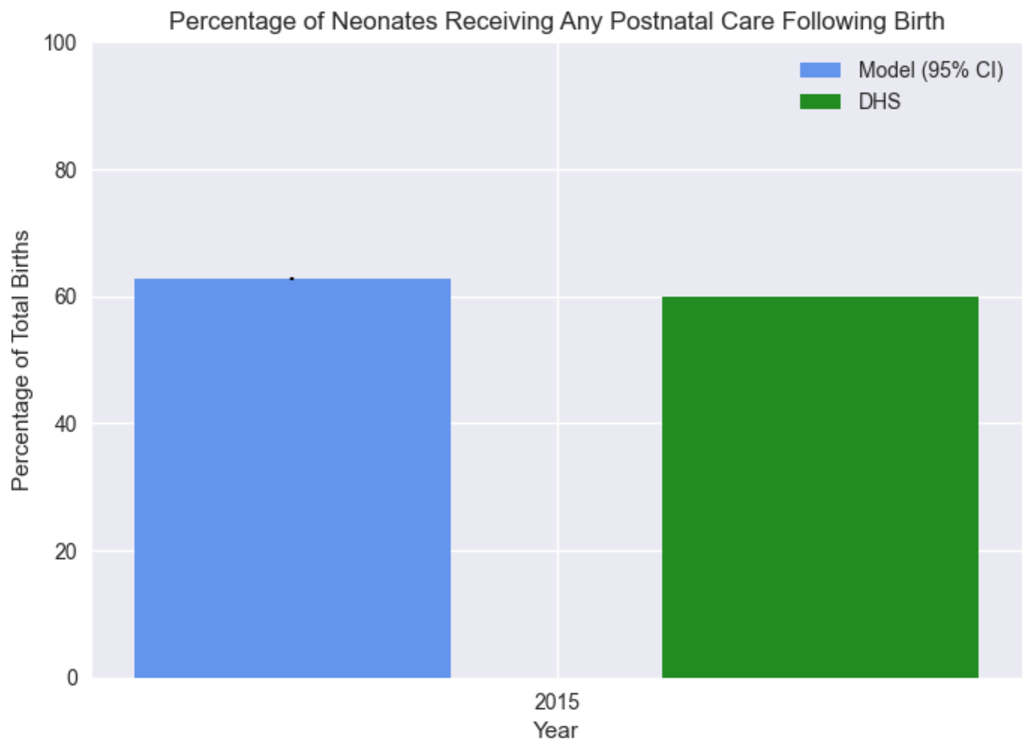

Figure S46 – Model output of the percentage of neonates who received any postnatal care after birth compared to calibration data
The mean percentage of newborns receiving any postnatal care after birth (95% CI) across 20 simulation runs per year outputted from the model (shown in blue) is plotted against estimates of newborn postnatal care after birth coverage from the DHS (green).

4.2.3.7 Complication incidence

Figures S47- S79 demonstrates the modelled rate/prevalence of each of the complications included in the MPHM alongside a relevant calibration target sourced either from Malawi or another relevant setting. The rationale for the rates and data used for these complications in the model has been provided in the complication descriptions §3.

We present the plots as blue line graphs over time with calibration data points in green. The shaded area around the model estimate represents the 95% CI. Where uncertainty around calibration estimates was available it has been presented – otherwise it has not been shown.

Plots start at 2011 so that they are stabilised after the first year of the simulation.

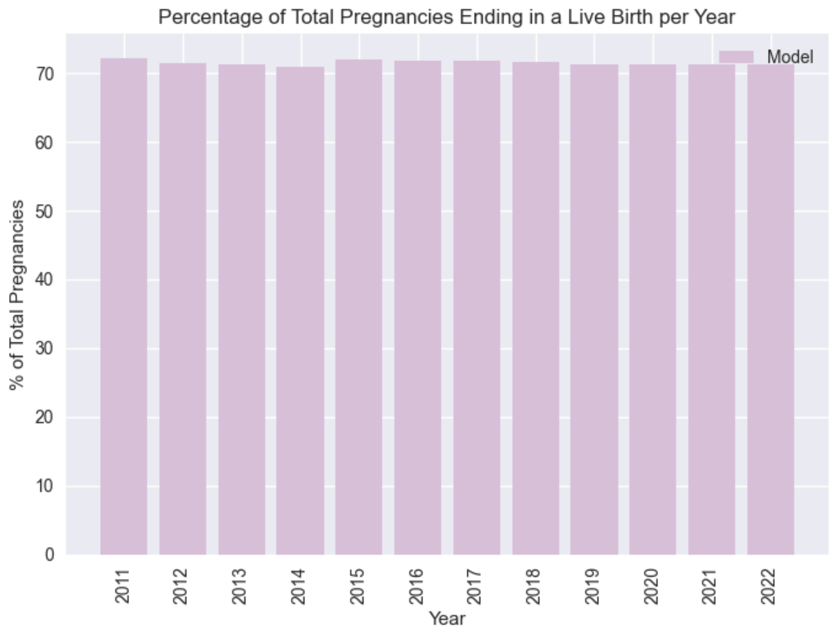

Figure S47 – Percentage of total modelled pregnancies ending in a live birth per year
The mean percentage of all modelled pregnancies which end in live birth across 20 simulation runs per year outputted from the model (shown in purple).

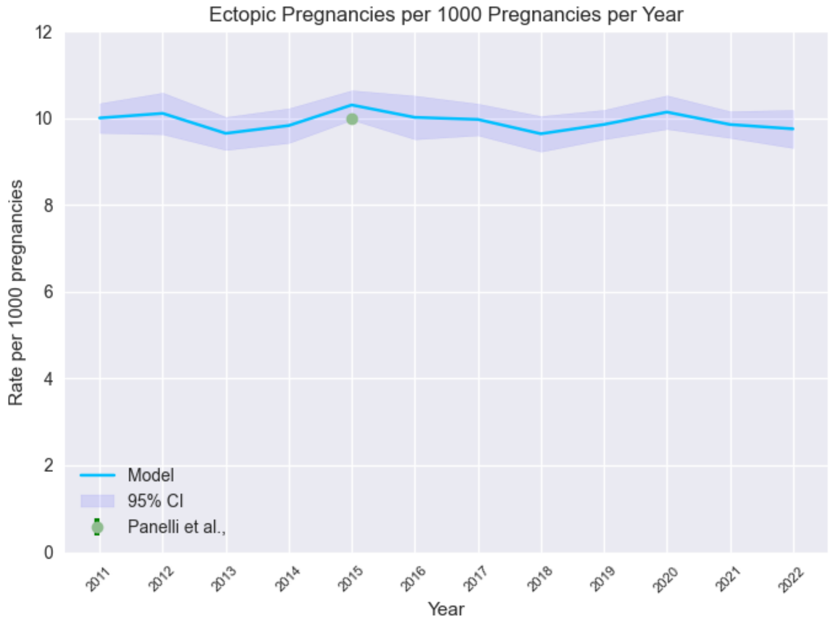

Figure S48 – Ectopic pregnancies per 1000 pregnancies per year in the model
The mean rate of ectopic pregnancy (95%) in the model per year across 20 simulation runs per year outputted from the model (shown in blue) compared to calibration data (shown in green).

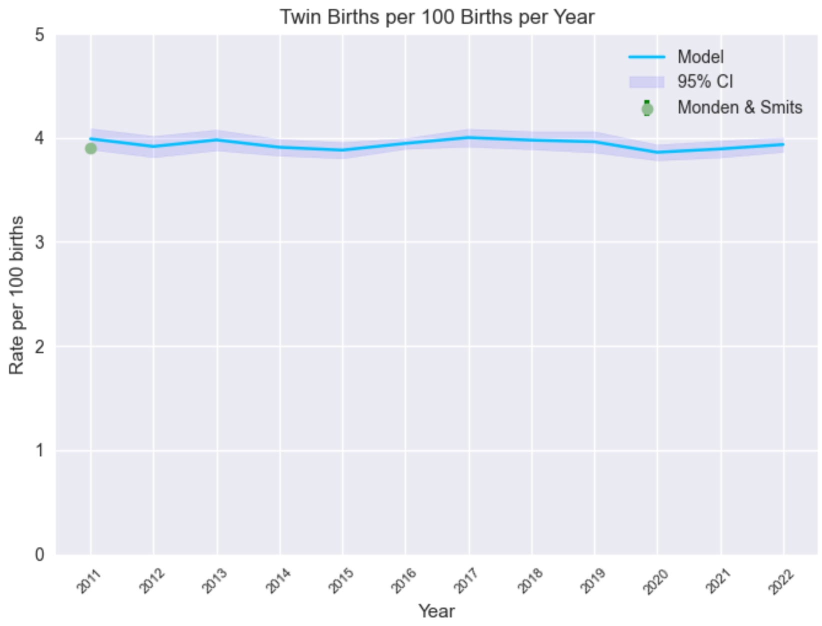

Figure S49 – Twin births per 100 births per year in the model
The mean rate of twin birth (95%) in the model per year across 20 simulation runs per year outputted from the model (shown in blue) compared to calibration data (shown in green).

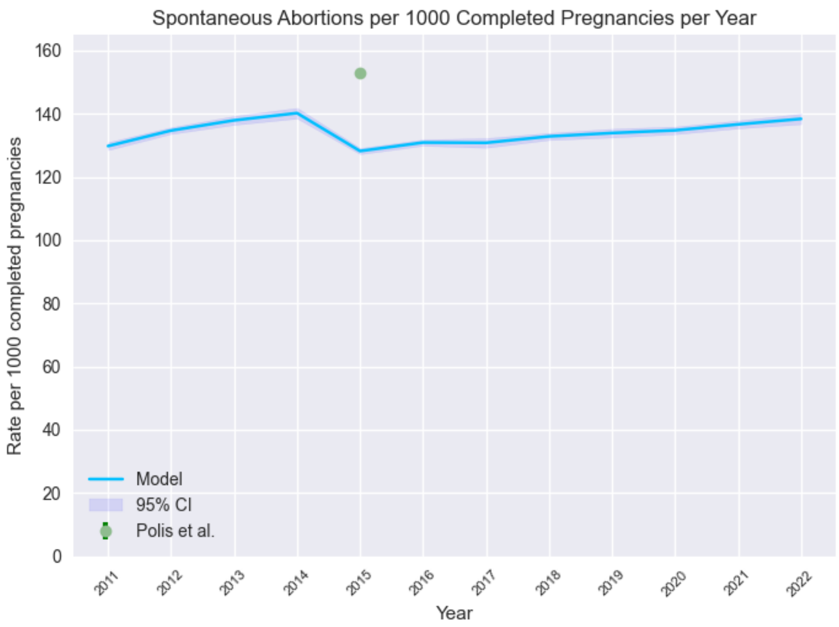

Figure S50 – Spontaneous abortions per 1000 complete pregnancies per year in the model
The mean rate of spontaneous abortion (95%) in the model per year across 20 simulation runs per year outputted from the model (shown in blue) compared to calibration data (shown in green).

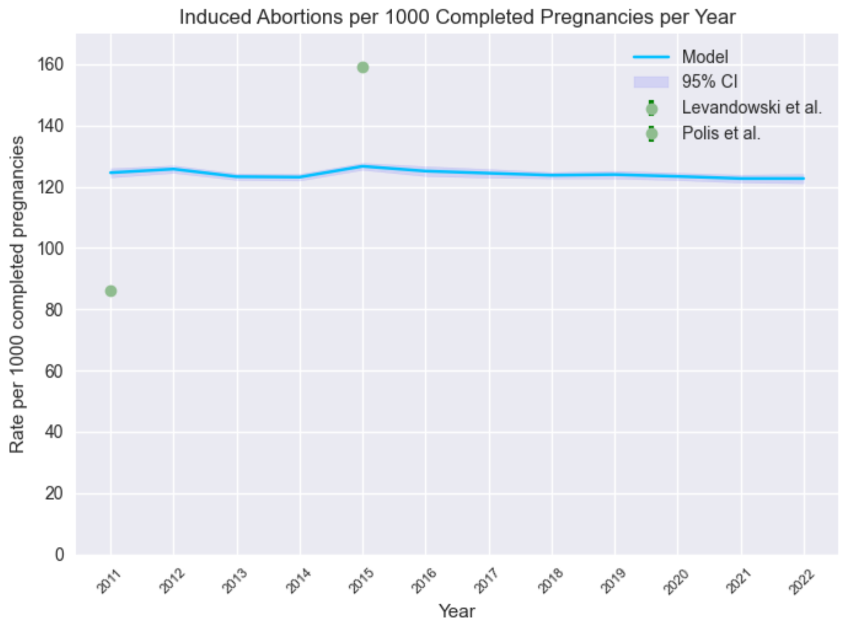

Figure S51 – Induced abortions per 1000 completed pregnancies per year in the model
The mean rate of induced abortion (95%) in the model per year across 20 simulation runs per year outputted from the model (shown in blue) compared to calibration data (shown in green).

When calibrated to the rate of abortion in Malawi estimated by Polis et al. (Table s40) the model generated too many deaths attributable to ineduced abortion given the modelled availablity of post abortion care. Therefore we opted to reduce the rate to an estimate between the data points shown on the figure

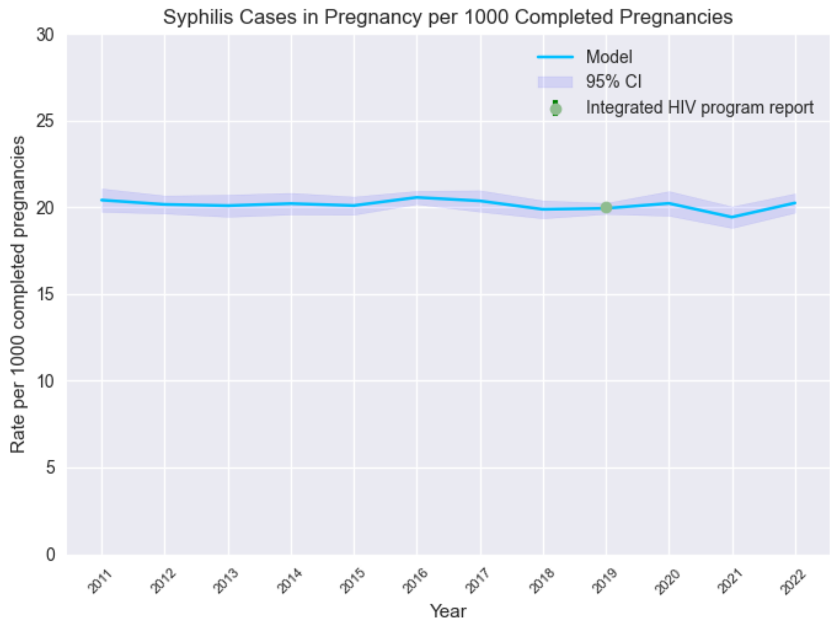

Figure S52 – Syphilis cases in pregnancies per 1000 completed pregnancies in the model
The mean rate of syphilis (95%) in the model per year across 20 simulation runs per year outputted from the model (shown in blue) compared to calibration data (shown in green).

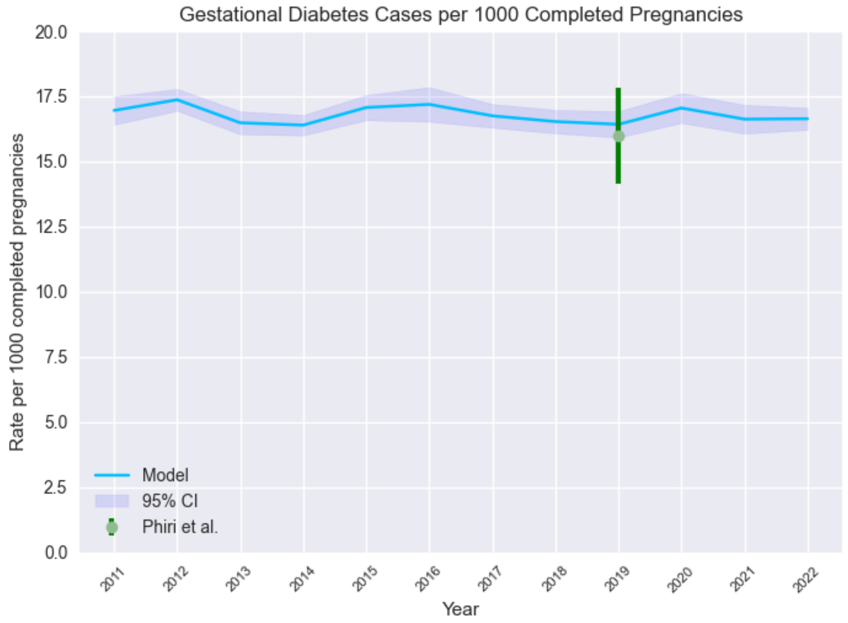

Figure S53 – Yearly rate of gestational diabetes mellitus within the model
The mean rate of gestational diabetes (95%) in the model per year across 20 simulation runs per year outputted from the model (shown in blue) compared to calibration data (shown in green).

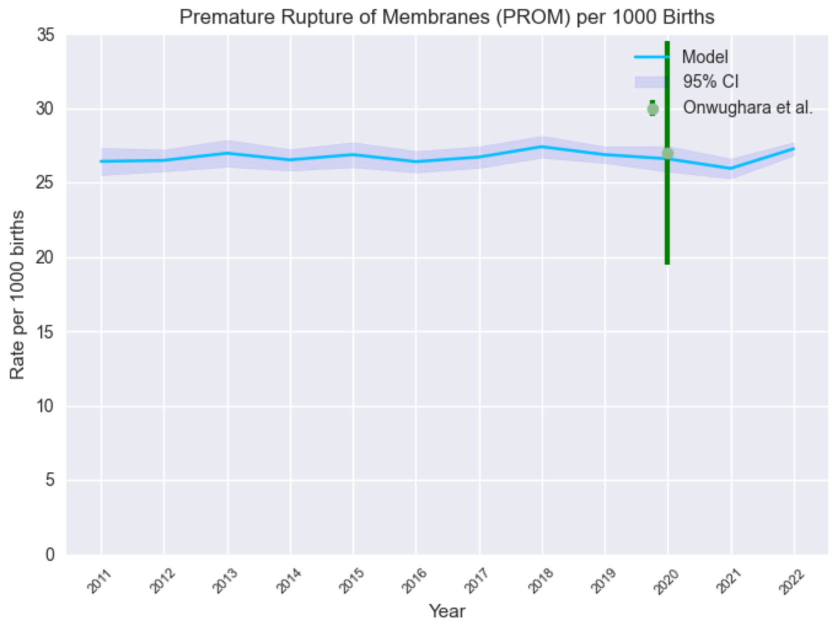

Figure S54 – Premature Rupture of Membranes per 1000 births per year in the model
The mean rate of PROM (95%) in the model per year across 20 simulation runs per year outputted from the model (shown in blue) compared to calibration data (shown in green).

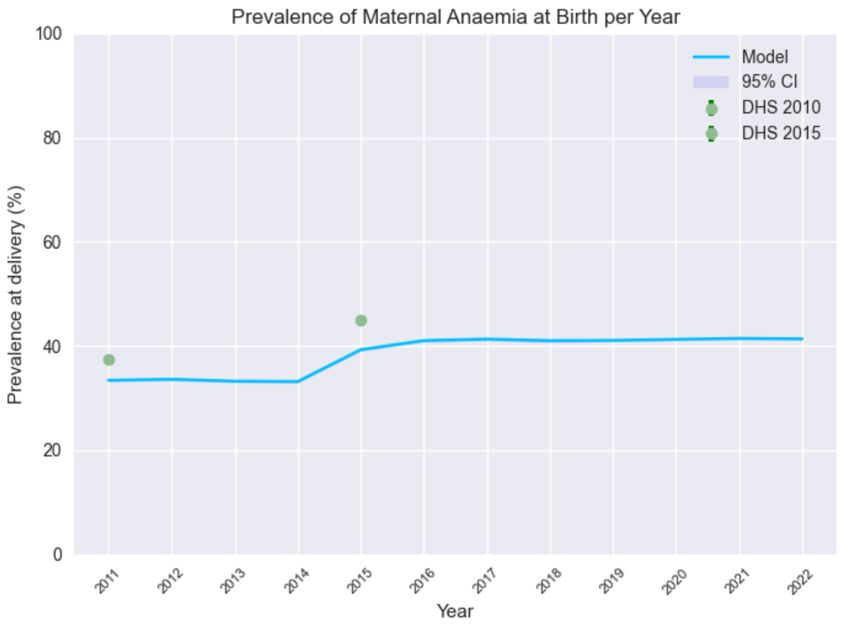

Figure S55 – Prevalence of maternal anaemia at birth per year in the model
The mean prevalence of anaemia at birth (95%) in the model per year across 20 simulation runs per year outputted from the model (shown in blue) compared to calibration data (shown in green).

Whilst the DHS does report uncertaintly around the estimate for anaemia prevalence in women of reproductive age, this is not presented for the estiamte of anaemia prevalence at birth for pregnant women and is therefore not reported in Figure S55.

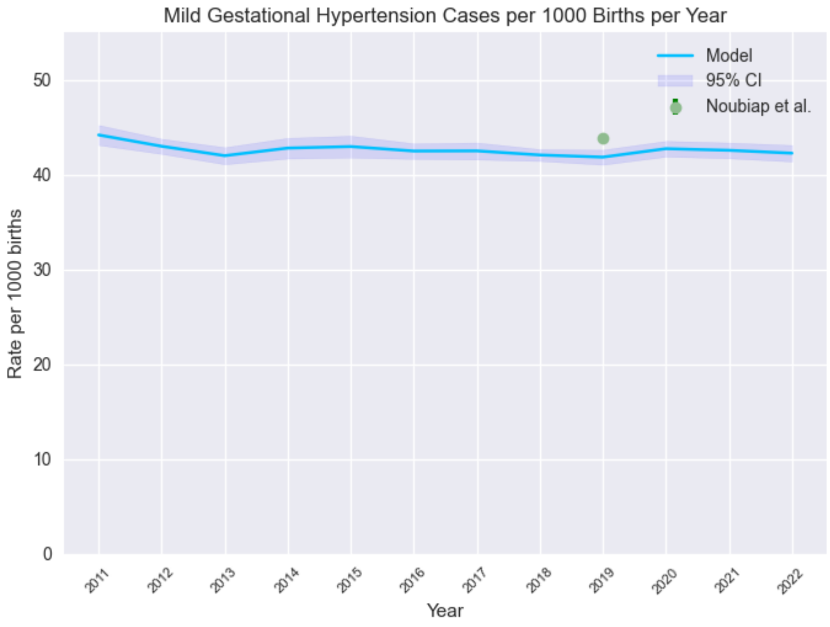

Figure S56 – Mild gestational hypertension cases per 1000 births per year in the model
The mean rate of mild gestational hypertension (95%) in the model per year across 20 simulation runs per year outputted from the model (shown in blue) compared to calibration data (shown in green).

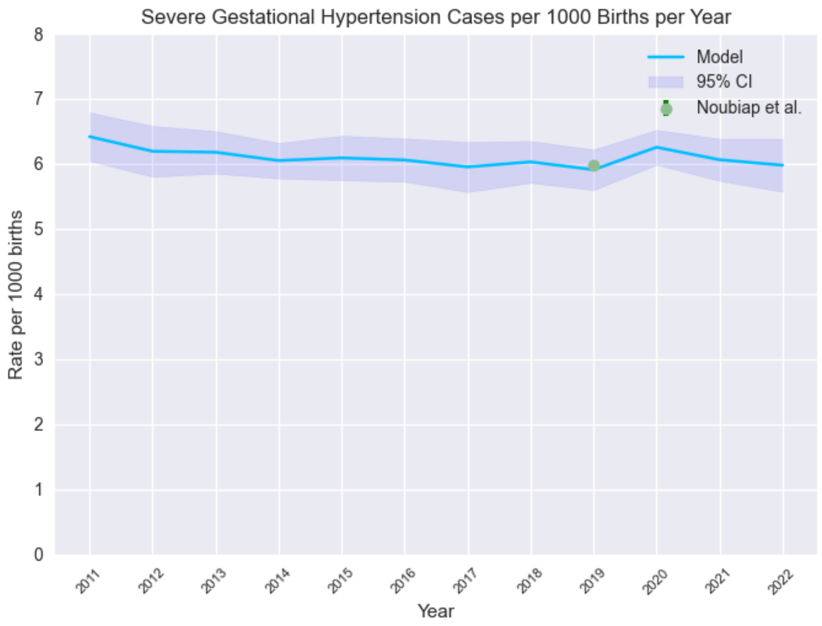

Figure S57 – Severe gestational hypertension cases per 1000 births per year in the model
The mean rate of severe gestational hypertension (95%) in the model per year across 20 simulation runs per year outputted from the model (shown in blue) compared to calibration data (shown in green).

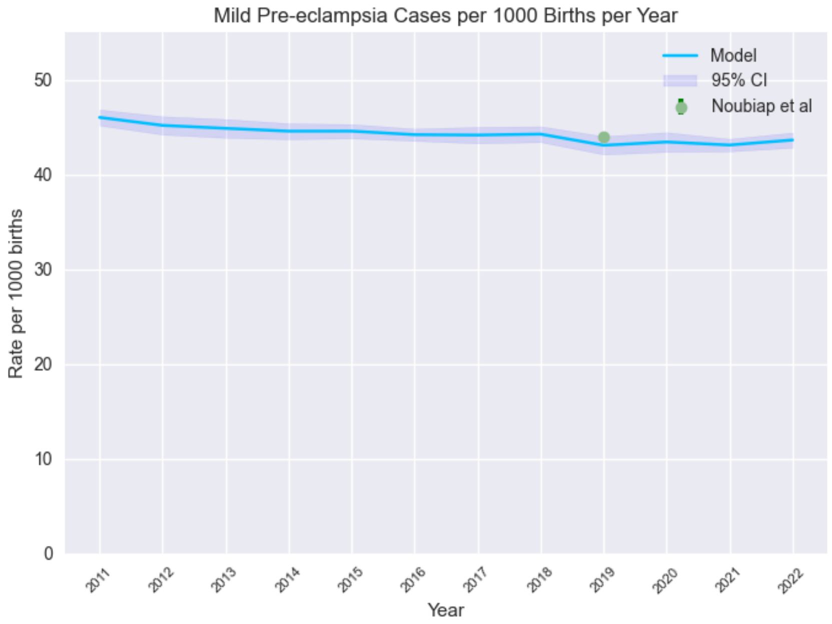

Figure S58 – Mild pre-eclampsia cases per 1000 births per year in the model
The mean rate of pre-eclampsia (95%) in the model per year across 20 simulation runs per year outputted from the model (shown in blue) compared to calibration data (shown in green).

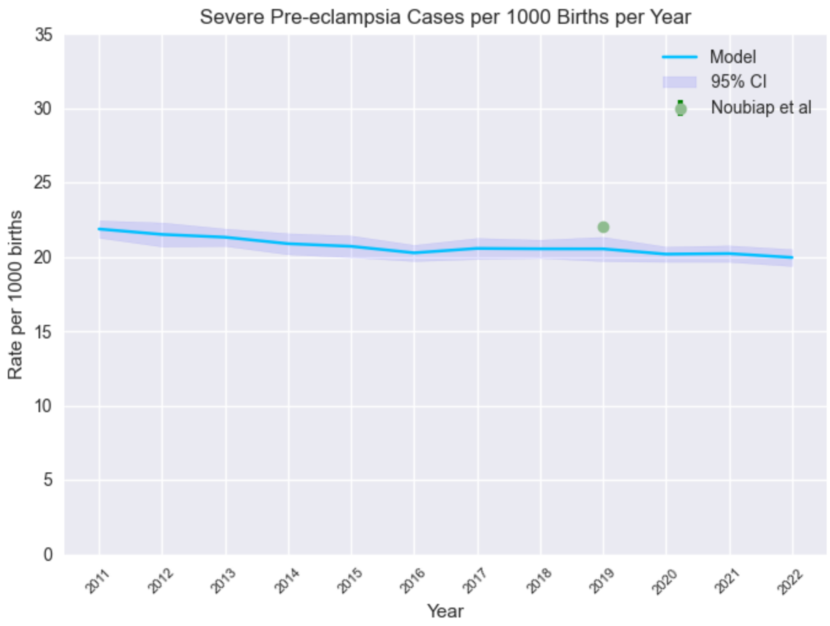

Figure S59 – Severe pre-eclampsia cases per 1000 births per year in the model
The mean rate of severe pre-eclampsia (95%) in the model per year across 20 simulation runs per year outputted from the model (shown in blue) compared to calibration data (shown in green).

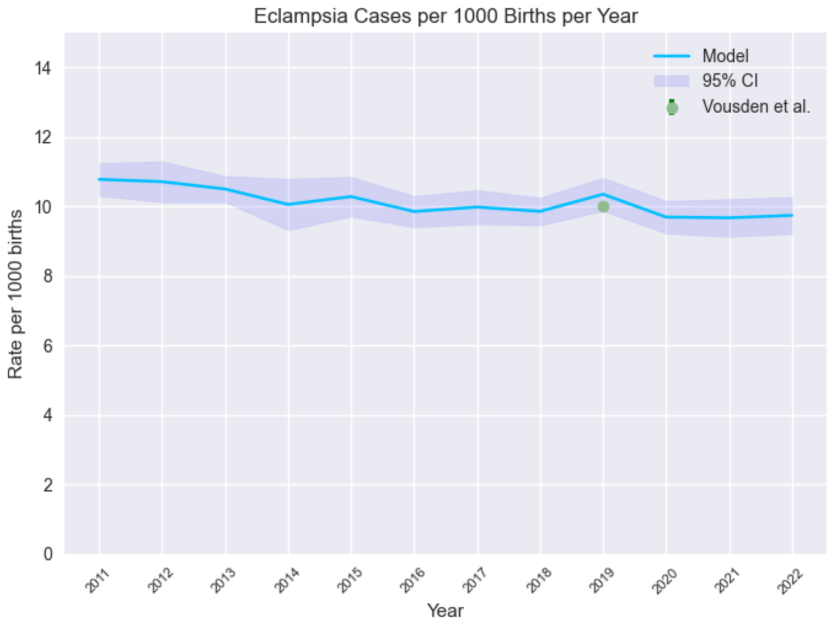

Figure S60 – Eclampsia cases per 1000 births per year in the model
The mean rate of eclampsia (95%) in the model per year across 20 simulation runs per year outputted from the model (shown in blue) compared to calibration data (shown in green).

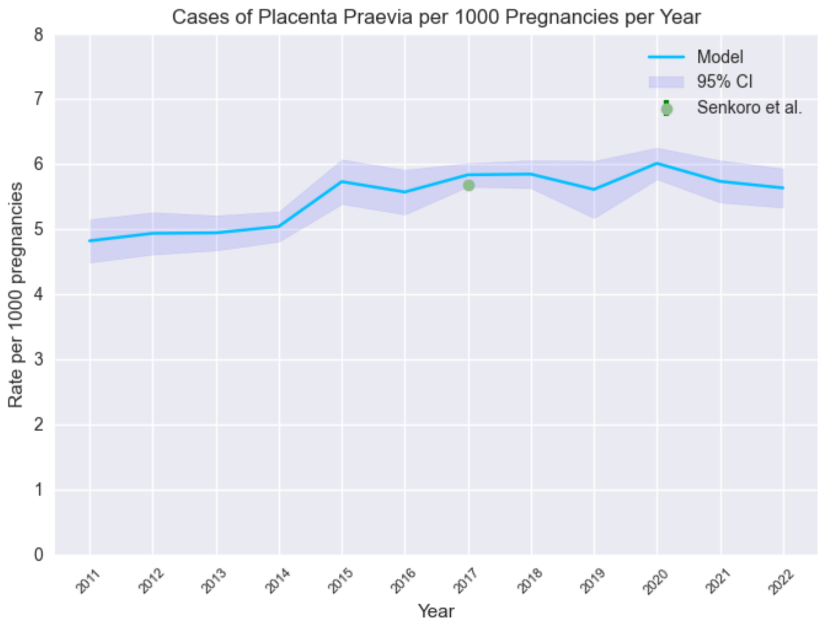

Figure S61 – Placenta praevia per 1000 pregnancies per year in the model
The mean rate of placenta praevia (95%) in the model per year across 20 simulation runs per year outputted from the model (shown in blue) compared to calibration data (shown in green).

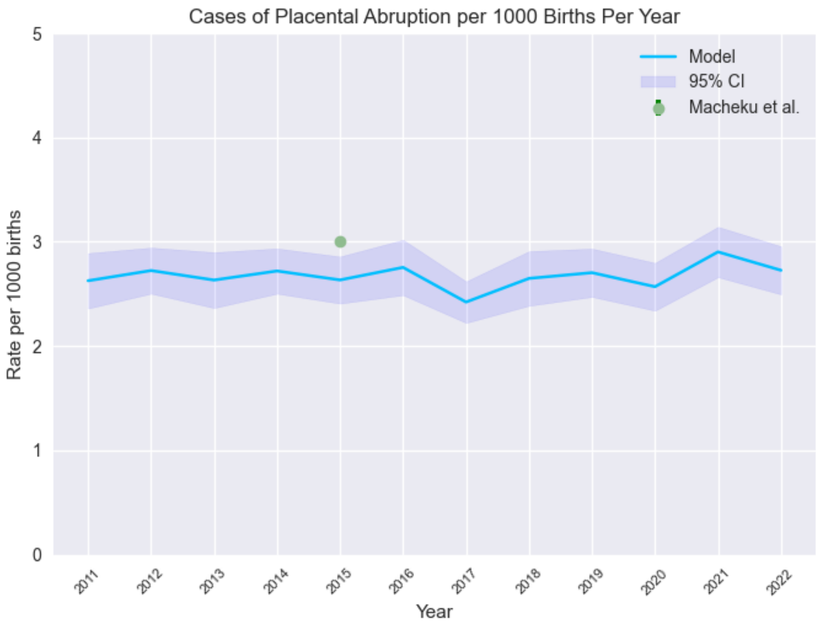

Figure S62 – Placental abruption per 1000 births per year in the model
The mean rate of placental abruption (95%) in the model per year across 20 simulation runs per year outputted from the model (shown in blue) compared to calibration data (shown in green).

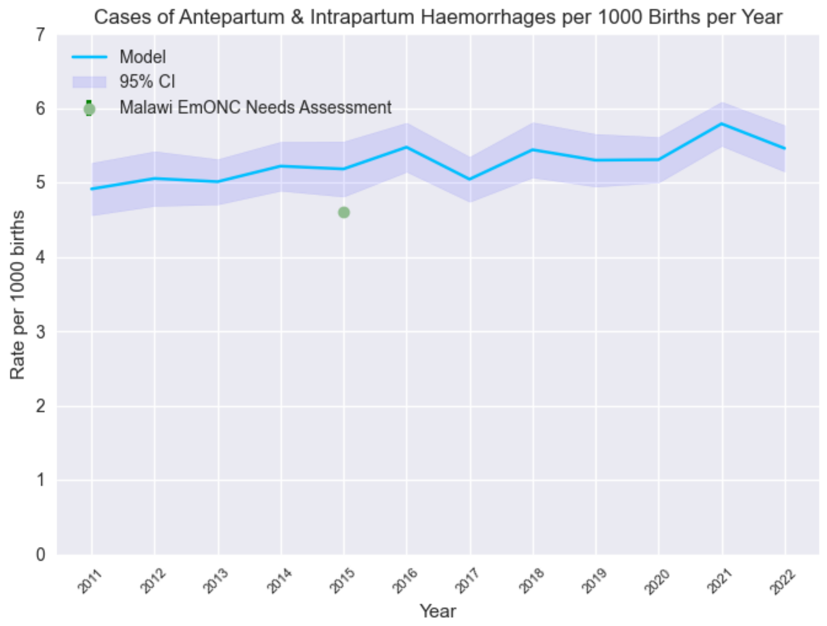

Figure S63 – Antepartum and intrapartum haemorrhages per 1000 births per year in the model
The mean rate of antepartum and intrapartum haemorrhage (95%) in the model per year across 20 simulation runs per year outputted from the model (shown in blue) compared to calibration data (shown in green).

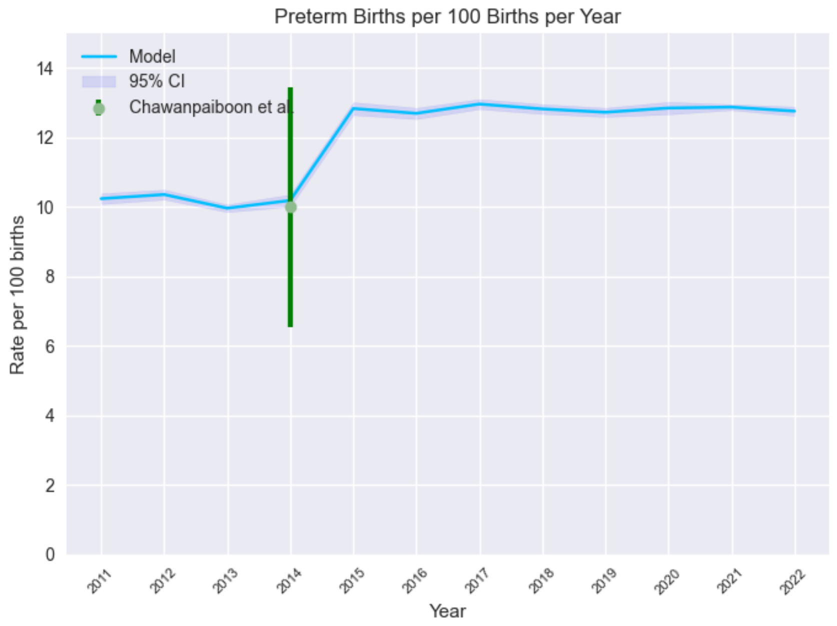

Figure S64 – Preterm births per 100 births per year in the model
The mean rate of preterm birth (95%) in the model per year across 20 simulation runs per year outputted from the model (shown in blue) compared to calibration data (shown in green).

The increase in rate of preterm birth after 2015 evident in Figure S64 is likely associated with increased prevelance of anaemia (a predictor of preterm birth) as shown above.

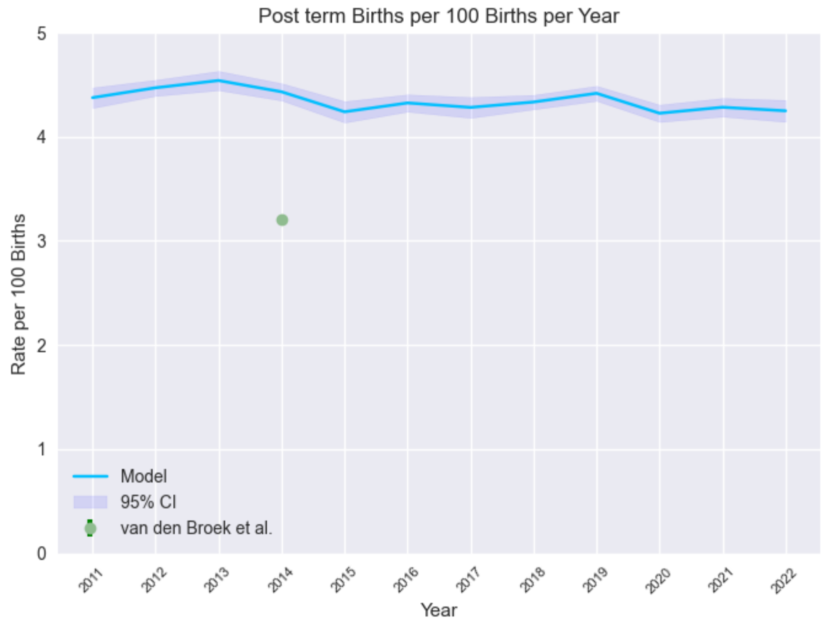

Figure S65 – Post term births per 100 births per year in the model
The mean rate of post term birth (95%) in the model per year across 20 simulation runs per year outputted from the model (shown in blue) compared to calibration data (shown in green).

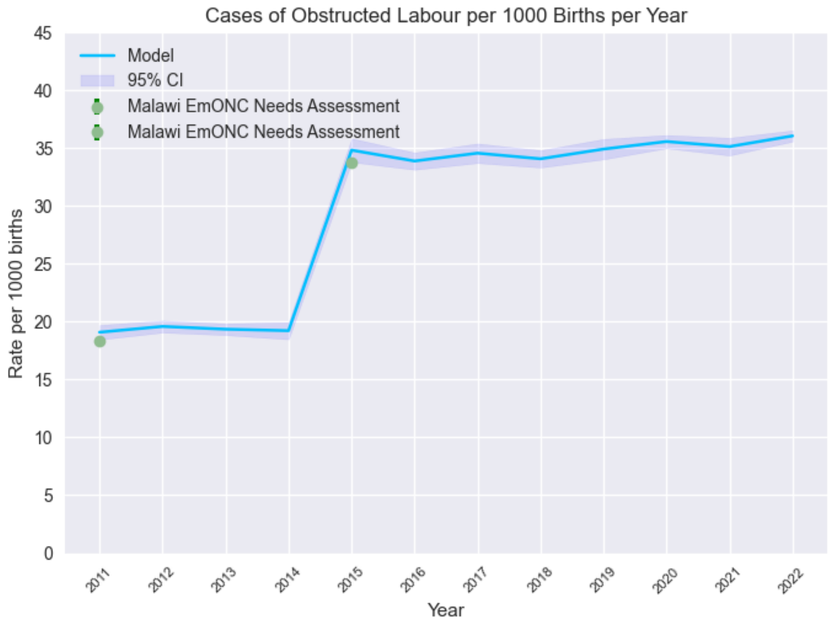

Figure S66 – Obstructed labour per 1000 births per year in the model
The mean rate of obstructed labour (95%) in the model per year across 20 simulation runs per year outputted from the model (shown in blue) compared to calibration data (shown in green).

Change in the observed rate of obstructed labour cases between 2010 and 2015 EmONC needs assessment surveys in Malawi could be due to many factors such as a change in the distribution of predictive factors.

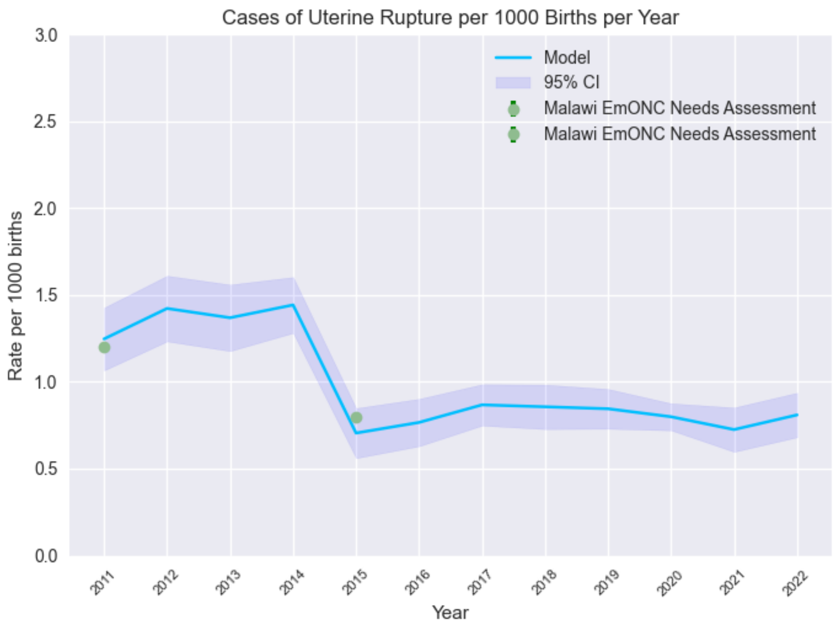

Figure S67 – Uterine ruptures per 1000 births per year in the model
The mean rate of uterine rupture (95%) in the model per year across 20 simulation runs per year outputted from the model (shown in blue) compared to calibration data (shown in green).

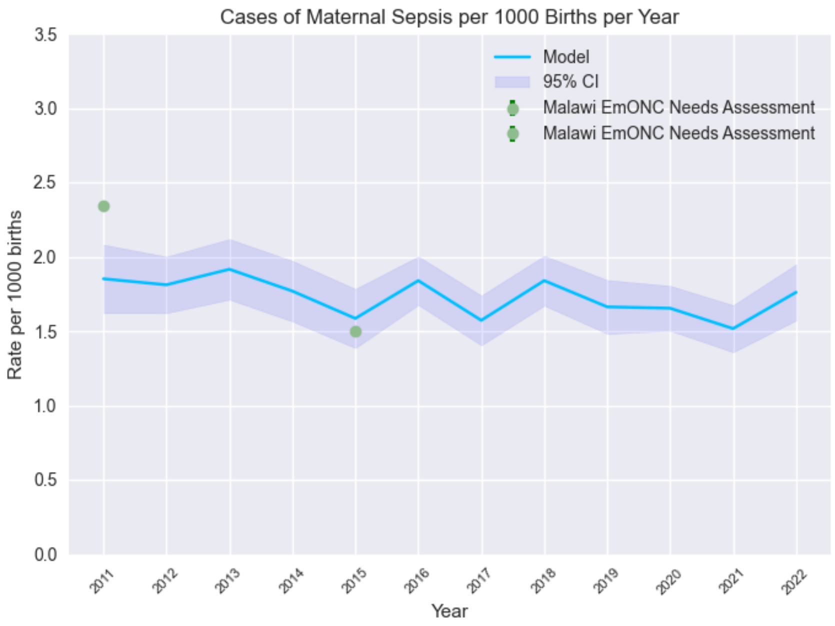

Figure S68 – Maternal sepsis per 1000 births per year in the model
The mean rate of maternal sepsis (95%) in the model per year across 20 simulation runs per year outputted from the model (shown in blue) compared to calibration data (shown in green).

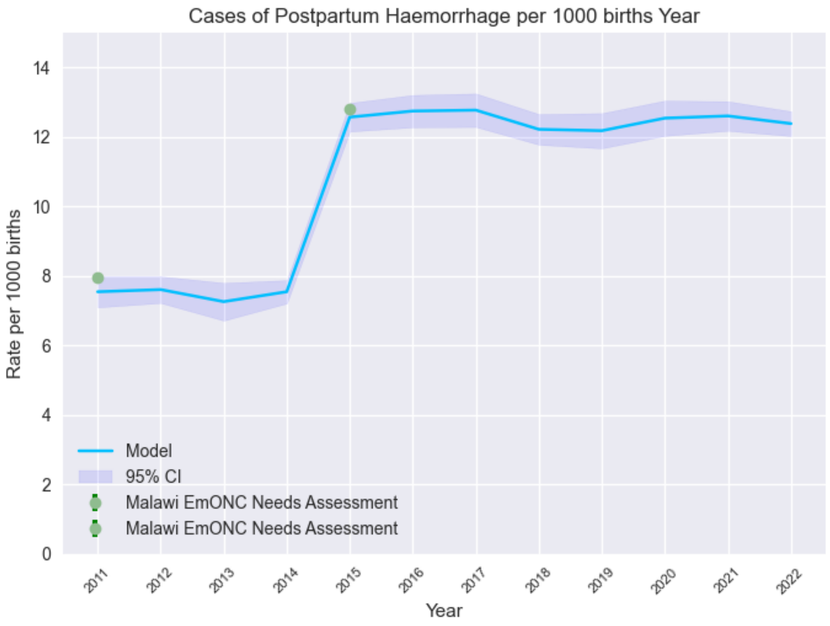

Figure S69 – Postpartum haemorrhages per 1000 births per year in the model
The mean rate of postpartum haemorrhage (95%) in the model per year across 20 simulation runs per year outputted from the model (shown in blue) compared to calibration data (shown in green).

Similarly to obstructed labour, change in the observed rate of postpartum haemorrhage cases between 2010 and 2015 in Malawi could be due to many factors such as a change in the distribution of predictive factors.

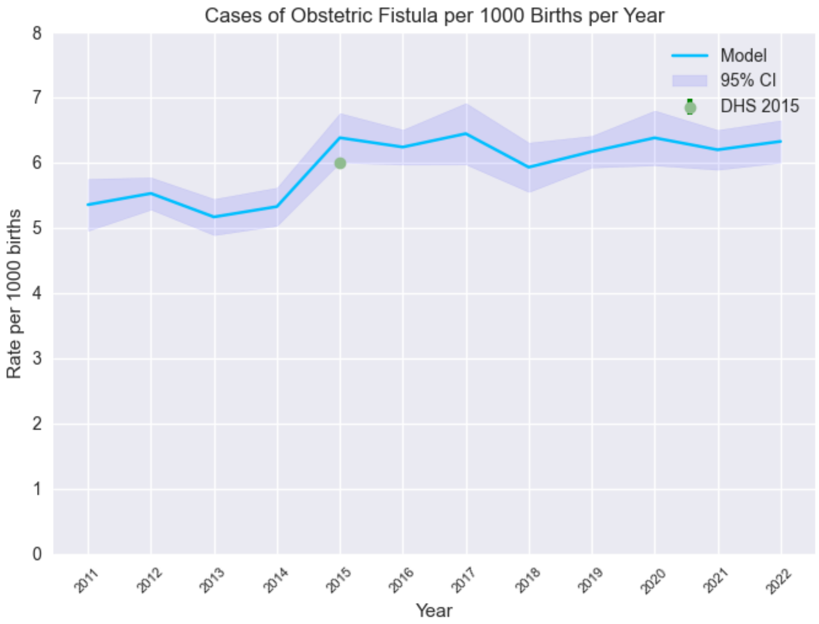

Figure S70 – Obstetric fistulas per 1000 births per year in the model
The mean rate of obstetric fistula (95%) in the model per year across 20 simulation runs per year outputted from the model (shown in blue) compared to calibration data (shown in green).

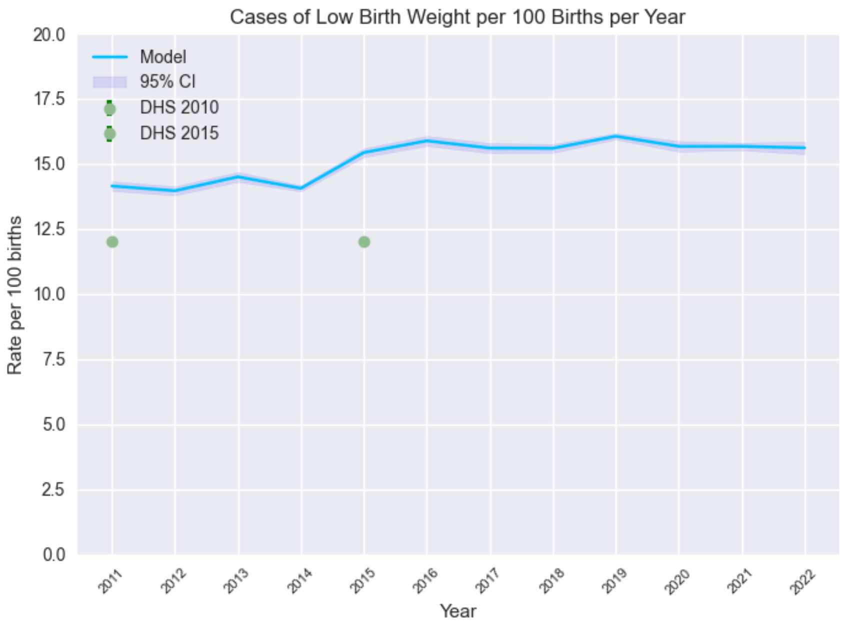

Figure S71 – Low birth weight births per 100 births per year in the model
The mean rate of low-birth-weight births (95%) in the model per year across 20 simulation runs per year outputted from the model (shown in blue) compared to calibration data (shown in green).

The model generates a slightly greater number of lowbirth weight newborns than reported within the DHS as shown in Figure S71. This is attributable to the distribution used to determine birthweight which can be adjusted in future iterations of the model. Currently lowbirth weight is not modelled to directly effect outcomes in the MPHM.

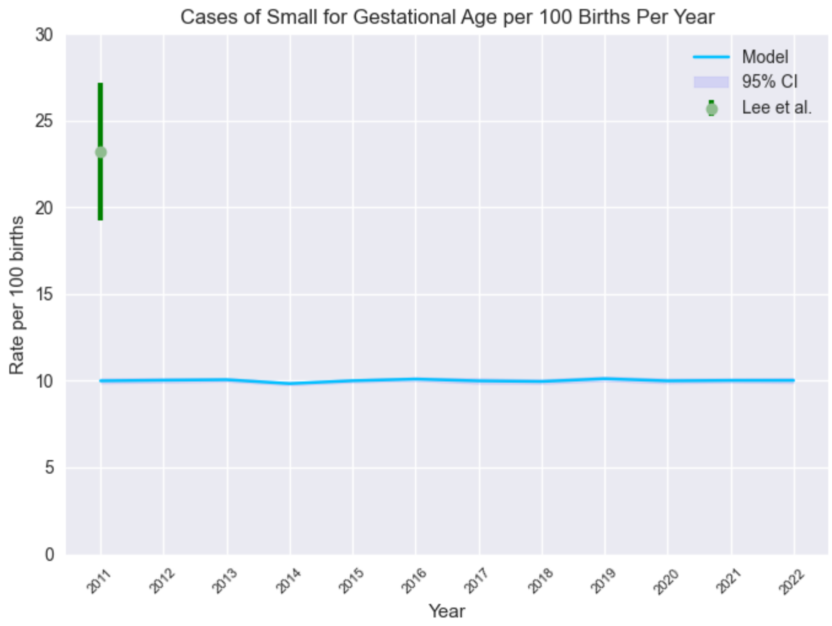

Figure S72 – Small for gestational age births per 100 births per year in the model
The mean rate of small for gestational age births (95%) in the model per year across 20 simulation runs per year outputted from the model (shown in blue) compared to calibration data (shown in green).

Alongside fewer low birthweight newborns the model also outputs fewer small for GA newborns, this is also due to the distribution used for birthweight. Similarly to low birth weight, small for GA does not directly effect any outcomes in the MPHM

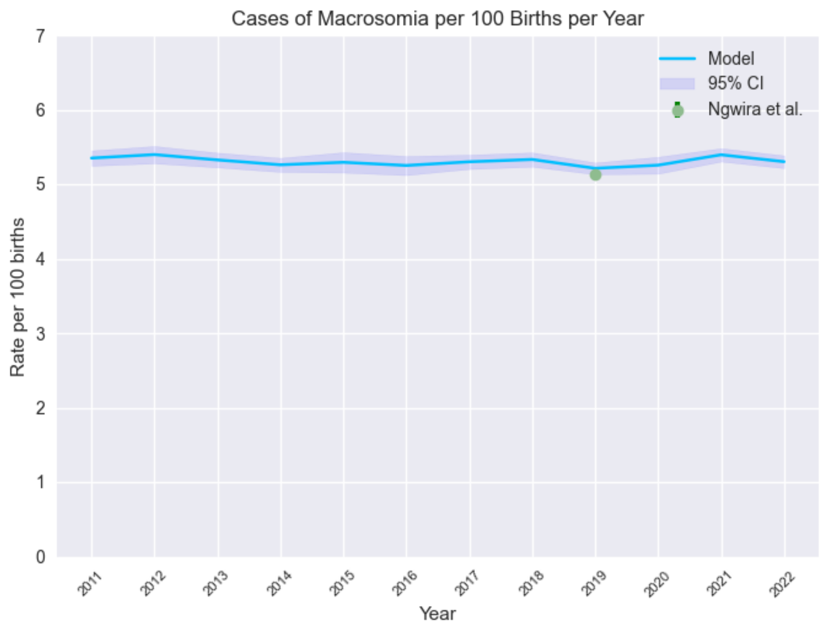

Figure S73 – Macrosomic births per 100 births per year in the model
The mean rate of macrosomic births (95%) in the model per year across 20 simulation runs per year outputted from the model (shown in blue) compared to calibration data (shown in green).

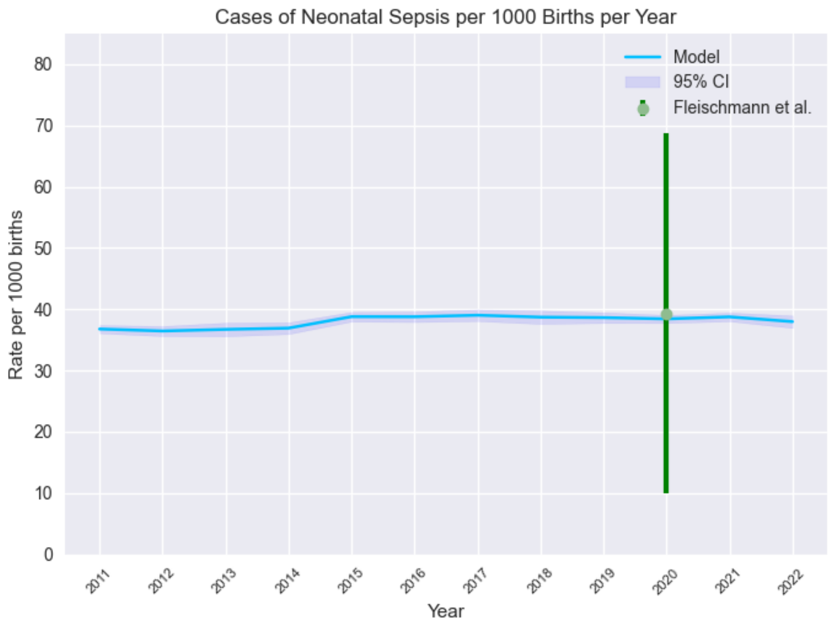

Figure S74 – Neonatal sepsis per 1000 births per year in the model
The mean rate of neonatal sepsis (95%) in the model per year across 20 simulation runs per year outputted from the model (shown in blue) compared to calibration data (shown in green).

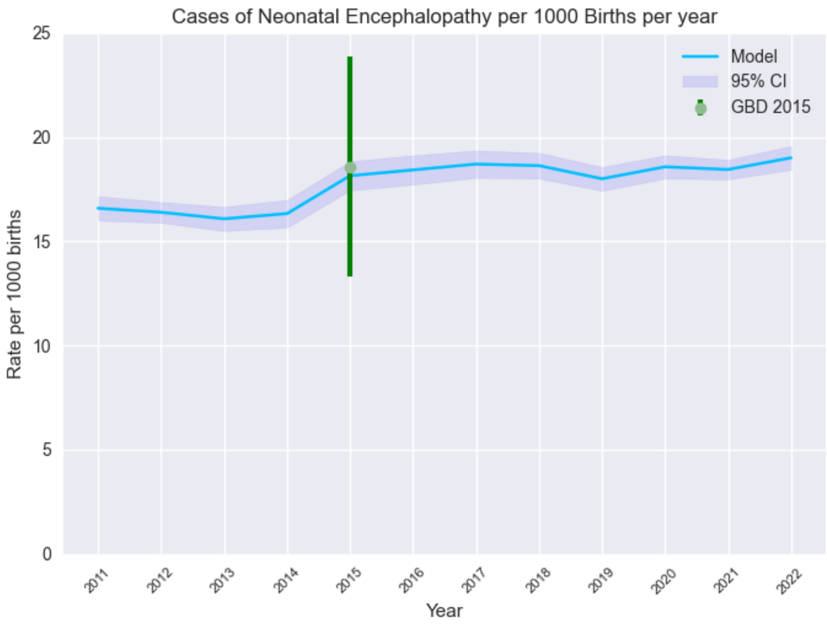

Figure S75 – Neonatal encephalopathy per 1000 births per year in the model
The mean rate of neonatal encephalopathy (95%) in the model per year across 20 simulation runs per year outputted from the model (shown in blue) compared to calibration data (shown in green).

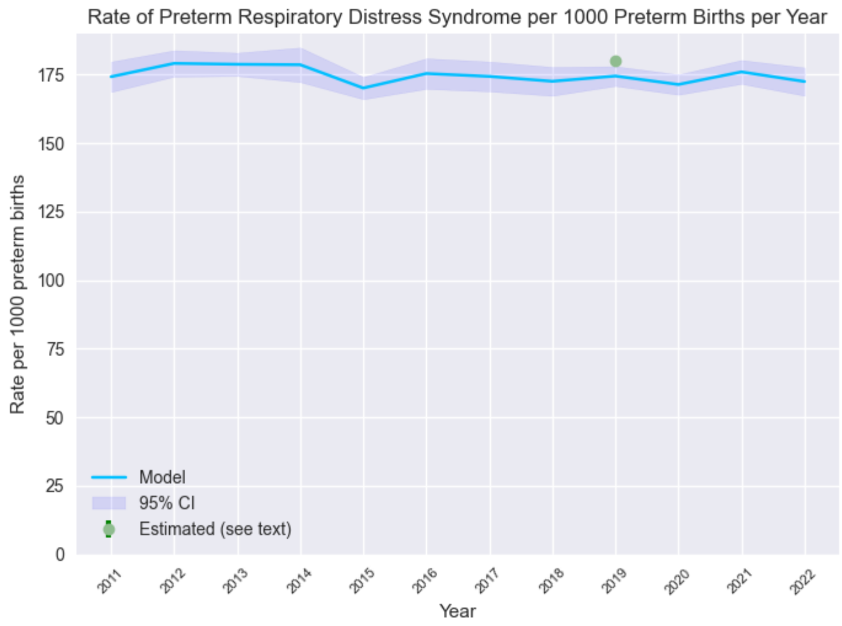

Figure S76 – Preterm respiratory distress syndrome per 1000 preterm births per year in the model
The mean rate of preterm respiratory distress syndrome (95%) in the model per year across 20 simulation runs per year outputted from the model (shown in blue) compared to calibration data (shown in green).

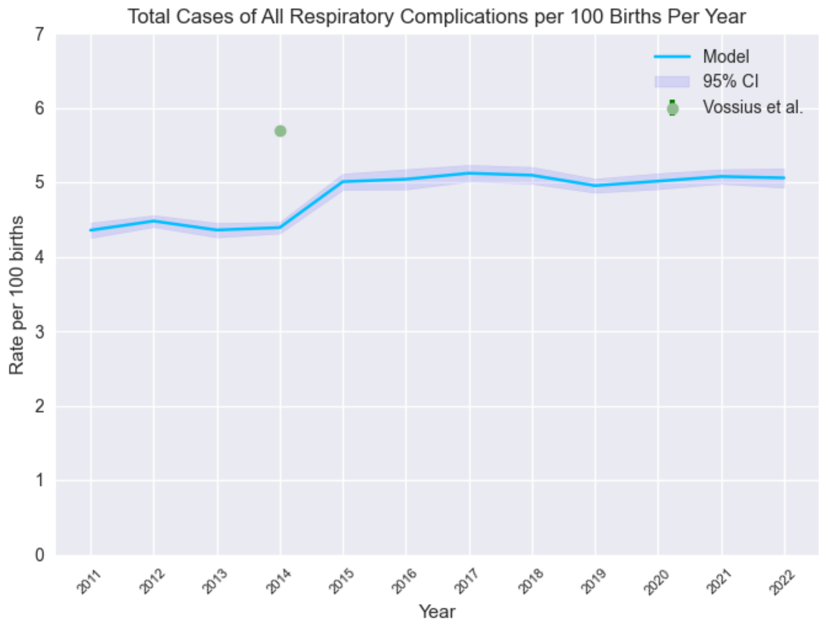

Figure S77 – All respiratory complications per 100 births per year
The mean rate of all respiratory complications (95%) in the model per year across 20 simulation runs per year outputted from the model (shown in blue) compared to calibration data (shown in green).

Here the rate of all respiratory complications, including total cases of neonatal respiratory depression, neonatal encephaloapthy and preterm respiratory distress syndrome, is shown compared to an estimate of the total rate of call cause respiratory distress (thoses infants requiring resuscitation).

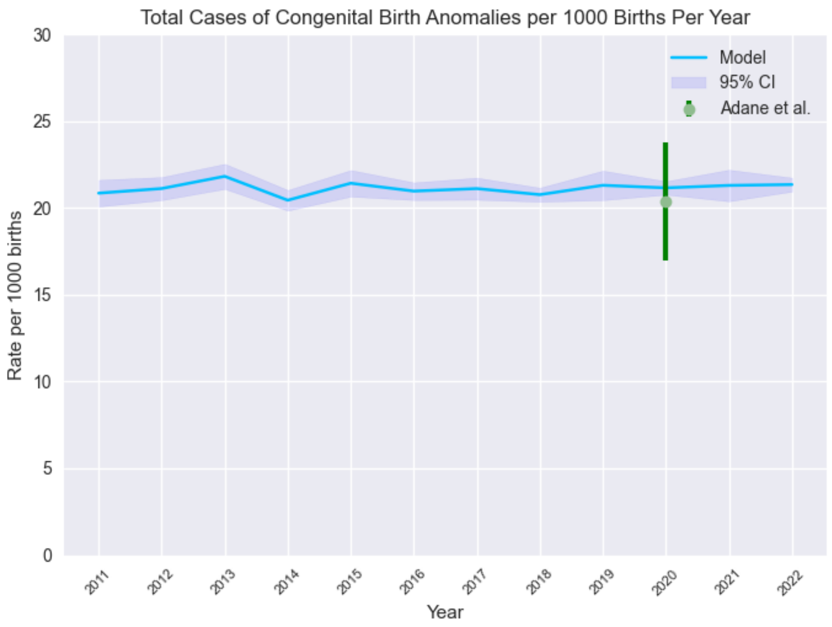

Figure S78 – Congenital birth anomalies per 1000 births per year in the model
The mean rate of congenital birth anomalies (95%) in the model per year across 20 simulation runs per year outputted from the model (shown in blue) compared to calibration data (shown in green).

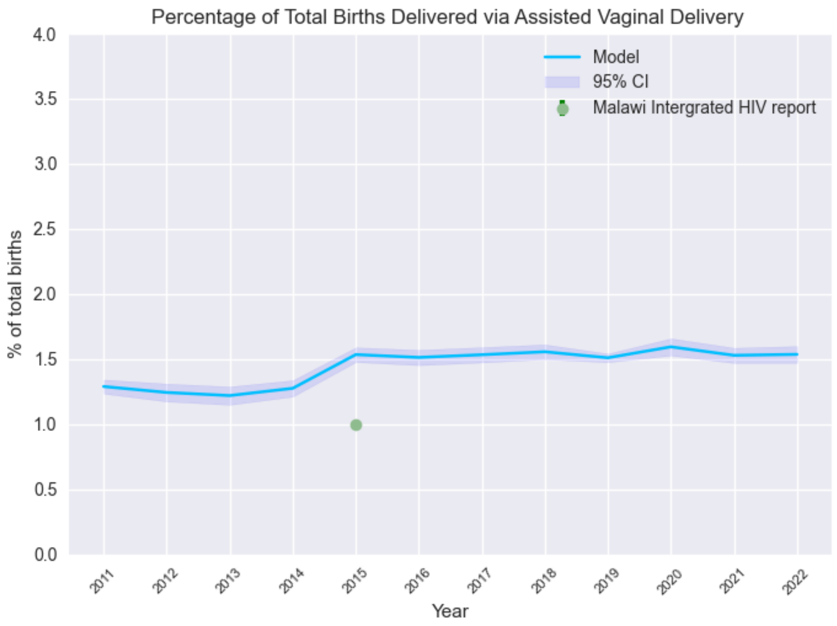

Figure S79 – Percentage of total births delivered by assisted vaginal delivery per year in the model
The mean percentage of deliveries occurring via assisted vaginal delivery (95%) in the model per year across 20 simulation runs per year outputted from the model (shown in blue) compared to calibration data (shown in green).

5 – Additional analyses figures and tables

Tables S61-S62 and Figures S80-S83 present additional findings discussed within in the accompanying manuscript.

Year	Pregnancies (mean (95% CI))	Live births (mean (95% CI))	Stillbirths (mean (95% CI))	Maternal deaths (mean (95% CI))	Neonatal deaths (mean (95% CI))
2010±	**744,259** (741,035, 747,484)	**200,658** (198,841, 202,475)	**4490** (4288, 4692)	**1715** (1595, 1835)	**4467** (4235, 4698)
2011	**731,886** (728,142, 735,630)	**529,544** (527,361, 531,727)	**9919** (9520, 10,318)	**3113** (2905, 3320)	**10,300** (9952, 10,648)
2012	**770,934** (768,069, 773,799)	**554,235** (551,433, 557,038)	**10,460** (10,202, 10,718)	**3199** (2995, 3403)	**10,503** (10155, 10,852)
2013	**800,152** (797,121, 803,184)	**578,778** (575,513, 582,043)	**10,643** (10,214, 11,072)	**3041** (2796, 3286)	**10,922** (10,468, 11,377)
2014	**826,225** (821,741, 830,709)	**599,509** (595,868, 603,149)	**11,006** (10,597, 11,416)	**3314** (3109, 3519)	**11,286** (10,928, 11,644)
2015	**846,071** (842,568, 849,575)	**616,727** (613,143, 620,310)	**10,358** (10,005, 10,711)	**2096** (1953, 2239)	**10,375** (10,046, 10,705)
2016	**863,391** (858,616, 868,166)	**634,279** (631,468, 637,090)	**10,492** (10,172, 10,812)	**2073** (1879, 2267)	**10,626** (10,300, 10,952)

2017	**878,361**<br>(875,084, 881,638)	**647,181**<br>(643,667, 650,696)	**10,623**<br>(10,274, 10,971)	**2236**<br>(2138, 2333)	**11,044**<br>(10,740, 11,348)
2018	**892,261**<br>(888,531, 895,991)	**655,940**<br>(652,481, 659,399)	**10,954**<br>(10,607, 11,302)	**2224**<br>(2002, 2447)	**11,015**<br>(10,664, 11,366)
2019	**899,304**<br>(894,714, 903,894)	**664,795**<br>(661,593, 667,996)	**10,826**<br>(10,462, 11,190)	**2218**<br>(2082, 2353)	**10,716**<br>(10,441, 10,990)
2020	**908,772**<br>(905,604, 911,940)	**670,003**<br>(666,354, 673,651)	**11,120**<br>(10,838, 11,402)	**2219**<br>(2045, 2394)	**11,271**<br>(10,929, 11,614)
2021	**916,815**<br>(912,339, 921,292)	**673,228**<br>(669,919, 676,536)	**11,213**<br>(10,811, 11,615)	**2198**<br>(2022, 2374)	**11,222**<br>(10,833, 11,610)
2022	**928,811**<br>(925,091, 932,530)	**679,317**<br>(675,794, 682,839)	**11,132**<br>(10,697, 11,566)	**2303**<br>(2145, 2460)	**11,370**<br>(10,967, 11,773)
2023	**948,529**<br>(944,730, 952,329)	**690,344**<br>(687,186, 693,501)	**11,559**<br>(11,244, 11,874)	**2386**<br>(2175, 2597)	**11,623**<br>(11,332, 11,914)
2024	**965,497**<br>(962,066, 968,928)	**702,618**<br>(699,055, 706,181)	**11,565**<br>(11,136, 11,993)	**2321**<br>(2159, 2484)	**11,827**<br>(11,468, 12,185)
2025	**989,618**<br>(984,980, 994,256)	**719,914**<br>(717,269, 722,559)	**12,109**<br>(11,763, 12,455)	**2470**<br>(2293, 2647)	**12,213**<br>(11,738, 12,688)
2026	**1,010,765**<br>(1,007,270, 1,014,259)	**729,644**<br>(726,884, 732,404)	**12,178**<br>(11,798, 12,558)	**2391**<br>(2234, 2548)	**12,216**<br>(11,854, 12,579)

2027	**1,028,863** (1,024,646, 1,033,081)	**751,084** (748,355, 753,814)	**12,286** (11,992, 12,580)	**2445** (2282, 2607)	**12,772** (12,373, 13,170)
2028	**1,054,540** (1,050,692, 1,058,389)	**759,500** (755,972, 763,027)	**12,600** (12,133, 13,067)	**2358** (2203, 2513)	**13,121** (12,637, 13,604)
2029	**1,065,893** (1,063,127, 1,068,659)	**775,950** (772,793, 779,107)	**13,211** (12,933, 13,489)	**2470** (2295, 2644)	**12,998** (12,647, 13,349)
2030	**1,082,715** (1,078,941, 1,086,490)	**787,166** (784,911, 789,420)	**12,850** (12,461, 13,239)	**2701** (2500, 2901)	**13,132** (12,749, 13,516)
Values are scaled to correspond to the real size of the population of Malawi (e.g. values from the model are multiplied by a scaling factor calculated as (1 / (modelled population size in 2010 / estimated population size in Malawi in 2010 from World Population Prospects 2019))<br><br>*±As the simulation is initialised on 01/01/2010, 2010 acts a 'burn-in' period for the simulation where outputs from the model have yet to stabilise. This is evident when reviewing the number of births and deaths for this year because no women are modelled as being pregnant at the initialisation of the simulation*					

Table S62- Yearly pregnancies, births, stillbirths, maternal deaths and neonatal deaths during the status quo scenario 2010-2030

Scenario (short name)	Total maternal and neonatal DALYs averted 2023-2030 (mean)	Maximum ability to pay (USD)	% of projected total national health spending 2023-2030[±]
AN coverage	424,940	$ 26,473,762	0.26%
AN coverage and qual	839,929	$ 52,327,577	0.52%
AN max.	906,456	$ 56,472,209	0.56%
IP BEmONC	1,676,275	$ 104,431,933	1.04%
IP CEmONC	1,697,125	$ 105,730,888	1.05%
IP max.	2,181,368	$ 135,899,226	1.35%
PN coverage and qual	757,982	$ 47,222,279	0.47%
PN max.	1,022,957	$ 63,730,221	0.63%
All services coverage	2,035,389	$ 126,804,735	1.26%
All services coverage and qual.	2,898,963	$ 180,605,395	1.80%
All services max.	3,633,832	$ 226,387,734	2.25%
[±]Projected total national health spending between 2023 and 2030 calculated as $10,058,089,622. See methods section of accompanying manuscript for details on how this value was calculated.			

Table S63- Maximum ability to pay for modelled scenarios

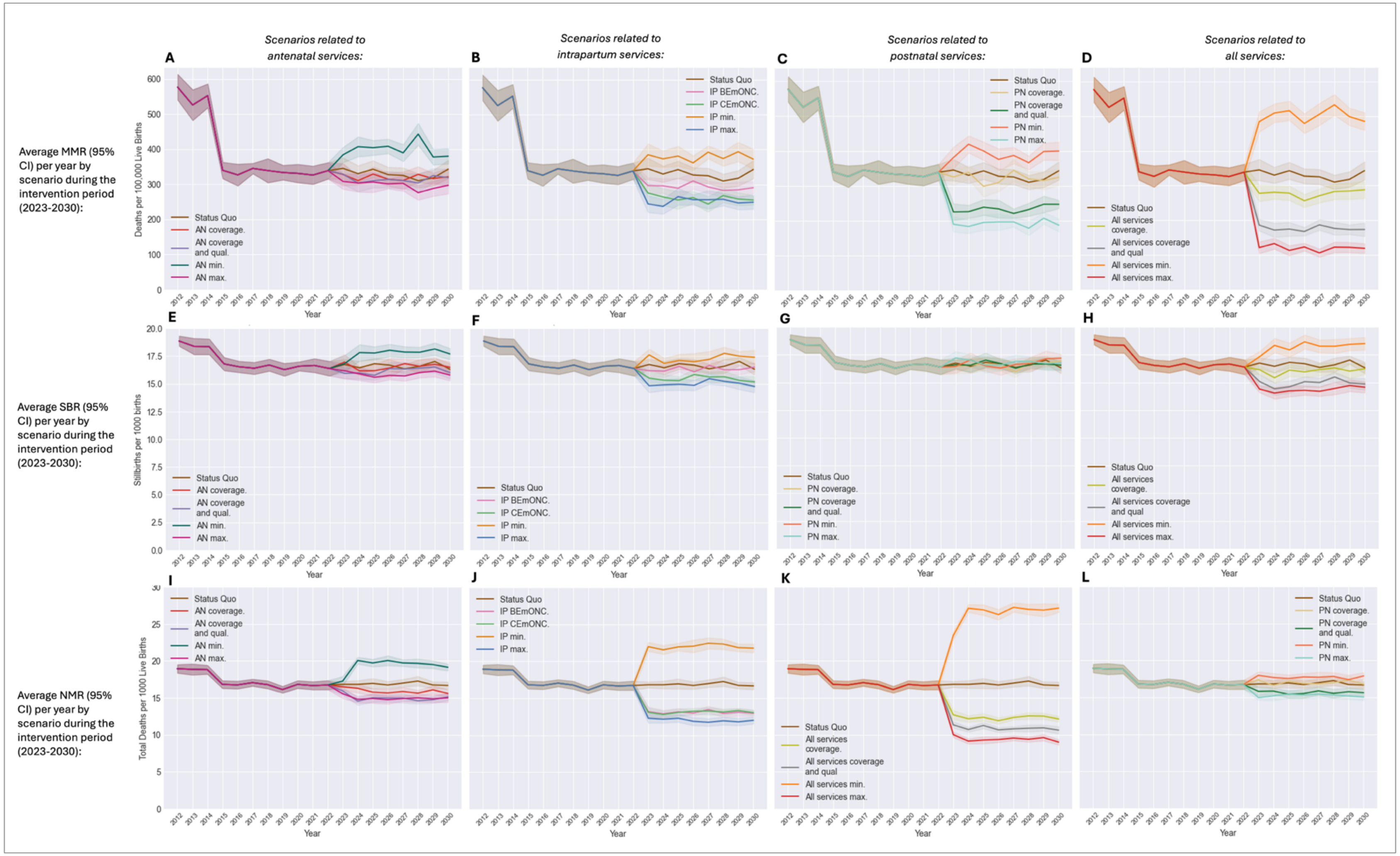

Figure S80 – Yearly MMR, SBR, NMR by scenario organised by scenarios relating to antenatal, intrapartum, and postnatal service delivery
(A-D) The average yearly MMR across the model period (2011-2030) and 95% CI for all scenarios. (E-H) The average yearly SBR across the model period (2011-2030) and 95% CI for all scenarios. (I-L) The average yearly NMR across the model period (2011-2030) and 95% CI for all scenarios.

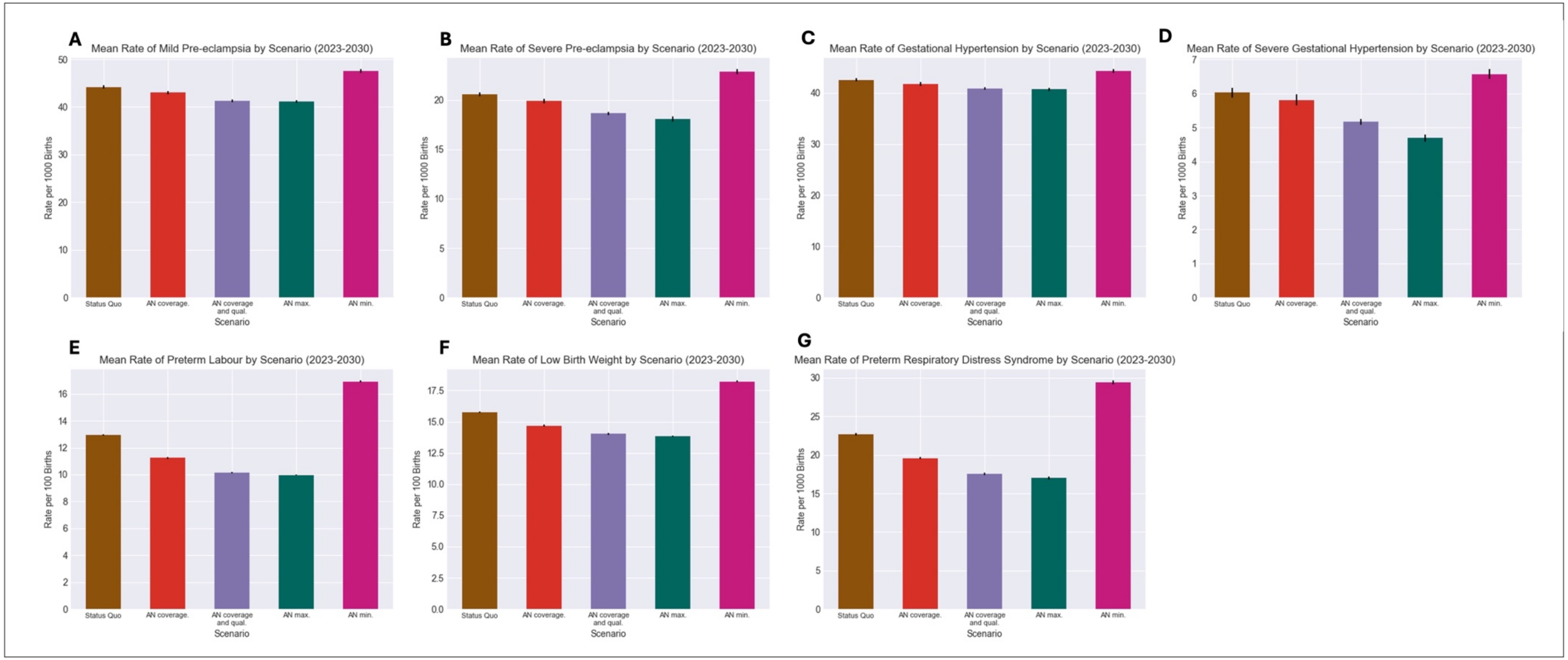

Figure S81 – Average rate or prevalence of selected complications during the intervention period within scenarios relating to delivery of antenatal services

(A) Mean rate of pre-eclampsia (cases per 1000 births) by modelled antenatal care scenarios during 2023-2030. (B) Mean rate of severe pre-eclampsia (cases per 1000 births) by modelled antenatal care scenarios during 2023-2030. (C) Mean rate of gestational hypertension (cases per 1000 births) by modelled antenatal care scenarios during 2023-2030. (D) Mean rate of severe gestational hypertension (cases per 1000 births) by modelled antenatal care scenarios during 2023-2030. (E) Mean rate of preterm labour (cases per 100 births) by modelled antenatal care scenarios during 2023-2030. (F) Mean rate of low birth weight (cases per 100 births) by modelled antenatal care scenarios during 2023-2030. (G) Mean preterm respiratory distress syndrome (cases per 1000 births) by modelled antenatal care scenarios during 2023-2030.

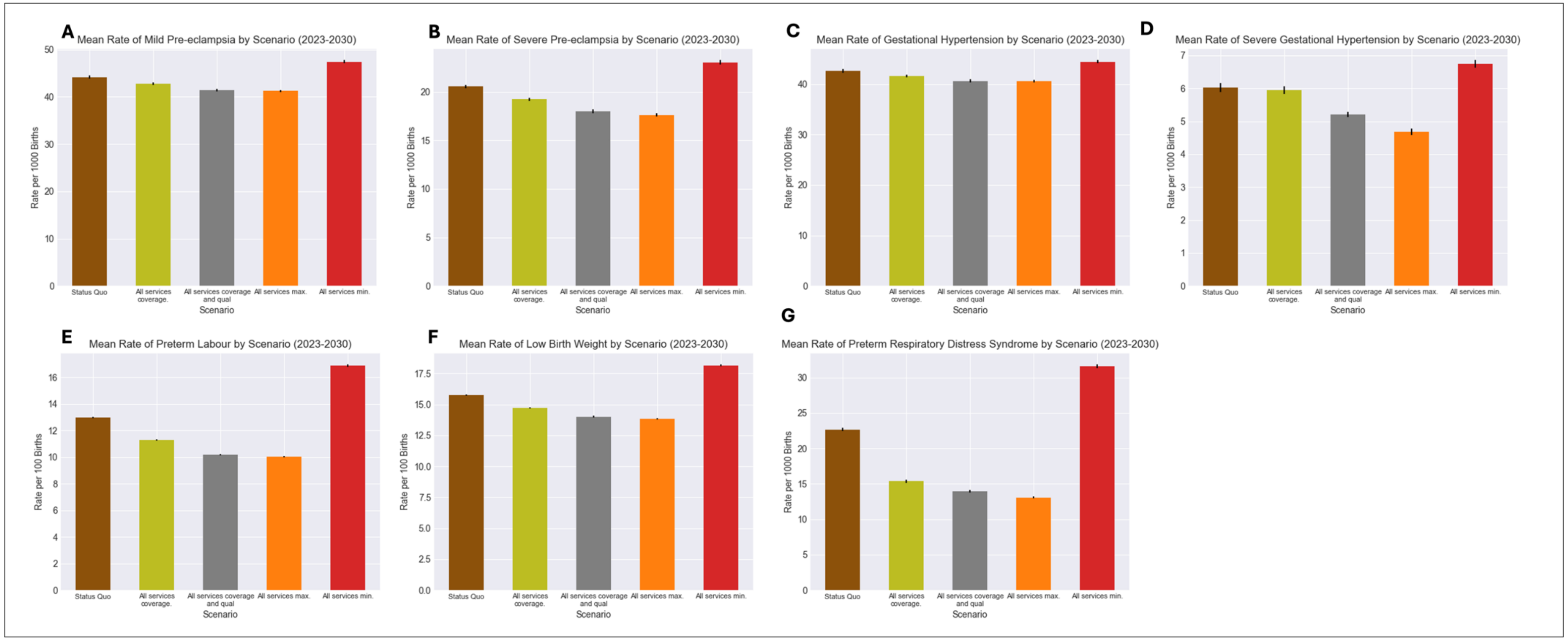

Figure S82 – Average rate or prevalence of selected complications during the intervention period within scenarios relating to delivery of all services
(A) Mean rate of pre-eclampsia (cases per 1000 births) by scenarios relating to delivery of all services during 2023-2030. (B) Mean rate of severe pre-eclampsia (cases per 1000 births) by modelled scenarios relating to delivery of all services during 2023-2030. (C) Mean rate of gestational hypertension (cases per 1000 births) by modelled scenarios relating to delivery of all services during 2023-2030. (D) Mean rate of severe gestational hypertension (cases per 1000 births) by modelled scenarios relating to delivery of all services during 2023-2030. (E) Mean rate of preterm labour (cases per 100 births) by modelled scenarios relating to delivery of all services during 2023-2030. (F) Mean rate of low birth weight (cases per 100 births) by modelled scenarios relating to delivery of all services during 2023-2030. (G) Mean preterm respiratory distress syndrome (cases per 1000 births) by modelled antenatal care scenarios during 2023-2030.

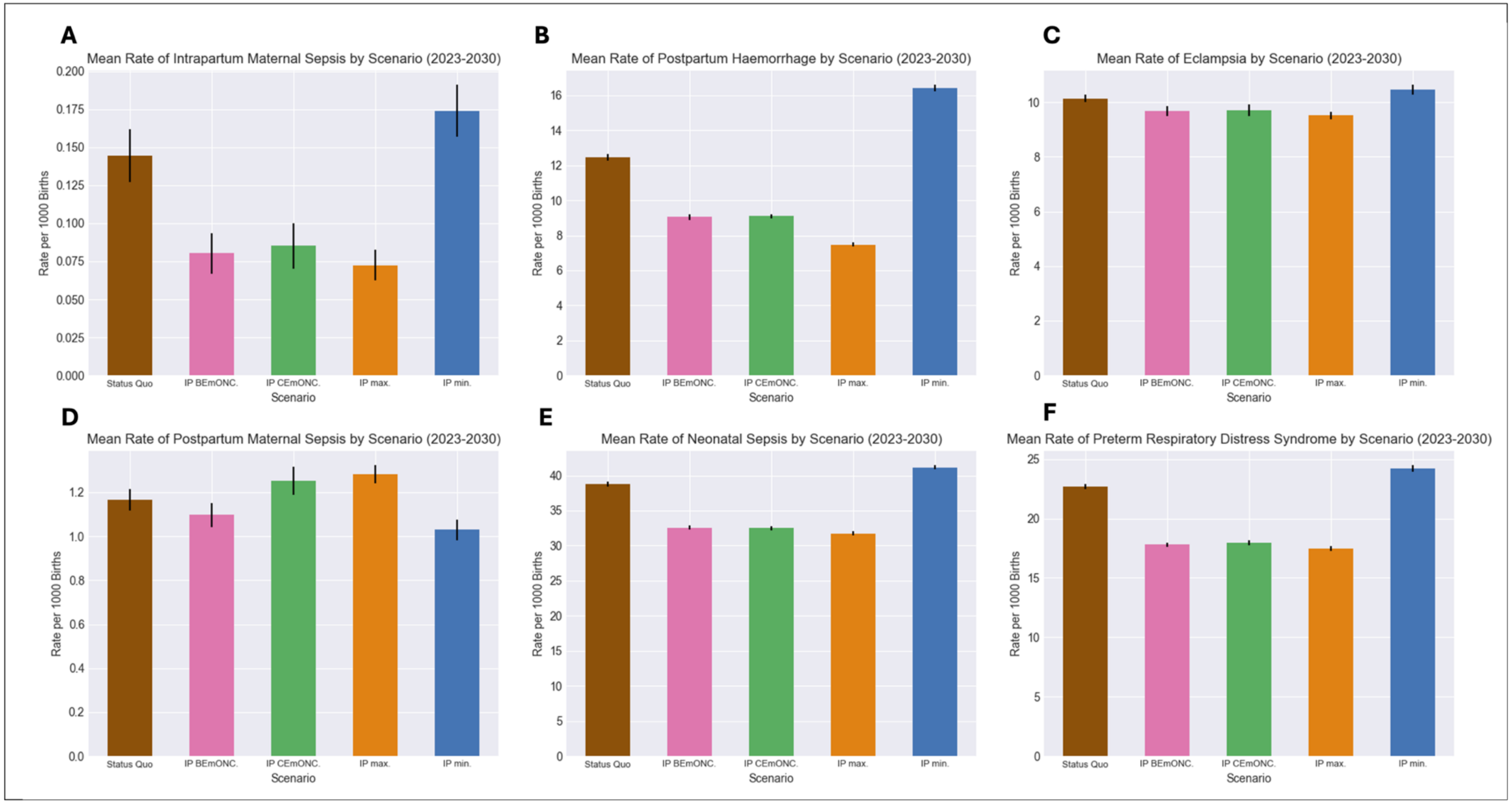

Figure S83 – Average rate or prevalence of selected complications during the intervention period within scenarios relating to delivery of intrapartum services

(A) Mean rate of intrapartum maternal sepsis (cases per 1000 births) by modelled intrapartum care scenarios during 2023-2030. (B) Mean rate of postpartum haemorrhage (cases per 1000 births) by modelled intrapartum care scenarios during 2023-2030. (C) Mean rate of eclampsia (cases per 1000 births) by modelled intrapartum care scenarios during 2023-2030. (D) Mean rate of postpartum maternal sepsis (cases per 1000 births) by modelled intrapartum care scenarios during 2023-2030. (E) Mean rate of neonatal sepsis (cases per 1000 births) by modelled intrapartum care scenarios during 2023-2030. (F) Mean rate of preterm respiratory distress syndrome (cases per 1000 births) by modelled intrapartum care scenarios during 2023-2030.

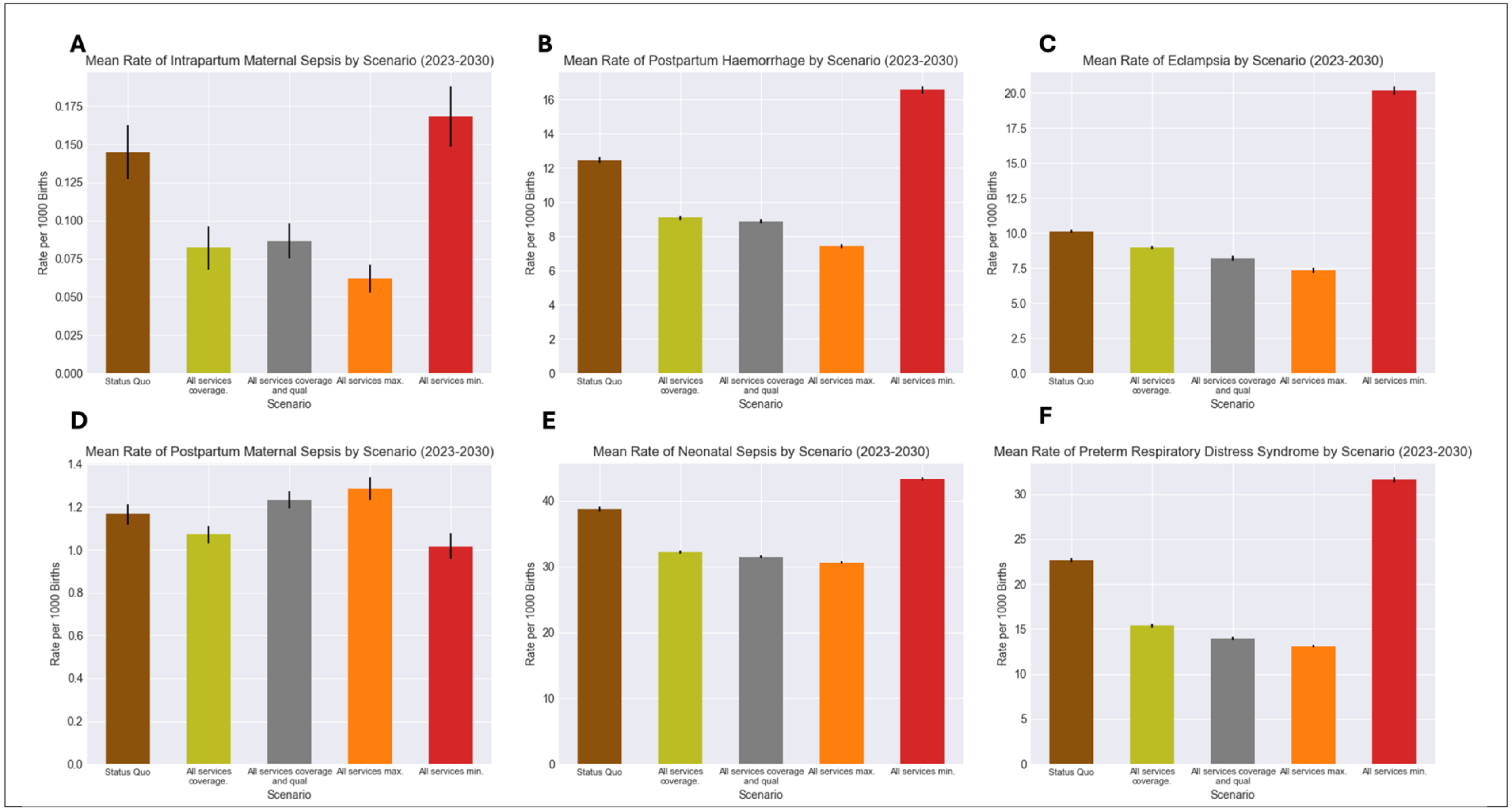

Figure S84 – Average rate or prevalence of selected complications during the intervention period within scenarios relating to delivery of all services
(A) Mean rate of intrapartum maternal sepsis (cases per 1000 births) by scenarios relating to delivery of all services during 2023-2030. (B) Mean rate of postpartum haemorrhage (cases per 1000 births) by modelled scenarios relating to delivery of all services during 2023-2030. (C) Mean rate of eclampsia (cases per 1000 births) by modelled scenarios relating to delivery of all services during 2023-2030. (D) Mean rate of postpartum maternal sepsis (cases per 1000 births) by modelled scenarios relating to delivery of all services during 2023-2030. (E) Mean rate of neonatal sepsis (cases per 1000 births) by modelled scenarios relating to delivery of all services during 2023-2030. (F) Mean rate of preterm respiratory distress syndrome (cases per 1000 births) by modelled scenarios relating to delivery of all services during 2023-2030.

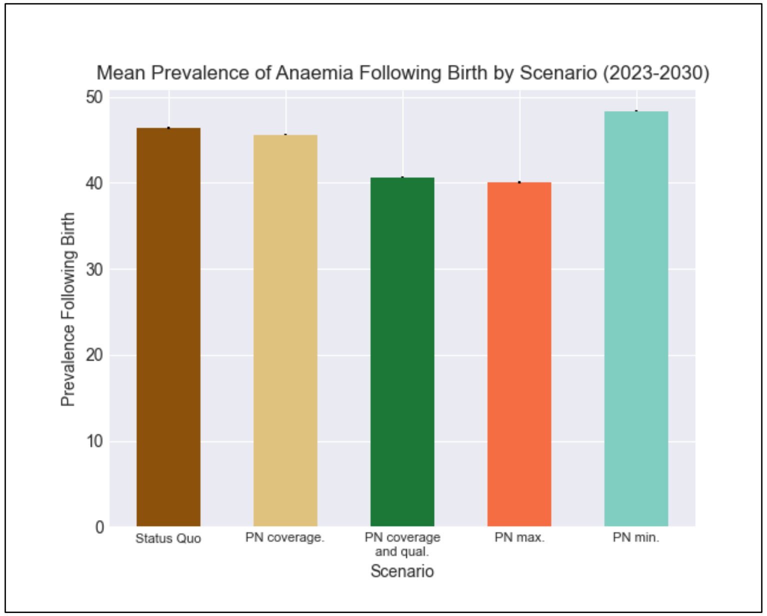

Figure S85 – Average prevalence of maternal anaemia at the end of the postnatal period within scenarios relating to delivery of postnatal services
Mean prevalence of maternal anaemia at six-weeks postnatal across scenarios relating to the delivery of postnatal services

Supplementary references

1. Abbafati C, Abbas KM, Abbasi M, Abbasifard M, Abbasi-Kangevari M, Abbastabar H, et al. Global burden of 369 diseases and injuries in 204 countries and territories, 1990-2019: a systematic analysis for the Global Burden of Disease Study 2019. LANCET. 2020;396(10258):1204–22.

2. Khaki JJ, Sithole L. Factors associated with the utilization of postnatal care services among Malawian women. Malawi Med J [Internet]. 2019;31(1):2–11. Available from: https://pubmed.ncbi.nlm.nih.gov/31143390/

3. Allott H. Consultant Obstetrician. Personal Communication. 9th April 2021.

4. Monden CWS, Smits J. Mortality among twins and singletons in sub-Saharan Africa between 1995 and 2014: a pooled analysis of data from 90 Demographic and Health Surveys in 30 countries. Lancet Glob Health [Internet]. 2017;5(7):e673–9. Available from: http://www.thelancet.com/article/S2214109X17301973/fulltext

5. Pietravalle A, Spolverato S, Brasili L, Cavallin F, Gabrielli V, Azzimonti G, et al. Comparison of alternative gestational age assessment methods in a low resource setting: a retrospective study. BMC Pregnancy Childbirth [Internet]. 2022;22(1):1–5. Available from: https://pubmed.ncbi.nlm.nih.gov/35869463/

6. Kalanda BF, van Buuren S, Verhoeff FH, Brabin BJ. Anthropometry of Malawian live births between 35 and 41 weeks of gestation. Ann Hum Biol [Internet]. 2005;32(5):639–49. Available from: https://pubmed.ncbi.nlm.nih.gov/16316919/

7. Boghossian NS, Geraci M, Edwards EM, Morrow KA, Horbar JD. Anthropometric charts for infants born between 22 and 29 weeks' gestation. Pediatrics [Internet]. 2016;138(6). Available from: https://pubmed.ncbi.nlm.nih.gov/27940694/

8. Cutland CL, Lackritz EM, Mallett-Moore T, Bardají A, Chandrasekaran R, Lahariya C, et al. Low birth weight: Case definition & guidelines for data collection, analysis, and presentation of maternal immunization safety data. Vaccine [Internet]. 2017;35(48Part A):6492. Available from: https://www.ncbi.nlm.nih.gov/pmc/articles/PMC5710991/

9. Royal College of Obstetricians & Gynaecologists. Small-for-Gestational-Age Fetus, Investigation and Management (Green-top Guideline No. 31) [Internet]. 2013. Available from: https://www.rcog.org.uk/guidance/browse-all-guidance/green-top-guidelines/small-for-gestational-age-fetus-investigation-and-management-green-top-guideline-no-31/

10. Ngwira A. Spatial quantile regression with application to high and low child birth weight in Malawi. BMC Public Health [Internet]. 2019;19(1):1593. Available from: https://doi.org/10.1186/s12889-019-7949-9

11. National Statistical Office - NSO/Malawi and ICF. Malawi Demographic and Health Survey 2015-16 [Internet]. 2017;Available from: https://dhsprogram.com/publications/publication-FR319-DHS-Final-Reports.cfm

12. National Statistical Office - NSO/Malawi and ICF. Malawi Demographic and Health Survey 2010 [Internet]. 2011. Available from: https://dhsprogram.com/publications/publication-fr247-dhs-final-reports.cfm

13. World Health Organization, UNICEF. Capture the moment: early initiation of breastfeeding : the best start for every newborn [Internet]. 2018. Available from: https://apps.who.int/nutrition/publications/infantfeeding/capture-moment-early-initiation-bf/en/index.html#:~:text=The%20World%20Health%20Organization%20(WHO,liquids%20are%20provided%2C%20including%20water.

14. Global Burden of Disease Collaborative Network. Institute for Health Metrics and Evaluation (IHME). 2020. Global Burden of Disease Study 2019 (GBD 2019) Disability Weights. Available from: https://ghdx.healthdata.org/record/ihme-data/gbd-2019-disability-weights

15. Blencowe H, Lee ACC, Cousens S, Bahalim A, Narwal R, Zhong N, et al. Preterm birth-associated neurodevelopmental impairment estimates at regional and global levels for 2010. Pediatr Res [Internet]. 2013;74 Suppl 1(Suppl 1):17–34. Available from: https://pubmed.ncbi.nlm.nih.gov/24366461/

16. Blencowe H, Lawn JE, Vazquez T, Fielder A, Gilbert C. Preterm-associated visual impairment and estimates of retinopathy of prematurity at regional and global levels for 2010. Pediatr Res [Internet]. 2013;74 Suppl 1(Suppl 1):35–49. Available from: https://pubmed.ncbi.nlm.nih.gov/24366462/

17. Lee AC, Kozuki N, Blencowe H, Vos T, Bahalim A, Darmstadt GL, et al. Intrapartum-related neonatal encephalopathy incidence and impairment at regional and global levels for 2010 with trends from 1990. Pediatr Res [Internet]. 2013;74(S1):50–72. Available from: http://www.nature.com/articles/pr2013206

18. Seale AC, Blencowe H, Zaidi A, Ganatra H, Syed S, Engmann C, et al. Neonatal severe bacterial infection impairment estimates in South Asia, sub-Saharan Africa, and Latin America for 2010. Pediatr Res. [Internet]. 2013;74(Suppl. 1):73–85. Available from: http://www.nature.com/pr/journal/v74/n1s/full/pr2013207a.html

19. Malawi Ministry of Health and Population. Malawi Human Resources for Health Strategic Plan, 2018-22. 2018.

20. Thaddeus S, Maine D. Too far to walk: maternal mortality in context. Soc Sci Med [Internet]. 1994;38(8):1091–110. Available from: http://www.ncbi.nlm.nih.gov/pubmed/8042057

21. Vink NM, de Jonge HCC, Ter Haar R, Chizimba EM, Stekelenburg J. Maternal death reviews at a rural hospital in Malawi. Int J GynecolObstet. 2013;120(1):74–7. Available from: https://pubmed.ncbi.nlm.nih.gov/23182799/

22. Mgawadere F, Unkels R, Kazembe A, van den Broek N. Factors associated with maternal mortality in Malawi: Application of the three delays model. BMC Pregnancy Childbirth [Internet]. 2017;17(1):1–9. Available from: https://bmcpregnancychildbirth.biomedcentral.com/articles/10.1186/s12884-017-1406-5

23. Tiruneh GA, Arega DT, Kassa BG, Bishaw KA. Delay in making decision to seek care on institutional delivery and associated factors among postpartum mothers in South Gondar zone hospitals, 2020: A cross-sectional study. Heliyon. 2022;8(3):e09056. Available from: https://www.ncbi.nlm.nih.gov/pmc/articles/PMC8908018/#:~:text=Conclusion,care%20in%20South%20Gondar%20zone.

24. Combs Thorsen V, Sundby J, Malata A, Thorsen VC, Sundby J, Malata A. Piecing Together the Maternal Death Puzzle through Narratives: The Three Delays Model Revisited. PLoS One [Internet]. 2012;7(12). Available from: https://journals.plos.org/plosone/article?id=10.1371/journal.pone.0052090

25. Changole J, Combs Thorsen V, Kafulafula U. A road to obstetric fistula in Malawi: capturing women's perspectives through a framework of three delays. Int J Womens Health [Internet]. 2018;10:699–713. Available from: https://pubmed.ncbi.nlm.nih.gov/30464646/

26. Okafor O, Roos N, Abdosh AA, Adesina O, Alaoui Z, Romero WA, et al. International virtual confidential reviews of infection-related maternal deaths and near-miss in 11 low- and middle-income countries - case report series and suggested actions. BMC Pregnancy Childbirth [Internet]. 2022;22(1):431. Available from: https://pubmed.ncbi.nlm.nih.gov/35606709/

27. Kasahun AW, Wako WG. Predictors of maternal near miss among women admitted in Gurage zone hospitals, South Ethiopia, 2017: a case control study. BMC Pregnancy Childbirth [Internet]. 2018;18(1). Available from: https://pubmed.ncbi.nlm.nih.gov/29940889/

28. Kumela L, Tilahun T, Kifle D. Determinants of Maternal Near Miss in Western Ethiopia. Ethiop J Health Sci [Internet]. 2020;30(2):161–8. Available from: https://pubmed.ncbi.nlm.nih.gov/32165805/

29. Habtei A, Wondimu M. Determinants of maternal near miss among women admitted to maternity wards of tertiary hospitals in Southern Ethiopia, 2020: A hospital-based case-control study. PLoS One [Internet]. 2021;16(5):e0251826. Available from: https://journals.plos.org/plosone/article?id=10.1371/journal.pone.0251826

30. Woldeyes WS, Asefa D, Muleta G. Incidence and determinants of severe maternal outcome in Jimma University teaching hospital, south-West Ethiopia: a prospective cross-sectional study.

BMC Pregnancy Childbirth [Internet]. 2018;18(1). Available from: https://pubmed.ncbi.nlm.nih.gov/29925329/

31. Malawi Ministry of Health and Population. Malawi Standard Treatment Guidelines (MSTG) 5th Edition [Internet]. 2015. Available from: https://extranet.who.int/ncdccs/Data/MWI_D1_Malawi-Standard-Treatment-Guidelines-Essential-Medicines-List-2015.pdf

32. The Association of Obstetricians & Gynaecologists of Malawi. Obstetrics & Gynaecology Protocols and Guidelines. 2014.

33. Malawi Ministry of Health and Population, USAID, UNFPA, UNICEF, CHAI, Save the Children. Malawi Emergency Obstetric and Newborn Care Needs Assessment, 2014 [Internet]. 2015. Available from: https://www.healthynewbornnetwork.org/hnn-content/uploads/Malawi-EmONC-Report-June-2015_FINAL.pdf

34. Malawi Ministry of Health and Population, UNICEF, UNFPA, WHO, AMDD. Malawi 2010 EmONC Needs Assessment Final Report. 2010.

35. Arsenault C, English M, Gathara D, Malata A, Mandala W, Kruk ME. Variation in competent and respectful delivery care in Kenya and Malawi: a retrospective analysis of national facility surveys. Trop Med Int Health [Internet]. 2020;25(4):442–53. Available from: https://pubmed.ncbi.nlm.nih.gov/31828923/

36. Chinkhumba J, De Allegri M, Mazalale J, Brenner S, Mathanga D, Muula AS, et al. Household costs and time to seek care for pregnancy related complications: The role of results-based financing. PLoS One [Internet]. 2017 [cited 2022 Nov 10];12(9):e0182326. Available from: https://journals.plos.org/plosone/article?id=10.1371/journal.pone.0182326

37. Pollard SL, Mathai M, Walker N. Estimating the impact of interventions on cause-specific maternal mortality: A Delphi approach. BMC Public Health [Internet]. 2013;13. Available from: https://bmcpublichealth.biomedcentral.com/articles/10.1186/1471-2458-13-S3-S12#:~:text=Results,having%20effectiveness%20estimates%20above%2070%25.

38. Malawi Ministry of Health and Population Antenatal Care Matrix. Unk.

39. World Health Organization. WHO recommendations on antenatal care for a positive pregnancy experience [Internet]. 2016. Available from: WHO recommendations on antenatal care for a positive pregnancy experience

40. Ng'ambi WF, Collins JH, Colbourn T, Mangal T, Phillips A, Kachale F, et al. Socio-demographic factors associated with early antenatal care visits among pregnant women in Malawi: 2004–2016. PLoS One [Internet]. 2022;17(2):e0263650. Available from: https://journals.plos.org/plosone/article?id=10.1371/journal.pone.0263650

41. McHenga M, Burger R, Von Fintel D. Examining the impact of WHO's Focused Antenatal Care policy on early access, underutilisation and quality of antenatal care services in Malawi: A retrospective study. BMC Health Serv Res [Internet]. 2019;19(1):1–14. Available from: https://bmchealthservres.biomedcentral.com/articles/10.1186/s12913-019-4130-1

42. Peña-Rosas JP, De-Regil LM, Garcia-Casal MN, Dowswell T. Daily oral iron supplementation during pregnancy. Cochrane Database of Syst. Rev [Internet]. 2015; (7):CD004736. Available from: https://www.cochranelibrary.com/cdsr/doi/10.1002/14651858.CD004736.pub5/full

43. Ota E, Hori H, Mori R, Tobe-Gai R, Farrar D. Antenatal dietary education and supplementation to increase energy and protein intake. Cochrane Database Syst Rev [Internet]. 2015;(6):CD000032. Available from: https://www.cochranelibrary.com/cdsr/doi/10.1002/14651858.CD000032.pub3/full

44. Hofmeyr GJ, Lawrie TA, Atallah ÁN, Torloni MR. Calcium supplementation during pregnancy for preventing hypertensive disorders and related problems. Cochrane Database of Syst Rev. [Internet]. 2018; 10(10):CD001059. Available from: https://www.cochranelibrary.com/cdsr/doi/10.1002/14651858.CD001059.pub5/full

45. Malawi Ministry of Health and Population, ICF International. Malawi Service Provision Assessment (SPA) 2013-14 [Internet]. 2014. Available from: https://dhsprogram.com/pubs/pdf/SPA20/SPA20%5BOct-7-2015%5D.pdf

46. Malawi Ministry of Health and Population. Malawi Harmonised Health Facility Assessment (HHFA) 2018/2019 Report. 2019.

47. The Government of the Republic of Malawi. 2012 Global AIDS Response Progress Report: Malawi Country Report for 2010 and 2011 [Internet]. 2012. Available from: https://www.unaids.org/sites/default/files/country/documents/ce_MW_Narrative_Report%5b1%5d.pdf

48. The Government of the Republic of Malawi. Malawi AIDS Response Progress Report 2015 [Internet]. 2015. Available from: http://catalogue.safaids.net/sites/default/files/publications/Malawi%20AIDS%20Response%20Progress%20Report%202015.pdf

49. Karnjanapiboonwong A, Anothaisintawee T, Chaikledkaew U, Dejthevaporn C, Attia J, Thakkinstian A. Diagnostic performance of clinic and home blood pressure measurements compared with ambulatory blood pressure: a systematic review and meta-analysis. BMC Cardiovasc Disord [Internet]. 2020;20(1):1–17. Available from: https://doi.org/10.1186/s12872-020-01736-2

50. Gangaram R, Ojwang PJ, Moodley J, Maharaj D. The accuracy of urine dipsticks as a screening test for proteinuria in hypertensive disorders of pregnancy. Hypertens Pregnancy [Internet]. 2005;24(2):117–23. Available from: https://pubmed.ncbi.nlm.nih.gov/16036396/

51. Abebe J, Eigbefoh J, Isabu P, Okogbenin S, Eifediyi R, Okusanya B. Accuracy of urine dipsticks, 2-h and 12-h urine collections for protein measurement as compared with the 24-h collection. J Obstet Gynaecol [Internet]. 2008;28(5):496–500. Available from: https://pubmed.ncbi.nlm.nih.gov/18850422/

52. Van Den Broek NR, Ntonya C, Mhango E, White SA. Diagnosing anaemia in pregnancy in rural clinics: assessing the potential of the Haemoglobin Colour Scale. Bull World Health Organ [Internet]. 1999;77(1):15. Available from: https://pubmed.ncbi.nlm.nih.gov/10063656/

53. Bristow CC, Larson E, Anderson LJ, Klausner JD. Cost-effectiveness of HIV and syphilis antenatal screening: a modeling study. Sex Transm Infect [Internet]. 2016;92(5):340. Available from: https://www.ncbi.nlm.nih.gov/pmc/articles/PMC4956558/

54. Allott H. Consultant Obstetrician. Personal Communication. 23rd October 2020.

55. Balsells M, García-Patterson A, Solà I, Roqué M, Gich I, Corcoy R. Glibenclamide, metformin, and insulin for the treatment of gestational diabetes: a systematic review and meta-analysis. BMJ [Internet]. 2015;350. Available from: https://www.bmj.com/content/350/bmj.h102

56. Abalos E, Duley L, Steyn DW, Gialdini C. Antihypertensive drug therapy for mild to moderate hypertension during pregnancy. Cochrane Database Syst Rev [Internet]. 2018; 10(10):CD002252. Available from: https://pubmed.ncbi.nlm.nih.gov/30277556/

57. Duley L, Gülmezoglu A, Henderson-Smart D, Chou J. Magnesium sulphate and other anticonvulsants for women with pre-eclampsia. Cochrane Database Syst Rev [Internet]. 2010;2010(11):1269–70. Available from: https://pubmed.ncbi.nlm.nih.gov/21069663/

58. Cousens S, Blencowe H, Gravett M, Lawn JE. Antibiotics for pre-term pre-labour rupture of membranes: Prevention of neonatal deaths due to complications of pre-term birth and infection. Int J Epidemiol [Internet]. 2010;39(Suppl.1):i134. Available from: https://www.ncbi.nlm.nih.gov/pmc/articles/PMC2845869/#:~:text=Thus%2C%20we%20estimate%20that%20giving,due%20to%20sepsis%20by%208%25.

59. Kenyon S, Boulvain M, Neilson JP. Antibiotics for preterm rupture of membranes. Cochrane Database Syst Rev [Internet]. 2013;2013(12). Available from: https://www.cochranelibrary.com/cdsr/doi/10.1002/14651858.CD001058.pub3/full

60. Government of the Republic of Malawi. Health Sector Strategic Plan II (2017-2022) [Internet]. 2017. Available from: https://www.healthdatacollaborative.org/fileadmin/uploads/hdc/Documents/Country_documents/HSSP_II_Final_HQ_complete_file.pdf.pdf

61. Blencowe H, Cousens S, Mullany LC, Lee AC, Kerber K, Wall S, et al. Clean birth and postnatal care practices to reduce neonatal deaths from sepsis and tetanus: a systematic review and Delphi estimation of mortality effect. BMC Public Health [Internet]. 2011;11(Suppl 3):S11. Available from: https://bmcpublichealth.biomedcentral.com/articles/10.1186/1471-2458-11-S3-S11#:~:text=The%20panel%20judged%20that%20clean,both%20sepsis%20and%20tetanus%20deaths.

62. McGoldrick E, Stewart F, Parker R, Dalziel SR. Antenatal corticosteroids for accelerating fetal lung maturation for women at risk of preterm birth. Cochrane Database Syst Rev [Internet]. 2020;2021(2). Available from: https://pubmed.ncbi.nlm.nih.gov/33368142/

63. Begley CM, Gyte GML, Devane D, McGuire W, Weeks A, Biesty LM. Active versus expectant management for women in the third stage of labour. Cochrane Database of Syst Rev [Internet]. 2019;2019(2). Available from: https://www.cochranelibrary.com/cdsr/doi/10.1002/14651858.CD007412.pub5/full

64. Lee ACC, Cousens S, Darmstadt GL, Blencowe H, Pattinson R, Moran NF, et al. Care during labor and birth for the prevention of intrapartum-related neonatal deaths: a systematic review and Delphi estimation of mortality effect. BMC Public Health [Internet]. 2011;11 Suppl 3(Suppl 3):S10. Available from: https://bmcpublichealth.biomedcentral.com/articles/10.1186/1471-2458-11-S3-S10

65. Malawi Ministry of Health and Population. Integrated HIV Program Report January -March 2015. 2015.

66. The Government of the Republic of Malawi. Malawi National Reproductive Health Service Delivery Guidelines. 2014.

67. World Health Organization. WHO recommendations on maternal and newborn care for a positive postnatal experience [Internet]. 2022. Available from: https://www.who.int/publications/i/item/9789240045989

68. Sagawa J, Kabagenyi A, Turyasingura G, Mwale SE. Determinants of postnatal care service utilization among mothers of Mangochi district, Malawi: a community-based cross-sectional study. BMC Pregnancy Childbirth [Internet]. 2021;21(1):1–11. Available from: https://link.springer.com/articles/10.1186/s12884-021-04061-4

69. Chaka EE, Abdurahman AA, Nedjat S, Majdzadeh R. Utilization and Determinants of Postnatal Care Services in Ethiopia: A Systematic Review and Meta-Analysis. Ethiop J Health Sci [Internet]. 2019;29(1):935. Available from: https://www.ncbi.nlm.nih.gov/pmc/articles/PMC6341430/

70. Dol J, Hughes B, Bonet M, Dorey R, Dorling J, Grant A, et al. Timing of maternal mortality and severe morbidity during the postpartum period: a systematic review. JBI Evid Synth [Internet]. 2022;20(9):2119. Available from: https://pubmed.ncbi.nlm.nih.gov/35916004/

71. Gallos ID, Papadopoulou A, Man R, Athanasopoulos N, Tobias A, Price MJ, et al. Uterotonic agents for preventing postpartum haemorrhage: A network meta-analysis. Cochrane Database Syst Rev. [Internet]. 2018;4(4):CD011689. Available from: https://pubmed.ncbi.nlm.nih.gov/29693726/

72. Lawn JE, Mwansa-Kambafwile J, Horta BL, Barros FC, Cousens S. 'Kangaroo mother care' to prevent neonatal deaths due to preterm birth complications. Int J Epidemiol [Internet]. 2010;39 Suppl 1(Suppl 1). Available from: https://pubmed.ncbi.nlm.nih.gov/20348117/

73. Zaidi AKM, Ganatra HA, Syed S, Cousens S, Lee AC, Black R, et al. Effect of case management on neonatal mortality due to sepsis and pneumonia. BMC Public Health [Internet]. 2011;11 Suppl 3(Suppl 3). Available from: https://pubmed.ncbi.nlm.nih.gov/21501430/

74. Chavula K, Likomwa D, Valsangkar B, Luhanga R, Chimtembo L, Dube Q, et al. Readiness of hospitals to provide Kangaroo Mother Care (KMC) and documentation of KMC service delivery: Analysis of Malawi 2014 Emergency Obstetric and Newborn Care (EmONC) survey data. J Glob Health [Internet]. 2017;7(2). Available from: https://pubmed.ncbi.nlm.nih.gov/29085623/

75. Gebremedhin S, Asefa A. Treatment-seeking for vaginal fistula in sub-Saharan Africa. PLoS One [Internet]. 2019;14(11):e0216763. Available from: https://journals.plos.org/plosone/article?id=10.1371/journal.pone.0216763

76. Panelli DM, Phillips CH, Brady PC. Incidence, diagnosis and management of tubal and nontubal ectopic pregnancies: a review. Fertil Res Pract [Internet]. 2015;1(1):15. Available from: https://www.ncbi.nlm.nih.gov/pmc/articles/PMC5424401/

77. Shaw JL V, Dey SK, Critchley HOD, Horne AW. Current knowledge of the aetiology of human tubal ectopic pregnancy. Hum Reprod Update [Internet]. 2010;16(4):432–44. Available from: https://pubmed.ncbi.nlm.nih.gov/20071358/

78. Mpiima DP, Wasswa Salongo G, Lugobe H, Ssemujju A, Mumbere Mulisya O, Masinda A, et al. Association between Prior Chlamydia trachomatis Infection and Ectopic Pregnancy at a Tertiary Care Hospital in South Western Uganda. Obstet Gynecol Int [Internet]. 2018. Available from: https://pubmed.ncbi.nlm.nih.gov/29686708/

79. Assouni Mindjah YA, Essiben F, Foumane P, Dohbit JS, Mboudou ET. Risk factors for ectopic pregnancy in a population of Cameroonian women: A case-control study. Mitchell C, editor. PLoS One [Internet]. 2018;13(12):e0207699. Available from: https://journals.plos.org/plosone/article?id=10.1371/journal.pone.0207699

80. Asah-Opoku K, Oppong SA, Ameme DK, Nuamah MA, Mumuni K, Yeboah AO, et al. Risk factors for ectopic pregnancy among pregnant women attending a tertiary healthcare facility

in Accra, Ghana. Int J of Gynaecol Obstet [Internet]. 2019;147(1):120–5. Available from: https://pubmed.ncbi.nlm.nih.gov/31344263/

81. Anorlu RI, Oluwole A, Abudu OO, Adebajo S. Risk factors for ectopic pregnancy in Lagos, Nigeria. Acta Obstet Gynecol Scand [Internet]. 2005;84(2):184–8. Available from: https://obgyn.onlinelibrary.wiley.com/doi/10.1111/j.0001-6349.2005.00684.x

82. Bailey PE, Andualem W, Brun M, Freedman L, Gbangbade S, Kante M, et al. Institutional maternal and perinatal deaths: a review of 40 low and middle income countries. BMC Pregnancy Childbirth [Internet]. 2017;17(1):295. Available from: https://bmcpregnancychildbirth.biomedcentral.com/articles/10.1186/s12884-017-1479-1

83. Farquhar CM. Ectopic pregnancy. Lancet [Internet]. 2005;366(9485):583–91. Available from: https://www.thelancet.com/journals/lancet/article/PIIS0140-6736(05)67103-6/fulltext

84. Flores AH, Kassamali S, Won GY, Stein JC, Reynolds T. Frequency of utilisation of ultrasound in the diagnosis of ectopic pregnancy in Sub-Saharan Africa countries: A systematic review. African Journal of Emergency Medicine [Internet]. 2015;5(1):31-36. Available from: https://www.sciencedirect.com/science/article/pii/S2211419X14000792

85. Saxon D, Falcone T, Mascha EJ, Marino T, Yao M, Tulandi T. A study of ruptured tubal ectopic pregnancy. Obstet Gynaecol [Internet]. 1997;90(1):46–9. Available from: https://pubmed.ncbi.nlm.nih.gov/9207811/

86. Long Y, Zhu H, Hu Y, Shen L, Fu J, Huang W, et al. Interventions for non-tubal ectopic pregnancy. Cochrane Database. Syst Rev [Internet]. 2020;7. Available from: https://www.cochranelibrary.com/cdsr/doi/10.1002/14651858.CD011174/references

87. Gure T, Sultan S, Alishum R, Ali A, Dibaba B, Usmael I, et al. Term Abdominal Pregnancy with Live Baby: Case Report from Hiwot Fana Specialized University Hospital, Eastern Ethiopia. Int Med Case Rep J [Internet]. 2021;14:689. Available from: https://www.ncbi.nlm.nih.gov/pmc/articles/PMC8488043/

88. Poordast T, Naghmehsanj Z, Vahdani R, Moradi Alamdarloo S, Ashraf MA, Samsami A, et al. Evaluation of the recurrence and fertility rate following salpingostomy in patients with tubal ectopic pregnancy. BMC Pregnancy Childbirth [Internet]. 2022;22(1):1–5. Available from: https://bmcpregnancychildbirth.biomedcentral.com/articles/10.1186/s12884-021-04299-y

89. Chen L, Zhu D, Wu Q, Yu Y. Fertility outcomes after laparoscopic salpingectomy or salpingotomy for tubal ectopic pregnancy: A retrospective cohort study of 95 patients. Int J Surg [Internet]. 2017;48:59–63. Available from: https://pubmed.ncbi.nlm.nih.gov/28951291/

90. World Health Organisation. Stillbirths [Internet] 2016 . Available from: http://www.who.int/maternal_child_adolescent/epidemiology/stillbirth/en/

91. Garrido-Gimenez C, Alijotas-Reig J. Recurrent miscarriage: causes, evaluation and management. Postgrad Med J [Internet]. 2015;91(1073):151–62. Available from: https://pubmed.ncbi.nlm.nih.gov/25681385/

92. Magnus MC, Wilcox AJ, Morken NH, Weinberg CR, Håberg SE. Role of maternal age and pregnancy history in risk of miscarriage: prospective register based study. BMJ. 2019;364:869. Available from: https://www.bmj.com/content/364/bmj.l869

93. Dellicour S, Aol G, Ouma P, Yan N, Bigogo G, Hamel MJ, et al. Weekly miscarriage rates in a community-based prospective cohort study in rural western Kenya. BMJ Open [Internet]. 2016;6(4). Available from: https://pubmed.ncbi.nlm.nih.gov/27084287/

94. Farren J, Mitchell-Jones N, Verbakel JY, Timmerman D, Jalmbrant M, Bourne T. The psychological impact of early pregnancy loss. Hum Reprod Update [Internet]. 2018;24(6):731–49. Available from: https://pubmed.ncbi.nlm.nih.gov/30204882/

95. Adeniran A, Fawole A, Abdul I, Adesina K. Spontaneous abortions (miscarriages): Analysis of cases at a tertiary center in North Central Nigeria. J Med Trop [Internet]. 2015;17(1):22. Available from: https://www.jmedtropics.org/article.asp?issn=2276-7096;year=2015;volume=17;issue=1;spage=22;epage=26;aulast=Adeniran;type=0

96. San Lazaro Campillo I, Meaney S, O'Donoghue K, Corcoran P. Miscarriage hospitalisations: A national population-based study of incidence and outcomes, 2005-2016. Reprod Health [Internet]. 2019;16(1):1–8. Available from: https://reproductive-health-journal.biomedcentral.com/articles/10.1186/s12978-019-0720-y

97. Quenby S, Gallos ID, Dhillon-Smith RK, Podesek M, Stephenson MD, Fisher J, et al. Miscarriage matters: the epidemiological, physical, psychological, and economic costs of early pregnancy loss. Lancet [Internet]. 2021;397(10285):1658–67. Available from: https://www.thelancet.com/journals/lancet/article/PIIS0140-6736(21)00682-6/fulltext

98. Polis CB, Mhango C, Philbin J, Chimwaza W, Chipeta E, Msusa A. Incidence of induced abortion in Malawi, 2015. PLoS One [Internet]. 2017;12(4). Available from: https://journals.plos.org/plosone/article?id=10.1371/journal.pone.0173639

99. Faúndes A, Alvarez F. Abortion. In: International Encyclopedia of Public Health [Internet]. Elsevier Inc.; 2008. p. 1–12. Available from: https://doi.org/10.1016/B978-012373960-5.00468-8

100. World Health Organization. Abortion care guideline [Internet]. 2022. Available from: https://www.who.int/publications/i/item/9789240039483

101. Berer M. Abortion Law and Policy Around the World: In Search of Decriminalization. Health Hum Rights [Internet]. 2017;19(1):13. Available from: https://pubmed.ncbi.nlm.nih.gov/28630538/

102. Ganatra B, Gerdts C, Rossier C, Johnson BR, Tunçalp Ö, Assifi A, et al. Global, regional, and subregional classification of abortions by safety, 2010–14: estimates from a Bayesian hierarchical model. Lancet [Internet]. 2017;390(10110):2372–81. Available from: https://pubmed.ncbi.nlm.nih.gov/28964589/

103. Daire J, Kloster MO, Storeng KT. Political Priority for Abortion Law Reform in Malawi: Transnational and National Influences. Health Hum Rights [Internet]. 2018;20(1):225. Available from: https://pubmed.ncbi.nlm.nih.gov/30008565/

104. unk. Malawi Penal Code Chapter 7:01. 1930.

105. Kalilani-Phiri L, Gebreselassie H, Levandowski BA, Kuchingale E, Kachale F, Kangaude G. The severity of abortion complications in Malawi. Int J Gynecol Obstet [Internet]. 2015;128(2):160–4. Available from: https://pubmed.ncbi.nlm.nih.gov/25468057/

106. Boah M, Bordotsiah S, Kuurdong S. Predictors of Unsafe Induced Abortion among Women in Ghana. J Pregnancy [Internet].2019;2019. Available from: https://www.hindawi.com/journals/jp/2019/9253650/

107. Adjei G, Enuameh Y, Asante KP, Baiden F, A Nettey OE, Abubakari S, et al. Predictors of abortions in Rural Ghana: A cross-sectional study. BMC Public Health [Internet].2015;15(1):1–7. Available from: https://bmcpublichealth.biomedcentral.com/articles/10.1186/s12889-015-1572-1

108. Okonofua FE, Odimegwu C, Ajabor H, Daru PH, Johnson A. Assessing the prevalence and determinants of unwanted pregnancy and induced abortion in Nigeria. Stud Fam Plann [Internet]. 1999;30(1):67–77. Available from: https://pubmed.ncbi.nlm.nih.gov/10216897/

109. Rasch V, Muhammad H, Urassa E, Bergström S. The problem of illegally induced abortion: results from a hospital-based study conducted at district level in Dar es Salaam. Trop Med Int Health [Internet]. 2000;5(7):495–502. Available from: https://pubmed.ncbi.nlm.nih.gov/10964272/

110. Stöckl H, Filippi V, Watts C, Mbwambo JKK. Induced abortion, pregnancy loss and intimate partner violence in Tanzania: A population based study. BMC Pregnancy Childbirth [Internet]. 2012;12(1):1–8. Available from: https://bmcpregnancychildbirth.biomedcentral.com/articles/10.1186/1471-2393-12-12

111. Kaye DK, Mirembe FM, Bantebya G, Johansson A, Ekstrom AM. Domestic violence as risk factor for unwanted pregnancy and induced abortion in Mulago Hospital, Kampala, Uganda. Trop Med Int Health [Internet]. 2006;11(1):90–101. Available from: https://onlinelibrary.wiley.com/doi/10.1111/j.1365-3156.2005.01531.x

112. Norris A, Harrington BJ, Grossman D, Hemed M, Hindin MJ. Abortion experiences among Zanzibari women: A chain-referral sampling study. Reprod Health [Internet]. 2016;13(1). Available from: https://reproductive-health-journal.biomedcentral.com/articles/10.1186/s12978-016-0129-9

113. Bearak JM, Popinchalk A, Beavin C, Ganatra B, Moller AB, Tunçalp Ö, et al. Country-specific estimates of unintended pregnancy and abortion incidence: a global comparative analysis of levels in 2015–2019. BMJ Glob Health [Internet]. 2022;7(3):e007151. Available from: https://gh.bmj.com/content/7/3/e007151

114. Calvert C, Owolabi OO, Yeung F, Pittrof R, Ganatra B, Tunçalp Ö, et al. The magnitude and severity of abortion-related morbidity in settings with limited access to abortion services: a systematic review and meta-regression. BMJ Glob Health [Internet]. 2018;3(3):e000692. Available from: https://gh.bmj.com/content/3/3/e000692

115. Branum A, Ahrens K. Trends in Timing of Pregnancy Awareness Among US Women. Matern Child Health J [Internet]. 2017;21(4):715–26. Available from: https://pubmed.ncbi.nlm.nih.gov/27449777/

116. Balarajan Y, Ramakrishnan U, Özaltin E, Shankar AH, Subramanian S V. Anaemia in low-income and middle-income countries. Lancet [Internet]. 2011;378:2123–35. Available from: https://pubmed.ncbi.nlm.nih.gov/21813172/

117. Munasinghe S, van den Broek N. Anaemia In Pregnancy In Malawi- A Review. Malawi Med J [Internet]. 2006;18(4):160–74. Available from: https://www.ncbi.nlm.nih.gov/pmc/articles/PMC3345625/#:~:text=Summary,%3C7.0g%2Fdl).

118. Broek NR, Rogerson SJ, Mhango CG, Kambala B, White SA, Molyneux ME. Anaemia in pregnancy in southern Malawi: prevalence and risk factors. BJOG [Internet]. 2000;107(4):445–51. Available from: https://pubmed.ncbi.nlm.nih.gov/10759260/

119. Baingana RK, Enyaru JK, Tjalsma H, Swinkels DW, Davidsson L. The aetiology of anaemia during pregnancy: A study to evaluate the contribution of iron deficiency and common infections in pregnant Ugandan women. Public Health Nutr [Internet]. 2015;18(8):1423–35. Available from: https://www.ncbi.nlm.nih.gov/pmc/articles/PMC10271691/

120. Butwick AJ, McDonnell N. Antepartum and postpartum anemia: a narrative review. Int J Obstet Anesth [Internet]. 2021;47. Available from: https://pubmed.ncbi.nlm.nih.gov/33893005/

121. Bergmann RL, Richter R, Bergmann KE, Dudenhausen JW. Prevalence and risk factors for early postpartum anemia. Eur J Obstet Gynecol Reprod Biol [Internet]. 2010;150(2):126–31. Available from: https://pubmed.ncbi.nlm.nih.gov/20303210/

122. Daru J, Zamora J, Fernández-Félix BM, Vogel J, Oladapo OT, Morisaki N, et al. Risk of maternal mortality in women with severe anaemia during pregnancy and post partum: a multilevel analysis. Lancet Glob Health [Internet]. 2018;6(5): e548–54. Available from: https://www.thelancet.com/journals/langlo/article/PIIS2214-109X(18)30078-0/fulltext

123. Liyew AM, Tesema GA, Alamneh TS, Worku MG, Teshale AB, Alem AZ, et al. Prevalence and determinants of anemia among pregnant women in East Africa; A multi-level analysis of recent Demographic and Health Surveys. PLoS One [Internet]. 2021;16(4):e0250560. Available from: https://journals.plos.org/plosone/article?id=10.1371/journal.pone.0250560

124. Ayoya MA, Spiekermann-Brouwer GM, Traoré AK, Stoltzfus RJ, Garza C. Determinants of Anemia among Pregnant Women in Mali. Food Nutr Bull [Internet]. 2006;27(1):3–11. Available from: https://journals.sagepub.com/doi/10.1177/156482650602700101

125. Adamu AL, Crampin A, Kayuni N, Amberbir A, Koole O, Phiri A, et al. Prevalence and risk factors for anemia severity and type in Malawian men and women: Urban and rural differences. Popul Health Metr [Internet]. 2017;15(1):12. Available from: http://pophealthmetrics.biomedcentral.com/articles/10.1186/s12963-017-0128-2

126. Brenner A, Roberts I, Balogun E, Bello FA, Chaudhri R, Fleming C, et al. Postpartum haemorrhage in anaemic women: assessing outcome measures for clinical trials. Trials [Internet]. 2022;23(1):1–8. Available from: https://trialsjournal.biomedcentral.com/articles/10.1186/s13063-022-06140-z

127. Ba DM, Ssentongo P, Kjerulff KH, Na M, Liu G, Gao X, et al. Adherence to Iron Supplementation in 22 Sub-Saharan African Countries and Associated Factors among Pregnant Women: A Large Population-Based Study. Curr Dev Nutr [Internet]. 2019;3(12). Available from: https://pubmed.ncbi.nlm.nih.gov/31777771/

128. Fouelifack FY, Sama JD, Sone CE. Assessment of adherence to iron supplementation among pregnant women in the Yaounde gynaeco-obstetric and paediatric hospital. Pan Afr Med J [Internet]. 2019;34. Available from: https://pubmed.ncbi.nlm.nih.gov/32201546/

129. Nasir BB, Fentie AM, Adisu MK. Adherence to iron and folic acid supplementation and prevalence of anemia among pregnant women attending antenatal care clinic at Tikur Anbessa Specialized Hospital, Ethiopia. PLoS One [Internet]. 2020;15(5):e0232625. Available from: https://www.ncbi.nlm.nih.gov/pmc/articles/PMC7197778/

130. Bambo GM, Kebede SS, Sitotaw C, Shiferaw E, Melku M. Postpartum anemia and its determinant factors among postnatal women in two selected health institutes in Gondar, Northwest Ethiopia: A facility-based, cross-sectional study. Front Med (Lausanne) [Internet]. 2023;10:1105307. Available from: https://www.ncbi.nlm.nih.gov/pmc/articles/PMC10157165/

131. Titilayo A, Palamuleni ME, Omisakin O. Sociodemographic factors influencing adherence to antenatal iron supplementation recommendations among pregnant women in Malawi: Analysis of data from the 2010 Malawi Demographic and Health Survey. Malawi Medical Journal [Internet]. 2016;28(1):1–5. Available from: https://pubmed.ncbi.nlm.nih.gov/27217909/

132. Ba DM, Ssentongo P, Kjerulff KH, Na M, Liu G, Gao X, et al. Adherence to Iron Supplementation in 22 Sub-Saharan African Countries and Associated Factors among Pregnant Women: A Large Population-Based Study. Curr Dev Nutr [Internet]. 2019;3(12). Available from: https://academic.oup.com/cdn/article/3/12/nzz120/5601697

133. World Health Organization. Diagnostic criteria and classification of hyperglycaemia first detected in pregnancy [Internet]. 2013. Available from: https://apps.who.int/iris/handle/10665/85975

134. Napso T, Yong HEJ, Lopez-Tello J, Sferruzzi-Perri AN. The Role of Placental Hormones in Mediating Maternal Adaptations to Support Pregnancy and Lactation. Front Physiol [Internet]. 2018;9(AUG). Available from: https://pubmed.ncbi.nlm.nih.gov/30174608/

135. Tal R, Taylor H. Endocrinology of Pregnancy. In: Finegold K (ed.) Endotext [Internet]. South Datmouth, MDText.com Inc., 2021 [cited 2022 May 10]; p469–76. Available from: https://www.ncbi.nlm.nih.gov/books/NBK278962/

136. Plows J, Stanley J, Baker P, Reynolds C, Vickers M. The Pathophysiology of Gestational Diabetes Mellitus. Int J Mol Sci [Internet]. 2018;19(11):3342. Available from: https://www.ncbi.nlm.nih.gov/pmc/articles/PMC6274679/

137. Mwanri AW, Kinabo J, Ramaiya K, Feskens EJM. Gestational diabetes mellitus in sub-Saharan Africa: systematic review and metaregression on prevalence and risk factors. Trop. Med. Int. Health [Internet]. 2015;20(8):983–1002. Available from: http://doi.wiley.com/10.1111/tmi.12521

138. Muche AA, Olayemi OO, Gete YK. Prevalence and determinants of gestational diabetes mellitus in Africa based on the updated international diagnostic criteria: A systematic review and meta-analysis. Arch of Public Health [Internet]. 2019;77(1):36. Available from: https://archpublichealth.biomedcentral.com/articles/10.1186/s13690-019-0362-0

139. Yang Y, Wang Z, Mo M, Muyiduli X, Wang S, Li M, et al. The association of gestational diabetes mellitus with fetal birth weight. J Diabetes Complications [Internet]. 2018;32(7):635–42. Available from: https://pubmed.ncbi.nlm.nih.gov/29907325/

140. KC K, Shakya S, Zhang H. Gestational Diabetes Mellitus and Macrosomia: A Literature Review. Ann Nutr Metab [Internet]. 2015;66(Suppl. 2):14–20. Available from: https://pubmed.ncbi.nlm.nih.gov/26045324/

141. Ye W, Luo C, Huang J, Li C, Liu Z, Liu F. Gestational diabetes mellitus and adverse pregnancy outcomes: systematic review and meta-analysis. BMJ [Internet]. 2022;377. Available from: https://www.bmj.com/content/377/bmj-2021-067946

142. Natamba BK, Namara AA, Nyirenda MJ. Burden, risk factors and maternal and offspring outcomes of gestational diabetes mellitus (GDM) in sub-Saharan Africa (SSA): a systematic review and meta-analysis. BMC Pregnancy Childbirth [Internet]. 2019;19(1):450. Available from: https://doi.org/10.1186/s12884-019-2593-z

143. Phiri TJ, Kasiya M, Allain Theresa. Prevalence of gestational diabetes mellitus in urban women in Blantyre, Malawi: a cross sectional study evaluating diagnostic criteria and traditional risk factors. South African Journal of Diabetes and Vascular Disease [Internet]. 2022;18(1). Available from: https://journals.co.za/doi/full/10.10520/ejc-sajdvd_v18_n1_a2

144. International Association of Diabetes and Pregnancy Study Group. International Association of Diabetes and Pregnancy Study Groups Recommendations on the Diagnosis and Classification of Hyperglycemia in Pregnancy. Diabetes Care [Internet]. 2010;33(3):676. Available from: https://pubmed.ncbi.nlm.nih.gov/20190296/

145. Santos S, Voerman E, Amiano P, Barros H, Beilin LJ, Bergström A, et al. Impact of maternal body mass index and gestational weight gain on pregnancy complications: an individual participant data meta-analysis of European, North American and Australian cohorts. BJOG [Internet]. 2019;126(8):984–95. Available from: https://pubmed.ncbi.nlm.nih.gov/30786138/

146. Li Z, Cheng Y, Wang D, Chen H, Chen H, Ming WK, et al. Incidence Rate of Type 2 Diabetes Mellitus after Gestational Diabetes Mellitus: A Systematic Review and Meta-Analysis of 170,139 Women. J Diabetes Res [Internet]. 2020;3076463. Available from: https://pubmed.ncbi.nlm.nih.gov/32405502/#:~:text=Results%3A%20Twenty%2Deight%20studies%20involving,)%20per%201000%20person%2Dyears.

147. Bellamy L, Casas JP, Hingorani AD, Williams D. Type 2 diabetes mellitus after gestational diabetes: a systematic review and meta-analysis. Lancet [Internet]. 2009;373(9677):1773–9. Available from: http://www.thelancet.com/article/S0140673609607315/fulltext

148. Kojima N, Klausner JD. An Update on the Global Epidemiology of Syphilis. Curr. Epidemiol. Rep. [Internet]. 2018;5(1):24–38. Available from: https://www.ncbi.nlm.nih.gov/pmc/articles/PMC6089383/

149. Korenromp EL, Rowley J, Alonso M, Mello MB, Saman Wijesooriya N, Guy Mahiané S, et al. Global burden of maternal and congenital syphilis and associated adverse birth outcomes—Estimates for 2016 and progress since 2012. PLoS One [Internet]. 2019;14(2):e0211720. Available from: https://www.ncbi.nlm.nih.gov/pmc/articles/PMC6392238/#:~:text=The%20estimated%20total%20number%20of,%E2%80%93571%3B%20Table%201).

150. Malawi Ministry of Health and Population. Integrated HIV Program Report October-December 2019. 2019.

151. Arnesen L, Martínez G, Mainero L, Serruya S, Durán P. Gestational syphilis and stillbirth in Latin America and the Caribbean. Int J Gynecol Obstet. [Internet]. 2015;128(3):241–5. Available from: https://obgyn.onlinelibrary.wiley.com/doi/abs/10.1016/j.ijgo.2014.09.017

152. Tao YT, Gao TY, Li HY, Ma YT, Li HJ, Xian-Yu CY, et al. Global, regional, and national trends of syphilis from 1990 to 2019: the 2019 global burden of disease study. BMC Public Health [Internet]. 2023;23(1):1–13. Available from: https://bmcpublichealth.biomedcentral.com/articles/10.1186/s12889-023-15510-4

153. Tchirikov M, Schlabritz-Loutsevitch N, Maher J, Buchmann J, Naberezhnev Y, Winarno AS, et al. Mid-trimester preterm premature rupture of membranes (PPROM): etiology, diagnosis, classification, international recommendations of treatment options and outcome. J Perinat Med [Internet]. 2018;46(5):465–88. Available from: https://pubmed.ncbi.nlm.nih.gov/28710882/

154. Kaya D. Risk factors of preterm premature rupture of membranes at Mulago hospital Kampala. East Afr Med J[Internet]. 2001;78(2):65–9. Available from: https://pubmed.ncbi.nlm.nih.gov/11682948/

155. Nakubulwa S, Kaye DK, Bwanga F, Tumwesigye NM, Mirembe FM. Genital infections and risk of premature rupture of membranes in Mulago Hospital, Uganda: a case control study. BMC Res Notes[Internet]. 2015;8(1). Available from: https://pubmed.ncbi.nlm.nih.gov/26475265/

156. Esteves JS, De Sá RAM, De Carvalho PRN, Coca Velarde LG. Neonatal outcome in women with preterm premature rupture of membranes (PPROM) between 18 and 26 weeks. J Matern Fetal Neonatal Med [Internet]. 2016;29(7):1108–12. Available from: https://pubmed.ncbi.nlm.nih.gov/26138545/

157. Yu H, Wang X, Gao H, You Y, Xing A. Perinatal outcomes of pregnancies complicated by preterm premature rupture of the membranes before 34 weeks of gestation in a tertiary center in China: A retrospective review. Biosci Trends [Internet]. 2015;9(1):35–41. Available from: https://pubmed.ncbi.nlm.nih.gov/25787907/

158. Chebbo A, Tan S, Kassis C, Tamura L, Carlson RW. Maternal Sepsis and Septic Shock. Crit Care Clin [Internet]. 2016;32(1):119–35. Available from: https://pubmed.ncbi.nlm.nih.gov/26600449/

159. Sae-Lin P, Wanitpongpan P. Incidence and risk factors of preterm premature rupture of membranes in singleton pregnancies at Siriraj Hospital. Journal Obstet Gynaecol Res. [Internet]. 2019;45(3):573–7. Available from: https://pubmed.ncbi.nlm.nih.gov/30537150/

160. Onwughara CE, Moodley D, Valashiya N, Sebitloane M. Preterm prelabour rupture of membranes (PPROM) and pregnancy outcomes in association with HIV-1 infection in KwaZulu-Natal, South Africa. BMC Pregnancy Childbirth [Internet]. 2020;20(1). Available from: https://pubmed.ncbi.nlm.nih.gov/32272919/

161. Tiruye G, Shiferaw K, Tura AK, Debella A, Musa A. Prevalence of premature rupture of membrane and its associated factors among pregnant women in Ethiopia: A systematic review and meta-analysis. SAGE Open Med. [Internet]. 2021;9. Available from: https://www.ncbi.nlm.nih.gov/pmc/articles/PMC8558797/

162. Okeke T, Enwereji J, Okoro O, Adiri C, Ezugwu E, Agu P. The Incidence and Management Outcome of Preterm Premature Rupture of Membranes (PPROM) in a Tertiary Hospital in Nigeria. American Journal of Clinical Medicine Research [Internet]. 2014;2(1):14–7. Available from: http://pubs.sciepub.com/ajcmr/2/1/4/index.html

163. The American College of Obstetricians and Gynaecologists. Definition of Term Pregnancy. Available from: https://www.acog.org/clinical/clinical-guidance/committee-opinion/articles/2013/11/definition-of-term-pregnancy. [Cited June 1 2022]

164. van den Broek NR, Jean-Baptiste R, Neilson JP. Factors Associated with Preterm, Early Preterm and Late Preterm Birth in Malawi. PLoS One [Internet]. 2014;9(3):e90128. Available from: https://journals.plos.org/plosone/article?id=10.1371/journal.pone.0090128

165. Frey HA, Klebanoff MA. The epidemiology, etiology, and costs of preterm birth. Semin Fetal Neonatal Med [Internet]. 2016;21(2):68–73. Available from: https://pubmed.ncbi.nlm.nih.gov/26794420/

166. Laelago T, Yohannes T, Tsige G. Determinants of preterm birth among mothers who gave birth in East Africa: Systematic review and meta-analysis. Ital J Pediat [Internet]. 2020;46(1):10. Available from: https://pubmed.ncbi.nlm.nih.gov/31992346/

167. Antony KM, Kazembe PN, Pace RM, Levison J, Phiri H, Chiudzu G, et al. Population-Based Estimation of the Preterm Birth Rate in Lilongwe, Malawi: Making Every Birth Count. AJP Rep [Internet]. 2020;10(1):E78–86. Available from: https://www.ncbi.nlm.nih.gov/pmc/articles/PMC7062552/

168. Elphinstone RE, Weckman AM, McDonald CR, Tran V, Zhong K, Madanitsa M, et al. Early malaria infection, dysregulation of angiogenesis, metabolism and inflammation across pregnancy, and risk of preterm birth in Malawi: A cohort study. PLoS Med [Internet]. 2019;16(10). Available from: https://pubmed.ncbi.nlm.nih.gov/31574087/

169. Perin J, Mulick A, Yeung D, Villavicencio F, Lopez G, Strong KL, et al. Global, regional, and national causes of under-5 mortality in 2000–19: an updated systematic analysis with implications for the Sustainable Development Goals. Lancet Child Adolesc Health [Internet].

2022;6(2):106–15. Available from: http://www.thelancet.com/article/S2352464221003114/fulltext

170. Muhe LM, McClure EM, Nigussie AK, Mekasha A, Worku B, Worku A, et al. Major causes of death in preterm infants in selected hospitals in Ethiopia (SIP): a prospective, cross-sectional, observational study. Lancet Glob Health [Internet]. 2019;7(8):e1130–8. Available from: https://www.thelancet.com/journals/langlo/article/PIIS2214-109X(19)30220-7/fulltext

171. Johnson S, Evans TA, Draper ES, Field DJ, Manktelow BN, Marlow N, et al. Neurodevelopmental outcomes following late and moderate prematurity: a population-based cohort study. Arch Dis Child Fetal Neonatal Ed [Internet]. 2015;100(4):F301–8. Available from: https://pubmed.ncbi.nlm.nih.gov/25834170/

172. Jarjour IT. Neurodevelopmental Outcome After Extreme Prematurity: A Review of the Literature. Pediatr Neurol [Internet]. 2015;52(2):143–52. Available from: https://www.sciencedirect.com/science/article/abs/pii/S0887899414006584

173. Heslehurst N, Vieira R, Hayes L, Crowe L, Jones D, Robalino S, et al. Maternal body mass index and post-term birth: a systematic review and meta-analysis. Obes Rev. [Internet]. 2017;18(3): 293–308. Available from: https://www.ncbi.nlm.nih.gov/pmc/articles/PMC5324665/

174. Muglu J, Rather H, Arroyo-Manzano D, Bhattacharya S, Balchin I, Khalil A, et al. Risks of stillbirth and neonatal death with advancing gestation at term: A systematic review and meta-analysis of cohort studies of 15 million pregnancies. PLoS Med [Internet]. 2019;16(7):e1002838. Available from: https://journals.plos.org/plosmedicine/article?id=10.1371/journal.pmed.1002838

175. Chawanpaiboon S, Vogel JP, Moller AB, Lumbiganon P, Petzold M, Hogan D, et al. Global, regional, and national estimates of levels of preterm birth in 2014: a systematic review and modelling analysis. Lancet Glob Health [Internet]. 2019;7(1):e37–46. Available from: https://www.thelancet.com/journals/langlo/article/PIIS2214-109X(18)30451-0/fulltext

176. Deng K, Huang Y, Wang Y, Zhu J, Mu Y, Li X, et al. Prevalence of postterm births and associated maternal risk factors in China: data from over 6 million births at health facilities between 2012 and 2016. Sci Rep [Internet]. 2019;9(1). Available from: https://pubmed.ncbi.nlm.nih.gov/30670707/

177. Bonet M, Nogueira Pileggi V, Rijken MJ, Coomarasamy A, Lissauer D, Souza JP, et al. Towards a consensus definition of maternal sepsis: results of a systematic review and expert consultation. Reprod Health [Internet]. 2017;14(1):1–13. Available from: https://reproductive-health-journal.biomedcentral.com/articles/10.1186/s12978-017-0321-6

178. Bonet M, Brizuela V, Abalos E, Cuesta C, Baguiya A, Chamillard M, et al. Frequency and management of maternal infection in health facilities in 52 countries (GLOSS): a 1-week

inception cohort study. Lancet Glob Health [Internet]. 2020;8(5):e661–71. Available from: https://linkinghub.elsevier.com/retrieve/pii/S2214109X20301091

179. Kachikis A, Eckert LO, Walker C, Bardají A, Varricchio F, Lipkind HS, et al. Chorioamnionitis: Case definition & guidelines for data collection, analysis, and presentation of immunization safety data. Vaccine [Internet]. 2019;37(52):7610–22. Available from: https://www.ncbi.nlm.nih.gov/pmc/articles/PMC6891229/

180. Menon R, Taylor RN, Fortunato SJ. Chorioamnionitis - A complex pathophysiologic syndrome. Placenta [Internet]. 2010;31(2):113–20. Available from: https://pubmed.ncbi.nlm.nih.gov/20031205/

181. Seaward PG, Hannah ME, Myhr TL, Farine D, Ohlsson A, Wang EE, et al. International Multicentre Term Prelabor Rupture of Membranes Study: evaluation of predictors of clinical chorioamnionitis and postpartum fever in patients with prelabor rupture of membranes at term. Am J Obstet Gynecol [Internet]. 1997;177(5):1024–9. Available from: https://pubmed.ncbi.nlm.nih.gov/9396886/

182. Racusin DA, Chen HY, Bhalwal A, Wiley R, Chauhan SP. Chorioamnionitis and adverse outcomes in low-risk pregnancies: a population-based study. J Matern-Fetal Neonatal Med [Internet]. 2021;1–9. Available from: https://pubmed.ncbi.nlm.nih.gov/33596755/

183. Beck C, Gallagher K, Taylor LA, Goldstein JA, Mithal LB, Gernand AD. Chorioamnionitis and Risk for Maternal and Neonatal Sepsis: A Systematic Review and Meta-analysis. Obstet Gynaecol. [Internet]. 2021;137(6):1007–22. Available from: https://pubmed.ncbi.nlm.nih.gov/33957655/

184. Pugni L, Pietrasanta C, Acaia B, Merlo D, Ronchi A, Ossola MW, et al. Chorioamnionitis and neonatal outcome in preterm infants: a clinical overview. J Matern-Fetal Neonatal Med [Internet]. 2016;29(9):1525–9. Available from: https://pubmed.ncbi.nlm.nih.gov/26135227/

185. Woodd SL, Montoya A, Barreix M, Pi L, Calvert C, Rehman AM, et al. Incidence of maternal peripartum infection: A systematic review and meta-analysis. PLoS Med [Internet]. 2019;16(12). Available from: https://pubmed.ncbi.nlm.nih.gov/31821329/

186. Dalton E, Castillo E. Post partum infections: A review for the non-OBGYN. Obstet Med [Internet]. 2014;7(3):98–102. Available from: https://www.ncbi.nlm.nih.gov/pmc/articles/PMC4934978/

187. Mackeen AD, Packard RE, Ota E, Speer L. Antibiotic regimens for postpartum endometritis. Cochrane Database Syst Rev [Internet]. 2015;2015(2). Available from: https://pubmed.ncbi.nlm.nih.gov/25922861/

188. Newton ER, Prihoda TJ, Gibbs RS. A clinical and microbiologic analysis of risk factors for puerperal endometritis. Obstet Gynecol [Internet]. 1990;75(3 Pt 1):402–6. Available from: https://pubmed.ncbi.nlm.nih.gov/2406660/

189. Adane F, Mulu A, Seyoum G, Gebrie A, Lake A. Prevalence and root causes of surgical site infection among women undergoing caesarean section in Ethiopia: A systematic review and meta-analysis. Patient Safety in Surgery [Internet]. 2019;13:34 Available from: https://pubmed.ncbi.nlm.nih.gov/31673291/

190. World Health Organization. WHO recommendation on prophylactic antibiotics for women undergoing caesarean section [Internet]. 2021. Available from: https://apps.who.int/iris/handle/10665/341865

191. Getaneh T, Negesse A, Dessie G. Prevalence of surgical site infection and its associated factors after cesarean section in Ethiopia: systematic review and meta-analysis. BMC Pregnancy Childbirth [Internet]. 2020;20(1). Available from: https://pubmed.ncbi.nlm.nih.gov/32434486/

192. Moulton L, Lachiewicz M, Liu X, Goje O. Catheter-associated urinary tract infection (CAUTI) after term cesarean delivery: incidence and risk factors at a multi-center academic institution. J Matern Fetal Neonatal Med [Internet]. 2018;31(3):395–400. Available from: https://pubmed.ncbi.nlm.nih.gov/28114875

193. Gundersen TD, Krebs L, Loekkegaard ECL, Rasmussen SC, Glavind J, Clausen TD. Postpartum urinary tract infection by mode of delivery: a Danish nationwide cohort study. BMJ Open [Internet]. 2018;8(3). Available from: https://bmjopen.bmj.com/content/8/3/e018479

194. Say L, Chou D, Gemmill A, Tunçalp Ö, Moller AB, Daniels J, et al. Global causes of maternal death: A WHO systematic analysis. Lancet Glob Health [Internet]. 2014;2(6):e323–33. Available from: https://www.thelancet.com/journals/langlo/article/PIIS2214-109X(14)70227-X/fulltext

195. Gluck O, Mizrachi Y, Ganer Herman H, Bar J, Kovo M, Weiner E. The correlation between the number of vaginal examinations during active labor and febrile morbidity, a retrospective cohort study. BMC Pregnancy Childbirth [Internet]. 2020;20(1). Available from: https://bmcpregnancychildbirth.biomedcentral.com/articles/10.1186/s12884-020-02925-9

196. Newton ER, Prihoda TJ, Gibbs RS. A clinical and microbiologic analysis of risk factors for puerperal endometritis. Obstet and Gynaecol [Internet]. 1990;75(3 Pt 1):402–6. Available from: https://pubmed.ncbi.nlm.nih.gov/2406660/

197. Ngonzi J, Bebell LM, Fajardo Y, Boatin AA, Siedner MJ, Bassett I V., et al. Incidence of postpartum infection, outcomes and associated risk factors at Mbarara regional referral hospital in Uganda. BMC Pregnancy Childbirth [Internet]. 2018;18(1):1–11. Available from: https://bmcpregnancychildbirth.biomedcentral.com/articles/10.1186/s12884-018-1891-1

198. The Royal College of Obstetricians & Gynaecologists. Antepartum Haemorrhage (Green-top Guideline No. 63).2011. Available from: https://www.rcog.org.uk/guidance/browse-all-guidance/green-top-guidelines/antepartum-haemorrhage-green-top-guideline-no-63/

199. Fan D, Wu S, Liu L, Xia Q, Wang W, Guo X, et al. Prevalence of antepartum hemorrhage in women with placenta previa: A systematic review and meta-analysis. Sci Rep [Internet]. 2017;7. Available from: Prevalence of antepartum hemorrhage in women with placenta previa: A systematic review and meta-analysis

200. Senkoro EE, Mwanamsangu AH, Chuwa FS, Msuya SE, Mnali OP, Brown BG, et al. Frequency, Risk Factors, and Adverse Fetomaternal Outcomes of Placenta Previa in Northern Tanzania. J Pregnancy [Internet]. 2017. Available from: https://pubmed.ncbi.nlm.nih.gov/28321338/

201. Tikkanen M, Nuutila M, Hiilesmaa V, Paavonen J, Ylikorkala O. Clinical presentation and risk factors of placental abruption. Acta Obstet Gynecol Scand [Internet]. 2006;85(6):700–5. Available from: https://pubmed.ncbi.nlm.nih.gov/16752262/

202. Downes KL, Grantz KL, Shenassa ED. Maternal, Labor, Delivery, and Perinatal Outcomes Associated with Placental Abruption: A Systematic Review. Am J Perinatol [Internet]. 2017;34(10):935–57. Available from: https://pubmed.ncbi.nlm.nih.gov/28329897/

203. Beltman J, Van Den Akker T, Van Lonkhuijzen L, Schmidt A, Chidakwani R, Van Roosmalen J. Beyond maternal mortality: Obstetric hemorrhage in a Malawian district. Acta Obstet Gynecol Scand [Internet]. 2011;90(12):1423–7. Available from: https://pubmed.ncbi.nlm.nih.gov/21682698/

204. Takai IU, Sayyadi BM, Galadanci HS. Antepartum Hemorrhage: A Retrospective Analysis from a Northern Nigerian Teaching Hospital. Int J Appl Basic Med Res [Internet]. 2017;7(2):112. Available from: https://pubmed.ncbi.nlm.nih.gov/28584742/

205. Jauniaux E, Alfirevic Z, Bhide A, Belfort M, Burton G, Collins S, et al. Placenta Praevia and Placenta Accreta: Diagnosis and Management. BJOG [Internet]. 2019;126(1):e1–48. Available from: https://obgyn.onlinelibrary.wiley.com/doi/full/10.1111/1471-0528.15306

206. Yang Q, Wen SW, Oppenheimer L, Chen XK, Black D, Gao J, et al. Association of caesarean delivery for first birth with placenta praevia and placental abruption in second pregnancy. BJOG [Internet]. 2007;114(5):609–13. Available from: https://obgyn.onlinelibrary.wiley.com/doi/10.1111/j.1471-0528.2007.01295.x

207. To WWK, Leung WC. Placenta previa and previous cesarean section. Int J Gynaecol Obstet [Internet]. 1995;51(1):25–31. Available from: https://pubmed.ncbi.nlm.nih.gov/8582514/

208. Hendricks MS, Chow YH, Bhagavath B, Singh K. Previous Cesarean Section and Abortion as Risk Factors for Developing Placenta Previa. Int J Gynaecol [Internet]. 1999;25(2):137–42. Available from: https://pubmed.ncbi.nlm.nih.gov/10379130/

209. Ananth C V., Lavery JA, Vintzileos AM, Skupski DW, Varner M, Saade G, et al. Severe placental abruption: Clinical definition and associations with maternal complications. Am J Obstet Gynecol [Internet]. 2016;214(2):272.e1-272.e9. Available from: https://pubmed.ncbi.nlm.nih.gov/26393335/

210. Macheku GS, Philemon RN, Oneko O, Mlay PS, Masenga G, Obure J, et al. Frequency, risk factors and feto-maternal outcomes of abruptio placentae in Northern Tanzania: A registry-based retrospective cohort study. BMC Pregnancy Childbirth [Internet]. 2015;15(1):1–10. Available from: https://pubmed.ncbi.nlm.nih.gov/26446879/

211. Wandabwa J, Doyle P, Paul K, Wandabwa MA, Aziga F. Risk factors for severe abruptio placenta in Mulago Hospital, Kampala, Uganda. Afr Health Sci [Internet]. 2005;5(4):285–90. Available from: https://www.ncbi.nlm.nih.gov/pmc/articles/PMC1831945/

212. Cresswell JA, Ronsmans C, Calvert C, Filippi V. Prevalence of placenta praevia by world region: A systematic review and meta-analysis. Trop Med Int Health [Internet]. 2013; 18(6):712-24. Available from: https://pubmed.ncbi.nlm.nih.gov/23551357/

213. Owolabi H, Ameh C, Bar-Zeev S, Adaji S, Kachale F, van den Broek N. Establishing cause of maternal death in Malawi via facility-based review and application of the ICD-MM classification. BJOG. 2014;121:95–101. Available from: https://obgyn.onlinelibrary.wiley.com/doi/full/10.1111/1471-0528.12998

214. Mammaro A, Carrara S, Cavaliere A, Ermito S, Dinatale A, Pappalardo EM, et al. Hypertensive Disorders of Pregnancy. J Prenat Med [Internet]. 2009; 3(1):1. Available from: https://pubmed.ncbi.nlm.nih.gov/22439030/

215. Sibai BM. Etiology and management of postpartum hypertension-preeclampsia. Am J Obstet Gynecol [Internet]. 2012;206(6):470–5. Available from: https://pubmed.ncbi.nlm.nih.gov/21963308/

216. Matthys LA, Coppage KH, Lambers DS, Barton JR, Sibai BM. Delayed postpartum preeclampsia: An experience of 151 cases. Am J Obstet Gynecol [Internet]. 2004;190(5):1464–6. Available from: https://pubmed.ncbi.nlm.nih.gov/15167870/

217. Goel A, Maski MR, Bajracharya S, Wenger JB, Zhang D, Salahuddin S, et al. Epidemiology and mechanisms of de novo and persistent hypertension in the postpartum period. Circulation [Internet]. 2015;132(18):1726–33. Available from: https://pubmed.ncbi.nlm.nih.gov/26416810/

218. Gathiram P, Moodley J. Pre-eclampsia: its pathogenesis and pathophysiolgy. Cardiovasc J Afr [Internet]. 2016;27(2):71–8. Available from: https://www.ncbi.nlm.nih.gov/pmc/articles/PMC4928171/

219. Foo L, Tay J, Lees CC, McEniery CM, Wilkinson IB. Hypertension in Pregnancy: Natural History and Treatment Options. Curr Hypertens Rep [Internet]. 2015;17(5):36. Available from: https://pubmed.ncbi.nlm.nih.gov/25833457/

220. Laine K, Murzakanova G, Sole KB, Pay AD, Heradstveit S, Raïsänen S. Prevalence and risk of pre-eclampsia and gestational hypertension in twin pregnancies: A population-based register study. BMJ Open [Internet]. 2019;9(7). Available from: https://bmjopen.bmj.com/content/9/7/e029908

221. Meazaw MW, Chojenta C, Muluneh MD, Loxton D. Systematic and meta-analysis of factors associated with preeclampsia and eclampsia in sub-Saharan Africa. PLoS One [Internet]. 2020;15(8). Available from: https://pubmed.ncbi.nlm.nih.gov/32813750/

222. Bartsch E, Medcalf KE, Park AL, Ray JG, Al-Rubaie ZTA, Askie LM, et al. Clinical risk factors for pre-eclampsia determined in early pregnancy: Systematic review and meta-analysis of large cohort studies. The BMJ [Internet]. 2016;353. Available from: https://www.bmj.com/content/353/bmj.i1753#:~:text=Considering%20each%20risk%20factor%20and,prepregnancy%20BMI%20%3E30%2C%20and%20assisted

223. Nakimuli A, Nakubulwa S, Kakaire O, Osinde MO, Mbalinda SN, Kakande N, et al. The burden of maternal morbidity and mortality attributable to hypertensive disorders in pregnancy: A prospective cohort study from Uganda. BMC Pregnancy Childbirth [Internet]. 2016;16(1):1–8. Available from: https://bmcpregnancychildbirth.biomedcentral.com/articles/10.1186/s12884-016-1001-1#:~:text=The%20case%2Dspecific%20maternal%20mortality%20ratio%20was%20780%20per%20100%2C000,100%2C000%20live%20births%20for%20eclampsia.

224. Shen M, Smith GN, Rodger M, White RR, Walker MC, Wen SW. Comparison of risk factors and outcomes of gestational hypertension and pre-eclampsia. Räisänen SH, editor. PLoS One [Internet]. 2017;12(4):e0175914. Available from: https://journals.plos.org/plosone/article?id=10.1371/journal.pone.0175914

225. Chen KH, Seow KM, Chen LR. Progression of gestational hypertension to pre-eclampsia: A cohort study of 20,103 pregnancies. Pregnancy Hypertens [Internet]. 2017;10:230–7. Available from: https://pubmed.ncbi.nlm.nih.gov/29153686/

226. Kumar NR, Grobman WA, Barry O, Clement AC, Lancki N, Yee LM. Evaluating the maternal and perinatal sequelae of severe gestational hypertension. Am J Obstet Gynecol MFM [Internet]. 2021;3(1). Available from: https://pubmed.ncbi.nlm.nih.gov/33451611/

227. Wiles K, Damodaram M, Frise C. Severe hypertension in pregnancy. Clinical Medicine [Internet]. 2021;21(5):e451. Available from: https://www.rcpjournals.org/content/clinmedicine/21/5/e451

228. Wang W, Xie X, Yuan T, Wang Y, Zhao F, Zhou Z, et al. Epidemiological trends of maternal hypertensive disorders of pregnancy at the global, regional, and national levels: a population-based study. BMC Pregnancy Childbirth [Internet]. 2021;21(1):364. Available from: https://bmcpregnancychildbirth.biomedcentral.com/articles/10.1186/s12884-021-03809-2

229. Noubiap JJ, Bigna JJ, Nyaga UF, Jingi AM, Kaze AD, Nansseu JR, et al. The burden of hypertensive disorders of pregnancy in Africa: A systematic review and meta-analysis. The Journal of Clinical Hypertension [Internet]. 2019;21(4):479–88. Available from: https://pubmed.ncbi.nlm.nih.gov/30848083/

230. Melamed N, Ray JG, Hladunewich M, Cox B, John Kingdom. Gestational Hypertension and Preeclampsia: Are They the Same Disease? JOGC [Internet]. 2014;36(7):642-647.Available from: https://pubmed.ncbi.nlm.nih.gov/25184984/

231. Nyfløt LT, Ellingsen L, Yli BM, Øian P, Vangen S. Maternal deaths from hypertensive disorders: lessons learnt. Acta Obstet Gynecol Scand [Internet]. 2018;97(8):976–87. Available from: https://pubmed.ncbi.nlm.nih.gov/29663318/

232. World Health Organization. WHO recommendations for Prevention and treatment of pre-eclampsia and eclampsia [Internet]. 2011. Available from: https://www.who.int/publications/i/item/9789241548335

233. Matthys LA, Coppage KH, Lambers DS, Barton JR, Sibai BM. Delayed postpartum preeclampsia: An experience of 151 cases. Am J Obstet Gynecol [Internet]. 2004;190(5):1464–6. Available from: https://pubmed.ncbi.nlm.nih.gov/15167870/

234. Chames MC, Livingston JC, Ivester TS, Barton JR, Sibai BM. Late postpartum eclampsia: A preventable disease? Am J Obstet Gynecol [Internet]. 2002;186(6):1174–7. Available from: http://www.ajog.org/article/S0002937802000145/fulltext

235. Vousden N, Lawley E, Seed PT, Gidiri MF, Goudar S, Sandall J, et al. Incidence of eclampsia and related complications across 10 low- and middle-resource geographical regions: Secondary analysis of a cluster randomised controlled trial. PLoS Med [Internet]. 2019;16(3):e1002775. Available from: http://dx.plos.org/10.1371/journal.pmed.1002775

236. Harrison MS, Ali S, Pasha O, Saleem S, Althabe F, Berrueta M, et al. A prospective population-based study of maternal, fetal, and neonatal outcomes in the setting of prolonged labor, obstructed labor and failure to progress in low- and middle-income countries. Reprod Health [Internet]. 2015;12 Suppl 2(Suppl 2). Available from: https://pubmed.ncbi.nlm.nih.gov/26063492/

237. Pavličev M, Romero R, Mitteroecker P. Evolution of the human pelvis and obstructed labor: new explanations of an old obstetrical dilemma. Am J Obstet Gynecol [Internet]. 2020;222(1):3–16. Available from: https://pubmed.ncbi.nlm.nih.gov/31251927/

238. Ayenew AA. Incidence, causes, and maternofetal outcomes of obstructed labor in Ethiopia: systematic review and meta-analysis. Reprod Health [Internet]. 2021;18(1):61. Available from: https://pubmed.ncbi.nlm.nih.gov/33691736/

239. Neilson JP, Lavender T, Quenby S, Wray S. Obstructed labour: Reducing maternal death and disability during pregnancy. Br Med Bull [Internet]. 2003;67(1):191–204. Available from: https://academic.oup.com/bmb/article/67/1/191/330404

240. Duffy CR, Moore JL, Saleem S, Tshefu A, Bose CL, Chomba E, et al. Malpresentation in low- and middle-income countries: Associations with perinatal and maternal outcomes in the Global Network. Acta Obstet Gynecol Scand [Internet]. 2019;98(3):300–8. Available from: https://pubmed.ncbi.nlm.nih.gov/30414270/

241. Tsvieli O, Sergienko R, Sheiner E. Risk factors and perinatal outcome of pregnancies complicated with cephalopelvic disproportion: A population-based study. Arch Gynecol Obstet [Internet]. 2012;285(4):931–6. Available from: https://pubmed.ncbi.nlm.nih.gov/21932085/

242. Barageine JK, Tumwesigye NM, Byamugisha JK, Almroth L, Faxelid E. Risk factors for obstetric fistula in western uganda: A case control study. PLoS One [Internet]. 2014;9(11). Available from: https://pubmed.ncbi.nlm.nih.gov/25401756/

243. Howells I, Israel J. Predictors of cephalopelvic disproportion in labour a tertiary hospital in Bayelsa State, Nigeria. Nigerian Journal of Medicine [Internet]. 2018;27(3):205. Available from: https://www.ajol.info/index.php/njm/article/view/177613#:~:text=Short%20stature%2C%20fetal%20macrosomia%20and%20pelvic%20abnormalities%20are%20common%20risk%20factors.

244. Toh-Adam R, Srisupundit K, Tongsong T. Short stature as an independent risk factor for cephalopelvic disproportion in a country of relatively small-sized mothers. Arch Gynecol Obstet [Internet]. 2012;285(6):1513–6. Available from: https://pubmed.ncbi.nlm.nih.gov/22187064/#:~:text=The%20short%20stature%20was%20defined,%25%20CI%201.8%2D3.0).

245. Drew LB, Wilkinson JP, Nundwe W, Moyo M, Mataya R, Mwale M, et al. Long-term outcomes for women after obstetric fistula repair in Lilongwe, Malawi: A qualitative study. BMC Pregnancy Childbirth [Internet]. 2016;16(1):2. Available from: https://bmcpregnancychildbirth.biomedcentral.com/articles/10.1186/s12884-015-0755-1#:~:text=Nearly%20all%20women%20believed%20their,had%20limited%20challenges%20when%20reintegrating.

246. Verhoeven CJM, Nuij C, Janssen-Rolf CRM, Schuit E, Bais JMJ, Oei SG, et al. Predictors for failure of vacuum-assisted vaginal delivery: a case-control study. Eur J Obstet Gynecol Reprod Biol [Internet]. 2016;200:29–34. Available from: https://pubmed.ncbi.nlm.nih.gov/26967343/

247. Kabiri D, Lipschuetz M, Cohen SM, Yagel O, Levitt L, Herzberg S, et al. Vacuum extraction failure is associated with a large head circumference. J Matern Fetal Neonatal Med [Internet]. 2018;32(20):3325–30. Available from: https://www.tandfonline.com/doi/abs/10.1080/14767058.2018.1463364

248. Damron DP, Capeless EL. Operative vaginal delivery: A comparison of forceps and vacuum for success rate and risk of rectal sphincter injury. Am J Obstet Gynecol [Internet]. 2004;191(3):907–10. Available from: https://pubmed.ncbi.nlm.nih.gov/15467563/

249. NMPA Project Team. National Maternity and Perinatal Audit: Clinical Report 2022 [Internet]. 2022 [cited 2023 Aug 5]. Available from: www.hqip.org.uk/national

250. Hofmeyr GJ, Say L, Gülmezoglu AM. WHO systematic review of maternal mortality and morbidity: the prevalence of uterine rupture. BJOG [Internet]. 2005;112(9):1221–8. Available from: https://pubmed.ncbi.nlm.nih.gov/16101600/

251. Guise JM, McDonagh MS, Osterweil P, Nygren P, Chan BKS, Helfand M. Systematic review of the incidence and consequences of uterine rupture in women with previous caesarean section. BMJ [Internet]. 2004;329(7456):19. Available from: https://pubmed.ncbi.nlm.nih.gov/15231616/

252. Delafield R, Pirkle CM, Dumont A. Predictors of uterine rupture in a large sample of women in Senegal and Mali: cross-sectional analysis of QUARITE trial data. BMC Pregnancy Childbirth [Internet]. 2018;18(1):432. Available from: https://bmcpregnancychildbirth.biomedcentral.com/articles/10.1186/s12884-018-2064-y

253. Alemu AA, Bitew MS, Gelaw KA, Zeleke LB, Kassa GM. Prevalence and determinants of uterine rupture in Ethiopia: a systematic review and meta-analysis. Sci Rep [Internet]. 2020;10(1):1–11. Available from: https://www.nature.com/articles/s41598-020-74477-z

254. Motomura K, Ganchimeg T, Nagata C, Ota E, Vogel JP, Betran AP, et al. Incidence and outcomes of uterine rupture among women with prior caesarean section: WHO Multicountry Survey on Maternal and Newborn Health. Sci Rep [Internet]. 2017;7. Available from: https://www.nature.com/articles/srep44093#:~:text=In%20conclusion%2C%20based%20on%20a,%25%20in%20low%2DHDI%20countries.

255. Abrar S, Abrar T, Sayyed E, Naqvi SA. Ruptured uterus: Frequency, risk factors and feto-maternal outcome: Current scenario in a low-resource setup. PLoS One [Internet]. 2022;17(4):e0266062. Available from: https://journals.plos.org/plosone/article?id=10.1371/journal.pone.0266062

256. Astatikie G, Limenih MA, Kebede M. Maternal and fetal outcomes of uterine rupture and factors associated with maternal death secondary to uterine rupture. BMC Pregnancy

Childbirth [Internet]. 2017;17(1):117. Available from: http://bmcpregnancychildbirth.biomedcentral.com/articles/10.1186/s12884-017-1302-z

257. World Health Organization, United Nations Population Fund (UNFPA), United Nations Children's Fund (UNICEF). Managing complications in pregnancy and childbirth: a guide for midwives and doctors 2nd ed. [Internet]. 2017. Available from: https://apps.who.int/iris/handle/10665/255760

258. Wang YL, Su TH. Obstetric Uterine Rupture of the Unscarred Uterus: A Twenty-Year Clinical Analysis. Gynecol Obstet Invest [Internet]. 2006;62(3):131–5. Available from: https://dx.doi.org/10.1159/000093031

259. Vernekar M, Rajib R. Unscarred Uterine Rupture: A Retrospective Analysis. J Obstet Gynaecol India [Internet]. 2016;66(Suppl 1):51. Available from: https://www.ncbi.nlm.nih.gov/pmc/articles/PMC5016409/#:~:text=The%20key%20factor%20of%20uterine,with%20improvement%20in%20obstetric%20practice.

260. Sinha M, Gupta R, Gupta P, Rani R, Kaur R, Singh R. Uterine Rupture: A Seven Year Review at a Tertiary Care Hospital in New Delhi, India. Indian J Community Med [Internet]. 2016;41(1):45. Available from: https://www.ncbi.nlm.nih.gov/pmc/articles/PMC4746953/

261. Anderson JM, Etches D. Prevention and Management of Postpartum Haemorrhage. BJOG [Internet]. 2017;124(5):e106–49. Available from: https://pubmed.ncbi.nlm.nih.gov/17390600/

262. Sebghati M, Chandraharan E. An update on the risk factors for and management of obstetric haemorrhage. Women's Health [Internet]. 2017;13(2):34. Available from: https://pubmed.ncbi.nlm.nih.gov/28681676/

263. Majid E, Malik S, Huma Z, Fatima S, Yasmeen H, Korejo R, et al. Frequency and Clinical Outcomes of Women with Postpartum Haemorrhage at a Tertiary Care Hospital Karachi. Journal of The Society of Obstetricians and Gynaecologists of Pakistan [Internet]. 2022;12(2):95–9. Available from: https://jsogp.net/index.php/jsogp/article/view/491

264. Ijaiya MA, Aboyeji AP, Abubakar D. Analysis of 348 consecutive cases of primary postpartum haemorrhage at a tertiary hospital in Nigeria.J Obstet Gynaecol [Internet]. 2004;23(4):374–7. Available from: https://pubmed.ncbi.nlm.nih.gov/12881075/

265. Lutomski JE, Byrne BM, Devane D, Greene RA. Increasing trends in atonic postpartum haemorrhage in Ireland: an 11-year population-based cohort study. BJOG [Internet]. 2012;119(3):306–14. Available from: https://pubmed.ncbi.nlm.nih.gov/22168794/

266. Dupont C, Rudigoz RC, Cortet M, Touzet S, Colin C, Rabilloud M, et al. [Frequency, causes and risk factors of postpartum haemorrhage: a population-based study in 106 French maternity

units]. J Gynecol Obstet Biol Reprod (Paris) [Internet]. 2013;43(3):244–53. Available from: https://pubmed.ncbi.nlm.nih.gov/22168794/

267. Hoveyda F, MacKenzie IZ. Secondary postpartum haemorrhage: incidence, morbidity and current management. BJOG [Internet]. 2001;108(9):927–30. Available from: https://pubmed.ncbi.nlm.nih.gov/11563461/

268. Ende HB, Lozada MJ, Chestnut DH, Osmundson SS, Walden RL, Shotwell MS, et al. Risk Factors for Atonic Postpartum Hemorrhage: A Systematic Review and Meta-analysis. Obstet Gynecol. 2021;137(2):305–23. Available from: https://pubmed.ncbi.nlm.nih.gov/33417319/

269. Kassebaum NJ, Barber RM, Bhutta ZA, Dandona L, Gething PW, Hay SI, et al. Global, regional, and national levels of maternal mortality, 1990–2015: a systematic analysis for the Global Burden of Disease Study 2015. The Lancet [Internet]. 2016;388(10053):1775–812. Available from: https://linkinghub.elsevier.com/retrieve/pii/S0140673616314702

270. Tort J, Rozenberg P, Traoré M, Fournier P, Dumont A. Factors associated with postpartum hemorrhage maternal death in referral hospitals in Senegal and Mali: A cross-sectional epidemiological survey. BMC Pregnancy Childbirth [Internet]. 2015;15(1):235. Available from: https://pubmed.ncbi.nlm.nih.gov/26423997/

271. Carroli G, Cuesta C, Abalos E, Gulmezoglu AM. Epidemiology of postpartum haemorrhage: a systematic review. Best Pract Res Clin Obstet Gynaecol [Internet]. 2008;22(6):999–1012. Available from: https://pubmed.ncbi.nlm.nih.gov/18819848/

272. Calvert C, Thomas SL, Ronsmans C, Wagner KS, Adler AJ, Filippi V. Identifying Regional Variation in the Prevalence of Postpartum Haemorrhage: A Systematic Review and Meta-Analysis. Hernandez A V., editor. PLoS One [Internet]. 2012;7(7):e41114. Available from: https://pubmed.ncbi.nlm.nih.gov/22844432/

273. Lokken EM, Mathur A, Bunge KE, Fairlie L, Makanani B, Beigi R, et al. Pooled Prevalence of Adverse Pregnancy and Neonatal Outcomes in Malawi, South Africa, Uganda, and Zimbabwe: Results From a Systematic Review and Meta-Analyses to Inform Trials of Novel HIV Prevention Interventions During Pregnancy. Front Reprod Health [Internet]. 2021;3. Available from: https://pubmed.ncbi.nlm.nih.gov/35187529/

274. World Health Organization. WHO recommendations for the prevention and treatment of postpartum haemorrhage [Internet]. 2012. Available from: https://apps.who.int/iris/bitstream/handle/10665/75411/9789241548502_eng.pdf

275. World Health Organization. WHO recommendations: Uterotonics for the prevention of postpartum haemorrhage [Internet]. 2018. Available from: https://apps.who.int/iris/bitstream/handle/10665/277276/9789241550420-eng.pdf

276. B-Lynch C, Coker A, Lawal AH, Abu J, Cowen MJ. The B-Lynch surgical technique for the control of massive postpartum haemorrhage: an alternative to hysterectomy? Five cases reported. BJOG [Internet]. 1997;104(3):372–5. Available from: https://pubmed.ncbi.nlm.nih.gov/9091019/

277. Chainarong N, Deevongkij K, Petpichetchian C. Secondary postpartum hemorrhage: Incidence, etiologies, and clinical courses in the setting of a high cesarean delivery rate. PLoS One [Internet]. 2022;17(3). Available from: https://www.ncbi.nlm.nih.gov/pmc/articles/PMC8887715/

278. Babarinsa IA, Hayman RG, Draycott TJ. Secondary post-partum haemorrhage: challenges in evidence-based causes and management. Eur J Obstet Gynecol Reprod Biol [Internet]. 2011;159(2):255–60. Available from: https://pubmed.ncbi.nlm.nih.gov/21839573/

279. Bateman BT, Berman MF, Riley LE, Leffert LR. The epidemiology of postpartum hemorrhage in a large, nationwide sample of deliveries. Anesth Analg [Internet]. 2010;110(5):1368–73. Available from: https://journals.lww.com/anesthesia-analgesia/Fulltext/2010/05000/The_Epidemiology_of_Postpartum_Hemorrhage_in_a.21.aspx

280. Cengiz H, Ekin M, Karakas S, Kaya C, Yasar L. Management of intractable postpartum haemorrhage in a tertiary center: A 5-year experience. Niger Med J [Internet]. 2012;53(2):85. Available from: https://www.ncbi.nlm.nih.gov/pmc/articles/PMC3530254/

281. UNICEF, WHO, World Bank Group, UN. A neglected tragedy: The global burden of stillbirths 2020 [Internet]. 2020. Available from: https://www.unicef.org/reports/neglected-tragedy-global-burden-of-stillbirths-2020

282. Madhi SA, Briner C, Maswime S, Mose S, Mlandu P, Chawana R, et al. Causes of stillbirths among women from South Africa: a prospective, observational study. Lancet Glob Health [Internet]. 2019;7(4):e503-e512. Available from: https://www.thelancet.com/journals/langlo/article/PIIS2214-109X(18)30541-2/fulltext#:~:text=Of%20298%20cases%20(born%20to,with%20fetal%20invasive%20bacterial%20infection)%2C

283. Metaferia AM, Muula AS. Stillbirths and hospital early neonatal deaths at Queen Elizabeth Central Hospital, Blantyre-Malawi. Int Arch Med [Internet]. 2009;2(1):25. Available from: https://pubmed.ncbi.nlm.nih.gov/19719841/#:~:text=Stillbirths%20comprised%203.4%25%20of%20all,deaths%20(PD)%20were%20EHND.

284. McClure EM, Goldenberg RL. Understanding causes of stillbirth: moving in the right direction. Lancet Glob Health [Internet]. 2019;7(4):e400–1. Available from: https://pubmed.ncbi.nlm.nih.gov/30879498

285. Ahmed I, Ali SM, Amenga-Etego S, Ariff S, Bahl R, Baqui AH, et al. Population-based rates, timing, and causes of maternal deaths, stillbirths, and neonatal deaths in south Asia and sub-Saharan Africa: a multi-country prospective cohort study. Lancet Glob Health. 2018;6(12):e1297–308. Available from: https://www.thelancet.com/journals/langlo/article/PIIS2214-109X(18)30385-1/fulltext

286. Reinebrant H, Leisher S, Coory M, Henry S, Wojcieszek A, Gardener G, et al. Making stillbirths visible: a systematic review of globally reported causes of stillbirth. BJOG [Internet]. 2018;125(2):212–24. Available from: https://pubmed.ncbi.nlm.nih.gov/29193794/

287. Ashish KC, Wrammert J, Ewald U, Clark RB, Gautam J, Baral G, et al. Incidence of intrapartum stillbirth and associated risk factors in tertiary care setting of Nepal: A case-control study. Reprod Health [Internet]. 2016;13(1):1–11. Available from: https://reproductive-health-journal.biomedcentral.com/articles/10.1186/s12978-016-0226-9

288. McClure EM, Silver RM, Kim J, Ahmed I, Kallapur M, Ghanchi N, et al. Maternal infection and stillbirth: a review. J Matern Fetal Neonatal Med [Internet]. 2022; 35(23):4442-4450. Available from: https://www.tandfonline.com/doi/abs/10.1080/14767058.2020.1852206

289. Moore KA, Simpson JA, Scoullar MJL, McGready R, Fowkes FJI. Quantification of the association between malaria in pregnancy and stillbirth: a systematic review and meta-analysis. Lancet Glob Health [Internet]. 2017;5(11):e1101–12. Available from: https://pubmed.ncbi.nlm.nih.gov/28967610/

290. Xiong T, Mu Y, Liang J, Zhu J, Li X, Li J, et al. Hypertensive disorders in pregnancy and stillbirth rates: a facility-based study in China. Bull World Health Organ [Internet]. 2018;96(8):531. Available from: https://www.ncbi.nlm.nih.gov/pmc/articles/PMC6083384/

291. Tabatabaee HR, Zahedi A, Etemad K, Valadbeigi T, Mahdavi S, Enayatrad M, et al. Risk of Stillbirth in Women with Gestational Diabetes and High Blood Pressure. Iran J Public Health [Internet]. 2020;49(4):773. Available from: https://pubmed.ncbi.nlm.nih.gov/32548058/

292. Yu L, Zeng XL, Cheng ML, Yang GZ, Wang B, Xiao ZW, et al. Quantitative assessment of the effect of pre-gestational diabetes and risk of adverse maternal, perinatal and neonatal outcomes. Oncotarget [Internet]. 2017;8(37):61048. Available from: https://pubmed.ncbi.nlm.nih.gov/28977845/

293. Da Silva FT, Gonik B, McMillan M, Keech C, Dellicour S, Bhange S, et al. Stillbirth: Case definition and guidelines for data collection, analysis, and presentation of maternal immunization safety data. Vaccine [Internet]. 2016;34(49):6057–68. Available from: https://www.ncbi.nlm.nih.gov/pmc/articles/PMC5139804/

294. Ashish KC, Berkelhamer S, Gurung R, Hong Z, Wang H, Sunny AK, et al. The burden of and factors associated with misclassification of intrapartum stillbirth: Evidence from a large scale

multicentric observational study. Acta Obstet Gynecol Scand [Internet]. 2020;99(3):303–11. Available from: https://pubmed.ncbi.nlm.nih.gov/31600823

295. Lawn JE, Blencowe H, Waiswa P, Amouzou A, Mathers C, Hogan D, et al. Stillbirths: Rates, risk factors, and acceleration towards 2030. Lancet [Internet]. 2016;387(10018):587–603. Available from: https://www.thelancet.com/journals/lancet/article/PIIS01406736(15)00837-5/fulltext

296. Aminu M, Mathai M, van den Broek N. Application of the ICD-PM classification system to stillbirth in four sub-Saharan African countries. Dandona R, editor. PLoS One [Internet]. 2019;14(5):e0215864. Available from: https://journals.plos.org/plosone/article?id=10.1371/journal.pone.0215864

297. Aminu M, Unkels R, Mdegela M, Utz B, Adaji S, van den Broek N. Causes of and factors associated with stillbirth in low- and middle-income countries: a systematic literature review. BJOG [Internet]. 2014;121:141–53. Available from: https://pubmed.ncbi.nlm.nih.gov/25236649/

298. Drukker L, Hants Y, Sharon E, Sela HY, Grisaru-Granovsky S. Perimortem cesarean section for maternal and fetal salvage: concise review and protocol. Acta Obstet Gynecol Scand [Internet]. 2014;93(10):965–72. Available from: https://onlinelibrary.wiley.com/doi/full/10.1111/aogs.12464

299. UN Inter-agency Group for Child Mortality Estimation. CME Info - Stillbirth rate (Malawi) [Internet]. 2021. Available from: https://childmortality.org/data/Malawi

300. Syed M, Javed H, Yakoob MY, Bhutta ZA. Effect of screening and management of diabetes during pregnancy on stillbirths. BMC Public Health. 2011;11 Suppl 3(Suppl 3):S2. Available from: https://bmcpublichealth.biomedcentral.com/articles/10.1186/1471-2458-11-S3-S2

301. Darmstadt GL, Yakoob M, Haws RA, Menezes E V., Soomro T, Bhutta ZA. Reducing stillbirths: Interventions during labour. BMC Pregnancy Childbirth [Internet]. 2009;9(Suppl. 1):1–43. Available from: https://bmcpregnancychildbirth.biomedcentral.com/articles/10.1186/1471-2393-9-S1-S6

302. Ota E, da Silva Lopes K, Middleton P, Flenady V, Wariki WMV, Rahman MO, et al. Antenatal interventions for preventing stillbirth, fetal loss and perinatal death: an overview of Cochrane systematic reviews. Cochrane Database Syst Rev [Internet]. 2020;2020(12). Available from: https://www.cochranelibrary.com/cdsr/doi/10.1002/14651858.CD009599.pub2/full

303. Yakoob MY, Ali MA, Ali MU, Imdad A, Lawn JE, Van Den Broek N, et al. The effect of providing skilled birth attendance and emergency obstetric care in preventing stillbirths. BMC Public Health [Internet]. 2011;11 Suppl 3(Suppl 3). Available from: https://pubmed.ncbi.nlm.nih.gov/21501458/

304. Stamatakos M, Sargedi C, Stasinou T, Kontzoglou K. Vesicovaginal Fistula: Diagnosis and Management. Indian J Surg [Internet]. 2014. p. 131–6. Available from: https://pubmed.ncbi.nlm.nih.gov/24891778/

305. Debeche-Adams TH, Bohl JL. Rectovaginal fistulas. Clin Colon Rectal Surg [Internet]. 2010;23(2):99–103. Available from: https://pubmed.ncbi.nlm.nih.gov/21629627/

306. Arrowsmith S, Hamlin EC, Wall LL. Obstructed labor injury complex: obstetric fistula formation and the multifaceted morbidity of maternal birth trauma in the developing world. Obstet Gynecol Surv [Internet]. 1996;51(9):568–74. Available from: https://pubmed.ncbi.nlm.nih.gov/8873157/

307. Roka ZG, Akech M, Wanzala P, Omolo J, Gitta S, Waiswa P. Factors associated with obstetric fistulae occurrence among patients attending selected hospitals in Kenya, 2010: a case control study. BMC Pregnancy Childbirth [Internet]. 2013;13. Available from: https://bmcpregnancychildbirth.biomedcentral.com/articles/10.1186/1471-2393-13-56#:~:text=From%20this%20study%2C%20the%20significant,services%20and%20illiteracy%20among%20women.

308. Changole J, Kafulafula U, Sundby J, Thorsen V. Community perceptions of obstetric fistula in Malawi. Cult Health Sex [Internet]. 2019;21(5):605–17. Available from: https://pubmed.ncbi.nlm.nih.gov/30280975/

309. Yeakey MP, Chipeta E, Taulo F, Tsui AO. The lived experience of Malawian women with obstetric fistula. Cult Health Sex [Internet]. 2009;11(5):499–513. Available from: https://pubmed.ncbi.nlm.nih.gov/19444686/

310. Adler AJ, Ronsmans C, Calvert C, Filippi V. Estimating the prevalence of obstetric fistula: A systematic review and meta-analysis. BMC Pregnancy Childbirth [Internet]. 2013;13(1):1–14. Available from: https://pubmed.ncbi.nlm.nih.gov/24373152/

311. Trovik J, Thornhill HF, Kiserud T. Incidence of obstetric fistula in Norway: a population-based prospective cohort study. Acta Obstet Gynecol Scand [Internet]. 2016;95(4):405–10. Available from: https://pubmed.ncbi.nlm.nih.gov/26713965/

312. Kalilani-Phiri L V., Umar E, Lazaro D, Lunguzi J, Chilungo A. Prevalence of obstetric fistula in Malawi. Int J Gynecol Obstet [Internet]. 2010;109(3):204–8. Available from: https://obgyn.onlinelibrary.wiley.com/doi/abs/10.1016/j.ijgo.2009.12.019

313. Lewis Wall L, Belay S, Haregot T, Dukes J, Berhan E, Abreha M. A case-control study of the risk factors for obstetric fistula in Tigray, Ethiopia. Int Urogynecol J [Internet]. 2017;28(12):1817–24. Available from: https://pubmed.ncbi.nlm.nih.gov/28550462

314. Rijken Y, Chilopora GC. Urogenital and recto-vaginal fistulas in southern Malawi: a report on 407 patients. Int J Gynaecol Obstet [Internet]. 2007;99 Suppl 1(SUPPL. 1). Available from: https://pubmed.ncbi.nlm.nih.gov/17869256

315. Manuck TA, Rice MM, Bailit JL, Grobman WA, Reddy UM, Wapner RJ, et al. Preterm neonatal morbidity and mortality by gestational age: A contemporary cohort. Am J Obstet Gynecol [Internet]. 2016;215(1):103.e1-103.e14. Available from: https://pubmed.ncbi.nlm.nih.gov/26772790/

316. Wang D, Duke R, Chan RP, Campbell JP. Retinopathy of prematurity in Africa: a systematic review. Ophthalmic Epidemiol [Internet]. 2019;2(4):223–30. Available from: https://doi.org/10.1080/09286586.2019.1585885

317. Pickerd N, Kotecha S. Pathophysiology of respiratory distress syndrome. Paediatr Child Health [Internet]. 2009;19(4):153–7. Available from: https://linkinghub.elsevier.com/retrieve/pii/S1751722208003053

318. Stoelhorst GMSJ, Rijken M, Martens SE, Brand R, den Ouden AL, Wit JM, et al. Changes in neonatology: comparison of two cohorts of very preterm infants (gestational age. Pediatrics [Internet]. 2005;115(2):396–405. Available from: https://pubmed.ncbi.nlm.nih.gov/15689337/

319. st. Clair C, Norwitz ER, Woensdregt K, Cackovic M, Shaw JA, Malkus H, et al. The Probability of Neonatal Respiratory Distress Syndrome as a Function of Gestational Age and Lecithin/Sphingomyelin Ratio. Am J Perinatol [Internet]. 2008;25(8):473. Available from: https://www.ncbi.nlm.nih.gov/pmc/articles/PMC3095020/

320. Condò V, Cipriani S, Colnaghi M, Bellù R, Zanini R, Bulfoni C, et al. Neonatal respiratory distress syndrome: are risk factors the same in preterm and term infants? J Matern Fetal Neonatal Med. [Internet]. 2016;30(11):1267–72. Available from: https://www.tandfonline.com/doi/abs/10.1080/14767058.2016.1210597

321. Li Y, Wang W, Zhang D. Maternal diabetes mellitus and risk of neonatal respiratory distress syndrome: a meta-analysis [Internet]. Vol. 56, Acta Diabetologica [Internet]. 2019;56:p729–40. Available from: https://pubmed.ncbi.nlm.nih.gov/30955125/

322. Olack B, Santos N, Inziani M, Moshi V, Oyoo P, Nalwa G, et al. Causes of preterm and low birth weight neonatal mortality in a rural community in Kenya: evidence from verbal and social autopsy. BMC Pregnancy Childbirth [Internet]. 2021;21(1):1–9. Available from: https://bmcpregnancychildbirth.biomedcentral.com/articles/10.1186/s12884-021-04012-z

323. Minuye Birihane B, Alebachew Bayih W, Yeshambel Alemu A, Belay DM, Demis A. The burden of hyaline membrane disease, mortality and its determinant factors among preterm neonates

admitted at Debre Tabor General Hospital, North Central Ethiopia: A retrospective follow up study. PLoS One [Internet]. 2021;16(3):e0249365. Available from: https://pubmed.ncbi.nlm.nih.gov/33784349/

324. Donda K, Vijayakanthi N, Dapaah-Siakwan F, Bhatt P, Rastogi D, Rastogi S. Trends in epidemiology and outcomes of respiratory distress syndrome in the United States. Pediatr Pulmonol [Internet]. 2019;54(4):405–14. Available from: https://pubmed.ncbi.nlm.nih.gov/30663263/

325. Azad K, Costello A. Extreme caution is needed before scale-up of antenatal corticosteroids to reduce preterm deaths in low-income settings. Lancet Glob Health [Internet]. 2014;2(4):e191–2. Available from: https://www.thelancet.com/journals/langlo/article/PIIS2214-109X(14)70020-8/fulltext

326. The World Health Organization. WHO recommendations on antenatal corticosteroids for improving preterm birth outcomes [Internet]. 2022. Available from: https://www.who.int/publications/i/item/9789240057296

327. Tann C. Consultant Neonatologist. Personal Communication. 4th March 2020.

328. Basnet S, Adhikari S, Jha J, Pandey MR. Neonatal Intensive Care Unit Admissions among Preterm Babies in a Tertiary Care Centre: A Descriptive Cross-sectional Study. JNMA J Nepal Med Assoc [Internet]. 2022;60(248):364. Available from: https://www.ncbi.nlm.nih.gov/pmc/articles/PMC9252253/

329. Li Y, Wang W, Zhang D. Maternal diabetes mellitus and risk of neonatal respiratory distress syndrome: a meta-analysis. Acta Diabetol. 2019;56(7):729–40. Available from: https://pubmed.ncbi.nlm.nih.gov/30955125/

330. Gou X, Li X, Qi T, Pan Z, Zhu X, Wang H, et al. A birth population-based survey of preterm morbidity and mortality by gestational age. BMC Pregnancy Childbirth [Internet]. 2021;21(1):1–12. Available from: https://bmcpregnancychildbirth.biomedcentral.com/articles/10.1186/s12884-021-03726-4

331. Fottrell E, Osrin D, Alcock G, Azad K, Bapat U, Beard J, et al. Cause-specific neonatal mortality: analysis of 3772 neonatal deaths in Nepal, Bangladesh, Malawi and India. Arch Dis Child Fetal Neonatal Ed [Internet]. 2015;100(5):F439–47. Available from: https://fn.bmj.com/content/100/5/F439

332. Wang D, Duke R, Chan RP, Campbell JP. Retinopathy of prematurity in Africa: a systematic review. Ophthalmic Epidemiol [Internet]. 2019;2(4):223–30. Available from: https://doi.org/10.1080/09286586.2019.1585885

333. Lloyd T, Isenberg S, Lambert SR. Current management of retinopathy of prematurity in sub-Saharan Africa. J AAPOS [Internet]. 2020;24(3):151.e1. Available from: https://www.ncbi.nlm.nih.gov/pmc/articles/PMC7508758/

334. American College of Obstetricians and Gynaecologists (ACOG). Neonatal Encephalopathy and Neurologic Outcome: Task Force Report [Internet]. Available from: https://www.acog.org/clinical/clinical-guidance/task-force-report/articles/2014/neonatal-encephalopathy-and-neurologic-outcome

335. Gopagondanahalli KR, Li J, Fahey MC, Hunt RW, Jenkin G, Miller SL, et al. Preterm Hypoxic–Ischemic Encephalopathy. Front Pediatr [Internet]. 2016;4(OCT). Available from: https://www.ncbi.nlm.nih.gov/pmc/articles/PMC5071348/

336. Aslam S, Strickland T, Molloy EJ. Neonatal Encephalopathy: Need for Recognition of Multiple Etiologies for Optimal Management. Front Pediatr [Internet]. 2019;7(MAR):142. Available from: https://pubmed.ncbi.nlm.nih.gov/31058120/

337. Sandoval Karamian AG, Mercimek-Andrews S, Mohammad K, Molloy EJ, Chang T, Chau V, et al. Neonatal encephalopathy: Etiologies other than hypoxic-ischemic encephalopathy. Semin Fetal Neonatal Med [Internet]. 2021;26(5):101272. Available from: https://pubmed.ncbi.nlm.nih.gov/34417137/

338. Tann CJ, Nakakeeto M, Willey BA, Sewegaba M, Webb EL, Oke I, et al. Perinatal risk factors for neonatal encephalopathy: an unmatched case-control study. Arch Dis Child Fetal Neonatal Ed [Internet]. 2018;103(3):F250–6. Available from: https://fn.bmj.com/content/103/3/F250

339. Mrelashvili A, Russ JB, Ferriero DM, Wusthoff CJ. The Sarnat score for neonatal encephalopathy: looking back and moving forward. Pediatric Research 2020 88:6 [Internet]. 2020;88(6):824–5. Available from: https://www.nature.com/articles/s41390-020-01143-5

340. Sarnat HB, Sarnat MS. Neonatal Encephalopathy Following Fetal Distress: A Clinical and Electroencephalographic Study. Arch Neurol [Internet]. 1976;33(10):696–705. Available from: https://jamanetwork.com/journals/jamaneurology/fullarticle/574959

341. Kurinczuk JJ, White-Koning M, Badawi N. Epidemiology of neonatal encephalopathy and hypoxic–ischaemic encephalopathy. Early Hum Dev [Internet]. 2010;86(6):329–38. Available from: https://pubmed.ncbi.nlm.nih.gov/20554402/

342. Kukka AJ, Waheddoost S, Brown N, Litorp H, Wrammert J, KC A. Incidence and outcomes of intrapartum-related neonatal encephalopathy in low-income and middle-income countries: a systematic review and meta-analysis. BMJ Glob Health [Internet]. 2022;7(12):e010294. Available from: https://pubmed.ncbi.nlm.nih.gov/36581333/

343. Vossius C, Lotto E, Lyanga S, Mduma E, Msemo G, Perlman J, et al. Cost-Effectiveness of the "Helping Babies Breathe" Program in a Missionary Hospital in Rural Tanzania. PLoS One

[Internet]. 2014;9(7):e102080. Available from: https://journals.plos.org/plosone/article?id=10.1371/journal.pone.0102080

344. Shane AL, Sánchez PJ, Stoll BJ. Neonatal sepsis. Lancet [Internet]. 2017;390(10104):1770–80. Available from: https://www.thelancet.com/journals/lancet/article/PIIS0140-6736(17)31002-4/fulltext

345. Seale AC, Mwaniki M, Newton CR, Berkley JA. Maternal and early onset neonatal bacterial sepsis: burden and strategies for prevention in sub-Saharan Africa. Lancet Infect Dis [Internet]. 2009;9(7):428–38. Available from: https://linkinghub.elsevier.com/retrieve/pii/S1473309909701720

346. Simonsen KA, Anderson-Berry AL, Delair SF, Dele Davies H. Early-Onset Neonatal Sepsis. Clin Microbiol Rev [Internet]. 2014;27(1):21. Available from: https://pubmed.ncbi.nlm.nih.gov/24396135/

347. Bedford Russell AR, Kumar R. Early onset neonatal sepsis: diagnostic dilemmas and practical management. Arch Dis Child Fetal Neonatal Ed [Internet]. 2015;100(4):F350–4. Available from: https://pubmed.ncbi.nlm.nih.gov/25425652/

348. Chan GJ, Lee AC, Baqui AH, Tan J, Black RE. Risk of early-onset neonatal infection with maternal infection or colonization: a global systematic review and meta-analysis. PLoS Med [Internet]. 2013;10(8). Available from: https://pubmed.ncbi.nlm.nih.gov/23976885/

349. Belachew A, Tewabe T. Neonatal sepsis and its association with birth weight and gestational age among admitted neonates in Ethiopia: Systematic review and meta-analysis. BMC Pediatr [Internet]. 2020;20(1):1–7. Available from: https://doi.org/10.1186/s12887-020-1949-x

350. Pan T, Zhu Q, Li P, Hua J, Feng X. Late-onset neonatal sepsis in Suzhou, China. BMC Pediatr [Internet]. 2020;20(1). Available from: https://pubmed.ncbi.nlm.nih.gov/32471377/

351. Glaser MA, Hughes LM, Jnah A, Newberry D, Harris-Haman PA. Neonatal Sepsis: A Review of Pathophysiology and Current Management Strategies. Advances in Neonatal Care [Internet]. 2021;21(1):49–60. Available from: https://pubmed.ncbi.nlm.nih.gov/32956076/

352. Giannoni E, Agyeman PKA, Stocker M, Posfay-Barbe KM, Heininger U, Spycher BD, et al. Neonatal Sepsis of Early Onset, and Hospital-Acquired and Community-Acquired Late Onset: A Prospective Population-Based Cohort Study. J Pediatr [Internet]. 2018;201:106-114.e4. Available from: https://pubmed.ncbi.nlm.nih.gov/30054165/

353. Seale AC, Blencowe H, Manu AA, Nair H, Bahl R, Qazi SA, et al. Estimates of possible severe bacterial infection in neonates in sub-Saharan Africa, south Asia, and Latin America for 2012: a systematic review and meta-analysis. Lancet Infect Dis [Internet]. 2014;14(8):731–41. Available from: http://www.ncbi.nlm.nih.gov/pubmed/24974250

354. Fleischmann C, Reichert F, Cassini A, Horner R, Harder T, Markwart R, et al. Global incidence and mortality of neonatal sepsis: a systematic review and meta-analysis. Arch Dis Child [Internet]. 2021;106(8): 745–752. Available from: http://dx.doi.org/10.1136/archdischild-2020-320217

355. Cai S, Thompson DK, Yang JYM, Anderson PJ. Short- and Long-Term Neurodevelopmental Outcomes of Very Preterm Infants with Neonatal Sepsis: A Systematic Review and Meta-Analysis. Children [Internet]. 2019;6(12). Available from: https://pubmed.ncbi.nlm.nih.gov/31805647/

356. Ou Z, Yu D, Liang Y, He H, He W, Li Y, et al. Global trends in incidence and death of neonatal disorders and its specific causes in 204 countries/territories during 1990–2019. BMC Public Health [Internet]. 2022;22(1):1–13. Available from: https://pubmed.ncbi.nlm.nih.gov/35183143/

357. The World Health Organization. WHO recommendations on newborn health: guidelines approved by the WHO Guidelines Review Committee [Internet]. 2017. Available from: https://www.who.int/publications/i/item/WHO-MCA-17.07

358. Chan GJ, Lee AC, Baqui AH, Tan J, Black RE. Risk of Early-Onset Neonatal Infection with Maternal Infection or Colonization: A Global Systematic Review and Meta-Analysis. PLoS Med [Internet]. 2013;10(8).Available from: https://pubmed.ncbi.nlm.nih.gov/23976885/

359. Debes AK, Kohli A, Walker N, Edmond K, Mullany LC. Time to initiation of breastfeeding and neonatal mortality and morbidity: a systematic review. BMC Public Health [Internet]. 2013;13 Suppl 3(Suppl 3). Available from: https://pubmed.ncbi.nlm.nih.gov/24564770/

360. Duijts L, Ramadhani MK, Moll HA. Breastfeeding protects against infectious diseases during infancy in industrialized countries. A systematic review. Matern Child Nutr [Internet]. 2009;5(3):199. Available from: https://pubmed.ncbi.nlm.nih.gov/19531047/

361. Hanson LÅ, Korotkova M. The role of breastfeeding in prevention of neonatal infection. Seminars in Neonatology [Internet]. 2002;7(4):275–81. Available from: https://pubmed.ncbi.nlm.nih.gov/12401297/#:~:text=Thus%20breastfeeding%20modulates%20the%20early,well%20as%20several%20other%20infections.

362. World Health Organization, CDC, International Clearinghouse for Birth Defects. Birth defects surveillance: a manual for programme managers, second edition. [Internet]. 2020. Available from: https://www.who.int/publications/i/item/9789240015395

363. Feldkamp ML, Carey JC, Byrne JLB, Krikov S, Botto LD. Etiology and clinical presentation of birth defects: population based study. BMJ [Internet]. 2017;357:2249. Available from: https://www.bmj.com/content/357/bmj.j2249

364. Toufaily MH, Westgate MN, Lin AE, Holmes LB. Causes of Congenital Malformations. Birth Defects Res [Internet]. 2018;110(2):87–91. Available from: https://onlinelibrary.wiley.com/doi/full/10.1002/bdr2.1105

365. Brent RL. Environmental Causes of Human Congenital Malformations: The Pediatrician's Role in Dealing With These Complex Clinical Problems Caused by a Multiplicity of Environmental and Genetic Factors. Pediatrics [Internet]. 2004;113(Supplement_3):957–68. Available from: https://pubmed.ncbi.nlm.nih.gov/15060188/

366. Kang L, Cao G, Jing W, Liu J, Liu M. Global, regional, and national incidence and mortality of congenital birth defects from 1990 to 2019. Eur J Pediatr [Internet]. 2023;182(4):1781–92. Available from: https://link.springer.com/article/10.1007/s00431-023-04865-w

367. Sattolo ML, Arbour L, Bilodeau-Bertrand M, Lee GE, Nelson C, Auger N. Association of Birth Defects With Child Mortality Before Age 14 Years. JAMA Netw Open [Internet]. 2022;5(4):e226739–e226739. Available from: https://jamanetwork.com/journals/jamanetworkopen/fullarticle/2790917

368. Boyle B, Addor MC, Arriola L, Barisic I, Bianchi F, Csáky-Szunyogh M, et al. Estimating Global Burden of Disease due to congenital anomaly: an analysis of European data. Arch Dis Child Fetal Neonatal Ed [Internet]. 2018;103(1):F22–8. Available from: https://fn.bmj.com/content/103/1/F22

369. Strong KL, Pedersen J, Johansson EW, Cao B, Diaz T, Guthold R, et al. Patterns and trends in causes of child and adolescent mortality 2000–2016: setting the scene for child health redesign. BMJ Glob Health [Internet]. 2021;6(3):e004760. Available from: https://gh.bmj.com/content/6/3/e004760

370. Tataryn M, Polack S, Chokotho L, Mulwafu W, Kayange P, Banks LM, et al. Childhood disability in Malawi: A population based assessment using the key informant method. BMC Pediatr [Internet]. 2017;17(1):1–12. Available from: https://bmcpediatr.biomedcentral.com/articles/10.1186/s12887-017-0948-z

371. Gajewski J, Pittalis C, Borgstein E, Bijlmakers L, Mwapasa G, Cheelo M, et al. Critical shortage of capacity to deliver safe paediatric surgery in sub-Saharan Africa: evidence from 67 hospitals in Malawi, Zambia, and Tanzania. Front Pediatr. 2023;11:1189676. Available from: https://pubmed.ncbi.nlm.nih.gov/37325346/

372. Stokes SC, Farmer DL. Paediatric surgery for congenital anomalies: the next frontier for global health. Lancet [Internet]. 2021;398(10297):280–1. Available from: http://www.thelancet.com/article/S0140673621015476/fulltext

373. Adane F, Afework M, Seyoum G, Gebrie A. Prevalence and associated factors of birth defects among newborns in sub-Saharan African countries: a systematic review and meta-analysis.

Pan Afr Med J [Internet]. 2020;36:1–22. Available from: https://www.ncbi.nlm.nih.gov/pmc/articles/PMC7388615/

374. I-TECH. Birth Defects Surveillance in Malawi – I-TECH [Internet]. 2022. Available from: https://www.go2itech.org/2022/03/birth-defects-surveillance-in-malawi/

375. Sargent RG. Verification and validation of simulation models. Journal of Simulation [Internet]. 2013;7(1):12–24. Available from: https://www.tandfonline.com/action/journalInformation?journalCode=tjsm20

376. ISO, IEC, IEEE. ISO/IEC/IEEE 24765:2017. Systems and software engineering — Vocabulary. 2017. Available from: https://www.iso.org/standard/71952.html

377. Krekel H. pytest: helps you write better programs — pytest documentation [Internet]. 2015. Available from: https://docs.pytest.org/en/7.3.x/

378. Eddy DM, Hollingworth W, Caro JJ, Tsevat J, McDonald KM, Wong JB. Model Transparency and Validation - A Report of the ISPOR-SMDM Modeling Good Research Practices Task Force–7. Medical Decision Making [Internet]. 2012;32(5):733–43. Available from: https://journals.sagepub.com/doi/10.1177/0272989X12454579

379. Klügl F. A validation methodology for agent-based simulations. In: Proceedings of the ACM Symposium on Applied Computing [Internet]. 2008;39–43. Available from: https://www.researchgate.net/publication/221001583_A_validation_methodology_for_agent-based_simulations

380. Hazelbag CM, Dushoff J, Dominic EM, Mthombothi ZE, Delva W. Calibration of individual-based models to epidemiological data: A systematic review. PLoS Comput Biol [Internet]. 2020;16(5):e1007893. Available from: https://dx.plos.org/10.1371/journal.pcbi.1007893

381. Murray EJ, Robins JM, Seage GR, Lodi S, Hyle EP, Reddy KP, et al. Using Observational Data to Calibrate Simulation Models. Medical Decision Making [Internet]. 2018;38(2):212–24. Available from: http://www.ncbi.nlm.nih.gov/pubmed/29141153

382. McCulloch J, Ge J, Ward JA, Heppenstall A, Polhill JG, Malleson N. Calibrating Agent-Based Models Using Uncertainty Quantification Methods. 2021:65:3 [Internet]. 2022;25(2). Available from: https://www.jasss.org/25/2/1.html

383. Malawi Ministry of Health and Population. National Digital Health Strategy 2020-2025 [Internet]. 2020. Available from: https://www.healthdatacollaborative.org/fileadmin/uploads/hdc/Documents/Country_documents/Malawi/Malawi_Digital_Health_Strategy__20-25.pdf

384. World Health Organization, UNICEF, UNFPA, World Bank Group, UNDESA/Population Division. Trends in maternal mortality 2000 to 2020 [Internet]. 2023. Available from: https://www.who.int/publications/i/item/9789240068759

385. Wang Z, Fix MJ, Hug L, Mishra A, You D, Blencowe H, et al. Estimating the Stillbirth Rate for 195 Countries Using A Bayesian Sparse Regression Model with Temporal Smoothing. Annals of Applied Statistics [Internet]. 2020;16(4):2101–21. Available from: https://arxiv.org/abs/2010.03551v1

386. World Health Orgnaization. Strategies toward ending preventable maternal mortality (EPMM) [Internet]. 2015. Available from: https://www.who.int/publications/i/item/9789241508483

387. Dieleman J, Sadat N, Chang A, Fullman N, Abbafati C, Acharya P et al. Trends in future health financing and coverage: future health spending and universal health coverage in 188 countries, 2016-40. Lancet [Internet]. 2018;391(10132):1783-1798. Available from: https://www.thelancet.com/journals/lancet/article/PIIS0140-6736(18)30697-4/fulltext#app-1
