# Supplementary material for: An individual-based modelling study estimating the impact of maternity service delivery on health in Malawi
Source: Nat Commun. 2025 Apr 25;16:3925. doi: 10.1038/s41467-025-59060-2 (PMC12032021; doi:10.1038/s41467-025-59060-2)
Supplement: Supplementary file 2 — Supplementary Information [file 41467_2025_59060_MOESM2_ESM.pdf]

nature portfolio

Corresponding author(s): Dr Joseph H Collins

Last updated by author(s): 25/02/25

Reporting Summary

Nature Portfolio wishes to improve the reproducibility of the work that we publish. This form provides structure for consistency and transparency in reporting. For further information on Nature Portfolio policies, see our Editorial Policies and the Editorial Policy Checklist.

Statistics

For all statistical analyses, confirm that the following items are present in the figure legend, table legend, main text, or Methods section.

n/a	Confirmed	
☐	☒	The exact sample size (n) for each experimental group/condition, given as a discrete number and unit of measurement
☒	☐	A statement on whether measurements were taken from distinct samples or whether the same sample was measured repeatedly
☒	☐	The statistical test(s) used AND whether they are one- or two-sided<br>*Only common tests should be described solely by name; describe more complex techniques in the Methods section.*
☒	☐	A description of all covariates tested
☐	☒	A description of any assumptions or corrections, such as tests of normality and adjustment for multiple comparisons
☐	☒	A full description of the statistical parameters including central tendency (e.g. means) or other basic estimates (e.g. regression coefficient) AND variation (e.g. standard deviation) or associated estimates of uncertainty (e.g. confidence intervals)
☒	☐	For null hypothesis testing, the test statistic (e.g. F, t, r) with confidence intervals, effect sizes, degrees of freedom and P value noted<br>*Give P values as exact values whenever suitable.*
☒	☐	For Bayesian analysis, information on the choice of priors and Markov chain Monte Carlo settings
☒	☐	For hierarchical and complex designs, identification of the appropriate level for tests and full reporting of outcomes
☒	☐	Estimates of effect sizes (e.g. Cohen's d, Pearson's r), indicating how they were calculated

Our web collection on statistics for biologists contains articles on many of the points above.

Software and code

Policy information about availability of computer code

Data collection	No software was used
Data analysis	Data analysis was conducted using Python (v3.8.18) and Pandas (v2.0.3) data analysis library.

For manuscripts utilizing custom algorithms or software that are central to the research but not yet described in published literature, software must be made available to editors and reviewers. We strongly encourage code deposition in a community repository (e.g. GitHub). See the Nature Portfolio guidelines for submitting code & software for further information.

Data

Policy information about availability of data

All manuscripts must include a data availability statement. This statement should provide the following information, where applicable:

- Accession codes, unique identifiers, or web links for publicly available datasets
- A description of any restrictions on data availability
- For clinical datasets or third party data, please ensure that the statement adheres to our policy

Documentation for all data sources, model parameters, and model assumptions have been provided as supplementary information. Additional documentation relating to the model is available online (https://www.tlomodel.org/writeups.html).

Research involving human participants, their data, or biological material

Policy information about studies with human participants or human data. See also policy information about sex, gender (identity/presentation), and sexual orientation and race, ethnicity and racism.

Reporting on sex and gender	This is a modeling study which does not involve human participants. Where data previously collected from human participants was used in model development this has been noted.
Reporting on race, ethnicity, or other socially relevant groupings	This is a modeling study which does not involve human participants. Where data previously collected from human participants was used in model development this has been noted.
Population characteristics	This is a modeling study which does not involve human participants. Where data previously collected from human participants was used in model development this has been noted.
Recruitment	This is a modeling study which does not involve human participants. Where data previously collected from human participants was used in model development this has been noted.
Ethics oversight	This is a modeling study which does not involve human participants. Where data previously collected from human participants was used in model development this has been noted.

Note that full information on the approval of the study protocol must also be provided in the manuscript.

Field-specific reporting

Please select the one below that is the best fit for your research. If you are not sure, read the appropriate sections before making your selection.

☒ Life sciences ☐ Behavioural & social sciences ☐ Ecological, evolutionary & environmental sciences

For a reference copy of the document with all sections, see nature.com/documents/nr-reporting-summary-flat.pdf

Life sciences study design

All studies must disclose on these points even when the disclosure is negative.

Sample size	Modelled sample size was set at a model-real-population ratio in 2010 of 1:58. No formal sample size caluculation was performed. This sample size was determined based on initial exploration of the stochastic variation of model outputs at smaller sample sizes given that the some of the studies primary outcome (e.g. maternal mortality) remain rare in the modelled population.
Data exclusions	This study does not involve data exclusions
Replication	As this is a modelling study and any inherent stochastic variation in the model is controlled with a random seed this study is entirely replicable by running the model via the associated scenario files and analysis scripts. These files are freely available online as refereced in the main manuscript.
Randomization	No randomization was inolved in this study. As this is a modelling study, we compare outcomes at the population level between identical simulated populations following a given intervention and therefore there is no need to control for differences between the populations themselves.
Blinding	No blinding was involved in this study as the study is a modelling study.

Reporting for specific materials, systems and methods

We require information from authors about some types of materials, experimental systems and methods used in many studies. Here, indicate whether each material, system or method listed is relevant to your study. If you are not sure if a list item applies to your research, read the appropriate section before selecting a response.

Materials & experimental systems

n/a	Involved in the study
☒	☐ Antibodies
☒	☐ Eukaryotic cell lines
☒	☐ Palaeontology and archaeology
☒	☐ Animals and other organisms
☒	☐ Clinical data
☒	☐ Dual use research of concern
☒	☐ Plants

Methods

n/a	Involved in the study
☒	☐ ChIP-seq
☒	☐ Flow cytometry
☒	☐ MRI-based neuroimaging

Plants

Seed stocks

Report on the source of all seed stocks or other plant material used. If applicable, state the seed stock centre and catalogue number. If plant specimens were collected from the field, describe the collection location, date and sampling procedures.

Novel plant genotypes

Describe the methods by which all novel plant genotypes were produced. This includes those generated by transgenic approaches, gene editing, chemical/radiation-based mutagenesis and hybridization. For transgenic lines, describe the transformation method, the number of independent lines analyzed and the generation upon which experiments were performed. For gene-edited lines, describe the editor used, the endogenous sequence targeted for editing, the targeting guide RNA sequence (if applicable) and how the editor was applied.

Authentication

Describe any authentication procedures for each seed stock used or novel genotype generated. Describe any experiments used to assess the effect of a mutation and, where applicable, how potential secondary effects (e.g. second site T-DNA insertions, mosiacism, off-target gene editing) were examined.
