# Supplementary material for: An individual-based modelling study estimating the impact of maternity service delivery on health in Malawi
Source: Nat Commun. 2025 Apr 25;16:3925. doi: 10.1038/s41467-025-59060-2 (PMC12032021; doi:10.1038/s41467-025-59060-2)
Supplement: Supplementary file 3 — Supplementary Information [file 41467_2025_59060_MOESM3_ESM.pdf]

nature portfolio

Peer Review File

An individual-based modelling study estimating the impact of maternity service delivery on health in Malawi

Corresponding Author: Dr Joseph Collins

This file contains all reviewer reports in order by version, followed by all author rebuttals in order by version.

Version 0:

Reviewer comments:

Reviewer #1

(Remarks to the Author)
Thanks for the opportunity to review this paper. I found it to be well-written, clear, and the topic is interesting. However I am not sure whether there is a substantially new contribution to the literature, as it identifies important challenges (utilization alone is insufficient; it will be very hard -- if not impossible -- for many countries to meet their SDG targets; etc.) that have already been established in other papers. I feel that this particular analysis would be informative to policymakers in Malawi, but was not convinced of its broader impact. I do feel that a formal analysis of the trade-offs of pursuing investments into approaches that increase coverage vs quality for different groups (for example, "should" we invest in option A to increase coverage of interventions for neonates vs option B to improve quality of interventions for mothers) could be useful, but this would constitute an entirely different analysis (cost-benefit or similar) and a new paper, in my opinion.

(Remarks on code availability)

Reviewer #2

(Remarks to the Author)
The manuscript presents a detailed and comprehensive stochastic individual-based simulation model of maternal and perinatal health in Malawi. Notable results include the finding that current service delivery averts a significant number of maternal and neonatal deaths and DALYs. The model predicts substantial improvements in population health if higher coverage of high-quality services is achieved. Of particular interest is the finding that no single scenario alone was sufficient to meet SDG target 3.1, highlighting the need for a system-wide approach to improve maternal health.

The conclusions and claims made in the manuscript are generally well-supported by the simulation results, the methodology is sound and described in detail. The supplementary material is extensive

Recommendations (all minor):
- In Table 5, add a column which shows the SQ prevalence of all the interventions
- I find Figures 3 and 4 illegible. Consider alternate visualizations for these results.
- Figure 1a shows a sharp drop in the modeled maternal mortality ratio between 2014 and 2015 (and between 2010-2011). Is this drop primarily due to the difference in assumptions used between pre- and post- the Malawi 2014-2015 DHS? If not, what in the model most explains these drops?
o If it is due to the difference in assumptions, consider estimating an odds ratio that modifies the primary parameter (i.e. odds_will_attend_pnc) by year of birth in addition to the socio-demographic parameters, this would potentially lead to smoother estimates.
- The supplement notes that the referral process is only in place for individuals who deliver at a health center and require a CEmONC intervention. However, if a needed BEmONC intervention is modeled as not being available because of lack of health worker competence or lack of necessary consumables, shouldn’t this also trigger a referral process?
- How do the assumptions and results compare with those in the Lives Saved Tool?
- Perhaps it is included somewhere in the Appendix, but it is unclear to me if or how the probability of care-seeking for intrapartum and postpartum care is informed by care-seeking and the quality of the care received in the prior stages (antenatal and intrapartum care). This should be made more explicit in the methods section.

- Are probabilities of health worker competence and consumable availability independent or conditional on one another? I.e. if health worker is not trained in providing one intervention, is it more likely they are not trained in another?
- In the limitations section, examples of missing maternal and perinatal health conditions and interventions are clearly listed. However, it is worth noting more broadly that the model is by necessity a simplification of reality and cannot incorporate all possible parameters. Beyond those listed, the lack of consideration of public versus private facilities, the degree of delay depending on transportation availability, interventions such as counseling (which may affect other parameters such as IFA adherence), etc, are not considered.
- Given the emphasis on the 'whole system', it seems worth developing a scenario in which all three interventions are set to have an increase in coverage and quality.

(Remarks on code availability)

Version 1:

Reviewer comments:

Reviewer #1

(Remarks to the Author)

(Remarks on code availability)

Reviewer #2

(Remarks to the Author)
Thank you for the revision to this manuscript. I have found the revisions, particularly the inclusion of the additional concurrent scenarios, to have substantially strengthened the conclusions of study. The authors have adequately addressed all of my concerns and I have no additional feedback.

(Remarks on code availability)

Reviewer #3

(Remarks to the Author)
General Comments:
Thank you for the opportunity to review this paper. We believe it presents an excellent individual-based simulation model, meticulously designed to assess the impact of improving antenatal, intrapartum, and postnatal services in Malawi on reducing maternal and neonatal mortality rates, aligned with the SDG 3.1 and 3.2 goals. The results clearly demonstrate the positive health effects of the current coverage of these services in Malawi and predict that improving coverage could lead to a maximum reduction in stillbirth, maternal, and neonatal mortality rates by 10%, 52%, and 57%, respectively. The topic is highly relevant, and the paper is well-organized and well-written. We are pleased to provide comments that we hope will further enhance this valuable study. Below are our detailed comments.

Major comments:

Comment 1: Regarding the intuitive impression, we believe the model calibration does not perform well for maternal and neonatal mortality rates as shown in Figure 1. The maternal mortality rate for 2010 seems to be overestimated, while the rate for 2015 is underestimated. Similarly, neonatal mortality rates are consistently underestimated. These two calibration targets are crucial since they represent key model outputs. Poor calibration in these areas raises concerns about the reliability and accuracy of the predicted model outcomes.

Comment 2: For an individual-based simulation model, it can accurately represent each individual in the system along with their characteristics and event occurrences throughout the simulation process. However, this approach requires highly precise data to initialize the status of individuals at the model's start. For instance, the authors mention that the model's individuals are characterized by demographic (e.g., age, sex, region of residence) and lifestyle (e.g., smoking status) attributes (Methods, third paragraph). Could the authors please clarify how these characteristics were assigned to the individuals within the model? Additionally, readers would benefit from a more detailed explanation of this process, particularly regarding the data sources used for sampling each characteristic and the results of these sampling procedures.

Comment 3: We believe the authors should clarify the nature of the simulated population in the model. Is the simulation based on the entire population or only on pregnancies? If it encompasses the entire population, how did the authors model the occurrence of pregnancies during each model cycle throughout the time horizon? On the other hand, if the simulation focuses solely on pregnancies, it implies that the incidence of pregnancy is modeled as independent for each year. This would suggest that the results are generated on a year-by-year basis, where differences across years would be influenced

only by the parameters specific to each year, with no continuity between them. In this case, the authors should provide a detailed presentation of the parameter differences across years in the supplementary material, while clearly indicating the corresponding differences in the output results.

Comment 4: The authors applied three sets of parameters during three distinct phases (2010-2014, 2015-2022, and 2023-2030), with corresponding results illustrated in Figure S80. From an intuitive perspective, the outcomes resemble a three-stage function, particularly in the predicted phases where the outcomes appear quite stable. Based on this, one could argue that if the intervention achieves the SDG goals by 2030, it would also achieve them by 2023, which raises questions about the model's progression. While this may be reasonable within the confines of the simulation, it is difficult to justify such an outcome in real-world scenarios. This undermines the objective of achieving reductions of maternal and neonatal mortality rates by 2030, potentially rendering the study's goal of achieving reductions by that year meaningless. We recommend that the authors recheck these parameter sets and reassess whether the model assumptions are truly appropriate and reflective of real-world dynamics.

Comment 5: The authors performed model calibrations. From a technical perspective, it would be helpful if the authors could clearly present the estimated parameters obtained during this calibration process, distinguishing them from the parameters sourced from other references. This would provide clarity on which parameters were derived through calibration versus those sourced externally, offering a more comprehensive understanding of the model's foundation and how it was fine-tuned to fit the data.

Minor comments:
1. The authors referred to SDG goals 3.1 and 3.2, citing reference 34 (https://www.unfpa.org/resources/transforming-our-world-2030-agenda-sustainable-development). As per the reference, the exact targets are:
"3.1 By 2030, reduce the global maternal mortality ratio to less than 70 per 100,000 live births.
3.2 By 2030, end preventable deaths of newborns and children under 5 years of age, with all countries aiming to reduce neonatal mortality to at least as low as 12 per 1,000 live births and under-5 mortality to at least as low as 25 per 1,000 live births."

However, in the Results section (ii), second paragraph, the authors state: "As evident from Table 2, only the scenario 'All services max.' led to an average MMR during the intervention period below the SDG 3.1 target of 140 maternal deaths per 100,000 live births." This threshold differs from the official SDG 3.1 target of less than 70 maternal deaths per 100,000 live births. If there are specific reasons for using a different threshold, the authors should clarify them to avoid confusion for the readers and ensure alignment with SDG targets.

Moreover, the SDG 3.1 target specifically aims to reduce the maternal mortality ratio (MMR) by 2030. However, the authors report the *average* MMR during the period from 2023 to 2030 as their main result. It would be helpful if the authors could clarify why they focus on the average over this period, rather than reporting the MMR specifically in the year 2030, as the SDG target is for that specific year. This distinction is important to ensure that the study's findings align more closely with the actual SDG goals.
2. As Harold C. Sox mentions in *Medical Decision Making* (3rd edition), "The terms calibration and validation are not used consistently in the literature; although they are sometimes used interchangeably, they are not synonymous. We won't dive deeper into these two terms because they can be hard to remember unless you actually need to use them." In light of this, the authors should strive to use terminology that is appropriate and consistent throughout the manuscript to enhance the reader's understanding and experience.
3. The authors calculated the total burden of maternal and neonatal health in Malawi, which depends on the number of pregnancies each year as well as the model's initialization parameters. Please provide the detailed numbers used in these calculations to enhance the transparency and reproducibility of the study.
4. Please provide the detailed model procedures for the model cycle and time horizon. This information will help clarify how the model operates over time and how different phases are implemented within the simulation.

(Remarks on code availability)

Reviewer #5

(Remarks to the Author)
I co-reviewed this manuscript with one of the reviewers who provided the listed reports. This is part of the Nature Communications initiative to facilitate training in peer review and to provide appropriate recognition for Early Career Researchers who co-review manuscripts.

(Remarks on code availability)

Version 2:

Reviewer comments:

Reviewer #3

(Remarks to the Author)
Thank you for the detailed response, which addressed many of our concerns. However, we still have some reservations regarding the methodology, as outlined below.

For Comment 1, the authors explained that “The MMR value during the initial year of simulation (2010) is inflated because pregnancies are only modeled to occur as the simulation progresses, resulting in fewer births in that year.” However, it remains unclear why fewer births in that year would result in a higher MMR value. More importantly, the quality of model calibration is predominantly influenced by technical aspects, specifically the calibration targets, parameters, and the loss function. While this may not be the authors’ primary area of expertise, the observed calibration issues warrant a deeper investigation to identify the root cause of the suboptimal performance.

For Comment 2, considering that the TLO modeling framework was developed by a team of researchers, it is acceptable that its initiation is not described in detail within this manuscript. However, the authors should explicitly acknowledge the contributions of that team to this work. By the way, it seems like to draw legs on a snake for reply “Importantly, ... intervention period (2023)”, because refining the initiation is to ensure the model simulates from an accurate startline, not to reflect future scenarios

For Comment 3, it is commendable that the probability of pregnancy in the simulated population is derived from the contraception model within the TLO framework. Thank you for providing a brief description to clarify this aspect.

For Comment 4, it would be a meaningful contribution if the authors focused on presenting the potential health gains that could be achieved during the period from 01/01/2023 to 31/12/2030. Thanks for your responses.

For Comment 5, it is commendable to highlight the names of each parameter derived through calibration. However, we could not locate these changes in the appendix. Could the authors consider adding a separate table to clearly list the calibrated parameters for better clarity?

For Minor Comment 4, although the model may include various modules with differing model cycles, or some may utilize event-based structures, we believe it is essential to clarify these elements. Specifically, it would be helpful to detail what modules are included, their respective cycles, and the type of structure they use. For example, as referenced in your response to Comment 3, the parameters in the contraception model are applied on a monthly timestep throughout the modeled time horizon.

(Remarks on code availability)

Reviewer #5

(Remarks to the Author)
I co-reviewed this manuscript with one of the reviewers who provided the listed reports. This is part of the Nature Communications initiative to facilitate training in peer review and to provide appropriate recognition for Early Career Researchers who co-review manuscripts.

(Remarks on code availability)

Version 3:

Reviewer comments:

Reviewer #3

(Remarks to the Author)
The author has addressed our concerns. We appreciate the detailed response.

(Remarks on code availability)

Reviewer #5

(Remarks to the Author)
I co-reviewed this manuscript with one of the reviewers who provided the listed reports. This is part of the Nature Communications initiative to facilitate training in peer review and to provide appropriate recognition for Early Career Researchers who co-review manuscripts.

(Remarks on code availability)

Open Access This Peer Review File is licensed under a Creative Commons Attribution 4.0 International License, which permits use, sharing, adaptation, distribution and reproduction in any medium or format, as long as you give appropriate credit to the original author(s) and the source, provide a link to the Creative Commons license, and indicate if changes were made.
In cases where reviewers are anonymous, credit should be given to 'Anonymous Referee' and the source.
The images or other third party material in this Peer Review File are included in the article's Creative Commons license, unless indicated otherwise in a credit line to the material. If material is not included in the article's Creative Commons license and your intended use is not permitted by statutory regulation or exceeds the permitted use, you will need to obtain permission directly from the copyright holder.
To view a copy of this license, visit https://creativecommons.org/licenses/by/4.0/

Response to reviewers' comments

Reviewer #1 (Remarks to the Author):

Thanks for the opportunity to review this paper. I found it to be well-written, clear, and the topic is interesting. However I am not sure whether there is a substantially new contribution to the literature, as it identifies important challenges (utilization alone is insufficient; it will be very hard -- if not impossible -- for many countries to meet their SDG targets; etc.) that have already been established in other papers. I feel that this particular analysis would be informative to policymakers in Malawi but was not convinced of its broader impact.

I do feel that a formal analysis of the trade-offs of pursuing investments into approaches that increase coverage vs quality for different groups (for example, "should" we invest in option A to increase coverage of interventions for neonates vs option B to improve quality of interventions for mothers) could be useful, but this would constitute an entirely different analysis (cost-benefit or similar) and a new paper, in my opinion.

We thank the Reviewer for their review of the manuscript and appreciate that they recognise the paper is clear, well-written and on an interesting topic. We also appreciate that the reviewer concurs with us that our analysis is informative to policymakers in Malawi and, by implication, that our results can help to lead to improved maternal and newborn health in the country. We contend that the research also has wider relevance beyond Malawi, as detailed below.

We agree that future analyses estimating the cost-effectiveness of alternative scenarios is particularly important to guide policy and improve outcomes. However, given that the costs of policies are uncertain, especially as there are alternative approaches to policy implementation, we have focused on the impacts of said policies on health outcomes. Work is ongoing within our group to incorporate costs into the model and in future we are likely to have more reliable evidence on costs (including from immediate and downstream consequences).

Importantly, our study goes beyond simply reiterating previous thinking relating to the predicted effects of changes in maternity service coverage and/or quality on health outcomes. This is because our model mechanistically simulates the effect of these policy changes within the population, along with the downstream effect of such changes, across a diverse range of health outcomes in the context of the wider epidemiological and health systems. As such, we have more completely quantified the predicted effect of these changes in Malawi on a range of outcomes than would not have been possible through the application of other available tools (e.g. the Lives Saved Tool, the MANDATE model, Shrime et al. (2019, cited in text), Ward et al. (2023, cited in text)). Notably our model estimates health outcomes, as opposed to surrogate outcomes and our analyses considered a long time horizon to capture future effects of policy change. Whilst the services themselves are not yet fully costed; we do explore

the resource implication by quantifying changes in service use through appointment numbers.

Furthermore, this has not been achieved before, given that ours is the first individual-based model of maternal and newborn health to sit within a whole-system, all-disease framework, The Thanzi La Onse model (https://www.tlomodel.org/), which is also the first of its kind. The novelty of this approach is supported by a recent scoping review conducted by Collins et al. (2023) cited in the main text, currently under review at PLOS ONE, a preprint of which is available here - https://doi.org/10.1101/2023.12.16.23300088. This review highlighted a significant gap in the literature regarding individual-based or systems thinking approaches to modelling of maternal and newborn health services and did not identify any models which represented the wider health system.

Vitally, whilst our approach is country-specific and is rooted firmly in data sourced from Malawi, where available, the maternal and newborn conditions we studied and the intervention effects are not specific to Malawi and whilst our specific effect estimates relate closely to Malawi the overall findings are likely to be relevant to multiple other settings in the region. As such we feel our research aligns closely with the aims of the 'Health in Africa' collection in your journal in supporting African nations in achieving the SDGs goals.

Reviewer #2 (Remarks to the Author):

The manuscript presents a detailed and comprehensive stochastic individual-based simulation model of maternal and perinatal health in Malawi. Notable results include the finding that current service delivery averts a significant number of maternal and neonatal deaths and DALYs. The model predicts substantial improvements in population health if higher coverage of high-quality services is achieved. Of particular interest is the finding that no single scenario alone was sufficient to meet SDG target 3.1, highlighting the need for a system-wide approach to improve maternal health.

The conclusions and claims made in the manuscript are generally well-supported by the simulation results, the methodology is sound and described in detail. The supplementary material is extensive

We thank the Reviewer for their time and their provided comments. Please see our responses below.

Recommendations (all minor):
- In Table 5, add a column which shows the SQ prevalence of all the interventions

The coverage of each service (e.g. antenatal care) in the SQ scenario is provided in Table 1 where the scenarios are outlined. We do not currently output the average coverage of each of the listed interventions in Table 5 as the simulation runs. The model is not explicitly calibrated to the population coverage of each intervention which

constitutes one of the wider health services due to limited reliable data from Malawi. Instead, the probability that each intervention being delivered is the product of the probability of care seeking (derived from calibration to population level service coverage), consumable availability at the facility level (sourced from Malawi specific data sources including the Harmonised Health Facilities Assessment 2018/19 the open LMIS) and clinical competence. This is outlined within the supplementary material and the main text.

- I find Figures 3 and 4 illegible. Consider alternate visualizations for these results.

We have increased the font size to hopefully improve legibility of the conditions listed on the x axis of both these figures. Additionally we have rechecked the images resolution to ensure that on zooming in, headings can be read clearly. We hope that were the paper to be published the resolution would remain high enough for easy interpretation of these results. Whilst we would ideally have fewer plots on one figure, we are limited by the number of figures allowed by the journal. In addition we noted a labelling issue with one of the sub plots which has now been corrected.

- Figure 1a shows a sharp drop in the modeled maternal mortality ratio between 2014 and 2015 (and between 2010-2011). Is this drop primarily due to the difference in assumptions used between pre- and post- the Malawi 2014-2015 DHS? If not, what in the model most explains these drops? - If it is due to the difference in assumptions, consider estimating an odds ratio that modifies the primary parameter (i.e. odds_will_attend_pnc) by year of birth in addition to the socio-demographic parameters, this would potentially lead to smoother estimates.

We would like to emphasise that, given this drop occurs in the baseline period, after which the MMR can be seen stabilising, this change will not have impacted the results in any way. The initial drop between 2010 and 2011 shown in this figure is due to stabilisation of the population as the simulation initialises. Reading the change between 2014 and 2015, within the supplementary material we explain that we used two parameter sets to achieve calibration to the DHS mortality estimates from the two surveys. These parameters are switched when the simulation reaches 2015. The second parameter set is therefore used for the remainder of the simulation. As such these drops in MMR are therefore explained by increased coverage in essential maternal health services (ANC, delivery care, PNC) as reported in the 2015 DHS and any other temporal changes in disease incidence or health system factors.

- The supplement notes that the referral process is only in place for individuals who deliver at a health center and require a CEmONC intervention. However, if a needed BEmONC intervention is modeled as not being available because of lack of health worker competence or lack of necessary consumables, shouldn’t this also trigger a referral process?

We were not able to identify reliable data on the referral of patients in Malawi who cannot receive care due to poor quality (e.g. no consumables, lacking trained staff etc.). We were unable to identify if this was common practice and how such referrals worked (i.e. are patients referred only to higher level facilities or to facilities of the same level which may be more appropriately stocked). We do however know that referral is recommended based on acuity of condition as highlighted in Malawian national Standard Treatment Guidelines, which, as you mention, is included in the model. As such, we have chosen to assume that if care cannot be delivered due to quality or competence that the intervention will not be delivered. This likely does not reflect reality and is a limitation of our approach. In response to your comment we have now highlighted this in the limitation section of the manuscript.

- How do the assumptions and results compare with those in the Lives Saved Tool?

In the 6th paragraph of the discussion section we outline some of the key features of our approach that we believe differentiates our model from other commonly applied approaches. We have now added a sentence drawing additional comparison between our approach and LiST. Given the detail in which we discuss our own assumptions in the main text and supplementary material, and the availability of detailed documentation of the LiST tool available online, and the word count, we felt it was not necessary to compare our models in substantial detail.

Considering model results, our initial intention was to compare the outcomes of our model with similar scenarios within the Lives Saved Tool (LiST). However we were unable to construct scenarios within LiST that we felt were sufficiently comparable to the ones developed for this study. As such, and given the word limit, we have opted not to attempt to compare our results to results from scenarios modelled using LiST.

- Perhaps it is included somewhere in the Appendix, but it is unclear to me if or how the probability of care-seeking for intrapartum and postpartum care is informed by care-seeking and the quality of the care received in the prior stages (antenatal and intrapartum care). This should be made more explicit in the methods section.

At present within the model, the quality of previous ‘interactions’ within the health system does not impact the probability of future care seeking. As stated in the manuscript and supplementary material the probability of individual care seeking is calculated using a regression model of DHS data. Quality of care experience at previous appointments was not included as a covariate in the model. Furthermore, due to the absence of data, we do not capture other dimensions of quality of care which may impact future care seeking such as respectful care. We have now clarified this in the main text.

- Are probabilities of health worker competence and consumable availability independent or conditional on one another? I.e. if health worker is not trained in providing one intervention, is it more likely they are not trained in another?

Healthcare workers are not modelled at the individual level. We use probabilities that represent the average competence/availability of trained healthcare workers within a given healthcare interaction. These probabilities are independent. So if an individual requires treatment for both condition X and condition Y, the probability of treatment delivery for each of these conditions is calculated separately using the relevant parameters (including consumable availability). We have added a sentence to clarify this in the main text.

- In the limitations section, examples of missing maternal and perinatal health conditions and interventions are clearly listed. However, it is worth noting more broadly that the model is by necessity a simplification of reality and cannot incorporate all possible parameters. Beyond those listed, the lack of consideration of public versus private facilities, the degree of delay depending on transportation availability, interventions such as counseling (which may affect other parameters such as IFA adherence), etc, are not considered.

Thank you for this. We have now added a clarifying sentence to this effect within the limitations section.

- Given the emphasis on the 'whole system', it seems worth developing a scenario in which all three interventions are set to have an increase in coverage and quality.

Thank you for your comment. We agree that additional scenarios estimating the impact of increased coverage/quality of all services concurrently would strengthen the paper and better contextualise the results relating the individual services. As such we have ran four additional scenarios estimating the impact of to improved coverage, coverage and quality, maximisation and removal of all services together to align with the format of the other groups of scenarios described in Table 1. We have added these results to the relevant tables of the manuscript and updated the abstract, main text and figures within accordingly. Thank you for this suggestion.

“**Reviewer #1’** confidentially maintain their initial concerns, and thus we strongly encourage the addition of the cost analyses recommended by them in previous review.

We thank the Reviewer for their time reviewing our manuscript again and agree that there is a need to connect the results of our analyses with the contemporary health-economics literature to better contextualise our findings.

Therefore, for each scenario in which a meaningful reduction in DALYs was detected compared to the Status Quo (e.g. those for which the 95%CI of the mean difference between scenarios does not span 0), we have now calculated the maximum amount of money (USD) which could be invested by funders of the Malawian health system (i.e. government, development assistance for health) to achieve the modelled changes within the health system which is still consistent with such changes being cost effective. We refer to this as the maximum ability to pay, drawing on the health economics literature (Mohan et al., reference below).

Given that health economic evaluation assumes an intervention should be delivered within a health system if the intervention generates more health benefits than what could be produced with the same resources elsewhere within the system with the same resources (i.e., health benefits exceed health opportunity costs), we assume that for every unit of ill-health averted due to an intervention (DALYs), the system should not pay more than the cost at the margin at which it is already able to avert a DALY from existing interventions. Therefore the maximum ability to pay for each scenario was calculated as the product of the incremental health impact of a scenario compared to the Status Quo (DALYs averted) and the cost-effectiveness threshold (CET) for Malawi, representing marginal productivity.

We compared these values to the projected total national health spending in Malawi during 2023 to 2030, which was calculated using population projections from the SQ scenario and projected health spending per capita during 2023-2030 in Malawi (measured in 2017 USD) estimated by Dieleman et al. (reference below). We used a CET value of $62.3 for Malawi (adjusted for inflation to compare with Dieleman et al’s estimate) as estimated from extensive analyses undertaken to inform Malawi's current Health Sector Strategic Plan III (2024-30).

We present the results of this new analysis within the results section and report both the maximum ability to pay values and the percentage of projected total health spending in Malawi that each value constitutes in Table S62 of the supplementary material. We have added a new paragraph to the discussion section outlining these results and briefly discussing the limitations of our approach.

References:

Mohan S, Revill P, Malvolti S, Malhame M, Sculpher M, Kaye PM. Estimating the global demand curve for a leishmaniasis vaccine: A generalisable approach based on global burden of disease estimates. PLoS Negl Trop Dis [Internet]. 2022;16(6):e0010471. Available from: https://pmc.ncbi.nlm.nih.gov/articles/PMC9232160/

Dieleman J, Sadat N, Chang A, Fullman N, Abbafati C, Acharya P et al. Trends in future health financing and coverage: future health spending and universal health coverage in 188 countries, 2016-40. Lancet [Internet]. 2018;391(10132):1783-1798. Available from: https://www.thelancet.com/journals/lancet/article/PIIS0140-6736(18)30697-4/fulltext#app-1

Reviewer #2 (Remarks to the Author):

Thank you for the revision to this manuscript. I have found the revisions, particularly the inclusion of the additional concurrent scenarios, to have substantially strengthened the conclusions of study. The authors have adequately addressed all of my concerns and I have no additional feedback.

We thank the Reviewer for their time reviewing our manuscript again and are glad they feel the study has been strengthened further.

Reviewer #3 (Remarks to the Author):

General Comments:

Thank you for the opportunity to review this paper. We believe it presents an excellent individual-based simulation model, meticulously designed to assess the impact of improving antenatal, intrapartum, and postnatal services in Malawi on reducing maternal

and neonatal mortality rates, aligned with the SDG 3.1 and 3.2 goals. The results clearly demonstrate the positive health effects of the current coverage of these services in Malawi and predict that improving coverage could lead to a maximum reduction in stillbirth, maternal, and neonatal mortality rates by 10%, 52%, and 57%, respectively. The topic is highly relevant, and the paper is well-organized and well-written. We are pleased to provide comments that we hope will further enhance this valuable study. Below are our detailed comments.

We thank the Reviewer for their time reviewing our manuscript and for highlighting the potential impact of our work. We have responded to the Reviewer's additional comments below.

Major comments:

Comment 1: Regarding the intuitive impression, we believe the model calibration does not perform well for maternal and neonatal mortality rates as shown in Figure 1. The maternal mortality rate for 2010 seems to be overestimated, while the rate for 2015 is underestimated. Similarly, neonatal mortality rates are consistently underestimated. These two calibration targets are crucial since they represent key model outputs. Poor calibration in these areas raises concerns about the reliability and accuracy of the predicted model outcomes.

We thank the reviewer for their comment regarding the model's calibration to mortality data. Whilst we appreciate that there is some difference between model outputs and the data presented in Figure 1, we believe that for the purpose of the analyses conducted here, the model calibrations are robust as explained below.

Considering first the yearly maternal mortality ratio (MMR) displayed in Figure 1a, we note that the 2010 value is generated following the first year during a model run. For simplicity, at initialisation of a given simulation we do not model any female individuals of reproductive age as being already pregnant on the first day of the simulation (01/01/2010 in this instance). Therefore, pregnancies can only occur from 01/01/2010 onwards meaning that there are fewer births in 2010 than subsequent years which inflates the MMR for that year due to a smaller denominator.

The suggested ‘underestimation’ of the MMR from 2015 onwards (compared to the DHS 2015/16 estimate) is attributable to a slight underestimation in the number of indirect maternal deaths (those “resulting from previous existing disease or disease that developed during pregnancy and not due to direct obstetric causes but were aggravated by the physiologic effects of pregnancy”) in the model. As shown in Figure S33, the model's calibration to the DHS MMR estimates after adjustment to represent only mortality attributable to direct obstetric causes (approximately 70% of total mortality) is very strong. As there is very little reliable existing data in Malawi to estimate the number of indirect maternal deaths by cause it would be challenging to calibrate the model beyond this.

Importantly, indirect deaths are caused by other disease models within the Thanzi La Onse (TLO) framework and each model which generates indirect deaths (e.g. HIV) is rigorously calibrated to epidemiological and health systems data (Hallett et al. (reference below)). We would also highlight that from 2011 onwards our estimate, and the associated uncertainty intervals, lie within the uncertainty range for the UN MMEIG and/or DHS estimates of MMR in Malawi with the closest agreement in 2020 prior to the start of the intervention (results) period of the simulation.

Considering the neonatal mortality rate (NMR) shown in Figure 1c we would highlight a similar point that not all deaths in the model during days 0-28 are driven by the maternal and newborn health model described in this manuscript. However, as shown in Figure 1d the model likely estimates the correct proportion of deaths by the leading causes. We note the large uncertainty estimates around the calibration data and that by 2022 alignment between the mean model output and the mean UN IGCME estimate is sufficiently close prior to the start of the intervention (results) period.

In response to your comments we have now added clarifying sentences/paragraphs to:

1. The legend of Figure 1 addressing the inflated 2010 MMR value
2. Sections 4.2.3.1.1 of the supplementary material addressing the inflated 2010 MMR value in Figure S33
3. Sections 4.2.3.1.1 of the supplementary material comparing the results presented in Figure 1a to Figure S33 and attributing underestimation of yearly total MMR to reduced indirect mortality in the model as described above.

Reference:

Hallett TB, Mangal TD, Tamuri AU, Arinaminpathy N, Cambiano V, Chalkley M, et al. Estimates of resource use in the public-sector health-care system and the effect of strengthening health-care services in Malawi during 2015-19: a modelling study (*Thanzi La Onse*). Lancet Glob Health [Internet]. 2024. ISSN 2214-109X. Available from: https://doi.org/10.1016/S2214-109X(24)00413-3.

Comment 2: For an individual-based simulation model, it can accurately represent each individual in the system along with their characteristics and event occurrences throughout the simulation process. However, this approach requires highly precise data to initialize the status of individuals at the model's start. For instance, the authors mention that the model's individuals are characterized by demographic (e.g., age, sex, region of residence) and lifestyle (e.g., smoking status) attributes (Methods, third paragraph). Could the authors please clarify how these characteristics were assigned to the individuals within the model? Additionally, readers would benefit from a more detailed explanation of this process, particularly regarding the data sources used for sampling each characteristic and the results of these sampling procedures.

We thank the Reviewer for their comment. We agree that we could improve the clarity of the description of our methodology further. We have now added a paragraph to the Methods section briefly describing the key variables generated by the components of the TLO model which initialise the baseline demographic and lifestyle characteristics and the data sources used, along with additional references to additional publications and/or model documentation which details all the data sources used to characterise the population.

As discussed above, the model described in this manuscript is part of the TLO modelling framework which has been developed by a team of researchers with different areas of expertise. The components of the TLO model which initialise the baseline demographic and lifestyle characteristics were not developed as part of this research study and therefore have not been described in detail within this manuscript. Importantly, we have opted not to present the distribution of demographic or lifestyle characteristics of the baseline population in 2010 given that such results would not be reflective of the demographics of

the population of women of reproductive age at the beginning of the intervention period (2023).

Comment 3: We believe the authors should clarify the nature of the simulated population in the model. Is the simulation based on the entire population or only on pregnancies? If it encompasses the entire population, how did the authors model the occurrence of pregnancies during each model cycle throughout the time horizon? On the other hand, if the simulation focuses solely on pregnancies, it implies that the incidence of pregnancy is modeled as independent for each year. This would suggest that the results are generated on a year-by-year basis, where differences across years would be influenced only by the parameters specific to each year, with no continuity between them. In this case, the authors should provide a detailed presentation of the parameter differences across years in the supplementary material, while clearly indicating the corresponding differences in the output results.

We have now clarified under the heading '*Analysis plan - estimating the impact of maternity service delivery'* that the population modelled in this analysis is not a cohort of pregnant women but rather an 'entire' population designed to be representative of Malawi. This decision was made to preserve the functionality of other TLO components which generate disease incidence based on transmission between men and women and drive maternal outcomes (e.g. HIV).

Regarding the probability of pregnancy in the simulated population, in the section *'Maternal and perinatal health'* within the manuscript's Methods section we outlined that the probability a woman can become pregnant during the simulation is informed by their contraceptive use and fertility, determined by the contraception model within the TLO framework. As cited in-text this model has previously been described within a publication by Colbourn et al. (reference below) which is referenced in the text for readers to refer to and contains a detailed overview of the model structure, assumptions, data used and calibration. To aid reader's understanding of our methods we have now briefly described how the contraception model determines risk of pregnancy in women of reproductive age and the relevant data sources used to derive parameters and for model calibration (DHS contraception calendar, World Population Prospects medium variant population projections). As stated in this added description, parameters are applied on a monthly timestep throughout the modelled time horizon.

References:

Colbourn T, Janoušková E, Li Lin I, Collins J, Connolly E, Graham M, et al. Modeling Contraception and Pregnancy in Malawi: A Thanzi La Onse Mathematical Modeling Study. Stud Fam Plann [Internet]. 2023;54(4):585–607. Available from: https://onlinelibrary.wiley.com/doi/full/10.1111/sifp.12255

Comment 4: The authors applied three sets of parameters during three distinct phases (2010-2014, 2015-2022, and 2023-2030), with corresponding results illustrated in Figure S80. From an intuitive perspective, the outcomes resemble a three-stage function, particularly in the predicted phases where the outcomes appear quite stable. Based on this, one could argue that if the intervention achieves the SDG goals by 2030, it would also achieve them by 2023, which raises questions about the model's progression. While this may be reasonable within the confines of the simulation, it is difficult to justify such an outcome in real-world scenarios. This undermines the objective of achieving reductions of maternal and neonatal mortality rates by 2030, potentially rendering the study's goal of achieving reductions by that year meaningless. We recommend that the authors recheck these parameter sets and reassess whether the model assumptions are truly appropriate and reflective of real-world dynamics.

Within our analysis we have opted to model changes to service delivery occurring on 1/1/2023. We do not model a phased or gradual implementation of these changes over the intervention period of 2023 to 2030. This is because the rate at which such changes would or could be implemented within Malawi is unknown. However, as described in our methodology, we represent the potential health gains which could be achieved between 01/01/2023 and 31/12/2030 if the coverage/quality changes described in each scenario (Table 1) were to occur from this date. This is clearly an abstraction of reality; however this does not impact our conclusions presented in this paper. Whilst outcomes such as the 'total' DALYs averted during the intervention period would be lower in a 'phased approach', the 'final' mortality rate in 2030 would be the same if we had modelled a gradual increase in coverage/quality over time (e.g. reaching desired coverage or quality by 2029).

Our approach, where we have taken the average mortality rates across the intervention period following the change in intervention coverage being 'switched on', does however

significantly increase the power of our analysis and the accuracy of our estimates given we are averaging data for outcomes which are reasonably rare in our modelled population (e.g. maternal deaths) over 7 years (inclusive of multiple runs).

We agree that we must remain mindful regarding the interpretation of our results. As such we have added additional detail to the methodology section of the manuscript to clarify why we have made the decisions discussed above to aid interpretation of our results.

Comment 5: The authors performed model calibrations. From a technical perspective, it would be helpful if the authors could clearly present the estimated parameters obtained during this calibration process, distinguishing them from the parameters sourced from other references. This would provide clarity on which parameters were derived through calibration versus those sourced externally, offering a more comprehensive understanding of the model's foundation and how it was fine-tuned to fit the data.

We thank the Reviewer for their comment. We have provided a comprehensive description of the process through which each of the modelled parameters was obtained within the supplementary material tables and have now underlined the names of each of the parameters which were derived through calibration to clarify this. We have also added a sentence explaining this distinction within the supplementary material starting from table S8 and to section 1.2.1 of the supplementary material to this effect.

Minor comments:

1. The authors referred to SDG goals 3.1 and 3.2, citing reference 34 (https://www.unfpa.org/resources/transforming-our-world-2030-agenda-sustainable-development). As per the reference, the exact targets are:

"3.1 By 2030, reduce the global maternal mortality ratio to less than 70 per 100,000 live births.

3.2 By 2030, end preventable deaths of newborns and children under 5 years of age, with all countries aiming to reduce neonatal mortality to at least as low as 12 per 1,000 live births and under-5 mortality to at least as low as 25 per 1,000 live births."

However, in the Results section (ii), second paragraph, the authors state: “As evident from Table 2, only the scenario ‘All services max.’ led to an average MMR during the intervention period below the SDG 3.1 target of 140 maternal deaths per 100,000 live births.” This threshold differs from the official SDG 3.1 target of less than 70 maternal deaths per 100,000 live births. If there are specific reasons for using a different threshold, the authors should clarify them to avoid confusion for the readers and ensure alignment with SDG targets.

Thank you for drawing our attention to this and we apologise for the confusion regarding the reported SDG indicator. When presenting the target of 140 per 100,000 we were in fact referring to the mortality targets presented in the WHO Ending Preventable Maternal Mortality guidelines (https://iris.who.int/bitstream/handle/10665/153544/9789241508483_eng.pdf?sequence=1) which, whilst developed to align with the SDG 3.1 indicator of 70 per 100,000 live births include an additional ‘supplementary national target’ of no country exceeding 140 per 100,000 live births is also included (please see page 6 of the linked document).

We have now updated the manuscript to reflect that no scenario led to a sufficient reduction in MMR to achieve SDG 3.1 and that only one scenario met the WHO EPMM supplementary national target of 140 per 100,000.

Moreover, the SDG 3.1 target specifically aims to reduce the maternal mortality ratio (MMR) by 2030. However, the authors report the *average* MMR during the period from 2023 to 2030 as their main result. It would be helpful if the authors could clarify why they focus on the average over this period, rather than reporting the MMR specifically in the year 2030, as the SDG target is for that specific year. This distinction is important to ensure that the study’s findings align more closely with the actual SDG goals.

As discussed above (response to Comment 3) we have modelled a simulated population representative of Malawi’s entire population and not a cohort of pregnant women. The initial population of individuals was modelled with a model-to-real population ratio in 2010 of 1:58. We have opted to average the yearly mortality/morbidity rates during the intervention period because of the inherent variability in the yearly mortality rates (evident in Figure S80 A-D). This variability is driven by the inherent stochasticity of the model and

that maternal/neonatal deaths remain relatively rare events within a modelled population of this size. We discuss the benefits of taking the average of the yearly values as our primary result in response to Comment 4.

2. As Harold C. Sox mentions in *Medical Decision Making* (3rd edition), "The terms calibration and validation are not used consistently in the literature; although they are sometimes used interchangeably, they are not synonymous. We won't dive deeper into these two terms because they can be hard to remember unless you actually need to use them." In light of this, the authors should strive to use terminology that is appropriate and consistent throughout the manuscript to enhance the reader's understanding and experience.

Within our manuscript’s supplementary material we provide our working definition for the term validation as “substantiation that a model within its domain of applicability possesses a satisfactory range of accuracy consistent with the intended application of the model” (Sargent, reference below). We go on to describe two complementary, but distinct, components of validation - face validation, the process in which individuals with expert domain knowledge related to the system of interest are asked to determine if the model and/or its behaviour are reasonable given their expertise (Sargent, reference below), and model calibration (sometimes referred to as operational validation) in which the model is fit to data to improve confidence in the model’s predictions. We have opted to use the term model calibration over operational validation due to its wider use in the academic literature. In the main manuscript we have opted to discuss validation of the model through the process of calibration to data, as opposed to face validation of the model which is described in the supplementary material.

To improve clarity we have now changed the title of the section to ‘Model calibration’.

<u>References:</u>

Sargent RG. Verification and validation of simulation models. Journal of Simulation [Internet]. 2013;7(1):12–24. Available from: https://www.tandfonline.com/action/journalInformation?journalCode=tjsm20

3. The authors calculated the total burden of maternal and neonatal health in Malawi, which depends on the number of pregnancies each year as well as the model's initialization parameters. Please provide the detailed numbers used in these calculations to enhance the transparency and reproducibility of the study.

To further aid interpretation of our results, we have now added a table to the supplementary material (S61) describing the number of pregnancies, births, stillbirths, maternal deaths and neonatal deaths per year between 2023-2030 for the Status Quo scenario.

The version of the TLO model which was used to conduct the analyses presented in this manuscript is available open access as reference in text (doi: 10.5281/zenodo.10474177). This includes the scripts used to enact the parameter changes defined in the scenarios (Table 1) and the scripts used to output all of the primary and secondary results reported here and in the supplementary material. Furthermore, all model parameters are available within the parameter tables provided in the supplementary material or within the documentation of other components of the TLO model which have been referenced within the manuscript (this includes the TLO website (https://www.tlomodel.org/) where documentation of all developed and in development models is available).

4. Please provide the detailed model procedures for the model cycle and time horizon. This information will help clarify how the model operates over time and how different phases are implemented within the simulation.

Within the manuscript we present the time horizon for our analysis (2010-2030, with changes enacted on 01/01/2023) and we discuss the role of time within the modelling of maternal and newborn health in the sections (i) Maternal and perinatal health and (ii) Maternity health service unitisation and delivery. Within the supplementary material we provide a detailed overview of how risk of complications/outcomes is applied to pregnant women as the simulation moves forward in time (Figures S1 and S2). However, due to the number of models constituting the TLO framework, and the events-based structure of the model there is not one fixed time-step which can be reported in the paper. We have extensively referenced the documentation/papers detailing other TLO model components where readers can explore the relevant time-steps used within these models.

Reviewer #5 (Remarks to the Author):

I co-reviewed this manuscript with one of the reviewers who provided the listed reports. This is part of the Nature Communications initiative to facilitate training in peer review and to provide appropriate recognition for Early Career Researchers who co-review manuscripts.

We thank the Reviewer for their time reviewing our manuscript and their comments which we have addressed above.

Reviewer #3 (Remarks to the Author):

Thank you for the detailed response, which addressed many of our concerns. However, we still have some reservations regarding the methodology, as outlined below.

We thank the reviewer for their time in reviewing our manuscript again. We have responded to the Reviewer's additional comments below.

For Comment 1, the authors explained that "The MMR value during the initial year of simulation (2010) is inflated because pregnancies are only modelled to occur as the simulation progresses, resulting in fewer births in that year." However, it remains unclear why fewer births in that year would result in a higher MMR value.

More importantly, the quality of model calibration is predominantly influenced by technical aspects, specifically the calibration targets, parameters, and the loss function. While this may not be the authors' primary area of expertise, the observed calibration issues warrant a deeper investigation to identify the root cause of the suboptimal performance.

Thank you for your comment. In response to your first point we apologise for any confusion. As each simulation is initialised on 01/01/2010 we treat this first simulated year as a 'burn-in' period. This is a frequently applied procedure in simulation studies in which we allow for outputs from the model to stabilize. In our study, results in 2010 are not stabilized because women only become pregnant after the simulation has started and no women begin the simulation pregnant. We understand that presenting the results for 2010 in Figure 1, and some supplementary figures, has led to confusion (which we have now amended as described below).

To clarify our previous comments, as requested in response to Minor Comment 3, we previously added table S61 to the supplementary material which details the number of births and deaths generated by the model during the calibration period. In this table you will see that in 2010 there were 200, 658 births and 1715 maternal deaths leading to an MMR of 855 per 100,000 live births. In 2011 the number of births increased (529,544) along with the number of deaths (3113) as expected – here the MMR stabilises to 588 per 100,000 live births. We would therefore clarify our initial response – both the denominator and the numerator are reduced in 2010 (due to pregnancies only occurring after the simulation starts on 01/01/2010) however only 37% of the number of births occurring in 2011 occurred in 2010 whilst 55% of the deaths observed in 2011 occurred in 2011 leading to an inflated MMR value. Importantly, we can infer, by reviewing Figure

S33, that this is due to a greater number of deaths from indirect causes given the direct MMR is only marginally higher than the 2011 value in this figure.

We would once again highlight; indirect deaths are caused by other disease models within the Thanzi La Onse (TLO) framework and each model which generates indirect deaths (e.g. HIV) is rigorously calibrated to epidemiological and health systems data (Hallett et al. and Mangal et al. (referenced below)). Additionally, as evident from figure S33 the model is extremely well calibrated to direct obstetric mortality (mortality driven by complications in the MPHM described in this manuscript) from 2011 onwards.

We agree with the reviewer that we could improve clarity to this effect. To avoid further confusion for readers we have now:

1. Removed model outputs from 2010 in Figures 1A, 1C and 1E.
2. Added a sentence to Figure 1's legend explaining that 2010 is a burn in period and that data from the 2010 DHS is plotted in the year 2011
3. Added a sentence to the section *Model calibration* in the methods section and *4.2.3 Additional model calibration* results of the supplementary material explaining that 2010 is part of the initial burn in period and what this means in practice
4. Update the legend of table S61 explaining why there are fewer births and deaths in the first year of the simulation
5. Removed any plots using 2010 data from the supplementary material replacing them with outputs from 2011

Regarding your second point, we respectfully disagree that the model calibration requires deeper investigation or is sub-optimal. Within the supplementary material (Section 4.2.2) we provide a detailed overview of the calibration procedures including how and why calibration targets were selected (summarised in Table S60), how parameter values were determined (Tables S8 to S11, S14-S19, S23, S25-26, S28-29, S31-32, S34, S36-37, S39-59), how we assessed goodness-of-fit to calibration targets and why we were unable to take an algorithmic approach such as Approximate Bayesian Computation (section 4.2.2.2.2). Importantly, in addition to Figure 1, we demonstrate in Figures S33-S79 the model is fit well to data estimating mortality rates, coverage of key health services, and incidence of key health conditions. Please see our comment above regarding figure S33.

We believe in our previous response (and the clarification above) we have highlighted why there is not complete agreement between the model output and the primary calibration targets (DHS data) for maternal and neonatal mortality and believe that by present multiple estimates for mortality rates from different sources (DHS, GBD, WHO) we demonstrate that there is not complete agreement in the estimates of maternal mortality for Malawi in that time period. This is due to limited reliable population level data (beyond the DHS). Finally, we would once again highlight that in 2023, before the intervention period begins, there is good agreement between the model output and calibration data.

References:

Hallett TB, Mangal TD, Tamuri AU, Arinaminpathy N, Cambiano V, Chalkley M, et al. Estimates of resource use in the public-sector health-care system and the effect of strengthening health-care services in Malawi during 2015-19: a modelling study (Thanzi La Onse). Lancet Glob Health [Internet]. 2024. ISSN 2214-109X. Available from: https://doi.org/10.1016/S2214-109X(24)00413-3

Mangal T, Mohan S, Colbourn T, Collins JH, Graham M d, Jahn A, et al. Assessing the effect of health system resources on HIV and tuberculosis programmes in Malawi: a modelling study. Lancet Glob Health [Internet]. 2024;12:10. Available from: https://doi.org/10.1016/S2214-109X(24)00259-6

For Comment 2, considering that the TLO modeling framework was developed by a team of researchers, it is acceptable that its initiation is not described in detail within this manuscript. However, the authors should explicitly acknowledge the contributions of that team to this work. By the way, it seems like to draw legs on a snake for reply “Importantly, ... intervention period (2023)”, because refining the initiation is to ensure the model simulates from an accurate startline, not to reflect future scenarios

The developers of each component of the TLO are included as co-authors of this manuscript. The first author (JHC) had primary responsibility for the development of the maternal and newborn health model described in this manuscript. The manuscript and the supplementary material provide clear links to the TLO website and all additional publications in which the primary authors of the components of the model are clearly identified.

Furthermore, we agree with the reviewer that ensuring the simulated population at initialisation of the model is reflective of the population of Malawi is vital hence why the model is carefully calibrated to data estimates from 2010 - 2022 (please see Hallet et al. (reference above) and https://www.tlomodel.org/writeups.html for further information). We would reiterate again that in 2010 there is a 'burn-in' period given women are not initialised as pregnant when simulations start on 1/1/2010 and must go through conception and the start of pregnancy such that pregnancies are only reflective of the population of Malawi towards the end of 2010. We do not present results from 2010 for this reason and that the characteristics of the baseline population are not reflective of the population at the start of the intervention period.

For Comment 4, it would be a meaningful contribution if the authors focused on presenting the potential health gains that could be achieved during the period from 01/01/2023 to 31/12/2030. Thanks for your responses.

Thank you for your comment. In this study we describe in detail the impact of the proposed interventions on health during this time (01/01/2023 to 31/12/2030) including on morbidity, mortality and DALYs. We do not attempt to predict improvements in population health which may occur during the 2023-2030 period that are not directly associated with the modelled intervention. Whilst we appreciate that there are very likely to be factors which have a net positive impact on population health during this time, these are unknown and therefore not captured in the model. We strongly believe this does not affect our conclusions as, most importantly, any 'predicted' improvements in population health not driven by changes in the interventions modelled would be assumed to happen equally within each of the modelled scenarios – this would not therefore impact the difference between a given scenario and the status quo for any of the outcomes of interest (i.e. mortality) as is reported here.

To aid interpretation of our results we have added a sentence to the methodology (section *Analysis plan - estimating the impact of maternity service delivery*) to that effect.

For Comment 5, it is commendable to highlight the names of each parameter derived through calibration. However, we could not locate these changes in the appendix. Could the authors consider adding a separate table to clearly list the calibrated parameters for better clarity?

We thank the reviewer for their comment. We have reviewed the supplementary

material and at line 599 can confirm that we had previously added the sentence "*Finally, the names of any parameters which have been estimated through the process of calibration have been underlined within the relevant parameter table with additional information provided in the parameter description*" in section 1.2 and have checked that the relevant parameters have been underlined in their associated tables.

To improve clarity further we have now highlighted the relevant parameters in yellow and updated the above sentence accordingly.

For Minor Comment 4, although the model may include various modules with differing model cycles, or some may utilize event-based structures, we believe it is essential to clarify these elements. Specifically, it would be helpful to detail what modules are included, their respective cycles, and the type of structure they use. For example, as referenced in your response to Comment 3, the parameters in the contraception model are applied on a monthly timestep throughout the modeled time horizon.

We thank you for your comment. Please see our response to comment 2. We have comprehensively referenced all relevant documentation to all of the modules of the TLO model which is available open access allowing readers to review. Additionally, for those interested we provide in the reference list a link to the model code in its entirety which can be reviewed by readers. We feel we have described the MNHM in significant detail across the manuscript and supplementary material and sufficiently described the relationship between the MNHM and core components of the TLO model.

Reviewer #5 (Remarks to the Author):

I co-reviewed this manuscript with one of the reviewers who provided the listed reports. This is part of the Nature Communications initiative to facilitate training in peer review and to provide appropriate recognition for Early Career Researchers who co-review manuscripts.

We thank the Reviewer for their time reviewing our manuscript and their comments which we have addressed above.
