# Supplementary material for: An individual-based modelling study estimating the impact of maternity service delivery on health in Malawi
Source: Nat Commun. 2025 Apr 25;16:3925. doi: 10.1038/s41467-025-59060-2 (PMC12032021; doi:10.1038/s41467-025-59060-2)
Supplement: Supplementary file 4 — Supplementary Information [file 41467_2025_59060_MOESM4_ESM.pdf]

nature portfolio

Peer Review File

An individual-based modelling study estimating the impact of maternity service delivery on health in Malawi

Corresponding Author: Dr Joseph Collins

This file contains all reviewer reports in order by version, followed by all author rebuttals in order by version.

Version 0:

Reviewer comments:

Reviewer #1

(Remarks to the Author)
Thanks for the opportunity to review this paper. I found it to be well-written, clear, and the topic is interesting. However I am not sure whether there is a substantially new contribution to the literature, as it identifies important challenges (utilization alone is insufficient; it will be very hard -- if not impossible -- for many countries to meet their SDG targets; etc.) that have already been established in other papers. I feel that this particular analysis would be informative to policymakers in Malawi, but was not convinced of its broader impact. I do feel that a formal analysis of the trade-offs of pursuing investments into approaches that increase coverage vs quality for different groups (for example, "should" we invest in option A to increase coverage of interventions for neonates vs option B to improve quality of interventions for mothers) could be useful, but this would constitute an entirely different analysis (cost-benefit or similar) and a new paper, in my opinion.

(Remarks on code availability)

Reviewer #2

(Remarks to the Author)
The manuscript presents a detailed and comprehensive stochastic individual-based simulation model of maternal and perinatal health in Malawi. Notable results include the finding that current service delivery averts a significant number of maternal and neonatal deaths and DALYs. The model predicts substantial improvements in population health if higher coverage of high-quality services is achieved. Of particular interest is the finding that no single scenario alone was sufficient to meet SDG target 3.1, highlighting the need for a system-wide approach to improve maternal health.

The conclusions and claims made in the manuscript are generally well-supported by the simulation results, the methodology is sound and described in detail. The supplementary material is extensive

Recommendations (all minor):
- In Table 5, add a column which shows the SQ prevalence of all the interventions
- I find Figures 3 and 4 illegible. Consider alternate visualizations for these results.
- Figure 1a shows a sharp drop in the modeled maternal mortality ratio between 2014 and 2015 (and between 2010-2011). Is this drop primarily due to the difference in assumptions used between pre- and post- the Malawi 2014-2015 DHS? If not, what in the model most explains these drops?
o If it is due to the difference in assumptions, consider estimating an odds ratio that modifies the primary parameter (i.e. odds_will_attend_pnc) by year of birth in addition to the socio-demographic parameters, this would potentially lead to smoother estimates.
- The supplement notes that the referral process is only in place for individuals who deliver at a health center and require a CEmONC intervention. However, if a needed BEmONC intervention is modeled as not being available because of lack of health worker competence or lack of necessary consumables, shouldn't this also trigger a referral process?
- How do the assumptions and results compare with those in the Lives Saved Tool?
- Perhaps it is included somewhere in the Appendix, but it is unclear to me if or how the probability of care-seeking for intrapartum and postpartum care is informed by care-seeking and the quality of the care received in the prior stages (antenatal and intrapartum care). This should be made more explicit in the methods section.

- Are probabilities of health worker competence and consumable availability independent or conditional on one another? I.e. if health worker is not trained in providing one intervention, is it more likely they are not trained in another?
- In the limitations section, examples of missing maternal and perinatal health conditions and interventions are clearly listed. However, it is worth noting more broadly that the model is by necessity a simplification of reality and cannot incorporate all possible parameters. Beyond those listed, the lack of consideration of public versus private facilities, the degree of delay depending on transportation availability, interventions such as counseling (which may affect other parameters such as IFA adherence), etc, are not considered.
- Given the emphasis on the 'whole system', it seems worth developing a scenario in which all three interventions are set to have an increase in coverage and quality.

(Remarks on code availability)

Version 1:

Reviewer comments:

Reviewer #1

(Remarks to the Author)

(Remarks on code availability)

Reviewer #2

(Remarks to the Author)
Thank you for the revision to this manuscript. I have found the revisions, particularly the inclusion of the additional concurrent scenarios, to have substantially strengthened the conclusions of study. The authors have adequately addressed all of my concerns and I have no additional feedback.

(Remarks on code availability)

Reviewer #3

(Remarks to the Author)
General Comments:
Thank you for the opportunity to review this paper. We believe it presents an excellent individual-based simulation model, meticulously designed to assess the impact of improving antenatal, intrapartum, and postnatal services in Malawi on reducing maternal and neonatal mortality rates, aligned with the SDG 3.1 and 3.2 goals. The results clearly demonstrate the positive health effects of the current coverage of these services in Malawi and predict that improving coverage could lead to a maximum reduction in stillbirth, maternal, and neonatal mortality rates by 10%, 52%, and 57%, respectively. The topic is highly relevant, and the paper is well-organized and well-written. We are pleased to provide comments that we hope will further enhance this valuable study. Below are our detailed comments.

Major comments:

Comment 1: Regarding the intuitive impression, we believe the model calibration does not perform well for maternal and neonatal mortality rates as shown in Figure 1. The maternal mortality rate for 2010 seems to be overestimated, while the rate for 2015 is underestimated. Similarly, neonatal mortality rates are consistently underestimated. These two calibration targets are crucial since they represent key model outputs. Poor calibration in these areas raises concerns about the reliability and accuracy of the predicted model outcomes.

Comment 2: For an individual-based simulation model, it can accurately represent each individual in the system along with their characteristics and event occurrences throughout the simulation process. However, this approach requires highly precise data to initialize the status of individuals at the model's start. For instance, the authors mention that the model's individuals are characterized by demographic (e.g., age, sex, region of residence) and lifestyle (e.g., smoking status) attributes (Methods, third paragraph). Could the authors please clarify how these characteristics were assigned to the individuals within the model? Additionally, readers would benefit from a more detailed explanation of this process, particularly regarding the data sources used for sampling each characteristic and the results of these sampling procedures.

Comment 3: We believe the authors should clarify the nature of the simulated population in the model. Is the simulation based on the entire population or only on pregnancies? If it encompasses the entire population, how did the authors model the occurrence of pregnancies during each model cycle throughout the time horizon? On the other hand, if the simulation focuses solely on pregnancies, it implies that the incidence of pregnancy is modeled as independent for each year. This would suggest that the results are generated on a year-by-year basis, where differences across years would be influenced

only by the parameters specific to each year, with no continuity between them. In this case, the authors should provide a detailed presentation of the parameter differences across years in the supplementary material, while clearly indicating the corresponding differences in the output results.

Comment 4: The authors applied three sets of parameters during three distinct phases (2010-2014, 2015-2022, and 2023-2030), with corresponding results illustrated in Figure S80. From an intuitive perspective, the outcomes resemble a three-stage function, particularly in the predicted phases where the outcomes appear quite stable. Based on this, one could argue that if the intervention achieves the SDG goals by 2030, it would also achieve them by 2023, which raises questions about the model's progression. While this may be reasonable within the confines of the simulation, it is difficult to justify such an outcome in real-world scenarios. This undermines the objective of achieving reductions of maternal and neonatal mortality rates by 2030, potentially rendering the study's goal of achieving reductions by that year meaningless. We recommend that the authors recheck these parameter sets and reassess whether the model assumptions are truly appropriate and reflective of real-world dynamics.

Comment 5: The authors performed model calibrations. From a technical perspective, it would be helpful if the authors could clearly present the estimated parameters obtained during this calibration process, distinguishing them from the parameters sourced from other references. This would provide clarity on which parameters were derived through calibration versus those sourced externally, offering a more comprehensive understanding of the model's foundation and how it was fine-tuned to fit the data.

Minor comments:
1. The authors referred to SDG goals 3.1 and 3.2, citing reference 34 (https://www.unfpa.org/resources/transforming-our-world-2030-agenda-sustainable-development). As per the reference, the exact targets are:
"3.1 By 2030, reduce the global maternal mortality ratio to less than 70 per 100,000 live births.
3.2 By 2030, end preventable deaths of newborns and children under 5 years of age, with all countries aiming to reduce neonatal mortality to at least as low as 12 per 1,000 live births and under-5 mortality to at least as low as 25 per 1,000 live births."

However, in the Results section (ii), second paragraph, the authors state: "As evident from Table 2, only the scenario 'All services max.' led to an average MMR during the intervention period below the SDG 3.1 target of 140 maternal deaths per 100,000 live births." This threshold differs from the official SDG 3.1 target of less than 70 maternal deaths per 100,000 live births. If there are specific reasons for using a different threshold, the authors should clarify them to avoid confusion for the readers and ensure alignment with SDG targets.

Moreover, the SDG 3.1 target specifically aims to reduce the maternal mortality ratio (MMR) by 2030. However, the authors report the *average* MMR during the period from 2023 to 2030 as their main result. It would be helpful if the authors could clarify why they focus on the average over this period, rather than reporting the MMR specifically in the year 2030, as the SDG target is for that specific year. This distinction is important to ensure that the study's findings align more closely with the actual SDG goals.
2. As Harold C. Sox mentions in *Medical Decision Making* (3rd edition), "The terms calibration and validation are not used consistently in the literature; although they are sometimes used interchangeably, they are not synonymous. We won't dive deeper into these two terms because they can be hard to remember unless you actually need to use them." In light of this, the authors should strive to use terminology that is appropriate and consistent throughout the manuscript to enhance the reader's understanding and experience.
3. The authors calculated the total burden of maternal and neonatal health in Malawi, which depends on the number of pregnancies each year as well as the model's initialization parameters. Please provide the detailed numbers used in these calculations to enhance the transparency and reproducibility of the study.
4. Please provide the detailed model procedures for the model cycle and time horizon. This information will help clarify how the model operates over time and how different phases are implemented within the simulation.

(Remarks on code availability)

Reviewer #5

(Remarks to the Author)
I co-reviewed this manuscript with one of the reviewers who provided the listed reports. This is part of the Nature Communications initiative to facilitate training in peer review and to provide appropriate recognition for Early Career Researchers who co-review manuscripts.

(Remarks on code availability)

Version 2:

Reviewer comments:

Reviewer #3

(Remarks to the Author)
Thank you for the detailed response, which addressed many of our concerns. However, we still have some reservations regarding the methodology, as outlined below.

For Comment 1, the authors explained that “The MMR value during the initial year of simulation (2010) is inflated because pregnancies are only modeled to occur as the simulation progresses, resulting in fewer births in that year.” However, it remains unclear why fewer births in that year would result in a higher MMR value. More importantly, the quality of model calibration is predominantly influenced by technical aspects, specifically the calibration targets, parameters, and the loss function. While this may not be the authors’ primary area of expertise, the observed calibration issues warrant a deeper investigation to identify the root cause of the suboptimal performance.

For Comment 2, considering that the TLO modeling framework was developed by a team of researchers, it is acceptable that its initiation is not described in detail within this manuscript. However, the authors should explicitly acknowledge the contributions of that team to this work. By the way, it seems like to draw legs on a snake for reply “Importantly, ... intervention period (2023)”, because refining the initiation is to ensure the model simulates from an accurate startline, not to reflect future scenarios

For Comment 3, it is commendable that the probability of pregnancy in the simulated population is derived from the contraception model within the TLO framework. Thank you for providing a brief description to clarify this aspect.

For Comment 4, it would be a meaningful contribution if the authors focused on presenting the potential health gains that could be achieved during the period from 01/01/2023 to 31/12/2030. Thanks for your responses.

For Comment 5, it is commendable to highlight the names of each parameter derived through calibration. However, we could not locate these changes in the appendix. Could the authors consider adding a separate table to clearly list the calibrated parameters for better clarity?

For Minor Comment 4, although the model may include various modules with differing model cycles, or some may utilize event-based structures, we believe it is essential to clarify these elements. Specifically, it would be helpful to detail what modules are included, their respective cycles, and the type of structure they use. For example, as referenced in your response to Comment 3, the parameters in the contraception model are applied on a monthly timestep throughout the modeled time horizon.

(Remarks on code availability)

Reviewer #5

(Remarks to the Author)
I co-reviewed this manuscript with one of the reviewers who provided the listed reports. This is part of the Nature Communications initiative to facilitate training in peer review and to provide appropriate recognition for Early Career Researchers who co-review manuscripts.

(Remarks on code availability)

Version 3:

Reviewer comments:

Reviewer #3

(Remarks to the Author)
The author has addressed our concerns. We appreciate the detailed response.

(Remarks on code availability)

Reviewer #5

(Remarks to the Author)
I co-reviewed this manuscript with one of the reviewers who provided the listed reports. This is part of the Nature Communications initiative to facilitate training in peer review and to provide appropriate recognition for Early Career Researchers who co-review manuscripts.

(Remarks on code availability)

Open Access This Peer Review File is licensed under a Creative Commons Attribution 4.0 International License, which permits use, sharing, adaptation, distribution and reproduction in any medium or format, as long as you give appropriate credit to the original author(s) and the source, provide a link to the Creative Commons license, and indicate if changes were made.
In cases where reviewers are anonymous, credit should be given to 'Anonymous Referee' and the source.
The images or other third party material in this Peer Review File are included in the article's Creative Commons license, unless indicated otherwise in a credit line to the material. If material is not included in the article's Creative Commons license and your intended use is not permitted by statutory regulation or exceeds the permitted use, you will need to obtain permission directly from the copyright holder.
To view a copy of this license, visit https://creativecommons.org/licenses/by/4.0/
